# Translation readiness of model-based synthetic tabular data in healthcare: a systematic review and governance audit

**DOI:** 10.1093/jamia/ocag102

**Published:** 2026-06-18

**Authors:** Simone Castagno, Alagu Subramanian, Ilias E Epanomeritakis, Benjamin Gompels, Stephen McDonnell, Mark Birch, Mihaela van der Schaar, Andrew McCaskie

**Affiliations:** Department of Surgery, University of Cambridge, Cambridge, CB2 0QQ, United Kingdom; Department of Surgery, University of Cambridge, Cambridge, CB2 0QQ, United Kingdom; Department of Surgery, University of Cambridge, Cambridge, CB2 0QQ, United Kingdom; Department of Surgery, University of Cambridge, Cambridge, CB2 0QQ, United Kingdom; Department of Surgery, University of Cambridge, Cambridge, CB2 0QQ, United Kingdom; Department of Surgery, University of Cambridge, Cambridge, CB2 0QQ, United Kingdom; Department of Applied Mathematics and Theoretical Physics, University of Cambridge, Cambridge, CB3 0WA, United Kingdom; Department of Surgery, University of Cambridge, Cambridge, CB2 0QQ, United Kingdom

**Keywords:** synthetic data, generative AI, systematic review, governance and translation readiness, privacy

## Abstract

**Objectives:**

To evaluate the clinical applications and translation readiness of model-based synthetic tabular data in healthcare, and identify gaps in governance reporting that may hinder translation.

**Materials and Methods:**

We systematically searched Ovid MEDLINE and Embase (2010-August 2025; PROSPERO: CRD42025635514) for studies that generated and applied model-based synthetic tabular data in clinical contexts. Screening used a “human-in-the-loop” large language model workflow alongside independent manual review, achieving 100% sensitivity for included studies. Unlike prior reviews focused primarily on evaluation methodology, we mapped use-cases and deployment paradigms, and audited translation-readiness reporting using a predefined governance framework (validation depth, privacy, fairness, regulatory alignment).

**Results:**

Thirty-seven studies (2019-2025) were included. GANs predominated; other approaches included VAEs, diffusion models, LLM-based synthesis, and Bayesian networks. Dataset augmentation was the primary application, often improving downstream model performance for rare outcomes. Emerging applications included synthetic control cohorts and algorithmic bias mitigation. Translation-readiness reporting was limited: 34/37 studies (92%) relied solely on internal validation, 9/37 (24%) used formal privacy models, 6/37 (16%) reported explicit fairness evaluations, and 6/37 (16%) addressed regulatory alignment. Few studies distinguished “no-release” from “delayed-release” paradigms.

**Discussion:**

A systemic gap exists between methodological innovation and deployment-readiness reporting. Model-based synthetic data show clear value for augmentation and class balancing, but inconsistent reporting of validation, privacy, fairness, and regulatory considerations limits confidence in clinical deployment.

**Conclusion:**

We propose TRUST-SD (Transparency and Reporting for Utility, Safety, and Translation of Synthetic Data), an author-derived, preliminary, evidence-informed reporting checklist spanning 7 domains, as a starting point for community refinement and consensus-building.

## Introduction

### Background and significance

Healthcare’s digital transformation has generated unprecedented clinical data volumes, increasing the potential for personalized medicine and optimized treatment.^1^ However, realizing these opportunities faces significant obstacles, including data scarcity for rare conditions, the challenge of creating diverse datasets for robust predictive models, and stringent privacy regulations that limit data sharing.^2^^,^^3^

Synthetic data preserve source-data structure without exposing original records and are increasingly used for healthcare research, model development, and privacy-preserving analytics.^4^ Unlike traditional anonymization techniques that may retain re-identification risk, synthetic data generation creates new records that reflect the statistical properties of source data and may reduce direct linkage risk.^5^ However, residual disclosure risk can remain, particularly through rare categorical combinations, quasi-identifiers, small cells, outlier records, and potential memorization by generative models.^5^^,^^6^ Recent advances in machine learning (ML), particularly in generative models such as Generative Adversarial Networks (GANs) and Variational Autoencoders (VAEs), have enabled the creation of highly realistic synthetic datasets that can augment existing data, balance underrepresented populations, and facilitate research where real-world data are scarce.^7^^,^^8^

Prior reviews have primarily analyzed how synthetic data are evaluated, rather than *where* and *why* they are used in clinical workflows.^9–11^ We therefore apply a clinical-application lens restricted to model-based synthetic tabular data: structured datasets organized in rows (patients) and columns (variables), distinct from images, free text, or time-series waveforms. Within this scope, we map dominant use-cases and intended deployment paradigms, and audit translation-readiness reporting (including validation depth, privacy threats, fairness, and regulatory considerations) using a predefined framework. On this basis, we propose TRUST-SD (**T**ransparency and **R**eporting for **U**tility, **S**afety, and **T**ranslation of **S**ynthetic **D**ata), an evidence-informed reporting checklist to improve transparency and enable context-specific risk-benefit assessment.

## Materials and methods

### Protocol and registration

This systematic review was conducted and reported in accordance with the Preferred Reporting Items for Systematic Reviews and Meta-Analyses (PRISMA) 2020 statement.^12^ The protocol was registered with PROSPERO (ID: CRD42025635514) in January 2025.

### Eligibility criteria

Eligible studies were original, peer-reviewed, quantitative research in English published from 2010 onwards that generated model-based synthetic tabular human clinical data and applied it to a clinical or health use case with empirical, quantifiable outcomes. Eligible clinical applications included diagnostic support, prognostic modeling, treatment planning, patient safety, trial design or other outcomes directly linked to patient care.

We defined model-based synthetic tabular data as structured patient-level or cohort-level data generated using AI, ML, or related computational generative methods. Eligible methods included GANs, VAEs, diffusion models, and LLM-based synthesis, Bayesian networks trained generatively, copula-based models, synthpop, and proprietary platforms such as MDClone. We excluded studies using only SMOTE, ADASYN, or purely statistical simulation without a generative model; comparator-only use of SMOTE/ADASYN was allowed.

We excluded reviews, comments, conference abstracts, letters, editorials, preprints, non-tabular data, non-human or non-clinical data, privacy-only or methods-only studies without a clinical application, and studies that only reused preexisting synthetic datasets without new synthesis. Full peer-reviewed conference papers were eligible if they were indexed in MEDLINE or Embase and met all other inclusion criteria.

### Information sources and search strategy

A comprehensive literature search was performed in Ovid MEDLINE(R) and Embase on August 7, 2025 for English-language records published from 2010 onwards. The search combined 3 concept blocks: synthetic data; AI, ML, and computational generative methods; and healthcare or clinical application terms. The full Ovid syntax is provided in Supplementary File S1.

### Selection process

We adopted a hybrid screening strategy comprising AI-assisted and manual review. The Elicit platform (https://elicit.com/) generated a relevance score for each record^13^; a threshold of >4 was selected for flagging inclusion. Independently, all papers were manually reviewed at the title and abstract stage by 3 reviewers (I.E.E., B.G., and A.S.), blinded to Elicit scores. Disagreements were resolved through discussion and consensus, with SC available for arbitration.

All records passing either Elicit scoring (>4) or manual evaluation underwent full-text review by IEE, BG, and AS, with SC resolving conflicts. We recorded whether included studies were prioritized by Elicit to quantify sensitivity for the final included set.

### Data extraction and synthesis

We utilized NotebookLM (https://notebooklm.google.com/), an AI-powered research assistant that grounds its responses in user-provided source documents,^14^^,^^15^ to extract study characteristics, clinical application details, technical methods, outcome metrics, and author-reported findings. NotebookLM was selected for extraction because its retrieval-augmented generation architecture grounds outputs in specific source passages,^14^^,^^15^ facilitating source-anchored human verification.

Authors BG, IEE, AS, and SC checked extracted fields against source articles, resolving discrepancies by consensus. Discrepancies were infrequent and predominantly involved minor numerical inconsistencies rather than substantive errors. Because all fields were verified prior to analysis, any NotebookLM errors identified during extraction were intercepted before analysis.

Given the anticipated heterogeneity in clinical domains, synthetic data methods, and outcome measures, we planned a narrative synthesis grouped by clinical domain and application type.

### Assessment of translation-readiness and governance reporting

Before data extraction, we developed a predefined translation-readiness and governance framework from regulatory and methodological literature.^5^^,^^16–18^ After applying this framework to the included studies, we derived TRUST-SD by mapping observed reporting gaps onto these domains and refining them into reporting items where supported by the evidence (Discussion and Panel 1). For each study, 2 reviewers (A.S. and I.E.E.), with SC resolving disagreements, scored 5 domains on a 3-level ordinal scale (0 = absent; 1 = partial; 2 = explicit/substantial): intended clinical use; validation depth (internal, external, or prospective); privacy model (none, *ad hoc*, or formal); fairness evaluation; and regulatory relevance.

As a *post hoc* quality-control step, SC performed a full-text audit of all included studies against the 4 core governance fields: validation, privacy, fairness, and regulatory alignment. This confirmed 4 discussion-level omissions across 148 governance-reporting cells (2.7%). Correcting these items did not alter the headline counts for external validation, formal privacy modeling, explicit fairness evaluation, or explicit regulatory alignment.

### Quality assessment

Given the predominantly computational nature of the included research, standard risk-of-bias tools such as QUADAS-2 or PROBAST were not applied because most studies did not evaluate a diagnostic or prognostic model in the conventional clinical-study sense, and many focused on synthetic-data generation, augmentation, or utility benchmarking. Instead, we conducted a structured content synthesis of author-reported opportunities and limitations, grouping recurring content into categories such as bias propagation, generalizability, interpretability, technical gaps, standardization, and regulatory uncertainty.^19^ This approach characterizes the field’s maturity but may underestimate weaknesses because authors may underreport unfavorable findings.

## Results

### Study selection

The search yielded 781 records; 281 duplicates were removed, leaving 500 for parallel title/abstract screening by Elicit (scores > 4) and blinded manual review by IEE, BG, and AS. Elicit identified 53 records and manual screening 62, with 53 common to both, yielding 62 unique articles for full-text review.

Thirty-seven studies^20–56^ met eligibility criteria, all captured by Elicit (100% sensitivity). Although we did not formally track time or cost, AI-assisted screening and extraction required <5 minutes of compute time, versus ∼10-15 person-days for manual screening and extraction by 3 reviewers.

*Post hoc* review of the 500 screened records identified one excluded study using a preexisting public synthetic tabular healthcare dataset for clinical prediction and 5 further records using existing synthetic/simulated datasets or benchmarks outside scope. Within the screened MEDLINE/Embase corpus, we found no prospective, external, or real-world reevaluation of a previously published synthetic tabular healthcare dataset.

The PRISMA flow diagram (Figure 1) summarizes the study selection process, and Table S1 provides the screening results.

**Figure 1.**
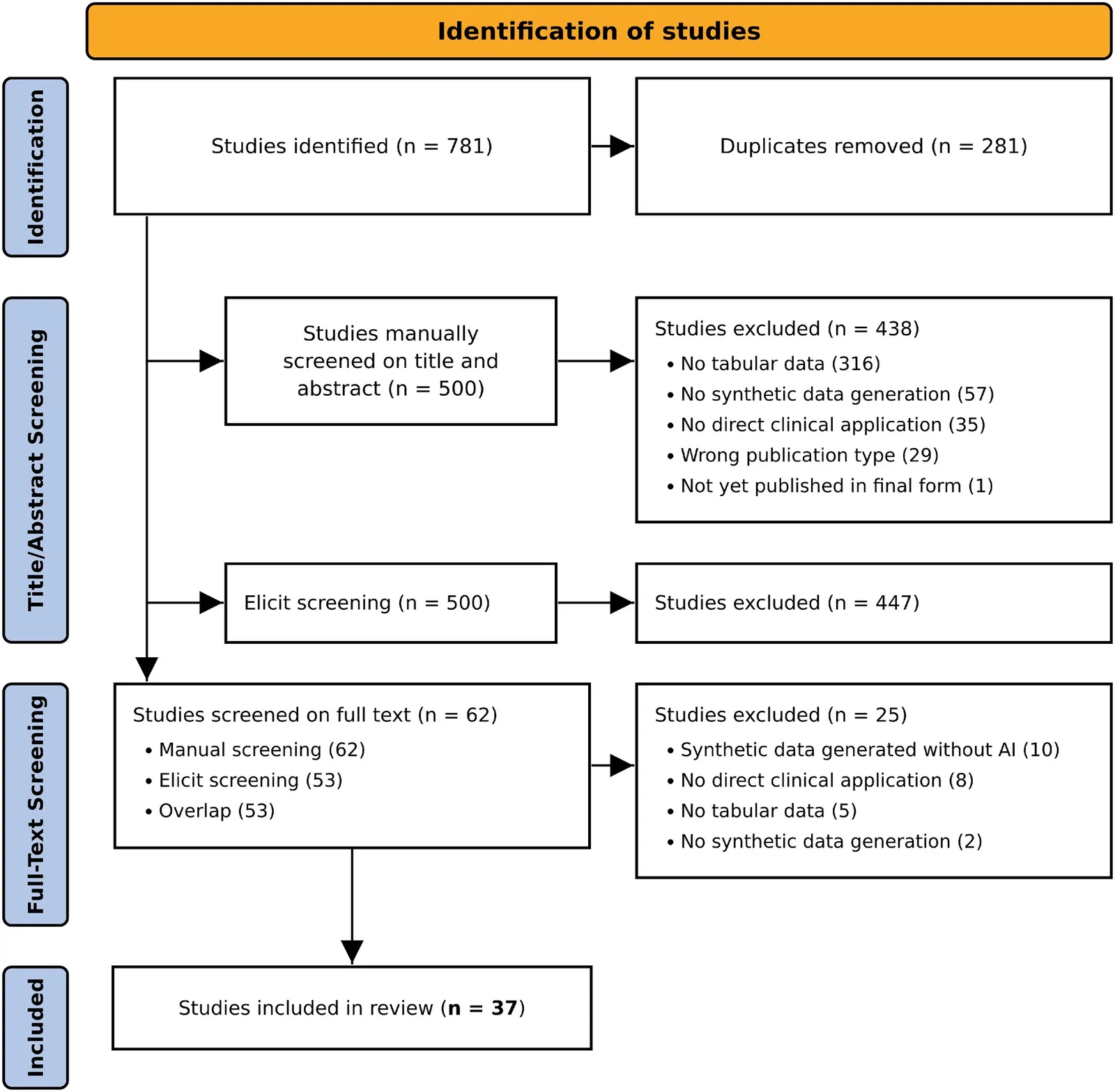
PRISMA flow diagram of article identification and selection. This diagram illustrates the flow of information through the different phases of the systematic review. It details the number of records identified, included, and excluded at each stage, including identification, screening, full-text assessment, and final inclusion in the narrative synthesis.

### Characteristics of included studies

Included studies were published between 2019 and 2025, with most appearing from 2023 onwards: 2019 (*n* = 1), 2021 (*n* = 2), 2022 (*n* = 1), 2023 (*n* = 12), 2024 (*n* = 9), and 2025 (*n* = 12) (Table S3; Figure S1). Designs were predominantly computational and retrospective; no RCTs were identified. In addition, 39 records published between 2010 and 2018 were screened and all were excluded: 30 because they did not use tabular data, 8 because they did not generate synthetic data, and one because it did not use an eligible AI, ML, or related computational generative method.

Clinical applications of model-based synthetic tabular data spanned diverse medical specialties (Table 1), with oncology and hematological malignancy predominant,^20–22^^,^^29^^,^^30^ alongside cardiology^24^^,^^27^ and critical care.^32^^,^^38^ Other significant areas included endocrinology,^54^ hepatology,^23^ and chronic diseases like HIV^43^ and Parkinson’s Disease.^51^

**Table 1.** Characteristics of the included studies.

Article	Study design	Clinical domain	Synthetic data generation method	Sample sizes (real and synthetic)
**Abbaszadeh Bavil Soflaei et al^20^**	Original Article	Breast cancer	SMOTE, ADASYN, CTGAN, ROS	**Real:** Wisconsin Breast Cancer Diagnostic (WBCD) dataset (569 instances: 357 benign, 212 malignant). **Synthetic:** Dataset balanced using various methods. 145 instances generated.
**Ahmadian et al^21^**	Retrospective cohort study; framework evaluation	Radiomics/radiogenomics (colorectal cancer, TP53 mutational status)	Tabular Preset, Gaussian Copula, TVAE, CTGAN, Copula GAN. SDV library used.	**Real:** 386 patients (2570 lesions total); training sets of 200, 400, 1000, 2055 lesions. **Synthetic:** Combined with real data at 1:5 stretch (synthetic is 5x real).
**Ahmed et al^22^**	Comparative analysis	Breast cancer prediction	CTGAN, TVAE, Gaussian Copula, Copula GAN. SDV Python module.	**Real:** WBCD dataset: 569 instances. **Synthetic:** 10 000 instances generated.
**Ahn et al^23^**	Original article; validation study	Hepatology (liver transplant waitlist)	Diffusion Model	**Real:** 38 183 patients (UNOS Liver Transplant Waitlist). **Synthetic:** 38 183 patients.
**Araújo et al^24^**	Benchmarking study	Doxorubicin-induced cardiotoxicity prediction	Data Augmentation and Smoothing (DAS). Benchmarked against SMOTE, ADASYN, SDV data generation methods.	**Real:** 78 breast cancer patients. **Synthetic:** DAS produced 4892 instances.
**Arora and Arora^25^**	Comparative analysis	Blood pressure prediction	*synthpop* library in R.	**Real:** Training dataset *n* = 2408. Testing dataset *n* = 424. **Synthetic:** Multiple datasets generated (*n* = 2408, *n* = 4816).
**Azizi et al^26^**	Validation study	Oncology (stage III colon cancer)	Sequential decision trees (conditional trees).	**Real:** 1543 patients. **Synthetic:** Generated for the 1543 patients.
**Bahudian and Valdovinos^27^**	Study to demonstrate generation and utility	Paediatric Cardiology (Fontan patients); predicting ventricular functional status	Tabular Generative Adversarial Network (tabGAN).	**Real:** 546 subjects (imputed); 286 subjects (dropped). **Synthetic:** 532 subjects (imputed); 219 subjects (dropped).
**Christoforou et al^28^**	Framework for generation, evaluation, selection	Radiation Oncology (various cancers)	Four generators implemented via SDV library (GANs, VAEs, copula-based modelling).	**Real:** 5 datasets: 1038, 117, 48, 1269, 353 patients. **Synthetic:** Multiple variants per model.
**D’Amico et al^29^**	Study to apply generative AI, develop validation framework	Haematology (MDS, AML)	Conditional GAN architecture (conditional Wasserstein GAN with gradient penalty).	**Real:** 187-2043 MDS patients depending on clinical application; 1002 AML patients. **Synthetic:** 100-300% augmented MDS cohort depending on clinical applications; 1002 AML patients.
**Eckardt et al^30^**	Study using generative AI methodologies	Acute Myeloid Leukaemia (AML)	CTAB-GAN+, Normalising Flows (NFlow).	**Real:** 1606 AML patients from 4 multicentre trials. **Synthetic:** 1606 patients each for CTAB-GAN+ and NFlow.
**Fisher et al^31^**	Approach to simulating disease progression	Precision medicine and clinical trial simulation (Alzheimer’s disease)	Conditional Restricted Boltzmann Machines (CRBMs).	**Real:** 18-month trajectories from 1909 patients. **Synthetic:** ∼190 900 synthetic trajectories (100 simulations per patient).
**Foraker et al^32^**	Validation workstream	COVID-19 (EHRs)	MDClone’s computationally derived data.	**Real:** 230 703 patients. **Synthetic:** 230 650 patients.
**Ganguli et al^33^**	Study to develop neural network	Metastatic cancer (postoperative)	Generative Recurrent Neural Network (LSTM).	**Real:** 167 474 metastatic cancer patients. **Synthetic:** Volume-matched (167 474 patients); Reduced-size (5000 patients).
**Ghareeb et al^34^**	Multicentre validation of AI platform	Acute appendicitis diagnosis (general surgery)	Generative Adversarial Network (GAN).	**Real:** 2579 patients. **Synthetic:** 9736 patients.
**Greenberg et al^35^**	Retrospective cohort study	Spine surgery (anterior cervical/posterior lumbar fusion)	MDClone platform (proprietary statistical/AI techniques).	**Real:** Data from 15 hospitals. **Synthetic:** MDClone creates datasets with ≥200 patients.
**Guillaudeux et al^36^**	Study presenting new method (“Avatar”)	Biomedical data analysis (clinical trial—AIDS data; cancer observational study—Wisconsin Breast Cancer data)	“Avatar” method (patient-centric). Compared with Synthpop and CT-GAN.	**Real:** AIDS clinical trial dataset included 2139 patients; Wisconsin Breast Cancer (Original) dataset included 683 observations. **Synthetic:** Overall size for assessment of synthetic data utility not explicitly stated. For privacy comparison between synthetic data generation methods, 10 synthetic datasets per method per use case were generated.
**Gurcan and Soylu^37^**	Empirical study	Breast cancer prediction	Generative Adversarial Networks (GANs).	**Real:** SEER breast-cancer dataset with 4024 patient records with 16 variables (alive: dead imbalance ratio ≈5.5). **Synthetic:** Generated minority-class samples to achieve a 1:1 ratio.
**Hameed and Alamgir^38^**	Research to devise scheme	Acute Pancreatitis (AP) mortality prediction	Advanced tabular GANs (CTGAN, TGAN, CTAB, CopulaGAN) and Variational Autoencoders (TVAE). SMOTE. SDV library.	**Real:** MIMIC-III (916), MIMIC-IV (4867), EICU-CRD (543). **Synthetic:** Datasets with 50:50 and 70:30 majority-minority class splits.
**Hsieh et al^39^**	Framework introduction and evaluation	Medical diagnostics (supplementing X-ray images with clinical context to improve diagnostic accuracy)	DALL-M framework [Large Language Models (LLMs)].	**Real:** MIMIC-IV dataset with 799 patient cases. **Synthetic:** DALL-M extended 8 original features per patient case to 78 features, then to 91 features with expert input.
**Jacobson et al^40^**	Study on dataset augmentation	Chronic Obstructive Pulmonary Disease (COPD), AECOPDs prediction.	synthpop R package.	**Real:** Not explicitly stated for overall size. Test set of 18 patients. **Synthetic:** 6 datasets (1000 to 10 000 records).
**Kim et al^41^**	Retrospective study	Early-Onset Colorectal Cancer (EOCRC) survival status prediction	Bayesian network-based synthesising model (DataSynthesiser) with differential privacy (DP).	**Real:** 1253 patients with 93 features. **Synthetic:** 5005 individuals.
**Koloi et al^42^**	Comparative analysis	Coronary artery bypass graft surgery (CABG)	Tabular Preset, Gaussian Copula, CTGAN, Copula GAN, TVAE.	**Real:** 224 patients **Synthetic:** 2000 patients.
**Kuo et al^43^**	Study extending GAN setup	HIV [Antiretroviral Therapy (ART)]	Extended GAN setup (WGAN-GP+GEOT+VAE+Buffer).	**Real:** 8916 patients; total 534 960 records (monthly data for 60 months). **Synthetic:** Not explicitly stated.
**Laatifi et al^44^**	Study on VAE network-based approach	COVID-19 severity (cytokine data)	Variational Autoencoder (VAE) network-based approach.	**Real:** 87 records and 48 attributes. **Synthetic:** 500 samples.
**Lin and Lyu^45^**	Comparative study	Intradialytic Hypotension (IDH) prediction	Conditional Wasserstein GAN (cWGAN-GP). Compared with ADASYN and SMOTE.	**Real:** “Original Training Data” (size not given). **Synthetic:** GAN Balanced (163 470 samples), GAN Augmented (191 712 samples).
**Mahmoud et al^46^**	Proposed novel visual learning approach	Healthcare (cancer, heart disease, diabetes, etc.)	Customized Generative Adversarial Network (GAN).	**Real:** Immunotherapy data (90 patients). **Synthetic:** Quantity varies as needed.
**Nagro^47^**	Proposed advanced ML framework	Monkeypox diagnosis (symptom-based)	Conditional Tabular GAN (CTGAN).	**Real:** 25 000 symptom-based records with 11 attributes. **Synthetic/augmented**: CTGAN-balanced dataset total 30 909 records (minority-class augmentation).
**Ogwel et al^48^**	Study on predictive modeling	Pediatric health (linear growth faltering for children with diarrhoea)	Synthpop R package.	**Real:** 1106 enrolled patients. **Synthetic:** 8894 samples generated. **Combined:** 10 000 samples.
**Paladugu et al^49^**	Secondary analysis, model development	Predicting Cerebrospinal Fluid (CSF) Pressure/Intracranial Pressure (ICP) regulation	LLM-aided GAN (GPT Neural Network LLM, Custom Three-Layer GAN).	**Real:** 29 miRNA, 13 mRNA paired samples. **Synthetic:** 250 samples generated.
**Sharma et al^50^**	Paper proposing phylaGAN for data augmentation	Microbiome-based disease prediction (Type 2 diabetes, liver cirrhosis)	Conditional GAN combined with an autoencoder (phylaGAN).	**Real:** T2D study—174 cases, 170 controls; cirrhosis study—118 cases, 114 controls; Le Chatelier et al. study (used for external validation) - 164 obese cases, 89 lean controls. **Synthetic:** 600 cases and 600 controls per study.
**Sood et al^51^**	Study on Bayesian Network modeling	Parkinson’s Disease (PD) risk and prodromal markers	Bayesian Network (BN) based representation.	**Real:** 1178 PD-free participants. **Synthetic:** 1178 synthetic participants generated.
**Theodorou et al^52^**	Study introducing FairPlay	Clinical outcome prediction (mortality prediction)	FairPlay [Large Language Models (LLMs)]. Compared with SMOTE, MCRAGE, SMA.	MIMIC-IV and eICU datasets. Specific sizes not given.
**Titar and Ramanathan^53^**	Tutorial	Drug dosing determinants in renal, hepatic, metabolic, and cardiac disease states	Variational Autoencoders (VAEs).	**Real:** 17 480 patients. **Synthetic:** Not explicitly stated for synthetic data size.
**Topşir et al^54^**	Study on thyroid disease classification	Thyroid disease classification	Generative Adversarial Networks (GANs). Compared with Oversampling, MixUp.	**Real:** 7200 individuals with 19 variables/class labels. **Synthetic:** Not explicitly stated.
**Tran et al^55^**	Study on predicting all-cause mortality	Chronic Kidney Disease (CKD) all-cause mortality	Bayesian imputation using Netica software (Bayesian network).	**Real:** 534 non-dialysis stage 4/5 CKD patients. **Synthetic:** 2000 synthetic patients generated for a balanced learning database.
**Yang et al^56^**	Study on synthesising feature-aligned and category-aware EHR data	Prediction of intracranial aneurysm (IA) rupture	TransCGAN—a Transformer-based conditional GAN.	**Real:** In-house intracranial aneurysm dataset (607 patients and 845 IAs) and a public heart failure survival dataset (299 patients). **Synthetic:** 600 synthetic samples merged with training data set.

This table provides a detailed summary of the 37 studies included in the review. For each article, it outlines the study design, the specific clinical domain of application, the synthetic data generation method used, and the respective sample sizes of the real and synthetic datasets used.

Real-world cohort sizes varied markedly, from small observational cohorts (*n* = 18^40^) to large EHR extracts (*n* > 230 000^32^). Synthetic datasets ranged from a few hundred rows to expansions exceeding the real cohort. Most studies generated data at similar scale or a few-fold larger than the real dataset, often producing class-balanced cohorts.

GANs and their variants (such as CTGAN, WGAN, and CopulaGAN) were the most common generation methods, followed by VAEs. Several studies benchmarked eligible generative methods against traditional oversampling methods such as SMOTE or ADASYN. Technical details are provided in Table 1.

### Clinical applications and performance

The primary motivations for generating synthetic data fell into 4 categories: (1) augmenting datasets to improve ML model performance, (2) enabling privacy-preserving data sharing, (3) supporting in-silico trials or synthetic control cohorts, and (4) mitigating algorithmic bias. Each study’s primary motivation category is indicated in Table S2.

Dataset augmentation was the most frequent application. Ahmadian et al^21^ demonstrated that augmenting a radiomics dataset with synthetic data to 5 times its original size improved AUC for predicting TP53 mutation status by up to 16.7%. Lin and Lyu^45^ reported that a GAN-balanced dataset for intradialytic hypotension prediction yielded significantly higher performance across all metrics (*P* < .001) than the original imbalanced dataset. However, performance depended strongly on the generation method: Ahmed et al^22^ found that a TVAE-generated dataset approached real-data test accuracy, whereas a Gaussian Copula-based model overfitted.

Privacy-preserving sharing was a second major use case. Azizi et al^26^ created a synthetic version of a phase III colon cancer trial dataset that directionally replicated the original study’s primary conclusions, with hazard ratio confidence intervals overlapping the original by 61% for overall survival and 86% for disease-free survival. Other privacy-preserving examples included shareable EHR data via MDClone,^32^^,^^35^ patient-centric Avatar generation,^36^ and organ-allocation modeling via diffusion models.^23^

Several studies proposed synthetic data for trial design or synthetic control cohorts. D’Amico et al^29^ used a conditional GAN to replicate trial endpoints for hematology studies, Fisher et al^31^ simulated Alzheimer’s disease progression and trial outcomes, and Eckardt et al^30^ explored synthetic acute myeloid leukemia trial cohorts.

Bias-mitigation applications were less common but clinically important. Kuo et al^43^ designed an extended GAN setup to mitigate mode collapse in antiretroviral-therapy data, while Theodorou et al^52^ introduced “FairPlay,” an LLM-based framework that improved mortality-prediction performance while reducing subgroup performance gaps.

### Performance and fidelity evaluation

Utility was typically assessed by comparing ML model performance when trained on synthetic versus real data, using metrics such as AUC-ROC, accuracy, and F1-score (Table S2). Fidelity was evaluated through statistical distribution comparisons (Kolmogorov-Smirnov, KL-divergence), correlation matrix comparisons, and dimensionality reduction visualizations. Several studies achieved high fidelity, with models unable to reliably distinguish synthetic from real data.^24^^,^^27^

### Translation readiness and governance characteristics

Clinical decision support was the predominant application (31/37; 84%), followed by trial design (24%), service planning (19%), and quality improvement (14%); domains were not mutually exclusive (Table 2). Table 3 provides a cross-tabulation of these 4 clinical application domains against the 4 primary motivation categories described earlier. In all 37 studies, the intended clinical use was indirect: synthetic data were used to train or validate predictive models that would subsequently support clinical decisions, rather than being consulted directly as a patient-level resource. Although 35 studies (95%) achieved the maximum rubric score for a clearly stated clinical purpose, these descriptions were typically high-level, lacking detail regarding specific workflows, users, or decision thresholds. Validation depth was limited: 34/37 studies (92%) relied solely on internal validation, only 3 (8%) reported any external validation on an independent dataset, and none undertook prospective evaluation in routine care. Treatment of privacy was heterogeneous: 15 studies (41%) reported no explicit privacy mechanism beyond basic access controls, 13 (35%) described operational safeguards or *ad hoc* statistical disclosure control, and 9 (24%) implemented formal approaches such as differential privacy, k-anonymity, or re-identification testing. Fairness evaluation was similarly sparse: 23 studies (62%) provided no group-stratified assessment, 8 (22%) offered only descriptive or indirect consideration of subgroup performance or representation, and 6 (16%) reported explicit subgroup or fairness metrics. The translation-readiness heatmap (Figure 2) shows persistent under-reporting across privacy, fairness and regulatory columns, despite well-specified clinical use.

**Figure 2.**
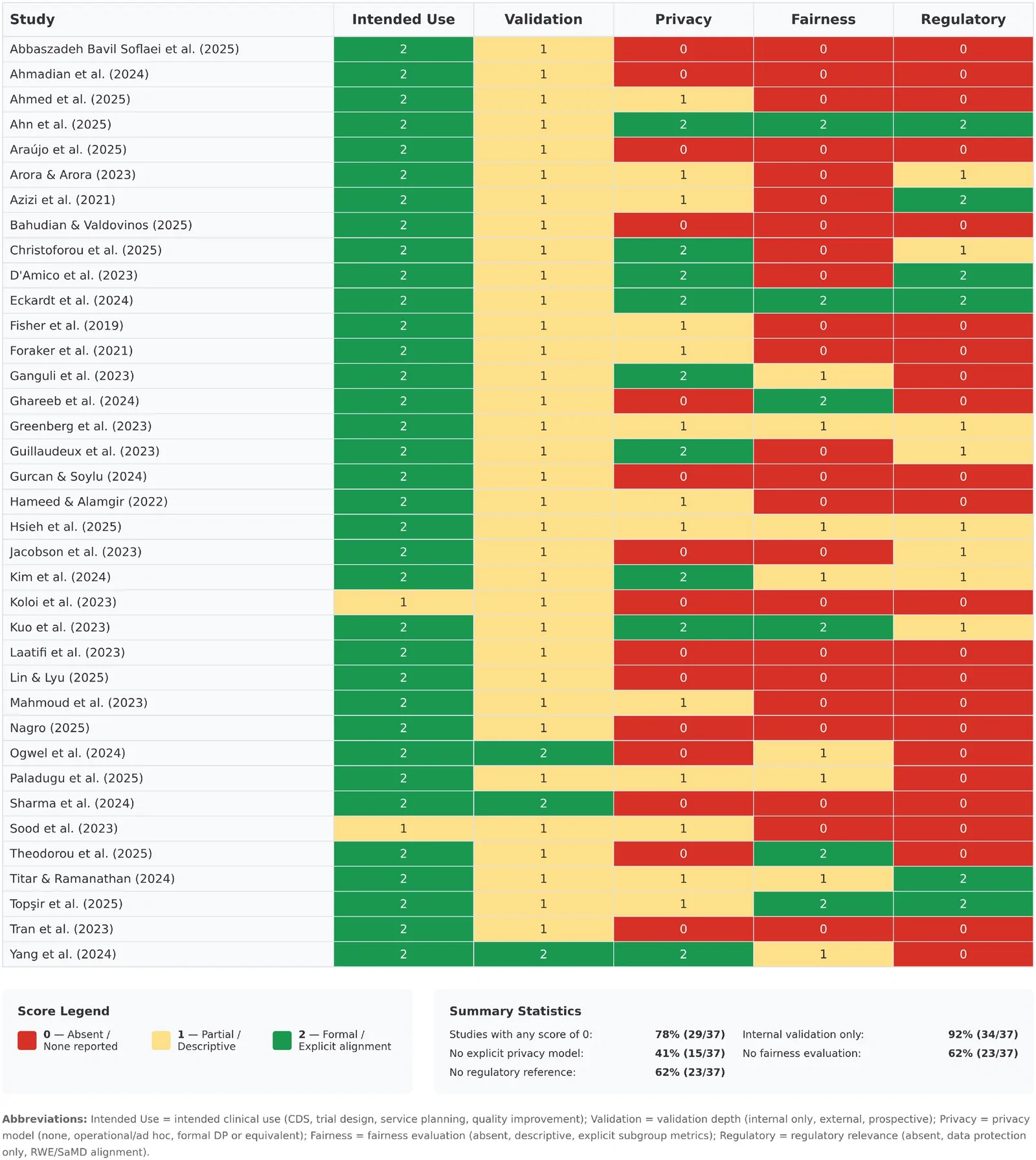
Translation readiness heatmap of included studies. Each row represents one of 37 included studies; columns represent the 5 translation-readiness domains. Cell colour indicates score: red = 0 (absent/none reported), yellow = 1 (partial/descriptive), green = 2 (formal/explicit alignment). The predominance of red and yellow across privacy, fairness, and regulatory domains illustrates persistent under-reporting of clinical deployment-readiness evidence despite generally clear clinical-use statements.

**Table 2.** Translation readiness and governance of included studies.

Article	Intended clinical use	Validation depth	Privacy model	Fairness evaluation	Regulatory relevance
**Abbaszadeh Bavil Soflaei et al^20^**	Clinical Decision Support (CDS) (2)	Internal only (1)	None stated (0)	Absent (0)	Absent (0)
**Ahmadian et al^21^**	CDS (2)	Internal only (1)	None stated (0)	Absent (0)	Absent (0)
**Ahmed et al^22^**	CDS (2)	Internal only (1)	Operational/General Privacy (1)	Absent (0)	Absent (0)
**Ahn et al^23^**	Trial design & execution/Service planning (2)	Internal only (1)	Formal DP or other formal model (2)	Explicit subgroup evaluation (2)	RWE—external control discussion/SaMD or device-regulation explicitly considered (2)
**Araújo et al^24^**	CDS (2)	Internal only (1)	None stated (0)	Absent (0)	Absent (0)
**Arora and Arora^25^**	CDS (2)	Internal only (1)	Operational/General Privacy (1)	Absent (0)	Data protection only (1)
**Azizi et al^26^**	Trial design & execution (2)	Internal only (1)	Ad hoc SDC (1)	Absent (0)	RWE—external control discussion/SaMD or device-regulation explicitly considered (2)
**Bahudian and Valdovinos^27^**	CDS (2)	Internal only (1)	None stated (0)	Absent (0)	Absent (0)
**Christoforou et al^28^**	CDS/Service planning (2)	Internal only (1)	Formal DP or other formal model (2)	Absent (0)	Data protection only (1)
**D’Amico et al^29^**	CDS/Trial design & execution (2)	Internal only (1)	Formal DP or other formal model (2)	Absent (0)	RWE—external control discussion/SaMD or device-regulation explicitly considered (2)
**Eckardt et al^30^**	Trial design & execution/CDS (2)	Internal only (1)	Formal DP or other formal model (2)	Explicit subgroup evaluation (2)	RWE—external control discussion/SaMD or device-regulation explicitly considered (2)
**Fisher et al^31^**	Trial design & execution (2)	Internal only (1)	Operational/General Privacy (1)	Absent (0)	Absent (0)
**Foraker et al^32^**	CDS/Service planning/Quality improvement (2)	Internal only (1)	Ad hoc SDC (1)	Absent (0)	Absent (0)
**Ganguli et al^33^**	CDS/Service planning (2)	Internal only (1)	Formal DP or other formal model (2)	Discussion of under-represented groups (1)	Absent (0)
**Ghareeb et al^34^**	CDS/Quality improvement (2)	Internal only (1)	None stated (0)	Explicit subgroup evaluation (2)	Absent (0)
**Greenberg et al^35^**	CDS/Quality improvement (2)	Internal only (1)	Ad hoc SDC (1)	Discussion of disparity/subgroups (1)	Data protection only (1)
**Guillaudeux et al^36^**	CDS/Trial design & execution (2)	Internal only (1)	Formal DP or other formal model (2)	Absent (0)	Data protection only (1)
**Gurcan and Soylu^37^**	CDS (2)	Internal only (1)	None stated (0)	Absent (0)	Absent (0)
**Hameed and Alamgir^38^**	CDS (2)	Internal only (1)	Operational/General Privacy (1)	Absent (0)	Absent (0)
**Hsieh et al^39^**	CDS (2)	Internal only (1)	Operational/General Privacy (1)	Ethical scrutiny/Bias mitigation needs (1)	Data protection only (1)
**Jacobson et al^40^**	CDS (2)	Internal only (1)	None stated (0)	Absent (0)	Data protection only (1)
**Kim et al^41^**	CDS/Trial design & execution (2)	Internal only (1)	Formal DP or other formal model (2)	Explicit discussion of cohorts (1)	Data protection only (1)
**Koloi et al^42^**	Service planning (1)	Internal only (1)	None stated (0)	Absent (0)	Absent (0)
**Kuo et al^43^**	CDS/Service planning (2)	Internal only (1)	Formal DP or other formal model (2)	Explicit subgroup evaluation (2)	Data protection only (1)
**Laatifi et al^44^**	CDS (2)	Internal only (1)	None stated (0)	Absent (0)	Absent (0)
**Lin and Lyu^45^**	CDS (2)	Internal only (1)	None stated (0)	Absent (0)	Absent (0)
**Mahmoud et al^46^**	CDS/Quality improvement (2)	Internal only (1)	Ad hoc SDC (1)	Absent (0)	Absent (0)
**Nagro^47^**	CDS (2)	Internal only (1)	None stated (0)	Absent (0)	Absent (0)
**Ogwel et al^48^**	CDS/Service planning (2)	Internal + external (2)	None stated (0)	Fairness via interpretability/explicit mention (1)	Absent (0)
**Paladugu et al^49^**	CDS (2)	Internal only (1)	Operational/General Privacy (1)	Discussion of disparity/subgroups (1)	Absent (0)
**Sharma et al^50^**	CDS (2)	Internal + external (2)	None stated (0)	Absent (0)	Absent (0)
**Sood et al^51^**	Trial design & execution (1)	Internal only (1)	Ad hoc SDC (1)	Absent (0)	Absent (0)
**Theodorou et al^52^**	CDS/Quality improvement (2)	Internal only (1)	None stated (0)	Explicit subgroup evaluation (2)	Absent (0)
**Titar and Ramanathan^53^**	Trial design & execution (2)	Internal only (1)	Operational/General Privacy (1)	Discussion of under-represented groups (1)	RWE—external control discussion (2)
**Topşir et al^54^**	CDS (2)	Internal only (1)	Operational/General Privacy (1)	Explicit subgroup evaluation (2)	SaMD or device-regulation explicitly considered (2)
**Tran et al^55^**	CDS (2)	Internal only (1)	None stated (0)	Absent (0)	Absent (0)
**Yang et al^56^**	CDS (2)	Internal + external (2)	Formal DP or other formal model (2)	Addresses class imbalance (1)	Absent (0)

This table summarizes, for each study, the intended clinical use, validation depth, privacy model, fairness evaluation, and regulatory relevance, using a simple rubric in which scores in parentheses indicate increasing levels of translation readiness (0 = none reported, 1 = partial or descriptive, 2 = formal or explicit alignment).

Abbreviations: CDS, clinical decision support; DP, differential privacy; SDC, statistical disclosure control; RWE, real-world evidence.

**Table 3.** Cross-tabulation of primary motivation categories and clinical application domains.

	Clinical application domain				
Primary motivation	Clinical decision support	Trial design and execution	Service planning	Quality improvement	Total studies
**Dataset augmentation**	21	2	2	1	22
**Privacy-preserving data sharing**	7	4	4	3	10
**In-silico trials**	1	3	0	0	3
**Bias mitigation**	2	0	1	1	2
**Total (non-unique)**	**31**	**9**	**7**	**5**	**37**

This table maps the 4 primary motivation categories against the 4 clinical application domains for the 37 included studies. Cell values represent study counts; studies may appear in more than one column.

Bold values indicate summary totals. Row totals represent unique study counts within each primary motivation category; individual cells and column totals are non-unique because one study may contribute to multiple clinical application domains.

Regulatory benchmarking was limited: 23 studies (62%) made no regulatory reference, 8 (22%) mentioned data-protection law or regulatory guidance only, and 6 (16%) linked their work to real-world evidence frameworks or SaMD/device-regulation concepts (Table 2). Of the 37 studies, only 4 explicitly stated whether the synthetic data were intended for a no-release or delayed-release paradigm; the remainder left the deployment context implicit. These observed gaps across validation, privacy, fairness, and regulatory reporting directly informed the 7-domain TRUST-SD reporting checklist presented in the Discussion. These proportions should be interpreted cautiously given the small corpus (*n* = 37), though they are consistent with broader observations in the synthetic data literature.^5^^,^^10^

### Opportunities and limitations

Taken together, the included studies demonstrate significant opportunities while also serving to illustrate current limitations. The following synthesis draws on the limitations explicitly reported by the original authors. A summary of these author-reported findings is provided in Table 4.

**Table 4.** Author-reported opportunities and limitations of synthetic data.

Article	Reported findings (opportunities)	Reported findings (limitations)
**Abbaszadeh Bavil Soflaei et al^20^**	CTGAN is a potent solution for generating synthetic data that faithfully replicates real-world data distribution to tackle challenges like mixed data types and highly imbalanced data.	Performance depends on feature quality; hyper-parameter tuning is time-consuming; generalisability to new datasets may be limited.
**Ahmadian et al^21^**	Synthetic data, when combined with real data, can enhance predictive model performance and help overcome data scarcity. Offers a promising solution for data/class imbalance.	Computational limitations. Current methods may not fully emulate real-world data complexity. Deep learning models are difficult to interpret. Synthetic data might carry original biases.
**Ahmed et al^22^**	Synthetic data can augment datasets, addressing data scarcity and improving model generalization. Offers a compelling privacy-utility trade-off in healthcare.	Small real-world dataset limits generalisability. Synthetic data struggled to replicate the complexity of real clinical distributions, especially edge cases.
**Ahn et al^23^**	AI-generated synthetic data can faithfully replicate complex hepatology datasets, maintain clinical signals, and ensure strong privacy. Can address data scarcity, enhance model generalisability, and accelerate research.	The study excluded patients with missing data. Acceptance in the clinical community will require transparent validation and regulatory guidelines. The risk of propagating biases from the original data remains.
**Araújo et al^24^**	The DAS method demonstrates high performance in cardiotoxicity prediction following doxorubicin treatment which exceeds other methods.	Limited dataset size from single center may affect generalisability. 1-year follow-up might miss late-onset cardiotoxicity. Class imbalance in existing dataset may affect model’s performance and generalizability. Basing synthetic data on existing cases may miss novel emerging patterns seen in real-world data. Prone to overfitting and requires external validation.
**Arora and Arora^25^**	Potential to act as a replacement for real data, increasing access and dataset size. Can replicate research results with high concordance.	Needs further research to confirm privacy protection against re-identification. Did not test generalisability to a larger population or explore re-identification risk.
**Azizi et al^26^**	Can serve as a “reasonable proxy” for real clinical trial datasets. Offers more options for privacy and can alleviate ethics reviews, accelerating research.	Will not give exact numeric results, but the same conclusions. More difficult to justify for primary analyses.
**Bahudian and Valdovinos^27^**	Synthetic datasets can be effective surrogates for real data in training predictive models. Dropping missing values before synthesis improved model utility.	The study only used a subset of the full clinical dataset, omitting important variables. Ventricular dysfunction was simplified to a binary outcome.
**Christoforou et al^28^**	The framework successfully creates and selects high-quality synthetic data. Enhances data exchange by overcoming legislative regulations with high similarity and good privacy.	Acceptable %EM levels for sharing synthetic data are not established, which is a main barrier to adoption. Requires future research and regulatory work.
**D’Amico et al^29^**	Conditional GANs can generate high-fidelity synthetic cohorts, resolve missing data, and serve as control arms in clinical trials, accelerating research and precision medicine.	Generating high-fidelity synthetic patients with comprehensive clinical and genomic information remains challenging. Synthetic data may not fully capture all nuances of rare diseases.
**Eckardt et al^30^**	Democratizes clinical research by enabling exploratory analysis, ML model training, and cohort augmentation. Offers high potential for creating synthetic control cohorts.	Tabular models may not adequately capture time-to-event data distribution shifts. Current survival metrics may not fully capture long-term nuances. Lack of regulatory guidance.
**Fisher et al^31^**	Produces synthetic datasets with lower privacy concerns. Can run simulated clinical trials, act as synthetic control arms, and forecast individual risks for treatment selection.	Progress is slowed by limited high-quality longitudinal health datasets and the ability of ML methods to extract insights from them.
**Foraker et al^32^**	Enables privacy-preserving data downloads and accelerates discovery. Useful for hypothesis generation and developing digital health tools.	Use cases may not represent all possible uses; validation should continue. Needs exploration of other commercial systems and their privacy aspects.
**Ganguli et al^33^**	Deep learning models can generate accurate and confidential synthetic copies, preserving statistical distributions. Improves research speed and access to sensitive medical data.	Traditional techniques struggle with complex, multi-categorical patient data. GANs can suffer from mode collapse, limited diversity, and inaccuracy.
**Ghareeb et al^34^**	The AI platform achieved high sensitivity and specificity. Synthetic data enabled external validation and global data sharing.	The AI model cannot distinguish complicated from uncomplicated appendicitis. Retrospective design and limited geographic diversity may reduce generalisability.
**Greenberg et al^35^**	Offers a novel approach for EHR analytics that markedly expedites EHR research by removing IRB/broker needs.	Slight but statistically significant descriptive differences for rare categorical variables. The proprietary algorithm limits independent investigation.
**Guillaudeux et al^36^**	A patient-centric approach generating synthetic data that allows the same interpretations to be made without risking patient privacy.	Applying the Avatar method to data types other than tabular data (images, high-dimensional, time series) requires specific developments. Privacy risk is rarely documented for synthetic data in general.
**Gurcan and Soylu^37^**	Combining deep generative models (GANs) with supervised ensemble learning can yield balanced results and is superior for generating realistic, diverse synthetic samples.	GANs can suffer from mode collapse, overfitting, and representational bias.
**Hameed and Alamgir^38^**	Synthetic data improves ML accuracy for clinical practice. Effectively eliminates class imbalance and data scarcity, preventing overfitting/underfitting.	Lack of proper benchmarks due to private datasets. Most studies use small, noisy, imbalanced private datasets, which can be misleading.
**Hsieh et al^39^**	DALL-M provides a unique capability to generate new context-aware features in tabular data, anchored to medical knowledge, improving model performance.	Traditional augmentation can introduce inconsistencies. Existing generative approaches for image/text data are challenging for tabular clinical data. Needs bias mitigation strategies.
**Jacobson et al^40^**	Synthetic data can augment datasets, leading to more stable and reliable predictions that are less sensitive to noise.	Modelling very small datasets is challenging: small sample size (18 patients) leads to underfitting/overfitting and limited generalisability.
**Kim et al^41^**	Viable for AI model development of high-quality oncology data, especially when used for small sample sizes. Retains statistical correlation and privacy risks are mitigated by DP.	Single institution/Asian population limits generalisability. Time-varying variables were transformed to fixed points, not reflecting real dynamics.
**Koloi et al^42^**	Virtual populations increase ML model predictive performance. Addresses the shortage of clinical data for AI/ML. Copulas can model continuously correlated variables.	Copulas had no reported application for real-patient clinical data (prior to this study).
**Kuo et al^43^**	The extended GAN setup effectively mitigates mode collapse and captures imbalanced distributions, producing highly secure synthetic datasets with high utility for downstream ML algorithms.	GANs are notorious for mode collapse, leading to low diversity and potential omission of minority patients/practices. This can indirectly harm patient care.
**Laatifi et al^44^**	Synthetic data generation is a promising approach to overcome limited data availability, opening opportunities for collaborative research, model building, and pattern identification.	Limited dataset availability due to acquisition accessibility and costs.
**Lin and Lyu^45^**	GAN-based synthetic data significantly improves predictive model performance for imbalanced datasets. It can produce high-utility samples for discriminative power.	Traditional measures of distributional fidelity may not align with ultimate utility for specific clinical applications.
**Mahmoud et al^46^**	The visual learning approach is applicable in the first stage of data analysis for early/personalised diagnosis, providing human-in-the-loop ML visualization tools.	Medical datasets are typically collected from specific areas, in varied formats, and are challenging to access. Real-world data is often not ready for ML implementation.
**Nagro^47^**	CTGAN addresses data imbalance/scarcity, improving model generalizability, accuracy, and robustness. Enables reliable predictions in diverse populations.	Synthetic data quality depends on the diversity of the original dataset. Synthetic augmentation may introduce overfitting if the original data are biased.
**Ogwel et al^48^**	Strategic use of synthetic data strengthens reliability, generalizability, and ethical integrity of predictive models. Helps overcome data scarcity and biases.	Synthetic data may advance bias propagation if biases exist in primary data. Quality is dependent on the underlying data.
**Paladugu et al^49^**	Synthetic data is useful for overcoming sample size limitations, especially for uncommon health issues. Enhances data diversity and improves predictive accuracy.	Unconditioned GANs may underrepresent complex biological dynamics which lead to fluctuations in real-world data (eg, circadian rhythm). A high synthetic-to-real ratio may introduce overfitting. Requires external validation.
**Sharma et al^50^**	Prediction performance is enhanced using C-GANs in comparison to standard GANs and in combination with autoencoders.	The analysis was limited to gut microbiome and did not include microbiomes residing in other body sites. Synthetic datasets used balanced case-control numbers, which may not reflect real-world dataset imbalances.
**Sood et al^51^**	The Bayesian network captured relationships and produced realistic synthetic subjects, facilitating data sharing and simulation studies while preserving privacy.	Models trained on synthetic data exhibited reduced predictive performance. The synthetic data may not fully capture subtle interdependencies.
**Theodorou et al^52^**	FairPlay enhances algorithmic performance and reduces bias without additional data/pipeline changes. It addresses imbalanced datasets and preserves privacy.	Other methods (SMOTE, MCRAGE, SMA) struggled with high-dimensional, skewed, binary data, showing performance decay or computational inefficiency.
**Titar and Ramanathan^53^**	VAEs can be used for generative modelling of drug dosing determinants, demonstrating potential as an AI model for generating virtual populations for clinical trial simulations.	The model’s performance was characterized as “mixed.” It performed poorly for variables with low minority class frequency.
**Topşir et al^54^**	Data augmentation (including GANs) significantly improves model performance. GANs show robust ability to generate synthetic data, enabling better generalization.	Alternative approaches (VAE, SMOTE) could be considered for comparison. Future studies could compare GAN-based data augmentation with other methods.
**Tran et al^55^**	A synthetic population allowed the development of a simpler prediction tool with 7 easily collected variables, aiding early identification of high-risk CKD patients.	The synthetic learning database included only 2000 synthetic patients with balanced classes, which may not reflect real-world distributions. The model lacks external validation.
**Yang et al^56^**	Synthetic data can improve the robust performance of classifiers when balanced. TransCGAN shows high consistency with original data and supports clinical usability.	Synthetic sample size must be tuned—performance degrades when synthetic sample ratios are too high. The study evaluates only 2 datasets, and external validation is limited.

This table presents a structured content synthesis of the key opportunities and limitations of using synthetic data as reported by the authors of the 37 included studies. It captures the main benefits, such as accelerating research and improving models, alongside significant challenges, including bias propagation, lack of standardization, and regulatory uncertainty.

- **Author-reported opportunities** 
  Key opportunities include the democratization of clinical research by bypassing data-sharing bottlenecks,^30^^,^^32^ enhancement of AI through more robust and generalizable models for data-scarce scenarios,^21^^,^^41^^,^^52^ and emerging roles in creating synthetic control cohorts to reduce the recruitment burden and cost of RCTs.^26^^,^^30^ This is particularly valuable for enabling privacy‑preserving access to otherwise restricted clinical trial and EHR datasets, supporting hypothesis generation and downstream model development.
- **Author-reported limitations** 
  Despite this potential, authors consistently reported significant limitations, including bias propagation from source datasets,^21^^,^^23^^,^^37^^,^^39^^,^^48^ limited generalizability from small single-center cohorts,^24^^,^^41^ and the computational and interpretability burden of advanced generative models (the “black box” challenge).^21^ Technical gaps were also noted in capturing time-to-event dynamics^30^ and representing rare events and outliers.^21^

The field also appears constrained by a lack of standardization: authors repeatedly noted the absence of consensus on measuring similarity between real and synthetic data^56^ and acceptable privacy risk thresholds,^28^^,^^36^ with regulatory uncertainty remaining a challenge for clinical translation.

## Discussion

This systematic review shows that model-based synthetic tabular data have matured from a privacy-enhancing theoretical concept into a practical tool applied across diverse clinical domains, from oncology to critical care and beyond. However, by analyzing applications through a translation-readiness and governance lens, we identify a critical disconnect: while methodological innovation is accelerating, the reporting and governance infrastructure required to support or refute fitness for safe, equitable, and regulatory-compliant deployment is frequently under-reported and not yet standardized. This is a reporting and standardization gap rather than a verdict on the technology itself, which has clearly demonstrated value for specific use-cases such as dataset augmentation and class balancing. Notably, many of these translational challenges (including bias propagation, limited generalizability, and regulatory uncertainty) are explicitly acknowledged by the original study authors themselves (Table 4), indicating that the field is collectively aware of the challenges it faces.

### Application-driven versus methodology-driven corpora

Dataset augmentation was the dominant use-case in our clinical-application corpus, whereas privacy-preserving data sharing was the dominant motivation in the evaluation-focused corpus analyzed by Kaabachi et al.^10^ This difference should not be interpreted as a temporal replacement of privacy by performance. Rather, the 2 reviews capture different facets of the same field: Kaabachi et al^10^ targeted studies on synthetic data *evaluation*, while our eligibility criteria required that authors *generate and apply* model-based synthetic tabular data in a clinical context, excluding studies focused solely on privacy or methods. Privacy preservation remains an important parallel use case, particularly where synthetic data are intended for release or sharing.

Our review shares some findings with Kaabachi et al,^10^ including the lack of consensus on evaluation metrics and limited use of rigorous privacy-risk evaluation. We add a complementary clinical-application perspective by mapping intended use-cases and deployment paradigms, auditing translationally relevant evidence such as validation depth, fairness, and regulatory alignment, introducing the no-release versus delayed-release distinction, and deriving TRUST-SD from these application-level reporting gaps.

The emergence of frameworks designed to mitigate bias and improve minority representation, such as those proposed by Kuo et al^43^ and Theodorou et al,^52^ marks a crucial step in the field’s maturation toward the responsible use of synthetic data methods.

### The validation and governance gap

Despite this promise, the field faces a systemic trust gap. While specific use cases have shown feasibility (such as Azizi et al^26^ directionally replicating a Phase III oncology trial outcome), our review highlights a pervasive lack of standardized reporting for quality assurance. Crucially, 92% (34/37) of studies relied solely on internal validation. This reliance is problematic because synthetic datasets that score highly on distributional fidelity metrics may still fail to capture the complex conditional relationships required for accurate clinical prediction.^5^ Without validation on independent, external real-world datasets, phenomena such as “mode collapse” (where the generator ignores minority subgroups) or “mode invention” (where it creates implausible patient profiles) may go undetected.^5^^,^^16^

This lack of rigorous validation is compounded by ambiguity regarding intended deployment. Recent work distinguishes between “no-release” paradigms (where only synthetic data are shared) and “delayed-release” paradigms (where synthetic data accelerate access pending real-data approval).^16^ Most studies we reviewed did not specify their intended deployment strategy. This distinction is important because “no-release” applications demand robust privacy guarantees, whereas “delayed-release” workflows may tolerate lower privacy assurances if final clinical validation is performed on real data. The absence of clear reporting on these constraints currently limits confidence in, and the ability to support or refute, safe translation of these models into practice.

### Regulatory implications and in-silico trials

Synthetic tabular data sit at the intersection of data protection, clinical evidence generation, and medical-device regulation, and each pillar imposes distinct reporting demands.

Under EU General Data Protection Regulation (GDPR), data that are effectively anonymized fall outside the scope of data-protection law, and the European Health Data Space is reshaping how secondary use of health data is governed.^18^ In the United States, HIPAA provides 2 de-identification pathways: Safe Harbor, requiring removal of specified identifiers and no actual knowledge that remaining information could identify someone, and Expert Determination, where qualified expert determines and documents that re-identification risk is very small.^57^ Across all of these frameworks, however, anonymization is context-dependent, and synthetic health data can still present residual re-identification risks through rare categorical combinations, quasi-identifiers, small cells, outlier records, or model memorization.^6^^,^^17^ Reliable disclosure-risk assessment is therefore central to determining whether a synthetic dataset can defensibly be treated as de-identified or anonymous.

Beyond data protection, synthetic data increasingly intersect with regulatory frameworks for clinical evidence generation. As regulators such as the FDA, EMA, and MHRA expand guidance on Real-World Evidence (RWE),^58^ synthetic or externally derived control arms^29^^,^^31^ that rely on synthetic-data or RWD evidence would need to meet the evidentiary standards applied to external controls. A more ambitious application is the in-silico trial, in which virtual patient cohorts substitute for, or augment, real participants. Regulators are increasingly engaging with these approaches, but their use as primary evidence remains emerging, context-specific, and dependent on model credibility.^59^^,^^60^ The trial-replication work of Azizi et al^26^ illustrates the directional plausibility of this paradigm while also exposing the validation burden it carries.

On the medical-device side, 2 pathways are particularly relevant. Where synthetic-data tools support device development or evaluation, they may be candidates for FDA Medical Device Development Tools qualification within a defined Context of Use, requiring evidence that the tool is scientifically substantiated for that purpose.^61^ Where synthetic data feed diagnostic or prognostic software, the resulting system may itself fall within the scope of Software as a Medical Device (SaMD) or analogous medical-device software regulation, depending on intended use and jurisdiction.^62^^,^^63^ In both cases regulators require evidence that the software yields clinically meaningful outputs associated with the target condition,^64^ a standard that may require prospective evaluation and post-market performance monitoring, neither of which featured in the studies we reviewed. Only 6/37 (16%) of included studies addressed regulatory alignment in any explicit form, underscoring the distance between current methodological practice and the deployment-readiness evidence that regulators across these 3 pillars expect.

### The TRUST-SD reporting checklist

To improve transparency and enable context-specific risk-benefit assessment, we propose the **TRUST-SD** (**T**ransparency and **R**eporting for **U**tility, **S**afety, and **T**ranslation of **S**ynthetic **D**ata) checklist (Panel 1). TRUST-SD is an author-derived, preliminary, evidence-informed checklist derived from the reporting gaps identified in this review; it has not undergone formal consensus validation and should not be interpreted as a final reporting standard. Table S4 maps each TRUST-SD item to the corresponding observed gap and supporting rationale. TRUST-SD complements, rather than replaces, broader reporting guidance for clinical prediction models and AI-enabled health research, such as TRIPOD+AI,^65^ by focusing on synthetic-data-specific governance issues.

The checklist spans 7 domains: context and intended use, source data transparency, generation method, utility evaluation, privacy evaluation, fairness evaluation, and regulatory alignment. It is designed as a context-sensitive reporting tool: not all items apply to all applications, and authors should state which items are not applicable with justification. The deployment paradigm stated in Item 1.3 determines which evaluation domains are essential. Future validation should use a modified-Delphi process involving clinicians, data scientists, regulators, and patient representatives, to refine the items and distinguish universally essential from context-specific reporting elements.

### Strengths and limitations

A key strength of this review is the integration of a “human-in-the-loop” AI workflow,^66^^,^^67^ using Elicit to support high-sensitivity screening and NotebookLM to assist structured, reference-checked data extraction. Relative to manual screening, this approach identified all included studies (37/37), illustrating how LLMs can reduce manual burden in evidence synthesis while preserving methodological rigor through human verification. This focus on high-sensitivity screening within a human-in-the-loop design is consistent with recent evaluations of LLM-assisted systematic review workflows.^68^

Several limitations should be noted. First, consistent with our registered protocol, we restricted eligibility to model-based synthetic tabular data in indexed MEDLINE/Embase literature. As a result, studies using different terminology, non-indexed computer-science proceedings, preprints, and foundation-model or LLM-based studies not indexed or described using synthetic-data or AI/ML terminology may have been missed. Second, our scope was deliberately restricted to tabular data; findings may not generalize to imaging, free-text, or waveform synthesis, where generation methods, evaluation practices, and disclosure risks differ. These modalities represent an important parallel area that would benefit from a separate dedicated review. Third, substantial heterogeneity across clinical contexts, generator types, validation strategies, and reported outcomes precluded quantitative meta-analysis. Fourth, our *post hoc* review of pre-existing synthetic-dataset records was bounded to the already-screened corpus and does not exclude the possibility that re-evaluations exist under different terminology or outside the searched databases. Fifth, the field is evolving rapidly: although our search captures a broad snapshot of current practice, new approaches (including those leveraging foundation models) are emerging and may alter best practices. Sixth, use of a hosted LLM-assisted extraction tool may limit exact reproducibility because model behavior and platform implementation can change over time; this risk was mitigated by source-anchored human verification and a full-corpus omission audit of key governance fields. Finally, publication bias is likely, with successful applications preferentially reported and negative or null findings under-represented.

## Conclusions

Model-based synthetic tabular data are already solving practical problems in healthcare, particularly for augmenting small datasets, addressing class imbalance, and supporting bias-mitigation research. However, the prevalence of internal-only validation and the scarcity of formal governance reporting mean that, for most studies, there is insufficient evidence to support or refute fitness for specific translational scenarios (particularly those involving data sharing or regulatory-relevant applications). By adopting context-sensitive reporting checklists such as TRUST-SD and aligning with emerging regulatory standards for SaMD and real-world evidence, the field can continue to move from successful proof-of-concept studies toward the deployment of safe, reliable, and equitable clinical tools.

## Supplementary Material

ocag102_Supplementary_Data

## Figures and Tables

**Table:** 

Item	Reporting requirement
**1.1**	Clinical question and target population
**1.2**	Intended application (decision support, trial design, service planning, quality improvement)
**1.3**	Deployment paradigm: **no-release** (synthetic data only) or **delayed-release** (synthetic data pending real data access)
**1.4**	Intended end-users and decision context

**Table:** 

Item	Reporting requirement
**2.1**	Real-data source, setting, and time period
**2.2**	Sample size, missingness, and preprocessing steps
**2.3**	Known biases, limitations, or underrepresented groups in source data
**2.4**	Ethical approval and data governance for source data

**Table:** 

Item	Reporting requirement
**3.1**	Generator architecture (eg, GAN variant, VAE, diffusion model, LLM)
**3.2**	Training regime, hyperparameters, and software/library used
**3.3**	Privacy mechanism: none, operational controls, or formal model (specify type and parameters, eg, differential privacy with ε value)
**3.4**	Synthetic dataset size and any post-processing applied

**Table:** 

Item	Reporting requirement
**4.1**	Distributional fidelity metrics (eg, Kolmogorov-Smirnov, correlation preservation)
**4.2**	Downstream task utility: performance of models trained on synthetic vs real data, evaluated on held-out real data
**4.3**	Validation depth: internal only, external (independent dataset), or prospective
**4.4**	Calibration assessment where applicable

**Table:** 

Item	Reporting requirement
**5.1**	Re-identification risk assessment (eg, k-anonymity, distance to nearest neighbor)
**5.2**	Membership inference attack testing (eg, DOMIAS score) where feasible
**5.3**	Statement on how the synthetic data are **treated for governance purposes** under applicable law (and the basis for this position), including any residual re-identification risk considerations

**Table:** 

Item	Reporting requirement
**6.1**	Subgroup-specific fidelity: distributional comparisons across protected characteristics and rare conditions
**6.2**	Subgroup-specific utility: downstream model performance stratified by key groups
**6.3**	Assessment for mode collapse (underrepresentation) or mode invention (implausible combinations)

**Table:** 

Item	Reporting requirement
**7.1**	Applicable data protection framework (eg, GDPR, HIPAA) and compliance statement
**7.2**	Relevance to medical device or software regulation (eg, SaMD, MDDT) where applicable
**7.3**	If used for regulatory-relevant evidence (eg, synthetic control arms), alignment with real-world evidence guidance

## Data Availability

All data generated or analyzed during this study are included in this published article and its supplementary information files.

## Panel 1. TRUST-SD: An evidence-informed reporting checklist for synthetic tabular data in healthcare

**Preamble:** TRUST-SD is an author-derived, preliminary, evidence-informed reporting checklist informed by the systematic review and governance audit reported here; it has not been validated through a formal consensus process and should not be interpreted as a final reporting standard. Its purpose is to stimulate community and stakeholder refinement. These proposed reporting items ask authors to *document* (not necessarily to *perform*) specific evaluations when generating model-based synthetic tabular data intended to inform clinical decision-making, trial design, service planning, or quality improvement. Where an evaluation was not conducted, reporting the rationale for its omission is sufficient. The items are designed to facilitate reproducibility, enable risk assessment, and support regulatory alignment. Not all items are applicable to all use-cases; authors should report the deployment paradigm (Item 1.3) and indicate which items are not applicable to their specific context, with justification. For example, studies using synthetic data solely for internal model augmentation without any data release may justifiably report that privacy evaluation (Domain 5) is not applicable.

## Explanatory notes

- **Context-sensitivity** (general): Not all items apply to all applications. The deployment paradigm (Item 1.3) determines which evaluation domains are essential. Authors should indicate which items are not applicable to their context, with brief justification.
- **Deployment paradigm** (1.3): No-release applications require stronger privacy guarantees because synthetic data are the primary releasable asset; delayed-release applications may accept lower privacy or fidelity guarantees only if downstream validation is ultimately performed on real data [16].
- **Downstream task utility** (4.2): Distributional similarity alone is insufficient; synthetic data can preserve marginal distributions while corrupting conditional relationships critical for prediction [5].
- **Mode collapse/invention** (6.3): Generators may systematically underrepresent minority subgroups or create implausible feature combinations, directly affecting fairness [5, 16].
