# Supplementary material for: Translation readiness of model-based synthetic tabular data in healthcare: a systematic review and governance audit
Source: J Am Med Inform Assoc. 2026 Jun 18;33(10):2008–28. doi: 10.1093/jamia/ocag102 (PMC13630292; doi:10.1093/jamia/ocag102)
Supplement: Supplementary file 1 [file ocag102_supplementary_data.zip › Supplementary File 1.pdf]

**Supplementary File 1. Full search strategy.** This file contains the complete search terms and logic used to conduct the literature search in the Ovid MEDLINE(R) and Embase databases.

Embase <1996 to 2025 Week 31>

Ovid MEDLINE(R) and Epub Ahead of Print, In-Process, In-Data-Review & Other Non-Indexed Citations, Daily and Versions <1946 to August 06, 2025>

1 synthetic data.ti,ab. 9339

2 (healthcare or medical or medicine or clinical or health).ti,ab. 19644174

3 exp Artificial Intelligence/ 390761

4 exp Machine Learning/ 693644

5 exp Deep Learning/ 113114

6 (artificial intelligence or AI or machine learning or deep learning or neural network$ or computational intelligence).ti,ab. 788676

7 3 or 4 or 5 or 6 1229345

8 1 and 7 3675

9 2 and 8 1391

10 limit 9 to (english language and yr="2010 -Current") 1343

11 limit 10 to humans 1003

12 11 not (review or meta-analysis or systematic review or chapter or conference abstract or conference paper or editorial or letter or note or preprint or short survey).pt. 781
