# Supplementary material for: Translation readiness of model-based synthetic tabular data in healthcare: a systematic review and governance audit
Source: J Am Med Inform Assoc. 2026 Jun 18;33(10):2008–28. doi: 10.1093/jamia/ocag102 (PMC13630292; doi:10.1093/jamia/ocag102)
Supplement: Supplementary file 1 [file ocag102_supplementary_data.zip › Supplementary Table 1.pdf]

Supplementary Table 1. Detailed results of the literature screening process. This table provides a comprehensive list of the 500 unique articles that underwent title and abstract screening. For each article, it documents the final inclusion or exclusion decision, the reason for exclusion where applicable, and the corresponding recommendation and relevance score assigned by the Elicit AI platform.

Title	Authors	Year	Application Focus	Reasoning for "Application Focus"	Data Format	Reasoning for "Data Format"	Human Clinical Data	Reasoning for "Human Clinical Data"	Is the study an original research article rather than a review, comment, conference abstract, letter or editorial?	Reasoning for "Is the study an original research article rather than a review, comment, conference abstract, letter or editorial?"	Original Data Creation	Reasoning for "Original Data Creation"	Synthetic Data Generation	Reasoning for "Synthetic Data Generation"	Clinical Application	Reasoning for "Clinical Application"	Empirical Evaluation	Reasoning for "Empirical Evaluation"	Quantifiable Outcomes	Reasoning for "Quantifiable Outcomes"	Screening judgement	Reasoning for "Screening judgement"	Screening score	Independent screening ti/ab	Final decision	Reasoning for "Final decision"
A framework to create, evaluate and select synthetic datasets for survival prediction in oncology.	Christoforou, A T, Spohn, S K B, Sprave, T, Fechter, T, Ruhle, A, Nicolay, N H, Popp, I, Grosu, A L, Peeken, J C, Thieme, A H, Stylianopoulos, T, Strouthos, I, Ferentinos, K, Roussakis, Y, Zamboglou, C	2025	yes	The study focuses on clinical applications in radiation oncology, specifically dealing with survival datasets and clinical decision-making, rather than purely technical, privacy, methodological or educational aspects.	yes	The study explicitly deals with tabular data generation, as mentioned in "high-quality tabular SD" and discusses survival datasets.	yes	The study uses real-world clinical data from human patients, including prostate cancer and head and neck cancer datasets.	yes	This appears to be an original research article with clear methodology, results, and conclusions, not a review or commentary.	yes	The study actively creates synthetic data using machine learning models, comparing four different approaches and generating multiple variants.	yes	The study explicitly uses AI methods (mentions "Tabular Variational Autoencoder" and other machine learning models) for synthetic data generation.	yes	The study demonstrates direct clinical applications, specifically in radiation oncology for survival analysis and clinical decision-making.	yes	The study includes thorough empirical evaluation with clear metrics for assessing synthetic data quality, including privacy, clinical behavior, and feature distribution.	yes	The study reports clear quantifiable outcomes, including concordance indexes (C-indexes) and hazard ratios with confidence intervals.	Include	A comprehensive study in radiation oncology demonstrating synthetic data generation using machine learning techniques across multiple human clinical datasets, with robust empirical evaluation of privacy, clinical behavior, and feature distribution. The research provides quantifiable outcomes through concordance indexes and hazard ratios, focusing on tabular data generation for clinical decision-making in cancer treatment. By actively creating synthetic data and comparing multiple machine learning models, the study addresses critical challenges in clinical data sharing and privacy preservation.	4.9	Include	Include	
A holistic framework for intradialytic hypotension prediction using generative adversarial networks-based data balancing.	Lin, Hsuan-Ming, Lyu, JrJung	2025	yes	The study focuses on clinical applications, specifically predicting intradialytic hypotension (IDH) in hemodialysis patients, rather than purely technical, privacy, methodological or educational aspects.	yes	The study uses tabular data from hemodialysis patients, including hemodynamic indicators and other clinical parameters, and does not involve imaging, AI-generated text, or other non-tabular data formats.	yes	The study uses real clinical data from 40 chronic hemodialysis patients, meeting the human clinical data criterion.	yes	The study appears to be an original research article with clear methodology, results, and conclusions, not a review or commentary.	yes	The study actively creates synthetic data using a CWGAN-GP framework, which is a type of GAN, rather than using pre-existing synthetic datasets or non-AI techniques.	yes	The study explicitly uses AI methods (CWGAN-GP) to generate synthetic tabular data for balancing the dataset.	yes	The study demonstrates direct clinical application in predicting IDH, which is a significant clinical complication in hemodialysis.	yes	The study includes thorough empirical evaluation with clear metrics, statistical comparisons, and SHAP analysis for interpretability.	yes	The study reports multiple quantifiable outcomes including PR-AUC, Accuracy, ROC-AUC, and F1 scores, with statistical comparisons.	Include	A comprehensive clinical study utilizing advanced generative AI techniques to predict intradialytic hypotension in hemodialysis patients, demonstrating original research with empirical evaluation of synthetic data generation. The research employs a novel CWGAN-GP framework to create balanced clinical datasets, reporting quantifiable outcomes across multiple performance metrics and providing direct clinical application in patient care.	4.9	Include	Include	
A machine learning framework to adjust for learning effects in medical device safety evaluation.	Koola, Jejo D, Ramesh, Karthik, Mao, Jialin, Ahn, Minyoung, Davis, Sharon E, Govindarajulu, Usha, Perkins, Amy M, Westerman, Dax, Ssemaganda, Henry, Speroff, Theodore, Ohno-Machado, Lucila, Ramsay, Craig R, Sedrakyan, Art, Resnic, Frederic S, Matheny, Michael E	2025	yes	The study focuses on clinical applications, specifically medical device post-market surveillance and safety evaluation, rather than purely technical, privacy, methodological or educational aspects.	yes	The study uses tabular data, as evidenced by the mention of "patient features" and the use of gradient-boosted decision trees, which typically operate on tabular data.	yes	The study uses data from the Department of Veterans Affairs between 2005 and 2012, which is human clinical data.	yes	This appears to be an original research article with clear methodology, results, and conclusions.	yes	The study actively creates synthetic data using machine learning methods, generating 2,494 synthetic datasets based on underlying distributions from clinical data.	yes	While the exact AI method isn't explicitly stated as GAN or VAE, the study clearly uses machine learning for synthetic data generation, creating datasets that "replicate the complexity of clinical scenarios."	yes	The study has direct clinical applications in medical device safety evaluation and post-market surveillance.	yes	The study includes original research with clear empirical evaluation, including specific metrics for assessing the framework's performance.	yes	The study reports clear quantifiable outcomes, including accuracy metrics (93.6% for learning effects, 93.4% for safety signals) and performance metrics.	Include	A comprehensive machine learning study generating synthetic clinical datasets for medical device safety evaluation, demonstrating strong empirical research with quantifiable outcomes across multiple clinical dimensions. The research effectively addresses post-market surveillance challenges by creating 2,494 synthetic datasets based on Veterans Affairs clinical data, with high-performance metrics in identifying learning effects and device safety signals. The study represents an original research approach to improving medical device safety assessment through advanced AI-driven synthetic data generation techniques.	4.9	Include	Exclude	No AI generation
AI-driven synthetic data generation for accelerating hepatology research: A study of the United Network for Organ Sharing (UNOS) database	Ahn J.C., Noh Y.-K., Hu M., Shen X., Simonetto D.A., Kamath P.S., Loomba R., Shah V.H.	2025	yes	The study focuses on clinical applications, specifically liver transplant waitlist data and clinical outcomes like survival rates and MELD score performance, rather than purely technical, privacy, methodological or educational aspects.	yes	The study deals with tabular data from the UNOS database, focusing on patient records and clinical variables, and does not include imaging or non-tabular data formats.	yes	The study uses real human clinical data from the UNOS liver transplant waitlist database, which contains patient information.	yes	Based on the structure and content, this appears to be an original research article with clear methods, results, and conclusions.	yes	The study actively creates synthetic data using diffusion models, a type of AI generative technique, as explicitly stated in the methods section.	yes	The study uses diffusion models, which are AI methods, to generate synthetic tabular data, as clearly described in the methodology.	yes	The study demonstrates direct clinical applications, including transplant-free survival analysis and MELD score performance evaluation.	yes	The study includes thorough empirical evaluation with clear metrics for assessing the synthetic data's clinical utility, including statistical comparisons and clinical performance measures.	yes	The study reports multiple quantifiable outcomes, including statistical fidelity metrics, survival rates, 5-year survival rates, and AUC values for MELD-based mortality prediction.	Include	This study comprehensively addresses synthetic data generation in clinical hepatology using AI diffusion models, demonstrating robust methodology for creating high-fidelity patient records that maintain statistical and clinical integrity. The research provides quantifiable outcomes across multiple clinical metrics, including survival rates and mortality prediction, while ensuring privacy protection and potential for multi-institutional research collaboration. By generating synthetic liver transplant waitlist data, the study offers a novel approach to addressing data scarcity and enhancing clinical research capabilities.	4.9	Include	Include	
Bayesian network modeling of risk and prodromal markers of Parkinson's disease.	Sood, Meemansa, Suenkel, Ulrike, von Thaler, Anna-Katharina, Zacharias, Helena U, Brockmann, Kathrin, Eschweiler, Gerhard W, Maetzler, Walter, Berg, Daniela, Frohlich, Holger, Heinzel, Sebastian	2023	yes	The study focuses on clinical applications, specifically Parkinson's disease prediction and risk assessment, rather than purely technical, privacy, methodological or educational aspects.	yes	The study uses tabular data (18 risk and prodromal markers) and generates synthetic tabular data, not imaging or other non-tabular formats.	yes	The study uses human clinical data from 1178 healthy individuals and 24 PD cases from the TREND study.	yes	This appears to be an original research article with novel findings and methodology.	yes	The study actively creates synthetic data using AI (Bayesian networks) rather than using pre-existing synthetic datasets.	yes	The study explicitly uses AI methods (Bayesian networks) to generate synthetic tabular data.	yes	The study demonstrates direct clinical applications in PD prediction and risk assessment.	yes	The study includes empirical evaluation with clear metrics, including bootstrapping and multiple comparison approaches to assess synthetic data realism.	yes	The study reports quantifiable outcomes through probabilistic confidence estimation and comparison metrics between real and synthetic data.	Include	This research demonstrates a comprehensive approach to synthetic data generation for Parkinson's disease prediction using Bayesian networks, with strong clinical relevance and empirical validation. The study successfully creates synthetic tabular data from human clinical markers, employs AI methods for data generation, and provides quantifiable outcomes in a direct clinical application. By utilizing original research methodology and focusing on human clinical data, the work offers a robust approach to understanding and modeling Parkinson's disease risk markers.	4.9	Include	Include	
Beyond imbalance: advancing breast cancer diagnosis with synthetic data and ML modeling	Abbaszadeh Bavil Soflaei M.R., Samadzamini K., Salehpour A.	2025	yes	The study focuses on clinical applications, specifically breast cancer detection and diagnosis, which is directly stated and is the primary focus of the research.	yes	The study uses the Wisconsin Diagnostic Breast Cancer dataset (WDBC), which is a tabular dataset, and there is no mention of imaging or other non-tabular data formats.	yes	The study uses the WDBC dataset, which is a well-known dataset containing human clinical data for breast cancer diagnosis.	yes	Based on the abstract's structure and content, this appears to be an original research article with novel contributions.	yes	The study actively creates synthetic data using CTGAN, a synthetic data generation method, rather than using pre-existing synthetic datasets.	yes	The study explicitly uses CTGAN for synthetic data generation, which is an AI-based method for generating synthetic tabular data.	yes	The study demonstrates direct clinical application in breast cancer detection and diagnosis, with clear focus on improving diagnostic accuracy.	yes	The abstract indicates clear empirical evaluation with comparative analysis of different machine learning algorithms and metrics for assessing performance.	yes	The study reports specific quantifiable outcomes, including accuracy rates of 99.3% on the balanced dataset.	Include	A comprehensive study on breast cancer detection utilizing synthetic data generation techniques demonstrates high clinical relevance through empirical evaluation of machine learning algorithms. The research employs the Wisconsin Diagnostic Breast Cancer dataset, generates synthetic data using CTGAN, and achieves unprecedented diagnostic accuracy of 99.3%, highlighting significant potential for improving clinical diagnostic approaches. By focusing on human clinical data and quantifiable outcomes, the study provides a novel approach to addressing class imbalance in machine learning-based medical diagnostics.	4.9	Include	Include	
Can synthetic data be a proxy for real clinical trial data? A validation study.	Azizi, Zahra, Zheng, Chaoyi, Mosquera, Lucy, Pilote, Louise, El Emam, Khaled	2021	yes	The study focuses on clinical applications, specifically analyzing bowel obstruction and survival outcomes in colon cancer patients, rather than purely technical, privacy, methodological or educational aspects.	yes	The study deals with tabular clinical trial data, with no mention of imaging or other non-tabular formats.	yes	The study uses data from 1543 human patients in a clinical trial (NCT00079274).	yes	This is an original research article with clear methodology and results, not a review or commentary.	yes	The abstract explicitly mentions using "synthetic data generated by a machine learning method" and compares it with real data, indicating active creation of synthetic data using AI.	yes	While the specific AI method isn't named, the abstract clearly indicates synthetic data generation using machine learning methods.	yes	The study demonstrates direct clinical applications by analyzing relationships between bowel obstruction and survival outcomes.	yes	The study includes thorough empirical evaluation, comparing synthetic and real data using multiple metrics (univariate distributions, bivariate relationships, Cox models).	yes	The study reports clear quantifiable outcomes including hazard ratios, confidence intervals, and information theoretic metrics.	Include	A comprehensive clinical study utilizing machine learning to generate synthetic data from a human colon cancer trial, demonstrating robust empirical evaluation of clinical outcomes through quantifiable metrics. The research successfully replicates real-world clinical findings using synthetic data, with high concordance in survival analysis and statistical comparisons across multiple clinical parameters. The study provides a methodologically rigorous approach to synthetic data generation in healthcare research, focusing on practical clinical applications and patient-related outcomes.	4.9	Include	Include	
Early diagnosis and personalised treatment focusing on synthetic data modelling: Novel visual learning approach in healthcare.	Mahmoud, Ahsanullah Yunas, Neagu, Daniel, Scrimieri, Daniele, Abdullatif, Amr Rashad Ahmed	2023	yes	The study focuses on clinical applications, specifically early diagnosis and personalized treatment of diseases including cancer, heart disease, diabetes, cryotherapy, and immunotherapy.	yes	The study appears to work with tabular data, as evidenced by the use of statistical characteristics, correlation matrices, and classification models that typically operate on tabular data.	yes	The study uses real medical datasets for various human clinical conditions (cancer, heart disease, diabetes, etc.).	yes	This appears to be an original research article with novel contributions, including a "novel visual learning approach" and original experiments.	yes	The study actively creates synthetic data using GANs, as explicitly stated: "Generative Adversarial Network is used to create and manipulate synthetic data."	yes	The study explicitly uses GANs for synthetic data generation, which is a valid AI method for generating synthetic tabular data.	yes	The study demonstrates direct clinical applications in diagnosis and treatment planning, specifically mentioning "early diagnosis and personalised treatment."	yes	The study includes original empirical evaluation with clear metrics and comparison between original and synthetic data performance.	yes	The study reports clear quantifiable outcomes including precision, recall, f-measure, accuracy, sensitivity, and specificity metrics.	Include	A comprehensive study utilizing Generative Adversarial Networks to generate synthetic medical data for clinical applications, demonstrating empirical evaluation across multiple disease domains with quantifiable performance metrics. The research focuses on improving diagnostic accuracy through visual learning techniques, using real-world medical datasets and reporting detailed classification performance across various clinical conditions. By actively creating synthetic data and comparing its utility against original data, the study provides a novel approach to addressing data challenges in medical machine learning.	4.9	Include	Include	

Generating synthetic clinical data that capture class imbalanced distributions with generative adversarial networks: Example using antiretroviral therapy for HIV.	Kuo, Nicholas I-Hsien, Garcia, Federico, Sonnerborg, Anders, Bohm, Michael, Kaiser, Rolf, Zazzi, Maurizio, Polizzotto, Mark, Jorm, Louisa, Barbieri, Sebastiano	2023	yes	The study focuses on clinical applications, specifically generating synthetic data for antiretroviral therapy (ART) for HIV treatment, rather than purely technical, privacy, methodological or educational aspects.	yes	The study deals with tabular clinical data, as evidenced by the discussion of clinical variables and class distributions, and does not mention imaging or other non-tabular formats.	yes	The study uses human clinical data related to HIV treatment and patient information.	yes	This appears to be an original research article with clear methodology and results sections.	yes	The study actively creates synthetic data using AI (GANs with VAE and memory mechanism), not using pre-existing synthetic datasets.	yes	The paper explicitly uses AI methods (GANs with VAE) for synthetic data generation.	yes	The study demonstrates direct clinical applications, specifically for HIV treatment data and enabling downstream machine learning models for healthcare applications.	yes	The study includes clear empirical evaluation with specific metrics and results.	yes	The study reports multiple quantifiable metrics including patient disclosure risk (0.095%), five specific evaluation metrics, and utility for downstream machine learning models.	Include	This research presents an innovative approach to generating synthetic clinical data using advanced AI techniques, specifically addressing challenges in HIV treatment data generation. The study demonstrates a comprehensive methodology for creating realistic, privacy-preserving synthetic datasets with high clinical utility, meeting multiple rigorous screening criteria for original research in healthcare data generation. By overcoming traditional limitations in synthetic data creation, the work offers significant potential for developing machine learning models in clinical applications.	4.9	Include	Include	
Generating Synthetic Data for Machine Learning Models from the Pediatric Heart Network Fontan I Dataset	Bahudian V., Valdovinos J.	2025	yes	The study focuses on clinical applications, specifically generating synthetic data for predicting health outcomes in Fontan patients, rather than purely technical, privacy, methodological or educational aspects.	yes	The study uses tabular data (echocardiographic and BNP measures) and does not involve imaging or other non-tabular formats.	yes	The study uses real clinical data from the Pediatric Heart Network Fontan I dataset, which contains human patient data.	yes	The study appears to be an original research article with clear methodology and results.	yes	The study actively creates synthetic data using GANs, not using pre-existing synthetic datasets.	yes	The study explicitly uses GANs for synthetic data generation, which is a valid AI method.	yes	The study demonstrates direct clinical applications, specifically focusing on predicting ventricular dysfunction in Fontan patients.	yes	The study includes thorough empirical evaluation with multiple metrics, including visual evaluation (histograms, PCA plots), quantitative evaluation (Kolmogorov-Smirnov test), classification performance metrics, and neural network distinguishability tests.	yes	The study reports clear quantifiable outcomes, including classification errors (10.99% and 9.44%) for ventricular dysfunction prediction.	Include	This research demonstrates a comprehensive approach to generating synthetic clinical data using GANs for Fontan patients, focusing on predicting ventricular dysfunction through empirical evaluation of tabular healthcare data. The study meets all screening criteria by actively creating synthetic data, utilizing human clinical information, and providing quantifiable outcomes with clear clinical applications. Rigorous methodological approaches, including multiple evaluation metrics and neural network testing, validate the synthetic data's potential utility in clinical decision-making.	4.9	Include	Include	
Improving medical machine learning models with generative balancing for equity and excellence	Theodorou B., Danek B., Tummala V., Kumar S.P., Malin B., Sun J.	2025	yes	The paper clearly focuses on clinical applications, specifically mortality prediction and addressing healthcare inequities, rather than purely technical, privacy, methodological or educational aspects.	yes	The abstract implies tabular data generation, mentioning "patient data" and "dataset patterns" without reference to imaging or other non-tabular formats.	yes	The study uses patient data and discusses clinical outcomes, clearly indicating human clinical data.	yes	The abstract describes original research with experiments and results, not a review or commentary.	yes	FairPlay is described as a "synthetic data generation approach" that actively creates new synthetic data using large language models.	yes	The study explicitly uses AI (large language models) for synthetic data generation.	yes	The study demonstrates direct clinical application in mortality prediction and addressing healthcare inequities.	yes	The study includes empirical evaluation with clear metrics and multiple experimental setups.	yes	The abstract reports specific quantifiable outcomes (21% improvement in F1 Score, performance metrics across subgroups).	Include	A novel synthetic data generation approach using large language models demonstrates significant improvements in clinical mortality prediction, addressing healthcare inequities through AI-driven data augmentation. The research provides empirical evidence of enhanced performance across diverse patient subgroups, with quantifiable metrics showing up to 21% improvement in predictive accuracy while preserving patient data privacy.	4.9	Include	Include	
Improving mortality prediction in Acute Pancreatitis by machine learning and data augmentation	Hameed M.A.B., Alamgir Z.	2022	yes	The study focuses on clinical applications, specifically the early detection and mortality prediction of Acute Pancreatitis, rather than purely technical, privacy, methodological or educational aspects.	yes	The study works with tabular data from MIMIC-III, MIMIC-IV, and eICU datasets, and does not include imaging, AI-generated text, or other non-tabular formats.	yes	The study uses real clinical data from MIMIC-III, MIMIC-IV, and eICU databases, which are known human clinical datasets.	yes	The study appears to be an original research article with novel experiments and results.	yes	The study actively creates synthetic data using various AI methods including CTGAN, TGAN, CopulaGAN, CTAB, and TVAE.	yes	The study explicitly uses AI methods (GANs and VAEs) for synthetic tabular data generation.	yes	The study demonstrates direct clinical application in mortality prediction for Acute Pancreatitis patients.	yes	The study includes extensive empirical evaluation with clear metrics and comparisons between different approaches.	yes	The study reports clear quantifiable outcomes including Fbeta scores and recall metrics for different classifiers.	Include	This research focuses on Acute Pancreatitis mortality prediction using synthetic data generation techniques, employing advanced machine learning methods on human clinical datasets. The study demonstrates a comprehensive approach to addressing data challenges through AI-driven synthetic data creation, with clear quantifiable outcomes and direct clinical applications. Multiple generative adversarial networks and a variational autoencoder were utilized to generate synthetic tabular data, enabling improved mortality prediction performance.	4.9	Include	Include	
Indirect reference interval estimation using a convolutional neural network with application to cancer antigen 125.	LeBien, Jack, Velev, Julian, Roche-Lima, Abiel	2024	yes	The study focuses on clinical applications, specifically reference interval estimation for clinical assessments and cancer diagnostics, rather than purely technical, privacy, methodological or educational aspects.	yes	The study deals with tabular data (clinical laboratory results), not imaging or other non-tabular formats.	yes	The study uses human clinical data, specifically pathology testing data and CA-125 biomarker data from human patients.	yes	This appears to be an original research article presenting novel methodology and results.	yes	The abstract explicitly mentions "methodology to generate synthetic data" and describes creating synthetic test datasets, indicating active creation of synthetic data using AI.	yes	The study uses AI methods (CNN) for synthetic data generation, though the exact method of synthetic data creation isn't fully detailed in the abstract.	yes	The study demonstrates direct clinical applications in reference interval estimation and cancer diagnostics (ovarian cancer).	yes	The study includes empirical evaluation using synthetic test datasets and real-world application with clear metrics for assessment.	yes	The abstract mentions "significant improvements" compared to state-of-the-art methods and discusses age-specific reference intervals, suggesting quantifiable outcomes.	Include	A novel AI-driven approach for generating synthetic clinical data demonstrates significant potential in reference interval estimation and cancer diagnostics, utilizing convolutional neural networks to identify healthy distributions within pathological datasets. The study presents an original research methodology that creates synthetic data, applies it to human clinical information, and provides quantifiable outcomes with direct clinical applications in ovarian cancer biomarker analysis.	4.9	Include	Exclude	No AI generation
Leveraging Artificial Intelligence and Synthetic Data Derivatives for Spine Surgery Research	Greenberg J.K., Landman J.M., Kelly M.P., Pennicooke B.H., Molina C.A., Foraker R.E., Ray W.Z.	2023	yes	The study focuses on clinical applications, specifically spine surgery research and predicting clinical outcomes (readmissions and complications), rather than purely technical, privacy, methodological or educational aspects.	yes	The study uses tabular EHR data, which meets the criterion for tabular data generation. There is no mention of imaging or other non-tabular formats.	yes	The study uses real EHR data from 15 hospitals, involving human patients undergoing spine surgery.	yes	This is clearly an original research article (retrospective cohort study) with empirical results, not a review or commentary.	yes	The abstract explicitly mentions generating synthetic data to simulate properties of real data, indicating active creation rather than using pre-existing synthetic datasets.	yes	While the specific AI method isn't explicitly stated, the study clearly involves generating synthetic tabular data using some form of statistical modeling or AI technique.	yes	The study demonstrates direct clinical applications by predicting 30-day readmissions and complications, which are important clinical outcomes.	yes	The study includes thorough empirical evaluation, comparing real and synthetic data performance using multiple metrics and statistical methods.	yes	The study reports clear quantifiable outcomes, including c-statistics for predictive models (.69-.71 for cervical fusion, .66-.69 for lumbar fusion).	Include	A comprehensive study on synthetic data generation in spine surgery research demonstrates robust clinical applications by predicting patient outcomes using electronic health records. The research effectively validates synthetic data's potential by generating tabular data that closely replicates real-world clinical characteristics and predictive performance across multiple patient cohorts. Quantifiable metrics and empirical evaluation provide strong evidence for the utility of synthetic data in expanding clinical research capabilities.	4.9	Include	Include	
Machine learning for comprehensive forecasting of Alzheimer's Disease progression	Fisher C.K., Smith A.M., Walsh J.R.	2019	yes	The study focuses on clinical applications, specifically personalized medicine for Alzheimer's Disease and disease progression prediction, rather than purely technical, privacy, methodological or educational aspects.	yes	The study deals with tabular data, including "44 clinical variables" and "cognitive exams, laboratory tests," and does not mention imaging or non-tabular data formats.	yes	The study uses real patient data from "1909 patients with Mild Cognitive Impairment or Alzheimer's Disease."	yes	Based on the abstract's structure and content, this appears to be an original research article with novel methodology and results.	yes	The study actively creates synthetic data using a Conditional Restricted Boltzmann Machine (CRBM), not using pre-existing synthetic datasets.	yes	The study explicitly uses AI methods (CRBM) to generate synthetic tabular data.	yes	The study demonstrates direct clinical applications in disease progression prediction and personalized medicine for Alzheimer's Disease.	yes	The study includes clear empirical evaluation, comparing synthetic data to real data using logistic regression and evaluating prediction accuracy against supervised models.	yes	The abstract mentions specific quantifiable outcomes, including accuracy in predicting ADAS-Cog scores and correlation structure analysis.	Include	A comprehensive machine learning study generating synthetic patient data for Alzheimer's Disease using a Conditional Restricted Boltzmann Machine, demonstrating clinical applications in disease progression prediction with quantifiable outcomes. The research involves original data creation from human clinical data, employing AI methods to simulate patient trajectories and predict cognitive changes with high accuracy. The study meets multiple screening criteria by focusing on clinical applications, using tabular data, and providing empirical evaluation of synthetic data generation.	4.9	Include	Include	
Mimicking clinical trials with synthetic acute myeloid leukemia patients using generative artificial intelligence	Eckardt J.-N., Hahn W., Rollig C., Stasik S., Platzbecker U., Muller-Tidow C., Serve H., Baldus C.D., Schliemann C., Schafer-Eckart K., Hanoun M., Kaufmann M., Burchert A., Thiede C., Schetelig J., Sedlmayr M., Bornhauser M., Wolfien M., Middeke J.M.	2024	yes	The study focuses on clinical applications, specifically dealing with acute myeloid leukemia patient data and clinical trials, rather than purely technical, privacy, methodological or educational aspects.	yes	The study deals with tabular data including "demographic, laboratory, molecular and cytogenetic variables," and does not mention imaging or non-tabular data formats.	yes	The study explicitly uses human clinical data from "1606 patients with acute myeloid leukemia" from multicenter clinical trials.	yes	Based on the abstract's structure and content, this appears to be an original research article with novel findings.	yes	The study actively creates synthetic data using AI methods (CTAB-GAN+ and normalizing flows), not using pre-existing synthetic datasets.	yes	The study clearly uses AI methods (CTAB-GAN+ and normalizing flows) for generating synthetic tabular data.	yes	The study demonstrates clinical applications through survival analysis and outcome analysis, showing direct relevance to clinical research.	yes	Clear empirical evaluation is present, including fidelity assessment, usability metrics, survival analysis, and inter-variable relationship analysis.	yes	The abstract mentions specific quantifiable outcomes including "performance scores," "survival curves," and "Hamming distances" for privacy assessment.	Include	This study demonstrates a comprehensive approach to synthetic data generation in clinical research, utilizing AI methods to create high-fidelity patient data for acute myeloid leukemia while addressing critical challenges in data accessibility and privacy. By employing advanced generative techniques and providing empirical validation through survival analysis and outcome metrics, the research offers a novel methodology for generating synthetic clinical datasets with potential broad applicability in healthcare research.	4.9	Include	Include	
Novel Generative Recurrent Neural Network Framework to Produce Accurate, Applicable, and Deidentified Synthetic Medical Data for Patients With Metastatic Cancer	Ganguli R., Lad R., Lin A., Yu X.	2023	yes	The study focuses on clinical applications, specifically dealing with postoperative metastatic cancer patient data and developing predictive classifiers for clinical outcomes.	yes	The study works with tabular data, analyzing 27 clinical features from patient records. There is no mention of imaging or other non-tabular data formats.	yes	The study uses real human clinical data from the National Surgical Quality Improvement Program, involving 167,474 patients.	yes	This appears to be an original research article with clear methodology, results, and conclusions.	yes	The study actively creates synthetic data using a neural network, not relying on pre-existing synthetic datasets.	yes	The study explicitly uses AI methods (neural network) to generate synthetic tabular data, creating both volume-matched and reduced-size synthetic cohorts.	yes	The study demonstrates direct clinical applications, including mortality prediction (30-day post-surgery death) and developing predictive classifiers.	yes	The study includes thorough empirical evaluation, comparing synthetic data against ground truth data using multiple metrics and statistical analyses.	yes	The study reports clear quantifiable outcomes, including accuracy metrics (93.2%), statistical comparisons (P-values), and classifier performance.	Include	A comprehensive study generating synthetic clinical data for metastatic cancer patients using neural network techniques, demonstrating robust empirical evaluation across multiple clinical and statistical dimensions. The research successfully creates volume-matched and reduced-size synthetic patient cohorts, achieving high accuracy in replicating original data characteristics and developing predictive classifiers. By addressing data sharing challenges while maintaining clinical utility, the methodology offers significant potential for advancing medical research and innovation.	4.9	Include	Include	

Overcoming data scarcity in radiomics/radiogenomics using synthetic radiomic features.	Ahmadian, Milad, Bodalal, Zuhir, van der Hulst, Hedda J, Vens, Conchita, Karssemakers, Luc H E, Bogveradze, Nino, Castagnoli, Francesca, Landolfi, Federica, Hong, Eun Kyoung, Gennaro, Nicolo, Pizzi, Andrea Delli, Beets-Tan, Regina G H, van den Brekel, Michiel W M, Castelijns, Jonas A	2024	yes	The study focuses on clinical applications in radiomics/radiogenomics for colorectal cancer, specifically for predictive modeling of TP53 mutational status.	yes	The study deals with tabular radiomic data, which is explicitly mentioned as "tabular synthetic data generation."	yes	The study uses data from 386 colorectal cancer patients with 2,570 lesions.	yes	This appears to be an original research article with clear methodology and results sections.	yes	The study actively creates synthetic data using five different models for synthetic data generation.	yes	The study clearly involves AI-based synthetic data generation, though the specific AI methods (GANs, VAEs, etc.) are not explicitly mentioned in the abstract.	yes	The study demonstrates direct clinical application in predictive modeling for cancer, specifically for TP53 mutational status prediction.	yes	The study includes thorough empirical evaluation with clear metrics, comparing performance across different sample sizes and evaluating the quality of synthetic data.	yes	The study reports clear quantifiable outcomes including AUC scores and statistical correlations (Chi-square = 0.932, correlation = 0.844).	Include	A comprehensive study on synthetic radiomic data generation for colorectal cancer demonstrates robust clinical applications using human patient data, with clear quantifiable outcomes and empirical evaluation. The research effectively addresses data scarcity challenges through AI-driven synthetic data creation, showing significant performance enhancements in predictive modeling across different sample sizes. By generating tabular synthetic data and integrating it with real-world radiomic data, the study provides a novel approach to improving medical AI predictive capabilities.	4.9	Include	Include	
Patient-centric synthetic data generation, no reason to risk re-identification in biomedical data analysis	Guillaudeux M., Rousseau O., Petot J., Bennis Z., Dein C.-A., Goronflot T., Vince N., Limou S., Karakachoff M., Wargny M., Gourraud P.-A.	2023	yes	The study focuses on clinical applications, specifically dealing with clinical trial and cancer observational study data. While privacy is a key aspect, it is in service of clinical data utility.	yes	The study appears to work with tabular data, as evidenced by the statistical analyses (hazard ratios, AUC) and comparison with other synthetic data generation methods.	yes	The study explicitly uses real health data from a clinical trial and a cancer observational study.	yes	Based on the abstract, this appears to be original research presenting a new method with empirical evaluation.	yes	The study presents a new method ("Avatar method") for actively creating synthetic data, not just using existing synthetic datasets.	yes	The study clearly involves AI-based synthetic data generation, comparing their method with CT-GAN (a type of GAN) and Synthpop.	yes	The study demonstrates clinical applications by evaluating treatment effectiveness (hazard ratios) and classification properties.	yes	The study includes empirical evaluation with clear metrics, comparing original and synthetic data performance.	yes	The study reports clear quantifiable outcomes including hazard ratios and AUC values with confidence intervals.	Include	A novel synthetic data generation method for clinical research demonstrates strong alignment with screening criteria, utilizing AI techniques to create privacy-preserving patient data while maintaining statistical integrity. The study successfully applies the method to real-world clinical trial and cancer observational data, providing quantifiable outcomes and empirical validation of synthetic data's clinical utility. Rigorous evaluation across multiple dimensions confirms the method's potential for generating high-quality, privacy-protected healthcare data.	4.9	Include	Include	
phylaGAN: data augmentation through conditional GANs and autoencoders for improving disease prediction accuracy using microbiome data	Sharma D., Lou W., Xu W.	2024	yes	The study focuses on clinical applications, specifically disease prediction and classification using microbiome data for conditions like T2D, Cirrhosis, and obesity.	yes	The study deals with tabular microbiome data, which is typically represented in tabular format.	yes	The study uses human clinical data, specifically microbiome data from human subjects with various conditions.	yes	This appears to be an original research article with novel methodology and results.	yes	The study actively creates synthetic data using phylaGAN, a combination of C-GAN and autoencoder.	yes	The study explicitly uses AI methods (C-GAN and autoencoder) to generate synthetic tabular data.	yes	The study demonstrates direct clinical applications in disease prediction and classification.	yes	The study includes extensive evaluation with clear metrics and validation across multiple datasets.	yes	The study reports quantifiable outcomes, specifically improvements in AUC (11%, 5%, and 32% in different scenarios).	Include	A novel AI-driven approach using generative adversarial networks demonstrates synthetic data generation for microbiome research, focusing on clinical disease prediction across multiple human health conditions. The study provides empirical evidence of improved diagnostic performance through synthetic data augmentation, with quantifiable outcomes showing significant improvements in predictive accuracy across different clinical datasets.	4.9	Include	Include	
Predicting doxorubicin-induced cardiotoxicity in breast cancer: leveraging machine learning with synthetic data.	Araujo, Daniella Castro, Simoes, Ricardo, Sabino, Adriano de Paula, Oliveira, Angelica Navarro de, Oliveira, Camila Maciel de, Veloso, Adriano Alonso, Gomes, Karina Braga	2025	yes	The study focuses on clinical applications, specifically predicting cardiotoxicity in breast cancer patients treated with Doxorubicin, rather than purely technical, privacy, methodological or educational aspects.	yes	The study uses tabular data (blood biomarkers and clinical measures) and generates synthetic tabular data, without including imaging, AI-generated text or other non-tabular formats.	yes	The study uses real clinical data from 78 Brazilian breast cancer patients, clearly involving human clinical data.	yes	Based on the abstract's structure and content, this appears to be an original research article with novel methodology and results.	yes	The study actively creates synthetic data using the DAS method, generating 4,892 synthetic samples from the original dataset.	yes	The study explicitly uses AI methods for synthetic data generation, comparing their DAS method with other established synthetic data generation techniques (ADASYN, SMOTE, SDV).	yes	The study demonstrates direct clinical application in predicting cardiotoxicity risk for breast cancer patients, which could inform treatment decisions.	yes	The study includes empirical evaluation with clear metrics and comparison against established methods.	yes	The study reports clear quantifiable outcomes including AUROC (0.85±0.10), sensitivity (0.89), and specificity (0.69).	Include	A comprehensive clinical study utilizing AI-driven synthetic data generation to predict cardiotoxicity in breast cancer patients, demonstrating robust methodology with quantifiable outcomes across multiple performance metrics. The research integrates human clinical data, generates synthetic tabular samples, and provides empirical evidence of predictive model performance, meeting critical screening criteria for clinical AI applications.	4.9	Include	Include	
Prediction of Cerebrospinal Fluid (CSF) Pressure with Generative Adversarial Network Synthetic Plasma-CSF Biomarker Pairing.	Paladugu, Phani, Kumar, Rahul, Yelamanchi, Jahnavi, Waisberg, Ethan, Ong, Joshua, Masalkhi, Mouayad, Gowda, Chirag, Lee, Ryung, Amiri, Dylan, Jagadeesan, Ram, Zaman, Nasif, Tavakkoli, Alireza, Lee, Andrew G	2025	yes	The study focuses on clinical applications (ICP monitoring) rather than purely technical aspects. While it includes technical elements (GANs), these are in service of a clinical goal.	yes	The study deals with tabular biomarker data (plasma-CSF biomarker pairs) rather than imaging or other non-tabular formats.	yes	The study uses human clinical data from NASA's Open Science Data Repository, focusing on human biomarkers.	yes	This appears to be an original research article with novel methodology and results.	yes	The study actively creates synthetic data using GANs, expanding the dataset from 29 to 279 samples.	yes	The study explicitly uses GANs for synthetic data generation of tabular biomarker data.	yes	The study demonstrates direct clinical applications in ICP monitoring for conditions like SANS, idiopathic intracranial hypertension, and traumatic brain injury.	yes	The study includes empirical evaluation with clear metrics and validation against real biomarker data.	yes	The study reports quantifiable outcomes including Mean Squared Error (MSE) of 0.0044 and discusses predictive accuracy.	Include	This research demonstrates a comprehensive approach to synthetic data generation using GANs for clinical applications, specifically focusing on non-invasive intracranial pressure monitoring through plasma biomarker analysis. The study meets all screening criteria by utilizing human clinical data, generating synthetic tabular data, and providing quantifiable outcomes with empirical evaluation of a novel diagnostic framework. The research shows strong potential for both spaceflight and clinical applications, with clear methodological rigor and clinical relevance.	4.9	Include	Include	
Spot the difference: Comparing results of analyses from real patient data and synthetic derivatives	Foraker R.E., Yu S.C., Gupta A., Michelson A.P., Pineda Soto J.A., Colvin R., Loh F., Kollef M.H., Maddox T., Evanoff B., Dror H., Zamstein N., Lai A.M., Payne P.R.O.	2020	yes	The study focuses on clinical applications rather than purely technical, privacy, methodological or educational aspects. It specifically addresses precision healthcare and clinical research.	yes	The study includes only tabular data generation, as evidenced by the statistical analyses and machine learning approaches mentioned. There is no mention of imaging or other non-tabular formats.	yes	The study uses real clinical data as the basis for synthesis, as evidenced by the comparison between synthetic and real data.	yes	The study appears to be an original research article with empirical evaluation, not a review or commentary.	yes	The study uses a data-synthesizing platform (MDClone) to actively create synthetic data, rather than using pre-existing synthetic datasets or non-AI techniques.	yes	The study explicitly focuses on synthetic data generation using AI methods, though the specific AI technique (GAN, VAE, etc.) is not mentioned in the abstract.	yes	The study demonstrates direct clinical applications in precision healthcare and clinical research.	yes	The study includes clear empirical evaluation across three use cases with specific metrics for assessing synthetic data's utility.	yes	The study reports quantifiable outcomes through statistical comparisons (P > 0.05) between synthetic and real data.	Include	A comprehensive study exploring synthetic data generation for precision healthcare, demonstrating empirical evaluation across multiple use cases with quantifiable outcomes. The research focuses on clinical applications using tabular data, actively creating synthetic datasets through AI methods and comparing them statistically to real clinical data. Multiple criteria are met, including original research, human clinical data usage, and direct clinical relevance with clear methodological rigor.	4.9	Include	Exclude	No direct clinical application
Synthesizing Feature-Aligned and Category-Aware Electronic Medical Records for Intracranial Aneurysm Rupture Prediction.	Yang, Qian, Li, Caizi, Ou, Chubin, Li, Kang, Liao, Xiangyun, Duan, Chuanzhi, Yu, Lequan, Si, Weixin	2024	yes	The study focuses on clinical applications, specifically rupture prediction for intracranial aneurysms, which is directly related to patient treatment and management.	yes	The study works with electronic medical records (EMRs) in tabular format, as evidenced by the discussion of feature-wise context relationships and clinical factors.	yes	The study uses real EMR data from patients with intracranial aneurysms, which is human clinical data.	yes	Based on the abstract's structure and content, this appears to be an original research article with novel methodology and experimental results.	yes	The study actively creates synthetic data using their novel TransCGAN method, which is an original AI-based approach.	yes	The study explicitly uses AI methods (specifically a Transformer-based conditional GAN) for synthetic data generation.	yes	The study demonstrates direct clinical application in rupture prediction for intracranial aneurysms, which is crucial for treatment and follow-up management.	yes	The abstract describes empirical evaluation with clear metrics and comparison against existing methods.	yes	The study reports clear quantifiable outcomes, including AUC of 0.89 and F1 score improvements of 5-33% compared to state-of-the-art methods.	Include	A novel AI-driven approach for generating synthetic electronic medical records demonstrates significant potential in clinical applications, specifically intracranial aneurysm rupture prediction. The study successfully addresses data scarcity and category imbalance through an innovative Transformer-based conditional GAN method, achieving quantifiable improvements in predictive performance with an AUC of 0.89 and substantial F1 score enhancements. By creating synthetic data that closely matches real clinical information, the research offers a promising solution for enhancing medical machine learning models.	4.9	Include	Include	
Synthetic Data Generation by Artificial Intelligence to Accelerate Research and Precision Medicine in Hematology	D'amico S., Dall'olio D., Sala C., Dall'olio L., Sauta E., Zampini M., Asti G., Lanino L., Maggioni G., Campagna A., Ubezio M., Russo A., Bicchieri M.E., Riva E., Tentori C.A., Travaglino E., Morandini P., Savevski V., Santoro A., Prada-Luengo I., Krogh A., Santini V., Kordasti S., Platzbecker U., Diez-Campelo M., Fenaux P., Haferlach T., Castellani G., Della Porta M.G.	2023	yes	The study focuses on clinical applications in hematology, specifically for myelodysplastic syndromes (MDS) and AML, rather than purely technical, privacy, methodological or educational aspects.	yes	The study deals with tabular clinical data including "clinical features, genomics, treatment, and outcomes," and does not mention imaging or non-tabular data formats.	yes	The study uses real patient data from 7,133 patients with MDS and AML.	yes	The study appears to be an original research article with clear methodology and results sections.	yes	The study actively creates synthetic data using a conditional generative adversarial network (cGAN) architecture, rather than using pre-existing synthetic datasets.	yes	The study explicitly uses AI methods (cGAN) for synthetic data generation.	yes	The study demonstrates direct clinical applications, including molecular classification, clinical trials, and precision medicine applications.	yes	The study includes original research with empirical evaluation, including a validation framework and clear metrics for assessing synthetic data's clinical utility.	yes	The study reports quantifiable outcomes, including data fidelity metrics and clinical endpoints from the synthetic cohort.	Include	This study demonstrates a comprehensive approach to synthetic data generation in hematology using AI, focusing on clinical applications with human patient data and quantifiable outcomes. The research employs a conditional generative adversarial network to create synthetic cohorts for myelodysplastic syndromes and AML, with clear empirical evaluation and direct clinical utility in precision medicine and clinical trials. By generating high-fidelity synthetic data, the study accelerates research potential while maintaining patient information privacy.	4.9	Include	Include	
Synthetic Data Improve Survival Status Prediction Models in Early-Onset Colorectal Cancer.	Kim, Hyunwook, Jang, Won Seok, Sim, Woo Seob, Kim, Han Sang, Choi, Jeong Eun, Baek, Eun Sil, Park, Yu Rang, Shin, Sang Joon	2024	yes	The study focuses on clinical applications, specifically improving prediction models for colorectal cancer patients, rather than purely technical, privacy, methodological or educational aspects.	yes	The study works with tabular clinical data (93 clinical features) and does not involve imaging or other non-tabular formats.	yes	The study uses real patient data from the Yonsei Cancer Center, specifically focusing on colorectal cancer patients under 50.	yes	This is clearly an original research article with a retrospective study design and original analysis.	yes	The study actively creates synthetic data using a Bayesian network-based synthesizing model, not just using pre-existing synthetic datasets.	yes	The study explicitly uses AI methods (Bayesian network-based synthesizing model) to generate synthetic tabular data.	yes	The study demonstrates direct clinical applications by improving prediction models for patient outcomes, specifically overall survival prediction.	yes	The study includes thorough empirical evaluation with clear metrics, comparing synthetic vs. original data performance and privacy measures.	yes	The study reports clear quantifiable outcomes including performance metrics (0.850, 0.836, 0.790 for different models) and statistical measures (Hellinger distance, correlation difference).	Include	A comprehensive study generating synthetic clinical data for colorectal cancer patients using Bayesian network methods, demonstrating clear clinical applications in predictive modeling with quantifiable performance metrics. The research successfully creates synthetic tabular data from human clinical information, empirically evaluating its utility in improving patient outcome predictions while maintaining statistical and privacy integrity.	4.9	Include	Include	
The national COVID cohort collaborative: Analyses of original and computationally derived electronic health record data	Foraker R., Guo A., Thomas J., Zamstein N., Payne P.R.O., Wilcox A.	2021	yes	The study focuses on clinical applications, specifically analyzing COVID-19 patient data for research purposes, rather than purely technical, privacy, methodological or educational aspects.	yes	The study deals with electronic health record (EHR) data, which is tabular in nature, and does not include imaging, AI-generated text, or other non-tabular formats.	yes	The study uses real EHR data from 34 institutional partners, clearly involving human clinical data.	yes	The study appears to be an original research article with clear methodology and results, not a review or commentary.	yes	The study uses MDClone's data-synthesizing capabilities to actively create synthetic data, rather than using pre-existing synthetic datasets or non-AI techniques.	yes	The study explicitly involves generating synthetic data using computational methods, though the specific AI technique (GAN, VAE, etc.) is not mentioned.	yes	The study demonstrates direct clinical applications, including predictive modeling for admission risk and analysis of COVID-19 outcomes.	yes	The study includes clear empirical evaluation, comparing synthetic data results with original data across multiple use cases.	yes	The study reports quantifiable outcomes, including odds ratios (0.97 vs 1.01) and predictive model performance comparisons.	Include	This research comprehensively addresses synthetic data generation in clinical research, utilizing electronic health records to create computationally derived data for COVID-19 analysis. The study demonstrates robust clinical applications through predictive modeling, empirical evaluation, and quantifiable outcomes across multiple use cases, while maintaining a strong focus on human clinical data and direct healthcare research applications.	4.9	Include	Include	

Variational autoencoders for generative modeling of drug dosing determinants in renal, hepatic, metabolic, and cardiac disease states	Titar R.R., Ramanathan M.	2024	yes	The study focuses on clinical applications, specifically precision dosing and disease progression analysis, rather than purely technical, privacy, methodological or educational aspects.	yes	The study explicitly deals with tabular data, including biomarkers and demographic characteristics, and does not mention any non-tabular data formats like imaging or AI-generated text.	yes	The study uses data from NHANES, which is a human clinical dataset containing patient-related healthcare information.	yes	Based on the abstract's structure and content, this appears to be an original research article with novel findings.	yes	The study uses a TVAE (Tabular VAE) generative model to actively create synthetic data, rather than using pre-existing synthetic datasets or non-AI techniques.	yes	The study clearly uses AI methods (specifically a VAE) for synthetic data generation.	yes	The study demonstrates direct clinical applications in precision dosing and disease progression analysis.	yes	The study includes empirical evaluation with clear metrics, comparing generated data distributions against test data using various statistical methods.	yes	The study reports quantifiable outcomes through distribution proximity measures and comparisons between generated and test data.	Include	A comprehensive study utilizing tabular variational autoencoder (TVAE) to generate synthetic clinical data for precision dosing, demonstrating strong alignment with clinical research criteria through empirical evaluation of human healthcare data. The research effectively employs AI methods to create synthetic patient information, focusing on disease progression and potential clinical trial simulations with quantifiable outcomes.	4.9	Include	Include	
A stacked ensemble approach for symptom-based monkeypox diagnosis	Nagro S.	2025	yes	The study focuses on clinical applications, specifically monkeypox detection and diagnosis, rather than purely technical, privacy, methodological or educational aspects.	yes	The study appears to use tabular data (patient symptoms) and mentions tabular GAN, suggesting tabular data generation.	yes	The study uses patient symptoms data, which implies human clinical data.	yes	Based on the structure and content, this appears to be an original research article with empirical results.	yes	The abstract explicitly mentions using Conditional Tabular GAN to generate synthetic data, indicating active creation of synthetic data using AI.	yes	The study uses Conditional Tabular GAN, which is an AI method for generating synthetic tabular data.	yes	The study demonstrates direct clinical application in disease detection and diagnosis.	yes	The study includes empirical evaluation with clear metrics and comparative analysis of different models.	yes	Clear quantifiable outcomes are reported (accuracy, precision, recall, F1-score).	Include	A comprehensive machine learning study on monkeypox detection demonstrates clinical application through synthetic data generation using AI techniques, with empirical evaluation of multiple models and quantifiable performance metrics. The research effectively addresses clinical data challenges by employing advanced methods like Conditional Tabular GAN to improve diagnostic accuracy and model robustness. Quantitative outcomes, including high accuracy and precision scores, validate the approach's potential for timely disease detection and management.	4.7	Include	Include	
Thyroid disease classification using generative adversarial networks and Kolmogorov-Arnold network for three-class classification.	Topsir, Aysel, Guler, Ferdi, Cetin, Ecesu, Burak, Mehmet Furkan, Agraz, Melih	2025	yes	The study focuses on clinical applications, specifically thyroid disease classification and diagnosis, as evidenced by the clinical context and outcomes.	yes	The study appears to work with tabular data, as evidenced by the use of machine learning models and features like thyroid stimulating hormone. There is no mention of imaging or other non-tabular data formats.	maybe	While the study deals with thyroid disease classification, which implies human clinical data, the abstract doesn't explicitly state whether the original data is from human patients. However, the context strongly suggests human clinical data.	yes	The abstract presents original research with methodology, results, and conclusions, indicating this is a full research article.	yes	The study explicitly uses GANs for data augmentation, indicating active creation of synthetic data using AI methods.	yes	The abstract clearly states the use of GANs for synthetic data generation, meeting this criterion directly.	yes	The study demonstrates direct clinical application in thyroid disease diagnosis and classification, with clear implications for clinical decision support systems.	yes	The abstract describes clear empirical evaluation with multiple machine learning models, comparison metrics, and explainability methods.	yes	The study reports specific quantifiable outcomes, including accuracy (98.68%) and F1-score (98.00%) for different models.	Include	A comprehensive machine learning study focusing on thyroid disease classification demonstrates advanced AI techniques for synthetic data generation, with clear clinical applications and quantifiable outcomes. The research integrates generative adversarial networks and Kolmogorov-Arnold networks to improve diagnostic accuracy, employing explainability methods and achieving high-performance metrics in a clinically relevant context. The study provides a robust framework for addressing data limitations and enhancing clinical decision support systems through sophisticated AI-driven approaches.	4.7	Include	Include	
A comparison study on creating simulated patient data for individuals suffering from chronic coronary disorders.	Koloi, Angela, Loukas, Vasileios S, Sakellarios, Antonis, Bosch, Jos A, Quax, Rick, Nowakowska, Karina, Tachos, Nikolaos, Kazmierski, Jakub, Papaloukas, Costas, Fotiadis, Dimitrios	2023	yes	The study focuses on clinical applications, specifically for CABG surgery patients and clinical research databases, rather than purely technical, privacy, methodological or educational aspects.	yes	The study explicitly deals with tabular data generation, as evidenced by the use of correlation matrices and the mention of "Tabular Preset" and other tabular data generation methods.	yes	The study uses real patient data from CABG surgery patients.	yes	Based on the abstract, this appears to be an original research article with novel comparative analysis.	yes	The study actively creates synthetic data using multiple AI methods (GANs, VAEs) and statistical approaches, not just using pre-existing synthetic datasets.	yes	The study clearly involves AI methods for synthetic data generation, including GANs (CTGAN), VAEs (TVAE), and other approaches.	maybe	While the study mentions potential clinical applications and ML model performance improvement, it doesn't directly demonstrate clinical applications like diagnosis or treatment planning.	yes	The study includes clear empirical evaluation with specific metrics for assessing synthetic data quality.	yes	The study reports quantifiable outcomes including correlation matrices, cosine similarity, density histograms, and statistical tests (KS and CS tests).	Include	A comprehensive study on synthetic data generation using multiple AI and statistical techniques, focusing on clinical data from CABG surgery patients with robust empirical evaluation and quantifiable outcomes. The research demonstrates strong potential for augmenting clinical research databases through advanced data synthesis methods, with particular emphasis on generating high quality virtual patient populations. While showing significant methodological rigor, the study partially addresses direct clinical applications, primarily highlighting potential improvements in machine learning model performance.	4.6	Include	Include	
A method for generating synthetic longitudinal health data	Mosquera L., El Emam K., Ding L., Sharma V., Zhang X.H., Kababji S.E., Carvalho C., Hamilton B., Palfrey D., Kong L., Jiang B., Eurich D.T.	2023	yes	The study focuses on generating synthetic administrative health data for clinical research purposes, rather than purely technical, privacy, methodological or educational aspects.	yes	The study deals with administrative health data in tabular format, with no mention of imaging or other non-tabular data types.	yes	The study uses real administrative health data from 120,000 individuals from Alberta Health's database.	yes	This appears to be an original research article with empirical evaluation and results.	yes	The study actively creates synthetic data using a recurrent deep learning model, not using pre-existing synthetic datasets.	yes	The study explicitly uses AI methods (recurrent deep learning model) to generate synthetic tabular data.	maybe	While the study demonstrates utility for research purposes, it doesn't directly demonstrate clinical applications like diagnosis or treatment planning. However, it does show applicability to clinical research through Cox regression analysis.	yes	The study includes thorough empirical evaluation with clear metrics for assessing synthetic data's utility and privacy risks.	yes	The study reports multiple quantifiable metrics including Hellinger distances, confidence interval overlaps, and disclosure risk thresholds.	Include	A comprehensive study generating synthetic administrative health data using recurrent deep learning, demonstrating strong alignment with most screening criteria through empirical evaluation of data generation, privacy risks, and research utility. The research effectively creates synthetic tabular data from human clinical sources, providing quantifiable outcomes and robust methodological approaches to synthetic data generation in healthcare research. While showing significant potential for clinical research applications, the study slightly falls short of direct clinical implementation.	4.6	Include	Exclude	No direct clinical application
A synthetic data generation system for myalgic encephalomyelitis/chronic fatigue syndrome questionnaires	Lacasa M., Prados F., Alegre J., Casas-Roma J.	2023	yes	The study focuses on clinical applications, specifically generating synthetic data for ME/CFS diagnosis and assessment. While it has educational applications, the primary focus is clinical.	yes	The study deals with questionnaire responses, which are tabular data. There's no mention of imaging or other non-tabular formats.	yes	The study uses real patient data from 2,522 ME/CFS patients from Vall Hebron Hospital.	yes	This appears to be an original research article presenting new synthetic data generation work.	yes	The abstract clearly indicates they created synthetic data using deep learning algorithms, not just using pre-existing synthetic datasets.	yes	The study explicitly uses deep learning algorithms to generate synthetic data, though the specific AI method (GAN, VAE, etc.) isn't specified.	yes	The study demonstrates direct clinical applications in disease diagnosis and assessment, specifically for ME/CFS.	yes	The study includes empirical evaluation with clear metrics and validation using real patient data.	yes	The abstract reports specific accuracy metrics (0.69-0.81) for their models.	Include	A comprehensive study generating synthetic clinical data for Myalgic Encephalomyelitis using deep learning algorithms, demonstrating high accuracy in predicting patient questionnaire responses. The research leverages a large patient dataset from Vall Hebron Hospital, creating a novel approach to understanding disease progression through AI-generated synthetic data. Quantifiable outcomes and direct clinical applications underscore the study's potential significance in medical research.	4.6	Include	Exclude	No direct clinical application
Advancing breast cancer prediction: Comparative analysis of ML models and deep learning-based multi-model ensembles on original and synthetic datasets	Ahmed K.A., Humaira I., Khan A.R., Hasan Md.S., Islam M., Roy A., Karim M., Uddin M., Mohammad A., Xames Md.D.	2025	yes	The study focuses on clinical applications, specifically breast cancer diagnosis and treatment, which is the primary focus of the research.	yes	The study appears to work with tabular data, as evidenced by the use of ML algorithms typically applied to tabular data (KNN, SVM, ANN, etc.). No mention of imaging or other non-tabular formats.	maybe	The study focuses on breast cancer diagnosis, which implies the use of human clinical data, though this could be more explicitly stated.	yes	The abstract describes original research with methodology and results, not a review or commentary.	yes	The abstract explicitly mentions synthetic data generation using TVAE (a type of VAE) and Gaussian Copula methods, indicating active creation of synthetic data.	yes	The study clearly uses AI methods (TVAE) for synthetic data generation.	yes	The study directly addresses clinical applications in breast cancer diagnosis and treatment planning.	yes	The study includes clear empirical evaluation with multiple ML models and comparative analysis.	yes	The abstract mentions accuracy and prediction performance as quantifiable outcomes.	Include	A comprehensive machine learning study on breast cancer diagnosis demonstrates synthetic data generation using advanced AI techniques, with a clear focus on clinical applications and empirical evaluation. The research employs multiple machine learning models to generate and analyze synthetic data, providing quantifiable outcomes in breast cancer prediction and treatment planning. The study meets key screening criteria by utilizing human clinical data, generating synthetic tabular data, and reporting performance metrics across different AI methodologies.	4.6	Include	Include	
Comparative analysis of machine learning models for malaria detection using validated synthetic data: a cost-sensitive approach with clinical domain knowledge integration.	Sekhar, Gudi V Chandra, Alemu, Chekol	2025	yes	The study focuses on clinical applications, specifically malaria detection and diagnosis, rather than purely technical, privacy, methodological or educational aspects.	yes	The study uses tabular data for machine learning model evaluation, though the exact format is not explicitly stated. Given the context of malaria detection, it is likely tabular clinical data.	yes	The study uses data representing "Sub-Saharan African epidemiological conditions" and mentions clinical validation, indicating the use of human clinical data or patient-related healthcare information as its basis.	yes	This is clearly an original research article with novel analysis and results.	yes	The study mentions a "rigorously validated synthetic dataset" that was created for this study, indicating active creation of synthetic data using AI rather than using pre-existing synthetic datasets or non-AI techniques.	maybe	While synthetic data is used, the abstract does not explicitly state the AI methods used for generation (GANs, VAEs, etc.), creating some uncertainty.	yes	The study directly addresses clinical application in malaria detection and screening.	yes	The study includes comprehensive empirical evaluation with statistical validation, bootstrap confidence intervals, and significance testing.	yes	The study reports clear quantifiable outcomes, including AUC (0.956), confidence intervals, and clinical cost metrics.	Include	A comprehensive machine learning study on malaria detection using synthetic clinical data demonstrates robust empirical evaluation with quantifiable outcomes across multiple algorithms. The research systematically compares machine learning models for diagnostic accuracy, employing a rigorously validated synthetic dataset representing Sub-Saharan African epidemiological conditions and providing clear clinical application metrics. While most screening criteria are strongly met, some uncertainty exists regarding the specific AI methods used for synthetic data generation.	4.6	Include	Exclude	No AI generation
CTGAN-driven synthetic data generation: A multidisciplinary, expert-guided approach (TIMA)	Parise O., Kronenberger R., Parise G., de Asmundis C., Gelsomino S., La Meir M.	2025	yes	The study focuses on clinical applications, specifically generating synthetic data from COVID-19 patient data. While it has technical aspects, the primary focus is clinical.	yes	The study explicitly deals with tabular data generation using CTGAN, with no mention of imaging or other non-tabular formats.	yes	The study uses real clinical data from 238 adult COVID-19 patients from a hospital.	yes	This appears to be an original research article with clear methodology and results, not a review or commentary.	yes	The study clearly involves active creation of synthetic data using AI (CTGAN), not just using pre-existing synthetic datasets.	yes	The study explicitly uses AI methods (CTGAN) for generating synthetic tabular data.	maybe	While the study uses clinical data, the abstract doesn't explicitly demonstrate direct clinical applications like diagnosis or treatment planning. It focuses more on the generation and validation of synthetic data.	yes	The study includes thorough empirical evaluation with clear metrics for assessing synthetic data quality.	yes	The study reports clear quantifiable outcomes including contingency scores (0.94), correlation similarity scores (0.97), and novelty metrics.	Include	A comprehensive study generating synthetic COVID-19 patient data using AI techniques demonstrates robust methodology with high-quality tabular data creation, utilizing real clinical patient information and advanced generative modeling. The research provides quantifiable outcomes and empirical validation of synthetic data generation, with strong potential for clinical applications despite some uncertainty around direct clinical utility.	4.6	Include	Exclude	No direct clinical application

DALL-M: Context-aware clinical data augmentation with large language models	Hsieh C., Moreira C., Nobre I.B., Sousa S.C., Ouyang C., Brereton M., Jorge J., Nascimento J.C.	2025	yes	The study focuses on clinical applications, specifically enhancing medical diagnostics by generating synthetic clinical data to improve diagnostic accuracy.	maybe	While the study involves tabular data (clinical features), it also includes radiology reports and X-ray images. The focus appears to be on augmenting tabular data, but the mixed data types create some uncertainty.	yes	The study uses the MIMIC-IV dataset, which is a well-known human clinical database containing patient data.	yes	Based on the abstract's structure and content, this appears to be an original research article with novel methodology and empirical results.	yes	The study actively creates synthetic data using LLMs (large language models) to generate new clinical features and synthetic values.	yes	The study uses AI methods (LLMs) to generate synthetic tabular data, specifically expanding from 9 to 91 clinical features.	yes	The study demonstrates direct clinical applications in medical diagnostics, specifically improving diagnostic accuracy through enhanced clinical data.	yes	The study includes original research with empirical evaluation using multiple machine learning models and clear metrics.	yes	The study reports clear quantifiable outcomes, including 16.5% improvement in F1 score and 25% increase in Precision and Recall.	Include	A novel AI-driven framework for generating synthetic clinical data demonstrates significant improvements in medical diagnostics by expanding tabular patient features using large language models. The study successfully creates original synthetic data from human clinical sources, achieving quantifiable performance gains in diagnostic accuracy through empirical validation across multiple machine learning models. While primarily focused on tabular data generation, the approach integrates radiology reports and shows potential for enhancing predictive modeling in healthcare.	4.6	Include	Include	
Optimizing clustering of electronic health tabular data: generative adversarial networks and Dirichlet process mixture models for advance healthcare analytics	Kita F.J., Rao G.S., Kirigiti P.J.	2025	yes	The study focuses on clinical applications including differential diagnosis, rare disease identification, and personalized treatment strategies, rather than purely technical aspects.	yes	The study explicitly focuses on EHR tabular data and mentions clustering of tabular data.	maybe	While not explicitly stated, EHR data typically refers to human clinical data. However, this could benefit from explicit confirmation.	yes	Based on the abstract's structure and content, this appears to be an original research article with novel methodology and results.	yes	The study actively creates synthetic data using GANs and other deep learning architectures, not just using pre-existing synthetic datasets.	yes	The study clearly uses AI methods (specifically GANs) for synthetic data generation.	yes	The study demonstrates direct clinical applications including differential diagnosis, multimorbidity pattern identification, and personalized treatment strategies.	yes	The study includes empirical evaluation with validation frameworks and clear assessment of synthetic data's utility in clustering.	maybe	While the abstract mentions validation frameworks and measuring differences between real and synthetic data distributions, specific metrics aren't clearly detailed.	Include	This study demonstrates a comprehensive approach to generating synthetic electronic health record data using advanced AI techniques, with a strong focus on clinical applications including differential diagnosis and personalized treatment strategies. The research employs generative adversarial networks to create synthetic tabular data, validates its utility through empirical evaluation, and shows potential for advancing healthcare research and delivery. While meeting most screening criteria, the study has minor uncertainties regarding specific quantifiable metrics and explicit confirmation of human clinical data use.	4.6	Include	Exclude	No direct clinical application
Pilot multi-omic analysis of human bile from benign and malignant biliary strictures: A machine-learning approach	Urman J.M., Herranz J.M., Uriarte I., Rullan M., Oyon D., Gonzalez B., Fernandez-Urien I., Carrascosa J., Bolado F., Zabalza L., Arechederra M., Alvarez-Sola G., Colyn L., Latasa M.U., Puchades-Carrasco L., Pineda-Lucena A., Iraburu M.J., Iruarrizaga-Lejarreta M., Alonso C., Sangro B., Purroy A., Gil I., Carmona L., Cubero F.J., Martinez-Chantar M.L., Banales J.M., Romero M.R., Macias R.I.R., Monte M.J., Marin J.J.G., Vila J.J., Corrales F.J., Berasain C., Fernandez-Barrena M.G., Avila M.A.	2020	yes	The study focuses on clinical applications, specifically differential diagnosis of biliary strictures between benign and malignant conditions, rather than purely technical, privacy, methodological or educational aspects.	maybe	The study appears to work with tabular data (metabolomic and proteomic analyses), though it's not explicitly stated how the data is formatted.	yes	The study uses human clinical data from patients with benign (n=36) and malignant conditions (n=36 CCA, n=57 PDAC).	yes	This appears to be an original research article with novel findings and methodology.	yes	The abstract mentions "generation of synthetic data with properties of real data" as part of their machine-learning pipeline, indicating that the study involves active creation of synthetic data using AI.	yes	The study uses AI methods (neural networks) for synthetic data generation, though the specific method (GAN, VAE, etc.) isn't specified.	yes	The study demonstrates direct clinical application in differential diagnosis of biliary strictures.	yes	The study includes empirical evaluation with clear metrics, using both synthetic and real data validation.	yes	The study reports quantifiable outcomes, specifically mentioning "unprecedented accuracy" in discriminating between patients with and without cancer.	Include	This research demonstrates a comprehensive clinical application using AI-generated synthetic data for differential diagnosis of biliary strictures, involving human patient data and machine learning techniques. The study meets key screening criteria by generating synthetic tabular data, employing neural networks for analysis, and reporting quantifiable outcomes in a clinical context. Original research with empirical validation and direct clinical relevance characterizes this investigation into metabolomic and proteomic biomarker identification.	4.6	Include	Exclude	No AI generation
Predictive modelling of linear growth faltering among pediatric patients with Diarrhea in Rural Western Kenya: an explainable machine learning approach.	Ogwel, Billy, Mzazi, Vincent H, Awuor, Alex O, Okonji, Caleb, Anyango, Raphael O, Oreso, Caren, Ochieng, John B, Munga, Stephen, Nasrin, Dilruba, Tickell, Kirkby D, Pavlinac, Patricia B, Kotloff, Karen L, Omore, Richard	2024	yes	The study focuses on clinical applications, specifically predicting linear growth faltering in children with diarrhea, which is a clear clinical application.	yes	The study uses tabular data including demographic, household-level characteristics, illness history, anthropometric and clinical data.	yes	The study uses human clinical data from children in Kenya.	yes	This appears to be an original research article with clear methodology and results.	yes	The study explicitly mentions using synthetic data (n=8,894) combined with real data (n=1,106), indicating active creation of synthetic data.	maybe	While synthetic data is used, the abstract doesn't specify the method of generation (GANs, VAEs, etc.), creating some uncertainty.	yes	The study has direct clinical applications in predicting linear growth faltering, which is relevant for diagnosis and treatment planning.	yes	The study includes empirical evaluation with clear metrics and validation using a separate dataset.	yes	The study reports clear quantifiable outcomes including AUC scores (83.5% [81.6-85.4] and 65.6% [60.8-70.4]).	Include	A machine learning study using synthetic and real clinical data to predict linear growth faltering in children demonstrates strong alignment with clinical research criteria, including human clinical data, quantifiable outcomes, and direct clinical applications. The research employs multiple machine learning algorithms to generate predictive models with high performance metrics, focusing on identifying risk factors for child growth challenges in a Kenyan population.	4.6	Include	Include	
Synthetic Boosted Resampling Using Deep Generative Adversarial Networks: A Novel Approach to Improve Cancer Prediction from Imbalanced Datasets	Gurcan F., Soylu A.	2024	yes	The study focuses on clinical applications, specifically cancer diagnosis and prognosis, as explicitly mentioned in the abstract.	yes	The study appears to work with tabular data, as it discusses classification and resampling methods typically used for tabular datasets. There is no mention of imaging or other non-tabular formats.	maybe	While the study mentions healthcare applications and cancer diagnosis, it's not explicitly clear if the original data is from human patients. However, the context strongly suggests this.	yes	The structure and content suggest this is an original research article with methods, results, and conclusions.	yes	The study actively creates synthetic data using GANs, as explicitly stated: "GANs... to generate high-quality synthetic data."	yes	The study clearly uses AI methods (specifically GANs) for synthetic data generation, which is a core focus of the research.	yes	The study demonstrates direct clinical applications in cancer diagnosis and prognosis.	yes	The study includes original research with clear empirical evaluation and metrics for assessing the synthetic data's utility.	yes	The study reports clear quantifiable outcomes including accuracy, precision, recall, F1 score, and ROC AUC metrics.	Include	A comprehensive study exploring synthetic data generation using GANs for clinical applications, specifically cancer diagnosis, with robust empirical evaluation and quantifiable outcomes across multiple classification models. The research demonstrates advanced AI techniques for addressing imbalanced datasets, focusing on improving predictive accuracy in healthcare through innovative data resampling strategies. Rigorous methodology and clear performance metrics underscore the potential of AI-generated synthetic data in enhancing clinical diagnostic capabilities.	4.6	Include	Include	
Utilization of machine learning methods for prediction of acute and late rectal toxicity due to curative prostate radiotherapy	Ozkan E.E., Serel T.A., Soyupek A.S., Kaymak Z.A.	2024	yes	The study focuses on clinical applications, specifically predicting rectal toxicity in prostate cancer radiotherapy patients, rather than purely technical, privacy, methodological or educational aspects.	yes	The study appears to use tabular data (clinical and dosimetric parameters) rather than imaging or other non-tabular formats.	yes	The study uses real patient data from 137 prostate cancer patients who received radiotherapy.	yes	This appears to be an original research article with clear methodology and results.	yes	The abstract explicitly mentions using "synthetic data generation method" to increase sample size from 137 to 274 patients, indicating active creation of synthetic data using AI.	maybe	While synthetic data is used, the abstract doesn't specify the AI method used for generation (GANs, VAEs, etc.), creating some uncertainty.	yes	The study demonstrates direct clinical application in predicting rectal toxicity for radiotherapy patients.	yes	The study includes empirical evaluation with clear metrics and performance measures.	yes	Clear quantifiable outcomes are reported (AUC 0.89-0.97, accuracy 95-99%, sensitivity 0.95-0.99).	Include	A clinical study on prostate cancer radiotherapy toxicity prediction using synthetic data generation demonstrates strong alignment with screening criteria, including human clinical data, direct clinical application, and quantifiable outcomes. The research employs machine learning algorithms to predict rectal toxicity with high accuracy, utilizing both original and synthetically generated patient data. While most criteria are clearly met, some uncertainty exists regarding the specific synthetic data generation method.	4.6	Include	Exclude	No AI generation
A Generative Adversarial Network (GAN) Technique for Internet of Medical Things Data	Vaccari I., Orani V., Paglialonga A., Cambiaso E., Mongelli M.	2021	yes	The study focuses on clinical applications, specifically COPD monitoring and clinical decision support systems, rather than purely technical, privacy, methodological or educational aspects.	yes	The study appears to work with tabular sensor data from IoMT devices, which fits the criterion for tabular data generation.	yes	The study uses patient data from COPD monitoring, indicating human clinical data usage.	yes	Based on the abstract's structure and content, this appears to be an original research article with novel implementation and evaluation.	yes	The study actively creates synthetic data using GANs, not using pre-existing synthetic datasets.	yes	The study explicitly uses GANs for synthetic data generation, which is a valid AI method.	yes	The study demonstrates direct clinical application in COPD monitoring and clinical decision support.	maybe	The abstract mentions validation "by a novel approach based on machine learning" but doesn't provide clear details about the evaluation metrics or methodology, creating some uncertainty.	maybe	While the abstract mentions "results obtained demonstrate how synthetic datasets created through a well-structured GAN are comparable with a real dataset," it doesn't explicitly detail specific quantifiable metrics, creating some uncertainty.	Include	A machine learning study generating synthetic patient data for COPD monitoring using GANs demonstrates strong clinical application and original research methodology. The work focuses on creating comparable synthetic datasets from real-world sensor data, with potential implications for clinical decision support systems. While most screening criteria are clearly met, some uncertainty exists regarding specific quantifiable outcomes and detailed empirical evaluation metrics.	4.3	Include	Exclude	No direct clinical application
Automated Identification of Stroke Thrombolysis Contraindications from Synthetic Clinical Notes: A Proof-of-Concept Study.	Chen, Bing Yu, Antaki, Fares, Gonzalez, Marco, Uchino, Ken, Albahra, Samer, Robertson, Scott, Ibrikji, Sidonie, Aube, Eric, Russman, Andrew, Hussain, Muhammad Shazam	2025	yes	The study focuses on clinical applications, specifically identifying thrombolysis contraindications for acute ischemic stroke patients, rather than purely technical, privacy, methodological or educational aspects.	maybe	The study uses tabular data in the form of clinical notes, though it's worth noting these are synthetic notes rather than actual tabular data. However, the output appears to be tabular (contraindications).	maybe	While the study is about human clinical data (stroke patients), it uses synthetic rather than real clinical data. However, the ultimate goal is to apply this to real EMR data.	yes	This appears to be an original research article with clear methodology and results.	yes	The study actively creates synthetic data using LLMs, specifically generating 150 synthetic clinical notes using five different models.	yes	The study explicitly uses AI methods (LLMs) to generate synthetic data, though it's generating text rather than purely tabular data.	yes	The study has direct clinical applications in identifying thrombolysis contraindications for stroke patients.	yes	The study includes original research with clear empirical evaluation and metrics.	yes	The study reports clear quantifiable outcomes including sensitivity (90.9%), specificity (99.2%), PPV, NPV, accuracy, and F1 score.	Include	A proof-of-concept study using large language models to generate synthetic clinical notes for identifying stroke thrombolysis contraindications, demonstrating high-performance metrics across multiple AI models. The research focuses on clinical applications with quantifiable outcomes, utilizing AI-generated synthetic data to potentially improve stroke patient care decision-making. While using synthetic rather than real clinical data, the study provides a promising approach to automating clinical screening processes.	4.3	Include	Exclude	No tabular data
Detection of abnormal behaviour for dementia sufferers using Convolutional Neural Networks.	Arifoglu, Damla, Bouchachia, Abdelhamid	2019	yes	The study focuses on clinical applications, specifically detecting early indicators of dementia and abnormal behavior in elderly people. This is clearly a clinical application rather than purely technical.	yes	The study appears to work with tabular data representing activity sequences, though this could be more explicitly stated.	yes	The study focuses on human clinical data, specifically behavioral patterns of elderly people with dementia.	yes	This appears to be an original research article with methodology and results.	yes	The abstract explicitly mentions "generating synthetic data reflecting on some behavioural difficulties of people with dementia."	maybe	While synthetic data generation is mentioned, the specific AI methods used (GANs, VAEs, etc.) are not explicitly stated. The focus seems to be on the CNN analysis rather than the synthetic data generation method.	yes	The study demonstrates direct clinical applications in detecting early indicators of dementia and abnormal behavior.	yes	The study includes empirical evaluation with clear comparison metrics.	yes	The study reports comparative performance metrics against state-of-the-art methods (NB, HMMs, HSMM, CRFs).	Include	A clinical study focusing on dementia detection through synthetic data generation and CNN-based activity recognition, demonstrating direct clinical applications with quantifiable performance metrics. The research involves human clinical data, original empirical evaluation, and comparative analysis against state-of-the-art methods, with some uncertainties around specific synthetic data generation techniques. The study addresses early dementia indicators through behavioral pattern analysis, providing a comprehensive approach to clinical data interpretation.	4.3	Include	Exclude	No AI generation

Evaluating the Utility and Privacy of Synthetic Breast Cancer Clinical Trial Data Sets	El Kababji S., Mitsakakis N., Fang X., Beltran-Bless A.-A., Pond G., Vandermeer L., Radhakrishnan D., Mosquera L., Paterson A., Shepherd L., Chen B., Barlow W.E., Gralow J., Savard M.-F., Clemons M., El Emam K.	2023	yes	The study focuses on clinical applications, specifically breast cancer clinical trials and data sharing for secondary analysis.	yes	The study deals with tabular clinical trial data, with no mention of imaging or other non-tabular formats.	yes	The study uses breast cancer clinical trial data from human patients.	yes	This appears to be an original research article with clear methodology and results.	yes	The study actively creates synthetic data using three different AI methods (sequential synthesis, CGAN, and VAE).	yes	The study explicitly uses AI methods (CGAN, VAE, and sequential synthesis) for generating synthetic tabular data.	maybe	While the study focuses on clinical trial data sharing, it's not directly demonstrating clinical applications like diagnosis or treatment planning. Instead, it focuses on data sharing and privacy preservation.	yes	The study includes clear empirical evaluation with specific metrics for assessing synthetic data utility and privacy risks.	yes	The study reports quantifiable outcomes including effect estimates, confidence intervals, and privacy risk metrics.	Include	A comprehensive study on synthetic data generation for breast cancer clinical trials, utilizing advanced AI techniques to create privacy-preserving datasets while maintaining high utility across multiple generative models. The research demonstrates strong empirical evaluation of synthetic data generation methods, with quantifiable outcomes and a focus on human clinical data, though with a slightly indirect approach to direct clinical applications.	4.3	Include	Exclude	No direct clinical application
Explanatory predictive model for COVID-19 severity risk employing machine learning, shapley addition, and LIME	Laatifi M., Douzi S., Ezzine H., Asry C.E., Naya A., Bouklouze A., Zaid Y., Naciri M.	2023	yes	The study focuses on clinical applications, specifically investigating cytokine levels and their relationship to COVID-19 severity for potential treatment and diagnostic purposes.	yes	The study appears to work with tabular data (cytokine levels in plasma), though there is some ambiguity about the exact format of the synthetic data generated.	yes	The study uses data from 87 human participants in a COVID-19 study.	yes	This appears to be an original research article with empirical analysis and results.	yes	The abstract explicitly mentions using machine learning and deep learning to generate synthetic data.	yes	The study uses AI methods (machine learning and deep learning) for synthetic data generation, though specific methods (GANs, VAEs) aren't mentioned.	yes	The study demonstrates direct clinical applications, specifically in understanding disease severity and potential treatment pathways.	maybe	While there is empirical evaluation of the relationship between cytokines and disease severity, the abstract doesn't clearly describe metrics for assessing the synthetic data's clinical utility.	maybe	While the study reports findings about specific cytokine levels, it's not entirely clear from the abstract what specific quantifiable metrics were used to evaluate the synthetic data's utility.	Include	A clinical study investigating COVID-19 severity through cytokine analysis using machine learning and synthetic data generation, demonstrating direct clinical applications with human patient data. The research employs AI techniques to explore potential treatment pathways and diagnostic insights, with quantifiable outcomes related to cytokine levels and disease progression. While meeting most screening criteria, the study exhibits some ambiguity regarding specific synthetic data generation metrics and evaluation approaches.	4.3	Include	Include	
Improving the performance of machine learning penicillin adverse drug reaction classification with synthetic data and transfer learning	Stanekova V., Inglis J.M., Lam L., Lam A., Smith W., Shakib S., Bacchi S.	2024	yes	The study focuses on clinical applications, specifically penicillin allergy classification and risk stratification using EHR data, rather than purely technical, privacy, methodological or educational aspects.	yes	The study uses tabular data from EHRs, though it mentions "free-text" data which is then processed. The focus appears to be on structured classification rather than text generation.	yes	The study uses real EHR data from a public health system, which is human clinical data.	yes	This is clearly an original research article with methodology, results, and conclusions.	maybe	The abstract explicitly mentions using synthetic data as part of their methodology, though it's not entirely clear if they created it themselves or used existing synthetic data.	maybe	While synthetic data is mentioned, the abstract doesn't specify the AI methods used for generation (GANs, VAEs, etc.), creating some uncertainty.	yes	The study has direct clinical applications in penicillin allergy classification and risk stratification, with practical applications for delabelling programs.	yes	The study includes empirical evaluation with clear metrics and comparison of different approaches.	yes	The study reports clear quantifiable outcomes (AUC-ROC scores of 0.984 and 0.995).	Include	A machine learning study focusing on penicillin allergy classification using electronic health record data demonstrates strong clinical application with quantifiable outcomes and empirical evaluation. The research involves human clinical data, original research methodology, and potential synthetic data generation, though some uncertainty exists regarding the specific synthetic data creation techniques. The study provides clear clinical utility in allergy risk stratification and potential delabelling programs.	4.3	Include	Exclude	No AI generation
Machine learning models trained on synthetic datasets of multiple sample sizes for the use of predicting blood pressure from clinical data in a national dataset.	Arora, Anmol, Arora, Ananya	2023	yes	The study focuses on clinical applications, specifically blood pressure prediction using machine learning models.	yes	The study uses tabular data from the National Diet and Nutrition Survey (NDNS), focusing on variables like age, sex, weight, and height.	yes	The study uses human clinical data from the NDNS dataset.	yes	This appears to be an original research article with clear methodology and results.	yes	The study actively creates synthetic data using AI methods, though the specific AI technique isn't explicitly mentioned.	yes	The study generates synthetic tabular data, though the exact AI method (GAN, VAE, etc.) isn't specified in the abstract.	maybe	While the study demonstrates blood pressure prediction, which has clinical relevance, it's more focused on comparing synthetic vs real data performance rather than direct clinical applications.	yes	The study includes original empirical evaluation with clear metrics comparing synthetic and real data performance.	yes	The study reports clear quantifiable outcomes, including mean average error metrics (8.12-8.33).	Include	A comprehensive study exploring synthetic data generation for clinical applications, utilizing machine learning models to predict blood pressure from human clinical data. The research demonstrates the potential of synthetic data to match real data performance, with clear empirical evaluation and quantifiable outcomes across multiple machine learning techniques. While methodologically robust, the study's primary focus is on validating synthetic data methodology rather than direct clinical implementation.	4.3	Include	Include	
Multicenter validation of an artificial intelligence (AI)-based platform for the diagnosis of acute appendicitis	Ghareeb W.M., Draz E., Chen X., Zhang J., Tu P., Madbouly K., Moratal M., Ghanem A., Amer M., Hassan A., Hussein A.H., Gabr H., Faisal M., Khaled I., El Zaher H.A., Emile M.H., Espin-Basany E., Pellino G., Emile S.H.	2024	yes	The study focuses on clinical applications, specifically the diagnosis of acute appendicitis, rather than purely technical, privacy, methodological or educational aspects.	yes	The study uses tabular data (patient diagnostic data) and does not mention imaging or other non-tabular formats.	yes	The study uses real human clinical data (n=2,579) and synthetic data based on clinical cases.	yes	This is clearly an original research article, specifically described as an "international, multicenter, retrospective cohort study."	maybe	While the study mentions using synthetic data, it is not entirely clear if they actively created it using AI or used pre-existing synthetic datasets. The abstract mentions a "well-qualified synthetic data set" but does not specify how it was created.	maybe	Similar to criterion 3, while synthetic data is used, the abstract does not specify the generation method (whether it was AI-based or not).	yes	The study has direct clinical applications in diagnosis and decision-making for acute appendicitis.	yes	The study includes clear empirical evaluation with multiple metrics and comparisons to CT scanning.	yes	The study reports multiple quantifiable metrics including sensitivity, specificity, negative predictive value, AUC, precision, F1 score, and Matthews correlation coefficient.	Include	A clinical study on acute appendicitis diagnosis using artificial intelligence demonstrates strong empirical evaluation with quantifiable outcomes across multiple metrics. The research employs both real and synthetic patient data, focusing on clinical decision-making with high diagnostic accuracy and potential utility in limited-resource settings. While the study meets most screening criteria, some uncertainty exists regarding the specific method of synthetic data generation.	4.3	Include	Include	
Prediction of all-cause mortality for chronic kidney disease patients using four models of machine learning.	Tran, Nu Thuy Dung, Balezeaux, Margaux, Granal, Maelys, Fouque, Denis, Ducher, Michel, Fauvel, Jean-Pierre	2023	yes	The study focuses on clinical applications, specifically developing a prediction tool for mortality in CKD patients, rather than purely technical, privacy, methodological or educational aspects.	yes	The study appears to work with tabular data, using variables like age, erythropoietin-stimulating agent, cardiovascular history, etc., and does not mention imaging or non-tabular data.	yes	The study uses data from CKD patients (Photo-Graphe 3 cohort), which is human clinical data.	yes	This is clearly an original research article with methodology, results, and conclusions.	yes	The abstract mentions using synthetic data for optimization, indicating active creation of synthetic data rather than using pre-existing datasets.	maybe	While synthetic data is mentioned, the specific AI methods used for generation (GANs, VAEs, etc.) are not explicitly stated, creating some uncertainty.	yes	The study has direct clinical application in predicting 2-year all-cause mortality for CKD patients.	yes	The study includes empirical evaluation using 10-fold cross-validation and clear performance assessment.	yes	The study reports predictive performance metrics, though specific numbers aren't provided in the abstract.	Include	A clinical study developing a mortality prediction tool for chronic kidney disease patients using synthetic data and multiple machine learning models, demonstrating direct clinical application with quantifiable outcomes. The research involves human clinical data, empirical evaluation, and original research methodology, with the primary uncertainty being the specific synthetic data generation techniques employed.	4.3	Include	Include	
Privacy-preserving generative deep neural networks support clinical data sharing	Beaulieu-Jones B.K., Wu Z.S., Williams C., Lee R., Bhavnani S.P., Byrd J.B., Greene C.S.	2019	yes	The study focuses on clinical applications, specifically using synthetic data for secondary analyses of clinical trial data (SPRINT trial).	yes	The study appears to work with tabular data from a clinical trial, with no mention of imaging or other non-tabular formats.	yes	The study uses data from the SPRINT trial, which is a human clinical trial.	yes	Based on the structure and content, this appears to be an original research article with methods and results sections.	yes	The study actively creates synthetic data using deep neural networks, not using pre-existing synthetic datasets.	yes	The study explicitly uses deep neural networks to generate synthetic tabular data.	maybe	While the study enables secondary analyses of clinical data, it's not entirely clear from the abstract if it demonstrates direct clinical applications like diagnosis or treatment planning.	yes	The study includes empirical evaluation, showing that the synthetic data can be used for hypothesis-generating analyses and that machine learning predictors generalize to the original dataset.	maybe	The abstract mentions that "Machine learning predictors built on the synthetic population generalize to the original data set," but doesn't provide specific metrics or performance measures.	Include	A novel approach to generating synthetic clinical data using deep neural networks demonstrates potential for privacy-preserving data sharing in healthcare research. The study successfully creates synthetic participants from a clinical trial dataset, enabling machine learning predictors to generalize across original and synthetic populations while maintaining participant privacy. While showing strong methodological innovation, the research exhibits partial clarity regarding direct clinical applications and specific performance metrics.	4.3	Include	Exclude	No direct clinical application
Unleashing the Power of Very Small Data to Predict Acute Exacerbations of Chronic Obstructive Pulmonary Disease.	Jacobson, Petra Kristina, Lind, Leili, Persson, Hans Lennart	2023	yes	The study focuses on clinical applications, specifically predicting acute exacerbations of COPD using machine learning models, rather than purely technical, privacy, methodological or educational aspects.	yes	The study appears to use tabular data from telemonitoring, though the exact format isn't explicitly stated. There's no mention of imaging or other non-tabular data formats.	yes	The study uses real clinical data from 18 COPD patients in a telemonitoring study.	yes	This is clearly an original research article with methods, results, and conclusions.	yes	The abstract mentions using "different proportions of synthetic data," suggesting active creation of synthetic data rather than using pre-existing datasets.	maybe	While synthetic data is used, the specific AI methods (GANs, VAEs, etc.) aren't mentioned. The abstract only refers to "different proportions of synthetic data."	yes	The study has direct clinical applications, specifically predicting acute COPD exacerbations and identifying risk factors.	yes	The study includes empirical evaluation, comparing different approaches and evaluating against authentic data.	yes	The study compares different ML approaches and evaluates their performance against authentic data.	Include	A machine learning study exploring synthetic data generation for predicting chronic obstructive pulmonary disease exacerbations, utilizing real patient data from a telemonitoring study. The research demonstrates clinical application by identifying medication changes as a key risk factor for future exacerbations, with empirical evaluation comparing different machine learning approaches. While meeting most screening criteria, some uncertainty exists regarding the specific synthetic data generation methods.	4.3	Include	Include	
Generation of a virtual cohort of TAVI patients for in silico trials: a statistical shape and machine learning analysis	Scuoppo R., Castelbuono S., Cannata S., Gentile G., Agnese V., Bellavia D., Gandolfo C., Pasta S.	2025	yes	The study focuses on clinical applications for TAVI (transcatheter aortic valve implantation) device assessment, which is clearly clinical rather than purely technical.	maybe	While the study involves patient data, it appears to focus on shape features and morphological measurements rather than purely tabular data. The data format is not explicitly clear from the abstract.	yes	The study uses real TAVI patient data as its basis for generating synthetic data.	yes	This appears to be an original research article with clear methodology and results.	yes	The study actively creates synthetic data using statistical shape modeling and machine learning techniques to generate a synthetic cohort.	yes	The study uses AI methods (machine learning techniques) to generate synthetic data, specifically creating 100 virtual patients through statistical shape modeling.	yes	The study demonstrates direct clinical applications in TAVI device assessment, including predicting pressure gradients and optimal device sizing.	yes	The study includes empirical evaluation with clear metrics and validation against the original patient population.	yes	The study reports clear quantifiable outcomes including R-squared values, RMSE, and AUC metrics.	Exclude	A comprehensive study on synthetic data generation for TAVI device assessment, utilizing machine learning techniques to create virtual patient cohorts with quantifiable clinical outcomes. The research demonstrates strong potential in integrating computational modeling, advanced machine learning, and synthetic data generation to improve predictive accuracy in cardiovascular device evaluation. While meeting most screening criteria, the study's data format presents some uncertainty regarding pure tabular data generation.	4	Exclude	Exclude	No tabular data
Generative models for synthetic data generation: application to pharmacokinetic/pharmacodynamic data.	Jiang, Yulun, Garcia-Duran, Alberto, Losada, Idris Bachali, Girard, Pascal, Terranova, Nadia	2024	yes	The study focuses on clinical applications in pharmacology, specifically for data augmentation and sharing in clinical pharmacology.	maybe	While the study includes tabular data (patient covariates), it also mentions pharmacokinetic/pharmacodynamic endpoints which could be time-series data, creating some uncertainty about the data format.	yes	The study uses patient data and clinical datasets as its basis.	yes	This appears to be an original research article with empirical evaluation and results.	yes	The study actively creates synthetic data using various AI methods including MLP cGAN, TimeGAN, and PAR.	yes	The study clearly uses AI methods (GANs and other deep learning approaches) for synthetic data generation.	maybe	While the study has clinical relevance in pharmacology, it focuses more on data generation and sharing rather than direct clinical applications like diagnosis or treatment planning.	yes	The study includes clear empirical evaluation with multiple metrics and comparisons between real and synthetic data.	yes	The study reports quantifiable outcomes including discriminative and predictive scores, statistical tests, and pharmacometrics-related metrics.	Exclude	A comprehensive study exploring synthetic data generation in clinical pharmacology using advanced AI techniques, demonstrating empirical evaluation of multiple generative methods across patient datasets. The research focuses on creating synthetic patient data with potential applications in data augmentation and sharing, utilizing deep learning approaches to generate and validate synthetic clinical information. While meeting most screening criteria, the study exhibits some uncertainty regarding data format and direct clinical application specificity.	4	Exclude	Exclude	No tabular data

Real-time automated billing for tobacco treatment: Developing and validating a scalable machine learning approach	Baughman D.J., Qassem L., Sulieman L., Matheny M.E., Fabbri D., Tindle H.A., Goodman A.C., Nelson S.D., Wright A.	2025	yes	The study focuses on clinical applications (medical billing for tobacco cessation care) rather than purely technical aspects, though it is more focused on administrative tasks than direct clinical care.	yes	The study uses tabular data (clinical notes and billing codes), though it's not entirely clear if the synthetic data generation is purely tabular.	yes	The study uses human clinical data (physician notes) as its basis.	yes	This appears to be an original research article with clear methodology and results.	yes	The study uses ChatGPT for active creation of synthetic clinical notes, meeting this criterion.	yes	The study generates synthetic data using AI (ChatGPT), though it's not using traditional methods like GANs or VAEs.	maybe	While the study has clinical relevance, it's focused on administrative tasks (billing) rather than direct clinical applications like diagnosis or treatment.	yes	The study includes empirical evaluation with clear metrics and testing on deidentified notes.	yes	The study reports clear quantifiable outcomes (PRC AUC = 0.857, F1 score = 0.835).	Exclude	A medical billing prototype using synthetic clinical notes generated by ChatGPT demonstrates promising machine learning approaches for administrative healthcare tasks. The study meets most screening criteria through its original research design, empirical evaluation, and use of human clinical data, though its focus on billing rather than direct patient care introduces some nuanced considerations. Quantifiable outcomes and clear methodological approaches support the study's potential clinical utility.	4	Exclude	Exclude	No direct clinical application
Synthetic Patient Data Generation and Evaluation in Disease Prediction Using Small and Imbalanced Datasets	Rodriguez-Almeida A.J., Fabelo H., Ortega S., Deniz A., Balea-Fernandez F.J., Quevedo E., Soguero-Ruiz C., Wagner A.M., Callico G.M.	2023	maybe	The paper focuses on clinical applications, specifically mentioning "early diagnosis, prediction and analysis in the medical field." However, it appears to be more methodological in nature, focusing on the framework and evaluation of synthetic data generation.	yes	The abstract explicitly mentions "tabular data" and uses eight medical datasets containing tabular data.	maybe	While the study uses medical datasets, it's not explicitly stated whether these are human clinical data. However, the context suggests they are likely human clinical datasets.	yes	This appears to be an original research article with empirical evaluation, not a review or commentary.	yes	The study involves active creation of synthetic data using AI methods, as evidenced by "synthetic data generation algorithms" and testing different generation sizes.	yes	The study clearly involves AI methods for synthetic data generation, though specific techniques (GANs, VAEs) aren't mentioned.	maybe	The study focuses on classification performance and data integrity rather than direct clinical applications like diagnosis or treatment planning.	yes	The study includes clear empirical evaluation with specific metrics and testing of different generation sizes.	yes	The study reports quantifiable outcomes including F1-score for classification performance and three statistical metrics for data integrity.	Exclude	A methodological study exploring synthetic data generation in medical contexts, utilizing tabular datasets and AI techniques to assess data integrity and classification performance. The research demonstrates technical capabilities in generating synthetic medical data while maintaining classification accuracy, though with some uncertainty regarding direct clinical applications. Quantifiable outcomes and empirical evaluation are present, focusing on the technical feasibility of synthetic data generation in healthcare.	4	Exclude	Exclude	No direct clinical application
Introducing the Monitoring Equipment Mask Environment.	Pazienza, Andrea, Monte, Daniele	2022	yes	The study focuses on clinical applications through a mask-based monitoring system for health conditions, including COVID-19 detection.	maybe	While the study mentions generating synthetic data, it's unclear if this is tabular data. The system collects vital signs (temperature, heart rate, oxygen saturation) which could be tabular, but this isn't explicitly stated.	yes	The system is designed for human use and the validation involves human clinical conditions (COVID-19 vs. seasonal influenza).	yes	This appears to be an original research article presenting a novel system and its validation.	yes	The abstract explicitly mentions "generating new synthetic data" for model validation.	maybe	While synthetic data is generated, the abstract doesn't specify the AI methods used (GANs, VAEs, etc.).	yes	The study demonstrates direct clinical applications through disease detection and monitoring of vital signs.	yes	The study includes empirical evaluation with clear metrics for assessing the model's performance.	yes	The study reports a specific accuracy metric (94.80%) for the machine learning model.	Exclude	A novel mask-based monitoring system demonstrates clinical applications in disease detection, utilizing synthetic data generation and machine learning to differentiate COVID-19 from seasonal influenza with high accuracy. The study presents an original research approach with empirical validation, focusing on human clinical data and quantifiable outcomes in healthcare technology. While meeting most screening criteria, some uncertainties remain regarding the precise synthetic data generation methods and data format.	3.9	Exclude	Exclude	No tabular data
Prediction of all-cause mortality in haemodialysis patients using a Bayesian network	Siga M.M., Ducher M., Florens N., Roth H., Mahloul N., Fouque D., Fauvel J.-P.	2020	yes	The study focuses on clinical applications, specifically predicting all-cause mortality in hemodialysis patients, rather than purely technical, privacy, methodological or educational aspects.	yes	The study uses tabular data with 35 variables related to patient characteristics, laboratory values, and treatments, and does not include imaging, AI-generated text, or other non-tabular formats.	yes	The study uses real clinical data from 9,010 hemodialysis patients.	yes	This is clearly an original research article with a defined methodology and results.	maybe	The abstract mentions using synthetic data to improve model performance, but it is unclear whether the synthetic data was actively created using AI methods or if pre-existing synthetic datasets were used.	maybe	While synthetic data is mentioned, the abstract does not specify the AI methods used for its generation. It only mentions using AI methods for prediction.	yes	The study has a direct clinical application in predicting all-cause mortality, which is relevant for patient care and management.	yes	The study includes empirical evaluation with clear metrics and validation of the prediction model.	yes	The study reports clear quantifiable outcomes including ROC-AUC (0.78), sensitivity (72%), specificity (69%), and predictive values.	Exclude	A clinical study on hemodialysis mortality prediction using AI methods demonstrates strong alignment with most screening criteria, including human clinical data, quantifiable outcomes, and direct clinical application. Uncertainty exists around the specific synthetic data generation process, creating a nuanced assessment of the study's methodological approach. The research provides a comprehensive predictive model with clear performance metrics for patient mortality risk.	3.9	Exclude	Exclude	No AI generation
Clinical decision support for chemotherapy-induced neutropenia using a hybrid pharmacodynamic/machine learning model	Hughes J.H., Tong D.M.H., Burns V., Daly B., Razavi P., Boelens J.J., Goswami S., Keizer R.J.	2023	yes	The study focuses on clinical applications, specifically predicting chemotherapy-induced neutropenia and supporting clinical decision-making, rather than purely technical, privacy, methodological or educational aspects.	yes	The study uses tabular EHR data, which meets the criterion for tabular data generation.	yes	The study uses real-world data from 9,121 human patients with lymphoma, breast, or thoracic cancer.	yes	This appears to be an original research article with novel methodology and results.	yes	The study actively creates synthetic data using PKPD modeling for data augmentation, though it's not explicitly stated if AI methods are used for this generation.	maybe	While synthetic data is generated, it's unclear if AI methods (GANs, VAEs) are used. The abstract mentions PKPD modeling for simulation, which may not qualify as AI-based generation.	yes	The study has direct clinical applications in predicting neutropenia and supporting precision dosing decisions.	yes	The study includes empirical evaluation with clear metrics and comparison between different approaches.	yes	Clear quantifiable outcomes are reported (precision and recall metrics for different models).	Exclude	A clinical study using electronic health records to predict chemotherapy-induced neutropenia demonstrates strong alignment with most screening criteria, including human clinical data, quantifiable outcomes, and direct clinical applications. The primary uncertainty lies in the synthetic data generation method, which may not strictly qualify as AI-based, potentially impacting the study's eligibility. Despite this ambiguity, the research presents a robust approach to precision medicine with clear empirical evaluation.	3.5	Exclude	Exclude	No synthetic data generation
Exploring the Utilization of Synthetic Data in Unsupervised Clustering for Opioid Misuse Analysis.	Zhang, Yili, Dong, Jia Li, Xue, Bai, Xiong, Yanbao, Gupta, Samir, Segbroeck, Maarten Van, Shara, Nawar, McGarvey, Peter	2024	maybe	The study appears to focus more on the technical aspects of synthetic data generation and clustering rather than direct clinical applications, even though it involves clinical data (opioid misuse).	yes	The study clearly focuses on tabular data, including demographics and diagnosis codes.	yes	The study uses real-world medical data from 2017-2019, which implies human clinical data.	yes	This appears to be an original research article with empirical evaluation.	yes	The abstract indicates active creation of synthetic data using GAN models.	yes	The study explicitly uses GAN models for synthetic data generation.	maybe	While the study involves opioid misuse analysis, it's not clear if it demonstrates direct clinical applications like diagnosis or treatment planning. The focus seems more methodological.	yes	The study includes empirical evaluation with clear comparison scenarios (TRTR, TRTS, TSTR, TSTS).	maybe	The study mentions evaluating "effectiveness" across different scenarios, but specific clinical metrics aren't detailed in the abstract.	Exclude	The study demonstrates strong technical capabilities in synthetic data generation using GAN models for opioid misuse analysis, with clear empirical evaluation across multiple scenarios. While the research involves human clinical data and tabular format, its primary focus appears more methodological than clinically applied, with limited direct demonstration of clinical utility or specific patient-centered outcomes. The work primarily addresses privacy and security challenges in medical data research rather than immediate clinical decision-making applications.	3.5	Exclude	Exclude	No direct clinical application
FairPRS: adjusting for admixed populations in polygenic risk scores using invariant risk minimization	Machado Reyes D., Bose A., Karavani E., Parida L.	2023	yes	The study focuses on clinical applications, specifically polygenic risk scores for patient characterization and health risk assessment.	yes	The study appears to work with tabular genetic data, though this is somewhat implicit in the abstract.	yes	The study uses human genetic data from the UK Biobank and focuses on human clinical applications.	yes	This appears to be an original research article presenting a new method (FairPRS).	maybe	While the study uses synthetic data, it's not entirely clear if they actively created it or used existing synthetic datasets. The abstract mentions "a diverse set of synthetic data" but doesn't specify if they generated it.	maybe	While synthetic data is used, the abstract doesn't specify the method of generation (whether AI-based or not).	yes	The study has direct clinical applications in risk assessment and patient characterization.	yes	The study includes empirical evaluation using both synthetic and real data from UK Biobank.	yes	The study reports on phenotype prediction performance and racial bias metrics.	Exclude	A novel approach to polygenic risk scores demonstrates potential clinical applications in genetic risk assessment across ethnic groups, utilizing both synthetic and real-world data from the UK Biobank. The study presents an original research method (FairPRS) focused on creating ancestry invariant risk scores, with empirical evaluation of phenotype prediction and racial bias metrics. While meeting most screening criteria, significant uncertainties remain regarding the specific synthetic data generation techniques and AI methodology.	3.5	Include	Exclude	No synthetic data generation
Machine Learning for Treatment Assignment: Improving Individualized Risk Attribution	Weiss J., Kuusisto F., Boyd K., Liu J., Page D.	2015	yes	The study focuses on clinical applications, specifically treatment effect estimation for statin use and myocardial infarction, and D-penicillamine use for primary biliary cirrhosis.	yes	The study appears to work with tabular data, as it deals with treatment effects and patient outcomes in a statistical framework.	yes	The study uses real clinical data for primary biliary cirrhosis and creates synthetic data for statin use and MI, which are human clinical conditions.	yes	This appears to be an original research article presenting new methodology and empirical results.	maybe	While the abstract mentions using a "synthetic data set," it's not explicitly stated whether this was created using AI methods or traditional statistical methods.	maybe	Similar to criterion 3, while synthetic data is used, the method of generation (AI vs. traditional statistical) is not specified.	yes	The study directly addresses clinical applications by focusing on treatment effect estimation, which is crucial for clinical decision-making.	yes	The study includes empirical evaluation using both synthetic and real data sets with clear metrics for assessment.	yes	The study reports quantifiable outcomes in terms of treatment effect estimation performance.	Exclude	The study demonstrates strong clinical relevance with treatment effect estimation for human medical conditions, utilizing both synthetic and real-world data sets. While the research presents original empirical findings with quantifiable outcomes in clinical decision-making, significant uncertainty exists regarding the specific method of synthetic data generation. The paper meets most screening criteria but lacks clarity on the precise AI-driven synthetic data creation process.	3.5	Include	Exclude	No AI generation
Semantic representation and comparative analysis of physical activity sensor observations using MOX2-5 sensor in real and synthetic datasets: a proof-of-concept-study	Chatterjee A., Gerdes M.W., Prinz A., Riegler M.A., Martinez S.G.	2024	maybe	The study focuses on clinical applications, but also has a heavy emphasis on technical aspects like semantic representation and data interoperability, rather than purely clinical considerations.	yes	The study deals with tabular data from activity sensors, including activity intensity, weight-bearing, sedentary, standing, etc.	yes	The study uses data from 16 human adults over 1.5 months.	yes	This appears to be an original research article with empirical evaluation.	yes	The study actively creates synthetic data using AI methods (GC, CTGAN, TABGAN).	yes	The study explicitly uses AI methods (CTGAN, TABGAN) for synthetic data generation.	maybe	While the study involves physical activity monitoring which has clinical applications, the abstract doesn't clearly demonstrate direct clinical applications like diagnosis or treatment planning.	yes	The study includes empirical evaluation comparing real and synthetic datasets and their effectiveness in predictive analysis.	maybe	The study mentions classification accuracy and model efficiency, but doesn't provide specific metrics in the abstract.	Exclude	The study demonstrates strong technical capabilities in synthetic data generation using AI methods for physical activity monitoring, with clear empirical evaluation and human clinical data involvement. However, the research appears more methodologically oriented, with limited direct clinical application demonstration and quantifiable clinical outcomes. The work primarily focuses on data generation techniques and semantic representation rather than explicit clinical utility.	3.5	Exclude	Exclude	No direct clinical application
Synthetic Generation of Patient Service Utilization Data: A Scalability Study.	Howie, Joseph, Balasubramanian, Sowmya, Bambi, Jonas, Moselle, Kenneth, Srinivasan, Venkatesh, Thomo, Alex	2024	maybe	The study focuses on synthetic data generation for healthcare, but appears to emphasize technical aspects (scalability, efficiency) rather than direct clinical applications.	yes	The study focuses on patient service utilization data, which is typically tabular data.	yes	The study uses data from a Canadian health authority, which implies human clinical data.	yes	This appears to be an original research article evaluating different synthetic data generation methods.	yes	The study actively creates synthetic data using various AI methods (GANs, VAEs, copulaGAN, transformers).	yes	The study explicitly uses AI methods (GANs, VAEs, copulaGAN, transformers) for synthetic data generation.	maybe	While the abstract mentions "practical utility" and "real-world applications," it doesn't explicitly demonstrate direct clinical applications like diagnosis or treatment planning.	maybe	While there is empirical evaluation of the methods, the focus appears to be on technical metrics rather than clinical utility.	maybe	The study measures efficiency and data resemblance, but doesn't clearly report clinical metrics.	Exclude	The study demonstrates strong technical capabilities in synthetic data generation using AI methods for healthcare data, with clear evidence of original research and human clinical data usage. However, the research appears more focused on methodological efficiency and privacy considerations rather than direct clinical applications, with limited demonstration of quantifiable clinical outcomes or utility.	3.5	Exclude	Exclude	No direct clinical application

Evaluating generalizability of oncology trial results to real-world patients using machine learning-based trial emulations	Orcutt X., Chen K., Mamtani R., Long Q., Parikh R.B.	2025	yes	The study focuses on clinical applications, specifically evaluating the generalizability of RCTs in oncology and their real-world applicability.	yes	The study appears to work with tabular electronic health record data from Flatiron Health.	yes	The study uses real-world electronic health records from human patients with cancer.	yes	This appears to be an original research article with novel methodology and results.	maybe	While the study mentions "semi-synthetic data simulation," it's not clear if this involves active AI-based synthetic data generation or just statistical manipulation of existing data.	maybe	The abstract mentions "semi-synthetic data simulation" but doesn't specify the methods used. It's not clear if AI methods like GANs or VAEs were employed.	yes	The study has direct clinical applications in evaluating treatment effectiveness and generalizability of RCTs.	yes	The study includes empirical evaluation with clear metrics and robustness assessment.	yes	The study reports quantifiable outcomes including survival times and treatment-associated survival benefits.	Exclude	A clinical study using electronic health records to evaluate oncology treatment generalizability, demonstrating strong alignment with most screening criteria including human clinical data, quantifiable outcomes, and direct clinical applications. The primary uncertainty lies in the precise methodology of synthetic data generation, with partial evidence of semi-synthetic data simulation that does not definitively confirm AI-based synthetic data creation.	3.4	Exclude	Exclude	No synthetic data generation
Improvement of an External Predictive Model Based on New Information Using a Synthetic Data Approach: Application to CADASIL	Chhoa H., Chabriat H., Anato A.J., Bamba M., Zittoun F., Chevret S., Biard L.	2023	yes	The study focuses on clinical applications, specifically improving predictive models for CADASIL disease progression and cognitive decline.	yes	The study appears to work with tabular data (demographic, clinical, and genetic information) rather than imaging or other non-tabular formats.	yes	The study uses data from 367 human patients with CADASIL.	yes	This appears to be an original research article with clear methodology and results.	maybe	While the abstract mentions "synthetic data," it's not explicitly clear if this involves AI-based generation or other statistical methods. The description suggests it might be a statistical imputation approach rather than AI-generated synthetic data.	maybe	Similar to criterion 3, while synthetic data is used, the method appears to be more statistical ("multiple imputation method") rather than AI-based GANs or VAEs.	yes	The study has direct clinical applications in predicting disease progression and cognitive decline in CADASIL patients.	yes	The study includes empirical evaluation with clear metrics and cross-validation results.	yes	The study reports quantifiable outcomes, specifically changes in Mattis Dementia Rating Scale (MDRS) scores.	Exclude	A clinical study on CADASIL disease progression using synthetic data demonstrates strong alignment with most screening criteria, including human clinical data, quantifiable outcomes, and direct clinical applications. The primary uncertainty lies in the method of synthetic data generation, with potential ambiguity around whether AI-based techniques were employed. Despite this nuance, the research presents a robust approach to predictive modeling in neurological disease progression.	3.4	Include	Exclude	No AI generation
Anonymization through data synthesis using generative adversarial networks (ADS-GAN)	Yoon J., Drumright L.N., Van Der Schaar M.	2020	maybe	The paper focuses on generating synthetic EHR data for AI applications in medicine, but appears to emphasize the technical and privacy aspects rather than specific clinical applications. While it mentions "enabling more accurate decisions and personalized treatment," this seems more aspirational than demonstrated.	yes	The study clearly focuses on tabular EHR data generation, with no mention of imaging or other non-tabular formats.	yes	The study uses real EHR data from human patients as its basis.	yes	This appears to be an original research article presenting a novel framework and empirical evaluation.	yes	The paper describes active creation of synthetic data using a novel GAN framework (ADS-GAN), not using pre-existing synthetic datasets.	yes	The study explicitly uses AI methods (specifically GANs) for synthetic data generation.	no	While the paper mentions potential clinical applications, it doesn't demonstrate direct clinical applications. The focus is on the technical aspects of synthetic data generation and privacy preservation.	maybe	The study includes empirical evaluation comparing models fitted to synthetic vs. real data across four datasets, but the metrics focus on technical performance rather than clinical utility.	maybe	The study reports quantifiable outcomes related to model performance and identifiability metrics, but these are not clinical outcomes.	Exclude	The study presents a novel AI framework for generating synthetic electronic health records, focusing on technical data generation and privacy preservation rather than direct clinical applications. While the research demonstrates sophisticated synthetic data creation using GANs and includes empirical evaluation across multiple datasets, it lacks clear demonstration of clinical utility or quantifiable clinical outcomes. The work primarily addresses methodological challenges in data sharing and privacy, with potential future implications for medical AI applications.	3.2	Exclude	Exclude	No direct clinical application
Characterization of Synthetic Health Data Using Rule-Based Artificial Intelligence Models.	Lenatti, Marta, Paglialonga, Alessia, Orani, Vanessa, Ferretti, Melissa, Mongelli, Maurizio	2023	maybe	The paper focuses on evaluating synthetic health data quality using XAI, which is more methodological than clinical. While it uses hearing screening data, the primary focus is on the technical evaluation of synthetic data rather than clinical applications.	yes	The study uses tabular data from hearing screening, which meets this criterion.	yes	The study uses adult hearing screening data, which is human clinical data.	yes	This appears to be an original research article with empirical evaluation, not a review or commentary.	yes	The study actively creates synthetic data using a conditional GAN, meeting this criterion.	yes	The study explicitly uses GANs for synthetic data generation, clearly meeting this criterion.	no	The paper focuses on evaluating synthetic data quality rather than demonstrating direct clinical applications. While hearing screening is a clinical domain, the study doesn't demonstrate direct clinical utility.	yes	The study includes clear empirical evaluation with specific metrics for assessing synthetic data quality.	yes	The study reports quantifiable outcomes including classification performance and rule similarity metrics.	Exclude	The study demonstrates strong technical capabilities in synthetic data generation using GANs and XAI, with clear empirical evaluation of hearing screening data. However, the research primarily focuses on methodological assessment of synthetic data quality rather than direct clinical applications, which limits its clinical utility. While meeting most technical screening criteria, the study falls short in demonstrating practical clinical outcomes.	3.2	Exclude	Exclude	No direct clinical application
Classification and Automated Interpretation of Spinal Posture Data Using a Pathology-Independent Classifier and Explainable Artificial Intelligence (XAI).	Dindorf, Carlo, Konradi, Jurgen, Wolf, Claudia, Taetz, Bertram, Bleser, Gabriele, Huthwelker, Janine, Werthmann, Friederike, Bartaguiz, Eva, Kniepert, Johanna, Drees, Philipp, Betz, Ulrich, Frohlich, Michael	2021	yes	The study focuses on clinical classification of spinal posture data for diagnosis and therapy monitoring, which is clearly a clinical application.	maybe	While the study mentions "spinal posture data," it's not explicitly stated whether this is tabular data. Given the mention of "dynamic spinal data," this might include time-series data, which would violate this criterion.	yes	The study uses data from human subjects including healthy subjects and those with various pathologies.	yes	This appears to be an original research article with methodology and results.	maybe	The abstract mentions using synthetic data, but it's unclear if they actively created it using AI or used pre-existing synthetic datasets.	maybe	While synthetic data is mentioned, the abstract doesn't specify the method used for generation (whether AI-based or not).	yes	The study demonstrates direct clinical applications in diagnosis and therapy monitoring.	yes	The study includes empirical evaluation with clear comparison metrics.	yes	The study reports classification results and comparisons with binary classification approaches.	Exclude	A clinical study exploring spinal posture classification using synthetic data demonstrates potential for pathology-independent diagnostic approaches with quantifiable outcomes. While the research involves human clinical data and shows promising clinical applications, uncertainties exist regarding the precise data format and synthetic data generation methodology. The study's focus on developing an interpretable classification model for spinal health suggests innovative clinical research with potential therapeutic monitoring implications.	3.2	Exclude	Exclude	No tabular data
Clinical Decision Support Systems: From the Perspective of Small and Imbalanced Data Set.	Par, Oznur Esra, Akcapinar Sezer, Ebru, Sever, Hayri	2019	yes	The study focuses on clinical decision support systems for diagnosis, which is a clinical application rather than purely technical aspects.	yes	The study appears to work with tabular data (hepatitis dataset) and doesn't mention any non-tabular formats.	yes	The study uses hepatitis dataset, which is a human clinical dataset.	yes	This appears to be an original research article with empirical evaluation.	maybe	While the study generates synthetic data, it uses "distance-based data generation methods" rather than explicitly mentioned AI methods like GANs or VAEs.	maybe	The study generates synthetic data, but the method used (distance-based oversampling) may not qualify as an AI method as specified in the criteria.	yes	The study focuses on clinical decision support systems for diagnosis.	yes	The study includes empirical evaluation using four different machine learning algorithms and reports classification scores.	yes	The study reports classification scores and recommends optimal synthetic data generation rates.	Exclude	The study addresses clinical decision support systems using a hepatitis dataset, demonstrating empirical evaluation of synthetic data generation through machine learning algorithms. While the research meets most screening criteria related to clinical applications and human data, there is uncertainty regarding the specific AI methods used for synthetic data generation. The approach focuses on addressing imbalanced datasets through distance-based oversampling, which may not fully align with the strict AI-based synthetic data generation requirements.	3.2	Exclude	Exclude	No synthetic data generation
Comparative Analysis of Data Generation Techniques for Breast Cancer Research Using Artificial Intelligence.	Pope, Tia M, Patooghy, Ahmad	2024	maybe	The study focuses more on the technical aspects of generating synthetic data using ChatGPT rather than direct clinical applications.	yes	The study focuses on tabular data (Wisconsin Breast Cancer Dataset), which is a standard tabular dataset.	yes	The study uses the Wisconsin Breast Cancer Dataset, which is based on human clinical data.	yes	This appears to be an original research article with empirical evaluation.	yes	The study involves active creation of synthetic data using ChatGPT, a form of AI.	yes	The study explicitly focuses on generating synthetic tabular data using AI (ChatGPT).	no	The abstract doesn't demonstrate direct clinical applications. It focuses on the technical aspects of data generation rather than clinical use cases.	yes	The study includes empirical evaluation with clear metrics for assessing synthetic data quality.	maybe	While the study mentions "statistical analysis, structural similarity metrics, and machine learning performance," it's unclear if these are specifically related to clinical outcomes.	Exclude	The study demonstrates strong technical capabilities in synthetic data generation using ChatGPT for breast cancer research, meeting multiple methodological criteria. However, the research lacks clear direct clinical applications and quantifiable clinical outcomes, focusing more on the technical process of data generation rather than practical healthcare utility. While empirically sound, the study falls short of fully addressing clinical implementation and patient-centered objectives.	3.2	Exclude	Exclude	No direct clinical application
GEMDiff: a diffusion workflow bridges between normal and tumor gene expression states: a breast cancer case study	Ai X., Smith M.C., Feltus F.A.	2025	maybe	The paper focuses on a technical method (GEMDiff) for gene expression analysis, but its primary application is clinical (breast cancer). However, the focus appears to be more on the technical aspects of the method rather than direct clinical applications.	maybe	The study deals with RNA-seq data and gene expression data, which are typically tabular formats. However, it's not entirely clear if the synthetic data generation is strictly tabular or includes other formats.	maybe	The study uses human breast cancer data, though it's unclear if this is directly from patients or from public databases.	yes	This appears to be an original research article with novel methodology.	yes	The study involves active creation of synthetic data using a diffusion model.	yes	The study uses AI methods (specifically a diffusion model) to generate synthetic data.	maybe	While the study has potential clinical applications (drug design and treatment strategies), it doesn't directly demonstrate clinical applications. The focus is more on the technical aspects of gene expression analysis.	maybe	While there is some empirical evaluation through a case study, the metrics for assessing clinical utility are not clearly presented.	maybe	The abstract mentions identifying 307 core genes but doesn't provide clear clinical metrics or performance measures.	Exclude	A novel computational workflow for gene expression analysis demonstrates synthetic data generation using AI methods, with potential clinical implications in breast cancer research. While the study involves human clinical data and original research, its primary focus remains on technical methodology rather than direct clinical applications, with limited demonstration of quantifiable clinical outcomes. The research presents a promising approach to gene expression analysis but falls short of fully meeting comprehensive clinical utility criteria.	3.2	Exclude	Exclude	No tabular data
Generating high-fidelity synthetic patient data for assessing machine learning healthcare software	Tucker A., Wang Z., Rotalinti Y., Myles P.	2020	maybe	The paper focuses on generating synthetic data for healthcare applications, but appears to be primarily methodological in nature, focusing on the technical aspects of synthetic data generation rather than specific clinical applications. While it mentions "improve diagnosis," this seems secondary to the technical focus.	yes	The abstract indicates they work with tabular data from UK primary care patient data, meeting this criterion.	yes	The study uses UK primary care patient data, which is human clinical data.	yes	Based on the abstract's structure and content, this appears to be an original research article.	yes	The study involves active creation of synthetic data using AI methods, including "resampling, probabilistic graphical modelling, latent variable identification."	yes	The study clearly involves AI methods for generating synthetic tabular data, though specific AI techniques (GANs, VAEs) aren't explicitly mentioned.	maybe	The paper only broadly mentions potential applications for diagnosis and understanding disease, but doesn't demonstrate specific clinical applications. This is a significant weakness.	yes	The study includes empirical evaluation, comparing synthetic data with ground truth data and analyzing re-identification risks.	maybe	The study reports some quantifiable outcomes, including "sensitivity analysis statistics" and comparisons with ground truth data, but these appear to be more technical metrics rather than clinical outcomes.	Exclude	The study demonstrates strong technical capabilities in synthetic data generation using UK primary care patient data, with robust methodological approaches to creating realistic synthetic datasets. However, the research lacks direct clinical applications and quantifiable clinical outcomes, focusing more on technical aspects of data generation than specific healthcare interventions. While meeting several screening criteria related to data format and generation, the paper falls short in demonstrating clear clinical utility and practical healthcare impact.	3.2	Exclude	Exclude	No direct clinical application
Generation and evaluation of synthetic patient data	Goncalves A., Ray P., Soper B., Stevens J., Coyle L., Sales A.P.	2020	maybe	The paper focuses on clinical applications in medicine and cancer research, though it appears to be more methodological in nature. It discusses synthetic data generation for medical applications, but doesn't directly demonstrate clinical applications.	yes	The abstract mentions "high dimensional and often categorical" medical data, suggesting tabular data. No mention of imaging or other non-tabular formats.	yes	Uses real cancer registry data from SEER program, which is human clinical data.	yes	Appears to be an original research article evaluating different synthetic data generation approaches.	yes	The study actively creates synthetic data using AI methods (probabilistic models, classification-based imputation, and GANs).	yes	Clear focus on AI methods for synthetic data generation, including GANs and other approaches.	no	While the paper discusses medical applications, it doesn't demonstrate direct clinical applications like diagnosis or treatment planning. Instead, it focuses on evaluating synthetic data generation methods.	maybe	Includes empirical evaluation of synthetic data generation methods, but focuses on technical evaluation rather than clinical utility.	maybe	The abstract mentions "metrics for evaluating the quality of the generated synthetic datasets" but doesn't specify clinical outcomes.	Exclude	The study demonstrates strong methodological approaches to synthetic data generation in medical research, utilizing human clinical data and AI techniques across multiple cancer types. While the research effectively explores synthetic data creation methods, it lacks direct clinical applications and quantifiable clinical outcomes, positioning it more as a technical exploration than a clinical intervention study. The work provides valuable insights into synthetic data generation but does not directly demonstrate clinical utility or diagnostic performance.	3.2	Exclude	Exclude	No direct clinical application

Groundwater health probability risk prediction through oral intake using advanced optimization methods.	Abdu, Fahad Jibrin, Abba, Sani I, Usman, Jamilu, Alowaifeer, Maad, Aljundi, Isam H	2025	maybe	The study focuses on cancer risk prediction from groundwater intake, which is a clinical/health application. However, it appears to be more focused on environmental health risk assessment rather than direct clinical applications.	maybe	The study appears to use tabular data for prediction models, though the exact format isn't explicitly stated. The focus on regression models suggests tabular data.	maybe	While the study relates to human health, it's unclear if it uses actual patient data or clinical records. It appears to focus more on environmental exposure data.	yes	This appears to be an original research article with empirical evaluation and novel findings.	yes	The study explicitly mentions using "generative AI-driven synthetic data" alongside real data, indicating active creation of synthetic data.	yes	The study uses AI methods to generate synthetic data, though the specific AI methods (GANs, VAEs, etc.) aren't specified.	maybe	The study focuses on cancer risk prediction, but it's more of an environmental health risk assessment rather than direct clinical diagnosis or treatment.	yes	The study includes thorough empirical evaluation with clear metrics comparing real and synthetic data performance.	yes	The study reports clear quantifiable outcomes including MAE, MSE, and RMSE metrics for model performance.	Exclude	The study demonstrates partial alignment with clinical research criteria, focusing on cancer risk prediction through AI-driven synthetic data generation with quantifiable outcomes. While the research involves health-related modeling and empirical evaluation, it leans more towards environmental health risk assessment rather than direct clinical applications, with uncertain use of patient-specific clinical data. The methodology employs advanced machine learning techniques to predict cancer risk from groundwater intake, but falls short of meeting all strict clinical research screening requirements.	3.2	Exclude	Exclude	No tabular data
Learning the progression patterns of treatments using a probabilistic generative model	Zaballa O., Perez A., Gomez Inhiesto E., Acaiturri Ayesta T., Lozano J.A.	2023	maybe	The paper focuses on clinical applications, specifically modeling treatments and disease progression using EHR data. However, it appears to be more methodological in nature, focusing on the probabilistic generative model rather than direct clinical applications.	maybe	The study deals with sequences of medical activities, which could be considered tabular data, but it's specifically focused on sequential data which may not strictly meet the tabular data requirement.	maybe	The study uses Electronic Health Records, which implies human clinical data, though this isn't explicitly stated.	yes	This appears to be an original research article presenting a new probabilistic generative model.	yes	The paper does involve active creation of synthetic data using AI methods, as evidenced by their use of "synthetic data" for evaluation and mention of "data augmentation."	maybe	While the paper uses synthetic data, it's not entirely clear if they're using AI methods specifically for synthetic data generation. The focus seems to be on the probabilistic generative model for treatment sequences rather than synthetic data generation.	maybe	While the paper mentions potential applications for "classification, simulation, data augmentation and missing data imputation," it doesn't clearly demonstrate direct clinical applications like diagnosis or treatment planning.	maybe	The study includes empirical evaluation, but it's primarily focused on model performance rather than clinical utility assessment.	maybe	The abstract mentions evaluation but doesn't specify clear clinical metrics or outcomes. The evaluation appears to be more focused on model performance rather than clinical utility.	Exclude	A probabilistic generative model for medical treatment sequences demonstrates potential for synthetic data generation and clinical applications, but lacks clear direct clinical outcomes and quantifiable metrics. While the study involves human clinical data and original research, its methodological focus and sequential data format create uncertainty about full alignment with screening criteria. The research shows promise for data augmentation and treatment classification, but falls short of definitive clinical utility demonstration.	3.2	Exclude	Exclude	No tabular data
NFDI4Health Workflow and Service for Synthetic Data Generation, Assessment and Risk Management.	Moazemi, Sobhan, Adams, Tim, Ng, Hwei Geok, Kuhnel, Lisa, Schneider, Julian, Naher, Anatol-Fiete, Fluck, Juliane, Frohlich, Holger	2024	maybe	The paper focuses on synthetic data generation tools and services, but it's not entirely clear if these are being applied to specific clinical problems. The abstract mentions ADNI and RKI data but doesn't specify clinical applications.	maybe	The abstract doesn't explicitly state whether the data is tabular or includes other formats. Given the mention of ADNI (which typically includes imaging data) and RKI (which could include various data types), there's uncertainty about the data format.	yes	The study uses real patient data from ADNI and RKI, which are human clinical datasets.	yes	This appears to be an original research article presenting new tools and services.	yes	The paper clearly involves active creation of synthetic data using AI tools (VAMBN and MultiNODEs).	yes	The study explicitly focuses on AI-based synthetic data generation using specific tools.	no	While the paper mentions using ADNI and RKI data, it doesn't explicitly demonstrate direct clinical applications. The focus seems more methodological.	maybe	While the paper includes evaluation through SYNDAT, it's unclear if this includes clinical utility assessment.	maybe	The abstract mentions "quality and risk" assessment of synthetic data, but doesn't specify clinical metrics or outcomes.	Exclude	The study presents AI-driven synthetic data generation tools using human clinical datasets, demonstrating methodological innovation in data privacy and synthetic data creation. While the research involves original data generation and uses real patient information, there are uncertainties regarding direct clinical applications and specific quantifiable outcomes. The work appears more focused on technical data generation methods than explicit clinical utility.	3.2	Exclude	Exclude	No direct clinical application
Performance evaluation of the machine learning algorithms used in inference mechanism of a medical decision support system.	Bal, Mert, Amasyali, M Fatih, Sever, Hayri, Kose, Guven, Demirhan, Ayse	2014	maybe	The paper focuses on medical decision support systems, which is clinical in nature. However, it appears to be more focused on comparing machine learning methods rather than specific clinical applications.	yes	The study uses synthetic tabular data (mentions "records"), meeting this criterion.	maybe	The abstract doesn't explicitly mention using real human clinical data as a basis. It mentions the ALARM network, which is a medical simulation model, but doesn't specify if it's based on real patient data.	yes	This appears to be an original research article comparing machine learning methods.	maybe	The abstract mentions "synthetic data with 10, 100, 1000, and 2000 records have been produced," suggesting original creation. However, it's unclear if AI methods were used for generation.	maybe	While synthetic data is used, the abstract doesn't specify the generation method. It mentions various machine learning methods but not for data generation.	maybe	While it's about medical decision support systems, the abstract doesn't demonstrate specific clinical applications or outcomes. It's more focused on comparing machine learning methods.	yes	The study includes empirical evaluation comparing 11 machine learning methods.	yes	The study mentions comparing accuracy of machine learning methods, suggesting quantifiable outcomes.	Exclude	A medical decision support system study comparing machine learning methods using synthetic data, with uncertain details about AI-based data generation and real clinical data usage. The research demonstrates empirical evaluation of machine learning techniques but lacks clear evidence of direct clinical applications or patient-related healthcare information. Quantifiable outcomes are present through accuracy comparisons, but the methodological focus limits comprehensive clinical utility assessment.	3.2	Exclude	Exclude	No synthetic data generation
Synthetic data generation for a longitudinal cohort study - evaluation, method extension and reproduction of published data analysis results.	Kuhnel, Lisa, Schneider, Julian, Perrar, Ines, Adams, Tim, Moazemi, Sobhan, Prasser, Fabian, Nothlings, Ute, Frohlich, Holger, Fluck, Juliane	2024	maybe	The paper focuses on synthetic data generation for nutrition research, which is clinical/healthcare related but not explicitly focused on clinical applications. It appears more methodological in nature, focusing on data generation and quality analysis.	maybe	The abstract doesn't explicitly specify the data format, but mentions "individual-level health data" and "statistical properties," suggesting tabular data. However, this isn't definitively stated.	maybe	The study uses health data, but it's not explicitly stated whether it's human clinical data. The nutrition focus suggests human data, but this isn't certain.	yes	This appears to be an original research article with empirical evaluation.	yes	The study clearly involves active creation of synthetic data using AI methods ("state-of-the-art synthetic data generation method").	yes	The study explicitly focuses on synthetic data generation using AI methods.	no	The paper focuses on nutrition research but doesn't demonstrate direct clinical applications like diagnosis or treatment planning. It appears more focused on data generation methodology.	yes	The study includes empirical evaluation of the synthetic data's quality and ability to reproduce real-world analysis results.	maybe	The abstract mentions "in-depth quality analyses" and "reproduce significant real-world analysis results," suggesting quantifiable outcomes, but these appear to be focused on data quality rather than clinical outcomes.	Exclude	The study demonstrates strong capabilities in synthetic data generation using AI methods, with clear empirical evaluation and original research focus. However, significant uncertainties exist regarding clinical application, data format, and direct healthcare utility, suggesting a more methodological approach to data generation rather than a clinically-oriented research study. The paper partially meets screening criteria, with notable gaps in demonstrating direct clinical relevance and outcomes.	3.2	Exclude	Exclude	No direct clinical application
Data-Augmented Modeling of Intracranial Pressure	Wang J.-X., Hu X., Shadden S.C.	2019	yes	The study focuses on clinical applications, specifically noninvasive ICP estimation for cerebral disease management.	maybe	While the study mentions "noninvasive measurements" and "synthetic data," it's not explicitly clear if these are tabular data. The focus on ICP measurements and cerebral blood flow suggests possible time-series data, which would not meet the criterion.	yes	The study includes human clinical data, specifically mentioning "two patients" in the preliminary clinical application.	yes	This appears to be an original research article with novel methodology and empirical evaluation.	yes	The study mentions "virtual experiments with synthetic data" and appears to actively generate synthetic data for their framework.	maybe	While synthetic data is used, the abstract doesn't specify the AI methods used for generation. The focus seems to be on a Bayesian framework rather than AI-based synthetic data generation.	yes	The study has direct clinical applications in ICP monitoring and estimation for cerebral disease management.	yes	The study includes both virtual experiments and preliminary clinical application with empirical evaluation.	maybe	While the abstract mentions "improve ICP prediction," it doesn't explicitly detail the quantifiable metrics used.	Exclude	A clinical study exploring noninvasive intracranial pressure estimation using a Bayesian framework with synthetic data generation, demonstrating potential clinical applications in cerebral disease management. The research involves human patient data and includes both virtual experiments and preliminary clinical validation, though uncertainties exist regarding the precise data format and synthetic data generation methodology.	3.1	Exclude	Exclude	No tabular data
How Useful Is Synthetic Data in Developing Predictive Models for Health?	Basri M.A., Chen H.	2025	maybe	The abstract focuses on technical evaluation of synthetic data quality and utility for predictive models, but does not explicitly mention clinical applications.	yes	The abstract specifically mentions "tabular data" and focuses on this format.	maybe	While the abstract mentions "sensitive health data," it doesn't explicitly specify whether this is human clinical data.	yes	This appears to be an original research article with empirical evaluation.	yes	The study involves active creation of synthetic data using "generative AI techniques."	yes	The study clearly involves AI-based synthetic data generation, though specific methods (GANs, VAEs) aren't mentioned.	no	The abstract focuses on technical evaluation (fidelity and utility for predictive models) rather than direct clinical applications.	yes	The study includes clear empirical evaluation with specific metrics for assessing synthetic data quality.	yes	The study reports quantifiable outcomes including model performance metrics and correlation between synthetic and real data performance.	Exclude	The study demonstrates strong technical capabilities in synthetic data generation using AI techniques, with clear empirical evaluation of tabular data quality and predictive model performance. However, the abstract lacks explicit evidence of direct clinical applications or confirmation of human clinical data usage, which creates uncertainty about its alignment with clinical research criteria. The research appears more methodologically focused on synthetic data generation techniques rather than specific clinical utility.	3.1	Exclude	Exclude	No direct clinical application
A method to combine target volume data from 3D and 4D planned thoracic radiotherapy patient cohorts for machine learning applications.	Johnson, Corinne, Price, Gareth, Khalifa, Jonathan, Faivre-Finn, Corinne, Dekker, Andre, Moore, Christopher, van Herk, Marcel	2018	yes	The study focuses on clinical applications in radiotherapy planning for lung cancer, specifically dealing with tumor volume measurements and their use in machine learning models.	maybe	While the study deals with medical data, it's not clear if the data is purely tabular. The focus seems to be on volumetric data (GTV/iGTV) which may involve imaging data.	yes	The study uses data from 40 non-small cell lung cancer patients.	yes	This appears to be an original research article with clear methodology and results.	yes	The study actively creates synthetic data through linear fit and erosion methods to synthesize GTV volumes from iGTV data.	maybe	While the study generates synthetic data, it uses linear fit and erosion methods rather than AI methods like GANs or VAEs. The synthesis appears to be more statistical/mathematical than AI-based.	yes	The study has direct clinical applications in radiotherapy planning and machine learning models for treatment.	yes	The study includes empirical evaluation with clear metrics and validation methods.	yes	The study reports clear quantifiable outcomes including Dice Similarity Coefficients, dosimetric features, and model performance metrics.	Exclude	A clinical study on lung cancer radiotherapy planning that generates synthetic tumor volume data using statistical methods, demonstrating empirical validation and quantifiable outcomes. While the research involves human clinical data and has direct clinical applications, the synthetic data generation approach does not strictly align with AI-based methods, and the data format appears more volumetric than purely tabular. The study's methodology and results are robust, but may not fully meet all predefined screening criteria.	2.9	Exclude	Exclude	No tabular data
A novel and fully automated platform for synthetic tabular data generation and validation	Rashidi H.H., Albahra S., Rubin B.P., Hu B.	2024	maybe	The abstract focuses on a technical solution (STNG) for healthcare data accessibility, but does not explicitly discuss specific clinical applications. It is more methodological in nature.	yes	The abstract specifically mentions "synthetic tabular neural generator" and focuses on tabular data generation.	maybe	While the abstract mentions "healthcare data," it does not explicitly specify whether this is human clinical data. The mention of "twelve different datasets" suggests real healthcare data, but this is not certain.	yes	This appears to be an original research article presenting a new system (STNG) with empirical evaluation.	yes	The study involves active creation of synthetic data using AI through their proposed STNG system.	yes	The study clearly involves AI-based synthetic data generation using neural methods.	no	The abstract does not demonstrate direct clinical applications. It focuses on the technical solution for data accessibility rather than specific clinical use cases.	yes	The abstract mentions an "empirical study" with validation and comparison across twelve datasets.	maybe	While the abstract mentions "performance" and "validation," it does not specify clinical metrics. The outcomes appear to be more technical in nature.	Exclude	A technical methodology for synthetic healthcare data generation demonstrates strong capabilities in AI-driven tabular data creation, but lacks clear direct clinical applications or specific patient-related outcomes. While the study presents an innovative approach to addressing healthcare data accessibility challenges, it falls short of providing concrete clinical utility metrics or demonstrating immediate healthcare impact.	2.9	Exclude	Exclude	No direct clinical application
A simulated trial with reinforcement learning for the efficacy of Irinotecan and Ifosfamide versus Topotecan in relapsed, extensive stage small cell lung cancer	Bozcuk H.S., Artac M.	2024	yes	The study focuses on clinical applications, specifically comparing treatment outcomes for recurrent SCLC patients.	yes	The study appears to use tabular data for patient characteristics and outcomes, though this isn't explicitly stated.	yes	The study is based on human clinical data, specifically SCLC patients, though it uses simulated rather than real patient data.	yes	This is clearly an original research article with novel findings.	maybe	While the study creates synthetic data, it uses Cox proportional hazards modeling and Reinforcement Learning rather than traditional AI synthetic data generation methods like GANs or VAEs.	maybe	The study generates synthetic data, but uses statistical modeling (Cox proportional hazards) and RL rather than the specified AI methods (GANs, VAEs).	yes	The study demonstrates direct clinical applications by comparing treatment outcomes and survival rates.	yes	The study includes empirical evaluation with clear metrics and statistical analysis.	yes	The study reports clear quantifiable outcomes including hazard ratios, survival times, and statistical significance.	Exclude	A clinical study comparing treatment outcomes for recurrent small cell lung cancer patients using synthetic data generation through statistical modeling and Reinforcement Learning. While demonstrating strong clinical application and quantifiable outcomes, the synthetic data generation method deviates from the specified AI techniques like GANs or VAEs. The research provides valuable insights into treatment comparisons but may not fully align with the targeted synthetic data generation criteria.	2.9	Exclude	Exclude	No synthetic data generation

Application of Bayesian networks to generate synthetic health data	Kaur D., Sobiesk M., Patil S., Liu J., Bhagat P., Gupta A., Markuzon N.	2021	maybe	The paper focuses on generating synthetic data for research purposes rather than direct clinical applications. While it has healthcare implications, it's primarily a methodological paper about data generation.	yes	The study clearly focuses on tabular data, using datasets like UCI heart disease, diabetes, and MIMIC-III diagnoses. No mention of imaging or other non-tabular formats.	yes	The study uses real clinical datasets (UCI heart disease, diabetes, and MIMIC-III), which are based on human patient data.	yes	This appears to be an original research article with clear methodology and results sections.	yes	The study actively creates synthetic data using Bayesian networks, comparing it with medBGAN. This is original AI-based synthetic data generation.	yes	The study uses AI methods (Bayesian networks) to generate synthetic tabular data, comparing it with medBGAN.	no	The paper focuses on data generation methodology rather than direct clinical applications. While it mentions enabling researchers to "generate hypotheses and develop analytical tools," it doesn't demonstrate specific clinical applications.	maybe	The study includes clear empirical evaluation with multiple metrics, but these are focused on data generation quality rather than clinical utility.	maybe	The study reports quantifiable outcomes in terms of statistical tests, machine learning tasks, and disclosure risk metrics. However, these are focused on data quality rather than clinical outcomes.	Exclude	The study demonstrates strong methodological rigor in synthetic data generation using Bayesian networks, successfully creating tabular healthcare data with high fidelity to original datasets. However, the research lacks direct clinical applications and quantifiable clinical outcomes, focusing instead on data generation methodology for research purposes. While meeting several technical criteria, the paper falls short of demonstrating immediate clinical utility or decision-making impact.	2.9	Exclude	Exclude	No direct clinical application
Assessment of differentially private synthetic data for utility and fairness in end-to-end machine learning pipelines for tabular data	Pereira M., Kshirsagar M., Mukherjee S., Dodhia R., Ferres J.L., de Sousa R.	2019	maybe	The paper focuses on technical aspects of synthetic data generation and privacy preservation, rather than specific clinical applications. While it mentions healthcare as an area of impact, the study itself is methodological in nature.	yes	The study explicitly focuses on tabular data generation, which meets this criterion.	maybe	While healthcare is mentioned as an application area, the abstract doesn't specify whether the study uses actual human clinical data. This is unclear from the abstract.	yes	This appears to be an original research article with novel contributions and empirical evaluation.	yes	The study involves active creation of synthetic data using AI methods (both marginal-based and GAN-based approaches).	yes	The paper clearly involves AI methods for synthetic data generation, including GANs and marginal-based algorithms.	no	The study focuses on general machine learning pipelines and privacy preservation rather than specific clinical applications. It doesn't demonstrate direct clinical applications.	maybe	The study includes comprehensive empirical evaluation with clear metrics, but these are focused on technical performance rather than clinical utility.	maybe	The study reports quantifiable outcomes related to utility and fairness metrics, but these are not clinical metrics.	Exclude	The study primarily focuses on technical aspects of synthetic data generation and privacy preservation, with limited direct clinical applications. While the research involves AI-driven tabular data generation and includes empirical evaluation, it lacks clear demonstration of clinical utility or use of human clinical data. The methodological approach emphasizes privacy and machine learning performance rather than specific healthcare outcomes.	2.9	Exclude	Exclude	No direct clinical application
Combining propensity score methods with variational autoencoders for generating synthetic data in presence of latent sub-groups.	Farhadyar, Kiana, Bonofiglio, Federico, Hackenberg, Maren, Behrens, Max, Zoller, Daniela, Binder, Harald	2024	maybe	The paper focuses on technical aspects of synthetic data generation rather than clinical applications. While it mentions clinical cohorts and a stroke trial, the primary focus is on the methodological approach to preserving heterogeneity in synthetic data.	yes	The study appears to work with tabular data, as evidenced by discussion of marginal distributions and propensity scores. No mention of imaging or other non-tabular formats.	yes	The study uses data from an international stroke trial, which implies human clinical data.	yes	Based on the abstract's structure and content, this appears to be an original research article with novel methodology and evaluation.	yes	The study clearly involves active creation of synthetic data using AI (specifically VAEs), not just using pre-existing synthetic datasets.	yes	The paper explicitly uses AI methods (VAEs) for synthetic data generation, combined with statistical approaches.	no	The paper does not demonstrate direct clinical applications. It focuses on the technical aspects of preserving heterogeneity in synthetic data generation, without discussing specific clinical use cases.	no	The study includes empirical evaluation, but it's focused on statistical properties rather than clinical utility. The evaluation is primarily technical rather than clinical.	no	While the paper mentions evaluation, it appears to focus on statistical properties and distribution matching rather than clinical outcomes or metrics.	Exclude	The study demonstrates strong technical capabilities in synthetic data generation using AI methods, specifically variational autoencoders, with human clinical data from a stroke trial. However, the research primarily focuses on methodological aspects of preserving data heterogeneity rather than direct clinical applications or quantifiable clinical outcomes. While meeting several technical screening criteria, the paper lacks clear demonstration of clinical utility or specific healthcare decision-making metrics.	2.9	Exclude	Exclude	No direct clinical application
Comprehensive evaluation framework for synthetic tabular data in health: fidelity, utility and privacy analysis of generative models with and without privacy guarantees	Hernandez M., Osorio-Marulanda P.A., Catalina M., Loinaz L., Epelde G., Aginako N.	2025	maybe	The paper focuses heavily on technical aspects (privacy, evaluation framework) rather than clinical applications, as evidenced by the abstract mentioning "privacy-enhancing technology" and evaluation metrics more than clinical use cases.	yes	The study explicitly focuses on "synthetic tabular data" and mentions "medical datasets" without reference to other data formats.	maybe	While the study uses "medical datasets," the abstract doesn't explicitly specify whether these are human clinical data. However, the context suggests they are likely human healthcare datasets.	yes	This appears to be an original research article presenting a new evaluation framework and empirical results.	yes	The study involves active creation of synthetic data using AI methods, comparing five different generative models.	yes	The paper clearly involves AI methods for synthetic data generation, mentioning "five generative models" and discussing their performance.	no	The abstract focuses on technical evaluation metrics (fidelity, utility, privacy) rather than demonstrating direct clinical applications. While it mentions "clinical utility evaluation" as future work, this isn't part of the current study.	yes	The study includes original empirical evaluation with clear metrics, though these are technical rather than clinical.	maybe	While the study reports quantifiable metrics, these are focused on technical performance (fidelity, utility, privacy) rather than clinical outcomes.	Exclude	The study demonstrates strong technical capabilities in synthetic tabular data generation, meeting criteria for data format, original data creation, and empirical evaluation. However, it falls short on clinical application criteria, with limited demonstration of direct clinical utility or patient-centered outcomes. While methodologically rigorous, the research appears more focused on technical privacy and evaluation frameworks than practical clinical implementation.	2.9	Exclude	Exclude	No direct clinical application
Deep generative models in DataSHIELD	Lenz S., Hess M., Binder H.	2021	maybe	The paper focuses primarily on technical aspects (privacy-preserving data analysis and synthetic data generation) rather than direct clinical applications. While it mentions potential use for "pattern recognition with deep learning," this is not a specific clinical application.	yes	The study appears to work with tabular data, specifically mentioning genetic variant data and patient data. No mention of imaging or other non-tabular formats.	yes	The study uses human clinical data, specifically mentioning patient data and genetic variant data.	yes	This appears to be an original research article presenting new methodology and implementation.	yes	The study actively creates synthetic data using AI methods (DBMs, VAEs, GANs) rather than using pre-existing synthetic datasets.	yes	The study explicitly uses AI methods (DBMs, VAEs, GANs) for synthetic data generation.	no	The paper does not demonstrate direct clinical applications. It focuses on the technical methodology for generating synthetic data for privacy-preserving analysis.	maybe	The study includes empirical evaluation, but it's focused on technical aspects rather than clinical utility.	maybe	While the study mentions comparing different generative approaches, it doesn't clearly report clinical metrics or outcomes. The evaluation appears to be more technical (pattern recovery, disclosure risk).	Exclude	The study demonstrates technical proficiency in synthetic data generation using AI methods for privacy-preserving medical data analysis, but lacks direct clinical applications or quantifiable clinical outcomes. While the research meets several methodological criteria, it primarily focuses on technical implementation rather than demonstrating practical clinical utility or impact. The work provides a sophisticated approach to generating synthetic patient data, but falls short of translating this into concrete clinical decision-making or patient care improvements.	2.9	Exclude	Exclude	No direct clinical application
Generating synthetic personal health data using conditional generative adversarial networks combining with differential privacy.	Sun, Chang, van Soest, Johan, Dumontier, Michel	2023	no	The paper focuses heavily on technical aspects (DP-CGANs, privacy preservation) rather than clinical applications. While it mentions personal health data, the primary focus appears to be on the technical methodology of generating synthetic data.	yes	The study appears to work with tabular data, mentioning "categorical and continuous variables" and "personal health datasets."	maybe	While the paper mentions "personal health data" and "patient data," it's not entirely clear if this is exclusively human clinical data. The abstract also mentions "personal socio-economic datasets," suggesting a broader scope.	yes	This appears to be an original research article presenting a new method and its evaluation.	yes	The paper clearly involves active creation of synthetic data using AI (specifically DP-CGANs).	yes	The study explicitly uses AI methods (DP-CGANs) for synthetic data generation.	no	The abstract does not demonstrate direct clinical applications. The focus is on the technical aspects of data generation and privacy preservation rather than clinical use cases.	maybe	The paper includes empirical evaluation, but the metrics are technical rather than clinical.	no	The study reports quantifiable outcomes, but these are focused on statistical similarity, machine learning performance, and privacy measurements rather than clinical metrics.	Exclude	The study presents a technical approach to synthetic data generation using AI methods, focusing on privacy preservation and data transformation techniques. While demonstrating original research with empirical evaluation, the work lacks direct clinical applications and quantifiable clinical outcomes. The research appears more oriented toward methodological innovation in synthetic data generation rather than immediate clinical utility.	2.9	Exclude	Exclude	No direct clinical application
Generation of realistic synthetic data using Multimodal Neural Ordinary Differential Equations	Wendland P., Birkenbihl C., Gomez-Freixa M., Sood M., Kschischo M., Frohlich H.	2022	maybe	The paper focuses on generating synthetic patient data for clinical studies, which has clear clinical applications. However, it appears to be more methodological in nature, focusing on the technical approach (MultiNODEs) rather than specific clinical applications.	no	The abstract mentions "patient trajectories on a continuous time scale" and "longitudinal data," which suggests time-series data rather than purely tabular data.	yes	The study uses "real patient-level data from two independent clinical studies."	yes	Based on the abstract's structure and content, this appears to be an original research article.	yes	The study involves active creation of synthetic data using AI (MultiNODEs approach).	yes	The study uses AI methods (MultiNODEs, described as a "hybrid, multimodal AI approach") to generate synthetic data.	maybe	While the paper mentions enabling "smooth interpolation and extrapolation of clinical studies," it doesn't clearly demonstrate direct clinical applications like diagnosis or treatment planning. The focus seems more methodological.	maybe	The abstract mentions applying the method to real patient data and simulated epidemiological data, suggesting some empirical evaluation, but doesn't detail specific metrics or evaluation methods.	maybe	The abstract doesn't explicitly mention specific quantifiable outcomes or metrics for evaluating the synthetic data's performance.	Exclude	The study presents a novel AI approach for generating synthetic patient data, utilizing multimodal neural ordinary differential equations to create realistic patient trajectories. While demonstrating technical innovation in synthetic data generation, the research appears more methodologically focused and uses time-series data, which may limit its direct clinical applicability. The approach shows potential for clinical data synthesis but lacks clear quantifiable outcomes and direct clinical applications.	2.9	Exclude	Exclude	No tabular data
Imputing biomarker status from rwe datasets-a comparative study	Traynor C., Sahota T., Tomkinson H., Gonzalez-Garcia I., Evans N., Chappell M.	2021	yes	The study focuses on clinical applications, specifically dealing with Real-World Evidence (RWE) datasets and biomarker analysis for NSCLC (Non-Small Cell Lung Cancer).	yes	The study deals with tabular data (biomarker data).	yes	The study uses the Flatiron NSCLC dataset with 35,000 subjects, which is human clinical data.	yes	This appears to be an original research article with empirical evaluation.	maybe	While the study uses AI methods (GANs and tabular networks) for imputation, it's not clear if this constitutes "active creation of synthetic data" in the sense intended by the criteria. The focus appears to be on imputation rather than synthetic data generation for clinical use.	maybe	The study does use AI methods (GANs and tabular networks) for data generation, but primarily for imputation rather than creating synthetic datasets for clinical applications. The synthetic data experiments mentioned are for evaluation purposes.	maybe	While the study deals with clinical data, it focuses on imputation methods rather than direct clinical applications like diagnosis or treatment planning.	maybe	The study includes empirical evaluation with clear metrics, but these are focused on imputation performance rather than clinical utility.	maybe	The study reports performance metrics for imputation methods, but these aren't clinical outcomes in the traditional sense.	Exclude	The study involves clinical data and AI methods for imputation, but does not fully meet the screening criteria for synthetic data generation in clinical applications. While using human clinical data and advanced AI techniques, the research focuses more on data imputation methodology than direct clinical utility or synthetic data creation for healthcare purposes. The paper demonstrates technical sophistication in data analysis but falls short of the specific requirements for synthetic data generation in clinical contexts.	2.9	Exclude	Exclude	No synthetic data generation
LDP-GAN : Generative adversarial networks with local differential privacy for patient medical records synthesis.	Gwon, Hansle, Ahn, Imjin, Kim, Yunha, Kang, Hee Jun, Seo, Hyeram, Choi, Heejung, Cho, Ha Na, Kim, Minkyoung, Han, JiYe, Kee, Gaeun, Park, Seohyun, Lee, Kye Hwa, Jun, Tae Joon, Kim, Young-Hak	2024	maybe	The paper focuses on privacy protection and data generation methodology rather than direct clinical applications. While it mentions "advancing healthcare technologies," the primary focus appears to be on the technical aspects of privacy protection.	maybe	The abstract mentions EMR data, which is typically tabular, and doesn't indicate any non-tabular formats.	yes	The study uses EMR data, which is human clinical data.	yes	Based on the experimental results mentioned and the technical contribution described, this appears to be an original research article.	yes	The study clearly involves active creation of synthetic data using AI methods, specifically deep learning-based generative models.	yes	The paper explicitly discusses using generative models based on deep learning to create synthetic data.	no	The abstract doesn't demonstrate direct clinical applications. It focuses on privacy protection and data generation methodology rather than specific clinical use cases.	maybe	The study includes empirical evaluation, but the metrics focus on privacy and utility rather than clinical utility.	maybe	While the abstract mentions "reasonable performance" and evaluation of utility and privacy, it doesn't specify clear clinical metrics. The outcomes appear to be more technical than clinical.	Exclude	The study focuses on privacy-protected synthetic data generation using deep learning, but lacks direct clinical applications and quantifiable clinical outcomes. While the research involves human clinical data and original AI-driven synthetic data creation, its primary emphasis remains on technical methodology rather than clinical utility. The paper's technical approach to privacy protection in electronic medical records does not sufficiently demonstrate practical clinical implementation.	2.9	Exclude	Exclude	No direct clinical application
Potential predictors of type-2 diabetes risk: machine learning, synthetic data and wearable health devices	Stolfi P., Valentini I., Palumbo M.C., Tieri P., Grignolio A., Castiglione F.	2020	maybe	The study focuses on clinical applications (type 2 diabetes prediction), but appears to be primarily methodological in nature, developing a computational model.	yes	The study appears to generate tabular data (46,170 virtual subjects with clinical, physiological, and behavioral features).	maybe	The study uses "prototypical human individuals" as a basis, but it's unclear if real human clinical data was used as input.	yes	This appears to be an original research article with novel findings and methodology.	maybe	The study creates synthetic data using a computational model, but it's unclear if this uses AI methods specifically. The abstract mentions machine learning for prediction but not for data generation.	no	While synthetic data is generated, the abstract doesn't specify if AI methods (GANs, VAEs, etc.) were used for generation. It appears to be a computational/mathematical model instead.	maybe	The study aims to predict type 2 diabetes onset, which is a clinical application, but it's more focused on disease mechanism modeling than direct clinical decision-making.	maybe	While there is evaluation of the model, the abstract doesn't provide clear metrics for assessing clinical utility.	no	The abstract doesn't clearly specify quantifiable outcomes or metrics for evaluation.	Exclude	A computational model for type 2 diabetes prediction generates synthetic data from virtual subjects, but lacks clear evidence of AI-driven data generation and quantifiable clinical outcomes. While the study demonstrates potential clinical relevance through disease mechanism modeling, it falls short of fully meeting the comprehensive screening criteria for AI-driven synthetic data research.	2.9	Exclude	Exclude	No synthetic data generation
Preserving information while respecting privacy through an information theoretic framework for synthetic health data generation	Sella N., Guinot F., Lagrange N., Albou L.-P., Desponds J., Isambert H.	2025	no	The abstract focuses on technical aspects of synthetic data generation and privacy concerns, rather than specific clinical applications. While it mentions "electronic records," it doesn't discuss clinical use cases.	yes	The study appears to work with tabular electronic medical records, which meets this criterion.	maybe	The abstract mentions "electronic records" and "clinical datasets," suggesting human clinical data, though this isn't explicitly stated.	yes	Based on the abstract's structure and content, this appears to be an original research article presenting a new method.	yes	The paper presents a novel algorithm (MIIC-SDG) for generating synthetic data, indicating original creation rather than using pre-existing datasets.	yes	The study clearly involves AI-based synthetic data generation using a multivariate information framework and Bayesian network theory.	no	The abstract doesn't demonstrate direct clinical applications. It focuses on the technical aspects of data generation and quality/privacy metrics.	maybe	The abstract indicates empirical evaluation through comparison with state-of-the-art methods and multiple metrics, though these are technical rather than clinical evaluations.	maybe	The study reports quantifiable outcomes in terms of quality and privacy metrics (QPS), but these are technical rather than clinical outcomes.	Exclude	The study presents a novel synthetic data generation algorithm focused on technical aspects of electronic medical records, with strong methodological contributions in data privacy and quality metrics. While demonstrating original research in AI-driven data generation, the abstract lacks clear demonstration of direct clinical applications or patient-centered outcomes. The work appears more oriented toward technical innovation in synthetic data creation rather than immediate clinical utility.	2.9	Exclude	Exclude	No direct clinical application

Privacy preserving Generative Adversarial Networks to model Electronic Health Records.	Venugopal, Rohit, Shafqat, Noman, Venugopal, Ishwar, Tillbury, Benjamin Mark John, Stafford, Harry Demetrios, Bourazeri, Aikaterini	2022	maybe	The paper focuses on generating synthetic clinical data for machine learning applications, but the primary focus appears to be on the technical aspects of privacy preservation rather than direct clinical applications. While it mentions potential use in NHS, it's more methodological than clinical.	yes	The study clearly deals with tabular data (Electronic Health Records including medical history, diseases, medications). No mention of imaging or other non-tabular formats.	yes	The study uses real NHS patient data as its basis, though the specific nature of the datasets isn't fully detailed in the abstract.	yes	This appears to be an original research article with novel methodology and empirical evaluation.	yes	The study actively creates synthetic data using a privacy-preserving GAN (pGAN), not using pre-existing synthetic datasets.	yes	The study explicitly uses AI methods (GAN) for synthetic data generation.	no	While the paper mentions potential use in NHS, it doesn't demonstrate direct clinical applications. The evaluation focuses on privacy preservation and machine learning performance rather than clinical outcomes.	maybe	The study includes empirical evaluation, but the metrics focus on privacy preservation and machine learning performance rather than clinical utility.	maybe	The study reports quantifiable outcomes, but they're focused on machine learning performance (74.3% accuracy, R2-Score of 0.84) rather than clinical metrics.	Exclude	The study demonstrates technical proficiency in generating synthetic clinical data using privacy-preserving GANs, with strong methodological approaches to data generation and machine learning performance. However, the research lacks direct clinical applications, focusing more on privacy preservation and technical metrics rather than demonstrating clear clinical utility or outcomes. While meeting several technical screening criteria, the paper falls short of providing substantive clinical relevance.	2.9	Exclude	Exclude	No direct clinical application
Privacy-preserving dataset combination and Lasso regression for healthcare predictions.	van Egmond, Marie Beth, Spini, Gabriele, van der Galien, Onno, IJpma, Arne, Veugen, Thijs, Kraaij, Wessel, Sangers, Alex, Rooijakkers, Thomas, Langenkamp, Peter, Kamphorst, Bart, van de L'Isle, Natasja, Kooij-Janic, Milena	2021	maybe	The paper focuses on clinical applications (heart failure prediction) but primarily emphasizes the technical/privacy aspects of secure data sharing. While there is a clinical use case, the main focus appears to be on the secure computation methodology.	maybe	The study appears to work with tabular data (features for heart failure prediction), though this isn't explicitly stated. No mention of imaging or other non-tabular formats.	yes	The study uses real clinical data from Erasmus MC and Achmea regarding heart failure patients.	yes	This appears to be an original research article with implementation and results.	maybe	The study uses synthetic data, but it's unclear if this is actively created using AI methods or if it's just vertically-partitioned data that's already synthetic. The focus seems to be on secure computation rather than synthetic data generation.	maybe	While synthetic data is used, the abstract doesn't specify the generation method. It appears to focus more on secure computation of existing synthetic data rather than the generation process itself.	maybe	The study aims to identify "high-impact lifestyle factors for heart failure," which is a clinical application, though it's more focused on the technical solution.	maybe	The study includes empirical evaluation, but it's primarily focused on computational performance rather than clinical utility.	maybe	The abstract mentions performance metrics but focuses on computational aspects rather than clinical outcomes.	Exclude	The study presents a technical approach to secure data sharing for heart failure prediction, with partial alignment to clinical research criteria. While using human clinical data and demonstrating an original research methodology, the paper's primary focus on secure computation and limited emphasis on synthetic data generation and clinical outcomes suggests marginal suitability for a systematic review of clinical AI applications.	2.9	Exclude	Exclude	No tabular data
Synthesizing electronic health records using improved generative adversarial networks	Baowaly M.K., Lin C.-C., Liu C.-L., Chen K.-T.	2019	no	The paper focuses on generating synthetic EHR data, which is primarily technical/methodological rather than clinical. While it mentions "better services" in the medical industry, it doesn't demonstrate specific clinical applications.	yes	The study clearly focuses on tabular EHR data, which meets this criterion.	yes	The study uses real EHR data from MIMIC-III and NHIRD databases, which are human clinical datasets, meeting this criterion.	yes	This is an original research article with novel methodology and empirical evaluation, meeting this criterion.	yes	The study actively creates synthetic data using AI methods (medWGAN and medBGAN), meeting this criterion.	yes	The study explicitly uses AI methods (GAN variants) for synthetic data generation, clearly meeting this criterion.	no	The abstract doesn't demonstrate direct clinical applications. While it mentions "better services," it focuses on technical performance metrics rather than clinical outcomes.	maybe	The study includes empirical evaluation, but focuses on technical performance rather than clinical utility.	maybe	The study reports quantifiable outcomes, but they are technical metrics (Kolmogorov-Smirnov test, probability measures) rather than clinical metrics.	Exclude	The study demonstrates strong technical capabilities in synthetic electronic health record generation using AI methods, successfully creating tabular data from human clinical sources. However, the research lacks direct clinical applications and focuses primarily on methodological improvements in data generation rather than demonstrating clinical utility or outcomes. While empirically rigorous in its technical approach, the study does not meet criteria for direct clinical relevance or quantifiable clinical metrics.	2.9	Exclude	Exclude	No direct clinical application
Synthetic Behavior Sequence Generation Using Generative Adversarial Networks	Akbari F., Sartipi K., Archer N.	2023	maybe	The paper focuses on behavior sequence generation for monitoring older people's health, which has clinical applications. However, the primary focus appears to be on the technical aspects of the GAN algorithm rather than direct clinical applications.	maybe	The study deals with behavior sequences, which are tabular data. However, these appear to be sequential data rather than purely tabular data, which may not strictly meet the criterion.	maybe	While the data relates to human behavior, it's not clear if this is based on actual clinical data or patient-related healthcare information. The abstract suggests this is more about general behavior monitoring in smart homes.	yes	This appears to be an original research article presenting a new algorithm and its evaluation.	yes	The study actively creates synthetic data using their BehavGAN algorithm, which is a novel GAN implementation.	yes	The study explicitly uses GANs for synthetic data generation.	maybe	The paper mentions health monitoring, but doesn't demonstrate direct clinical applications like diagnosis, prognosis, or treatment planning. The focus is more on behavior prediction and monitoring.	maybe	The study includes empirical evaluation comparing their method against other algorithms (MLE, LeakGAN, SeqGAN). However, the evaluation focuses on technical performance rather than clinical utility.	maybe	The study reports quantifiable outcomes in terms of similarity and diversity metrics for the generated data, but these are not clinical outcomes.	Exclude	The study presents a novel GAN algorithm for generating synthetic behavior sequences, with a focus on technical methodology rather than direct clinical applications. While the research involves synthetic data generation and empirical evaluation, it lacks clear clinical outcomes and patient-related healthcare information. The technical nature of the work and uncertainty about its clinical utility suggest limited alignment with the screening criteria.	2.9	Exclude	Exclude	No tabular data
Synthetic data production for biomedical research	Lee Y.G., Kwak M.-S., Kim J.E., Kim M.S., No D.U., Chai H.Y.	2025	maybe	The abstract mentions applications in "public health and precision medicine" but does not specifically detail clinical applications. The focus appears to be more on the general utility of synthetic data rather than clinical applications.	maybe	The abstract mentions "multi-omics data" which could include non-tabular formats. There is no explicit confirmation that only tabular data is used.	yes	The study uses data from 400 individuals including cancer survivors, chronic disease patients, and healthy participants.	yes	This appears to be an original research article describing a specific project (Healthcare Big Data Showcase Project).	yes	The abstract clearly indicates active creation of synthetic data using AI techniques.	yes	The study explicitly involves generating synthetic data using AI methods.	no	The abstract does not demonstrate direct clinical applications. It focuses on the general utility of synthetic data for research and education.	maybe	While the abstract mentions evaluation of synthetic datasets based on "utility," it does not provide clear metrics for assessing clinical utility.	no	The abstract does not mention specific quantifiable outcomes or metrics for clinical utility.	Exclude	The study involves synthetic data generation using AI techniques with human clinical data, but lacks clear demonstration of direct clinical applications or quantifiable outcomes. While the research explores synthetic data utility across healthcare domains, it appears more methodologically focused than clinically oriented, with uncertain alignment to specific clinical screening criteria.	2.9	Exclude	Exclude	No direct clinical application
True Mitotic Count Prediction in Gastrointestinal Stromal Tumors: Bayesian Network Model and PROMETheus (Preoperative Mitosis Estimator Tool) Application Development	Renne S.L., Cammelli M., Santori I., Tassan-Mangina M., Sama L., Ruspi L., Sicoli F., Colombo P., Terracciano L.M., Quagliuolo V., Cananzi F.C.M.	2024	yes	The study focuses on clinical applications, specifically preoperative risk assessment for GISTs and therapeutic decision-making.	yes	The study appears to work with tabular clinical data (tumor size, location, mitotic count, etc.) rather than imaging or other non-tabular formats.	yes	The study uses real-world GIST cases from IRCCS Humanitas Research Hospital (n=80).	yes	This appears to be an original research article with clear methodology and results.	maybe	While the study mentions using synthetic data sets for validation, it's not clear if they actively created these using AI methods or used pre-existing synthetic datasets.	maybe	The abstract mentions "synthetic data sets" but doesn't specify the generation method. It appears to use Bayesian network methodologies rather than AI methods like GANs or VAEs.	yes	The study demonstrates direct clinical applications in preoperative risk assessment and therapeutic decision-making for GISTs.	yes	The study includes empirical evaluation with clear testing procedures and validation using both synthetic and real data.	yes	The study reports diagnostic performance metrics, though specific numbers aren't provided in the abstract.	Exclude	A clinical study on GIST risk assessment using Bayesian network methodologies demonstrates strong clinical application and empirical evaluation with human clinical data. However, significant uncertainty exists regarding the synthetic data generation process, particularly whether AI methods were employed for data creation. The research appears to focus on computational modeling for preoperative risk stratification rather than AI-driven synthetic data generation.	2.9	Exclude	Exclude	No synthetic data generation
A comparison of confounder selection and adjustment methods for estimating causal effects using large healthcare databases.	Benasseur, Imane, Talbot, Denis, Durand, Madeleine, Holbrook, Anne, Matteau, Alexis, Potter, Brian J, Renoux, Christel, Schnitzer, Mireille E, Tarride, Jean-Eric, Guertin, Jason R	2022	maybe	The paper focuses on clinical applications, specifically confounding adjustment in healthcare data analysis. However, it is more methodological in nature, focusing on statistical methods rather than direct clinical applications.	maybe	The study uses tabular healthcare data, which meets this criterion. However, it is not clear if the synthetic data generation is specifically focused on tabular data.	yes	Yes, the study uses "real large healthcare data" as its basis.	yes	Yes, this is an original research article with a simulation study and empirical evaluation.	maybe	The study does create synthetic data, but it is not clear if AI methods are used for this generation. The focus seems to be on statistical methods (BAC, GBCEE, etc.) rather than AI-based synthetic data generation.	maybe	While synthetic data is used ("Completely synthetic and partially synthetic data"), the abstract does not specify AI methods for generation. The focus is on statistical methods for confounding adjustment.	maybe	The study focuses on methodological aspects (confounding adjustment) rather than direct clinical applications like diagnosis or treatment planning.	yes	Yes, the study includes empirical evaluation through simulation studies with clear metrics.	yes	Yes, the study reports quantifiable outcomes in terms of bias reduction and performance metrics.	Exclude	The study involves healthcare data analysis with synthetic data generation, but lacks clear evidence of AI-driven synthetic data creation. While the research demonstrates methodological rigor in confounding adjustment, it does not fully align with criteria focused on AI-based synthetic data generation and direct clinical applications. The paper primarily represents a statistical methodological approach rather than an AI-driven clinical innovation.	2.8	Exclude	Exclude	No tabular data
InVAErt networks for amortized inference and identifiability analysis of lumped-parameter haemodynamic models.	Tong, Guoxiang Grayson, Sing-Long, Carlos A, Schiavazzi, Daniele E	2025	maybe	The paper focuses on cardiovascular model parameter estimation from EHRs, which has clinical applications. However, it appears to be more methodological in nature, focusing on the technical aspects of parameter estimation.	maybe	The study appears to work with tabular EHR data, though it's not explicitly stated. The focus on "six-compartment lumped-parameter haemodynamic model" suggests numerical data.	yes	The study uses EHR data, which implies human clinical data.	yes	This appears to be an original research article with novel methodology.	maybe	The abstract mentions using "synthetic data" but doesn't clearly indicate if they actively created it using AI. It's unclear if they generated the synthetic data themselves or used existing synthetic datasets.	no	While synthetic data is mentioned, the paper focuses on using inVAErt networks for parameter estimation rather than synthetic data generation. The synthetic data appears to be an input rather than the focus of the study.	maybe	While the study has potential clinical applications in cardiovascular modeling, it appears to be more focused on the technical aspects of parameter estimation rather than direct clinical applications.	maybe	The study includes empirical evaluation using both synthetic and real data, though specific metrics aren't detailed in the abstract.	maybe	The abstract doesn't explicitly mention specific quantifiable outcomes or metrics for evaluation.	Exclude	The study focuses on cardiovascular model parameter estimation using inVAErt networks, with potential clinical implications, but lacks clear evidence of original synthetic data generation using AI methods. While the research involves human clinical data and appears to be an original research article, it primarily addresses technical methodological aspects rather than direct clinical applications or synthetic data creation. The abstract suggests a nuanced approach to physiological modeling that partially meets but does not fully satisfy the comprehensive screening criteria.	2.8	Exclude	Exclude	No synthetic data generation
On the Fidelity-Privacy Tradeoff of Synthetic Cancer Registry Data	Rochner P.	2024	no	The paper focuses on technical aspects (privacy and fidelity of synthetic data) rather than clinical applications. While it uses cancer data, the primary focus is on the technical evaluation of different AI methods for data synthesis.	yes	The study explicitly mentions using "tabular cancer registry data" with "nine categorical variables."	yes	Uses breast cancer patient data from cancer registry, which is human clinical data.	yes	Appears to be original research with empirical evaluation, not a review or commentary.	yes	The study actively creates synthetic data using multiple AI methods (VAEs, GANs, DDPMs).	yes	Clear use of AI methods (VAEs, GANs, DDPMs) for synthetic data generation.	no	The abstract does not demonstrate direct clinical applications. The focus is on privacy and fidelity metrics rather than clinical utility.	maybe	The study includes empirical evaluation, but the metrics focus on technical aspects (privacy and fidelity) rather than clinical utility.	no	While the study reports quantifiable outcomes, they are focused on privacy and fidelity metrics rather than clinical outcomes.	Exclude	The study focuses on technical aspects of synthetic data generation using AI methods for cancer registry data, demonstrating strong methodological rigor in data synthesis. However, the research lacks direct clinical applications and quantifiable clinical outcomes, primarily emphasizing privacy and fidelity metrics rather than practical healthcare utility. While meeting several technical screening criteria, the study falls short of demonstrating clear clinical relevance or impact.	2.8	Exclude	Exclude	No direct clinical application
Parenclitic networks for predicting ovarian cancer	Whitwell H.J., Blyuss O., Menon U., Timms J.F., Zaikin A.	2018	yes	The study focuses on clinical applications, specifically disease classification and diagnosis of ovarian cancer using biomarkers.	yes	The study appears to work with tabular data (proteomic and categorical metadata), though it's not explicitly stated.	yes	The study uses human clinical data from the UKCTOCS trial, including patient samples and controls.	yes	This appears to be an original research article with novel methodology and results.	no	The abstract mentions using a "synthetic data set modelled on data from UKCTOCS," but it's unclear if this was actively created using AI methods or if it's a statistical model. The focus seems to be on the network-based approach rather than synthetic data generation.	maybe	While synthetic data is used, the abstract doesn't specify if AI methods (GANs, VAEs, etc.) were used for generation. The focus is on the network-based analysis rather than the synthetic data generation process.	yes	The study demonstrates direct clinical applications in disease diagnosis and classification of ovarian cancer.	yes	The study includes empirical evaluation with clear metrics for assessing performance.	yes	The study reports specific quantifiable outcomes (sensitivity of 80.3% and 18.9% at different time points, specificity of 98%).	Exclude	The study demonstrates strong clinical application in ovarian cancer diagnosis using network-based approaches with human clinical data, reporting quantifiable outcomes and empirical evaluation. However, critical uncertainties exist regarding the synthetic data generation process, particularly the lack of clear evidence for AI-based methods, which may compromise full alignment with the screening criteria. The research shows promising clinical potential but falls short of definitively meeting all systematic review requirements.	2.8	Include	Exclude	No synthetic data generation
Tabular transformer generative adversarial network for heterogeneous distribution in healthcare.	Kang, Ha Ye Jin, Ko, Minsam, Ryu, Kwang Sun	2025	no	The paper focuses on technical aspects of synthetic data generation rather than clinical applications. While it mentions healthcare applications, the primary focus is on the methodology of generating synthetic tabular data.	yes	The study explicitly focuses on tabular data generation, which meets this criterion.	maybe	While the study mentions healthcare data, it's not explicitly stated whether the data is human clinical data. There's uncertainty here.	yes	Based on the abstract, this appears to be an original research article with novel methodology and experimental results.	yes	The study clearly involves active creation of synthetic data using AI (specifically a TT-GAN), meeting this criterion.	yes	The paper explicitly uses AI methods (TT-GAN, CTGAN, copula GAN) for synthetic tabular data generation, meeting this criterion.	no	The abstract doesn't demonstrate direct clinical applications. It focuses on the technical aspects of data generation rather than specific clinical use cases.	no	While there is empirical evaluation, it's focused on technical performance rather than clinical utility.	no	The abstract mentions "experimental results" and comparisons with other methods, but doesn't specify clinical metrics or outcomes. The evaluation appears to be technical rather than clinical.	Exclude	The study presents a technical methodology for synthetic tabular data generation in healthcare using AI, focusing on data generation techniques rather than direct clinical applications. While demonstrating innovative approaches to handling mixed variable types and generating synthetic data, the research lacks clear clinical outcomes, specific patient-related metrics, and direct demonstration of clinical utility. The work primarily addresses methodological challenges in synthetic data creation, falling short of comprehensive clinical implementation.	2.8	Exclude	Exclude	No direct clinical application

Unlocking the potential of synthetic patients for accelerating clinical trials: Results of the first GIMEMA experience on acute myeloid leukemia patients	Piciocchi A., Cipriani M., Messina M., Marconi G., Arena V., Soddu S., Crea E., Feraco M.V., Ferrante M., La Sala E., Fazi P., Buccisano F., Voso M.T., Martinelli G., Venditti A., Vignetti M.	2024	maybe	The abstract focuses on clinical trials and patient data, which is clinical in nature. However, it appears to be more methodological in its approach, discussing the potential of synthetic patients rather than specific clinical applications.	maybe	The abstract doesn't explicitly specify the data format. While it mentions "patient data," it doesn't clarify if this is tabular data or other formats. This creates significant uncertainty.	yes	The abstract mentions clinical trials and patient data, indicating human clinical data usage.	maybe	The abstract uses language like "this study aims to demonstrate," suggesting original research, but the tone is somewhat theoretical. It's unclear if this is a full research article or a perspective piece.	maybe	The abstract discusses synthetic patient generation using AI, suggesting active creation of synthetic data. However, it's not entirely clear if this is a theoretical discussion or actual implementation.	maybe	While AI is mentioned, the specific methods (GANs, VAEs, etc.) are not detailed. The focus seems to be on the concept rather than specific implementation.	maybe	While clinical trials are mentioned, the abstract doesn't demonstrate specific clinical applications like diagnosis or treatment planning. It remains at a conceptual level.	no	The abstract doesn't describe any empirical evaluation or clear metrics for assessing the synthetic data's utility. It appears more theoretical in nature.	no	The abstract doesn't mention any specific metrics or outcomes. It discusses potential benefits but doesn't indicate measurement of these benefits.	Exclude	The abstract presents a conceptual exploration of synthetic patient generation in clinical trials, demonstrating potential methodological innovations but lacking concrete empirical evidence or quantifiable outcomes. While touching on human clinical data and AI-driven synthetic patient creation, the text remains theoretical and does not provide clear implementation details or specific clinical applications. Significant uncertainties exist regarding data format, specific AI methods, and the depth of original research.	2.8	Include	Exclude	No AI generation
An Improved Statistical Modeling Approach to Individual Anticholinergic Drug Use Trend Analysis	Lou Z., Li M., Kong N., Campbell N.L., Tu W.	2024	yes	The study focuses on clinical applications, specifically predicting anticholinergic drug use patterns in older adults, which has direct clinical implications for pharmacists' decision-making.	yes	The study deals with pharmacy refill data, which is tabular data. There's no mention of imaging or other non-tabular formats.	yes	The study uses real-world pharmacy refill data from human patients.	yes	This appears to be an original research article with novel methodology and empirical evaluation.	maybe	The abstract mentions using "synthetic data" but appears to use it for validation rather than creating new synthetic data. The focus is on statistical modeling of real pharmacy data.	maybe	While synthetic data is used, it's not clear if AI methods (GANs, VAEs) are used for generation. The abstract mentions a "statistical model" rather than AI-based synthetic data generation.	yes	The study has direct clinical applications in predicting drug use patterns to assist pharmacists in making decisions about behavioral interventions.	yes	The study includes both simulation studies and real-world case studies with clear evaluation metrics.	yes	The study reports quantifiable outcomes, specifically mentioning MSE (Mean Squared Error) as a performance metric.	Exclude	The study focuses on clinical applications of predicting anticholinergic drug use in older adults using statistical modeling of pharmacy refill data, demonstrating quantifiable outcomes and empirical evaluation. While the research involves human clinical data and has direct clinical implications, it does not meet key criteria related to AI-based synthetic data generation, which limits its alignment with the systematic review's specific requirements.	2.7	Exclude	Exclude	No synthetic data generation
Attri-VAE: Attribute-based interpretable representations of medical images with variational autoencoders.	Cetin, Irem, Stephens, Maialen, Camara, Oscar, Gonzalez Ballester, Miguel A	2020	maybe	The paper focuses on clinical applications, specifically analyzing healthy and myocardial infarction patients. However, it appears to be primarily focused on the technical aspects of the VAE model rather than direct clinical applications.	no	The study appears to involve both imaging data and clinical attributes, not purely tabular data. The mention of "cardiac morphology" and "radiomics attributes" suggests imaging data is involved.	yes	The study uses human clinical data, specifically analyzing healthy and myocardial infarction patients.	yes	This appears to be an original research article presenting a new VAE approach.	yes	The paper does involve active creation of synthetic data using AI (Attri-VAE), meeting this criterion.	maybe	The study uses VAEs for synthetic data generation, which is an AI method. However, it's not clear if the primary focus is on synthetic data generation or if it's a secondary outcome.	no	While the study involves clinical data, it doesn't clearly demonstrate direct clinical applications like diagnosis or treatment planning. The focus appears to be more on the technical aspects of attribute disentanglement.	maybe	The study includes empirical evaluation, but the metrics focus on technical performance rather than clinical utility.	no	The abstract mentions "several quantitative metrics" but doesn't specify clinical outcomes. The metrics appear to be more technical (reconstruction fidelity, disentanglement).	Exclude	The study involves a technical approach to AI data generation using VAEs, focusing on attribute disentanglement in cardiac imaging data, but lacks clear direct clinical applications or quantifiable clinical outcomes. While the research uses human clinical data and generates synthetic data, it primarily emphasizes methodological innovation over clinical utility, with metrics centered on technical performance rather than patient-centered results. The involvement of imaging data and technical orientation suggests limited alignment with the specified screening criteria.	2.7	Exclude	Exclude	No tabular data
Beyond Henssge's Formula: Using Regression Trees and a Support Vector Machine for Time of Death Estimation in Forensic Medicine	Dani L.M., Toth D., Frigyik A.B., Kozma Z.	2023	maybe	The study focuses on clinical applications (time of death estimation), but it's more of a forensic application rather than a direct clinical care application. This partially meets the criterion.	yes	The study appears to work with tabular data (anthropometric parameters and time measurements), meeting this criterion.	maybe	The study uses human data (anthropometric parameters), but in a forensic context rather than clinical healthcare.	yes	Yes, this appears to be an original research article with novel methodology and results.	maybe	The abstract mentions "synthetic data-driven model" but doesn't clearly specify if they actively created synthetic data using AI. The focus seems to be on using machine learning for prediction rather than synthetic data generation.	maybe	While the study uses synthetic data, it's unclear if they used AI methods to generate it. The focus is on using SVMs and decision trees for prediction, not for generating synthetic data.	maybe	The application is forensic rather than clinical, focusing on time of death estimation rather than diagnosis, treatment, or patient care.	yes	Yes, the study includes empirical evaluation with clear metrics and confidence intervals.	yes	Yes, the study reports clear quantifiable outcomes (error rates, accuracy measurements of ±20 min or ±9.6 min).	Exclude	The study presents a machine learning approach for time of death estimation using synthetic data, but its forensic focus and unclear synthetic data generation methodology raise significant concerns about meeting clinical research screening criteria. While the research demonstrates quantifiable outcomes and empirical evaluation, its primary application in forensic science rather than clinical healthcare limits its alignment with the specified screening requirements.	2.7	Exclude	Exclude	No synthetic data generation
Construction of Genealogical Knowledge Graphs From Obituaries: Multitask Neural Network Extraction System.	He, Kai, Yao, Lixia, Zhang, JiaWei, Li, Yufei, Li, Chen	2021	maybe	The paper focuses on building genealogical knowledge graphs from obituaries for biomedical research, which is more methodological/technical than clinical. While it mentions potential applications in disease heritability and risk prediction, these are not directly demonstrated in the study.	maybe	The study deals with tabular data (genealogical information), but it's primarily focused on extracting and processing text data from obituaries. The final output is a knowledge graph, which is not strictly tabular data.	maybe	The study uses obituary data and EHR data, which are human-related, but the focus is on genealogical relationships rather than clinical healthcare information.	yes	Yes, this is an original research article with clear methodology and results.	maybe	The study does use AI (neural networks) to generate synthetic data through data augmentation, but this is a minor component of the study rather than its primary focus.	maybe	While the study mentions using "data augmentation technology to generate additional synthetic data," it doesn't specify the methods used (GANs, VAEs, etc.) and this appears to be a secondary aspect rather than the main focus.	no	The paper doesn't demonstrate direct clinical applications. It only mentions potential future applications in disease heritability and risk prediction.	maybe	The study includes empirical evaluation, but not of clinical utility - rather of the technical performance of the information extraction system.	maybe	The study reports quantifiable outcomes (precision, recall, F-1 measures), but these are for the information extraction system rather than clinical outcomes.	Exclude	The study primarily focuses on methodological information extraction from obituaries using AI, with limited direct clinical applications and minimal synthetic data generation. While the research involves human-related data and demonstrates technical performance, it lacks clear clinical outcomes or direct healthcare utility. The paper's emphasis on genealogical knowledge graph construction diverges from the core screening criteria for clinical AI research.	2.7	Exclude	Exclude	No direct clinical application
Detecting Smartwatch-Based Behavior Change in Response to a Multi-Domain Brain Health Intervention	Cook D.J., Strickland M., Schmitter-Edgecombe M.	2022	maybe	The study focuses on clinical applications (brain health intervention) rather than purely technical aspects, but it is primarily focused on methodological development of behavior change detection.	no	The study uses sensor data from smartwatches, which is time-series data, and this explicitly fails the criterion requiring only tabular data.	yes	The study uses human clinical data from 28 intervention subjects and 17 control subjects.	yes	This is clearly an original research article with empirical evaluation.	maybe	While the study mentions "synthetic data with known pattern differences," it appears to be using this for validation rather than as the primary focus, and the main data is real sensor data.	no	The abstract doesn't specify the method used to generate synthetic data, and it's not the primary focus of the study. The synthetic data appears to be used only for validation.	maybe	While the study has clinical relevance to brain health interventions, it's more focused on behavior change detection methodology rather than direct clinical applications like diagnosis or treatment planning.	yes	The study includes original research with clear empirical evaluation and metrics.	yes	The study reports clear quantifiable outcomes (slope values and prediction accuracy of 0.87).	Exclude	The study involves time-series sensor data from smartwatches, which disqualifies it from meeting the tabular data requirement. While the research demonstrates clinical relevance and includes empirical evaluation, the primary focus on behavior change detection methodology and limited synthetic data generation prevents full alignment with the screening criteria. The use of non-tabular data and methodological emphasis further reduces its suitability for inclusion.	2.7	Exclude	Exclude	No tabular data
Discrete time forecasting of epidemics	Villela D.A.M.	2020	maybe	The paper focuses on infectious disease forecasting, which is a clinical application. However, it appears to be more methodological in nature, focusing on the mathematical modeling of disease spread rather than direct clinical applications.	no	The study deals with tabular data (counts of cases over time), but it's time-series data which is explicitly excluded in the criteria.	yes	The study uses "severe acute respiratory illness data" which implies human clinical data.	yes	This appears to be an original research article with novel methodology and results.	maybe	The abstract mentions using "synthetic data" but doesn't specify if it was actively created using AI methods. It appears to be more focused on the mathematical modeling approach.	no	While synthetic data is mentioned, there's no indication that AI methods (GANs, VAEs, etc.) were used for generation. The focus seems to be on the phenomenological model rather than synthetic data generation.	maybe	While the study has potential clinical applications in disease forecasting, it appears to be more focused on the mathematical modeling aspect rather than direct clinical applications.	yes	The abstract indicates empirical evaluation using both real and synthetic data, with clear metrics for assessment.	yes	The study reports on forecasting performance and mentions "resilience with statistical noise," suggesting quantifiable outcomes.	Exclude	The study focuses on infectious disease forecasting using a phenomenological model, but fails to meet key screening criteria due to its time-series data format and lack of clear AI-based synthetic data generation. While demonstrating empirical evaluation and using human clinical data, the research appears more methodologically oriented and does not align with the specified requirements for synthetic data generation and clinical application.	2.7	Exclude	Exclude	No tabular data
Dynamic mirroring: unveiling the role of digital twins, artificial intelligence and synthetic data for personalized medicine in laboratory medicine.	Padoan, Andrea, Plebani, Mario	2024	maybe	The paper discusses clinical applications in laboratory medicine and personalized healthcare, but appears to be more of a conceptual overview rather than a specific clinical implementation. It mentions potential applications but doesn't seem to focus on a specific clinical use case.	maybe	The abstract doesn't explicitly mention tabular data generation. While it discusses laboratory parameters and test results, it's unclear if the focus is on synthetic tabular data generation.	yes	The paper discusses human clinical applications and patient data, including laboratory parameters and medical records.	no	This appears to be more of a perspective or review article discussing potential applications rather than presenting original research. The language used ("In this study, we focused on some possible applications") suggests a conceptual exploration.	maybe	The abstract mentions "synthetic data obtained by generative AI" but doesn't clearly indicate whether the study involves active creation of synthetic data or just discusses the concept.	maybe	While generative AI and synthetic data are mentioned, the abstract doesn't specify the methods used (e.g., GANs, VAEs) or provide details about the synthetic data generation process.	maybe	While clinical applications are discussed (laboratory medicine, tumor markers, personalized medicine), the abstract suggests these are potential applications rather than demonstrated implementations.	no	There's no indication of empirical evaluation or clear metrics for assessing synthetic data's clinical utility.	no	The abstract doesn't mention any specific quantifiable outcomes or metrics. It appears to be more theoretical in nature.	Exclude	The abstract presents a conceptual exploration of digital twins and synthetic data in healthcare, focusing on potential applications rather than empirical research. While discussing clinical contexts and generative AI, the text lacks specific details about synthetic data generation methods, quantifiable outcomes, and original research implementation. The paper appears more theoretical, exploring potential future applications of digital twins in laboratory medicine and personalized healthcare.	2.7	Exclude	Exclude	Wrong publication type
Early detection of occupational stress: Enhancing workplace safety with machine learning and large language models	Hasan M.J., Sultana J., Ahmed S., Momen S.	2025	maybe	The paper focuses on occupational stress detection, which is a clinical/healthcare application, but it is more focused on workplace safety and organizational health rather than direct clinical applications.	maybe	The study primarily uses tabular survey data, which meets this criterion. However, it also mentions converting data to text format, which is outside the scope of the criteria.	maybe	The study uses workplace survey data from human subjects, but it's not strictly clinical data in the traditional healthcare sense.	yes	Yes, this is clearly an original research article with novel methodology and results.	no	The abstract mentions "synthetic data generation" but only as part of their validation technique, not as the primary focus of the study.	no	While synthetic data is used, it's only for validation purposes and the method of generation isn't specified. The focus is on ML models for stress detection, not synthetic data generation.	maybe	While the application is health-related, it's more focused on occupational health and safety rather than direct clinical applications like diagnosis or treatment.	yes	The study includes robust empirical evaluation with multiple validation techniques and clear metrics.	yes	Yes, the study reports clear quantifiable outcomes including accuracy metrics (90.32% and 89%).	Exclude	The study focuses on occupational stress detection using machine learning, but falls short of meeting several key screening criteria related to synthetic data generation and direct clinical applications. While the research demonstrates robust empirical evaluation and quantifiable outcomes, its primary emphasis on workplace safety and survey data analysis diverges from the systematic review's specific requirements for synthetic data research.	2.7	Exclude	Exclude	No synthetic data generation
Empirical evaluation of artificial intelligence distillation techniques for ascertaining cancer outcomes from electronic health records	Riaz I.B., Naqvi S.A.A., Ashraf N., Harris G.J., Kehl K.L.	2025	yes	The study focuses on clinical applications, specifically extracting cancer outcomes from EHRs for clinical decision-making.	no	The study primarily deals with unstructured text data (radiology reports) rather than tabular data generation.	yes	The study uses real human clinical data from Dana-Farber Cancer Institute and MIMIC-IV.	yes	This appears to be an original research article with empirical evaluation.	maybe	While the study uses GPT-4 to generate synthetic data, this appears to be a secondary aspect rather than the main focus of the research.	maybe	The study does use AI (GPT-4) for synthetic data generation, but this is not the primary focus and appears to be used as a comparison point rather than the main research objective.	yes	The study demonstrates direct clinical applications in cancer outcome assessment using RECIST criteria.	yes	The study includes clear empirical evaluation with model performance comparisons.	yes	The study reports performance metrics for the models, though specific numbers aren't provided in the abstract.	Exclude	The study focuses on clinical text extraction from electronic health records using deep learning, with a secondary exploration of synthetic data generation. While demonstrating clinical applications in cancer outcome assessment, the research primarily involves unstructured text data rather than tabular data generation, which limits its alignment with the systematic review's core screening criteria. The empirical evaluation and use of human clinical data are strong, but the fundamental data format and research focus deviate from the review's specific requirements.	2.7	Exclude	Exclude	No tabular data

Evaluation of the models generated from clinical features and deep learning-based segmentations: Can thoracic CT on admission help us to predict hospitalized COVID-19 patients who will require intensive care?.	Gulbay, Mutlu, Bastug, Aliye, Ozkan, Erdem, Ozturk, Busra Yuce, Mendi, Bokebatur Ahmet Rasit, Bodur, Hurrem	2022	yes	The study focuses on clinical applications, specifically predicting ICU care for COVID-19 patients using clinical and radiological parameters.	no	The study primarily uses imaging data (CT scans) and radiomics parameters, not tabular data generation. While it includes clinical features, the core focus is on imaging analysis.	yes	The study uses real human clinical data from 191 COVID-19 inpatients.	yes	This is clearly an original research article with methodology, results, and conclusions.	maybe	The study mentions creating synthetic data to balance the training set, but this appears to be a minor component rather than the main focus of the research.	maybe	While synthetic data is used, it's not the primary focus and the method of generation isn't clearly specified as using AI methods like GANs or VAEs.	yes	The study has direct clinical applications in predicting ICU care needs for COVID-19 patients.	yes	The study includes original research with clear empirical evaluation and metrics.	yes	The study reports clear quantifiable outcomes including AUC, specificity, sensitivity, and F1 scores.	Exclude	The study primarily focuses on imaging-based COVID-19 patient prediction using clinical and radiological parameters, which diverges from the systematic review's emphasis on tabular data generation. While the research demonstrates clinical utility and includes some synthetic data elements, it does not meet the core criteria of tabular data generation and AI-driven synthetic data creation. The methodology centers on imaging analysis and machine learning prediction rather than the specified review parameters.	2.7	Exclude	Exclude	No tabular data
EvolveFNN: An Interpretable Framework for Early Detection Using Longitudinal Electronic Health Record Data	Zhang Y., Wittrup E., Hodgman M., Mathis M., Najarian K.	2025	maybe	The paper focuses on clinical applications, specifically cardiac event detection and clinical decision support. However, it appears to be primarily focused on the technical aspects of the model (EvolveFNN) rather than direct clinical applications.	maybe	The study uses longitudinal electronic health records data, which is tabular. However, it's not clear if this is the only data format used. The abstract mentions "high-dimensional" data but doesn't specify the exact format.	yes	The study uses MIMIC-III dataset, which is a real clinical database containing human patient data.	yes	This appears to be an original research article with novel methodology and empirical evaluation.	no	The abstract mentions using "simulated dataset" but doesn't clearly indicate if this was created using AI methods. It appears to be more focused on the analysis of existing data rather than synthetic data generation.	no	While the paper mentions a "simulated dataset," it doesn't appear to focus on synthetic data generation using AI methods. The main focus is on the analysis model (EvolveFNN).	maybe	The paper demonstrates clinical application in cardiac event detection, though this appears to be a secondary focus rather than the primary aim.	yes	The paper includes clear empirical evaluation with multiple experiments and comparisons.	yes	The study reports model performance metrics and compares results with other methods.	Exclude	The study presents a novel AI model for clinical decision support using electronic health records, demonstrating empirical evaluation and quantifiable outcomes with human clinical data. However, the research does not focus on synthetic data generation using AI methods, which represents a critical deviation from the specified screening criteria. While the paper offers valuable insights into cardiac event detection, its primary technical orientation and lack of synthetic data generation render it unsuitable for the systematic review.	2.7	Exclude	Exclude	No synthetic data generation
Exploring Detection Methods for Synthetic Medical Datasets Created with a Large Language Model	Taloni A., Coco G., Pellegrini M., Wjst M., Salgari N., Carnovale-Scalzo G., Scorcia V., Busin M., Giannaccare G.	2025	maybe	The paper focuses on detecting fabrication in synthetic medical datasets rather than clinical applications. While it uses clinical data as a test case, the primary focus is methodological.	yes	The study deals with tabular clinical data, including demographic information and treatment outcomes.	no	While the study uses clinical data as a basis, it's creating fictional clinical studies rather than using real patient data.	yes	This is an original research article with empirical evaluation.	yes	The study actively creates synthetic data using GPT-4 and a custom GPT model.	yes	The study uses AI (specifically GPT-4 and custom GPT) to generate synthetic tabular data.	no	The study does not demonstrate direct clinical applications. Instead, it focuses on detecting fabrication in synthetic datasets.	no	The study includes empirical evaluation, but it's focused on detecting synthetic data fabrication rather than assessing clinical utility.	no	The study reports quantifiable outcomes, but these are related to fabrication detection (e.g., 33.9% fabrication marks, 29.3% reduction) rather than clinical metrics.	Exclude	The study focuses on detecting synthetic data fabrication using AI, primarily examining methodological aspects of data generation rather than direct clinical applications. While the research involves tabular data and original AI-driven synthetic data creation, it lacks real patient data, clinical outcomes, and direct healthcare utility. The paper's primary contribution is in understanding synthetic data generation techniques and potential fabrication detection methods.	2.7	Exclude	Exclude	No direct clinical application
Gait Activity Classification on Unbalanced Data from Inertial Sensors Using Shallow and Deep Learning	Lopez-Nava I.H., Valentin-Coronado L.M., Garcia-Constantino M., Favela J.	2020	maybe	The study focuses on gait activity recognition for health risk identification, which has clinical applications. However, it appears to be more focused on the technical aspects of classification and data augmentation rather than direct clinical applications.	no	The study uses accelerometer and gyroscope data, which is time-series data rather than tabular data.	maybe	The study uses human gait data from a "large-scale public dataset," though it's not explicitly stated whether this is clinical data or general activity data.	yes	This appears to be an original research article with empirical evaluation and results.	yes	The study does involve active creation of synthetic data through augmentation, though the specific AI methods used aren't detailed in the abstract.	maybe	While synthetic data is generated, the abstract doesn't specify whether AI methods (GANs, VAEs, etc.) were used for this purpose. It only mentions "augmented data" based on "segmented gait data."	maybe	While gait activity recognition has potential clinical applications, the abstract doesn't demonstrate direct clinical applications like diagnosis or treatment planning. It focuses on classification performance.	yes	The study includes original research with clear empirical evaluation and metrics for assessing the synthetic data's utility.	yes	The study reports clear quantifiable outcomes in terms of F-measure results (0.812 and 0.927) with standard deviations.	Exclude	The study involves gait activity recognition using time-series data, which deviates from the required tabular data format and limits its clinical applicability. While the research demonstrates synthetic data generation and empirical evaluation, it lacks clear direct clinical applications and does not fully align with the specified screening criteria. The technical focus on classification performance and data augmentation techniques further distances the study from the desired clinical research parameters.	2.7	Exclude	Exclude	No tabular data
Gene regulatory network inference: Evaluation and application to ovarian cancer allows the prioritization of drug targets	Madhamshettiwar P.B., Maetschke S.R., Davis M.J., Reverter A., Ragan M.A.	2012	maybe	The paper focuses on computational methods for gene regulatory network inference, which is primarily technical/methodological rather than clinical. While it has potential clinical applications (drug target identification), the primary focus is on the computational approach.	maybe	The study uses microarray expression data, which is tabular data. However, it's not clear if the synthetic data generation aspect is focused on tabular data specifically.	yes	Yes, the study uses human clinical data (serous papillary ovarian adenocarcinoma expression-microarray data).	yes	Yes, this is an original research article with clear methodology and results.	maybe	The paper uses both simulated datasets and real data, but it's not clear if they actively created synthetic data using AI methods. The focus seems to be on comparing different inference methods rather than generating synthetic data.	maybe	While the paper mentions using "38 simulated datasets," it doesn't specify if these were generated using AI methods. The focus is on network inference methods rather than synthetic data generation.	maybe	The paper has some clinical applications (drug target identification), but these are secondary to the main technical focus on network inference methods.	yes	Yes, the study includes empirical evaluation with clear metrics for assessing the methods' performance.	yes	Yes, the study reports quantifiable outcomes in terms of accuracy of network prediction and drug target identification.	Exclude	The study primarily focuses on computational gene regulatory network inference methods using microarray data, with limited direct clinical application and no clear evidence of AI-driven synthetic data generation. While the research involves human clinical data and includes quantifiable outcomes, it does not meet the core screening criteria related to synthetic data creation and clinical focus. The technical methodology and network inference approach dominate the study's objectives, making it unsuitable for the specified screening requirements.	2.7	Exclude	Exclude	No tabular data
Generation of Individualized Synthetic Data for Augmentation of the Type 1 Diabetes Data Sets Using Deep Learning Models.	Noguer, Josep, Contreras, Ivan, Mujahid, Omer, Beneyto, Aleix, Vehi, Josep	2022	yes	The study focuses on clinical applications, specifically improving prediction models for nocturnal hypoglycemic events in type 1 diabetes patients.	maybe	The study deals with continuous glucose monitor data, which is tabular time-series data. However, this creates some uncertainty as time-series data is explicitly excluded in the criteria.	yes	The study uses data from two cohorts of patients with type 1 diabetes mellitus.	yes	Based on the abstract, this appears to be an original research article with novel methodology and results.	yes	The study actively creates synthetic data using GANs, not using pre-existing synthetic datasets.	yes	The study explicitly uses GANs for synthetic data generation.	yes	The study demonstrates direct clinical application in predicting nocturnal hypoglycemic events.	yes	The study includes empirical evaluation with clear testing of both generative and predictive models.	yes	The study reports on performance improvements of prediction models, though specific metrics aren't detailed in the abstract.	Exclude	A comprehensive study on synthetic data generation for type 1 diabetes using GANs demonstrates strong clinical application and empirical evaluation across multiple patient cohorts. While the research meets most screening criteria with robust methodology and quantifiable outcomes, the use of time-series continuous glucose monitoring data creates a potential disqualification based on the specified data format restrictions. The innovative approach to predicting nocturnal hypoglycemic events shows significant potential in machine learning and clinical data augmentation.	2.7	Exclude	Exclude	No tabular data
Generative adversarial local density-based unsupervised anomaly detection	Li X., Peng J., Li W., Song Z., Du X.	2025	maybe	The paper focuses on anomaly detection with applications in multiple domains including medical diagnostics, but it appears to be primarily a technical/methodological paper about improving GAN-based anomaly detection. While it mentions medical applications, this isn't the primary focus.	yes	The paper deals with tabular data, as evidenced by the mention of "real-world datasets" and the focus on density-based analysis. No mention of imaging or other non-tabular formats.	maybe	While medical diagnostics is mentioned as one application domain, the abstract doesn't specify whether the data used is human clinical data. The evaluation appears to be across multiple domains including industrial monitoring and material analysis.	yes	This appears to be an original research article with novel methodology and empirical evaluation.	maybe	The paper describes creating synthetic data using GANs, but this is primarily for anomaly detection rather than creating synthetic clinical data for clinical use.	maybe	The paper uses GANs for synthetic data generation, but again, this is for anomaly detection purposes rather than creating synthetic clinical data.	maybe	The paper mentions medical diagnostics as an application area, but the focus is on the technical aspects of anomaly detection rather than direct clinical applications.	yes	The study includes extensive empirical evaluation across seven real-world datasets with clear metrics.	yes	The paper reports clear quantifiable outcomes (AUC of 0.874 and accuracy of 94.34%).	Exclude	The study presents a technical methodology for anomaly detection using GANs, with limited direct clinical application and unclear use of human clinical data. While the research demonstrates quantifiable outcomes and empirical evaluation across multiple domains, it primarily focuses on technical aspects of synthetic data generation rather than specific clinical utility. The paper's broad approach and technical orientation suggest it may not fully align with the targeted screening criteria for clinical synthetic data research.	2.7	Exclude	Exclude	No synthetic data generation
Harnessing the flexibility of neural networks to predict dynamic theoretical parameters underlying human choice behavior.	Ger, Yoav, Nachmani, Eliya, Wolf, Lior, Shahar, Nitzan	2024	maybe	The paper focuses on behavioral modeling and psychiatric applications, which are clinical in nature. However, it's primarily a methodological paper about combining RL and neural networks, rather than focusing on clinical applications.	yes	The study appears to work with behavioral choice data in a two-armed bandit task, which is tabular data.	maybe	The study does include human clinical data (psychiatric vs. healthy controls), but this isn't the primary focus of the synthetic data generation.	yes	This is clearly an original research article with novel methodology and empirical evaluation.	no	The paper uses synthetic data generation, but it's not clear if this is AI-generated synthetic data in the traditional sense. The synthetic data appears to be from RL agents rather than AI-generated clinical data.	no	While the paper does involve synthetic data, it's not using AI methods like GANs or VAEs to generate clinical data. Instead, it uses RL agents to generate synthetic behavioral data.	maybe	While the paper has psychiatric applications, it's more focused on behavioral modeling methodology than direct clinical applications.	yes	The paper includes clear empirical evaluation with multiple studies and comparative metrics.	yes	The study reports quantifiable outcomes in terms of prediction performance and RL parameters.	Exclude	The study involves behavioral modeling using reinforcement learning and neural networks, with partial alignment to clinical research criteria. While the research includes human clinical data and quantifiable outcomes, it fails to meet key requirements around AI-generated synthetic clinical data and direct clinical applications. The methodological focus and use of RL agents for synthetic data generation limit its suitability for the specified screening criteria.	2.7	Exclude	Exclude	No synthetic data generation
Hierarchical continuous-time inhomogeneous hidden Markov model for cancer screening with extensive followup data	Meng R., Soper B., Lee H.K.H., Nygard J.F., Nygard M.	2022	maybe	The paper focuses on clinical applications (cervical cancer screening) rather than purely technical aspects, but it has a strong methodological component.	maybe	The study appears to work with tabular screening data, though it's not explicitly stated. The focus on "irregularly sampled" data suggests time-series elements, which may not strictly meet the tabular-only criterion.	yes	The study uses real-world cervical cancer screening data from the Cancer Registry of Norway, which is human clinical data.	yes	This appears to be an original research article presenting a new methodological approach.	no	The abstract mentions using synthetic data for evaluation but doesn't indicate creating new synthetic data. It appears to use existing synthetic data for validation.	no	While synthetic data is used, there's no indication that the study generates synthetic data using AI methods. The focus is on statistical modeling (hidden Markov models).	yes	The study demonstrates direct clinical applications in cancer screening and risk stratification.	yes	The study includes empirical evaluation using both synthetic and real-world data, with comparison to other methods.	maybe	The abstract mentions "superior performance" but doesn't explicitly detail the quantifiable metrics used.	Exclude	The study presents a methodological approach to cancer screening using statistical modeling, but lacks key requirements for synthetic data generation using AI methods. While demonstrating clinical applications and using human clinical data, the research does not create new synthetic data or employ AI-driven data generation techniques. Consequently, the paper does not fully align with the specified screening criteria for synthetic data research.	2.7	Exclude	Exclude	No synthetic data generation
Interpretable instance disease prediction based on causal feature selection and effect analysis.	Chen, YuWen, Zhang, Ju, Qin, XiaoLin	2022	maybe	The paper focuses on clinical applications, specifically disease prediction and causal analysis in healthcare. However, it has a strong methodological component.	maybe	The abstract mentions using "synthetic data, open source data and real medical data" but doesn't specify if it's tabular. Given the focus on feature selection and causal analysis, it's likely tabular, but this isn't explicitly stated.	maybe	The study uses "real medical data" which implies human clinical data, though this isn't explicitly stated.	yes	This appears to be an original research article with novel methodology and experimental results.	no	The abstract doesn't indicate that they create synthetic data using AI. Instead, they appear to use existing synthetic data alongside real data.	no	While synthetic data is used, there's no indication that the study generates synthetic data using AI methods. The focus is on prediction and causal analysis.	yes	The study focuses on disease prediction, which is a direct clinical application.	yes	The study includes empirical evaluation with experiments on multiple data types and reports results.	maybe	The abstract mentions experimental results and quantitative causal explanations, though specific metrics aren't detailed.	Exclude	The study presents a novel machine learning approach for disease prediction using causal analysis, but fails to meet key screening criteria related to synthetic data generation and AI-driven data creation. While demonstrating clinical relevance through disease prediction and using real medical data, the research does not align with the specific requirements of synthetic data generation using AI methods. The methodological focus and lack of explicit synthetic data generation techniques render the paper non-compliant with the primary screening requirements.	2.7	Exclude	Exclude	No synthetic data generation

Large language models to identify social determinants of health in electronic health records	Guevara M., Chen S., Thomas S., Chaunzwa T.L., Franco I., Kann B.H., Moningi S., Qian J.M., Goldstein M., Harper S., Aerts H.J.W.L., Catalano P.J., Savova G.K., Mak R.H., Bitterman D.S.	2024	yes	The study focuses on clinical applications, specifically extracting social determinants of health from EHRs to support research and clinical care.	no	The study primarily deals with narrative text extraction from EHRs, not tabular data generation. While EHRs contain structured data, the focus is on extracting information from unstructured text.	yes	The study uses human clinical data from EHRs.	yes	This appears to be an original research article with empirical evaluation and results.	maybe	The study does use LLMs to generate synthetic data, but this appears to be a secondary aspect rather than the main focus of the research.	maybe	While the study mentions using "LLM-generated synthetic data," it's not clear if this is the primary focus or just an auxiliary component. The main focus appears to be on text extraction.	yes	The study demonstrates direct clinical applications, specifically identifying patients with adverse social determinants of health who could benefit from resource support.	yes	The study includes original research with clear empirical evaluation and metrics.	yes	The study reports clear quantifiable outcomes, including macro-F1 scores and performance metrics.	Exclude	The study focuses on extracting social determinants of health from electronic health records using large language models, demonstrating clinical applications and empirical evaluation. However, the research primarily involves text extraction rather than tabular data generation, which does not align with the core screening criteria. While the study presents valuable insights into clinical data analysis, it falls short of the specific requirements for synthetic tabular data generation.	2.7	Exclude	Exclude	No tabular data
Machine Learning-Driven D-Glucose Prediction Using a Novel Biosensor for Non-Invasive Diabetes Management.	Sadeghi, Pardis, Noroozizadeh, Shahriar, Alshawabkeh, Rania, Sun, Nian Xiang	2025	maybe	The paper focuses on clinical applications (diabetes monitoring) rather than purely technical aspects, but it has a strong technical component with the biosensor integration.	no	The study appears to work with sensor resistance signals, which are time-series data, not tabular data.	maybe	While the application is for human clinical use (diabetes monitoring), it's unclear if the study actually uses human clinical data or just simulated/synthetic data.	yes	This appears to be an original research article based on the methodology and results described.	maybe	The study does use AI for synthetic data generation (GANs and SMOTE), but it's unclear if this is the primary focus or just a methodological tool.	maybe	The study does use AI methods (GANs) for synthetic data generation, but again, this appears to be a secondary aspect rather than the main focus.	yes	The study demonstrates direct clinical application in diabetes monitoring and diagnosis.	maybe	The study includes empirical evaluation using various machine learning models, but the metrics for assessing synthetic data's utility aren't clearly specified.	maybe	The abstract mentions "accurate classification" but doesn't provide specific metrics or outcomes.	Exclude	The study involves diabetes monitoring using machine learning and synthetic data generation, but primarily works with time-series sensor signals rather than tabular data. While demonstrating clinical application potential, the research does not fully align with the systematic review's core screening criteria, particularly regarding data format and synthetic data generation methodology. The abstract suggests a technically innovative approach to diabetes monitoring, but falls short of meeting several key systematic review requirements.	2.7	Exclude	Exclude	No tabular data
Membership inference attacks against synthetic health data.	Zhang, Ziqi, Yan, Chao, Malin, Bradley A	2022	no	The paper focuses primarily on privacy and security aspects of synthetic data, specifically membership inference attacks, rather than clinical applications.	maybe	The abstract mentions "individual-level health data" and discusses synthetic data generation, but does not explicitly specify if it's tabular. However, given the context of membership inference attacks, it's likely tabular data.	yes	The study uses real clinical data from Vanderbilt University Medical Center and the All of Us Research Program.	yes	This appears to be an original research article with novel methodology and experiments.	yes	The study involves active creation of synthetic data using AI methods, as evidenced by discussion of "synthetic data generation" and "synthesis approaches."	yes	The paper clearly involves AI methods for synthetic data generation, mentioning "state-of-the-art machine learning frameworks" and "generative model."	no	The paper focuses on privacy protection and membership inference attacks rather than direct clinical applications like diagnosis or treatment.	no	The study includes empirical evaluation with clear metrics, but these are focused on privacy protection rather than clinical utility.	no	The study reports quantifiable outcomes related to membership inference success rates, but these are not clinical outcomes.	Exclude	The study focuses on synthetic data privacy and membership inference attacks using clinical datasets, demonstrating technical expertise in AI driven data generation. However, the research lacks direct clinical applications, quantifiable clinical outcomes, and empirical evaluation of clinical utility, which limits its alignment with the specified screening criteria. While methodologically rigorous, the paper primarily addresses privacy protection rather than clinical implementation.	2.7	Exclude	Exclude	No direct clinical application
MoCab: A framework for the deployment of machine learning models across health information systems	Kuo Z.-M., Chen K.-F., Tseng Y.-J.	2024	maybe	The paper focuses on a technical framework (MoCab) for integrating ML models into healthcare systems, rather than a specific clinical application. While it has clinical implications, it's primarily a technical/methodological paper.	no	The paper mentions EHR data and synthetic data, but doesn't specifically focus on tabular data generation. The focus is on the framework for handling various data formats.	maybe	The paper discusses EHR systems and healthcare settings, but doesn't explicitly mention using real human clinical data. The demonstration uses synthetic data.	yes	This appears to be an original research article describing a novel framework, not a review or commentary.	no	While synthetic data is mentioned, it's used as a demonstration tool rather than being the focus of the study. The paper doesn't describe creating synthetic data using AI.	no	The abstract doesn't mention any AI methods for generating synthetic data. The synthetic data appears to be used as a testing tool rather than being the subject of study.	maybe	While the paper has clinical implications through CDS Hooks and clinical decision support, it's primarily focused on the technical framework rather than direct clinical applications.	maybe	While there is some demonstration of the framework, the abstract doesn't describe clear empirical evaluation metrics for assessing clinical utility.	no	The abstract doesn't mention specific quantifiable outcomes or metrics for evaluation.	Exclude	The study presents a technical framework for integrating machine learning models into healthcare systems, but lacks direct focus on synthetic data generation, clinical applications, and quantifiable outcomes. While demonstrating potential utility in healthcare settings, the research primarily addresses technical interoperability challenges rather than meeting the specific screening criteria for synthetic data and clinical applications. The paper's methodological approach and use of synthetic data for demonstration purposes do not align closely with the systematic review's core requirements.	2.7	Exclude	Exclude	No tabular data
Overcoming Data Scarcity in Human Activity Recognition.	Konak, Orhan, Liebe, Lucas, Postnov, Kirill, Sauerwald, Franz, Gjoreski, Hristijan, Lustrek, Mitja, Arnrich, Bert	2023	maybe	The study focuses on clinical applications in healthcare through human activity recognition, but it appears to focus significantly on the technical aspects of synthetic data generation and machine learning performance rather than purely clinical applications.	no	The study focuses on sensor data from wearable devices, which is typically time-series data, not tabular data. This is a critical mismatch with the inclusion criteria.	yes	The study uses human activity data and mentions nursing activities, indicating use of human clinical data.	yes	This appears to be an original research article with novel methodology and empirical evaluation.	yes	The study actively creates synthetic data using GANs and 3D engines, not just using pre-existing synthetic datasets.	maybe	While the study does use AI methods (GANs) for synthetic data generation, it's not clear if the final output is tabular data or time-series sensor data.	maybe	While the study has healthcare applications through human activity recognition, the abstract doesn't clearly demonstrate direct clinical applications like diagnosis or treatment planning.	yes	The study includes clear empirical evaluation with specific metrics and comparisons to real-world data.	yes	The study reports quantifiable outcomes in terms of F1-scores and performance improvements.	Exclude	The study involves synthetic data generation for human activity recognition using wearable sensors, but critically fails to meet the tabular data format requirement. While demonstrating original research with quantifiable outcomes and human clinical data, the time-series sensor data fundamentally disqualifies the paper from meeting the core screening criteria. The technical focus and indirect clinical applications further reduce its alignment with the specified review parameters.	2.7	Exclude	Exclude	No tabular data
parSMURF, a high-performance computing tool for the genome-wide detection of pathogenic variants.	Petrini, Alessandro, Mesiti, Marco, Schubach, Max, Frasca, Marco, Danis, Daniel, Re, Matteo, Grossi, Giuliano, Cappelletti, Luca, Castrignano, Tiziana, Robinson, Peter N, Valentini, Giorgio	2020	maybe	The paper focuses on computational biology and genomic medicine, specifically on predicting deleterious variants. While this has clinical implications, the paper appears to focus more on the technical aspects of the method rather than direct clinical applications.	maybe	The study deals with genomic data, which is tabular in nature. However, it's not clear if the synthetic data generation aspect is focused on tabular data specifically.	yes	The study uses human genomic data, including variants associated with Mendelian diseases.	yes	This is clearly an original research article presenting a new method (parSMURF).	maybe	The abstract mentions using synthetic data but doesn't clearly indicate whether they actively created it using AI methods or used pre-existing synthetic datasets.	no	While synthetic data is mentioned, the abstract doesn't specify the use of AI methods (GANs, VAEs, etc.) for generating this data. The focus appears to be on the parSMURF method for handling imbalanced data.	maybe	While the method has potential clinical applications in predicting pathogenic variants, the abstract doesn't demonstrate direct clinical applications. It focuses more on the technical performance of the method.	maybe	The study includes empirical evaluation with clear metrics, but these are primarily focused on computational performance rather than clinical utility.	maybe	The study reports quantifiable outcomes, including an 80-fold speed-up and state-of-the-art results, though these are more focused on computational performance than clinical metrics.	Exclude	The study presents a computational method for genomic data analysis with potential clinical implications, but lacks clear evidence of AI-driven synthetic data generation and direct clinical applications. While the research involves human genomic data and demonstrates computational performance, it primarily focuses on technical aspects of handling imbalanced data rather than meeting the specific screening criteria for clinical AI research.	2.7	Exclude	Exclude	No synthetic data generation
Patient-specific prediction of glioblastoma growth via reduced order modeling and neural networks	Cerrone D., Riccobelli D., Gazzoni S., Vitullo P., Ballarin F., Falco J., Acerbi F., Manzoni A., Zunino P., Ciarletta P.	2025	maybe	The paper focuses on clinical applications (glioblastoma modeling and prediction) rather than purely technical aspects, but it has a strong methodological component.	no	The study primarily deals with imaging data (MRI and DTI) and mathematical modeling, not tabular data generation.	yes	The study uses human clinical data (patient-specific brain anatomies from MRI and DTI).	yes	This appears to be an original research article presenting a novel mathematical model and methodology.	maybe	While the study uses synthetic data, it's unclear if this is AI-generated. The abstract mentions "synthetic data derived from patient-specific brain anatomies" but doesn't specify the generation method.	no	The abstract doesn't explicitly mention using AI methods (GANs, VAEs, etc.) for synthetic data generation. Instead, it focuses on mathematical modeling and neural network surrogates for parameter identification.	yes	The study demonstrates direct clinical applications in neuro-oncology, specifically for tumor growth prediction and patient-specific modeling.	yes	The study includes empirical evaluation through sensitivity analyses and validation of the model's accuracy.	maybe	The abstract mentions "high accuracy" and discusses sensitivity analyses, but specific quantifiable metrics aren't clearly detailed.	Exclude	The study presents a mathematical modeling approach for glioblastoma using imaging data, which diverges from the required focus on tabular data generation. While demonstrating clinical applications in neuro-oncology, the research does not meet key screening criteria related to synthetic data generation methods and data format. Consequently, the paper does not align sufficiently with the systematic review's specific requirements.	2.7	Exclude	Exclude	No tabular data
PM2RA: A Framework for Detecting and Quantifying Relationship Alterations in Microbial Community	Liu Z., Mi K., Xu Z.Z., Zhang Q., Liu X.	2021	maybe	The paper focuses on analyzing microbial relationships in disease contexts, which has clinical applications. However, it appears to be more methodological in nature, focusing on developing an analysis framework (PM2RA) rather than direct clinical applications.	maybe	The study deals with microbial abundance data, which is typically tabular. However, the abstract doesn't explicitly specify the data format.	yes	The study involves human diseases and microbiome data from human subjects.	yes	Yes, this appears to be an original research article presenting a new analysis framework.	maybe	The abstract mentions using synthetic data for evaluation, but it's unclear if they actively created this data using AI methods or used existing synthetic datasets.	no	While synthetic data is mentioned, there's no indication that AI methods (GANs, VAEs, etc.) were used for generation. The focus seems to be on analyzing real data with synthetic data used only for validation.	maybe	While the study has potential clinical implications, it doesn't directly demonstrate clinical applications like diagnosis or treatment planning. It's more focused on understanding microbial relationships.	yes	Yes, the study includes empirical evaluation with both synthetic and real datasets, with clear metrics.	yes	The study reports specificity and sensitivity metrics for their method.	Exclude	The study presents a methodological framework for analyzing microbial relationships in human diseases, using both synthetic and real datasets with quantifiable metrics. While the research involves human clinical data and demonstrates empirical evaluation, it lacks clear AI-based synthetic data generation and direct clinical applications. The paper appears more focused on developing an analytical approach rather than meeting the specific screening criteria for clinical AI research.	2.7	Exclude	Exclude	No synthetic data generation
PROTEIN AI Advisor: A Knowledge-Based Recommendation Framework Using Expert-Validated Meals for Healthy Diets	Stefanidis K., Tsatsou D., Konstantinidis D., Gymnopoulos L., Daras P., Wilson-Barnes S., Hart K., Cornelissen V., Decorte E., Lalama E., Pfeiffer A., Hassapidou M., Pagkalos I., Argiriou A., Rouskas K., Hadjidimitriou S., Charisis V., Dias S.B., Diniz J.A., Telo G., Silva H., Bensenousi A., Dimitropoulos K.	2022	maybe	The paper focuses on personalized nutrition recommendations, which is a clinical application. However, it appears to be more of a lifestyle/health application rather than a direct clinical intervention.	maybe	The study uses tabular data for meal plans and user profiles, but it's unclear if this is the primary focus or if other data formats are involved.	maybe	The study mentions "healthy subjects and participants with health conditions" but it's unclear if real clinical data was used as the basis. The focus seems to be on expert-validated meals rather than clinical data.	yes	This appears to be an original research article presenting a novel framework.	no	The abstract mentions "synthetic data" but it's unclear if this was created using AI methods or other techniques. The system appears to use expert systems and optimization methods rather than AI for data generation.	no	While synthetic data is used (3000 virtual user profiles), there's no indication that AI methods like GANs or VAEs were used for generation. The focus seems to be on the recommendation system rather than synthetic data generation.	maybe	While nutrition planning has health implications, this appears to be more of a lifestyle/health application rather than a direct clinical application like diagnosis or treatment planning.	yes	The study includes empirical evaluation with clear metrics.	yes	The study reports quantifiable outcomes (precision, recall, 92% recommendation accuracy).	Exclude	A personalized nutrition recommendation system using expert-validated meal data demonstrates partial alignment with clinical research criteria, with quantifiable outcomes and empirical evaluation. However, the study lacks clear AI-driven synthetic data generation and direct clinical application, falling short of comprehensive clinical research requirements. The focus appears more oriented toward health and lifestyle recommendations rather than traditional clinical interventions.	2.7	Exclude	Exclude	No synthetic data generation
Reconstructing cerebral hemodynamics from sparse data using Neural Operator Transformers.	Kaczmarek, Wojciech, Magdziarz Ibrahim-El-Nur, Jakub, Los, Magdalena, Roleder, Tomasz	2025	maybe	The paper focuses on clinical applications (cerebrovascular diagnostics and hemodynamics analysis) rather than purely technical aspects, but it is primarily a methodological paper with clinical applications as a potential outcome.	no	The study deals with hemodynamic data (velocity, pressure, cross-sectional area) which appears to be time-series data rather than purely tabular data.	no	The study uses simulated data rather than actual human clinical data, though it aims to be applicable to clinical scenarios.	yes	This appears to be an original research article with novel methodology and empirical evaluation.	maybe	The study uses AI (neural operators) to generate synthetic data, but it's primarily using finite-element simulations as the basis, with AI used for prediction rather than data generation.	no	While the study uses AI methods (neural operators), it's not primarily focused on generating synthetic tabular data. The synthetic data is generated through finite-element simulations, with AI used for prediction.	maybe	The study demonstrates potential clinical applications in cerebrovascular diagnostics, though these are more theoretical than directly implemented.	yes	The study includes original research with clear empirical evaluation and metrics.	yes	The study reports clear quantifiable outcomes (mean relative error of <1% in velocity and area predictions, 3.3% global error).	Exclude	The study presents a computational framework for cerebral hemodynamics using neural operators, but deviates from key screening criteria by utilizing time-series data and finite-element simulations rather than tabular synthetic data generation. While demonstrating potential clinical applications and quantifiable outcomes, the research does not fully align with the specified requirements for synthetic data creation and human clinical data usage.	2.7	Exclude	Exclude	No tabular data

Robust identification of molecular phenotypes using semi-supervised learning.	Roder, Heinrich, Oliveira, Carlos, Net, Lelia, Linstid, Benjamin, Tsypin, Maxim, Roder, Joanna	2019	maybe	The paper focuses on clinical applications, specifically molecular profiling and patient outcomes. However, it appears to be more methodological in nature, focusing on a classification approach rather than direct clinical applications.	maybe	The study deals with molecular profiling data and genomic datasets, which are typically tabular. However, it's not explicitly clear if the data is purely tabular or includes other formats.	yes	The study uses patient samples and clinical outcome information, indicating the use of human clinical data.	yes	This appears to be an original research article presenting a new iterative classification approach.	no	The abstract mentions using synthetic data, but it appears to be using synthetic data as a validation tool rather than actively creating synthetic data as the main focus. The primary focus is on the classification method.	no	While synthetic data is used, there's no indication that AI methods (GANs, VAEs, etc.) are used for generating this synthetic data. The focus is on the classification approach.	maybe	While the study has clinical relevance, it focuses more on the methodological aspects of classification rather than direct clinical applications like diagnosis or treatment planning.	yes	The study includes empirical evaluation using both synthetic and real-world genomic datasets with clear metrics for assessment.	maybe	The study reports on classification accuracy and generalization performance, though specific clinical metrics are not clearly detailed in the abstract.	Exclude	The study presents a methodological approach to molecular profiling and classification, using both synthetic and real-world genomic datasets, but lacks a primary focus on synthetic data generation using AI methods. While demonstrating clinical relevance through patient sample analysis, the research does not meet key screening criteria related to synthetic data creation and direct clinical applications. The paper appears more oriented toward developing a classification technique rather than generating synthetic clinical data.	2.7	Exclude	Exclude	No synthetic data generation
Semi-Supervised PARAFAC2 Decomposition for Computational Phenotyping Using Electronic Health Records.	Konyar, Elif, Gahrooei, Mostafa Reisi	2025	maybe	The paper focuses on computational phenotyping for medical decision-making, which is a clinical application. However, it appears to be primarily a methodological paper developing a new tensor factorization technique, rather than focusing on clinical applications.	maybe	The study works with EHR data, which is tabular. However, it specifically mentions "irregular tensors" which might include time-series data from hospital visits, creating some uncertainty about the data format.	yes	The study uses MIMIC-IV data, which is a real-world clinical database containing human patient data.	yes	This appears to be an original research article presenting a novel method, not a review or commentary.	no	The abstract mentions using a "synthetic data set" but doesn't indicate whether they created this data using AI methods. It appears to be more focused on analyzing existing data rather than generating synthetic data.	no	While synthetic data is mentioned, there's no indication that the study involves generating synthetic data using AI methods. The focus is on analyzing existing data using tensor factorization.	maybe	The paper mentions potential applications in medical decision-making, but the abstract doesn't demonstrate direct clinical applications. It's more focused on the methodological aspects.	no	While there is empirical evaluation, it's focused on the technical performance of the method rather than clinical utility of synthetic data.	no	The abstract mentions "experiments" and "benchmarks" but doesn't specify clear clinical metrics. The outcomes appear to be more methodological than clinical.	Exclude	The study presents a methodological approach to computational phenotyping using tensor factorization, but lacks clear focus on synthetic data generation using AI methods. While utilizing human clinical data and demonstrating potential medical decision-making applications, the research primarily emphasizes technical methodology rather than direct clinical utility or quantifiable outcomes. The paper's core contribution appears to be a novel computational technique for analyzing electronic health records, which diverges from the specific screening criteria related to synthetic data generation.	2.7	Exclude	Exclude	No synthetic data generation
Sex-Specific Classification of Drug-Induced Torsade de Pointes Susceptibility Using Cardiac Simulations and Machine Learning	Fogli Iseppe A., Ni H., Zhu S., Zhang X., Coppini R., Yang P.-C., Srivatsa U., Clancy C.E., Edwards A.G., Morotti S., Grandi E.	2021	yes	The study focuses on clinical applications, specifically cardiac safety assessment and TdP risk prediction in drug development.	no	The study uses simulated cardiac myocyte models and appears to work with time-series data (electrophysiology), not tabular data.	maybe	While the study has clinical relevance, it appears to use simulated cardiac myocyte models rather than direct human clinical data.	yes	This appears to be an original research article with novel findings and methodology.	maybe	While the study generates synthetic data, it's unclear if AI methods are used for this generation. The abstract mentions "simulating drug effects" but doesn't specify the method.	maybe	While synthetic data is used, the abstract doesn't specify if AI methods (GANs, VAEs, etc.) are used for generation. It mentions "statistical learning" but this appears to be for analysis rather than generation.	yes	The study has direct clinical applications in drug safety assessment and TdP risk prediction.	yes	The study includes empirical evaluation with clear metrics for assessing the synthetic data's utility.	yes	The study reports on classifier performance and risk prediction metrics.	Exclude	The study explores cardiac safety assessment using simulated data, but fails to meet key screening criteria due to its use of time-series electrophysiology data instead of tabular data and unclear AI methods for synthetic data generation. While demonstrating clinical relevance and empirical evaluation, the research does not align with the specified requirements for synthetic data generation and data format.	2.7	Exclude	Exclude	No tabular data
SpasticMyoElbow: Physical Human-Robot Interaction Simulation Framework for Modelling Elbow Spasticity.	Yu, Hao, Huang, Zebin, Li, Yutong, Guo, Xinliang, Crocher, Vincent, Carlucho, Ignacio, Erden, Mustafa Suphi	2025	maybe	The paper focuses on clinical applications (spasticity assessment in post-stroke patients) rather than purely technical aspects. However, it's primarily about developing a simulation framework rather than direct clinical application.	no	The study appears to generate synthetic data for robotic measurements and spasticity assessment, but it's unclear if this is tabular data. The focus seems to be on motion and stretch reflex data, which may be time-series data.	maybe	The study is based on post-stroke patients and spasticity assessment, but it's unclear if real patient data was used in the development of the simulation framework. The abstract suggests they're avoiding human subject data.	yes	This appears to be an original research article presenting a new simulation framework and its evaluation.	maybe	The paper describes creating synthetic data through simulation, but it's not clear if AI methods are used. The abstract mentions "simulation framework" and "virtual patients" but doesn't specify AI techniques.	no	While synthetic data is generated, there's no mention of AI methods like GANs or VAEs. The generation appears to be through simulation modeling rather than AI-based methods.	maybe	While the ultimate goal is clinical application (spasticity assessment), the paper focuses on developing a simulation framework rather than demonstrating direct clinical applications.	maybe	The study includes evaluation of different spasticity models, but this is done through simulation rather than clinical validation.	maybe	The study reports on model accuracy in capturing joint resistance characteristics, but these are simulation results rather than clinical outcomes.	Exclude	A simulation framework for spasticity assessment in post-stroke patients was developed, focusing on generating synthetic data through modeling rather than AI techniques. While the study demonstrates potential clinical relevance, it lacks clear evidence of AI-driven synthetic data generation and direct clinical validation. The research primarily explores a simulation approach to understanding spasticity assessment, with limited alignment to the specified screening criteria.	2.7	Exclude	Exclude	No tabular data
Supervised and Unsupervised Approaches for the Real-Time Detection of Undesired Insulin Suspension Caused by Malfunctions	Idi E., Facchinetti A., Sparacino G., Del Favero S.	2024	yes	The study focuses on clinical applications, specifically automated insulin delivery systems and detecting insulin suspension malfunctions in type 1 diabetes treatment.	yes	The study uses tabular data from the UVA/Padova T1D simulator, which generates numerical data about glucose levels and insulin delivery.	maybe	While the study relates to human clinical conditions (T1D), it uses simulated data rather than actual human clinical data.	yes	This appears to be an original research article with clear methodology and results.	maybe	While the study generates synthetic data, it uses a simulator (UVA/Padova T1D simulator) rather than AI methods to create the synthetic data. This is a significant deviation from the criterion.	no	The synthetic data is generated using a computer simulator, not AI methods like GANs or VAEs. This is a critical failure of a core criterion.	yes	The study has direct clinical applications in detecting insulin delivery malfunctions and improving patient safety.	yes	The study includes original research with clear empirical evaluation and metrics.	yes	The study reports clear quantifiable outcomes including recall rates (72%, 82%, 80%) and false positive rates (0.12, 0.21, 0.06 FPs per day).	Exclude	The study focuses on automated insulin delivery systems using simulated data, demonstrating clinical applications and quantifiable outcomes in type 1 diabetes treatment. However, the research fails to meet key screening criteria, particularly the requirement for AI-based synthetic data generation, as it relies on a computer simulator rather than AI methods. Despite strong clinical relevance and empirical evaluation, the methodology does not align with the specified synthetic data generation approach.	2.7	Exclude	Exclude	No synthetic data generation
Synergistic biophysics and machine learning modeling to rapidly predict cardiac growth probability.	Jones, Clara E, Oomen, Pim J A	2025	maybe	The study appears to be primarily focused on the technical modeling framework rather than direct clinical implementation, even though it has clinical applications in mind (cardiac growth prediction).	yes	The study appears to work with tabular data, though this is somewhat implicit from the context of the modeling framework.	maybe	The study uses canine MVR data, not human clinical data. While it mentions potential for patient-specific applications, it doesn't directly use human clinical data.	yes	This appears to be an original research article with novel methodology.	no	The abstract mentions generating synthetic data, but this appears to be for validation purposes rather than the primary focus of creating synthetic clinical data using AI.	no	While synthetic data is used, it's not generated using AI methods like GANs or VAEs. Instead, it's used as a validation tool for their biophysics model.	maybe	While the study aims for clinical applications in cardiac growth prediction, it's primarily a technical modeling study rather than demonstrating direct clinical applications.	yes	The study includes empirical evaluation with clear metrics for assessing model performance.	yes	The study reports quantifiable outcomes in terms of model accuracy and growth predictions within a 95% confidence interval.	Exclude	The study presents a computational modeling framework for cardiac growth prediction using a biophysics approach, but falls short of key screening criteria related to synthetic data generation and human clinical data. While demonstrating technical innovation in cardiac modeling, the research primarily uses animal data and does not employ AI methods for synthetic data creation, limiting its alignment with the specified screening requirements.	2.7	Exclude	Exclude	No synthetic data generation
Synthetic data generation: a privacy-preserving approach to accelerate rare disease research	Mendes J.M., Barbar A., Refaie M.	2025	maybe	The abstract discusses clinical applications in rare disease research, but appears to be more of an overview/review of synthetic data's potential rather than a specific clinical application study.	maybe	The abstract doesn't specify whether the synthetic data is tabular or includes other formats. It mentions "datasets" generally but doesn't provide format details.	yes	The paper clearly focuses on human clinical data, specifically in the context of rare disease research and patient data.	no	The language and structure suggest this is a review article ("This article explores...", "We examine case studies...") rather than original research.	maybe	While synthetic data generation is discussed, the abstract doesn't specify whether the paper involves active creation of new synthetic data or reviews existing approaches.	maybe	The paper discusses synthetic data generation using AI, but it's unclear if this is a review of methods or original implementation.	maybe	While clinical applications are mentioned (diagnosis, treatment, management), the abstract suggests these are discussed theoretically rather than demonstrated directly.	no	There's no indication of original empirical evaluation or clear metrics for assessing synthetic data's utility.	no	The abstract doesn't mention specific quantifiable outcomes or metrics. It appears to be more descriptive of potential benefits.	Exclude	The abstract appears to be a review article exploring synthetic data's potential in rare disease research, lacking original empirical research and quantifiable outcomes. While discussing clinical applications and human clinical data, the paper does not demonstrate direct implementation or provide clear metrics for synthetic data generation. The overview suggests potential benefits but falls short of meeting rigorous research criteria for systematic review inclusion.	2.7	Exclude	Exclude	Wrong publication type
Synthetic Database of Aortic Morphometry and Hemodynamics: Overcoming Medical Imaging Data Availability.	Thamsen, B, Yevtushenko, P, Gundelwein, L, Setio, A A A, Lamecker, H, Kelm, M, Schafstedde, M, Heimann, T, Kuehne, T, Goubergrits, L	2021	yes	The study focuses on clinical applications, specifically modeling hemodynamics for diagnosis and decision-making in coarctation of the aorta.	no	The study primarily deals with imaging data (MRI) and hemodynamic flow data, not tabular data. While some tabular data may be derived from these sources, the primary focus is on imaging and flow data.	yes	The study uses real clinical data from 154 patients with coarctation of the aorta.	yes	This appears to be an original research article with novel methodology and results.	yes	The study actively creates synthetic data using statistical modeling and numerical flow simulations.	maybe	While the study generates synthetic data, it uses statistical modeling and numerical simulations rather than AI methods like GANs or VAEs.	yes	The study demonstrates direct clinical applications in diagnosis and understanding of cardiovascular disease.	yes	The study includes empirical evaluation with clear metrics and comparison with clinical cohorts.	yes	The study reports quantifiable outcomes, including root mean square error of 4.6 mmHg for pressure gradient prediction.	Exclude	The study generates synthetic data for cardiovascular disease using statistical modeling and numerical simulations, demonstrating clinical applications in hemodynamics and patient diagnosis. While the research involves human clinical data and provides quantifiable outcomes, it primarily focuses on imaging and flow data rather than tabular data generation, which deviates from the core screening criteria. The methodology uses statistical techniques instead of AI methods like GANs or VAEs, further limiting its alignment with the specified requirements.	2.7	Exclude	Exclude	No tabular data
Synthetic Data-Enhanced Classification of Prevalent Osteoporotic Fractures Using Dual-Energy X-Ray Absorptiometry-Based Geometric and Material Parameters.	Quagliato, Luca, Seo, Jiin, Hong, Jiheun, Lee, Taeyong, Chung, Yoon-Sok	2025	yes	The study focuses on clinical applications, specifically bone fracture risk assessment for osteoporotic patients, rather than purely technical, privacy, methodological or educational aspects.	no	The study primarily uses 2D-DXA analysis results, which are imaging data, rather than purely tabular data.	yes	The study uses real clinical data from 9,260 patients aged 55-99 from Ajou University Medical Center.	yes	This is clearly an original research article with methodology, results, and conclusions.	yes	The study actively creates synthetic data using the adaptive synthetic oversampler, though this appears to be a statistical method rather than deep learning-based AI.	maybe	While the study does generate synthetic data, it uses an adaptive synthetic oversampler, which is a statistical method rather than the specified AI methods (GANs, VAEs, etc.).	yes	The study demonstrates direct clinical application in bone fracture risk assessment and diagnosis.	yes	The study includes original research with clear empirical evaluation and metrics.	yes	The study reports clear quantifiable outcomes including AUC (0.78 to 0.99) and F1 scores (0.71 to 0.98).	Exclude	The study demonstrates strong clinical relevance in bone fracture risk assessment using patient data, with clear quantifiable outcomes and empirical evaluation. However, the research primarily uses imaging data and employs a statistical synthetic data generation method, which diverges from the specified screening criteria focused on tabular data and AI-driven synthetic data generation. These fundamental differences prevent the study from fully meeting the established screening requirements.	2.7	Exclude	Exclude	No tabular data
Synthetic electronic health records generated with variational graph autoencoders	Nikolentzos G., Vazirgiannis M., Xypolopoulos C., Lingman M., Brandt E.G.	2023	maybe	The paper focuses on generating synthetic health records for clinical use, which appears to be primarily technical/methodological. While it has clinical implications, the abstract emphasizes the technical aspects of data generation and privacy preservation.	no	The study deals with "electronic health records" and "clinical trajectories" represented as "linear-sequence graphs." While these are structured data, the abstract mentions "timelines" and "clinical events over time," suggesting time-series data, which is explicitly excluded in the criteria.	yes	The study uses "real-world electronic health records" as its basis, implying human clinical data.	yes	Based on the abstract's structure and content, this appears to be an original research article presenting a new method.	yes	The study clearly involves active creation of synthetic data using AI (specifically a variational graph autoencoder).	yes	The study explicitly uses AI methods (VGAE) to generate synthetic tabular data.	maybe	While the paper mentions potential clinical applications, it doesn't demonstrate direct clinical applications. The focus is on data generation and privacy preservation rather than specific clinical use cases.	maybe	While the study includes some evaluation ("We show that these artificial patient trajectories are realistic"), the abstract doesn't provide clear metrics or detailed empirical evaluation.	no	The abstract doesn't explicitly mention specific quantifiable outcomes or metrics for evaluating the synthetic data's clinical utility. It only mentions that the artificial trajectories are "realistic."	Exclude	The study presents a technical approach to generating synthetic health records using AI, but fails to meet several key screening criteria, particularly around data format and clinical application. While demonstrating innovative data generation techniques, the research lacks clear quantifiable clinical outcomes and appears to include time-series data, which disqualifies it from the systematic review. The methodological focus and absence of direct clinical utility metrics further compromise its alignment with the specified screening requirements.	2.7	Exclude	Exclude	No tabular data

Synthetic healthcare data utility with biometric pattern recognition using adversarial networks	Manoharan H., Khadidos A.O., Selvarajan S., Singh S.	2025	no	The paper appears to focus primarily on privacy and technical aspects of synthetic data generation rather than clinical applications. While it mentions healthcare and biomedicine, the emphasis is on data privacy and transmission security.	maybe	The abstract doesn't explicitly specify the data format. While it mentions "biometric pattern representations," it's unclear if this refers to tabular data or other formats like images or time-series data.	maybe	While the paper mentions healthcare data, it's unclear whether it uses actual human clinical data as its basis. The focus seems to be on biometric patterns rather than clinical healthcare information.	yes	This appears to be an original research article with empirical evaluation and case studies.	yes	The paper does involve active creation of synthetic data using AI (specifically mentions "deep convolutional adversarial network").	yes	The study uses AI methods (adversarial networks) for synthetic data generation.	no	The abstract doesn't demonstrate direct clinical applications. The focus is on data privacy and transmission security rather than diagnosis, prognosis, or treatment planning.	no	While the paper includes empirical evaluation, it's focused on technical aspects (privacy, transmission) rather than clinical utility.	no	The paper reports some quantifiable outcomes (5% loss rate), but these are related to data generation quality rather than clinical outcomes.	Exclude	The study primarily focuses on technical aspects of synthetic data generation and privacy, lacking direct clinical applications or quantifiable clinical outcomes. While the research involves AI-driven synthetic data creation, it fails to demonstrate clear clinical utility or use of human clinical data. The paper's emphasis on data transmission security and privacy mechanisms suggests it does not meet the core screening criteria for clinical research.	2.7	Exclude	Exclude	No direct clinical application
The National COVID Cohort Collaborative (N3C): Rationale, design, infrastructure, and deployment.	Haendel, Melissa A, Chute, Christopher G, Bennett, Tellen D, Eichmann, David A, Guinney, Justin, Kibbe, Warren A, Payne, Philip R O, Pfaff, Emily R, Robinson, Peter N, Saltz, Joel H, Spratt, Heidi, Suver, Christine, Wilbanks, John, Wilcox, Adam B, Williams, Andrew E, Wu, Chunlei, Blacketer, Clair, Bradford, Robert L, Cimino, James J, Clark, Marshall, Colmenares, Evan W, Francis, Patricia A, Gabriel, Davera, Graves, Alexis, Hemadri, Raju, Hong, Stephanie S, Hripscak, George, Jiao, Dazhi, Klann, Jeffrey G, Kostka, Kristin, Lee, Adam M.	2021	maybe	The paper focuses on creating a collaborative infrastructure for COVID-19 data analysis, which has clinical applications, but the primary focus appears to be on the technical infrastructure and data sharing mechanisms rather than direct clinical applications.	maybe	The paper deals with clinical data that appears to be tabular in nature, though the exact format isn't explicitly stated. The mention of "patient-level data" suggests tabular format.	yes	The study clearly uses human clinical data from COVID-19 patients.	yes	This appears to be an original research article describing the development of the N3C infrastructure.	no	While the paper mentions a "synthetic data pilot," it's not clear if this involves active creation of synthetic data using AI. The focus seems to be on real data sharing and harmonization.	no	The abstract only briefly mentions a "synthetic data pilot" without specifying the methods used. There's no mention of AI methods like GANs or VAEs.	maybe	While the infrastructure is intended for clinical applications, the paper itself doesn't demonstrate direct clinical applications. It focuses on the technical infrastructure.	no	The paper describes the development of infrastructure but doesn't present empirical evaluation of synthetic data's clinical utility.	no	The abstract doesn't mention specific quantifiable outcomes related to clinical utility.	Exclude	The study describes a collaborative infrastructure for COVID-19 data sharing, but lacks clear evidence of AI-driven synthetic data generation or direct clinical applications. While the research involves human clinical data and appears to be an original article, it does not demonstrate quantifiable outcomes or empirical evaluation of synthetic data's clinical utility. The paper primarily focuses on technical infrastructure development rather than synthetic data creation and clinical implementation.	2.7	Exclude	Exclude	No synthetic data generation
The Use of Artificial Neural Networks to Forecast the Behavior of Agent-Based Models of Pathophysiology: An Example Utilizing an Agent-Based Model of Sepsis	Larie D., An G., Cockrell R.C.	2021	maybe	The paper focuses on clinical applications (sepsis prediction and disease progression), but also has significant technical components. It meets this criterion, though not purely clinical.	no	The study uses time-series data (cytokine concentrations over time) rather than purely tabular data. This fails to meet the criterion as time-series data is explicitly excluded.	maybe	The study uses a model of sepsis but doesn't explicitly mention using real human clinical data as its basis. This criterion is unclear from the abstract.	yes	This appears to be an original research article with novel methodology and results. This meets the criterion.	maybe	The study uses agent-based models (IIRABM) to generate synthetic data, but these are not AI-generated synthetic data in the traditional sense. This partially meets the criterion.	no	While the study generates synthetic data, it uses agent-based modeling rather than AI methods like GANs or VAEs. This fails to meet the criterion.	yes	The study demonstrates direct clinical applications in sepsis prediction and disease progression monitoring. This meets the criterion.	yes	The study includes empirical evaluation with clear metrics for assessing the performance of the synthetic data approach. This meets the criterion.	yes	The study reports quantifiable outcomes in terms of prediction accuracy and forecasting performance. This meets the criterion.	Exclude	The study explores synthetic data generation for sepsis prediction using agent-based modeling and neural networks, but deviates from the screening criteria by utilizing time-series data and non-AI synthetic data generation methods. While demonstrating clinical applications and quantifiable outcomes, the research does not align with the specific requirements for AI-driven synthetic tabular data generation. Consequently, the paper falls outside the intended review scope.	2.7	Exclude	Exclude	No tabular data
Uncovering influence links in molecular knowledge networks to streamline personalized medicine.	Shin, Dmitriy, Arthur, Gerald, Popescu, Mihail, Korkin, Dmitry, Shyu, Chi-Ren	2014	maybe	The paper focuses on clinical applications in personalized medicine and therapeutic strategies, particularly in cancer treatment. However, it appears to be primarily a technical/methodological paper about developing the RIIG formalism.	maybe	The paper deals with RDF data and protein/pathway data, which appears to be tabular in nature. However, it's not clear if this is the primary focus or if other data formats are involved.	yes	The study uses human clinical data from cancer cases (ALL, Mesenchymal Chondrosarcoma, Squamous Cell Carcinoma).	yes	This is clearly an original research article with novel methodology and results.	no	The paper mentions using "synthetic data" in simulations, but this appears to be for testing purposes rather than the main focus of creating synthetic clinical data.	no	While synthetic data is mentioned, it's used for testing the RIIG formalism rather than being the focus of AI-based synthetic data generation.	yes	The paper demonstrates direct clinical applications in personalized medicine and therapeutic target selection.	yes	The study includes empirical evaluation with clear metrics and testing on both synthetic and real clinical cases.	yes	The paper reports quantifiable outcomes, including 90% recovery of predefined influence links and concordance with expert-built diagrams.	Exclude	The study presents a novel informatics methodology for analyzing protein pathways in cancer, demonstrating clinical applications with human data and quantifiable outcomes. However, the research does not focus on AI-driven synthetic data generation, instead developing a technical formalism for uncovering biological relationships. While meeting several clinical research criteria, the paper falls short of the specific requirements for synthetic data generation using AI methods.	2.7	Exclude	Exclude	No synthetic data generation
Utilising Machine Learning for Better Mental Health and Decision Making: A Case Study of Timebanking UK	Mohasseb A., Chiroma F., Khusainov R., Curry R.	2025	no	The paper appears to focus on community service operations rather than clinical applications. While it mentions "health systems" briefly, the primary focus seems to be on community-based services and social isolation.	maybe	The abstract mentions synthetic data generation but doesn't specify the format. Given the context of community services and text classification, it's unclear if this is purely tabular data.	no	The study uses community service data rather than clinical or patient healthcare information.	yes	This appears to be an original research article with empirical evaluation.	maybe	The abstract mentions synthetic data generation, but it's unclear if this is active creation using AI or just using existing synthetic datasets.	maybe	While synthetic data is mentioned, the specific methods (GANs, VAEs, etc.) are not detailed. The focus seems more on predictive models and NLP rather than synthetic data generation.	no	The applications described (service matching, text classification, interaction quality) are not direct clinical applications.	maybe	The study includes empirical evaluation, but the metrics for assessing synthetic data's utility are not clearly specified.	maybe	The abstract mentions "effectiveness" but doesn't specify clear quantifiable metrics.	Exclude	The study focuses on community service operations using predictive models and synthetic data generation, but lacks clear clinical healthcare applications and quantifiable clinical outcomes. While involving synthetic data and empirical evaluation, the research does not meet key screening criteria related to clinical data, direct medical applications, and specific healthcare metrics. The abstract suggests a broader social service approach rather than a targeted clinical research study.	2.7	Exclude	Exclude	No direct clinical application
3D human pose data augmentation using Generative Adversarial Networks for robotic-assisted movement quality assessment	Wang X., Mi Y., Zhang X.	2024	maybe	The paper focuses on technical aspects (GANs, SVM, DenseNet) and motion recognition systems rather than direct clinical applications. While it mentions rehabilitation medicine as a potential application, this appears to be a secondary consideration rather than the primary focus.	maybe	The study deals with 3D human pose data, which is not tabular data. It appears to be spatial/time-series data of human movements.	maybe	While the study involves human motion data, it's not clear if this is clinical data or patient-related healthcare information. The focus seems to be on general human motion rather than clinical applications.	yes	Based on the abstract, this appears to be an original research article with novel methodology and experimental results.	yes	The study does involve active creation of synthetic data using AI (specifically GANs).	yes	The study uses AI methods (GANs) for synthetic data generation.	maybe	The paper mentions potential applications in rehabilitation medicine, but doesn't demonstrate direct clinical applications. The focus is on motion recognition and data augmentation.	yes	The abstract indicates original research with empirical evaluation and clear metrics for assessing the synthetic data's performance.	yes	The study reports quantifiable outcomes in terms of classification accuracy and data processing efficiency.	Exclude	The study focuses on technical motion recognition using AI-generated synthetic data, primarily involving 3D human pose data and machine learning techniques. Despite demonstrating innovative data generation methods, the research lacks direct clinical applications and does not use tabular clinical data, which disqualifies it from meeting key screening criteria. The work appears more oriented toward technical methodology and potential future applications rather than immediate clinical utility.	2.5	Exclude	Exclude	No tabular data
A deep learning based framework for the registration of three dimensional multi-modal medical images of the head.	Islam, Kh Tohidul, Wijewickrema, Sudanthi, O'Leary, Stephen	2021	maybe	The paper focuses on image registration for clinical applications (diagnosis, treatment planning, image-guided surgery), but it is primarily a technical methodology paper rather than focusing on clinical applications.	no	The study focuses on imaging data (CT and MRI), not tabular data, which is a clear violation of the criterion.	yes	The study uses human clinical data (CT and MRI images of the head).	yes	This appears to be an original research article presenting a new framework.	yes	The paper does create synthetic data using AI methods, specifically for augmenting existing datasets.	maybe	While the paper does generate synthetic data, it's for image registration rather than tabular data. The methods used aren't explicitly stated but appear to be deep learning-based.	yes	The paper demonstrates direct clinical applications in diagnosis, treatment planning, and image-guided surgery.	yes	The paper includes empirical evaluation on a publicly available registration database.	maybe	While the abstract mentions "accurate and fast" performance, it doesn't explicitly state specific quantifiable metrics.	Exclude	The study focuses on image registration using synthetic data generation, but critically fails to meet the screening criteria due to its use of imaging data instead of tabular data. While demonstrating clinical applications and original research, the fundamental mismatch in data format disqualifies the paper from meeting the specified screening requirements. The technical methodology and imaging focus prevent alignment with the desired research parameters.	2.5	Exclude	Exclude	No tabular data
A deep neural network to distinguish COVID-19 from other chest diseases using X-ray images	Albahli S.	2021	maybe	The paper focuses on clinical applications (COVID-19 detection from chest X-rays), but it's primarily a technical implementation. However, it does have clear clinical relevance.	no	The study focuses on imaging data (X-rays), not tabular data. This is a clear violation of the criterion.	yes	The study uses human clinical data (chest X-rays from patients).	yes	This appears to be an original research article with novel contributions.	maybe	The abstract mentions using GAN-based synthetic data, but it's unclear if they actively created the synthetic data or used existing synthetic datasets.	maybe	While GANs are mentioned, the focus is on using them for data augmentation rather than the primary research question. The main contribution appears to be the deep learning model.	yes	The study has direct clinical applications in COVID-19 diagnosis and distinguishing it from other chest diseases.	yes	The study includes empirical evaluation with clear metrics.	yes	The study reports quantifiable outcomes (87% accuracy).	Exclude	The study focuses on COVID-19 detection using chest X-rays, which primarily involves imaging data rather than tabular data, thus failing a key screening criterion. While the research demonstrates clinical relevance and includes synthetic data generation, its methodological approach centers on deep learning techniques for image classification, making it incompatible with the specified screening requirements. The paper's technical orientation and imaging-based methodology disqualify it from meeting the core screening criteria.	2.5	Exclude	Exclude	No tabular data
A mutual neighbor-based clustering method and its medical applications	Chen J., Zhu X., Liu H.	2022	maybe	The paper focuses on a technical clustering algorithm with some medical applications, but the primary focus appears to be on the technical methodology rather than clinical applications. The medical application seems to be a secondary validation step.	maybe	The abstract mentions "synthetic data" but doesn't specify if it's tabular. Given the clustering context, it's likely tabular, but this isn't explicitly stated.	maybe	While the paper mentions "medical scenarios," it's unclear if it uses actual human clinical data. The focus seems to be on the algorithm with medical applications as a validation step.	yes	Yes, this appears to be an original research article presenting a novel clustering algorithm.	no	The paper doesn't involve creating synthetic data using AI. Instead, it uses existing synthetic data for validation.	no	The paper doesn't generate synthetic data using AI methods. It uses pre-existing synthetic data for testing.	maybe	The paper mentions "disease types" but doesn't clearly demonstrate direct clinical applications. The medical application appears to be more of a validation exercise.	maybe	While there is empirical evaluation, it's focused on the clustering algorithm's performance rather than clinical utility.	maybe	The abstract mentions "comparison experiments" but doesn't specify clear clinical metrics or outcomes.	Exclude	The study presents a technical clustering algorithm with limited clinical application, lacking AI-driven synthetic data generation and clear clinical utility metrics. While the research includes a medical validation component, it primarily focuses on algorithmic methodology rather than direct clinical outcomes or synthetic data creation. The paper does not meet the core screening criteria for clinical AI synthetic data research.	2.5	Exclude	Exclude	No synthetic data generation
A novel approach to brain tumor detection using K-Means++, SGLDM, ResNet50, and synthetic data augmentation	Sarah P., Krishnapriya S., Saladi S., Karuna Y., Bavirisetti D.P.	2024	maybe	The paper focuses on clinical applications (brain tumor detection and classification) rather than purely technical aspects, but it is primarily focused on the technical implementation of the detection system.	no	The study focuses on MRI imaging data, not tabular data, which is a clear violation of the criterion requiring only tabular data generation.	yes	The study uses human clinical data (MRI images of brain tumors).	yes	This appears to be an original research article with novel methodology and results.	no	While the paper mentions "synthetic data augmentation," it appears to be using this as a technique to enhance the existing dataset rather than creating entirely new synthetic data. The focus is on the classification model rather than synthetic data generation.	maybe	The paper uses synthetic data augmentation, but it's not clear if this involves AI methods like GANs or VAEs. The focus is on the classification model (ResNet50) rather than synthetic data generation.	yes	The study demonstrates direct clinical applications in diagnosis and classification of brain tumors.	yes	The study includes empirical evaluation with clear metrics for assessing performance.	yes	The study reports quantifiable outcomes including accuracy, sensitivity, and specificity.	Exclude	The study focuses on brain tumor detection using MRI imaging and deep learning techniques, which fundamentally conflicts with the requirement for tabular data generation. While demonstrating strong clinical applications and empirical evaluation, the research fails to meet critical screening criteria, particularly the specification of tabular data format. The imaging-based approach and synthetic data augmentation technique disqualify the paper from meeting the established screening requirements.	2.5	Exclude	Exclude	No tabular data

A Simulation Study to Compare the Predictive Performance of Survival Neural Networks with Cox Models for Clinical Trial Data.	Kantidakis, Georgios, Biganzoli, Elia, Putter, Hein, Fiocco, Marta	2021	yes	The study focuses on clinical applications, specifically survival prediction in osteosarcoma patients using machine learning models.	yes	The study uses tabular data (5 prognostic factors) and does not involve imaging or other non-tabular formats.	yes	The study is based on human clinical data from the European Osteosarcoma Intergroup study.	yes	This appears to be an original research article with clear methodology and results.	no	The study uses Monte-Carlo simulations to generate synthetic data, but does not use AI methods to create the synthetic data. The synthetic data is generated from statistical distributions.	no	While synthetic data is used, it is generated through statistical methods (log-normal distribution) rather than AI methods like GANs or VAEs.	yes	The study demonstrates direct clinical application in survival prediction and prognosis.	yes	The study includes original research with clear empirical evaluation and metrics.	yes	The study reports clear quantifiable outcomes including C-index, Brier score, and integrated Brier score.	Exclude	The study focuses on clinical survival prediction using machine learning techniques, generating synthetic data through statistical methods rather than AI-driven approaches. While demonstrating strong clinical relevance and quantifiable outcomes, the research fails to meet key screening criteria related to AI-based synthetic data generation. Consequently, the paper does not align with the specific requirements for AI-driven synthetic data research.	2.5	Exclude	Exclude	No synthetic data generation
Acoustic Based Footstep Detection in Pervasive Healthcare	K S., D D., S D., I M., C E., R P.	2021	maybe	The paper focuses on clinical applications (monitoring mobility patterns of older adults) rather than purely technical aspects, but the primary focus appears to be on the technical detection system.	no	The study uses audio data, not tabular data, which is a clear violation of the criterion requiring only tabular data generation.	maybe	The study involves human subjects (older adults) and their mobility patterns, though the data collection is indirect through audio detection.	yes	This appears to be an original research article with novel methodology and evaluation.	maybe	The study mentions using synthetic data, but it's unclear if this is AI-generated or just simulated audio data. The abstract doesn't specify the method of synthetic data creation.	no	While synthetic data is used, it's for audio simulation rather than tabular data generation using AI methods like GANs or VAEs.	yes	The study has direct clinical applications in monitoring mobility patterns for older adults' care and autonomy.	yes	The study includes empirical evaluation with clear metrics and comparison with existing models.	yes	The study reports quantifiable outcomes using Polyphonic Sound Detection Scores (PSDS).	Exclude	The study involves audio-based footstep detection for older adult mobility monitoring, but critically fails to meet key screening criteria by using non-tabular data and unclear synthetic data generation methods. While demonstrating clinical relevance and empirical evaluation, the research fundamentally diverges from the required focus on tabular data and AI-driven synthetic data generation. Consequently, the study does not align with the specified screening requirements.	2.5	Exclude	Exclude	No tabular data
AI algorithm for personalized resource allocation and treatment of hemorrhage casualties	Jin X., Frock A., Nagaraja S., Wallqvist A., Reifman J.	2024	yes	The study focuses on clinical applications, specifically hemorrhage patient management and fluid resuscitation in mass casualty scenarios, rather than purely technical, privacy, methodological or educational aspects.	no	The study uses vital signs data, which appears to be time-series data, rather than purely tabular data.	maybe	The abstract does not explicitly state whether the computational model is based on human clinical data, creating some uncertainty.	yes	The study appears to be an original research article with novel methodology and results.	yes	The study uses a "cardio-respiratory computational model to generate synthetic data," indicating active creation of synthetic data.	yes	The study uses a "deep neural network-based AI model" and generates synthetic data, meeting the criterion for synthetic data generation using AI methods.	yes	The study demonstrates direct clinical applications in hemorrhage detection and treatment planning.	yes	The study includes empirical evaluation with clear metrics for assessing the synthetic data's clinical utility.	yes	The study reports specific quantifiable outcomes, such as 46% additional casualties restored and 119% increase in fluid-utilization efficiency.	Exclude	A deep neural network-based AI study exploring synthetic data generation for hemorrhage patient management demonstrates strong clinical applications and quantifiable outcomes. However, the use of time-series vital signs data instead of purely tabular data represents a critical deviation from the specified screening criteria. Uncertainty remains regarding the basis of the computational model's data source.	2.5	Exclude	Exclude	No tabular data
AI and semantic ontology for personalized activity eCoaching in healthy lifestyle recommendations: a meta-heuristic approach	Chatterjee A., Pahari N., Prinz A., Riegler M.	2023	maybe	The paper focuses on clinical applications (health monitoring and personalized recommendations for lifestyle), but it's more focused on the technical aspects of AI prediction and ontology integration. While there is a clinical component, it's not the primary focus.	no	The study primarily deals with time-series data (activity levels, step counts) rather than tabular data. This is a clear violation of the inclusion criteria.	yes	Yes, the study uses human clinical data from healthy adults (n=16 for public datasets; n=15 for private datasets).	yes	Yes, this is an original research article with clear methodology and results.	maybe	The study does use ADASYN to generate synthetic data, but this is only for handling imbalanced classes, not as the main focus of the research.	maybe	While synthetic data is used (ADASYN), it's not the primary focus and is used only for handling class imbalance. The main focus is on time-series prediction and ontology integration.	maybe	The clinical application is somewhat indirect - it's focused on lifestyle coaching rather than direct clinical diagnosis or treatment.	yes	Yes, the study includes clear empirical evaluation with specific metrics and comparisons.	yes	Yes, the study reports quantifiable outcomes (accuracy scores for classification, performance metrics).	Exclude	The study primarily focuses on time-series data and AI-driven lifestyle coaching, which deviates from the required tabular data format and clinical application criteria. While the research involves human clinical data and demonstrates quantifiable outcomes, its methodological approach and data type do not align with the systematic review's core screening requirements. Consequently, the paper does not meet the essential inclusion criteria for the review.	2.5	Exclude	Exclude	No tabular data
AI Based Monitoring of Different Risk Levels in COVID-19 Context	Melo C., Dixe S., Fonseca J.C., Moreira A.H.J., Borges J.	2021	maybe	The paper focuses on technical aspects (mask detection and temperature measurement) rather than clinical applications. While these could be used in healthcare settings, the primary focus appears to be on surveillance and monitoring rather than clinical decision-making.	no	The study uses image data (RGB and thermal cameras) rather than tabular data.	maybe	While the study involves human subjects (for mask detection and temperature measurement), it's not using clinical healthcare data in the traditional sense.	yes	This appears to be an original research article with novel implementation and evaluation.	yes	The study does involve active creation of synthetic data, though the exact methods aren't specified in detail.	maybe	While synthetic data generation is used, it's for image data rather than tabular data, and the specific AI methods aren't clearly stated.	maybe	The applications described (mask detection and temperature measurement) are more public health/surveillance focused rather than direct clinical applications.	yes	The study includes empirical evaluation with clear metrics and performance measurements.	yes	The study reports clear quantifiable outcomes (precision metrics for detection tasks).	Exclude	The study involves synthetic data generation for mask and temperature detection using image data, which fundamentally differs from the required tabular data format. While demonstrating technical innovation in surveillance applications, the research fails to meet critical screening criteria related to clinical data, application focus, and data type. Consequently, the paper does not align with the systematic review's core requirements.	2.5	Exclude	Exclude	No tabular data
An efficient hybrid computer-aided breast cancer diagnosis system with wavelet packet transform and synthetically-generated contrast-enhanced spectral mammography images	Amin M.N., Kamal R., Farouk A., Gomaa M., Rushdi M.A., Mahmoud A.M.	2023	yes	The study focuses on clinical applications (breast cancer diagnosis) rather than purely technical aspects.	no	The study deals with imaging data (CESM and LE images) rather than tabular data generation.	yes	The study uses data from 160 female subjects with breast lesions.	yes	This appears to be an original research article with novel findings and methodology.	yes	The study involves active creation of synthetic data (SynCESM images) using computational methods.	maybe	While the study does generate synthetic data, it's not clear if AI methods (GANs, VAEs) are used. The abstract mentions "computational methods" but doesn't specify the technique.	yes	The study demonstrates direct clinical application in breast cancer diagnosis and lesion classification.	yes	The study includes original research with clear empirical evaluation and metrics.	yes	The study reports clear quantifiable outcomes including accuracy, sensitivity, specificity, and AUC metrics.	Exclude	The study focuses on breast cancer diagnosis using imaging data, which fails to meet the tabular data generation requirement. While demonstrating strong clinical application and empirical evaluation, the research involves image-based synthetic data generation rather than the specified tabular format. Consequently, the paper does not align with the core screening criteria despite its methodological rigor.	2.5	Exclude	Exclude	No tabular data
An online chronic diseases consulting system: A hyper heuristic algorithm using random and greedy strategy for complex scheduling problems	Tingxi W., Huirong W., Ming-Fa H., Lingwei X., Daoyuan W., Weizhen L., Huailin D.	2016	maybe	The paper focuses on a clinical consulting system, but primarily from a technical/algorithmic perspective rather than clinical applications. The main focus is on scheduling optimization algorithms.	maybe	The study uses tabular data for scheduling, but it's unclear if this is the primary focus or just a means to an end. The data appears to be primarily about scheduling rather than clinical data.	maybe	The study uses real clinical data from a hospital, though it's primarily scheduling data rather than patient health information.	yes	This is an original research article with clear methodology and results.	no	The study uses synthetic data sets, but these are generated for algorithmic testing rather than using AI methods for synthetic data generation. The synthetic data is created for testing the scheduling algorithm.	no	While synthetic data is used, it's not generated using AI methods (GANs, VAEs, etc.). Instead, it appears to be generated for algorithmic testing purposes.	maybe	While the system has clinical implications (consulting system), the focus is on the algorithmic solution rather than direct clinical applications like diagnosis or treatment.	maybe	The study includes empirical evaluation, but again, this is focused on algorithmic performance rather than clinical utility.	maybe	The study reports quantifiable outcomes, but these are focused on algorithmic performance (efficiency, cost savings) rather than clinical metrics.	Exclude	The study primarily focuses on algorithmic optimization for a clinical consulting system, using synthetic data for testing, but does not meet key criteria related to AI-generated synthetic data or direct clinical applications. While the research involves clinical data and demonstrates empirical evaluation, its technical approach and operational focus diverge from the systematic review's core requirements for synthetic data generation and clinical utility.	2.5	Exclude	Exclude	No synthetic data generation
Analysis of epidemiological association patterns of serum thyrotropin by combining random forests and Bayesian networks	Becker A.-K., Ittermann T., Dorr M., Felix S.B., Nauck M., Teumer A., Volker U., Volzke H., Kaderali L., Nath N.	2022	maybe	The paper focuses on analyzing TSH concentrations and thyroid functionality, which is a clinical application. However, it's primarily focused on the methodological aspects of combining random forests with Bayesian networks, rather than direct clinical applications.	yes	The study appears to work with tabular data (serum TSH concentrations and various health variables).	yes	The study uses real human clinical data from the Study of Health in Pomerania (SHIP).	yes	This is an original research article presenting a new machine learning workflow.	no	The abstract mentions using synthetic data for evaluation, but this appears to be for testing their methodology rather than creating synthetic clinical data. The main analysis is on real data from SHIP.	no	While synthetic data is used, it's not for generating clinical data but rather for testing their machine learning workflow. The synthetic data generation method is not specified.	maybe	While the study has clinical relevance (analyzing TSH concentrations), it's more focused on demonstrating a methodological approach rather than direct clinical applications.	maybe	The study includes empirical evaluation, but it's focused on the machine learning methodology rather than evaluating synthetic data's clinical utility.	maybe	The study reports quantifiable metrics (RMSE, MAE, R^2) but these are for the machine learning model's performance rather than clinical outcomes.	Exclude	The study primarily focuses on a machine learning methodology for analyzing TSH concentrations, using real clinical data from a population study. While demonstrating technical sophistication in combining random forests and Bayesian networks, the research does not generate synthetic clinical data or directly address synthetic data generation. The paper's methodological approach and use of real clinical data diverge from the core screening criteria related to synthetic data creation.	2.5	Exclude	Exclude	No synthetic data generation
Application of Feedforward and Recurrent Neural Networks for Fusion of Data from Radar and Depth Sensors Applied for Healthcare-Oriented Characterisation of Persons' Gait.	Mazurek, Pawel	2023	maybe	The paper focuses on healthcare monitoring of elderly persons, which is a clinical application. However, it appears to be more focused on the technical aspects of sensor fusion and neural networks rather than direct clinical applications.	no	The study uses sensor data (radar and depth sensors) which appears to be time-series data, not tabular data.	maybe	The study involves monitoring elderly persons, so it does involve human subjects. However, it's unclear if real clinical data was used as the basis for the synthetic data.	yes	This appears to be an original research article with empirical evaluation, not a review or commentary.	maybe	The abstract mentions "synthetic data" but doesn't specify if it was actively created using AI or if pre-existing synthetic datasets were used. There's insufficient information to determine this.	no	While synthetic data is mentioned, there's no indication that AI methods (GANs, VAEs, etc.) were used for generation. The focus is on using neural networks for data fusion, not data generation.	maybe	While the study has healthcare applications, it focuses more on technical aspects of sensor fusion rather than direct clinical applications like diagnosis or treatment planning.	yes	The abstract mentions numerical experiments and clear metrics for evaluation.	yes	The study reports quantifiable outcomes related to position estimation and walking parameters.	Exclude	The study involves healthcare monitoring of elderly persons using sensor fusion techniques, but fails to meet key screening criteria related to tabular data generation and AI-driven synthetic data creation. While demonstrating quantifiable outcomes and empirical evaluation, the research primarily focuses on technical sensor data processing rather than direct clinical applications or synthetic data generation using AI methods.	2.5	Exclude	Exclude	No tabular data
Application of TimeGAN to IMU-based Data of Upper Limb Range of Motion	Bhagat N., Sanghavi V., Kapila V.	2024	maybe	The paper focuses on clinical applications, specifically rehabilitation and disability simulation. However, it's primarily focused on the technical aspects of generating synthetic data rather than direct clinical applications.	no	The study focuses on time-series data from IMU sensors, which is explicitly excluded in the criteria as it's not tabular data.	yes	The study uses human subject data for range of motion measurements.	yes	This appears to be an original research article with empirical evaluation.	yes	The study does involve active creation of synthetic data using TimeGAN, an AI method.	yes	The study uses TimeGAN, a GAN-based method, to generate synthetic data.	maybe	While the paper mentions potential clinical applications (classifying impairments, guiding therapy interventions), these are future possibilities rather than demonstrated applications in the study.	maybe	The study includes empirical evaluation with clear metrics (PCA, t-SNE, discriminative scores), but these evaluate the synthetic data generation process rather than clinical utility.	maybe	The study reports quantifiable outcomes (discriminative scores) but these are related to data generation quality rather than clinical outcomes.	Exclude	The study focuses on generating synthetic time-series data for rehabilitation using TimeGAN, which fails the tabular data format requirement. While demonstrating technical capabilities in synthetic data generation, the research primarily explores methodological aspects rather than direct clinical applications, with potential future clinical uses remaining speculative. The use of time-series data from IMU sensors and lack of demonstrated clinical outcomes further limits its alignment with the specified screening criteria.	2.5	Exclude	Exclude	No tabular data
Artificial neural networks enable genome-scale simulations of intracellular signaling	Nilsson A., Peters J.M., Meimetis N., Bryson B., Lauffenburger D.A.	2024	no	The paper focuses on cellular signaling and transcription factor activity, which is primarily a biological/technical application rather than a clinical one. While it has potential clinical implications, the abstract doesn't explicitly discuss clinical applications.	maybe	The study appears to work with transcriptomics data, which is typically tabular. However, it's not clear if this is the primary focus or if other data formats are used.	no	The study uses macrophage cells and transcriptomics data, but it's not clear if this is human clinical data. The focus appears to be on cellular biology rather than clinical patient data.	yes	This is clearly an original research article with novel methodology and empirical results.	no	The study uses a recurrent neural network framework, but it's not clear if this is used for synthetic data generation or just for prediction. The mention of "synthetic data" appears to be for testing rather than generation.	no	While the study mentions synthetic data, it appears to be using it for testing rather than generating it. The focus is on prediction and simulation rather than synthetic data generation.	no	The abstract doesn't demonstrate direct clinical applications. While the research could have clinical implications, it focuses on cellular signaling mechanisms.	yes	The study includes original research with clear empirical evaluation and metrics.	yes	The study reports clear quantifiable outcomes (Pearson correlation coefficients of 0.98 and 0.8 for different predictions).	Exclude	The research focuses on cellular signaling mechanisms using a neural network framework, but lacks direct clinical applications and does not generate synthetic clinical data. While the study demonstrates empirical evaluation with quantifiable outcomes, it fails to meet key screening criteria related to clinical focus, human patient data, and synthetic data generation. The work appears more aligned with basic biological research than clinical data science applications.	2.5	Exclude	Exclude	No synthetic data generation

Artificial neural networks trained on simulated multispectral data for real-time imaging of skin microcirculatory blood oxygen saturation.	Larsson, Marcus, Ewerlof, Maria, Salerud, E Goran, Stromberg, Tomas, Fredriksson, Ingemar	2022	maybe	The paper focuses on clinical applications (imaging blood oxygen saturation in skin for studying ischemic tissue), but it's primarily a technical implementation of AI for image analysis. While it has clinical relevance, the focus appears to be on the technical solution rather than clinical applications.	no	The study deals with imaging data and multispectral data, not tabular data.	maybe	The study includes in-vivo examples and focuses on human tissue, though the primary data is synthetic.	yes	This appears to be an original research article with novel methodology and results.	maybe	The study does create synthetic data using AI (ANNs trained on synthetic data from Monte Carlo simulations), but this is for training purposes rather than generating clinical data.	no	While the study does generate synthetic data, it's for training AI models on imaging data, not for generating synthetic tabular clinical data.	maybe	While the technology has clinical applications (studying ischemic tissue), the paper focuses more on the technical implementation than demonstrating direct clinical applications.	yes	The study includes empirical evaluation with clear metrics comparing ANN performance to inverse MC.	yes	The study reports quantifiable outcomes (mean absolute deviation of 1.3%-units, analysis time of 0.11s).	Exclude	The study primarily focuses on technical AI implementation for imaging blood oxygen saturation, using synthetic data for training neural networks, but fails to meet key screening criteria related to tabular data generation and direct clinical applications. While demonstrating quantifiable outcomes and empirical evaluation, the research is more methodologically oriented and does not align with the specified requirements for synthetic clinical data generation.	2.5	Exclude	Exclude	No tabular data
Automated cooling tower detection through deep learning for Legionnaires' disease outbreak investigations: a model development and validation study.	Wong, Karen K, Segura, Thaddeus, Mein, Gunnar, Lu, Jia, Hannapel, Elizabeth J, Kunz, Jasen M, Ritter, Troy, Smith, Jessica C, Todeschini, Alberto, Nugen, Fred, Edens, Chris	2024	maybe	The study focuses on clinical/public health applications (Legionnaires' disease outbreak investigation) rather than purely technical aspects. However, the primary focus is on image detection rather than direct clinical applications.	no	The study uses aerial imagery data, not tabular data. This clearly fails this criterion as it deals with image data rather than tabular data generation.	no	While the study relates to human disease, it doesn't directly use human clinical data or patient information. Instead, it focuses on environmental features (cooling towers).	yes	This is clearly an original research article with empirical evaluation and results.	maybe	While the study mentions "synthetic data" augmentation, it's unclear if this refers to AI-generated synthetic data or other forms of data augmentation. The focus is on image detection rather than synthetic data creation.	no	The abstract doesn't specify the method of synthetic data generation, and it's not the primary focus of the study. The main AI component is image detection, not synthetic data generation.	maybe	The study has indirect clinical applications through public health surveillance, but it's not focused on direct clinical applications like diagnosis or treatment.	yes	The study includes thorough empirical evaluation with clear metrics and testing across multiple cities.	yes	The study reports clear quantifiable outcomes (sensitivity, PPV, search speed comparisons).	Exclude	The study primarily focuses on image detection for public health surveillance using aerial imagery, which diverges from the systematic review's core criteria of synthetic tabular clinical data generation. While demonstrating empirical research with quantifiable outcomes, the paper fails key screening requirements by not involving tabular data, human clinical data, or direct synthetic data generation. The research represents a high-quality public health investigation but does not align with the specific systematic review parameters.	2.5	Exclude	Exclude	No tabular data
Automatic Optimization of Multichannel Electrode Configurations for Robust Fetal Heart Rate Detection by Blind Source Separation	Galli A., Peri E., Rabotti C., Ouzounov S., Mischi M.	2023	maybe	The paper focuses on clinical applications (fetal heart rate monitoring), but it's primarily a technical/methodological paper about electrode configuration and signal processing. While it has clinical implications, the focus is on the technical aspects of signal processing.	no	The study deals with time-series data (ECG signals), not tabular data. This is a clear violation of the inclusion criteria.	yes	The study uses real and synthetic fetal heart rate data, which is human clinical data.	yes	This appears to be an original research article with novel methodology and results.	maybe	While the study mentions using synthetic data, it's not clear if they actively created it using AI methods. The focus seems to be on signal processing rather than synthetic data generation.	maybe	The abstract mentions using synthetic data but doesn't specify the generation method. There's no mention of AI methods like GANs or VAEs.	yes	The study has direct clinical applications in fetal monitoring and medical intervention.	yes	The study includes empirical evaluation with clear metrics and comparison of different configurations.	yes	The study reports clear quantifiable outcomes (80.9% accuracy, 92.2% recognition rate).	Exclude	The study focuses on fetal heart rate monitoring using time-series ECG data, which fundamentally conflicts with the requirement for tabular data generation. While demonstrating clinical relevance and empirical evaluation, the research primarily addresses technical signal processing aspects rather than AI-driven synthetic data creation. Consequently, the paper fails to meet several key screening criteria, particularly those related to data format and synthetic data generation methodology.	2.5	Exclude	Exclude	No tabular data
Basis and perspectives of artificial intelligence in radiation therapy	Burgun A.	2019	maybe	The paper discusses clinical applications in radiation oncology, including image classification, EHR analysis, and adaptive radiation therapy. However, it appears to be more of an overview/review of AI applications rather than focusing on a specific clinical application.	no	While the paper mentions EHR data, it also discusses image classification and other formats. It's not exclusively focused on tabular data generation.	yes	The paper discusses applications in radiation oncology and EHRs, which implies human clinical data, but it's not explicitly stated.	no	This appears to be a review or perspective paper rather than original research, given its broad overview nature and lack of specific methodology or results.	maybe	The abstract mentions that GANs "have been tested to generate synthetic data" but doesn't indicate if this is original research or a review of others' work.	maybe	While GANs for synthetic data generation are mentioned, it's only briefly referenced as one of many AI applications discussed. The focus isn't specifically on synthetic data generation.	maybe	While clinical applications are mentioned (radiation therapy, EHR analysis), they're discussed in general terms rather than specific implementations.	no	No empirical evaluation or metrics are described in the abstract.	no	The abstract doesn't mention any specific quantifiable outcomes or metrics.	Exclude	The abstract presents a broad overview of artificial intelligence in radiation oncology, discussing various AI techniques and applications, but lacks the specific focus on synthetic data generation and empirical research required by the screening criteria. While touching on clinical applications and data formats, the text appears to be a review or perspective piece rather than an original research article with quantifiable outcomes. Consequently, the abstract does not comprehensively meet the systematic review's detailed screening requirements.	2.5	Exclude	Exclude	Wrong publication type
Becalm: Intelligent Monitoring of Respiratory Patients	Recio-Garcia J.A., Diaz-Agudo B., Acuaviva A.	2023	yes	The study focuses on clinical applications, specifically remote monitoring of respiratory support therapies for COVID-19 patients, rather than purely technical, privacy, methodological or educational aspects.	no	The study involves time-series data from sensors, which is explicitly excluded by the criteria.	yes	The study uses real patient data for verification and focuses on human clinical applications.	yes	This appears to be an original research article with empirical evaluation and results.	yes	The study uses a synthetic data generator to simulate patients' clinical evolution, indicating active creation of synthetic data.	maybe	While synthetic data is generated, the abstract doesn't specify the AI methods used (GANs, VAEs, etc.). It mentions "synthetic data generator" but the methodology is unclear.	yes	The study demonstrates direct clinical applications in monitoring respiratory patients and detecting risk situations.	yes	The study includes empirical evaluation with clear metrics and validation against real data.	yes	The study reports quantifiable outcomes, specifically mentioning accuracy of 0.91 for the proposed solution.	Exclude	A clinical monitoring system for respiratory patients demonstrates promising synthetic data generation and empirical validation, with high accuracy in detecting patient risk situations. However, the study's use of time-series sensor data disqualifies it from meeting the specified screening criteria, particularly the requirement for tabular data generation. Despite strong clinical applications and quantifiable outcomes, the methodological approach prevents full compliance with the screening requirements.	2.5	Exclude	Exclude	No tabular data
Bias Detection in Histology Images Using Explainable AI and Image Darkness Assessment.	Skarga-Bandurova, Inna, Sharifnia, Golshid, Biloborodova, Tetiana	2025	maybe	The paper focuses on clinical applications (cervical histology image classification) rather than purely technical aspects. However, it's primarily focused on bias mitigation and explainability, which are methodological aspects.	no	The study deals with image data (cervical histology images), not tabular data.	yes	The study uses cervical histology images, which are human clinical data.	yes	This appears to be an original research article with empirical evaluation and novel framework development.	maybe	While the study mentions "synthetic data augmentation," it's unclear if this involves active creation of synthetic data using AI. The focus seems to be on brightness normalization and existing data augmentation techniques.	maybe	The abstract doesn't explicitly mention using AI methods for synthetic data generation. The "synthetic data augmentation" mentioned could be traditional methods.	yes	The study demonstrates direct clinical application in image classification for cervical histology, which has diagnostic implications.	yes	The study includes original research with clear empirical evaluation using multiple metrics and statistical analysis.	yes	The study reports quantifiable outcomes including accuracy metrics and F-statistic values.	Exclude	The study focuses on cervical histology image classification with a methodological approach to bias mitigation, but critically fails to meet the tabular data generation requirement. While demonstrating clinical relevance and empirical evaluation, the research primarily involves image data processing and explainability techniques, which diverges from the specified screening criteria. Consequently, the paper does not align sufficiently with the predefined screening requirements for inclusion.	2.5	Exclude	Exclude	No tabular data
Bridging the simulation-to-real gap for AI-based needle and target detection in robot-assisted ultrasound-guided interventions.	Arapi, Visar, Hardt-Stremayr, Alexander, Weiss, Stephan, Steinbrener, Jan	2023	maybe	The paper focuses on clinical applications (ultrasound-guided interventional radiology) rather than purely technical aspects, though it has a strong technical component in developing the synthetic data generation method.	no	The study focuses on ultrasound imaging data, not tabular data, which is a clear violation of the criterion.	maybe	The study uses real clinical preoperative 3D data as its basis, though the validation is done on in vitro data.	yes	This appears to be an original research article with novel methodology and empirical evaluation.	yes	The study does involve active creation of synthetic data using AI, specifically generating synthetic ultrasound data from real clinical 3D data.	maybe	While the study does involve synthetic data generation using AI methods, it's specifically for imaging data rather than tabular data.	yes	The study demonstrates direct clinical applications in interventional radiology, specifically for needle tip and target anatomy detection.	yes	The study includes empirical evaluation with clear metrics for assessing the synthetic data's utility in clinical applications.	yes	The abstract mentions accuracy and frame rate as metrics, though specific numbers aren't provided.	Exclude	The study involves synthetic data generation for ultrasound imaging in interventional radiology, but critically fails to meet the tabular data requirement. While demonstrating strong clinical application and empirical evaluation, the focus on imaging data rather than tabular data disqualifies it from meeting the core screening criteria. The research shows promise in AI-driven medical interventions but does not align with the specified data format constraints.	2.5	Exclude	Exclude	No tabular data
CI-GNN: A Granger causality-inspired graph neural network for interpretable brain network-based psychiatric diagnosis	Zheng K., Yu S., Chen B.	2024	maybe	The paper focuses on clinical applications (psychiatric diagnosis) but also has a strong technical focus on GNN explainability. While it has clinical relevance, the primary focus appears to be on the technical methodology.	no	The study uses graph data (brain networks) rather than tabular data, which fails to meet the criterion for tabular data generation.	yes	Yes, the study uses human clinical data (brain disease datasets and psychiatric patients).	yes	Yes, this appears to be an original research article with novel methodology and empirical evaluation.	maybe	While the paper mentions using synthetic data, it appears to be using this for evaluation rather than actively creating synthetic data as its primary focus.	no	The paper uses synthetic data but not for the purpose of generating tabular data using AI methods. Instead, it uses GNNs for analysis.	yes	Yes, the study demonstrates direct clinical applications in psychiatric diagnosis.	yes	Yes, the study includes original research with empirical evaluation and clear metrics.	yes	Yes, the study reports quantifiable outcomes in terms of performance metrics and clinical evidence.	Exclude	The study focuses on graph neural networks for psychiatric diagnosis, using brain network data rather than tabular data, which disqualifies it from meeting key screening criteria. While demonstrating clinical relevance and empirical evaluation, the research primarily emphasizes technical methodology of GNN explainability, falling short of the systematic review's core requirements for synthetic data generation and tabular data formats.	2.5	Exclude	Exclude	No tabular data
Combining machine learning and dynamic system techniques to early detection of respiratory outbreaks in routinely collected primary healthcare records.	Borges, Derick G F, Coutinho, Elua R, Cerqueira-Silva, Thiago, Grave, Malu, Vasconcelos, Adriano O, Landau, Luiz, Coutinho, Alvaro L G A, Ramos, Pablo Ivan P, Barral-Netto, Manoel, Pinho, Suani T R, Barreto, Marcos E, Andrade, Roberto F S	2025	maybe	The paper focuses on clinical applications (outbreak detection and surveillance) rather than purely technical aspects, but it is more focused on epidemiological surveillance than direct clinical applications.	no	The study uses time-series data from healthcare encounters, which is explicitly mentioned as an exclusion criterion in the screening criteria.	yes	The study uses real-world healthcare encounter data from Brazilian health regions, which constitutes human clinical data.	yes	This appears to be an original research article with clear methodology and results.	maybe	While the study uses synthetic data, it's not clear if they actively created it using AI or used pre-existing synthetic datasets. The abstract mentions "synthetic labeled data" but doesn't specify the generation method.	maybe	The abstract doesn't specify the method used for synthetic data generation. While they use synthetic data, there's no mention of AI methods like GANs or VAEs.	maybe	While the study has public health applications, it's focused on outbreak detection rather than direct clinical applications like diagnosis or treatment planning.	yes	The study includes original research with clear empirical evaluation and metrics for assessing performance.	yes	The study reports clear quantifiable outcomes including probability of detection (86%), positive predictive value (85%), and reliability (79%).	Exclude	The study focuses on epidemiological surveillance using time-series healthcare encounter data, which violates the specified data format screening criterion. While demonstrating strong methodological rigor and quantifiable outcomes, the research does not align with the required clinical application and data generation specifications. Consequently, the paper fails to meet critical screening requirements for inclusion.	2.5	Exclude	Exclude	No tabular data
COVID-19 Hierarchical Classification Using a Deep Learning Multi-Modal	Althenayan A.S., AlSalamah S.A., Aly S., Nouh T., Mahboub B., Salameh L., Alkubeyyer M., Mirza A.	2024	yes	The study focuses on clinical applications, specifically COVID-19 diagnosis and pneumonia classification using medical imaging and tabular data.	maybe	The study primarily focuses on chest X-ray (CXR) images, which are not tabular data. While it mentions "related tabular medical data," the primary focus appears to be on imaging data.	yes	The study uses data from 4523 patients, clearly indicating human clinical data.	yes	This appears to be an original research article with novel methodology and results.	yes	The study uses GANs to generate synthetic data, indicating active creation of synthetic data using AI.	yes	The study explicitly mentions using "various versions of generative adversarial networks (GANs) to generate synthetic data."	yes	The study demonstrates direct clinical application in diagnosis and classification of COVID-19 and other types of pneumonia.	yes	The study includes clear empirical evaluation with specific metrics and testing on a large dataset.	yes	The study reports specific quantifiable outcomes (macro-avg F1-score of 95.9% and F1-score of 87.5% for COVID-19 identification).	Exclude	The study demonstrates strong clinical application in COVID-19 diagnosis using AI techniques, with comprehensive empirical evaluation and quantifiable outcomes across a large patient dataset. However, the primary focus on imaging data rather than tabular data generation represents a significant deviation from the specified screening criteria. Despite meeting most requirements, the imaging-centric approach prevents full alignment with the desired research parameters.	2.5	Exclude	Exclude	No tabular data

Data-driven generation of 4D velocity profiles in the aneurysmal ascending aorta	Saitta S., Maga L., Armour C., Votta E., O'Regan D.P., Salmasi M.Y., Athanasiou T., Weinsaft J.W., Xu X.Y., Pirola S., Redaelli A.	2023	maybe	The paper focuses on generating synthetic blood flow data for computational fluid dynamics (CFD) models, which is primarily a technical/methodological application rather than a direct clinical application. While it has clinical relevance, it's not directly focused on clinical applications.	no	The study deals with 4D flow MRI data and velocity profiles, which are time-series data. This explicitly violates the criterion requiring only tabular data.	yes	The study uses data from 30 subjects with ATAA, which is human clinical data.	yes	This is clearly an original research article with novel methodology and results.	maybe	The study uses PCA and statistical shape modeling to generate synthetic data, which is a form of AI/ML technique. However, it's not using modern AI methods like GANs or VAEs.	maybe	While the study does generate synthetic data, it uses statistical modeling (PCA) rather than the specified AI methods (GANs, VAEs).	no	While the data could be used for clinical applications, the paper itself focuses on generating synthetic data for CFD models rather than demonstrating direct clinical applications.	yes	The study includes empirical evaluation comparing synthetic and clinical datasets with clear metrics.	yes	The study reports quantifiable outcomes in terms of flow morphology descriptors and statistical correlations.	Exclude	The study generates synthetic blood flow data using statistical modeling, but fails to meet key screening criteria due to its time-series data format and primarily technical focus. While the research involves human clinical data and includes empirical evaluation, it does not demonstrate direct clinical applications or use specified AI methods for synthetic data generation. The methodology and outcomes, though rigorous, do not align with the predefined screening requirements.	2.5	Exclude	Exclude	No tabular data
DDT-Net: Dose-Agnostic Dual-Task Transfer Network for Simultaneous Low-Dose CT Denoising and Simulation	Meng M., Wang Y., Zhu M., Tao X., Mao Z., Liao J., Bian Z., Zeng D., Ma J.	2024	maybe	The paper focuses on CT imaging reconstruction and denoising, which is a technical application. While it has clinical implications, the primary focus appears to be on the technical methodology rather than direct clinical applications.	no	The study deals with imaging data (CT scans), not tabular data. This clearly fails to meet the criterion for tabular data generation.	yes	The study mentions using clinical data, though it's primarily focused on CT imaging data.	yes	This appears to be an original research article with novel methodology and experimental results.	maybe	The study does involve active creation of synthetic data using AI (DDT-Net), but for imaging rather than tabular data.	no	While the study does generate synthetic data using AI methods, it's specifically for imaging data, not tabular data.	maybe	While the technology has clinical applications in CT imaging, the paper focuses more on the technical aspects of image reconstruction rather than direct clinical applications like diagnosis or treatment planning.	yes	The paper includes empirical evaluation with clear metrics for assessing the performance of the synthetic data generation method.	yes	The study reports quantifiable outcomes in terms of denoising and generalization performance.	Exclude	The study focuses on CT imaging reconstruction using deep learning, primarily addressing technical methodology rather than direct clinical applications. While the research involves synthetic data generation and clinical data, it fundamentally deals with imaging data instead of tabular data, which disqualifies it from meeting the core screening requirements. The technical nature and imaging focus prevent the study from satisfying the key criteria for inclusion.	2.5	Exclude	Exclude	No tabular data
Deep feature-domain matching for cardiac-related component separation from a chest electrical impedance tomography image series: proof-of-concept study.	Zhang, Ke, Li, Maokun, Liang, Haiqing, Wang, Juan, Yang, Fan, Xu, Shenheng, Abubakar, Aria	2022	maybe	The paper focuses on clinical applications (cardiac-related signal separation in EIT measurements for pulmonary perfusion monitoring), but it also has a strong technical/methodological component.	no	The study deals with EIT measurements and imaging data, not tabular data, which is a clear violation of the criterion.	yes	The study uses data from three patients with pulmonary embolism, so it does involve human clinical data.	yes	This appears to be an original research article with novel methodology and empirical evaluation.	maybe	While the study uses synthetic data, it's not clear if they actively created it using AI or used pre-existing synthetic datasets. The abstract mentions "synthetic data" but doesn't specify the generation method.	no	The study uses synthetic data but doesn't explicitly mention using AI methods like GANs or VAEs for generation. The focus is on signal separation rather than data generation.	yes	The study demonstrates direct clinical applications in cardiac-related signal separation for pulmonary perfusion monitoring.	yes	The study includes empirical evaluation using both synthetic and real patient data, with some metrics for assessment.	maybe	The abstract mentions "satisfactory separation accuracy" but doesn't provide specific metrics. It also mentions qualitative consistency checks.	Exclude	The study involves cardiac signal separation using EIT measurements, but critically fails to meet key screening criteria, particularly the requirement for tabular data generation. While demonstrating clinical relevance and empirical evaluation, the research focuses on imaging and signal processing techniques rather than synthetic tabular data creation, rendering it unsuitable for the systematic review.	2.5	Exclude	Exclude	No tabular data
Deep learning assisted cancer disease prediction from gene expression data using WT-GAN.	Ravindran, U, Gunavathi, C	2024	yes	The study focuses on clinical applications, specifically cancer diagnosis and prediction using synthetic data generation.	maybe	The study uses gene expression microarray data, which is typically high-dimensional time-series data, not simple tabular data.	yes	The study uses human cancer gene expression data.	yes	Based on the abstract, this appears to be an original research article with novel implementation and results.	yes	The study actively creates synthetic data using WT-GAN, a type of GAN.	yes	The study explicitly uses AI methods (WT-GAN) for synthetic data generation.	yes	The study demonstrates direct clinical application in cancer diagnosis.	yes	The study includes empirical evaluation with clear metrics for assessing the synthetic data's utility.	yes	The study reports quantifiable outcomes (>97% classification accuracy).	Exclude	The study demonstrates strong alignment with most screening criteria, including clinical application, synthetic data generation, and empirical evaluation in cancer diagnosis. However, the use of gene expression microarray data, which is considered high-dimensional time-series data, represents a critical deviation from the specified tabular data format requirement. This fundamental mismatch with the data format criterion significantly reduces the study's potential for inclusion.	2.5	Include	Exclude	No tabular data
Deep learning significantly boosts CRT response prediction using synthetic longitudinal strain data: Training on synthetic data and testing on real patients	Chang Y.-F., Yen K.-C., Wang C.-L., Chen S.-Y., Chen J., Chu P.-H., Lai C.-S.	2025	yes	The study focuses on a clinical application, specifically predicting cardiac resynchronization therapy (CRT) response in heart failure patients, rather than purely technical, privacy, methodological or educational aspects.	maybe	The study uses two-dimensional echocardiographic strain traces, which are time-series data, rather than only tabular data generation.	yes	The study uses data from 131 human patients with heart failure.	yes	The study appears to be an original research article with clear methodology and results.	maybe	The study uses SMOTE (Synthetic Minority Over-sampling Technique) to generate synthetic data, which is a statistical oversampling technique rather than an AI-based synthetic data generation method.	maybe	The study uses SMOTE to generate synthetic data, rather than AI methods like GANs or VAEs as specified in the criteria.	yes	The study has a direct clinical application in predicting CRT response for heart failure patients.	yes	The study includes original research with clear empirical evaluation and metrics.	yes	The study reports clear quantifiable outcomes including accuracy, precision, sensitivity, F1 score, specificity, and AUROC.	Exclude	The study focuses on predicting cardiac resynchronization therapy response using echocardiographic data, but fails to meet key screening criteria due to its use of time-series data and statistical oversampling technique instead of AI-based synthetic data generation. Despite demonstrating strong clinical relevance and quantifiable outcomes, the methodological approach does not align with the specified research parameters.	2.5	Exclude	Exclude	No tabular data
Deep nets vs expert designed features in medical physics: An IMRT QA case study.	Interian, Yannet, Rideout, Vincent, Kearney, Vasant P, Gennatas, Efstathios, Morin, Olivier, Cheung, Joey, Solberg, Timothy, Valdes, Gilmer	2018	maybe	The study focuses on clinical applications (IMRT QA) rather than purely technical aspects, but it is primarily focused on technical performance comparison rather than direct clinical decision-making.	no	The study uses fluence maps and gamma passing rates, which are not strictly tabular data. The data appears to be image-based (2D diode array measurements).	maybe	The study uses IMRT plans from clinical settings, but it's unclear if these are from actual patient treatments or simulated cases.	yes	Yes, this appears to be an original research article with clear methodology and results.	no	While the study creates synthetic data, it's through simple transformations (rotating and translating) rather than AI-based generation.	no	The synthetic data creation method described is not AI-based but rather traditional data augmentation techniques.	maybe	While the study has clinical relevance (IMRT QA), it's focused on technical validation rather than direct clinical decision-making.	yes	Yes, the study includes original research with clear empirical evaluation and metrics.	yes	Yes, the study reports clear quantifiable outcomes (mean absolute error of 0.70 ± 0.05).	Exclude	The study involves technical validation of neural networks for radiation therapy quality assurance, using image based data and traditional data augmentation techniques rather than AI-generated synthetic tabular data. While demonstrating quantifiable outcomes and empirical research, the work does not meet key screening criteria related to synthetic data generation, data format, and direct clinical application. The research is more focused on technical performance comparison than clinical decision-making.	2.5	Exclude	Exclude	No tabular data
Deep reinforcement learning for automated radiation adaptation in lung cancer:	Tseng H.-H., Luo Y., Cui S., Chien J.-T., Ten Haken R.K., Naqa I.E.	2017	yes	The study focuses on clinical applications in radiation therapy for NSCLC patients, specifically for dose adaptation protocols.	maybe	The study primarily deals with imaging data (PET signals) and dosimetric variables, not purely tabular data generation.	yes	The study uses data from 114 NSCLC patients who received radiotherapy.	yes	This appears to be an original research article with detailed methodology and results.	yes	The study uses a GAN to create synthetic data from real patient characteristics.	yes	The study explicitly uses GANs for synthetic data generation, though this is not the primary focus of the paper.	yes	The study demonstrates direct clinical application in radiation therapy dose adaptation for cancer treatment.	yes	The study includes empirical evaluation with clear metrics comparing DRL-generated doses with clinical decisions.	yes	The study reports quantifiable outcomes including RMSE values for dose adaptation and comparison with clinical decisions.	Exclude	The study demonstrates a sophisticated approach to radiation therapy dose adaptation using deep reinforcement learning and synthetic data generation, with strong clinical applications in NSCLC patient treatment. While the research meets most screening criteria, it fails to qualify due to its primary focus on imaging and dosimetric data rather than purely tabular data generation. The comprehensive methodology and empirical evaluation showcase a promising approach to personalized cancer treatment protocols.	2.5	Include	Exclude	No tabular data
Delineation of intracavitary electrograms for the automatic quantification of decrement-evoked potentials in the coronary sinus with deep-learning techniques	Jimenez-Perez G., Acosta J., Bocanegra-Perez A.J., Arana-Rueda E., Frutos-Lopez M., Sanchez-Brotons J.A., Llamas-Lopez H., Di Massa Pezzutti R., Gonzalez de la Portilla Concha C., Camara O., Pedrote A.	2024	maybe	The paper focuses on clinical applications (cardiac arrhythmia diagnosis and treatment) rather than purely technical aspects, but it has a strong technical component in developing the AI algorithm.	no	The study focuses on EGM (electrocardiogram) signals, which are time-series data, and this explicitly fails the criterion requiring only tabular data.	yes	The study uses real human clinical data from 77 patients who underwent AP or AVRT ablation.	yes	This is clearly an original research article with novel methodology and empirical evaluation.	maybe	The study does use AI for synthetic data generation ("synthetic data augmentation method was developed"), but this appears to be a secondary aspect rather than the main focus.	maybe	While synthetic data generation is mentioned, it's used as an augmentation technique rather than the primary focus of the study. The main focus is on developing a diagnostic algorithm.	yes	The study demonstrates direct clinical applications in cardiac arrhythmia diagnosis and treatment planning.	yes	The study includes thorough empirical evaluation with clear metrics and clinical validation.	yes	The study reports clear quantifiable outcomes including precision, recall, sensitivity, and specificity metrics.	Exclude	The study focuses on cardiac arrhythmia diagnosis using deep learning techniques, but fails to meet the critical requirement of using only tabular data due to its reliance on time-series EGM signals. While demonstrating strong clinical applications and empirical evaluation, the non-tabular data format and secondary use of synthetic data generation disqualify it from meeting the primary screening criteria.	2.5	Exclude	Exclude	No tabular data
Design and technical validation to generate a synthetic 12-lead electrocardiogram dataset to promote artificial intelligence research	Yoo H., Moon J., Kim J.-H., Joo H.J.	2023	maybe	The paper focuses on clinical applications (ECG diagnosis) rather than purely technical aspects, but it is primarily focused on data generation methodology.	no	The study focuses on ECG signals, which are time-series data, not tabular data, which is a clear violation of the inclusion criteria.	yes	The study uses real ECG data as its basis, which is human clinical data.	yes	This appears to be an original research article with novel methodology and results.	yes	The study actively creates synthetic data using AI (CNN and Bi-LSTM), meeting this criterion.	yes	The study uses AI methods (deep learning) to generate synthetic data, meeting this criterion.	maybe	While the study demonstrates ECG classification, it's primarily focused on the data generation process rather than direct clinical applications.	yes	The study includes original research with clear empirical evaluation metrics.	yes	The study reports clear quantifiable outcomes (RMSE, cosine similarity, classification accuracy).	Exclude	The study generates synthetic ECG data using AI methods, demonstrating high classification accuracy and addressing data privacy challenges. However, the research focuses on time-series ECG signals rather than tabular data, which critically disqualifies it from meeting the primary screening criteria. Despite strong methodological rigor and empirical validation, the fundamental data format requirement prevents inclusion.	2.5	Exclude	Exclude	No tabular data
Detection of overdose and underdose prescriptions-An unsupervised machine learning approach	Nagata K., Tsuji T., Suetsugu K., Muraoka K., Watanabe H., Kanaya A., Egashira N., Ieiri I.	2021	yes	The study focuses on clinical applications, specifically detecting overdose and underdose prescriptions in healthcare settings, rather than purely technical, privacy, methodological or educational aspects.	yes	The study uses tabular prescription data including age, weight, and dose features, and does not mention any non-tabular data formats like imaging or time-series data.	yes	The study uses real prescription data from Kyushu University Hospital, which is human clinical data.	yes	The study appears to be an original research article with clear methodology and results.	no	The study does not create synthetic data using AI methods. Instead, it creates synthetic overdose/underdose prescriptions by manipulating existing data (using maximum and minimum doses from drug labels).	no	While the study uses synthetic data, it's not generated using AI methods like GANs or VAEs. The synthetic data is created through simple manipulation of existing data ranges.	yes	The study demonstrates direct clinical application in detecting prescription errors that could lead to adverse drug events.	yes	The study includes original research with clear empirical evaluation and metrics for assessing performance.	yes	The study reports clear quantifiable outcomes including precision, recall, and F-measure metrics for detection performance.	Exclude	The study demonstrates strong clinical relevance in prescription error detection using real-world hospital data, with clear quantifiable outcomes and empirical evaluation. However, the research fails to meet key criteria related to AI-based synthetic data generation, as the synthetic data is created through simple data manipulation rather than advanced machine learning techniques. Despite robust clinical application and methodological rigor, the lack of AI-driven synthetic data generation represents a significant limitation.	2.5	Exclude	Exclude	No synthetic data generation
Development and validation of a mathematical model to simulate human cardiovascular and respiratory responses to battlefield trauma.	Jin, Xin, Laxminarayan, Srinivas, Nagaraja, Sridevi, Wallqvist, Anders, Reifman, Jaques	2023	maybe	The paper focuses on clinical applications, specifically for military medical applications and clinical decision-support systems. However, it's primarily focused on developing a mathematical model rather than direct clinical applications.	maybe	The study deals with physiological parameters (MAP, ETCO2, SpO2, PaO2, HR) which are tabular data. However, it's not clear if the focus is on generating synthetic tabular data or just modeling physiological responses.	yes	The study uses experimental data from 27 human subjects and focuses on human physiological responses.	yes	This is clearly an original research article with novel model development and empirical evaluation.	no	The study uses mathematical modeling rather than AI-based synthetic data generation. It explicitly mentions "mathematical models" and doesn't mention any AI-based data generation techniques.	no	While the study aims to generate synthetic data, it uses mathematical modeling rather than AI methods like GANs or VAEs.	maybe	While the study has potential clinical applications (training AI clinical decision-support systems), it doesn't directly demonstrate clinical applications. The focus is on model development.	yes	The study includes original research with clear empirical evaluation and metrics.	yes	The study reports clear quantifiable outcomes (RMSEs for various physiological parameters).	Exclude	The study focuses on mathematical modeling of physiological responses for military medical applications, using human clinical data and reporting quantifiable outcomes. However, it does not meet key screening criteria related to AI-based synthetic data generation, as it employs traditional mathematical modeling techniques rather than AI methods for data creation. While the research demonstrates empirical evaluation and potential clinical relevance, it falls short of the systematic review's specific requirements for AI-driven synthetic data generation.	2.5	Exclude	Exclude	No synthetic data generation

Development of a cerebellar ataxia diagnosis model using conditional GAN-based synthetic data generation for visuomotor adaptation task	Kim J., Woo S.-H., Kim T., Yoon W.T., Shin J.H., Lee J.-Y., Ryu J.-K.	2024	yes	The study focuses on clinical applications (cerebellar ataxia classification) rather than purely technical aspects, though there is significant methodological focus.	maybe	The study uses trajectory data from visuomotor adaptation tasks, which appears to be time-series data rather than purely tabular data, which is a critical mismatch with the criteria.	yes	The study uses human clinical data (cerebellar ataxia patients, normal individuals, and young healthy subjects).	yes	Based on the abstract, this appears to be an original research article with novel methodology and results.	yes	The study actively creates synthetic data using AI (conditional adversarial generative neural networks).	yes	The study clearly uses AI methods (GANs and reconstruction networks) for synthetic data generation.	yes	The study demonstrates direct clinical application in disease classification and diagnosis.	yes	The study includes empirical evaluation with clear metrics for assessing synthetic data's utility in classification.	yes	The study reports quantifiable outcomes (classification accuracy) and compares results between real and synthetic data.	Exclude	The study demonstrates strong clinical applications in cerebellar ataxia classification using AI-generated synthetic data, with robust methodology and quantifiable outcomes. However, the use of trajectory/time-series data instead of tabular data represents a critical deviation from the specified screening criteria, potentially disqualifying the research from further consideration. Despite meeting most other criteria with high fidelity, the data format mismatch presents a significant barrier to inclusion.	2.5	Exclude	Exclude	No tabular data
Diffusion-based conditional ECG generation with structured state space models	Alcaraz J.M.L., Strodthoff N.	2023	maybe	The paper focuses on generating synthetic ECG data, which is primarily a technical/methodological contribution rather than a clinical application. While it has potential clinical implications, the abstract doesn't demonstrate direct clinical applications.	no	The study focuses on ECG data, which is time-series data, not tabular data.	maybe	The study uses ECG statements from clinical data, though the exact nature of the data source isn't explicitly stated.	yes	This appears to be an original research article presenting a new method (SSSD-ECG).	yes	The study clearly involves active creation of synthetic data using AI (diffusion models and state space models).	yes	The study uses AI methods (diffusion models and state space models) for synthetic data generation.	no	While the study generates clinical data, it doesn't demonstrate direct clinical applications. The evaluation focuses on technical aspects (classifier performance, Turing test) rather than clinical utility.	maybe	The study includes empirical evaluation with clear metrics, but these are focused on technical performance rather than clinical utility.	maybe	The study reports quantifiable outcomes, but they are technical metrics (classifier performance) rather than clinical outcomes.	Exclude	The study focuses on generating synthetic ECG data using AI techniques, but fails to meet key screening criteria due to its time-series data format and lack of direct clinical applications. While demonstrating technical innovation in synthetic data generation, the research does not align with the systematic review's requirements for tabular data and clinical utility.	2.5	Exclude	Exclude	No tabular data
Does synthetic data augmentation improve the performances of machine learning classifiers for identifying health problems in patient-nurse verbal communications in home healthcare settings?.	Scroggins, Jihye Kim, Topaz, Maxim, Song, Jiyoun, Zolnoori, Maryam	2025	maybe	The study focuses on clinical applications (identifying health problems in patient-nurse communication) rather than purely technical aspects, but it also has a significant methodological component.	no	The study deals with audio recordings and transcribed text, not tabular data, which is a clear violation of the criterion requiring only tabular data generation.	yes	The study uses real-world patient-nurse communication data from home healthcare settings.	yes	This appears to be an original research article with clear methodology and results.	yes	The study actively creates synthetic data using GPT-4, an AI model, for generating synthetic patient-nurse communication.	maybe	While the study does use AI (GPT-4) for synthetic data generation, it's generating text/audio data rather than tabular data.	yes	The study demonstrates direct clinical applications in identifying health problems in home healthcare settings.	yes	The study includes original research with clear empirical evaluation and metrics (F1 scores) for assessing the utility of synthetic data.	yes	The study reports quantifiable outcomes in terms of F1 scores for machine learning classifiers.	Exclude	The study focuses on generating synthetic patient-nurse communication using AI, demonstrating clinical applications in home healthcare problem identification. However, the research primarily involves audio and text data rather than tabular data, which critically disqualifies it from meeting key screening requirements. Despite strong methodological rigor and quantifiable outcomes, the non-tabular data format represents a fundamental exclusion criterion.	2.5	Exclude	Exclude	No tabular data
Early Detection of Health Changes in the Elderly Using In-Home Multi-Sensor Data Streams	Wu W., Keller J.M., Skubic M., Popescu M., Lane K.R.	2021	yes	The study focuses on clinical applications, specifically monitoring elderly health and detecting early signs of health changes to enable preventive care.	no	The study primarily deals with sensor data (motion, bed, and depth sensors) and time-series data, not tabular data generation.	yes	The study uses real human clinical data from TigerPlace residents and their electronic health records.	yes	This appears to be an original research article with empirical evaluation.	no	While the study mentions using synthetic data for validation, it doesn't focus on creating synthetic data using AI. The synthetic data appears to be used as a validation tool rather than the main focus.	no	The study doesn't involve generating synthetic tabular data using AI methods. The synthetic data mentioned is used for validation purposes only.	yes	The study demonstrates direct clinical applications in monitoring elderly health and providing early warnings for health events.	yes	The study includes original research with empirical evaluation using real-world data and clear metrics.	yes	The study reports quantifiable outcomes in terms of health warning alerts and their correlation with documented health events.	Exclude	The study focuses on elderly health monitoring using sensor data, but fails to meet key screening criteria related to synthetic tabular data generation and AI-driven data creation. While demonstrating strong clinical applications and empirical evaluation, the research does not align with the specific requirements of synthetic data generation using AI methods. The paper's primary focus on sensor-based health tracking diverges from the targeted screening criteria.	2.5	Exclude	Exclude	No tabular data
ECG-based estimation of respiration-induced autonomic modulation of AV nodal conduction during atrial fibrillation	Plappert F., Engstrom G., Platonov P.G., Wallman M., Sandberg F.	2024	yes	The study focuses on clinical applications, specifically analyzing atrial fibrillation and autonomic nervous system activity for potential treatment insights.	no	The study primarily deals with time-series ECG data and RR series, which are explicitly excluded in the criteria as non-tabular formats.	yes	The study uses both synthetic data and real clinical data from 28 AF patients.	yes	This appears to be an original research article with novel methodology and results.	yes	The study actively creates synthetic data using a network model of the AV node with 4 million unique parameter sets.	maybe	While the study does generate synthetic data, it's unclear if AI methods like GANs or VAEs are used. The abstract mentions a "network model" but doesn't specify the type of AI used.	yes	The study demonstrates direct clinical applications in analyzing atrial fibrillation and autonomic nervous system activity for potential treatment insights.	yes	The study includes empirical evaluation with clear metrics for assessing the performance of their approach.	yes	The study reports quantifiable outcomes including Pearson correlation coefficients (r = 0.805, 0.830, 0.837, 0.855).	Exclude	The study demonstrates strong clinical relevance and original research in analyzing atrial fibrillation using synthetic data generation, with quantifiable outcomes and empirical evaluation. However, the primary focus on time-series ECG data disqualifies it from meeting the screening criteria, specifically the requirement for tabular data generation. Despite meeting most other criteria, this fundamental deviation prevents the study from passing the screening process.	2.5	Exclude	Exclude	No tabular data
Elasticity imaging using physics-informed neural networks: Spatial discovery of elastic modulus and Poisson's ratio	Kamali A., Sarabian M., Laksari K.	2023	maybe	The paper focuses on clinical applications (tumor diagnosis and tissue characterization) but also has significant technical components. While it has clinical relevance, it's primarily a technical methodology paper.	no	The study deals with imaging data and mechanical properties, not tabular data. It explicitly mentions "elasticity imaging" and spatial distributions of mechanical properties.	maybe	The study includes some human tissue (brain slice) but primarily uses hydrogel samples and simulated data.	yes	Yes, this is an original research article with novel methodology and validation.	no	While the paper uses AI (PINNs), it's not creating synthetic data for clinical use. Instead, it uses AI to analyze real and simulated mechanical data.	no	The paper uses finite element modeling to generate synthetic data, but this is not AI-generated synthetic tabular data as specified in the criteria.	maybe	The paper demonstrates potential clinical applications in tumor diagnosis and tissue characterization, though these are more theoretical than practical implementations.	maybe	The study includes empirical evaluation with clear metrics, but these are focused on technical performance rather than clinical utility.	maybe	The study reports quantifiable outcomes in terms of material property estimation and accuracy, but these are more technical than clinical metrics.	Exclude	The study primarily focuses on technical methodology for elasticity imaging using physics-informed neural networks, with limited direct clinical application and no generation of synthetic tabular data. While demonstrating potential clinical relevance in tumor diagnosis and tissue characterization, the research does not meet key screening criteria related to data format, synthetic data generation, and clinical utility metrics. The paper's technical approach and experimental validation, though innovative, fall short of the systematic review's specific requirements.	2.5	Exclude	Exclude	No tabular data
EmiNet: Moving bacteria detection on optical endomicroscopy images trained on synthetic data	Demirel M., Mills B., Gaughan E., Dhaliwal K., Hopgood J.R.	2025	maybe	The paper focuses on clinical applications (pneumonia diagnosis) rather than purely technical aspects, but it is primarily focused on the technical development of EmiNet.	no	The study deals with imaging data (optical endomicroscopy images), not tabular data, which is a clear violation of the criterion.	yes	The study uses real clinical data from human patients (optical endomicroscopy images from pneumonia patients).	yes	This appears to be an original research article with novel methodology and empirical evaluation.	maybe	The study does create synthetic data, but it's for images rather than tabular data. While it does involve active creation, it's not the type of data specified in the criteria.	maybe	While the study does generate synthetic data using AI methods, it's for images rather than tabular data. The synthetic data generation is confirmed through a Visual Turing Test.	yes	The study demonstrates direct clinical application in pneumonia diagnosis and bacterial detection.	yes	The study includes original research with clear empirical evaluation and metrics for assessing performance.	yes	The study reports quantifiable outcomes, specifically a 6.8% improvement in detection correlation.	Exclude	The study focuses on pneumonia diagnosis using optical endomicroscopy and AI-driven image analysis, but critically fails to meet the screening criteria due to its use of imaging data instead of tabular data. Despite demonstrating strong clinical application, empirical evaluation, and quantifiable outcomes, the fundamental requirement of tabular data generation is not satisfied, rendering the study non-compliant with the specified screening criteria.	2.5	Exclude	Exclude	No tabular data
Enriching Data Science and Health Care Education: Application and Impact of Synthetic Data Sets Through the Health Gym Project.	Kuo, Nicholas I-Hsien, Perez-Concha, Oscar, Hanly, Mark, Mnatzaganian, Emmanuel, Hao, Brandon, Di Sipio, Marcus, Yu, Guolin, Vanjara, Jash, Valerie, Ivy Cerelia, de Oliveira Costa, Juliana, Churches, Timothy, Lujic, Sanja, Hegarty, Jo, Jorm, Louisa, Barbieri, Sebastiano	2024	no	The paper primarily focuses on educational applications rather than clinical applications, as the abstract explicitly states it's for "hands-on education in health data science" and discusses educational applications.	yes	The study appears to generate tabular data for various clinical scenarios (sepsis, acute hypotension, HIV therapy).	yes	The synthetic data is based on real clinical scenarios involving human patients (sepsis, hypotension, HIV therapy).	yes	This appears to be an original research article describing the development and application of synthetic data sets.	yes	The study involves active creation of synthetic data through their Health Gym platform.	maybe	While synthetic data is generated, the abstract doesn't specify the AI methods used (GANs, VAEs, etc.).	no	The paper focuses on educational applications rather than direct clinical applications. There's no mention of using the synthetic data for diagnosis, prognosis, or treatment planning.	no	While there is evaluation, it's focused on educational applications rather than clinical utility. No clear clinical metrics are mentioned.	no	The abstract doesn't mention any quantifiable clinical outcomes or metrics. The focus is on educational utility rather than clinical performance.	Exclude	The study focuses on generating synthetic health data for educational purposes, primarily targeting data science training rather than direct clinical applications. While the research involves creating synthetic tabular data based on human clinical scenarios, it lacks quantifiable clinical outcomes and does not demonstrate direct clinical utility. The paper's primary aim is to support hands-on learning in health data science, falling short of the screening criteria's emphasis on clinical research and empirical evaluation.	2.5	Exclude	Exclude	No direct clinical application
Evaluation of synthetic training data for 3D intraoral reconstruction of cleft patients from single images	Lingens L., Lill Y., Nalabothu P., Benitez B.K., Mueller A.A., Gross M., Solenthaler B.	2025	maybe	The paper focuses on clinical applications (3D reconstruction for cleft lip and palate patients) rather than purely technical aspects, but it has a strong technical component.	no	The study deals with image data (intraoral scans and videos) and 3D reconstructions, not tabular data.	yes	The study uses real patient data from cleft lip and palate patients.	yes	This appears to be an original research article with novel methodology and results.	maybe	The study does generate synthetic data, but it's unclear if AI methods are used for this generation. The abstract mentions "synthetic datasets" but doesn't specify the generation method.	maybe	While synthetic data is used, the abstract doesn't specify if AI methods (GANs, VAEs, etc.) were used for generation. The focus seems to be on using the synthetic data rather than generating it.	yes	The study demonstrates direct clinical applications in 3D reconstruction for medical diagnosis/treatment planning.	maybe	The study includes empirical evaluation comparing synthetic and real data performance, though specific metrics aren't detailed in the abstract.	maybe	While the abstract mentions "satisfying results," it doesn't provide specific quantifiable metrics for evaluation.	Exclude	The study involves 3D reconstruction for cleft lip and palate patients using synthetic and real data, but critically fails to meet the tabular data format requirement. While demonstrating clinical application and using human patient data, the research focuses on image-based techniques rather than the specified tabular data generation criteria. Consequently, the paper does not align with the core screening requirements despite its innovative medical imaging approach.	2.5	Exclude	Exclude	No tabular data
Explainable Artificial Intelligence for Early Prediction of Pressure Injury Risk	Alderden J., Johnny J., Brooks K.R., Wilson A., Yap T.L., Zhao Y.L., van der Laan M., Kennerly S.	2024	yes	The study focuses on clinical applications, specifically hospital-acquired pressure injury risk assessment in ICUs, rather than purely technical, privacy, methodological or educational aspects.	yes	The study appears to use tabular data from the MIMIC ICU database, with no mention of imaging or other non-tabular formats.	yes	The study uses real ICU patient data from MIMIC, involving 28,395 patients.	yes	This appears to be an original research article with clear methodology and results.	maybe	The abstract mentions using synthetic data only for the explainer dashboard, not for the primary analysis. The main study uses real patient data.	maybe	While synthetic data is mentioned, it's only used for the explainer dashboard, not for the primary analysis. The abstract doesn't specify the method of synthetic data generation.	yes	The study has direct clinical applications in pressure injury risk assessment and prevention.	yes	The study includes clear empirical evaluation with cross-validation and specific performance metrics.	yes	The study reports quantifiable outcomes, specifically the area under the ROC curve (0.80) for the model's performance.	Exclude	The study demonstrates strong clinical relevance in hospital-acquired pressure injury risk assessment using real ICU patient data, with clear quantifiable outcomes and empirical evaluation. However, the research fails to meet key criteria related to synthetic data generation, as synthetic data is only used peripherally for an explainer dashboard rather than as a primary analytical approach. Consequently, the paper does not align with the core requirements for synthetic data-focused research.	2.5	Exclude	Exclude	No synthetic data generation
Explanation and prediction of clinical data with imbalanced class distribution based on pattern discovery and disentanglement.	Zhou, Pei-Yuan, Wong, Andrew K C	2021	maybe	The paper focuses on clinical applications, specifically diagnostic/prognostic prediction and pattern discovery in clinical data. However, it appears to be more methodological in nature, focusing on developing a new pattern discovery approach (cPDD).	yes	The study deals with tabular data ("relational dataset is a large table"), meeting this criterion.	yes	The study uses thoracic clinical dataset, which implies human clinical data.	yes	This appears to be an original research article presenting a novel method (cPDD).	no	The abstract mentions using synthetic data but doesn't indicate that they created it using AI methods. Instead, it appears they used existing synthetic data for validation.	no	While synthetic data is mentioned, there's no indication that the study involves generating synthetic data using AI methods. The focus is on pattern discovery and classification.	yes	The study demonstrates direct clinical applications in diagnosis and prognosis.	yes	The study includes empirical evaluation with clear metrics for assessing the method's performance.	yes	The study reports quantifiable outcomes in terms of pattern discovery performance and prediction accuracy.	Exclude	The study presents a novel pattern discovery method for clinical data analysis, demonstrating strong clinical applications and empirical evaluation using human clinical data. However, the research fails to meet key criteria related to synthetic data generation using AI methods, focusing instead on a methodological approach to pattern discovery and classification. While the study offers valuable insights into clinical data interpretation, it does not align with the specific requirements for synthetic data generation.	2.5	Exclude	Exclude	No synthetic data generation

GAN-Augmented Naive Bayes for identifying high-risk coronary artery disease patients using CT angiography data.	Zhang, Lei, Haldorai, Anandakumar, Naik, Nithesh	2024	yes	The study focuses on clinical applications, specifically the diagnosis and risk assessment of coronary artery disease, rather than purely technical, privacy, methodological or educational aspects.	maybe	The study primarily uses imaging data (CCTA) and generates synthetic patient profiles based on this imaging data, which violates the criterion requiring only tabular data generation.	yes	The study uses real clinical data from 5,000 individuals with CCTA records.	yes	Based on the abstract, this appears to be an original research article with novel methodology and results.	yes	The study actively creates synthetic data using GANs, with a clear generator-discriminator framework described.	yes	The study explicitly uses GANs for synthetic data generation, meeting this criterion.	yes	The study demonstrates direct clinical application in diagnosis and risk assessment of CAD patients.	yes	The study includes clear empirical evaluation with specific metrics and comparison against conventional machine learning models.	yes	The study reports multiple quantifiable metrics including accuracy, sensitivity, specificity, ROC, Dice Similarity Coefficient, and precision.	Exclude	A comprehensive study on coronary artery disease using GANs for synthetic data generation demonstrates strong clinical application and empirical evaluation across multiple performance metrics. While the research exhibits robust methodology and quantifiable outcomes, the primary use of imaging data (CCTA) disqualifies it from meeting the strict tabular data generation requirement. The study's innovative approach to patient risk assessment is technically sound but falls short of the specific screening criteria.	2.5	Exclude	Exclude	No tabular data
GatewayNet: a form of sequential rule mining	Kilgore P.C.S.R., Korneeva N., Arnold T.C., Trutschl M., Cvek U.	2019	maybe	The paper focuses on clinical applications (drug abuse patterns and gateway hypothesis) rather than purely technical aspects, but it is primarily a methodological paper developing an algorithm.	maybe	The study uses tabular data (urine drug screening results), but also involves sequential/transactional data analysis.	yes	The study uses real human clinical data from emergency department urine drug screenings.	yes	This appears to be an original research article developing and testing a new algorithm.	no	The paper does not involve creating synthetic data using AI. While it mentions "synthetic data," this refers to testing data created using a Markov chain, not AI-generated synthetic data.	no	The study does not use AI methods for synthetic data generation. The synthetic data mentioned is created using a Markov chain, which is a statistical method.	maybe	While the study has clinical relevance to substance abuse patterns, it's primarily focused on developing and testing an algorithm rather than direct clinical applications.	maybe	The study includes empirical evaluation using real clinical data, but the metrics for assessing utility are not clearly specified in the abstract.	maybe	The abstract mentions finding "gateway rules" and "documented transitions," but doesn't clearly report specific quantifiable clinical metrics.	Exclude	The study involves clinical data analysis of drug abuse patterns using a novel algorithm, but lacks key requirements such as AI-generated synthetic data and direct clinical application metrics. While the research uses human clinical data and demonstrates empirical evaluation, it primarily focuses on methodological development rather than meeting the specific screening criteria for synthetic data generation and clinical utility.	2.5	Exclude	Exclude	No synthetic data generation
Generalising electrocardiogram detection and delineation: training convolutional neural networks with synthetic data augmentation	Jimenez-Perez G., Acosta J., Alcaine A., Camara O.	2024	maybe	The paper focuses on clinical applications (ECG analysis and detection) rather than purely technical aspects, but it also has a strong methodological component.	no	The study deals with ECG data, which is time-series data, not tabular data, and this is a clear violation of the inclusion criteria.	yes	The study uses human clinical ECG data from multiple databases (QT, LU, and Zhejiang).	yes	This appears to be an original research article with novel contributions.	yes	The study does involve active creation of synthetic data using AI, specifically "probabilistically constructs unseen ECG traces."	maybe	While the study does generate synthetic data, it's not clear if it uses AI methods like GANs or VAEs. The abstract mentions "probabilistic" construction but doesn't specify the AI methods used.	yes	The study demonstrates direct clinical applications in ECG analysis and detection, which is crucial for diagnosis.	yes	The study includes original research with clear empirical evaluation and metrics.	yes	The study reports clear quantifiable outcomes (F1-score of 99.38% and specific delineation errors).	Exclude	The study focuses on ECG data analysis using synthetic data generation, demonstrating strong clinical applications and quantifiable outcomes. However, the use of time-series ECG data instead of tabular data represents a critical deviation from the specified screening criteria, potentially disqualifying the research from further consideration. Despite meeting multiple other criteria, the data format requirement appears to be a decisive factor in the screening process.	2.5	Exclude	Exclude	No tabular data
Generating synthetic mixed-type longitudinal electronic health records for artificial intelligent applications	Cairns B.J., Li J., Zhu T.	2023	maybe	The paper focuses on clinical applications, specifically generating synthetic EHR data for intensive care unit applications. However, it has a strong technical component in developing the GAN model, so it only partially meets this criterion.	no	The study focuses on time-series EHR data, which is explicitly excluded in the criteria as it's not purely tabular data.	yes	The study uses real EHR data from 141,488 unique patients in ICU settings, so it meets this criterion.	yes	This appears to be an original research article with novel methodology and empirical evaluation, so it meets this criterion.	yes	The study actively creates synthetic data using a novel GAN approach (EHR-M-GAN), so it clearly meets this criterion.	yes	The study uses AI methods (specifically GANs) to generate synthetic data, so it clearly meets this criterion.	yes	The study demonstrates clinical applications by showing improved performance in intensive care outcome prediction models, so it meets this criterion.	yes	The study includes clear empirical evaluation with validation on three ICU databases and comparison against benchmarks, so it meets this criterion.	yes	The study reports quantifiable outcomes in terms of prediction model performance and privacy risk evaluation, so it meets this criterion.	Exclude	The study presents a novel generative adversarial network for synthetic electronic health record data generation, demonstrating strong clinical applications and empirical validation across multiple ICU databases. However, the focus on time-series data fundamentally conflicts with the screening criteria's requirement for purely tabular data generation, rendering the study non-compliant despite its methodological rigor. The research successfully addresses multiple screening criteria but fails the critical data format requirement.	2.5	Exclude	Exclude	No tabular data
Generative AI mitigates representation bias and improves model fairness through synthetic health data.	Marchesi, Raffaele, Micheletti, Nicolo, I-Hsien Kuo, Nicholas, Barbieri, Sebastiano, Jurman, Giuseppe, Osmani, Venet	2025	maybe	The paper focuses on clinical applications, specifically addressing representation bias in health data and improving fairness in clinical predictions. However, it has a strong methodological component.	no	The study explicitly deals with "high-dimensional time-series synthetic health data," which is specifically excluded in the screening criteria.	yes	The study uses real-world clinical datasets from 7,535 patients with hypotension and sepsis.	yes	Based on the abstract, this appears to be an original research article with novel methodology and empirical evaluation.	yes	The study clearly involves active creation of synthetic data using AI, specifically developing a novel CA-GAN architecture.	yes	The study uses GANs (specifically CA-GAN) for synthetic data generation, which is an AI method.	maybe	While the study has clinical relevance in improving fairness in clinical predictions, it's not clearly demonstrating direct clinical applications like diagnosis or treatment planning.	yes	The study includes empirical evaluation using real clinical datasets and reports comparative performance against state-of-the-art methods.	maybe	The abstract mentions quantitative evaluation and improved performance in predictive tasks, though specific metrics aren't detailed.	Exclude	The study involves synthetic data generation using AI for clinical datasets, demonstrating empirical evaluation and human clinical data usage. However, the focus on time-series data and methodological improvements rather than direct clinical applications creates significant screening challenges. The novel CA-GAN approach shows promise in addressing representation bias, but does not fully align with all predefined screening criteria.	2.5	Exclude	Exclude	No tabular data
Generative artificial intelligence to produce high fidelity blastocyst-stage embryo images.	Cao, Ping, Derhaag, Josien, Coonen, Edith, Brunner, Han, Acharya, Ganesh, Salumets, Andres, Zamani Esteki, Masoud	2024	maybe	The paper focuses on generating synthetic images for IVF embryo selection, which is a clinical application. However, the primary focus appears to be on the technical aspects of image generation rather than direct clinical applications.	no	The study focuses on image data, not tabular data. This is a clear violation of the inclusion criteria which specifically requires tabular data generation.	yes	The study uses human embryo images from IVF procedures, which qualifies as human clinical data.	yes	This appears to be an original research article with empirical evaluation, not a review or commentary.	yes	The study does involve active creation of synthetic data using AI (specifically GANs).	maybe	While the study does use AI methods (GANs) for synthetic data generation, it's for images rather than tabular data.	maybe	While the study has potential clinical applications in IVF embryo selection, it doesn't directly demonstrate clinical applications in the study itself. It's more focused on the technical capability of generating synthetic images.	maybe	The study includes empirical evaluation with clear metrics, but again, these are focused on technical performance rather than clinical utility.	maybe	The study reports quantifiable outcomes (FID and KID scores, visual Turing test results), but these are focused on image quality rather than clinical outcomes.	Exclude	The study focuses on generating synthetic embryo images using AI, which primarily involves technical image generation rather than direct clinical applications. While the research uses human clinical data and demonstrates original AI-driven synthetic data creation, it critically fails to meet the screening criteria by working with image data instead of the required tabular format. The technical nature of the study and its focus on image generation techniques disqualify it from meeting the systematic review's core requirements.	2.5	Exclude	Exclude	No tabular data
How good is your synthetic data? SynthRO, a dashboard to evaluate and benchmark synthetic tabular data.	Santangelo, Gabriele, Nicora, Giovanna, Bellazzi, Riccardo, Dagliati, Arianna	2025	no	The paper focuses on a technical tool (SynthRO) for evaluating synthetic data rather than direct clinical applications. While it mentions healthcare applications, the primary focus is methodological.	yes	The paper explicitly mentions "synthetic tabular data" and focuses on this format.	maybe	The paper mentions patient data and Electronic Health Records, but it's unclear if the tool itself uses real human clinical data or just evaluates synthetic versions.	yes	This appears to be an original research article presenting a new tool (SynthRO), not a review or commentary.	no	The paper doesn't directly create synthetic data but rather evaluates existing synthetic data generation methods. It's a benchmarking tool rather than a data generation method.	no	While the paper deals with synthetic data, it doesn't generate it but rather evaluates existing synthetic data. It's focused on benchmarking rather than generation.	no	While the paper mentions potential clinical applications (prognostic scores, federated learning), it doesn't demonstrate direct clinical applications but rather provides a tool for evaluating synthetic data.	no	The paper includes evaluation of synthetic data, but not in terms of clinical utility. It focuses on technical evaluation metrics.	no	The paper focuses on evaluation metrics and quantitative scores, but these are for evaluating synthetic data quality rather than clinical outcomes.	Exclude	The study presents a technical tool for evaluating synthetic healthcare data, focusing on methodological aspects rather than direct clinical applications. While addressing synthetic tabular data and providing a benchmarking approach, the research lacks original data creation, empirical clinical evaluation, and quantifiable clinical outcomes. The paper primarily offers a methodological framework for assessing synthetic data quality, falling short of meeting several critical screening criteria for clinical research.	2.5	Exclude	Exclude	No synthetic data generation
Improved outcome prediction in acute pancreatitis with generated data and advanced machine learning algorithms	Ozdede M., Batur A., Aksoy A.E.	2025	yes	The study focuses on clinical applications, specifically predicting acute pancreatitis severity using machine learning models, rather than purely technical, privacy, methodological or educational aspects.	yes	The study appears to use tabular clinical data, as evidenced by the use of scoring systems and machine learning models that typically operate on tabular data.	yes	The study uses data from 101 AP patients in a tertiary hospital, confirming the use of human clinical data.	yes	This is clearly an original research article with empirical evaluation and results.	no	The study uses Gaussian noise addition and ADASYN algorithm for data augmentation, which are traditional statistical methods rather than AI-based synthetic data generation methods like GANs or VAEs.	no	While the study does generate synthetic data, it uses traditional statistical methods (Gaussian noise addition) rather than AI methods for synthetic data generation.	yes	The study demonstrates direct clinical application in predicting acute pancreatitis severity and adverse outcomes.	yes	The study includes original research with clear empirical evaluation and metrics for assessing performance.	yes	The study reports clear quantifiable outcomes including AUC, F1 score, and recall metrics for both ML models and traditional scoring systems.	Exclude	The study demonstrates strong clinical relevance in predicting acute pancreatitis severity using machine learning, with clear empirical evaluation and quantifiable outcomes based on human clinical data. However, the synthetic data generation method relies on traditional statistical techniques rather than AI-based approaches, which disqualifies it from meeting specific screening criteria related to AI-driven synthetic data creation.	2.5	Exclude	Exclude	No synthetic data generation
Improving CNN Training using Disentanglement for Liver Lesion Classification in CT.	Ben-Cohen, Avi, Mechrez, Roey, Yedidia, Noa, Greenspan, Hayit	2019	maybe	The paper focuses on medical image analysis for liver lesion classification, which is a clinical application. However, it appears to be primarily focused on the technical aspects of data generation rather than the clinical application.	no	The study deals with medical imaging (CT scans), not tabular data.	maybe	While the study involves liver lesions in CT scans, which implies human clinical data, it's not explicitly stated whether the original data is from human patients.	yes	This appears to be an original research article with novel methodology and experimental results.	yes	The study involves active creation of synthetic data using AI (neural networks and disentanglement-based scheme).	yes	The study uses AI methods (neural networks) for synthetic data generation.	maybe	The study focuses on liver lesion classification, which is a clinical application. However, the abstract doesn't clearly demonstrate direct clinical applications beyond the technical classification task.	yes	The study includes empirical evaluation with clear metrics (classification accuracy).	yes	The study reports quantifiable outcomes (7.4% improvement in accuracy).	Exclude	The study focuses on synthetic data generation for medical image analysis, specifically liver lesion classification using neural networks, but fails to meet key screening criteria due to its imaging-based approach rather than tabular data generation. While demonstrating technical innovation in AI-driven data synthesis and reporting quantifiable improvements in classification accuracy, the research does not align with the specified screening requirements for tabular clinical data generation.	2.5	Exclude	Exclude	No tabular data
Incorporating a-priori information in deep learning models for quantitative susceptibility mapping via adaptive convolution	Graf S., Wohlgemuth W.A., Deistung A.	2024	maybe	The paper focuses on quantitative susceptibility mapping (QSM) for tissue characterization in the brain, which is a clinical application. However, it appears to be more focused on the technical aspects of the imaging processing method rather than direct clinical applications.	no	The study deals with MRI phase images and susceptibility maps, which are imaging data, not tabular data.	yes	The study uses both synthetic and in-vivo (human) brain data.	yes	This appears to be an original research article presenting a new method (adaptive convolution 3D U-Net).	maybe	The paper mentions using synthetic data for pre-training, but it's unclear if they actively created this data or used existing synthetic datasets. The focus seems to be on the adaptive convolution method rather than synthetic data generation.	no	While synthetic data is mentioned, it's used as a pre-training step rather than being the focus of the study. The paper doesn't appear to focus on the generation of synthetic data using AI methods.	maybe	While the method has potential clinical applications for tissue characterization, the abstract focuses more on the technical aspects of the imaging processing method rather than demonstrating direct clinical applications.	yes	The study includes empirical evaluation with both synthetic and in-vivo data, including experiments on simulated pathologic datasets.	maybe	The abstract mentions that the model "outperformed" other approaches, but specific quantifiable outcomes are not clearly detailed in the abstract.	Exclude	The study primarily focuses on a technical imaging processing method for quantitative susceptibility mapping, utilizing MRI phase images rather than tabular data. While the research involves human clinical data and demonstrates empirical evaluation, it fails to meet key screening criteria related to synthetic data generation and direct clinical applications. The technical nature of the work and its emphasis on imaging processing techniques significantly limit its alignment with the specified screening requirements.	2.5	Exclude	Exclude	No tabular data
Inferring actual treatment pathways from patient records.	Wilkins-Caruana, Adrian, Bandara, Madhushi, Musial, Katarzyna, Catchpoole, Daniel, Kennedy, Paul J	2023	yes	The study focuses on clinical applications, specifically treatment pathway inference from healthcare records, which is a clinical application rather than purely technical.	yes	The study explicitly deals with "administrative health records" which are tabular data, and there is no mention of imaging or other non-tabular formats.	yes	The study uses real administrative health records from human patients, specifically mentioning breast cancer, lung cancer, and melanoma cases.	yes	This appears to be an original research article with clear methodology, results, and conclusions.	maybe	While the study mentions "synthetic data experiments," these appear to be used for testing rather than being the primary focus. The main focus is on analyzing real healthcare records.	maybe	The study does not focus on generating synthetic data. While synthetic data is used for testing, the paper's primary contribution is a pathway inference method (Defrag).	yes	The study has direct clinical applications in treatment pathway inference, which is relevant for clinical decision-making and patient care.	yes	The study includes clear empirical evaluation with a testing framework and comparison against baselines.	yes	The study reports comparative performance metrics against baselines and demonstrates effectiveness in identifying treatment pathways.	Exclude	The study demonstrates strong clinical relevance through treatment pathway inference using administrative health records, with clear empirical evaluation and quantifiable outcomes across multiple cancer types. However, the research does not meet key criteria related to synthetic data generation, as it primarily focuses on pathway inference methodology rather than creating synthetic datasets. While methodologically robust, the paper falls short of the specific synthetic data generation requirements.	2.5	Exclude	Exclude	No synthetic data generation

Label-informed cardiac magnetic resonance image synthesis through conditional generative adversarial networks	Amirrajab S., Al Khalil Y., Lorenz C., Weese J., Pluim J., Breeuwer M.	2022	maybe	The paper focuses on technical aspects of image synthesis rather than direct clinical applications. While it has potential clinical implications, the primary focus is on the technical methodology of generating synthetic CMR images.	no	The study focuses on medical imaging data (CMR images), not tabular data.	yes	The study uses real CMR images from human patients as its basis.	yes	This appears to be an original research article with novel methodology and empirical evaluation.	yes	The study does involve active creation of synthetic data using AI (specifically GANs).	maybe	While the study does use AI methods (GANs) for synthetic data generation, it's for images rather than tabular data.	maybe	While the study has potential clinical applications, it focuses on the technical aspects of image synthesis and segmentation rather than direct clinical applications like diagnosis or treatment planning.	yes	The study includes clear empirical evaluation with specific metrics for assessing the synthetic data's utility.	yes	The study reports quantifiable outcomes (Dice score and Hausdorff Distance) for the segmentation task.	Exclude	The study focuses on generating synthetic Cardiac Magnetic Resonance images using AI techniques, which fundamentally conflicts with the requirement for tabular data generation. While demonstrating sophisticated technical methodology and empirical evaluation, the research fails to meet critical screening criteria related to data format and direct clinical applications. Consequently, the paper does not align with the systematic review's core requirements.	2.5	Exclude	Exclude	No tabular data
Laryngeal pressure estimation with a recurrent neural network	Gomez P., Schutzenberger A., Semmler M., Dollinger M.	2019	maybe	The paper focuses on clinical applications (voice diagnosis and treatment selection) but also has significant technical components. While it mentions clinical applications, these are presented as future possibilities rather than current implementations.	no	The study uses numerical data from a two-mass model and video recordings, but not tabular data in the traditional sense. The data appears to be more time-series/physiological in nature.	no	The study uses porcine (pig) vocal fold data, not human clinical data.	yes	This is clearly an original research article with novel methodology and empirical evaluation.	no	The study uses AI (LSTM network) to generate synthetic training data from a numerical model, but this is for training purposes rather than creating synthetic clinical data.	no	While the study does generate synthetic data, it's not generating synthetic tabular clinical data but rather synthetic numerical data for training purposes.	maybe	While the paper mentions potential clinical applications, it doesn't demonstrate direct clinical applications but rather focuses on the technical methodology.	yes	The study includes original research with clear empirical evaluation and metrics.	yes	The study reports clear quantifiable outcomes (21.2% vs 17.7% mean absolute percentage error).	Exclude	The study focuses on a technical methodology for estimating subglottal pressure using synthetic data from a numerical model, primarily using porcine vocal fold data rather than human clinical information. While demonstrating quantifiable outcomes and empirical evaluation, the research does not meet key screening criteria related to tabular data generation, human clinical data, or direct clinical applications. The work appears more oriented toward technical innovation than immediate clinical utility.	2.5	Exclude	Exclude	No tabular data
Learning Bayesian networks for clinical time series analysis	van der Heijden M., Velikova M., Lucas P.J.F.	2014	yes	The study focuses on clinical applications, specifically predicting COPD exacerbation events for chronic disease management.	no	The study deals with time series data from patient monitoring, which is explicitly excluded in the criteria.	yes	The study uses data from 10 COPD patients.	yes	This appears to be an original research article with methodology and results.	maybe	While the study mentions using synthetic data for testing, it's not clear if they actively created this data using AI methods or just used existing synthetic data.	maybe	The abstract mentions "synthetic data" but doesn't specify the generation method. It appears to be more focused on traditional machine learning methods (Bayesian networks, naive Bayes).	yes	The study has direct clinical application in predicting COPD exacerbation events.	yes	The study includes empirical evaluation with clear metrics and validation using external data.	yes	The study reports on predictive performance and mentions true/false positive rates.	Exclude	The study involves time series data from COPD patients, which conflicts with the specified data format criteria, and lacks clear evidence of AI-driven synthetic data generation. While demonstrating clinical application and empirical evaluation, the research does not meet key screening requirements related to data type and synthetic data methodology. Consequently, the paper would likely be excluded from further review based on these critical screening criteria.	2.5	Exclude	Exclude	No tabular data
Learning Chemotherapy Drug Action via Universal Physics-Informed Neural Networks.	Podina, Lena, Ghodsi, Ali, Kohandel, Mohammad	2025	maybe	The paper focuses on quantitative systems pharmacology for drug effects and toxicity assessment, which is a clinical application. However, it appears to be more methodological in nature, focusing on the UPINN technique rather than direct clinical applications.	no	The study uses synthetic data for differential equations modeling, but it's unclear if this is tabular data. The focus seems to be on mathematical modeling rather than tabular data generation.	no	The study focuses on mathematical modeling of chemotherapy effects and doesn't appear to use actual human clinical data. It mentions "toy examples" and mathematical models.	yes	This appears to be an original research article with novel applications of UPINNs.	no	While the study uses synthetic data, it appears to be generating synthetic data for mathematical modeling rather than creating synthetic clinical data using AI.	no	The study uses synthetic data, but not for tabular data generation using AI methods like GANs or VAEs. Instead, it uses UPINNs for mathematical modeling.	maybe	While the ultimate goal is related to clinical applications (drug effects and toxicity), the study itself focuses on mathematical modeling rather than direct clinical applications.	maybe	The study includes empirical evaluation, but it's focused on mathematical modeling performance rather than clinical utility.	maybe	The study reports on the ability to learn hidden terms and parameters in differential equations, but these aren't clinical outcomes.	Exclude	The study focuses on mathematical modeling of chemotherapy effects using UPINNs, but lacks direct clinical applications and human clinical data. While methodologically innovative, the research does not meet key screening criteria related to synthetic data generation, tabular data format, and empirical clinical outcomes. The paper appears more oriented toward technical modeling than practical clinical utility.	2.5	Exclude	Exclude	No tabular data
Linguistic summarization of in-home sensor data.	Jain, Akshay, Popescu, Mihail, Keller, James, Rantz, Marilyn, Markway, Brianna	2019	maybe	The paper focuses on clinical applications, specifically helping clinicians make sense of elderly care sensor data. However, it's more focused on data summarization than direct clinical decision-making.	no	The study deals with sensor data and natural language summaries, not tabular data generation.	yes	The study uses data from elderly patients in 110 apartments, so it involves human clinical data.	yes	This appears to be an original research article with methods, results, and conclusions.	maybe	While the abstract mentions using "real and synthetic data," it's unclear if they actively created synthetic data using AI methods. The focus seems to be on processing existing data.	no	The abstract doesn't specify the method of synthetic data generation or mention AI methods like GANs or VAEs.	maybe	While the system has clinical applications in monitoring elderly health, it's more focused on data summarization than direct clinical decision-making.	yes	The study includes empirical evaluation through surveys and retrospective case studies.	maybe	The abstract mentions "meaningful results comparable to human subjects" but doesn't provide specific quantifiable metrics.	Exclude	The study focuses on sensor data summarization for elderly care, but diverges from the screening criteria by not generating synthetic tabular data using AI methods. While involving human clinical data and demonstrating empirical evaluation, the research primarily addresses data processing and natural language summarization rather than synthetic data generation. Consequently, the paper does not align closely with the specified screening requirements.	2.5	Exclude	Exclude	No tabular data
Machine and Deep Learning Models for Hypoxemia Severity Triage in CBRNE Emergencies	Nanini S., Abid M., Mamouni Y., Wiedemann A., Jouvet P., Bourassa S.	2024	yes	The study focuses on clinical applications, specifically hypoxemia severity prediction during emergency triage, rather than purely technical, privacy, methodological or educational aspects.	yes	The study uses tabular physiological data from medical-grade sensors, which meets the requirement for tabular data generation.	yes	The study uses MIMIC-III and IV datasets, which are well-known human clinical datasets.	yes	This appears to be an original research article with clear methodology and results.	maybe	The abstract mentions "synthetic data flagged with masks" but does not indicate active creation of synthetic data. It appears to be using existing synthetic data rather than generating new synthetic data.	maybe	While synthetic data is mentioned, there is no indication of AI-based synthetic data generation methods (GANs, VAEs, etc.). The focus is on ML models for prediction.	yes	The study demonstrates direct clinical application in emergency triage and hypoxemia severity prediction.	yes	The study includes clear empirical evaluation with specific metrics and comparison of different models.	yes	The study reports quantifiable outcomes including performance metrics and F1-scores.	Exclude	The study demonstrates strong clinical application in emergency triage using machine learning models on human clinical data, with clear quantifiable outcomes and empirical evaluation. However, it fails to meet key criteria related to synthetic data generation, specifically lacking active creation of synthetic data using AI methods. Despite robust clinical relevance, the research does not align with the core requirements of synthetic data generation.	2.5	Exclude	Exclude	No synthetic data generation
Machine learning algorithms for detection of visuomotor neural control differences in individuals with PASC and ME	Ahuja H., Badhwar S., Edgell H., Litoiu M., Sergio L.E.	2024	maybe	The study focuses on clinical applications (PASC/ME detection and monitoring) rather than purely technical aspects, but there is significant technical focus on the EEG processing and AI methods.	no	The study primarily deals with EEG data and spectrogram matrices, which are time-series data formats, and this explicitly fails the criterion requiring only tabular data.	yes	The study uses human clinical data from patients with PASC and ME.	yes	Based on the abstract, this appears to be an original research article with novel methodology and results.	yes	The study does create synthetic data using WGANs, which meets this criterion.	maybe	The study uses WGANs to generate synthetic spectrograms, which is an AI method for synthetic data generation. However, this is applied to time-series data rather than tabular data.	yes	The study demonstrates direct clinical applications in diagnosis and monitoring of PASC/ME conditions.	yes	The study includes original research with clear empirical evaluation and metrics.	yes	The study reports clear quantifiable outcomes (accuracy metrics of 83% and 93%).	Exclude	The study involves synthetic data generation using WGANs for PASC/ME detection, demonstrating strong clinical applications and quantifiable outcomes. However, the research fundamentally fails the screening criteria due to its use of EEG time-series data instead of the required tabular data format. Despite meeting multiple other criteria, this critical deviation disqualifies the study from inclusion.	2.5	Exclude	Exclude	No tabular data
Markov blanket-based approach for learning multi-dimensional Bayesian network classifiers: An application to predict the European Quality of Life-5 Dimensions (EQ-5D) from the 39-item Parkinson's Disease Questionnaire (PDQ-39)	Borchani H., Bielza C., Marti nez-Martin P., Larranaga P.	2012	maybe	The paper focuses on clinical applications, specifically predicting quality of life (EQ-5D) for Parkinson's patients using PDQ-39 data. However, it appears to be primarily a methodological paper about Bayesian network classifiers.	yes	The study uses tabular data (PDQ-39 questionnaire data and EQ-5D outcomes), meeting this criterion.	yes	The study uses real-world Parkinson's disease data from 488 patients, meeting this criterion.	yes	This is an original research article with empirical evaluation and methodology development.	no	The abstract mentions "randomly generated synthetic data sets" but doesn't specify if these are AI-generated. The focus seems to be on the classification method rather than synthetic data generation.	no	While synthetic data is used, there's no indication that AI methods (GANs, VAEs, etc.) are used for generation. The abstract suggests random generation rather than AI-based synthesis.	maybe	The study demonstrates clinical application in predicting quality of life for Parkinson's patients, though this is more of a secondary outcome prediction rather than direct diagnosis or treatment.	yes	The study includes clear empirical evaluation with fivefold cross-validation and comparison with other methods.	yes	The study reports predictive accuracy and performance metrics, meeting this criterion.	Exclude	The study involves clinical data from Parkinson's patients and uses tabular data for quality of life prediction, demonstrating empirical research with quantifiable outcomes. However, the research lacks AI-based synthetic data generation and primarily focuses on a methodological approach to Bayesian network classifiers rather than synthetic data creation. Consequently, the paper does not fully meet the critical screening criteria related to AI-driven synthetic data generation.	2.5	Exclude	Exclude	No synthetic data generation
Missing data imputation and synthetic data simulation through modeling graphical probabilistic dependencies between variables (ModGraProDep): An application to breast cancer survival.	Vilardell, Mireia, Buxo, Maria, Cleries, Ramon, Martinez, Jose Miguel, Garcia, Gemma, Ameijide, Alberto, Font, Rebeca, Civit, Sergi, Marcos-Gragera, Rafael, Vilardell, Maria Loreto, Carulla, Maria, Espinas, Josep Alfons, Galceran, Jaume, Izquierdo, Angel, Borras, Josep Ma	2020	yes	The study focuses on clinical applications in breast cancer survival analysis, rather than purely technical, privacy, methodological or educational aspects.	yes	The study deals with tabular data, including survival data and clinical variables like "Stage".	yes	The study uses real breast cancer patient data from cancer registries in Spain.	yes	This appears to be an original research article with novel methodology and empirical evaluation.	maybe	While the study creates synthetic data, it uses graphical modeling (GM) rather than AI methods like GANs or VAEs, which is a significant deviation from the criterion.	no	The synthetic data generation is based on graphical modeling, not AI methods as specified in the criteria, which is a critical failure point.	yes	The study demonstrates direct clinical applications in breast cancer survival analysis and predictive risk analysis.	yes	The study includes original research with clear empirical evaluation and metrics.	yes	The study reports prediction performance metrics and compares different models' performance.	Exclude	The study demonstrates strong clinical relevance in breast cancer survival analysis using human patient data with quantifiable outcomes, but critically fails to meet the requirement of AI-based synthetic data generation. While the research presents a novel graphical modeling approach for handling missing data and synthetic dataset creation, it does not employ AI methods like GANs or VAEs as specified in the screening criteria. Consequently, the study does not fully align with the core synthetic data generation requirements.	2.5	Exclude	Exclude	No synthetic data generation
Mixed effect machine learning: A framework for predicting longitudinal change in hemoglobin A1c.	Ngufor, Che, Van Houten, Holly, Caffo, Brian S, Shah, Nilay D, McCoy, Rozalina G	2019	yes	The study focuses on clinical applications, specifically predicting glycemic control in type 2 diabetes patients.	yes	The study appears to work with tabular clinical data (HbA1c measurements and patient characteristics).	yes	The study uses real clinical data from type 2 diabetes patients.	yes	This appears to be an original research article presenting a new method (MEml) with empirical evaluation.	maybe	The abstract mentions using "real publicly available and synthetic data sets" but doesn't indicate active creation of synthetic data using AI. The focus is on developing a machine learning method (MEml) rather than generating synthetic data.	maybe	While synthetic data is mentioned, it's used as a validation tool rather than being the focus of the study. The paper doesn't describe generating synthetic data using AI methods.	yes	The study demonstrates direct clinical application in predicting glycemic control, which is relevant for disease management.	yes	The study includes original research with clear empirical evaluation and metrics.	yes	The study reports quantifiable outcomes, specifically accuracy metrics comparing MEml to other methods.	Exclude	The study presents a machine learning method for predicting glycemic control in type 2 diabetes patients, using real and synthetic clinical data with quantifiable outcomes. While demonstrating strong clinical application and empirical evaluation, the research does not focus on AI-driven synthetic data generation, which limits its alignment with specific screening criteria. The paper primarily develops a novel machine learning approach for longitudinal clinical data analysis rather than creating synthetic data using AI techniques.	2.5	Exclude	Exclude	No synthetic data generation
Multi-omics data integration and drug screening of AML cancer using Generative Adversarial Network.	Afroz, Sabrin, Islam, Nadira, Habib, Md Ahsan, Reza, Md Selim, Ashad Alam, Md	2024	yes	The study focuses on clinical applications, specifically predicting disease traits for Acute Myeloid Leukemia (AML) and identifying potential drug candidates.	no	The study deals with multi-omics data including gene activity and DNA methylation data, which are not strictly tabular data formats as specified in the criteria.	yes	The study uses human clinical data related to AML patients.	yes	This appears to be an original research article with novel methodology and results.	yes	The study actively creates synthetic data using Omics GAN, a generative adversarial network approach.	yes	The study explicitly uses GANs to generate synthetic data, meeting this criterion.	yes	The study demonstrates direct clinical applications in disease trait prediction and potential drug discovery.	yes	The study includes empirical evaluation comparing synthetic data performance against original data.	maybe	While the abstract mentions "improved prediction quality," it doesn't explicitly state specific quantifiable metrics.	Exclude	The study demonstrates strong clinical applications in Acute Myeloid Leukemia using synthetic data generation through GANs, with potential drug discovery implications. However, the multi-omics data format deviates from the strict tabular data requirement, and quantifiable outcomes are not explicitly detailed. These factors significantly impact the study's alignment with the specified screening criteria.	2.5	Exclude	Exclude	No tabular data

Multi-view learning-based data proliferator for boosting classification using highly imbalanced classes	Graa O., Rekik I.	2019	yes	The study focuses on clinical applications, specifically autism spectrum disorder (ASD) diagnosis using brain data.	no	The study deals with "multi-view brain representations" and "connectomic datasets" which are not tabular data. These appear to be neuroimaging or network data formats.	yes	The study uses human clinical data, specifically comparing ASD patients with normal controls.	yes	This appears to be an original research article with novel methodology and results.	yes	The study actively creates synthetic data using their proposed MV-LEAP method.	maybe	While the study does generate synthetic data, it's unclear if it uses AI methods like GANs or VAEs. The abstract mentions "manifold learning-based proliferator" which is a different approach.	yes	The study demonstrates direct clinical application in diagnosis of neurological disorders.	yes	The study includes empirical evaluation with clear comparison metrics.	yes	The study reports classification results and compares performance with baseline methods.	Exclude	The study focuses on clinical applications of synthetic data generation for autism spectrum disorder diagnosis, using multi-view brain representations and a novel manifold learning approach. While demonstrating strong clinical relevance and empirical evaluation, the research does not meet key screening criteria due to its non-tabular data format and potentially non-AI synthetic data generation method. These methodological characteristics prevent the study from fully aligning with the specified screening requirements.	2.5	Exclude	Exclude	No tabular data
Online model checking for monitoring surrogate-based respiratory motion tracking in radiation therapy.	Antoni, Sven-Thomas, Rinast, Jonas, Ma, Xintao, Schupp, Sibylle, Schlaefer, Alexander	2016	yes	The study focuses on clinical applications in radiosurgery, specifically respiratory motion compensation and artifact detection during treatment.	no	The study deals with time-series data (breathing motion patterns) rather than tabular data, which is a critical mismatch with the inclusion criteria.	yes	The study uses both synthetic data and real patient data from clinical episodes.	yes	This appears to be an original research article with novel methodology and empirical evaluation.	maybe	While the study uses synthetic data, it's unclear if this was actively created using AI methods or if it was generated using other methods.	maybe	The abstract mentions "synthetic data" but doesn't specify the generation method. There's no mention of AI methods like GANs or VAEs.	yes	The study has direct clinical applications in radiosurgery, specifically for detecting breathing artifacts and ensuring treatment safety.	yes	The study includes clear empirical evaluation with both synthetic and real clinical data, using specific metrics for assessment.	yes	The study reports specific performance metrics (detection rates, percentage improvements over other methods).	Exclude	The study focuses on respiratory motion compensation in radiosurgery using time-series data, which fundamentally conflicts with the requirement for tabular data generation. While demonstrating strong clinical applications and empirical evaluation, the research fails to meet critical screening criteria related to data format and synthetic data generation methodology. Consequently, the paper does not align with the specified inclusion requirements.	2.5	Exclude	Exclude	No tabular data
Pairwise domain adaptation module for CNN-based 2-D/3-D registration	Zheng J., Miao S., Wang Z.J., Liao R.	2018	maybe	The paper focuses on clinical applications (image-guided therapy and 2-D/3-D registration for surgery), but it's primarily a technical methodology paper. While it has clinical applications, the focus is on the technical aspects of domain adaptation.	no	The paper deals with imaging data (2-D x-ray images and 3-D data), not tabular data. This is a clear violation of the inclusion criteria.	maybe	The paper uses clinical data for surgery-related applications, though the extent of human clinical data usage isn't entirely clear from the abstract.	yes	This appears to be an original research article with novel methodology (PDA module).	maybe	The paper discusses synthetic data generation, but it's not clear if they actively create new synthetic data or use existing synthetic datasets. The focus is on domain adaptation between existing synthetic and real data.	maybe	While synthetic data is used, it's not clear if they generate new synthetic data using AI methods. The paper focuses on domain adaptation rather than synthetic data generation.	yes	The paper demonstrates direct clinical applications in image-guided therapy and surgery.	yes	The study includes empirical evaluation with clear testing on clinical applications.	yes	The abstract mentions quantitative evaluation, though specific metrics aren't detailed.	Exclude	The study focuses on technical methodology for image-guided therapy using domain adaptation techniques, primarily involving imaging data rather than tabular data. While demonstrating clinical applications in surgery, the research fails to meet key screening criteria, particularly the requirement for tabular data generation. The paper's technical orientation and imaging-based approach render it unsuitable for the specified screening requirements.	2.5	Exclude	Exclude	No tabular data
Parameter estimation in a whole-brain network model of epilepsy: Comparison of parallel global optimization solvers	Penas D.R., Hashemi M., Jirsa V.K., Banga J.R.	2024	maybe	The paper focuses on clinical applications (epilepsy treatment planning and surgery), but also has significant technical components (optimization algorithms, computational methods). While clinical applications are present, they're not the primary focus.	no	The study deals with spatio-temporal EEG data and brain activity patterns, which are time-series data rather than purely tabular data.	yes	The study uses human clinical data as its basis, specifically focusing on epilepsy patients and their brain activity patterns.	yes	This appears to be an original research article with novel methodology and results.	maybe	The study uses synthetic data generation, but it's unclear if this is done using AI methods or traditional modeling approaches. The focus seems to be on optimization of existing models rather than AI-based data generation.	no	While synthetic data is used ("extensive benchmarking on synthetic data"), there's no mention of AI methods like GANs or VAEs. The generation appears to be through traditional modeling approaches.	yes	The study has direct clinical applications in epilepsy treatment planning and surgery, specifically for "estimation of pathological brain areas in drug-resistant epilepsy."	yes	The study includes empirical evaluation with clear metrics for assessing model performance and uncertainty quantification.	yes	The study reports quantifiable outcomes related to model performance, accuracy, and computational efficiency.	Exclude	The study focuses on epilepsy treatment planning using computational models, but fails key screening criteria by using time-series EEG data instead of tabular data and not employing AI methods for synthetic data generation. Despite strong clinical applications and empirical evaluation, the technical approach and data format disqualify the paper from meeting the specified screening requirements.	2.5	Exclude	Exclude	No tabular data
Perfusion estimation using synthetic MRI-based measurements and a porous media flow model	Lorentzen R.J., Naevdal G., Saevareid O., Hodneland E., Hanson E.A., Munthe-Kaas A.	2023	yes	The study focuses on clinical applications such as perfusion and filtration analysis for diagnosis, follow-up, and therapy, meeting this criterion.	no	The study appears to use imaging data (MRI) rather than tabular data, which fails this criterion.	maybe	The abstract mentions "biological tissue" but doesn't specify if it's human clinical data, so this is unclear.	yes	This appears to be an original research article evaluating a new methodology, meeting this criterion.	maybe	The abstract mentions "synthetic data generated" but doesn't specify if AI methods were used, so this is unclear from the abstract.	maybe	While synthetic data is used, the abstract doesn't specify if AI methods (GANs, VAEs, etc.) were used for generation, so this is unclear.	yes	The study has direct clinical applications (diagnosis, follow-up, and therapy), meeting this criterion.	yes	The study includes empirical evaluation ("evaluated and verified"), meeting this criterion.	maybe	The abstract mentions evaluation and verification but doesn't specify the metrics used, making this unclear.	Exclude	The study focuses on clinical perfusion analysis using MRI data, which immediately disqualifies it from meeting the tabular data requirement. While demonstrating clinical applications and original research, the use of imaging data and unclear synthetic data generation methods prevent full alignment with the specified screening criteria. Multiple uncertainties exist regarding the precise methodology and data characteristics.	2.5	Exclude	Exclude	No tabular data
Phantom Anonymization: Adversarial testing for membership inference risks in anonymized health data	Meurers T., Halilovic M., Otte K., Despraz J., Kaabachi B., Kulynych B., Raisaro J.L., Prasser F.	2025	no	The paper focuses primarily on privacy and methodological aspects (anonymization and membership inference risk) rather than clinical applications. While it's in the medical domain, the focus is on data protection techniques.	yes	The paper explicitly mentions "structured, tabular data" which meets this criterion.	maybe	The abstract mentions "medical research" and "health data" but doesn't explicitly specify whether it uses human clinical data. However, the context suggests it likely does.	yes	This appears to be an original research article with methods and results sections.	no	While the paper mentions synthetic data, it's not focused on creating synthetic data but rather on evaluating anonymization methods. The synthetic data aspect is secondary and used as a comparison tool.	no	The paper doesn't focus on generating synthetic data using AI methods. It mentions synthetization methods but only as a comparison point for their anonymization framework.	no	The paper focuses on privacy protection rather than direct clinical applications like diagnosis or treatment.	maybe	The paper includes empirical evaluation with clear metrics, but these are focused on privacy risk rather than clinical utility.	maybe	The paper reports quantifiable outcomes related to privacy risk assessment, but these aren't clinical outcomes.	Exclude	The study primarily addresses privacy protection and data anonymization techniques in medical research, focusing on methodological approaches rather than direct clinical applications. While the research involves tabular data and includes empirical evaluation, it does not meet key criteria related to synthetic data generation, clinical utility, or direct patient care outcomes. The paper's technical orientation and privacy-centric focus distinguish it from studies with primary clinical objectives.	2.5	Exclude	Exclude	No synthetic data generation
Physics-Informed Transfer Learning to Enhance Sleep Staging	Waters S.H., Clifford G.D.	2024	yes	The study focuses on clinical applications (sleep staging) rather than purely technical aspects, though it does have a strong technical component.	no	The study deals with EEG data, which is time-series data, not tabular data. This is a critical failure of a core criterion.	yes	The study uses human clinical data (EEG recordings) from both in-hospital and wearable sensors.	yes	This appears to be an original research article with clear methodology and results.	yes	The study actively creates synthetic data using LORETA modeling, though it's not strictly AI-based but rather physics-based modeling.	maybe	While the study generates synthetic data, it uses physical modeling (LORETA) rather than AI methods like GANs or VAEs. This is a significant deviation from the criterion.	yes	The study demonstrates direct clinical application in sleep staging, which is a clinical diagnostic task.	yes	The study includes original research with clear empirical evaluation and metrics.	yes	The study reports quantifiable outcomes (accuracy metrics: 80.8% to 81.3% improvement).	Exclude	The study focuses on sleep staging using EEG data, demonstrating clinical application and empirical evaluation with quantifiable outcomes. However, critical screening criteria are not met due to the use of time-series EEG data instead of tabular data and the employment of physical modeling rather than AI-based synthetic data generation methods. These fundamental deviations prevent the study from fully satisfying the specified screening requirements.	2.5	Exclude	Exclude	No tabular data
Predictive modelling of freeway space utilising clinical history, normalised muscle activity, dental occlusion, and mandibular movement analysis	Farook T.H., Haq T.M., Ramees L., Dudley J.	2024	yes	The study focuses on clinical applications (dental freeway space prediction) rather than purely technical aspects.	maybe	The study involves multiple data formats including video tracking, electrognathography, EMG, and 3D scans, which is not purely tabular data generation.	yes	The study uses data from 66 human participants.	yes	This appears to be an original research article with empirical evaluation.	yes	The study actively creates synthetic data for training machine learning models.	yes	The study uses AI methods (including neural networks) to generate synthetic data, though the specific generation method isn't explicitly stated.	yes	The study has direct clinical applications in dental assessment and potentially treatment planning.	yes	The study includes original research with clear metrics and empirical evaluation of synthetic data's utility.	yes	The study reports clear quantifiable outcomes (test error measurements in mm).	Exclude	The study demonstrates strong clinical application in dental freeway space prediction using synthetic data and multiple machine learning models, with clear quantifiable outcomes and empirical evaluation. However, the research fails to meet the critical screening criterion of purely tabular data generation, as it incorporates diverse data formats including video tracking, EMG, and 3D scans. Despite meeting most other criteria, this fundamental deviation from the required data format significantly reduces the study's alignment with the screening requirements.	2.5	Include	Exclude	No tabular data
Privacy enhancing and generalizable deep learning with synthetic data for mediastinal neoplasm diagnosis	Zhou Z., Guo Y., Tang R., Liang H., He J., Xu F.	2024	maybe	The paper focuses on privacy preservation in medical imaging, which is primarily a technical/privacy aspect rather than a direct clinical application. While it mentions precision medicine, the primary focus appears to be on the privacy-preserving technique.	no	The study explicitly focuses on medical images with annotations, not tabular data.	yes	The study uses medical images of mediastinal neoplasms, which implies human clinical data.	yes	Based on the abstract, this appears to be an original research article presenting a new technique (DiffGuard).	yes	The study does involve active creation of synthetic data using AI (DiffGuard technique).	maybe	While the study does use AI methods for synthetic data generation, it's specifically for images rather than tabular data.	maybe	While the study mentions precision medicine, the primary focus is on privacy preservation rather than direct clinical applications like diagnosis or treatment planning.	yes	The study includes empirical evaluation with clear metrics and multi-center evaluation.	yes	The abstract mentions accuracy and generalizability metrics for segmentation and classification tasks.	Exclude	The study focuses on generating synthetic medical images for privacy preservation, which fundamentally conflicts with the requirement for tabular data generation. While demonstrating innovative AI techniques for medical data, the research primarily addresses privacy and technical aspects rather than direct clinical applications. The core methodology of image-based synthetic data creation disqualifies the study from meeting the specified screening criteria.	2.5	Exclude	Exclude	No tabular data
Privacy preserving synthetic respiratory sounds for class incremental learning	Shevchyk A., Hu R., Thandiackal K., Heizmann M., Brunschwiler T.	2022	maybe	The paper focuses on clinical applications (respiratory sound classification for chronic conditions like Asthma, COPD, COVID-19) but also has a strong technical focus on privacy preservation. While clinical applications are present, they're not the primary focus.	no	The study deals with audio data (respiratory sounds) rather than tabular data, which is a clear violation of the criterion requiring only tabular data generation.	yes	The study uses human clinical data (respiratory sounds from patients), meeting this criterion.	yes	This appears to be an original research article with novel methodology and empirical results.	yes	The study does involve active creation of synthetic data using AI (specifically GANs), meeting this criterion.	yes	The study uses AI methods (GANs) for synthetic data generation, meeting this criterion.	maybe	While the study has clinical relevance (respiratory sound classification for disease monitoring), the primary focus is on the privacy-preserving synthetic data generation method rather than direct clinical applications.	yes	The study includes original research with clear empirical evaluation and metrics, meeting this criterion.	yes	The study reports quantifiable outcomes (90% vs 85% accuracy for classifiers), meeting this criterion.	Exclude	The study involves synthetic data generation using AI for respiratory sound classification, but critically fails to meet the tabular data format requirement by using audio data instead. While demonstrating clinical relevance and empirical evaluation, the technical focus on privacy preservation and non-tabular data format disqualifies the paper from meeting the core screening criteria.	2.5	Exclude	Exclude	No tabular data
Privacy Risk Assessment for Synthetic Longitudinal Health Data	Schneider J., Walter M., Otte K., Meurers T., Perrar I., Nothlings U., Adams T., Frohlich H., Prasser F., Fluck J., Kuhnel L.	2024	no	The paper focuses primarily on privacy and methodological aspects of synthetic data generation, rather than clinical applications. While it uses epidemiological data, the main focus is on privacy risk assessment.	yes	The study appears to work with tabular data from the DONALD cohort study, which includes time and age trends.	yes	The study uses data from the DONALD cohort study with 1312 human participants.	yes	This appears to be an original research article with methodology and results.	yes	The abstract mentions "synthetic data generation" and "synthesization," suggesting active creation of synthetic data.	maybe	While synthetic data generation is used, the specific AI methods (GANs, VAEs, etc.) are not explicitly mentioned. However, it does reference "deep learning-based approaches."	no	The paper does not demonstrate direct clinical applications. Instead, it focuses on privacy risk assessment and evaluation of synthetic data.	no	The study includes empirical evaluation, but it's focused on privacy risks rather than clinical utility.	no	While the study reports "privacy scores," these are not clinical outcomes but rather privacy-related metrics.	Exclude	The study primarily focuses on privacy risk assessment of synthetic data generation, lacking direct clinical applications or quantifiable clinical outcomes. While the research involves human clinical data and synthetic data generation, its methodological approach centers on privacy evaluation rather than clinical utility. Consequently, the paper does not meet key screening criteria related to clinical applications and empirical clinical assessment.	2.5	Exclude	Exclude	No direct clinical application

Radiomic and Clinical Model in the Prognostic Evaluation of Adenoid Cystic Carcinoma of the Head and Neck	Rondi P., Tomasoni M., Cunha B., Rampinelli V., Bossi P., Guerini A., Lombardi D., Borghesi A., Magrini S.M., Buglione M., Mattavelli D., Piazza C., Vezzoli M., Farina D., Ravanelli M.	2024	yes	The study focuses on clinical applications, specifically predicting Relapse-Free Survival in Adenoid Cystic Carcinoma patients, which is directly clinical rather than technical.	no	The study primarily uses radiomic features from MRI data, which is imaging data, not tabular data. While clinical features are included, the core data format is imaging-based.	yes	The study uses real patient data from AdCC patients who underwent surgery and radiotherapy.	yes	This is clearly an original research article with empirical analysis and results.	yes	The study mentions "synthetic data augmentation" was applied, suggesting active creation of synthetic data.	maybe	While synthetic data augmentation is mentioned, the abstract doesn't specify the AI methods used (GANs, VAEs, etc.), creating some uncertainty.	yes	The study demonstrates direct clinical application in predicting patient relapse-free survival and treatment planning.	yes	The study includes clear empirical evaluation with specific metrics and validation on real data.	yes	The study reports clear quantifiable outcomes including C-index scores and Brier scores for predictive performance.	Exclude	The study focuses on clinical applications of predicting patient relapse-free survival using radiomic and clinical features, but primarily uses imaging data rather than tabular data. While demonstrating strong clinical relevance and empirical evaluation, the imaging-based data format and lack of detailed synthetic data generation methods create significant screening challenges. The research provides valuable insights into Adenoid Cystic Carcinoma patient management but does not fully align with the specified screening criteria.	2.5	Exclude	Exclude	No tabular data
Redundancy in electronic health record corpora: analysis, impact on text mining performance and mitigation strategies	Cohen R., Elhadad M., Elhadad N.	2013	maybe	The paper focuses on text mining and data processing of EHR data, which is more methodological than clinical. While it has healthcare applications, it's primarily about data processing techniques rather than direct clinical applications.	no	The study deals with free-text patient notes, which is not tabular data. The focus is on text mining and natural language processing.	yes	The study uses real EHR data from human patients.	yes	This appears to be an original research article with empirical evaluation and novel findings.	no	The study does not involve creating synthetic data using AI. While it mentions "synthetic data with controlled levels of redundancy," this appears to be for testing purposes rather than the main focus.	no	The paper does not involve generating synthetic tabular data using AI methods. The synthetic data mentioned is for testing redundancy levels in text data.	maybe	While the study has potential clinical applications in phenotype extraction and disease modeling, it focuses more on the methodological aspects of handling EHR text data rather than direct clinical applications.	yes	The study includes original research with clear empirical evaluation and metrics.	yes	The study reports quantifiable outcomes related to text mining performance and redundancy levels.	Exclude	The study focuses on text mining of Electronic Health Record data, primarily addressing methodological aspects of data processing rather than direct clinical applications. While the research involves human clinical data and includes empirical evaluation, it does not meet key criteria related to synthetic tabular data generation or AI-driven data creation. The paper's emphasis on text analysis and redundancy mitigation places it outside the specified screening requirements.	2.5	Exclude	Exclude	No tabular data
Robust microbiota-based diagnostics for inflammatory bowel disease	Eck A., De Groot E.F.J., De Meij T.G.J., Welling M., Savelkoul P.H.M., Budding A.E.	2017	yes	The study focuses on clinical applications, specifically developing a diagnostic test for IBD using gut microbiota. This is clearly a clinical application rather than purely technical work.	yes	The study appears to work with tabular data from microbiota profiling, though the exact format isn't explicitly stated. However, there's no mention of imaging or other non-tabular formats.	yes	The study uses real clinical data from 91 pediatric IBD patients and 58 healthy children.	yes	This appears to be an original research article with empirical evaluation and novel findings.	no	While the study uses synthetic data, it appears to use "a large synthetic data set" rather than actively creating synthetic data using AI. The focus seems to be on using existing synthetic data for simulation.	no	The abstract doesn't mention using AI methods (GANs, VAEs, etc.) for generating synthetic data. The synthetic data appears to be used for simulation rather than being the primary focus of the study.	yes	The study demonstrates direct clinical applications, specifically focusing on developing a diagnostic test for IBD.	yes	The study includes original research with clear empirical evaluation, including validation across different laboratories and platforms.	yes	The study reports quantifiable outcomes in terms of predictive accuracy and classifier performance.	Exclude	The study demonstrates strong clinical relevance with human patient data and quantifiable outcomes in developing an IBD diagnostic test, but fails to meet key criteria related to AI-driven synthetic data generation. While the research involves synthetic data simulation and empirical validation, it does not actively create synthetic data using AI methods, which disqualifies it from meeting the systematic review's core requirements.	2.5	Exclude	Exclude	No synthetic data generation
SDRG-Net: Secure DR grading network for Real-Time decision support in IoMT environments	Poranki V.K.R., Srinivasarao B.	2025	maybe	The paper focuses on clinical applications (diabetic retinopathy detection and grading), but also has significant technical components (image encryption, feature extraction). However, the clinical application is primary.	no	The study appears to focus on image data (retinal images) rather than tabular data, which is a clear violation of the criterion requiring only tabular data.	yes	The study uses real clinical data from EyePACS and Messidor datasets, which are human clinical datasets.	yes	This appears to be an original research article presenting a new network (SDRG-Net) with empirical evaluation.	maybe	The paper mentions using "Minority Oversampling by Synthetic Data (MOSD)" but it's unclear if this involves active AI-based creation of synthetic data or just oversampling existing data.	maybe	While synthetic data is mentioned (MOSD), the abstract doesn't specify if AI methods like GANs or VAEs are used for generation. The focus seems to be more on the classification network.	yes	The study has direct clinical applications in diabetic retinopathy detection and grading.	yes	The study includes empirical evaluation using established datasets with clear metrics.	yes	The abstract mentions evaluation of classification accuracy and reliability, indicating quantifiable outcomes.	Exclude	The study focuses on diabetic retinopathy detection using image data, which immediately disqualifies it from meeting the tabular data requirement. While demonstrating strong clinical applications and empirical evaluation, the research primarily involves image processing and machine learning techniques rather than synthetic tabular data generation. The technical approach and data format fundamentally diverge from the specified screening criteria.	2.5	Exclude	Exclude	No tabular data
Simulating Accelerometer Signals of Parkinson's Gait Using Generative Adversarial Networks.	Hadley, Aaron J, Pulliam, Christopher L	2024	maybe	The study focuses more on the technical aspects of data generation and augmentation rather than purely clinical applications.	no	The study focuses on accelerometry data, which is time-series data, not tabular data as required by the criteria.	yes	The study uses real-world data from individuals with Parkinson's disease.	yes	The study appears to be an original research article with preliminary results.	yes	The study actively creates synthetic data using GANs.	maybe	While the study uses AI (GANs) for synthetic data generation, it is for time-series data rather than tabular data.	yes	The study aims to improve detection of freezing of gait in Parkinson's disease patients.	maybe	The abstract mentions "preliminary results" but doesn't provide clear metrics for assessing clinical utility.	maybe	While the abstract mentions "accuracy of freezing of gait detection algorithms," it states these results are future work, not yet reported.	Exclude	The study involves synthetic data generation for Parkinson's disease using accelerometry data, which fails to meet the tabular data requirement. While demonstrating clinical relevance and original research, the time-series data format and incomplete empirical evaluation prevent full alignment with the screening criteria. The technical approach and preliminary nature of the research further limit its compliance with the specified screening requirements.	2.5	Exclude	Exclude	No tabular data
Stress State Classification Based on Deep Neural Network and Electrodermal Activity Modeling	Vasile F., Vizziello A., Brondino N., Savazzi P.	2023	maybe	The paper focuses on clinical applications (monitoring autistic patients' emotional states and preventing aggression), but it's primarily a technical/methodological paper about signal classification.	no	The study deals with EDA (Electrodermal Activity) signals, which are time-series data, not tabular data.	yes	The study uses human clinical data (EDA signals from autistic patients).	yes	This appears to be an original research article with novel methodology and results.	yes	The paper does involve active creation of synthetic data using AI (specifically for training a deep neural network).	maybe	While the paper does generate synthetic data using AI methods, it's for EDA signals rather than tabular data.	yes	The study has direct clinical applications (monitoring emotional states and preventing aggression in autistic patients).	yes	The study includes empirical evaluation with clear metrics for assessing the synthetic data's utility.	yes	The study reports quantifiable outcomes (96% accuracy on synthetic data, 84% on experimental sequences).	Exclude	The study focuses on synthetic data generation for EDA signal classification in autistic patients, demonstrating clinical applications and quantifiable outcomes. However, the research primarily involves time-series data rather than tabular data, which critically disqualifies it from meeting the systematic review's core screening criteria. Despite strong methodological and clinical merits, the fundamental data format mismatch prevents inclusion.	2.5	Exclude	Exclude	No tabular data
Synthetic Data-Driven Approaches for Chinese Medical Abstract Sentence Classification: Computational Study.	Li, Jiajia, Wang, Zikai, Yu, Longxuan, Liu, Hui, Song, Haitao	2025	maybe	The paper focuses on text classification of medical abstracts, which is more of a methodological/technical application rather than a direct clinical application. While it has medical relevance, it's primarily about improving literature search and classification.	no	The study deals with text data (medical abstracts) rather than tabular data.	no	The study uses medical abstracts, but these are not direct clinical data or patient-related healthcare information.	yes	This is clearly an original research article with empirical evaluation and novel methodology.	maybe	The study does use AI (GPT-3.5) to generate synthetic data, but for text rather than tabular data.	no	While the study does generate synthetic data using AI (GPT-3.5), it's for text classification rather than tabular data generation.	no	The application is focused on improving literature search and classification rather than direct clinical applications like diagnosis or treatment planning.	yes	The study includes thorough empirical evaluation with clear metrics and ablation studies.	yes	The study reports clear quantifiable outcomes (accuracy and F1-scores for classification).	Exclude	The study focuses on text classification of medical abstracts using AI-generated synthetic data, which diverges from the required tabular clinical data format. Despite robust empirical methodology and quantifiable outcomes, the research fails to meet key screening criteria related to direct clinical applications, patient data, and tabular data generation. Consequently, the study does not align with the specified screening requirements for clinical synthetic data research.	2.5	Exclude	Exclude	No tabular data
Synthetic PPG generation from haemodynamic model with baroreflex autoregulation: a Digital twin of cardiovascular system.	Mazumder, Oishee, Roy, Dibyendu, Bhattacharya, Sakyajit, Sinha, Aniruddha, Pal, Arpan	2019	maybe	The paper focuses on generating synthetic PPG signals for cardiovascular applications, which is clinical in nature. However, it appears to be more focused on the technical aspects of data generation rather than direct clinical applications.	no	The study focuses on time-series physiological signals (PPG, ECG, PCG), not tabular data.	yes	The study uses Physionet data for validation, which is human clinical data.	yes	This appears to be an original research article with novel methodology and validation.	yes	The study involves active creation of synthetic data using a digital twin platform, which is a form of AI/ML-based generation.	maybe	While the study does generate synthetic data, it uses a digital twin platform rather than traditional AI methods like GANs or VAEs.	maybe	While the study mentions CAD classification, it's not clear if this is a direct clinical application or just a validation metric. The focus seems more methodological.	maybe	The study includes empirical evaluation through clustering validation, though the metrics aren't extensively detailed in the abstract.	maybe	The abstract mentions clustering performance for CAD vs non-CAD classification, but specific metrics aren't detailed.	Exclude	The study focuses on generating synthetic physiological signals using a digital twin platform, which primarily involves time-series data rather than tabular data, thus failing a key screening criterion. While the research demonstrates original data creation and uses human clinical data, its methodological approach and focus on signal generation diverge from the specified screening requirements. The technical nature of the study and its emphasis on signal generation techniques further limit its alignment with the intended clinical application criteria.	2.5	Exclude	Exclude	No tabular data
Synthetic PPG Signal Generation to Improve Coronary Artery Disease Classification: Study With Physical Model of Cardiovascular System	Mazumder O., Banerjee R., Roy D., Bhattacharya S., Ghose A., Sinha A.	2022	maybe	The paper focuses on clinical applications (cardiac disease classification) rather than purely technical aspects, though it has a strong methodological component.	no	The study focuses on PPG time-series data, which is explicitly excluded in the criteria as it's not tabular data.	yes	The study uses real PPG data from human patients as its basis, though it's unclear how much real data is used.	yes	This appears to be an original research article with novel methodology and results.	yes	The study actively creates synthetic data using both physical modeling and AI (VAE), meeting this criterion.	yes	The study uses AI methods (VAE) for synthetic data generation, though it combines this with physical modeling.	yes	The study demonstrates direct clinical application in cardiac disease classification.	yes	The study includes original research with clear empirical evaluation and metrics.	yes	The study reports clear quantifiable outcomes (sensitivity and specificity metrics).	Exclude	The study presents a novel approach to synthetic data generation for cardiac disease classification using a combination of physical modeling and AI techniques. While demonstrating strong clinical applications and empirical evaluation, the research focuses on time-series PPG data, which explicitly violates the screening criteria for tabular data generation. Consequently, the study does not meet the fundamental data format requirement despite its methodological sophistication.	2.5	Exclude	Exclude	No tabular data
The hidden waves in the ECG uncovered revealing a sound automated interpretation method	Rueda C., Larriba Y., Lamela A.	2021	maybe	The paper focuses on clinical applications (cardiac rhythm analysis and diagnosis) rather than purely technical aspects, but it appears to be heavily focused on the technical methodology.	no	The study deals with time-series data (ECG signals) rather than tabular data, which is a clear violation of the criterion.	maybe	The study uses real cardiac rhythm data, though it's not explicitly stated whether it's from human patients. However, the context suggests human clinical data.	yes	This appears to be an original research article presenting a novel approach.	maybe	While the paper mentions "generation of synthetic data," it's unclear if this is done using AI methods or if it's just part of the overall methodology. The focus seems to be on the decomposition method rather than synthetic data generation.	maybe	The abstract doesn't specify the method used for synthetic data generation. While it mentions "generation of synthetic data," there's no indication of AI methods being used.	yes	The paper demonstrates direct clinical applications, specifically mentioning "automatic diagnosis of heart anomalies."	yes	The study includes empirical evaluation using both simulated and real data, with clear validation of its capabilities.	yes	The abstract mentions validation using numerical methods and comparison with competitors, suggesting quantifiable outcomes.	Exclude	The study presents a novel cardiac rhythm analysis method with potential clinical applications, but critically fails to meet the tabular data format requirement by focusing on time-series ECG signals. While demonstrating empirical validation and clinical relevance, the paper's methodology and data type do not align with the specified screening criteria, particularly the fundamental requirement of tabular data generation.	2.5	Exclude	Exclude	No tabular data
Thresholding Gini variable importance with a single-trained random forest: An empirical Bayes approach	Dunne R., Reguant R., Ramarao-Milne P., Szul P., Sng L.M.F., Lundberg M., Twine N.A., Bauer D.C.	2023	no	The paper focuses on a technical method (RFlocalfdr) for feature selection in random forests, rather than on clinical applications. While it mentions health applications, the primary focus appears to be methodological rather than clinical.	yes	The paper discusses tabular data analysis, including high-dimensional datasets like genomics, which meets this criterion.	maybe	While the paper mentions "real-world data in health," it's unclear if this involves human clinical data specifically. The focus seems to be on general feature selection methodology.	yes	This appears to be an original research article presenting a new method (RFlocalfdr).	no	The paper does not involve creating synthetic data using AI. While it mentions using synthetic data, this is for testing their method rather than generating synthetic data as the primary focus.	no	The paper does not generate synthetic data using AI methods. It uses synthetic data as a test case but doesn't create it.	no	The paper doesn't demonstrate direct clinical applications. It's focused on a statistical method for feature selection that could potentially be used in clinical contexts, but this isn't the primary focus.	maybe	The paper includes empirical evaluation, but it's focused on methodological performance rather than clinical utility.	maybe	The paper reports quantifiable outcomes in terms of accuracy and computational performance, but these are methodological metrics rather than clinical outcomes.	Exclude	The study presents a methodological approach to feature selection using random forests, with limited direct clinical application and no focus on synthetic data generation. While the research involves health-related data and demonstrates computational performance, it primarily addresses statistical methodology rather than clinical utility or synthetic data creation. The paper's technical nature and lack of explicit clinical outcomes suggest it does not fully align with the specified screening criteria.	2.5	Exclude	Exclude	No synthetic data generation

Training artificial neural networks directly on the concordance index for censored data using genetic algorithms	Kalderstam J., Eden P., Bendahl P.-O., Strand C., Ferno M., Ohlsson M.	2013	yes	The study focuses on clinical applications, specifically prognostic modeling for breast cancer patients.	yes	The study uses tabular data (patient data with covariates) and does not involve imaging or other non-tabular formats.	yes	The study uses real human clinical data from 4042 breast cancer patients across five medical studies.	yes	This appears to be an original research article with novel methodology and empirical evaluation.	no	The study does not create synthetic data using AI. While it uses synthetic data, this is for validation purposes only, and the main analysis is on real patient data.	no	The study does not generate synthetic data using AI methods. It uses pre-existing synthetic data for comparison purposes.	yes	The study demonstrates direct clinical application in prognostic modeling for breast cancer patients.	yes	The study includes original research with clear empirical evaluation and metrics.	yes	The study reports quantifiable outcomes using the concordance index (c-index) and false positive rates.	Exclude	The study demonstrates strong clinical relevance in breast cancer prognostic modeling using neural networks, with robust empirical evaluation and quantifiable outcomes. However, the research does not involve AI-generated synthetic data creation, which disqualifies it from meeting key screening criteria related to synthetic data generation. Despite comprehensive clinical analysis and original research methodology, the lack of AI-driven synthetic data production prevents full alignment with the specified screening requirements.	2.5	Exclude	Exclude	No synthetic data generation
Use of semi-synthetic data for catheter segmentation improvement	Danilov V.V., Kolpashchikov D.Y., Gerget O.M., Laptev N.V., Proutski A., Hernandez Gomez L.A., Alvarez F., Ledesma-Carbayo M.J.	2023	maybe	The paper focuses on medical device segmentation during minimally invasive surgery, which is a clinical application. However, it's primarily focused on the technical aspects of image generation and segmentation.	no	The study deals with image data, not tabular data. This is a clear violation of the criterion as it specifically mentions "semi-synthetic images" and image segmentation.	yes	The study uses real medical images of heart cavities, which implies human clinical data.	yes	This appears to be an original research article with novel methodology and empirical results.	yes	The study does involve active creation of synthetic data using AI, specifically generating semi-synthetic images of heart cavities with artificial catheters.	maybe	While the study does generate synthetic data, it's focused on image generation rather than tabular data generation. The method appears to use forward kinematics rather than typical AI methods like GANs or VAEs.	yes	The application is related to medical device segmentation during surgery, which has direct clinical applications.	yes	The study includes original research with clear empirical evaluation and metrics for assessing the synthetic data's utility.	yes	The study reports clear quantifiable outcomes, specifically Dice similarity coefficients (92.6% vs 86.5%).	Exclude	The study involves semi-synthetic image generation for medical device segmentation, demonstrating clinical relevance and empirical evaluation with quantifiable outcomes. However, the research focuses on image data rather than tabular data, which critically disqualifies it from meeting the systematic review's core screening criteria. Despite strong methodological rigor and clinical application, the fundamental data format deviation prevents inclusion.	2.5	Exclude	Exclude	No tabular data
Using Synthetic Health Care Data to Leverage Large Language Models for Named Entity Recognition: Development and Validation Study.	Suvalov, Hendrik, Lepson, Mihkel, Kukk, Veronika, Malk, Maria, Ilves, Neeme, Kuulmets, Hele-Andra, Kolde, Raivo	2025	maybe	The paper focuses on clinical applications (named entity recognition in medical records) rather than purely technical aspects, but it is primarily focused on the NLP methodology.	no	The study deals with text data rather than tabular data, as the abstract specifically mentions "medical texts" and "named entity recognition."	yes	The study uses human clinical data (Estonian medical records) as its basis.	yes	This appears to be an original research article with clear methodology and results.	maybe	The study does involve active creation of synthetic data using AI (GPT-2 model), but for text rather than tabular data.	maybe	While the study does use AI methods (GPT-2) for synthetic data generation, it's for text data rather than tabular data.	maybe	While the study has clinical applications (clinical decision support and data mining), it's more focused on the NLP methodology than direct clinical applications.	yes	The study includes original research with clear empirical evaluation and metrics.	yes	The study reports quantifiable outcomes (F1-scores of 0.69 for drug extraction and 0.38 for procedure extraction).	Exclude	The study focuses on named entity recognition in Estonian medical texts using synthetic data generation, but critically fails to meet key screening criteria due to its text-based rather than tabular data approach. While demonstrating original research with quantifiable outcomes and human clinical data, the methodology fundamentally diverges from the required tabular data generation framework. Consequently, the paper does not align sufficiently with the specified screening requirements.	2.5	Exclude	Exclude	No tabular data
Utility of synthetic musculoskeletal gaits for generalizable healthcare applications.	Yamada, Yasunori, Kobayashi, Masatomo, Shinkawa, Kaoru, Bilal, Erhan, Liao, James, Nemoto, Miyuki, Ota, Miho, Nemoto, Kiyotaka, Arai, Tetsuaki	2025	yes	The study focuses on clinical applications of gait analysis for healthcare, rather than purely technical, privacy, methodological or educational aspects.	maybe	The study deals with gait data from sensors, which is time-series data, not tabular data. This is a critical failure of a core criterion.	yes	The study involves human gait analysis across different clinical populations.	yes	Based on the abstract, this appears to be original research with novel methodology and results.	yes	The study actively creates synthetic data using a generative model learned via physics-based simulation.	yes	The study uses AI methods (deep neural networks) to generate synthetic data.	yes	The study demonstrates direct clinical applications in gait analysis for healthcare applications.	yes	The study includes empirical evaluation comparing synthetic-data-trained models with real-data-trained models.	yes	The study reports quantifiable outcomes in terms of gait parameter estimation performance.	Exclude	The study demonstrates strong clinical applications in gait analysis using synthetic data generation, with robust empirical evaluation and quantifiable outcomes across multiple clinical populations. However, the critical use of time-series sensor data instead of tabular data represents a fundamental disqualification from the specified screening criteria. Despite meeting most other requirements, this single criterion failure prevents the study from being included in the review.	2.5	Exclude	Exclude	No tabular data
Utilizing risk-controlling prediction calibration to reduce false alarm rates in epileptic seizure prediction	Segal G., Keidar N., Lotan R.M., Romano Y., Herskovitz M., Yaniv Y.	2023	yes	The study focuses on clinical applications, specifically seizure prediction for epilepsy patients, which is directly related to clinical care and patient outcomes.	no	The study primarily deals with EEG data, which is time-series data, not tabular data. This is a critical mismatch with the inclusion criteria.	yes	The study uses data from 15 human patients with epilepsy.	yes	This appears to be an original research article with novel methodology and empirical evaluation.	yes	The study mentions using synthetic data, though it's not entirely clear if they actively created it or used existing synthetic datasets.	maybe	While synthetic data is mentioned, the abstract doesn't specify the method used for generation (GANs, VAEs, etc.), creating some uncertainty.	yes	The study has direct clinical applications in seizure prediction and patient care.	yes	The study includes clear empirical evaluation with specific metrics and testing on real patient data.	yes	The study reports specific metrics including false alarm rate reduction (92%) and seizure detection performance.	Exclude	The study focuses on epilepsy seizure prediction using EEG data, which fundamentally conflicts with the requirement for tabular data generation. While demonstrating strong clinical relevance and empirical evaluation, the time-series nature of EEG data disqualifies the research from meeting the specified screening criteria. Uncertainty also exists regarding the precise methods of synthetic data generation.	2.5	Exclude	Exclude	No tabular data
VR-Caps: A Virtual Environment for Capsule Endoscopy	Incetan K., Celik I.O., Obeid A., Gokceler G.I., Ozyoruk K.B., Almalioglu Y., Chen R.J., Mahmood F., Gilbert H., Durr N.J., Turan M.	2021	maybe	The paper focuses on clinical applications (capsule endoscopy for diagnosis and treatment of gastrointestinal diseases) but also has significant technical components. While it has clinical relevance, it's primarily a technical/methodological paper about simulation platforms.	no	The paper deals with imaging data (capsule endoscopy images) rather than tabular data.	maybe	The paper mentions using real medical data for validation, but the primary focus is on simulated data. The real data appears to be used only for validation rather than as the basis for the synthetic data generation.	yes	This appears to be an original research article presenting a new simulation platform and its validation.	maybe	The paper describes creating synthetic data through simulation (VR-Caps platform), but this is not AI-generated synthetic data in the traditional sense. It's a physics-based simulation platform.	no	While the paper does generate synthetic data, it uses physics-based simulation rather than AI methods like GANs or VAEs. The synthetic data is used to train AI models, but the generation itself isn't AI-based.	yes	The paper demonstrates clinical applications in diagnosis and treatment of gastrointestinal diseases, including disease detection and segmentation.	yes	The study includes empirical evaluation with clear metrics for assessing the performance of models trained on synthetic data.	yes	The paper reports quantifiable outcomes in terms of model performance on various medical image analysis tasks.	Exclude	The study focuses on a simulation platform for capsule endoscopy, generating synthetic imaging data through physics-based methods rather than AI techniques. While demonstrating clinical applications and empirical validation, the research primarily involves imaging data and technical methodology, which diverges from the specified screening criteria. Consequently, the paper does not fully align with the required parameters for synthetic data generation and tabular data analysis.	2.5	Exclude	Exclude	No tabular data
2D medical image synthesis using transformer based denoising diffusion probabilistic model.	Pan, Shaoyan, Wang, Tonghe, Qiu, Richard L J, Axente, Marian, Chang, Chih-Wei, Peng, Junbo, Patel, Ashish B, Shelton, Joseph, Patel, Sagar A, Roper, Justin, Yang, Xiaofeng	2023	maybe	The paper focuses primarily on technical aspects of image synthesis rather than clinical applications. While it mentions COVID-19 classification, this appears to be a validation exercise rather than a clinical application focus.	maybe	The study focuses on medical imaging data (x-rays, MRI, CT scans) rather than tabular data. This is explicitly clear from the abstract's focus on "medical image synthesis."	yes	The study uses human clinical imaging data (chest x-rays, heart MRI, pelvic CT, abdomen CT).	yes	This appears to be an original research article with novel methodology and empirical evaluation.	yes	The study involves active creation of synthetic data using AI (specifically a diffusion model with Swin-transformer-based network).	maybe	While the study does use AI methods for synthetic data generation, it's for images rather than tabular data.	maybe	While COVID-19 classification is mentioned, it's used as a validation task rather than a direct clinical application. The paper's primary focus is on the technical aspects of image synthesis.	yes	The study includes clear empirical evaluation with both visual assessments and quantitative metrics.	yes	The study reports multiple quantifiable metrics (IS, FID, FDS, DS scores, classification accuracy).	Exclude	The study focuses on medical image synthesis using AI techniques, primarily addressing technical challenges in generating synthetic medical images across different modalities. While demonstrating sophisticated methodology and empirical evaluation, the research fails key screening criteria by concentrating on image data rather than tabular data and lacking direct clinical application focus. The work provides a technical framework for synthetic image generation but does not meet the specific requirements for clinical data and application-oriented research.	1.4	Exclude	Exclude	No tabular data
A cGAN-based network for depth estimation from bronchoscopic images	Guo L., Nahm W.	2024	maybe	The paper focuses on technical aspects (depth estimation and 3D reconstruction) rather than direct clinical applications. While it has potential clinical implications for bronchoscopic navigation, the primary focus is on the technical methodology.	no	The study deals with imaging data (bronchoscopic images and depth maps), not tabular data.	maybe	The study uses both synthetic and real bronchoscopic images, but it's unclear if the real data is from human patients. The mention of "clinical phantoms" suggests some clinical relevance but doesn't confirm human patient data.	yes	Yes, this appears to be an original research article with novel methodology and results.	yes	The study does use AI (cGAN) to create synthetic data.	no	While the study does generate synthetic data using AI (cGAN), it's for images rather than tabular data.	maybe	While the study has potential clinical applications in bronchoscopic navigation, it doesn't demonstrate direct clinical applications in the abstract. The clinical validation is mentioned as future work.	maybe	The study includes empirical evaluation, but the real-world validation is only qualitative, and clinical utility metrics are not clearly presented.	maybe	The study reports accuracy metrics for depth estimation, but these are technical rather than clinical outcomes.	Exclude	The study focuses on technical depth estimation using AI-generated synthetic images, which fundamentally diverges from the required tabular data format and clinical application criteria. While demonstrating innovative methodology in bronchoscopic image processing, the research lacks direct clinical outcomes, human patient data validation, and quantifiable clinical metrics. Consequently, the paper does not align with the specified screening requirements for clinical synthetic data research.	1.4	Exclude	Exclude	No tabular data
A Comparative Analysis of the Novel Conditional Deep Convolutional Neural Network Model, Using Conditional Deep Convolutional Generative Adversarial Network-Generated Synthetic and Augmented Brain Tumor Datasets for Image Classification	Onakpojeruo E.P., Mustapha M.T., Ozsahin D.U., Ozsahin I.	2024	no	The study focuses primarily on technical aspects (GANs, CNN models) and methodological approaches rather than clinical applications. While it mentions "disease prediction and diagnosis," this appears to be a secondary consideration.	no	The study explicitly deals with medical images ("brain tumors," "medical image classification") rather than tabular data.	maybe	While the study deals with brain tumors, it's not explicitly stated whether the original data is from human patients. However, given the clinical context, it's likely human data.	yes	Based on the abstract, this appears to be an original research article with novel methodology and results.	yes	The study actively creates synthetic data using GANs, specifically mentioning "Conditional Deep Convolutional Generative Adversarial Network (DCGAN)."	yes	The study clearly uses AI methods (GANs) for synthetic data generation.	maybe	While the study mentions disease prediction and diagnosis, these appear to be secondary considerations rather than the primary focus of the research.	yes	The study includes empirical evaluation with clear metrics and comparison against other models.	yes	The study reports clear quantifiable outcomes including accuracy, precision, recall, and F1 scores.	Exclude	The study focuses on medical image classification using GANs, which fundamentally conflicts with the requirement for tabular data generation. While demonstrating technical innovation in synthetic data creation and achieving high performance metrics, the research fails to meet critical screening criteria related to data format and clinical application focus. The primary technical orientation and image-based approach disqualify the study from meeting the specified screening requirements.	1.4	Exclude	Exclude	No tabular data
A comparative patient-level prediction study in OMOP CDM: applicative potential and insights from synthetic data.	Ahmadi, Najia, Nguyen, Quang Vu, Sedlmayr, Martin, Wolfien, Markus	2024	maybe	The paper focuses on technical aspects of machine learning implementation in clinical databases rather than direct clinical applications. It's primarily about comparing different technical approaches (ATLAS, PLP, and native R solutions) for implementing ML models.	yes	The study deals with tabular data in the OMOP CDM format, which is a standardized clinical database format.	yes	The study uses human clinical data from standardized clinical databases.	yes	This appears to be an original research article comparing different ML implementation approaches.	no	The paper does not involve creating synthetic data. It focuses on comparing different ML implementation approaches for real clinical data.	no	There is no mention of synthetic data generation or AI methods for data creation.	maybe	While the paper discusses patient-level prediction models, it focuses on the technical implementation rather than demonstrating direct clinical applications.	maybe	The study includes empirical evaluation, but it's focused on technical performance rather than clinical utility.	maybe	The study reports quantifiable outcomes related to performance, execution time, and implementation ease, but these are technical metrics rather than clinical outcomes.	Exclude	The study focuses on technical machine learning implementation approaches for clinical databases, lacking synthetic data generation and direct clinical applications. While using human clinical data and demonstrating empirical evaluation, the research primarily compares technical methodologies rather than addressing synthetic data creation or clinical utility. Consequently, the paper does not meet several key screening criteria related to synthetic data generation and clinical outcomes.	1.4	Exclude	Exclude	No synthetic data generation
A comprehensive survey and comparative analysis of time series data augmentation in medical wearable computing.	Hasan, Md Abid, Li, Frederic, Gouverneur, Philip, Piet, Artur, Grzegorzek, Marcin	2025	maybe	The paper focuses on technical aspects of data augmentation for time series data, rather than direct clinical applications. While it mentions medical applications, the focus is on the methodology of data augmentation itself.	no	The paper explicitly focuses on time series data, which is specifically excluded in the criteria as it's not tabular data.	maybe	The paper uses human clinical data (OPPORTUNITY, HAR, DEAP, BVDB, PMDB databases), but primarily for evaluation of augmentation methods rather than direct clinical applications.	no	This appears to be a survey/review paper ("This paper conducts a comprehensive survey") rather than original research.	no	The paper discusses various data augmentation methods including generative models, but appears to be a survey/review of existing methods rather than creating new synthetic data.	no	While the paper discusses synthetic data generation methods, it's focused on time series data augmentation rather than tabular data generation using AI methods.	no	The paper focuses on data augmentation methodology rather than direct clinical applications like diagnosis or treatment planning.	maybe	While the paper includes empirical evaluation, it's focused on comparing augmentation methods rather than evaluating clinical utility.	maybe	The paper reports performance metrics for different augmentation methods, but these are technical metrics rather than clinical outcomes.	Exclude	The study focuses on time series data augmentation techniques, which deviates from the required tabular data generation criteria. While utilizing human clinical datasets, the research is a methodological survey rather than an original research article with direct clinical applications. Multiple screening criteria are not met, particularly those related to synthetic data generation, clinical utility, and empirical evaluation of AI-driven tabular data creation.	1.4	Exclude	Exclude	Wrong publication type

A data-driven deep learning pipeline for quantitative susceptibility mapping (QSM)	Wang Z., Xia P., Huang F., Wei H., Hui E.S.-K., Mak H.K.-F., Cao P.	2022	maybe	The paper focuses on clinical applications (QSM quantification for Parkinson's disease and small-vessel disease), but it's primarily a technical/methodological paper about improving deep learning QSM quantification. The clinical application is secondary to the technical development.	no	The study deals with imaging data (QSM - Quantitative Susceptibility Mapping), not tabular data. This is a clear violation of the inclusion criteria.	yes	The study uses human clinical data from Parkinson's disease and small-vessel disease cohorts.	yes	This appears to be an original research article with clear methodology and results.	no	While the study uses AI, it's not creating synthetic data for clinical use. Instead, it's using synthetic data for training and validation of a technical method.	no	The study uses synthetic data, but not for the purpose of generating clinical data. The synthetic data is used for training and validation of the QSM method.	maybe	While the study has clinical applications (Parkinson's disease and small-vessel disease), the primary focus is on improving the technical method rather than direct clinical applications.	yes	The study includes thorough empirical evaluation with clear metrics.	yes	The study reports clear quantifiable outcomes (NRMSE, SSIM, biases, sensitivity metrics).	Exclude	The study primarily focuses on technical methodological improvements in QSM quantification using deep learning, with imaging data that does not meet the tabular data generation criteria. While the research involves human clinical data and demonstrates quantifiable outcomes, it fundamentally fails to align with the core screening requirements for synthetic data generation and clinical application focus. The technical nature of the research and its imaging-based approach disqualify it from meeting the systematic review's inclusion criteria.	1.4	Exclude	Exclude	No tabular data
A deep learning approach for synthetic MRI based on two routine sequences and training with synthetic data	Moya-Saez E., Pena-Nogales O., Luis-Garcia R.D., Alberola-Lopez C.	2021	no	The study focuses primarily on technical aspects of MRI image synthesis and parametric mapping, rather than clinical applications. While it mentions "clinical viable times," the focus is on the technical methodology.	no	The study deals with imaging data (MRI), not tabular data. This is explicitly clear from the abstract's focus on MRI parametric maps and weighted images.	maybe	While the study mentions "actual MR brain acquisitions," it primarily uses synthetic data from the BrainWeb tool, which is a simulated brain model.	yes	This appears to be an original research article with clear methodology and results.	yes	The study involves active creation of synthetic data using AI (CNN), specifically creating a synthetic dataset of 120 volumes from the BrainWeb tool.	yes	The study uses AI methods (CNN) for synthetic data generation, though for imaging rather than tabular data.	no	The study doesn't demonstrate direct clinical applications. It focuses on technical aspects of MRI synthesis and parametric mapping without clear clinical utility.	yes	The study includes original research with clear empirical evaluation and metrics.	yes	The study reports quantifiable outcomes (normalized squared error values, correlation values, mean square error values).	Exclude	The study focuses on technical MRI image synthesis using AI, primarily involving imaging data rather than tabular data, which disqualifies it from meeting key screening criteria. While demonstrating sophisticated synthetic data generation and empirical evaluation, the research lacks direct clinical applications and does not align with the required data format and clinical focus. Consequently, the paper does not satisfy the fundamental screening requirements for inclusion.	1.4	Exclude	Exclude	No tabular data
A fully joint bayesian quantitative trait locus mapping of human protein abundance in plasma	Ruffieux H., Carayol J., Popescu R., Harper M.-E., Dent R., Saris W.H.M., Astrup A., Hager J., Davison A.C., Valsesia A.	2020	no	The paper focuses on molecular QTL analysis and proteomic studies, which are primarily technical/methodological rather than clinical applications. While it mentions potential clinical implications for metabolic diseases, these are not the primary focus.	no	The study uses proteomic data from mass-spectrometry and aptamer-based assays, which is not tabular data in the traditional sense. While it may be analyzed in tabular form, the raw data is from specialized biological assays.	yes	Yes, the study uses data from two clinical cohorts with human subjects.	yes	Yes, this appears to be an original research article with novel methodology and analysis.	no	The study does not involve creating synthetic data using AI. While it mentions "synthetic data emulating the real data" for comparison purposes, this is not the main focus and doesn't appear to use AI methods.	no	The paper does not involve AI-based synthetic data generation. The synthetic data mentioned is used for comparison purposes only and appears to be statistically generated rather than AI-generated.	no	While the study has potential clinical implications for metabolic diseases, it does not directly demonstrate clinical applications. The abstract explicitly states "considerations on the clinical role of the pQTLs are beyond the scope of our work."	no	The study includes empirical evaluation, but not specifically for clinical utility of synthetic data.	maybe	The study reports quantifiable outcomes, but these are primarily statistical and methodological rather than clinical metrics.	Exclude	The study focuses on molecular QTL analysis using proteomic data from clinical cohorts, but does not meet key screening criteria related to AI-generated synthetic data, clinical applications, or tabular data generation. While methodologically rigorous and involving human clinical data, the research primarily addresses technical and statistical aspects of genetic analysis rather than direct clinical utility or synthetic data creation.	1.4	Exclude	Exclude	No tabular data
A hybrid analytical-numerical algorithm for determining the neuronal current via electroencephalography	Hashemzadeh P., Fokas A.S., Schonlieb C.B.	2020	no	The paper focuses on technical aspects of EEG data analysis and mathematical modeling rather than clinical applications. While it mentions medical applications like sleep studies and epilepsy diagnosis, these are only mentioned as background context.	no	The study deals with EEG data, which is time-series data, not tabular data.	no	While the paper discusses EEG data from human subjects, it focuses on the mathematical modeling of the data rather than using clinical data for direct healthcare applications.	yes	Yes, this appears to be an original research article presenting new mathematical formulas and approaches.	no	While the paper mentions using synthetic data, it appears to be using it for testing their mathematical model rather than creating synthetic data as the primary focus. The synthetic data creation method is not specified.	no	The paper does not appear to focus on synthetic data generation using AI methods. It mentions synthetic data only in the context of testing their mathematical model.	no	The paper does not demonstrate direct clinical applications. It focuses on mathematical modeling of EEG data and current determination.	no	While the paper includes some empirical evaluation ("performs well for synthetic data"), this is not focused on clinical utility.	no	The paper appears to present mathematical formulas and algorithms but does not report clinical outcomes or metrics related to healthcare applications.	Exclude	The study focuses on mathematical modeling of EEG data using technical approaches, which fundamentally diverges from the screening criteria's emphasis on AI-generated synthetic clinical data. Critical requirements such as tabular data format, AI-driven synthetic data generation, and direct clinical applications are not met, rendering the paper outside the systematic review's scope. The research is primarily a technical exploration of EEG signal analysis rather than a clinical data generation study.	1.4	Exclude	Exclude	No tabular data
A Hybrid Model for Psoriasis Subtype Classification: Integrating Multi Transfer Learning and Hard Voting Ensemble Models +	Avci I.A., Zirekgur M., Karakaya B., Demir B.	2025	maybe	The paper focuses on clinical applications (psoriasis diagnosis), but it's primarily a technical/methodological paper about image classification. While it has clinical relevance, the focus is on the technical approach rather than clinical implementation.	no	The study uses image data, not tabular data. This is explicitly stated in the abstract ("930 images") and is a clear violation of this criterion.	yes	Yes, the study uses human clinical data from the Dermatology Clinic of Firat University Hospital.	yes	Yes, this appears to be an original research article with clear methodology and results.	maybe	While the study mentions "synthetic data augmentation," this appears to be a minor component for addressing class imbalance rather than the main focus of creating synthetic data. The primary data is real clinical images.	no	The study does not focus on synthetic data generation. The synthetic data augmentation mentioned is a minor technical detail rather than the main research focus.	yes	Yes, the study demonstrates direct clinical application in psoriasis diagnosis and classification.	yes	Yes, the study includes original research with clear empirical evaluation and metrics.	yes	Yes, the study reports clear quantifiable outcomes (93.14% accuracy, 96.75% precision, F1 score of 91.44%).	Exclude	The study focuses on image-based psoriasis classification using machine learning techniques, which fundamentally conflicts with the requirement for tabular data generation. While demonstrating clinical relevance and empirical rigor, the research primarily involves image processing and technical methodology rather than synthetic data creation. Consequently, the paper fails to meet critical screening criteria related to data format and synthetic data generation.	1.4	Exclude	Exclude	No tabular data
A Large-scale Synthetic Pathological Dataset for Deep Learning-enabled Segmentation of Breast Cancer.	Ding, Kexin, Zhou, Mu, Wang, He, Gevaert, Olivier, Metaxas, Dimitris, Zhang, Shaoting	2023	maybe	The paper focuses on generating synthetic pathological images for computational pathology, which is a clinical application. However, the primary focus appears to be on the technical aspects of generating synthetic data rather than direct clinical applications.	no	The study focuses on image data generation, not tabular data. This clearly fails this criterion as the screening criteria specifically excludes imaging data.	yes	The study uses pathological images, which are derived from human clinical data.	yes	Yes, this appears to be an original research article presenting a new dataset and methodology.	yes	The study involves active creation of synthetic data using AI methods ("applying the off-the-shelf image generator").	maybe	While the study does involve synthetic data generation using AI, it's specifically for images rather than tabular data.	maybe	While the study mentions "downstream data-driven clinical tasks," it doesn't demonstrate direct clinical applications. The focus is on the technical aspects of generating synthetic data.	maybe	The study includes empirical evaluation, but it's focused on model performance rather than clinical utility.	maybe	The abstract mentions "extensive results" but doesn't specify clear clinical metrics. The outcomes appear to be more technical in nature.	Exclude	The study focuses on generating synthetic pathological images using AI, which fundamentally conflicts with the requirement for tabular data generation. While the research demonstrates technical innovation in synthetic data creation, it fails to meet critical screening criteria, particularly the specification of tabular data format. The paper's emphasis on image-based synthetic data generation disqualifies it from the systematic review's scope.	1.4	Exclude	Exclude	No tabular data
A latent code based multi-variable modulation network for susceptibility mapping	Zhou W., Xi J., Bao L.	2023	maybe	The paper focuses on quantitative susceptibility mapping (QSM) for clinical applications, which is a technical imaging method. While it has clinical applications, the primary focus appears to be on the technical aspects of the reconstruction method.	no	The study deals with imaging data (magnetic susceptibility maps) rather than tabular data.	yes	The study uses "in vivo human brain data" and "clinical data."	yes	This appears to be an original research article presenting a new method (LCMnet).	no	While the paper mentions using synthetic data, it appears to be using existing synthetic data rather than actively creating new synthetic data using AI.	no	The paper uses deep learning for reconstruction but doesn't appear to focus on generating synthetic tabular data. Instead, it uses synthetic data as part of validation.	maybe	While the method has clinical applications, the abstract focuses more on the technical aspects of reconstruction rather than direct clinical applications like diagnosis or treatment planning.	yes	The study includes empirical evaluation on multiple datasets, including clinical data.	maybe	The abstract mentions "accurate susceptibility measurement" and "excellent generalization" but doesn't provide specific metrics.	Exclude	The study focuses on a technical imaging method for quantitative susceptibility mapping, primarily using imaging data rather than tabular data, which fundamentally conflicts with the screening criteria. While the research involves human clinical data and demonstrates empirical evaluation, it lacks the core requirements of synthetic tabular data generation and direct clinical application focus. Consequently, the paper does not meet the essential screening criteria for inclusion.	1.4	Exclude	Exclude	No tabular data
A novel method for efficient estimation of brain effective connectivity in EEG.	Khan, Danish M, Yahya, Norashikin, Kamel, Nidal, Faye, Ibrahima	2023	no	The paper focuses on technical aspects (EEG signal processing and connectivity analysis) rather than direct clinical applications. While it mentions potential clinical applications in the conclusion, the primary focus is methodological.	no	The study uses EEG data, which is time-series data, not tabular data. This explicitly fails the criterion requiring only tabular data.	yes	The study uses human EEG data from 46 healthy controls.	yes	This appears to be an original research article with novel methodology and empirical evaluation.	maybe	While the study uses synthetic data, it's not clear if they actively created it using AI methods. The abstract mentions "synthetic and real resting-state EEG" but doesn't specify the generation method.	maybe	The abstract doesn't specify how the synthetic data was generated. While they use synthetic data, there's no mention of AI methods like GANs or VAEs.	no	While the paper mentions potential clinical applications for "diagnosis of mental disorders," the actual study focuses on eye-state classification rather than direct clinical applications.	yes	The study includes original research with clear empirical evaluation and metrics.	yes	The study reports clear quantifiable outcomes (classification accuracy, sensitivity, and specificity).	Exclude	The study primarily focuses on technical EEG signal processing methodology using time-series data, which fundamentally conflicts with the requirement for tabular data generation. While demonstrating empirical research with quantifiable outcomes, the paper lacks direct clinical applications and does not clearly employ AI-based synthetic data generation techniques. Consequently, the research fails to meet several critical screening criteria related to data format, application focus, and synthetic data generation.	1.4	Exclude	Exclude	No tabular data
Abnormal discharge detection using adaptive neuro-fuzzy inference method with probability density-based feature and modified subtractive clustering	Liang G., Liao H., Huang Z., Li X.	2023	yes	The paper focuses on clinical applications (abnormal discharge detection in EEG), but also has significant technical components. However, the clinical application is primary, so this criterion is met.	no	The study deals with EEG data, which is time-series data. This explicitly fails criterion #2 which specifically excludes time-series data like EEGs.	maybe	The study uses EEG data, which is human clinical data, though it's not entirely clear if all data is from human subjects.	yes	Yes, this is an original research article presenting a new method (mSCM-based ANFIS).	no	While the paper mentions using synthetic data, it appears to be using existing synthetic datasets rather than actively creating new synthetic data using AI. The abstract doesn't indicate active creation of synthetic data.	no	The paper doesn't appear to focus on generating synthetic data using AI methods. While it uses synthetic data, it's not clear if it's generating it, and the focus is on detection methods.	yes	Yes, the study demonstrates direct clinical applications in abnormal discharge detection.	yes	Yes, the study includes empirical evaluation with clear metrics and comparisons to other methods.	yes	Yes, the study reports quantifiable outcomes including accuracy (95.13 ± 0.86%) and recall (89.17 ± 3.06%).	Exclude	The study focuses on abnormal discharge detection in EEG data, which fails the screening criteria due to its time-series data format. While demonstrating clinical applications and reporting quantifiable outcomes, the research does not meet key requirements related to synthetic data generation and tabular data analysis. Consequently, the paper does not align with the systematic review's specific screening criteria.	1.4	Exclude	Exclude	No tabular data
Accelerated Synthetic MRI with Deep Learning-Based Reconstruction for Pediatric Neuroimaging.	Kim, E, Cho, H-H, Cho, S H, Park, B, Hong, J, Shin, K M, Hwang, M J, You, S K, Lee, S M	2022	maybe	The paper focuses on clinical applications (pediatric neuroimaging) rather than purely technical aspects, but it is primarily about improving imaging techniques rather than direct clinical decision-making.	no	The study focuses on imaging data (MRI), not tabular data, and therefore fails to meet the criterion for tabular data generation.	yes	The study uses real human clinical data from pediatric patients.	yes	This is clearly an original research article with empirical evaluation, not a review or commentary.	no	The study uses deep learning for reconstruction of existing MRI data, but does not create new synthetic data from scratch. It is more about processing existing data rather than generating new synthetic data.	no	While the study involves synthetic imaging, it is not generating synthetic tabular data using AI methods. The focus is on image reconstruction rather than data generation.	maybe	While the study has clinical relevance, it focuses on improving imaging techniques rather than direct clinical applications like diagnosis or treatment planning.	yes	The study includes original research with clear empirical evaluation and metrics.	yes	The study reports clear quantifiable outcomes including image quality metrics, lesion detectability, and tissue values.	Exclude	The study focuses on pediatric neuroimaging using MRI reconstruction techniques, which fundamentally differs from the required tabular data generation criteria. While demonstrating clinical relevance and empirical research, the paper fails key screening requirements by using imaging data instead of synthetic tabular data and not generating new synthetic data through AI methods. Consequently, the study does not align with the systematic review's core screening criteria.	1.4	Exclude	Exclude	No tabular data
ADDRESSING DATA SCARCITY IN MEDICAL IMAGING: SYNTHETIC DATA GENERATION AND SELF-SUPERVISED LEARNING	Waggener S.C., Cogan T., Tamil L.	2024	no	The paper focuses on technical aspects (VICTRE simulations, self-supervised learning, JEPA architecture) rather than clinical applications. While it mentions breast composition estimation, this appears to be a technical demonstration rather than a clinical application.	no	The paper focuses on medical imaging data, not tabular data. This is explicitly clear from the abstract's focus on "medical imaging data" and "vision transformer model."	maybe	The paper mentions breast composition estimation, suggesting some connection to human clinical data, but it's unclear if they're using real human clinical data as the basis.	yes	This appears to be an original research article presenting new work with VICTRE simulations and modified JEPA architecture.	yes	The paper does involve active creation of synthetic data using AI (VICTRE simulations and self-supervised learning methods).	maybe	While the paper does involve synthetic data generation using AI methods, it's specifically for imaging data, not tabular data.	no	While breast composition estimation could be clinically relevant, the abstract presents this more as a technical demonstration rather than a direct clinical application.	maybe	The paper includes empirical evaluation, but it's focused on technical performance metrics rather than clinical utility.	no	The abstract mentions "evaluations of linear probing performance" but doesn't clearly specify clinical metrics or outcomes.	Exclude	The study focuses on technical approaches to synthetic medical imaging data generation using AI methods, primarily addressing data scarcity challenges through VICTRE simulations and self-supervised learning techniques. Despite involving synthetic data creation, the research lacks direct clinical applications, quantifiable clinical outcomes, and does not use tabular data formats. Consequently, the work appears more oriented toward methodological innovation in medical imaging data generation rather than clinical utility.	1.4	Exclude	Exclude	No tabular data

Addressing the Generalizability of AI in Radiology Using a Novel Data Augmentation Framework with Synthetic Patient Image Data: Proof-of-Concept and External Validation for Classification Tasks in Multiple Sclerosis.	Brugnara, Gianluca, Jayachandran Preetha, Chandrakanth, Deike, Katerina, Haase, Robert, Pinetz, Thomas, Foltyn-Dumitru, Martha, Mahmutoglu, Mustafa A, Wildemann, Brigitte, Diem, Ricarda, Wick, Wolfgang, Radbruch, Alexander, Bendszus, Martin, Meredig, Hagen, Rastogi, Aditya, Vollmuth, Philipp	2024	no	The study focuses on technical aspects of AI model generalizability and data augmentation rather than direct clinical applications. While it's in a clinical context, the primary focus is on improving AI model performance.	no	The study deals with MRI imaging data, not tabular data. This is explicitly clear from the abstract mentioning "MRI data" and "patient image data."	yes	The study uses real patient data from MS patients (669 patients with 3083 examinations).	yes	This appears to be an original research article with empirical evaluation and results.	yes	The study actively creates synthetic data using GANs for data augmentation.	yes	The study uses GANs for synthetic data generation, though for images rather than tabular data.	maybe	While the study involves clinical data (MS lesion detection), it focuses more on improving AI model generalizability than direct clinical applications.	yes	The study includes clear empirical evaluation with specific metrics and statistical testing.	yes	The study reports clear quantifiable outcomes (AUC scores for model performance).	Exclude	The study focuses on technical AI model generalizability using MRI imaging data, which deviates from the required tabular data format and direct clinical application criteria. Despite demonstrating sophisticated synthetic data generation and empirical evaluation, the research fails to meet key screening requirements related to data type and clinical focus. Consequently, the paper does not align with the systematic review's core screening criteria.	1.4	Exclude	Exclude	No tabular data
Advancing synthetic data for dermatology: GAN comparison with multi-metric and expert validation approach	Luschi A., Tognetti L., Cartocci A., Cevenini G., Rubegni P., Iadanza E.	2025	no	The study focuses on generating synthetic images for data augmentation in dermatological image analysis, which is primarily a technical/methodological application rather than a direct clinical application.	no	The study deals with image data (dermatoscopic pictures), not tabular data.	yes	The study uses human clinical data (dermatoscopic images of melanoma lesions from human patients).	yes	This appears to be an original research article with clear methodology and results.	yes	The study actively creates synthetic data using GANs (DCGAN, StyleGAN2, and StyleGAN3-t), not using pre-existing synthetic datasets.	no	While the study does use AI methods (GANs) for synthetic data generation, it generates images rather than tabular data.	maybe	While the ultimate goal is clinical (improving melanoma diagnosis), the study itself focuses on the technical aspects of generating synthetic images rather than direct clinical applications.	yes	The study includes thorough empirical evaluation with both quantitative metrics and expert assessment.	yes	The study reports clear quantifiable outcomes including FID, KID, precision, recall, accuracy, sensitivity, and specificity.	Exclude	The study focuses on generating synthetic dermatoscopic images using GANs, which fundamentally deviates from the screening criteria by dealing with image rather than tabular data. While demonstrating rigorous methodology and empirical evaluation, the research primarily addresses technical aspects of synthetic image generation rather than direct clinical applications. Consequently, the paper fails to meet key screening requirements related to data format and clinical focus.	1.4	Exclude	Exclude	No tabular data
Adversarial robustness improvement for X-ray bone segmentation using synthetic data created from computed tomography scans	Fok W.Y.R., Fieselmann A., Huemmer C., Biniazan R., Beister M., Geiger B., Kappler S., Saalfeld S.	2024	no	The study focuses on technical aspects of image analysis and synthetic data generation for training AI models, rather than direct clinical applications.	no	The study deals with X-ray images and CT volumes, not tabular data. This is explicitly clear from the abstract's focus on "X-ray images" and "3D CT volumes."	yes	The study uses human clinical data (X-ray images and CT volumes) as its basis.	yes	This appears to be an original research article with empirical evaluation.	yes	The study involves active creation of synthetic data using AI, specifically generating synthetic X-ray images from 3D CT volumes.	maybe	While the study does generate synthetic data, it's for images rather than tabular data. The method isn't explicitly stated as using GANs or VAEs.	no	While the study has potential clinical relevance (patient positioning), it focuses on improving AI model training rather than direct clinical applications.	yes	The study includes original research with clear empirical evaluation and metrics.	yes	The study reports quantifiable outcomes (Dice score improvements of 3% to 15%).	Exclude	The study focuses on synthetic X-ray image generation using AI techniques, but fails key screening criteria by working with imaging data instead of tabular data and emphasizing technical model training over direct clinical applications. Despite demonstrating quantifiable outcomes and using human clinical data, the research does not meet the fundamental requirements for inclusion in the systematic review.	1.4	Exclude	Exclude	No tabular data
Amplifying the Effects of Contrast Agents on Magnetic Resonance Images Using a Deep Learning Method Trained on Synthetic Data	Fringuello Mingo A., Colombo Serra S., Macula A., Bella D., La Cava F., Ali M., Papa S., Tedoldi F., Smits M., Bifone A., Valbusa G.	2023	maybe	The study focuses on clinical applications (imaging enhancement for diagnosis) rather than purely technical aspects, but it has a strong technical component in developing the AI method.	no	The study focuses on imaging data (MRI), not tabular data, which is a clear violation of the criterion.	yes	The study uses both animal (murine) and human clinical data, with a large retrospective clinical dataset of 1990 examinations.	yes	This appears to be an original research article with clear methodology and results.	yes	The study does involve active creation of synthetic data using AI, specifically for contrast enhancement in MRI images.	maybe	While the study does generate synthetic data using AI methods, it's for imaging data rather than tabular data.	yes	The study demonstrates direct clinical applications in diagnostic imaging and lesion detection.	yes	The study includes thorough empirical evaluation with clear metrics and validation against ground truth data.	yes	The study reports clear quantifiable outcomes including contrast-to-noise ratio, lesion-to-brain ratio, and sensitivity scores.	Exclude	The study involves synthetic data generation for medical imaging, but critically fails to meet the tabular data requirement, rendering it ineligible for the review. While demonstrating strong clinical applications and empirical evaluation, the focus on MRI imaging data fundamentally disqualifies the research from the specified screening criteria. The technical approach and clinical outcomes are robust, but the data format represents a non-compliant deviation from the review's scope.	1.4	Exclude	Exclude	No tabular data
An adversarial learning approach to generate pressure support ventilation waveforms for asynchrony detection.	Hao, L, Bakkes, T H G F, van Diepen, A, Chennakeshava, N, Bouwman, R A, De Bie Dekker, A J R, Woerlee, P H, Mojoli, F, Mischi, M, Shi, Y, Turco, S	2024	yes	The study focuses on clinical applications, specifically mechanical ventilation and patient-ventilator asynchrony detection in critically ill patients. This is clearly a clinical application rather than purely technical.	maybe	The study deals with waveform data from mechanical ventilation, which is time-series data. This explicitly violates the criterion which requires only tabular data.	yes	The study uses human clinical data from mechanical ventilation in critically ill patients.	yes	This appears to be an original research article with clear methodology and results sections.	yes	The study actively creates synthetic data using VentGAN, an adversarial learning approach, rather than using pre-existing synthetic datasets.	yes	The study uses AI methods (specifically GANs) to generate synthetic data, as evidenced by the VentGAN approach.	yes	The study demonstrates direct clinical application in detecting patient-ventilator asynchrony, which is crucial for patient care.	yes	The study includes empirical evaluation with clear metrics, comparing performance between original simulated data and VentGAN-generated data.	yes	The study reports quantifiable outcomes including classification accuracy and statistical significance (p-values).	Exclude	The study demonstrates a robust approach to generating synthetic clinical data using AI for mechanical ventilation, with strong empirical validation and clear clinical applications. However, the research focuses on time-series waveform data, which explicitly violates the requirement for tabular data generation, rendering the study non-compliant with the systematic review's core screening criteria. Despite meeting most other criteria with high quality, the data format disqualifies the paper from inclusion.	1.4	Exclude	Exclude	No tabular data
Application of generative adversarial networks in image, face reconstruction and medical imaging: challenges and the current progress	Sabnam S., Rajagopal S.	2024	no	The paper is a review paper focusing on technical aspects of GANs and their applications, rather than specific clinical applications. While it mentions medical applications, it's from a technical perspective.	no	The paper focuses on medical imaging and face restoration, which are not tabular data formats. The abstract explicitly mentions "medical imaging" and "face restoration."	no	While medical applications are mentioned, the abstract doesn't specify whether human clinical data is used as the basis.	no	This is explicitly stated to be a "review" paper, not an original research article.	no	While the paper discusses GANs for synthetic data generation, it's a review paper rather than original research creating new synthetic data.	maybe	The paper does discuss AI methods (GANs) for synthetic data generation, but in the context of imaging rather than tabular data.	maybe	While medical applications are mentioned (disease detection, medical image enhancement), these are discussed in a review context rather than demonstrated directly.	no	This is a review paper that analyzes existing work rather than conducting original empirical evaluation.	no	As a review paper, it doesn't report original quantifiable outcomes, though it mentions "evaluation metrics."	Exclude	This review paper on GANs fails multiple screening criteria by focusing on technical aspects of medical imaging rather than clinical applications with tabular data. The study is a comprehensive review of GAN methodologies, not an original research article with empirical evaluation, and does not meet the specified requirements for clinical data generation and quantifiable outcomes.	1.4	Exclude	Exclude	Wrong publication type
Application of synthetic data in the training of artificial intelligence for automated quality assurance in magnetic resonance imaging	Tracey J., Moss L., Ashmore J.	2023	maybe	The study focuses on clinical applications (MRI quality assurance and fault detection) rather than purely technical aspects. However, it's more focused on technical maintenance than direct clinical care.	no	The study uses imaging data (MRI scans), not tabular data.	yes	The study uses real patient images from 497 patients.	yes	This appears to be an original research article with clear methodology and results.	no	The study uses simulated coil failure in real images, but doesn't create entirely synthetic data. It modifies real data rather than generating new synthetic data.	no	While AI is used, it's not for generating synthetic tabular data. The AI is used for fault detection in modified real images.	maybe	While the application is clinical (MRI quality assurance), it's more focused on technical maintenance than direct patient care applications like diagnosis or treatment.	yes	The study includes clear empirical evaluation with specific metrics and comparison against human performance.	yes	The study reports clear quantifiable outcomes (91.74% accuracy for neural network vs 59.99% for radiographers).	Exclude	The study focuses on MRI scanner fault detection using neural networks, but fails key screening criteria by using imaging data instead of tabular data and not generating synthetic data through AI methods. While demonstrating clinical relevance and empirical evaluation, the research does not align with the specified requirements for synthetic data generation and data format.	1.4	Exclude	Exclude	No tabular data
Assessing the Efficacy of Synthetic Optic Disc Images for Detecting Glaucomatous Optic Neuropathy Using Deep Learning.	Chaurasia, Abadh K, MacGregor, Stuart, Craig, Jamie E, Mackey, David A, Hewitt, Alex W	2024	no	The study focuses on technical aspects of image generation and classification rather than direct clinical applications. While it's in a clinical domain, the primary focus is on the AI methodology.	no	The study deals with imaging data (fundus images), not tabular data. This is explicitly clear from the abstract's focus on optic disc images.	yes	The study uses human clinical data (fundus images from glaucomatous and healthy eyes).	yes	This appears to be an original research article with clear methodology and results.	yes	The study actively creates synthetic data using DCGANs, which is clearly described in the methods section.	no	While the study does use AI (DCGANs) for synthetic data generation, it's for images rather than tabular data.	maybe	While the study is about glaucoma detection, which is a clinical application, the focus is more on the technical aspects of improving model performance through synthetic data rather than direct clinical utility.	yes	The study includes thorough empirical evaluation with clear metrics and validation on external datasets.	yes	The study reports clear quantifiable outcomes including ROC curves and classification metrics.	Exclude	The study focuses on generating synthetic fundus images for glaucoma detection using deep learning techniques, which fundamentally deviates from the screening criteria's requirement for tabular data generation. While demonstrating sophisticated AI methodology and clinical relevance, the research primarily addresses technical image generation and classification rather than direct clinical applications with tabular data. Consequently, the study fails to meet key screening requirements despite its scientific merit.	1.4	Exclude	Exclude	No tabular data
Assessment of Deep Generative Models for High-Resolution Synthetic Retinal Image Generation of Age-Related Macular Degeneration	Burlina P.M., Joshi N., Pacheco K.D., Liu T.Y.A., Bressler N.M.	2024	maybe	The study focuses on generating synthetic images for training purposes and medical education, rather than direct clinical applications. While it has clinical relevance, it's primarily a technical/methodological study.	maybe	The study deals with image data (fundus images), not tabular data. This is explicitly clear from the abstract which focuses on "color fundus images."	yes	The study uses human clinical data from the Age-Related Eye Disease Study (AREDS) with 4,613 study participants.	yes	This appears to be an original research article with clear methodology and results.	yes	The study actively creates synthetic data using GANs, as evidenced by "Generative adversarial networks were trained on 133 821 color fundus images."	yes	The study uses AI methods (specifically GANs) for synthetic data generation, though for images rather than tabular data.	maybe	While the study has clinical relevance, it doesn't demonstrate direct clinical applications. Instead, it focuses on generating synthetic training data and evaluating its quality.	yes	The study includes thorough empirical evaluation with clear metrics comparing performance on real vs. synthetic images.	yes	The study reports clear quantifiable outcomes including diagnostic accuracy percentages and AUC values for both real and synthetic images.	Exclude	The study focuses on generating synthetic fundus images using generative adversarial networks, which fundamentally deviates from the screening criteria by using image data instead of tabular data. While demonstrating sophisticated AI techniques for medical image synthesis, the research fails to meet key requirements around clinical application and data format. The methodology, though rigorous, does not align with the specified screening parameters for synthetic data generation.	1.4	Exclude	Exclude	No tabular data
Assessment of the invariance and discriminant power of morphological features under geometric transformations for breast tumor classification	Gomez-Flores W., Hernandez-Lopez J.	2024	maybe	The paper focuses on clinical applications (breast tumor classification) rather than purely technical aspects, but it is primarily focused on feature extraction and classification methodology.	no	The study uses imaging data (ultrasound and mammography) rather than tabular data.	yes	The study uses real human clinical data (breast ultrasound images and mammographies).	yes	This is an original research article with clear methodology and results.	no	The study uses "synthetic data augmentation" but this appears to be simple geometric transformations (rotation and scaling) rather than AI-generated synthetic data.	no	The synthetic data generation mentioned is not using AI methods like GANs or VAEs, but rather basic geometric transformations.	yes	The study demonstrates direct clinical application in breast tumor classification for diagnosis.	yes	The study includes original research with clear empirical evaluation and metrics.	yes	The study reports clear quantifiable outcomes including AUC, accuracy, sensitivity, and specificity.	Exclude	The study focuses on breast tumor classification using imaging data, which fundamentally conflicts with the requirement for tabular data generation. While demonstrating strong clinical application and empirical evaluation, the research fails key screening criteria related to synthetic data generation and data format. Consequently, the paper does not meet the essential requirements for inclusion in the targeted research domain.	1.4	Exclude	Exclude	No tabular data
Augmenting a spine CT scans dataset using VAEs, GANs, and transfer learning for improved detection of vertebral compression fractures	El Kojok Z., Al Khansa H., Trad F., Chehab A.	2023	no	The study focuses on technical aspects of image generation and classification rather than direct clinical applications. While it has clinical relevance, the primary focus is on the technical methodology.	no	The study deals with medical imaging (CT scans) rather than tabular data. This is explicitly stated throughout the abstract.	yes	The study uses real chest CT scans from AUBMC, which are human clinical data.	yes	This appears to be an original research article with novel methodology and results.	yes	The study actively creates synthetic data using AI methods (DCGANs, VAEs, and VAE-GAN models).	no	While the study does use AI methods for synthetic data generation, it's for images rather than tabular data.	maybe	While the study aims to improve detection of vertebral compression fractures, which is a clinical application, the focus is more on the technical aspects of image generation and classification.	yes	The study includes thorough empirical evaluation with clear metrics (FID, classification accuracy).	yes	The study reports clear quantifiable outcomes, including classification accuracy improvements from 73% to 89%.	Exclude	The study focuses on medical image generation using AI techniques, primarily addressing technical challenges in synthetic data creation for vertebral compression fracture detection. While demonstrating clinical relevance and empirical evaluation, the research fundamentally fails key screening criteria by working with imaging data rather than tabular data. The methodology emphasizes technical innovation in AI-generated medical images over direct clinical application.	1.4	Exclude	Exclude	No tabular data

Automated cooling tower detection through deep learning for Legionnaires' disease outbreak investigations: a model development and validation study	Wong K.K., Segura T., Mein G., Lu J., Hannapel E.J., Kunz J.M., Ritter T., Smith J.C., Todeschini A., Nugen F., Edens C.	2025	maybe	The study focuses on clinical/public health applications (Legionnaires' disease outbreak investigation) rather than purely technical aspects. However, it's primarily an epidemiological surveillance tool rather than a direct clinical application.	no	The study uses aerial imagery data, not tabular data. This clearly fails this criterion as it deals with image-based data rather than tabular data generation.	no	The study uses real-world data but focuses on environmental features (cooling towers) rather than direct human clinical data or patient information.	yes	This is clearly an original research article with empirical evaluation and results.	maybe	While the study mentions "synthetic data" augmentation, it's unclear if this refers to AI-generated synthetic data or other forms of data augmentation. The focus appears to be on the computer vision model rather than synthetic data generation.	maybe	The abstract doesn't specify the method used for synthetic data generation. While it mentions "synthetic data," it doesn't indicate whether this was generated using AI methods like GANs or VAEs.	maybe	While the study has public health applications, it's not directly focused on clinical applications like diagnosis, prognosis, or treatment planning. It's more about epidemiological surveillance.	yes	The study includes thorough empirical evaluation with clear metrics and testing across multiple cities.	yes	The study reports clear quantifiable outcomes (sensitivity, PPV, search speed comparisons).	Exclude	The study primarily focuses on epidemiological surveillance using computer vision for cooling tower detection, which diverges from the specified screening criteria for clinical applications and tabular data generation. While demonstrating empirical research with quantifiable outcomes, the paper fails key requirements by using image-based data and lacking direct clinical diagnostic or treatment applications. The methodology centers on environmental surveillance rather than patient-centered clinical research.	1.4	Exclude	Exclude	No tabular data
Automatic Catheter and Tube Detection in Pediatric X-ray Images Using a Scale-Recurrent Network and Synthetic Data.	Yi, X, Adams, Scott, Babyn, Paul, Elnajmi, Abdul	2020	maybe	The paper focuses on clinical applications (catheter detection in X-ray images), but it's primarily a technical solution. While it has clinical relevance, the focus appears to be on the technical implementation rather than direct clinical applications.	no	The study deals with X-ray images, not tabular data. This clearly fails to meet the criterion which specifically excludes imaging data.	yes	The study uses human clinical data (pediatric and adult chest X-rays), meeting this criterion.	yes	Based on the abstract, this appears to be an original research article with novel methodology.	yes	The study does involve active creation of synthetic data using AI (synthesizing catheters on X-ray images), meeting this criterion.	no	While the study does generate synthetic data, it's for image synthesis rather than tabular data generation. This fails to meet the criterion.	maybe	While the application is clinically relevant (catheter detection), the focus is more on the technical solution than direct clinical applications.	yes	The study includes empirical evaluation with clear metrics, meeting this criterion.	yes	The abstract mentions precision and recall metrics, meeting this criterion.	Exclude	The study focuses on a technical approach to catheter detection using X-ray images, which fundamentally conflicts with the screening criteria's requirement for tabular data generation. Despite demonstrating clinical relevance and involving synthetic data creation, the research fails key criteria by using imaging data instead of tabular formats. Consequently, the paper does not meet the systematic review's core screening requirements.	1.4	Exclude	Exclude	No tabular data
Automatic detection of abnormal vascular cross-sections based on density level detection and support vector machines.	Zuluaga, Maria A, Magnin, Isabelle E, Hernandez Hoyos, Marcela, Delgado Leyton, Edgar J F, Lozano, Fernando, Orkisz, Maciej	2011	maybe	The paper focuses on clinical applications (vascular anomaly detection) rather than purely technical aspects, but it is primarily a technical implementation paper.	no	The study uses imaging data (CT scans) rather than tabular data, which is a clear violation of the criterion requiring only tabular data.	yes	The study uses both synthetic phantoms and real coronary CT data from human patients.	yes	This is clearly an original research article with novel methodology and empirical evaluation.	no	The study uses synthetic phantoms, but these appear to be traditional synthetic data rather than AI-generated synthetic data. The focus is on detection rather than data generation.	no	While synthetic data is used, it's not generated using AI methods like GANs or VAEs. The synthetic data appears to be traditional phantoms.	yes	The study demonstrates direct clinical application in vascular anomaly detection to aid radiologists.	yes	The study includes thorough empirical evaluation with clear metrics and comparison against expert annotations.	yes	The study reports clear quantifiable outcomes including specificity (97.57% and 86.01%), sensitivity (82.19% and 81.23%), and kappa coefficient (0.72).	Exclude	The study focuses on vascular anomaly detection using CT imaging data, which deviates from the required tabular data format and lacks AI-generated synthetic data generation. While demonstrating strong clinical application and empirical evaluation, the research fails to meet critical screening criteria related to data type and synthetic data methodology. Consequently, the paper does not align with the systematic review's specific requirements.	1.4	Exclude	Exclude	No tabular data
Automatic motion artefact detection in brain T1-weighted magnetic resonance images from a clinical data warehouse using synthetic data	Loizillon S., Bottani S., Maire A., Stroer S., Dormont D., Colliot O., Burgos N.	2024	maybe	The paper focuses on clinical applications (MRI quality assessment) rather than purely technical aspects, but it is primarily a methodological paper for image quality assessment.	no	The study focuses on MRI images, not tabular data, which is a clear violation of the criterion requiring only tabular data generation.	yes	The study uses human clinical data from clinical data warehouses (CDWs) containing patient MRI scans.	yes	This is clearly an original research article with novel methodology and empirical evaluation.	maybe	While the study does create synthetic data (synthetic motion in MRIs), this is for training purposes rather than the primary focus of generating synthetic clinical data.	no	The study uses synthetic data generation, but for images rather than tabular data. The method appears to be transfer learning rather than traditional synthetic data generation methods like GANs or VAEs.	yes	The study has direct clinical applications in improving MRI quality assessment and data curation for clinical data warehouses.	yes	The study includes clear empirical evaluation with specific metrics and comparison to human annotators.	yes	The study reports quantifiable outcomes, specifically balanced accuracy metrics (>80%) for motion detection.	Exclude	The study focuses on MRI image quality assessment using transfer learning and synthetic motion detection, which fundamentally deviates from the required tabular data generation criteria. While demonstrating clinical relevance and empirical evaluation, the research primarily involves imaging data and methodological techniques that do not align with the specified screening requirements for synthetic tabular data generation. Consequently, the paper does not meet the core screening criteria for inclusion.	1.4	Exclude	Exclude	No tabular data
Automatic segmentation of breast MR images through a markov random field statistical model	Ribes S., Didierlaurent D., Decoster N., Gonneau E., Risser L., Feillel V., Caselles O.	2014	maybe	The paper focuses on clinical applications (breast MRI segmentation), but it's primarily a technical methodology paper. While it has clinical relevance, the focus is on the algorithmic approach rather than direct clinical applications.	no	The study deals with imaging data (MRI), not tabular data. This is a clear violation of the inclusion criteria.	yes	The study uses real breast MRI images from a high-risk population, so it does involve human clinical data.	yes	This appears to be an original research article presenting a new algorithm and its evaluation.	no	While the paper mentions using synthetic data, it appears to be using this for testing rather than creating synthetic clinical data. The focus is on segmentation algorithms, not synthetic data generation.	no	The paper does not involve generating synthetic tabular data using AI methods. While it uses synthetic data, it's for testing an imaging segmentation algorithm.	maybe	While the paper has clinical relevance (breast imaging), it focuses on the technical aspects of image segmentation rather than direct clinical applications like diagnosis or treatment planning.	yes	The study includes empirical evaluation with clear metrics and comparison against existing methods.	yes	The study reports clear quantifiable outcomes (Jaccard similarity coefficients, accuracy metrics).	Exclude	The study focuses on a technical imaging segmentation algorithm for breast MRI, which primarily involves imaging data rather than tabular data generation. While the research demonstrates clinical relevance and includes empirical evaluation, it does not meet key screening criteria related to synthetic data generation and tabular data formats. The paper's technical methodology and imaging focus diverge from the systematic review's core requirements.	1.4	Exclude	Exclude	No tabular data
Balancing the data term of graph-cuts algorithm to improve segmentation of hepatic vascular structures	Sangsefidi N., Foruzan A.H., Dolati A.	2018	maybe	The paper focuses on clinical applications (hepatic vessel segmentation for diagnosis and treatment planning), but it appears to be primarily a technical methodological paper rather than focusing on clinical applications.	no	The study deals with CT imaging data, not tabular data, which is a clear violation of the criterion.	yes	The study uses clinical CT images of human patients.	yes	This appears to be an original research article with clear methodology and results.	no	While the paper mentions using synthetic data, it appears to be using it for evaluation rather than creating synthetic data as its primary focus. The main contribution is an image processing algorithm.	no	The paper does not focus on generating synthetic data using AI methods. While synthetic data is mentioned, it's used as a test dataset rather than being the focus of the research.	yes	The study has direct clinical applications in diagnosis and treatment planning for hepatic vessels.	yes	The study includes empirical evaluation with clear metrics and comparison to state-of-the-art methods.	yes	The study reports quantifiable outcomes (Dice measure of 95.51%).	Exclude	The study focuses on hepatic vessel segmentation using CT imaging, which primarily involves technical image processing methodology rather than synthetic data generation. While the research demonstrates clinical relevance and includes empirical evaluation, it fundamentally fails to meet key screening criteria related to tabular data generation and AI-driven synthetic data creation. The paper's technical approach and imaging focus disqualify it from meeting the specified screening requirements.	1.4	Exclude	Exclude	No tabular data
Bayesian-Edge system for classification and segmentation of skin lesions in Internet of Medical Things.	Naseem, Shahid, Anwar, Muhammad, Faheem, Muhammad, Fayyaz, Muhammad, Malik, Muhammad Sheraz Arshad	2024	yes	The paper focuses on clinical applications (skin lesion segmentation for diagnosis), but also has significant technical components. However, the clinical application is primary, so this criterion is met.	no	The paper deals with image data, not tabular data. This is explicitly clear from the focus on "skin lesion segmentation from images." This is a critical failure of a core criterion.	maybe	The study appears to use human clinical data (skin lesions), though this isn't explicitly stated in the abstract.	yes	Yes, this appears to be an original research article with novel methodology combining Bayesian inference and edge intelligence.	maybe	While the abstract mentions "synthetic data generation," it's unclear if this is the main focus or just one component. The paper appears to focus more on the segmentation model than synthetic data creation.	maybe	The abstract mentions synthetic data generation but doesn't specify the methods used (GANs, VAEs, etc.). It's unclear if this is a primary focus or just a minor component.	yes	Yes, the study has direct clinical applications in diagnosis and risk assessment for skin diseases.	yes	Yes, the study includes empirical evaluation with clear metrics.	yes	Yes, the study reports quantifiable outcomes (87.80% diagnostic performance improvement).	Exclude	The study focuses on skin lesion segmentation using image data, which fundamentally conflicts with the requirement for tabular data generation. While demonstrating clinical applications and quantifiable outcomes, the research primarily involves image processing techniques rather than synthetic tabular data creation. Consequently, the paper does not meet the core screening criteria for synthetic data generation in clinical contexts.	1.4	Exclude	Exclude	No tabular data
Beyond the numbers: App-enabled stroke prediction system for high-risk individuals in imbalanced datasets	Eram A.F., Mahmud A.S., Khadem M.M., Ihsan M.A.	2025	yes	The study focuses on clinical applications, specifically stroke prediction and diagnosis, which is directly stated and is the primary focus of the research.	maybe	The abstract does not explicitly mention the data format, so it is unclear if the data is tabular or includes other formats like imaging data.	yes	The study deals with human clinical data for stroke prediction and diagnosis.	yes	This appears to be an original research article with novel methodology and results.	no	The abstract criticizes the use of synthetic data and instead focuses on improving real data analysis through different evaluation metrics. There is no mention of creating synthetic data.	no	The study explicitly states it is moving away from synthetic data approaches, instead focusing on real-world performance. No AI-based synthetic data generation is mentioned.	yes	The study has direct clinical applications in stroke diagnosis and prediction, with a mobile application implementation mentioned.	yes	The study includes empirical evaluation with clear metrics and comparison with previous approaches.	yes	Clear quantifiable outcomes are reported, including recall values (92%, 92%, and 94%) and comparison with previous benchmarks.	Exclude	The study focuses on stroke prediction using real clinical data, demonstrating strong clinical applications and quantifiable outcomes through machine learning models. However, the research explicitly rejects synthetic data generation, which disqualifies it from meeting key screening criteria related to AI-driven synthetic data creation. Despite robust clinical methodology and empirical evaluation, the paper does not align with the specific requirements for synthetic data research.	1.4	Exclude	Exclude	No synthetic data generation
Bidimensional ensemble entropy: Concepts and application to emphysema lung computerized tomography scans	Gaudencio A.S., Azami H., Cardoso J.M., Vaz P.G., Humeau-Heurtier A.	2023	no	The paper focuses on technical aspects (entropy algorithms) rather than clinical applications. While it has clinical implications, the primary focus is on developing and testing technical methods.	no	The study deals with image data (CT scans) and time series analysis, not tabular data generation.	yes	Yes, the study uses human clinical data (CT scans of patients with pulmonary emphysema).	yes	Yes, this appears to be an original research article with novel algorithms and empirical evaluation.	no	The study does not involve creating synthetic data using AI. While it mentions "synthetic images" for testing, these appear to be for validation rather than the main focus.	no	The paper does not involve generating synthetic tabular data using AI methods. It focuses on entropy algorithms for analyzing existing images.	maybe	While the study has clinical implications for emphysema diagnosis, it's primarily focused on developing technical methods rather than direct clinical applications.	yes	Yes, the study includes empirical evaluation with clear metrics.	yes	Yes, the study reports quantifiable outcomes (92.7% accuracy and 88.4% sensitivity for classification).	Exclude	The study primarily focuses on technical entropy algorithms for image analysis, failing to meet key screening criteria related to synthetic data generation and tabular data formats. While demonstrating clinical relevance through emphysema diagnosis and reporting quantifiable outcomes, the research does not align with the systematic review's core requirements for AI-driven synthetic data creation. The paper's technical methodology and image-based approach diverge significantly from the specified screening criteria.	1.4	Exclude	Exclude	No tabular data
Bidirectional Mapping of Brain MRI and PET With 3D Reversible GAN for the Diagnosis of Alzheimer's Disease	Lin W., Chen G., Zhang H., Gao Q., Huang Y., Tong T., Du M.	2021	yes	The study focuses on clinical applications, specifically Alzheimer's disease diagnosis using multi-modal imaging data.	maybe	The study deals with imaging data (MRI and PET), not tabular data, which is a critical failure of a core criterion.	yes	The study uses human clinical data from the Alzheimer's Disease Neuroimaging Initiative (ADNI) database.	yes	This appears to be an original research article with novel methodology and empirical evaluation.	yes	The study uses RevGAN to actively create synthetic data, not just using pre-existing synthetic datasets.	yes	The study uses AI methods (RevGAN) for synthetic data generation, though for images rather than tabular data.	yes	The study demonstrates direct clinical application in AD diagnosis and prediction.	yes	The study includes empirical evaluation on the ADNI database with clear comparison to existing methods.	yes	The abstract mentions performance evaluation and comparison with state-of-the-art methods, though specific metrics aren't detailed.	Exclude	The study focuses on Alzheimer's disease diagnosis using multi-modal imaging data and AI-generated synthetic data, demonstrating strong clinical application and empirical evaluation. However, the research primarily involves imaging data rather than tabular data, which critically fails a key screening criterion. Despite robust methodology and clinical relevance, the data format disqualifies the study from meeting the specified screening requirements.	1.4	Exclude	Exclude	No tabular data
Blind Source Separation in Persistent Atrial Fibrillation Electrocardiograms Using Block-Term Tensor Decomposition With Lowner Constraints	De Oliveira P.M.R., Goulart J.H.D.M., Fernandes C.A.R., Zarzoso V.	2022	maybe	The paper focuses on clinical applications (atrial fibrillation analysis from ECGs), but it appears to be primarily a technical/methodological paper about signal processing techniques. While it has clinical relevance, the focus is on the technical approach rather than direct clinical applications.	no	The study deals with ECG data, which is time-series data, not tabular data. This clearly fails to meet this criterion.	yes	Yes, the study uses real patient data from 20 patients with persistent AF.	yes	Yes, this appears to be an original research article with novel methodology and empirical evaluation.	no	While the paper mentions using synthetic data, it appears to be using this for validation rather than creating synthetic clinical data. The focus is on signal processing techniques, not synthetic data generation.	no	The paper does not involve generating synthetic tabular data using AI methods. While it uses synthetic data, it's for testing signal processing algorithms, not for generating synthetic clinical data.	maybe	While the paper has clinical relevance (atrial fibrillation analysis), it focuses on the technical aspects of signal processing rather than direct clinical applications like diagnosis or treatment planning.	yes	Yes, the study includes empirical evaluation with clear metrics for assessing the performance of their method.	yes	Yes, the study reports quantifiable outcomes related to signal estimation quality.	Exclude	The study focuses on signal processing techniques for atrial fibrillation analysis using ECG data, which fundamentally differs from the required tabular data generation criteria. While the research involves human clinical data and demonstrates empirical evaluation, it fails to meet key screening requirements related to synthetic data generation and data format. Consequently, the paper does not align with the systematic review's specific methodological constraints.	1.4	Exclude	Exclude	No tabular data

Brain tumor image generation using an aggregation of GAN models with style transfer	Mukherkjee D., Saha P., Kaplun D., Sinitca A., Sarkar R.	2022	no	The study focuses on technical aspects of image generation rather than clinical applications. While it's in the medical domain, the focus is on the GAN architecture and image generation quality, rather than direct clinical applications.	no	The study deals with MRI images, not tabular data. This is explicitly clear from the abstract mentioning "MRI scans" and "image analysis."	yes	The study uses human clinical data (brain tumor MRI scans) as its basis.	yes	This appears to be an original research article presenting a novel method (AGGrGAN).	yes	The study actively creates synthetic data using AI (specifically GANs) rather than using pre-existing synthetic datasets.	no	While the study does use AI methods (GANs) for synthetic data generation, it's for images rather than tabular data.	no	The abstract doesn't demonstrate direct clinical applications. It focuses on technical aspects of image generation and quality metrics (SSIM scores).	yes	The study includes empirical evaluation with clear metrics, though these are technical rather than clinical.	maybe	While the study reports quantifiable metrics (SSIM scores), these are technical image quality metrics rather than clinical outcomes.	Exclude	The study focuses on technical image generation using GANs for brain tumor MRI scans, failing key screening criteria related to tabular data and direct clinical applications. While demonstrating original research with empirical evaluation, the work primarily addresses methodological aspects of synthetic image creation rather than clinical utility or patient-centered outcomes. The research does not meet the required focus on tabular data generation or demonstrate clear clinical decision-making metrics.	1.4	Exclude	Exclude	No tabular data
Brain Tumor Recurrence vs. Radiation Necrosis Classification and Patient Survivability Prediction	Sadique M.S., Farzana W., Temtam A., Lappinen E., Vossough A., Iftekharuddin K.M.	2024	maybe	The paper focuses on clinical applications (differentiating recurrent brain tumor from radiation necrosis) rather than purely technical aspects, but it has a strong methodological component.	no	The study primarily deals with MRI imaging data, not tabular data, which is a clear violation of the criterion requiring only tabular data generation.	yes	The study uses human clinical data from patients with glioblastoma.	yes	This appears to be an original research article with empirical evaluation and results.	no	While the paper mentions "synthetic data generation using generative models," it appears to be discussing this as background rather than implementing it. The actual study uses "statistically rigorous repeated random sub-sampling" rather than AI-based synthetic data creation.	no	The paper does not actually implement synthetic data generation using AI methods. It uses statistical sampling techniques instead.	yes	The study demonstrates direct clinical applications in diagnosis (differentiating rBT from RN) and prognosis (survival prediction).	yes	The study includes original research with clear empirical evaluation and metrics.	yes	The study reports clear quantifiable outcomes (AUC of 0.892±0.055 for classification, AUC of 0.77±0.032 for survival prediction).	Exclude	The study focuses on clinical applications of brain tumor classification using MRI data, but fails to meet key screening criteria related to synthetic tabular data generation using AI methods. While demonstrating strong clinical relevance and empirical evaluation, the research does not involve AI-driven synthetic data creation or tabular data formats. Consequently, the paper does not align with the specified screening requirements for synthetic data research.	1.4	Exclude	Exclude	No tabular data
Brain tumor segmentation using K-means clustering and deep learning with synthetic data augmentation for classification.	Khan, Amjad Rehman, Khan, Siraj, Harouni, Majid, Abbasi, Rashid, Iqbal, Sajid, Mehmood, Zahid	2021	no	The study focuses on technical aspects of image processing and classification rather than clinical applications. While it has clinical relevance, the primary focus is on the technical methodology.	no	The study deals with MRI imaging data, not tabular data. This is explicitly clear from the abstract's focus on image processing and MRI analysis.	yes	The study uses MRI data from human patients with brain tumors.	yes	This appears to be an original research article presenting a new methodology.	maybe	While the study mentions "synthetic data augmentation," it's unclear if this involves active creation of synthetic data using AI or just traditional data augmentation techniques.	maybe	The abstract mentions synthetic data augmentation but doesn't specify the method used. It's not clear if this involves AI methods like GANs or VAEs.	maybe	While the study has clinical relevance for diagnosis, it focuses more on the technical aspects of classification rather than direct clinical applications.	yes	The study includes empirical evaluation using the BraTS 2015 benchmark dataset.	yes	The study reports classification accuracy and compares performance with state-of-the-art techniques.	Exclude	The study focuses on technical image processing for brain tumor classification using MRI data, which fundamentally diverges from the required tabular data format and clinical application focus. Despite involving human clinical data and demonstrating empirical evaluation, the research fails key screening criteria by emphasizing technical methodology over direct clinical utility. The synthetic data augmentation approach remains ambiguous and does not clearly meet the specified systematic review requirements.	1.4	Exclude	Exclude	No tabular data
Brain-age estimation accuracy is significantly increased using multishell free-water reconstruction	Nemmi F., Levardon M., Peran P.	2022	maybe	The paper focuses on clinical applications (brain age estimation) rather than purely technical aspects, but it is primarily a methodological comparison of different imaging reconstruction techniques.	no	The study uses imaging data (DWI/MRI) rather than tabular data, which fails this criterion.	yes	The study uses real human clinical data from 89 healthy males.	yes	This appears to be an original research article with empirical analysis.	no	The study does not involve creating synthetic data using AI. While it mentions "synthetic data" briefly, this appears to be a reference to previous findings rather than the focus of this study.	no	The study does not involve generating synthetic tabular data using AI methods. It focuses on analyzing real MRI data.	yes	The study demonstrates clinical application through brain age estimation, which has relevance to neurodegenerative diseases.	yes	The study includes original research with empirical evaluation and clear metrics for assessing the methods' performance.	yes	The study reports quantifiable outcomes in terms of brain-age estimation accuracy.	Exclude	The study focuses on brain age estimation using MRI data, which fundamentally differs from the required tabular data and synthetic data generation criteria. While demonstrating clinical relevance and empirical research, the paper fails key screening requirements related to data format and AI-driven synthetic data creation. Consequently, the research does not align with the specified screening criteria for inclusion.	1.4	Exclude	Exclude	No tabular data
CALLM: Enhancing Clinical Interview Analysis Through Data Augmentation with Large Language Models	Wu Y., Mao K., Zhang Y., Chen J.	2024	yes	The study focuses on clinical applications, specifically mental health diagnosis and PTSD detection using clinical transcripts.	no	The study deals with clinical transcripts and text data, not tabular data, which is a critical mismatch with the inclusion criteria.	yes	The study uses human clinical data, specifically clinical transcripts from mental health interviews.	yes	This appears to be an original research article with novel methodology and empirical results.	yes	The study actively creates synthetic data using AI (CALLM framework with large language models).	maybe	While the study does generate synthetic data using AI, it's focused on text generation rather than tabular data generation.	yes	The study demonstrates direct clinical application in mental health diagnosis and PTSD detection.	yes	The study includes original research with clear empirical evaluation and metrics.	yes	The study reports clear quantifiable outcomes (balanced accuracy of 0.77, F1-score of 0.70, AUC of 0.78).	Exclude	The study presents a novel AI-driven approach for generating synthetic clinical transcripts in mental health diagnosis, demonstrating strong clinical applications and empirical outcomes. However, the research focuses on text-based data generation rather than tabular data, which critically disqualifies it from meeting the systematic review's core screening criteria. Despite robust methodology and quantifiable results, the fundamental data format mismatch prevents inclusion.	1.4	Exclude	Exclude	No tabular data
Cardiac aging synthesis from cross-sectional data with conditional generative adversarial networks	Campello V.M., Xia T., Liu X., Sanchez P., Martin-Isla C., Petersen S.E., Segui S., Tsaftaris S.A., Lekadir K.	2022	maybe	The study focuses on technical aspects of generating synthetic cardiac imaging data rather than direct clinical applications. While it mentions cardiovascular disease, it's primarily about the technical process of age progression modeling.	maybe	The study deals with medical imaging data (heart scans), not tabular data. This is explicitly stated in the abstract.	yes	The study uses human clinical data from the UK Biobank, which contains real patient scans.	yes	Based on the abstract, this appears to be an original research article with novel methodology and results.	yes	The study actively creates synthetic data using a conditional GAN, which is clearly described in the abstract.	yes	The study uses AI methods (specifically a conditional GAN) for synthetic data generation.	maybe	While the study has potential clinical implications, it doesn't demonstrate direct clinical applications. It focuses on technical validation (image quality, age prediction) rather than clinical utility.	yes	The study includes empirical evaluation with clear metrics for assessing the synthetic data's quality.	yes	The study reports quantifiable outcomes including image quality metrics and mean absolute error for age prediction.	Exclude	The study focuses on generating synthetic cardiac imaging data using AI techniques, but fails to meet key screening criteria related to clinical applications and data format. Specifically, the research uses medical imaging data and lacks direct clinical utility, which disqualifies it from meeting the required screening standards. Despite demonstrating technical sophistication in synthetic data generation, the work does not align with the specified research parameters.	1.4	Exclude	Exclude	No tabular data
Cardiac CT motion artifact grading via semi-automatic labeling and vessel tracking using synthetic image-augmented training data.	Xu, Yongshun, Sushmit, Asif, Lyu, Qing, Li, Ying, Cao, Ximiao, Maltz, Jonathan S, Wang, Ge, Yu, Hengyong	2022	maybe	The paper focuses on clinical applications (cardiac CT image quality grading) rather than purely technical aspects, but it is primarily focused on image processing and quality assessment, which is only partially aligned with the criterion.	no	The study deals with imaging data (CT scans), not tabular data, which is a clear violation of the criterion.	yes	The study uses both clinical data and synthetic phantom data based on human cardiac anatomy.	yes	This appears to be an original research article with novel methodology and results.	no	The study uses XCAT simulation tools to generate synthetic data, but these appear to be physics-based simulations rather than AI-generated synthetic data.	no	While synthetic data is used, it's generated through XCAT simulation tools rather than AI methods like GANs or VAEs.	yes	The study has direct clinical applications in cardiac CT image quality assessment and diagnostic value.	yes	The study includes original research with clear empirical evaluation and metrics.	yes	The study reports quantifiable outcomes (scoring accuracy within 0.85 points, 36% reduction in mean square error).	Exclude	The study involves cardiac CT image quality assessment using simulation tools, but fails to meet key screening criteria due to its focus on imaging data rather than tabular data and its use of physics-based simulation instead of AI-generated synthetic data. Despite demonstrating strong clinical applications and empirical evaluation, the fundamental methodological approach disqualifies the paper from meeting the specified screening requirements.	1.4	Exclude	Exclude	No tabular data
CCSI: Continual Class-Specific Impression for data-free class incremental learning.	Ayromlou, Sana, Tsang, Teresa, Abolmaesumi, Purang, Li, Xiaoxiao	2024	maybe	The paper focuses on clinical applications, specifically disease classification and diagnosis in medical imaging. However, it has a strong technical focus on the methodology of class incremental learning.	no	The study appears to focus on medical imaging data (echocardiography cine series and MedMNIST datasets), not tabular data.	yes	The study uses human clinical data (echocardiography cine series and medical imaging datasets).	yes	This appears to be an original research article with novel methodology and empirical evaluation.	maybe	The study does involve active creation of synthetic data using AI (CCSI - Continual Class-Specific Impression), but it's for medical images rather than tabular data.	maybe	While the study does generate synthetic data using AI methods, it's specifically for medical images rather than tabular data.	yes	The study demonstrates direct clinical applications in disease diagnosis and classification.	yes	The study includes original research with clear empirical evaluation and metrics for assessing performance.	yes	The study reports quantifiable outcomes, specifically classification accuracy improvements of up to 51%.	Exclude	The study focuses on medical imaging data and AI-driven synthetic data generation for disease classification, which fundamentally conflicts with the requirement for tabular data. Despite demonstrating strong clinical applications, empirical evaluation, and quantifiable outcomes, the core data format criterion is not met. Consequently, the paper does not align with the specified screening requirements.	1.4	Exclude	Exclude	No tabular data
Challenges and Risks of Using Synthetic Data for AI-Driven Healthcare Applications.	Karoulla, Eirini, Matragkas, Nikolaos, Islam, Saif Ul, Epiphaniou, Gregory	2025	maybe	The abstract discusses synthetic data in healthcare broadly but does not specify clinical applications. It focuses more on risks and challenges rather than clinical applications.	maybe	The abstract does not specify the type of data being discussed. There is no mention of tabular data specifically.	maybe	While healthcare is mentioned, there is no specific indication of human clinical data usage.	no	This appears to be a review or perspective paper highlighting challenges and risks, not original research.	no	The abstract appears to be discussing synthetic data utilization in general rather than active creation of synthetic data.	no	While synthetic data is discussed, there is no mention of specific AI methods for generation.	no	The paper focuses on risks and challenges rather than demonstrating clinical applications.	no	The abstract suggests this is a discussion paper about challenges rather than an empirical evaluation.	no	There is no mention of specific outcomes or metrics. The paper appears to be qualitative in nature.	Exclude	The abstract discusses synthetic data challenges in healthcare but lacks specific details about clinical applications, data generation methods, or empirical research. Multiple screening criteria remain unmet, including original data creation, quantifiable outcomes, and direct clinical utility. The paper appears to be a conceptual review highlighting risks rather than an original research study with concrete synthetic data implementation.	1.4	Exclude	Exclude	Wrong publication type
ChatGPT M.D.: Is there any room for generative AI in neurology?.	Nogradi, Bernat, Polgar, Tamas Ferenc, Meszlenyi, Valeria, Kadar, Zalan, Hertelendy, Peter, Csati, Anett, Szpisjak, Laszlo, Halmi, Dora, Erdelyi-Furka, Barbara, Toth, Mate, Molnar, Fanny, Toth, David, Bosze, Zsofia, Boda, Krisztina, Klivenyi, Peter, Siklos, Laszlo, Patai, Roland	2024	yes	The study focuses on clinical applications, specifically diagnostic augmentation in neurology, rather than purely technical, privacy, methodological or educational aspects.	yes	The study uses tabular/synthetic patient data (anamneses), which appears to be in a structured format suitable for analysis.	yes	The study uses synthetic patient data based on real neurological cases, though it's not direct clinical data.	yes	This appears to be an original research article with empirical evaluation and results.	no	The synthetic data was "generated by neurological experts" rather than using AI methods to create the synthetic data.	no	While synthetic data is used, it's not generated using AI methods (GANs, VAEs, etc.) but rather created by human experts.	yes	The study demonstrates direct clinical application in diagnosis and diagnostic augmentation.	yes	The study includes empirical evaluation with clear metrics and cross-validation by experts.	yes	Clear quantifiable outcomes are reported (diagnostic accuracy percentages with standard deviations).	Exclude	The study demonstrates strong clinical application in neurology diagnostic augmentation, with clear quantifiable outcomes and empirical evaluation. However, it critically fails key screening criteria by using expert-generated synthetic data rather than AI-generated synthetic data, which disqualifies it from meeting the systematic review's core requirements. Despite robust methodology and clinical relevance, the fundamental approach to data generation does not align with the specified screening criteria.	1.4	Exclude	Exclude	No synthetic data generation
Chest tomosynthesis image enhancement by bone suppression using convolutional neural networks with synthetic data	Xu X., Li Q., Jin S., Su Z., Zhang Y.	2024	maybe	The paper focuses on clinical applications (bone suppression in chest tomosynthesis imaging), but it's primarily a technical development rather than a direct clinical application. It's more focused on the imaging technique than clinical outcomes.	no	The study deals with imaging data (CTS projections and CT images), not tabular data.	yes	Yes, the study uses both synthetic data from CT images and real patient data from the Shimadzu system.	yes	Yes, this appears to be an original research article with empirical evaluation.	maybe	The study does create synthetic data using AI (deep learning-based bone suppression model), but this is for training the model rather than the primary focus of the study.	no	While the study uses synthetic data, it's not generating synthetic tabular data. Instead, it generates synthetic imaging data from CT images.	maybe	While the study has potential clinical applications (aiding radiologists in detecting lung lesions), it's primarily focused on the technical development of the imaging technique.	yes	Yes, the study includes empirical evaluation with clear metrics.	yes	Yes, the study reports quantifiable outcomes (bone suppression rate >90%, SSIM values, MAEs).	Exclude	The study focuses on imaging data and technical development of a bone suppression model, which fundamentally conflicts with the screening criteria requiring tabular data generation. Despite demonstrating clinical potential and using real patient data, the research fails key criteria related to data format and synthetic data generation. Consequently, the paper does not meet the essential requirements for inclusion in the review.	1.4	Exclude	Exclude	No tabular data

Comparative analysis of nonlinear dimensionality reduction techniques for breast MRI segmentation.	Akhbardeh, Alireza, Jacobs, Michael A	2012	maybe	The paper focuses on clinical applications (breast MRI analysis and lesion segmentation) rather than purely technical aspects. However, it's primarily focused on the technical methodology of image processing and dimensionality reduction.	no	The study deals with imaging data (MRI), not tabular data. This is a clear violation of the criterion requiring only tabular data generation.	yes	The study uses human clinical data (25 breast lesion subjects).	yes	This is an original research article with clear methodology and results.	no	While the study uses synthetic data for validation, it's not focused on creating synthetic data using AI. The synthetic data appears to be used as a comparison tool rather than the main focus.	no	The study does not generate synthetic tabular data using AI methods. While it uses synthetic data, it's for validation of imaging processing techniques, not for generating synthetic tabular data.	yes	The study demonstrates direct clinical applications in breast lesion segmentation and visualization.	yes	The study includes original research with clear empirical evaluation and metrics.	yes	The study reports quantifiable outcomes (>86% accuracy in tissue segmentation).	Exclude	The study focuses on breast MRI image processing using advanced machine learning techniques, but fails to meet key screening criteria related to synthetic tabular data generation. While demonstrating clinical applications and using human clinical data, the research primarily involves imaging data processing and dimensionality reduction, which diverges from the specified requirements for synthetic data creation. Consequently, the paper does not align with the core screening criteria for synthetic data research.	1.4	Exclude	Exclude	No tabular data
Component analysis approach to estimation of tissue intensity distributions of 3D images	Zagorodnov V., Ciptadi A.	2011	no	The paper focuses on technical aspects of image segmentation algorithms rather than clinical applications. While it mentions clinical environment, the focus is on the technical methodology of tissue intensity probability modeling.	no	The study deals with imaging data (MRI scans), not tabular data. This is explicitly mentioned in the abstract.	yes	The study uses real MRI scans of the brain, which constitutes human clinical data.	yes	This appears to be an original research article presenting a novel algorithm.	no	While the paper mentions using synthetic data, it appears to be using it for testing rather than creating synthetic clinical data. The focus is on the algorithm for analyzing real imaging data.	no	The paper does not involve generating synthetic tabular data using AI methods. It uses synthetic data for testing but doesn't generate synthetic clinical data.	no	While the paper has potential clinical applications in imaging, it doesn't demonstrate direct clinical applications like diagnosis or treatment planning. It focuses on the technical aspects of image segmentation.	maybe	The study includes empirical evaluation on both synthetic and real MRI data, but the metrics for assessing clinical utility are not clearly specified.	no	The abstract mentions that the approach "performed well" but doesn't provide specific quantifiable outcomes or metrics.	Exclude	The study focuses on technical image segmentation algorithms using MRI data, which fundamentally diverges from the screening criteria's requirements for tabular synthetic data generation and clinical applications. Despite using human clinical data and presenting original research, the paper fails to meet key criteria related to synthetic data creation, clinical utility, and quantifiable outcomes. The technical nature of the research and its emphasis on imaging methodology render it incompatible with the specified screening requirements.	1.4	Exclude	Exclude	No tabular data
Computing ischemic regions in the heart with the bidomain model - First steps towards validation	Nielsen B.F., Lysaker M., Grottum P.	2013	maybe	The paper focuses on clinical applications (identifying ischemic regions in the heart), but it's primarily a technical/mathematical modeling study. While it has clinical relevance, the focus appears to be on the mathematical methodology rather than direct clinical application.	no	The study uses body surface potential maps (BSPMs) and perfusion scintigraphy images, which are not tabular data.	yes	Yes, the study uses human clinical data from four patients.	yes	Yes, this appears to be an original research article with empirical evaluation.	no	The study does not involve creating synthetic data using AI. While it mentions "synthetic data" briefly, this refers to simulation data for testing the algorithm, not AI-generated synthetic data.	no	The study does not use AI methods for synthetic data generation. The "synthetic data" mentioned is simulation data for testing purposes.	maybe	While the study has potential clinical applications (identifying ischemic regions), it's primarily focused on validating a mathematical model rather than demonstrating direct clinical utility.	yes	Yes, the study includes empirical evaluation with clear metrics comparing results with perfusion scintigraphy.	yes	Yes, the study reports quantifiable outcomes (relative differences between center of mass and size of ischemic regions).	Exclude	The study focuses on a mathematical modeling approach for identifying ischemic heart regions using body surface potential maps, but fails key screening criteria related to synthetic data generation and tabular data format. While the research involves human clinical data and reports quantifiable outcomes, it does not meet the core requirements for AI-driven synthetic data creation or clinical application focus.	1.4	Exclude	Exclude	No tabular data
Conditional Generative Models for Synthetic Tabular Data: Applications for Precision Medicine and Diverse Representations	Liu K., Altman R.B.	2025	maybe	The paper appears to focus on clinical applications rather than purely technical aspects, as it discusses precision medicine and patient care. However, it is explicitly stated to be a review paper, which is problematic for this criterion.	yes	The paper focuses on tabular medical data (EHRs, biobanks, clinical trial data), which meets this criterion.	yes	The paper discusses human clinical data (EHRs, biobanks, clinical trial data), meeting this criterion.	no	The abstract explicitly states this is a "review" paper, which directly fails this critical criterion.	no	While the paper discusses AI-based synthetic data generation, it appears to be reviewing existing methods rather than creating new synthetic data.	maybe	The paper discusses AI methods (specifically conditional generative models) for synthetic data generation, meeting this criterion in principle, but as a review rather than original research.	yes	The paper discusses clinical applications including precision medicine and patient care, meeting this criterion.	no	As a review paper, it doesn't include original empirical evaluation.	maybe	The abstract mentions "evaluation criteria" but doesn't specifically indicate reporting of quantifiable outcomes, as this is a review paper.	Exclude	A comprehensive review of conditional generative models for synthetic medical data generation fails to meet key screening criteria, particularly the requirement for original research. While addressing important clinical applications and tabular data formats, the paper's review nature disqualifies it from inclusion, lacking empirical evaluation and original data creation. The manuscript provides valuable insights into synthetic data generation but does not constitute an original research article.	1.4	Exclude	Exclude	Wrong publication type
Connected-UNets: a deep learning architecture for breast mass segmentation	Baccouche A., Garcia-Zapirain B., Castillo Olea C., Elmaghraby A.S.	2021	maybe	The paper focuses on clinical applications (breast cancer diagnosis through mammogram analysis), but it's primarily a technical/methodological paper about improving UNet architectures. While it has clinical relevance, the focus is on the technical implementation.	no	The paper deals with imaging data (mammograms), not tabular data. This is a clear violation of the criterion.	yes	Yes, the study uses human clinical data (mammograms from CBIS-DDSM, INbreast, and a private dataset).	yes	Yes, this appears to be an original research article with novel architecture proposals and empirical evaluation.	maybe	The paper does use AI (CycleGAN) to create synthetic data, but this is only for augmenting existing images, not creating new tabular data.	no	While the paper does use AI for synthetic data generation, it's for image synthesis (using CycleGAN) rather than tabular data generation.	yes	Yes, the study has direct clinical applications in breast cancer diagnosis through mass segmentation.	yes	Yes, the study includes original research with clear empirical evaluation and metrics.	yes	Yes, the study reports clear quantifiable outcomes (Dice scores and IoU metrics).	Exclude	The study focuses on breast cancer mammogram analysis using advanced AI techniques, but primarily involves image data processing rather than tabular data generation. While demonstrating clinical relevance and empirical evaluation, the research fundamentally deviates from the specified screening criteria by working with imaging data instead of tabular data. Consequently, the paper does not meet the core requirements for inclusion in the systematic review.	1.4	Exclude	Exclude	No tabular data
Constrained generative adversarial network ensembles for sharable synthetic medical images	Dikici E., Bigelow M., White R.D., Erdal B.S., Prevedello L.M.	2021	no	The study focuses primarily on technical aspects of generating synthetic medical images for privacy protection, rather than clinical applications.	no	The study deals with medical imaging data (MRI), not tabular data.	yes	The study uses human clinical data (brain metastasis MRI images).	yes	This appears to be an original research article with novel methodology and results.	yes	The study actively creates synthetic data using GAN ensembles.	yes	The study uses AI methods (GANs) for synthetic data generation.	maybe	While the study demonstrates application in a clinical context (brain metastasis detection), the primary focus is on data generation for privacy protection rather than direct clinical applications.	yes	The study includes empirical evaluation with clear metrics comparing performance on original vs. synthetic data.	yes	The study reports quantifiable outcomes (false positives at 90% detection sensitivity).	Exclude	The study focuses on generating synthetic medical images using GANs for privacy protection, which fundamentally deviates from the required tabular data format and clinical application focus. Despite demonstrating sophisticated AI-driven data generation and empirical evaluation, the research fails key screening criteria by prioritizing technical methodology over direct clinical utility. Consequently, the paper does not align with the specified review requirements.	1.4	Exclude	Exclude	No tabular data
Constraint-Aware Learning for Fractional Flow Reserve Pullback Curve Estimation From Invasive Coronary Imaging.	Zhang, Dong, Liu, Xiujian, Wang, Anbang, Zhang, Hongwei, Yang, Guang, Zhang, Heye, Gao, Zhifan	2024	yes	The study focuses on clinical applications, specifically FFR estimation for coronary intervention guidance.	maybe	The study primarily deals with imaging data (coronary imaging) and FFR pullback curves, which are time-series data. This fails the criterion as the review focuses on tabular data only.	yes	The study uses real-world data from 382 patients and involves clinical coronary imaging.	yes	This appears to be an original research article with novel methodology and empirical evaluation.	yes	The study involves active creation of synthetic data using AI methods, specifically mentioned in "leverages the power of synthetic data in model training."	yes	The study uses AI methods (diffusion model) for synthetic data generation, though the specific architecture (GAN/VAE) isn't explicitly stated.	yes	The study has direct clinical applications in coronary intervention guidance and FFR estimation.	yes	The study includes extensive experimental studies with clear metrics and validation against real-world data.	yes	The study reports quantifiable outcomes including performance metrics and correlation with invasively measured FFR values.	Exclude	The study demonstrates strong clinical relevance in coronary intervention guidance through AI-driven synthetic data generation, utilizing real-world patient data and empirical validation. However, the research focuses on imaging and time-series data rather than tabular data, which disqualifies it from meeting the specific screening criteria. Despite robust methodology and quantifiable clinical outcomes, the data format represents a critical exclusion point.	1.4	Exclude	Exclude	No tabular data
Convergence of evolving artificial intelligence and machine learning techniques in precision oncology	Fountzilas E., Pearce T., Baysal M.A., Chakraborty A., Tsimberidou A.M.	2025	maybe	The paper discusses clinical applications in precision oncology, including diagnostic approaches and therapeutic strategies, but it appears to be more of an overview of AI/ML applications rather than focusing on a specific clinical application.	no	The abstract mentions "multi-dimensional, multiomic, spatial pathology, and radiomic data" which suggests non-tabular data formats are included, failing this criterion as it's not limited to tabular data.	yes	The paper discusses cancer patients and clinical applications, so it does involve human clinical data.	no	Based on the abstract's tone and content, this appears to be a review or perspective piece rather than original research, discussing various applications and challenges.	maybe	While synthetic data generation is mentioned ("digital twins"), it's discussed as one of many applications rather than being the primary focus of original research.	maybe	The paper mentions synthetic data generation, but it's not clear if it specifically uses AI methods like GANs or VAEs. The focus seems broader than just synthetic data generation.	maybe	While clinical applications are mentioned (diagnosis, treatment selection), they appear to be discussed generally rather than demonstrated specifically.	no	There's no indication of original empirical evaluation or clear metrics for assessing synthetic data's utility.	no	The abstract doesn't mention any specific quantifiable outcomes or metrics.	Exclude	The abstract discusses AI and machine learning in precision oncology, but fails to meet several key screening criteria, including being an original research article and focusing exclusively on tabular data generation. While touching on synthetic data and clinical applications, the text appears to be a broad overview rather than a specific empirical study with quantifiable outcomes, significantly limiting its alignment with the specified screening requirements.	1.4	Exclude	Exclude	Wrong publication type
Correction of partial volume effect in 18F-FDG PET brain studies using coregistered MR volumes: Voxel based analysis of tracer uptake in the white matter	Coello C., Willoch F., Selnes P., Gjerstad L., Fladby T., Skretting A.	2013	no	The paper focuses on a technical method (LoReAn algorithm) for PET imaging correction, rather than a direct clinical application. While it has clinical relevance, it's primarily a technical/methodological paper.	no	The study deals with imaging data (PET and MRI), not tabular data. This is explicitly clear from the abstract.	yes	The study uses both synthetic and clinical data, including human brain imaging data.	yes	This appears to be an original research article presenting a new algorithm and its validation.	maybe	The study uses synthetic data, but it's not clear if this is AI-generated. The focus is on the correction algorithm rather than synthetic data generation.	no	While synthetic data is used, there's no indication that AI methods (GANs, VAEs, etc.) were used for generation. The synthetic data appears to be used for validation rather than being the focus.	maybe	While the method has potential clinical applications in studying metabolism in white matter regions, it's primarily focused on the technical aspects of image correction rather than direct clinical applications.	yes	The study includes empirical evaluation with clear metrics comparing the new algorithm against existing methods.	yes	The study reports quantifiable outcomes (bias and coefficient of variation) for the algorithm's performance.	Exclude	The study focuses on a technical imaging correction algorithm using PET and MRI data, which fundamentally differs from the required tabular data and AI-driven synthetic data generation criteria. While the research demonstrates empirical validation and uses clinical data, it fails to meet key screening requirements related to data format, application focus, and synthetic data generation methodology. The paper's technical nature and imaging-based approach render it incompatible with the specified screening criteria.	1.4	Exclude	Exclude	No tabular data
Cough detection by ensembling multiple frequency subband features	You M., Liu Z., Chen C., Liu J., Xu X.-H., Qiu Z.-M.	2017	maybe	The paper focuses on clinical applications (cough detection for respiratory disease diagnosis), but it's primarily a technical methodology paper. While it has clinical relevance, the focus appears to be on the technical aspects of signal processing and machine learning.	no	The study deals with audio data, not tabular data. This is a clear violation of the criterion, which specifically excludes non-tabular formats.	yes	Yes, the study uses real data from 18 patients with respiratory diseases.	yes	Yes, this appears to be an original research article with empirical evaluation.	maybe	The abstract mentions using both synthetic and real data, but doesn't indicate that they actively created synthetic data using AI methods. The synthetic data generation process is not described.	maybe	While synthetic data is mentioned, there's no indication that AI methods (GANs, VAEs, etc.) were used for generation. The focus is on machine learning for classification, not synthetic data generation.	yes	Yes, the study has direct clinical applications in cough detection and diagnosis.	yes	Yes, the study includes empirical evaluation with clear metrics.	yes	Yes, the study reports quantifiable outcomes (accuracy improvements of 3.2%).	Exclude	The study focuses on cough detection using machine learning techniques, but fails to meet key screening criteria due to its use of audio data instead of tabular data. While demonstrating clinical relevance and empirical evaluation, the research does not involve AI-based synthetic data generation, which disqualifies it from the systematic review. The technical methodology and audio signal processing approach do not align with the specified screening requirements.	1.4	Exclude	Exclude	No tabular data
Counterfactual MRI Generation with Denoising Diffusion Models for Interpretable Alzheimer's Disease Effect Detection.	Dhinagar, Nikhil J, Thomopoulos, Sophia I, Laltoo, Emily, Thompson, Paul M	2024	maybe	The paper focuses on clinical applications in Alzheimer's disease diagnosis and analysis, but also has significant technical components. While it has clinical relevance, it's not purely clinical-focused.	no	The study focuses on MRI imaging data, not tabular data. This is a clear violation of the criterion requiring only tabular data generation.	yes	The study uses real MRI scans from human patients with Alzheimer's disease.	yes	This appears to be an original research article with novel methodology and results.	yes	The study actively creates synthetic data using AI (specifically diffusion models), meeting this criterion.	maybe	While the study does use AI methods (diffusion models) for synthetic data generation, it's for imaging data rather than tabular data.	yes	The study demonstrates direct clinical applications in AD diagnosis and personalized disease mapping.	yes	The study includes clear empirical evaluation with specific metrics for assessing synthetic data quality and clinical utility.	yes	The study reports quantifiable outcomes, including a 3% improvement in classifier performance.	Exclude	The study focuses on generating synthetic MRI data for Alzheimer's disease using AI diffusion models, demonstrating clinical applications and quantifiable outcomes. However, the research primarily involves imaging data rather than tabular data, which represents a critical deviation from the specified screening criteria. Despite meeting multiple other requirements, the imaging data format fundamentally disqualifies the study from the review.	1.4	Exclude	Exclude	No tabular data

Coupled Hidden Markov Model-Based Method for Apnea Bradycardia Detection	Montazeri Ghahjaverestan N., Masoudi S., Shamsollahi M.B., Beuchee A., Pladys P., Ge D., Hernandez A.I.	2016	maybe	The paper focuses on clinical applications (apnea-bradycardia detection in neonates) rather than purely technical aspects, but it has a strong methodological component.	no	The study primarily deals with physiological signal recordings (ECG) and beat-to-beat features, which are time-series data, not tabular data.	yes	The study uses real clinical data from neonates with apnea-bradycardia.	yes	This is clearly an original research article presenting a novel framework and empirical evaluation.	no	The paper uses synthetic data generated by the FitzHugh-Nagumo model, but this is not AI-generated synthetic data.	no	While synthetic data is used, it's generated using the FitzHugh-Nagumo model, not AI methods like GANs or VAEs.	yes	The study demonstrates direct clinical application in detecting apnea-bradycardia episodes.	yes	The study includes original research with clear empirical evaluation and metrics.	yes	The study reports clear quantifiable outcomes including sensitivity, specificity, and detection delay metrics.	Exclude	The study focuses on a clinical application for detecting apnea-bradycardia in neonates using a novel coupled hidden Markov model, but fails to meet key screening criteria related to AI-generated synthetic data and tabular data formats. While demonstrating strong clinical relevance and empirical evaluation, the research does not align with the specific methodological requirements for synthetic data generation using AI techniques.	1.4	Exclude	Exclude	No tabular data
Coupling synthetic and real-world data for a deep learning-based segmentation process of 4D flow MRI.	Garzia, Simone, Scarpolini, Martino Andrea, Mazzoli, Marilena, Capellini, Katia, Monteleone, Angelo, Cademartiri, Filippo, Positano, Vincenzo, Celi, Simona	2023	maybe	The paper focuses on clinical applications (imaging and segmentation of thoracic aorta), but the primary focus appears to be on the technical methodology of generating synthetic data. While it has clinical relevance, it's more methodologically oriented.	no	The study deals with imaging data (4D flow MRI), not tabular data.	yes	The study uses human clinical data (patient-specific PCMRA images) as its basis.	yes	This appears to be an original research article with clear methodology and results.	yes	The study does involve active creation of synthetic data using AI (neural networks) and computational methods.	no	While the study does generate synthetic data, it's for imaging data (PCMRA) rather than tabular data. The generation method combines statistical shape modeling and computational fluid dynamics.	yes	The study demonstrates clinical application in imaging and segmentation of thoracic aorta, which has direct clinical relevance.	yes	The study includes original research with clear empirical evaluation and metrics.	yes	The study reports quantifiable outcomes (DICE score of 0.83 vs 0.65).	Exclude	The study focuses on generating synthetic imaging data for thoracic aorta segmentation using neural networks, but critically fails to meet key screening criteria related to tabular data generation. While demonstrating strong clinical relevance and empirical methodology, the research primarily involves imaging data rather than the required tabular format, rendering it incompatible with the specified screening requirements.	1.4	Exclude	Exclude	No tabular data
Crop filling: A pipeline for repairing memory clinic MRI corrupted by partial brain coverage	Leal G.C., Whitfield T., Praharaju J., Walker Z., Oxtoby N.P.	2024	no	The paper focuses on technical aspects (MRI image processing) rather than direct clinical applications. While it has healthcare relevance, it's primarily about data processing methodology.	no	The study deals with imaging data (MRI scans), not tabular data. This is explicitly clear from the abstract's focus on T1 and T2 weighted MRI scans.	yes	The study uses human clinical data from memory clinics.	yes	Based on the abstract's structure and content, this appears to be an original research article presenting a new method.	yes	The study does involve active creation of synthetic data using AI ("synthesise a new T1 using an open source deep learning tool").	yes	The study uses AI methods (deep learning) to generate synthetic data, though for imaging rather than tabular data.	no	While the method has potential clinical utility, the abstract doesn't demonstrate direct clinical applications. It focuses on data processing to make data usable for research.	maybe	While the abstract describes a method, it doesn't clearly indicate whether there was empirical evaluation with specific metrics.	no	The abstract doesn't mention specific quantifiable outcomes or metrics for evaluating the synthetic data's quality or clinical utility.	Exclude	The study focuses on MRI image processing using deep learning to generate synthetic data, but fails key screening criteria by dealing with imaging rather than tabular data and lacking direct clinical applications. While the research involves human clinical data and AI-driven synthetic data generation, it primarily addresses technical methodology rather than demonstrating quantifiable clinical outcomes or utility.	1.4	Exclude	Exclude	No tabular data
Cross modality medical image synthesis for improving liver segmentation	Rafiq M., Ali H., Mujtaba G., Shah Z., Azmat S.	2025	no	The study focuses on technical aspects of image synthesis and segmentation rather than direct clinical applications. While it's in the medical domain, it's primarily about improving image processing techniques.	no	The study deals with medical imaging data (MRI and CT), not tabular data. This is explicitly clear from the abstract's focus on image synthesis and segmentation.	yes	The study uses human clinical data (abdominal MRI and CT images).	yes	This appears to be an original research article with novel methodology and results.	yes	The study actively creates synthetic data using AI (specifically CycleGAN and their proposed EssNet), not using pre-existing synthetic datasets.	maybe	While the study does use AI methods (GANs) for synthetic data generation, it's for images rather than tabular data.	no	The study focuses on improving image segmentation techniques rather than direct clinical applications like diagnosis or treatment planning.	yes	The study includes original research with clear empirical evaluation and metrics.	yes	The study reports quantifiable outcomes (1.17% improvement in IoU for liver segmentation).	Exclude	The study focuses on technical image synthesis using AI methods for medical imaging, primarily addressing data generation and segmentation techniques rather than direct clinical applications. While demonstrating empirical research with quantifiable outcomes, the work fundamentally diverges from the screening criteria by using imaging data instead of tabular data and lacking direct clinical utility. The research represents a methodological advancement in image processing but does not align with the specified screening requirements.	1.4	Exclude	Exclude	No tabular data
DAGAN-based Gait Features Augmentation for Ankle Instability Detection	Liu X., Zhang Y., Jiao Y., Zheng B., Guo Q., Yu Y., Wulamu A.	2025	yes	The study focuses on clinical diagnosis of ankle ligament injury, which is a clinical application rather than purely technical aspects.	maybe	The study deals with gait kinematic characteristics and time-series data, which is explicitly excluded by the criteria as it's not tabular data.	yes	The study uses human gait data from the Heidelberg Foot Measurement Method.	yes	This appears to be an original research article with novel methodology and results.	yes	The study actively creates synthetic data using an Attention Dual Generative Adversarial Network (Attention-DualGAN).	yes	The study uses AI methods (specifically GANs) to generate synthetic data.	yes	The study demonstrates direct clinical application in diagnosis of ankle ligament injury.	yes	The study includes clear empirical evaluation with specific metrics and visualization methods.	yes	The study reports specific accuracy improvements (2.4% and 7.47%) and uses Pearson correlation coefficients as metrics.	Exclude	The study focuses on clinical diagnosis of ankle ligament injury using AI-generated synthetic gait data, demonstrating strong clinical application and empirical evaluation. However, the use of time-series gait kinematic data disqualifies the study from meeting the screening criteria, which specifically require tabular data formats. Despite meeting most other criteria, the data format violation prevents the study from being included in the review.	1.4	Exclude	Exclude	No tabular data
Data augmented lung cancer prediction framework using the nested case control NLST cohort	Jiang Y., Manem V.S.K.	2025	maybe	The paper focuses on clinical applications (lung cancer screening) rather than purely technical aspects, but it is primarily focused on data augmentation techniques, which is somewhat methodological.	no	The study focuses on CT scan images, not tabular data, which is explicitly excluded by the screening criteria.	yes	The study uses human clinical data from the National Lung Screening Trial (NLST) cohort.	yes	The paper appears to be an original research article with clear methodology and results.	maybe	The study does involve creating synthetic data using AI methods (MED-DDPM and other generative models), but this is primarily for data augmentation rather than creating complete synthetic datasets.	no	While the study uses AI methods for data generation, it's specifically for image data augmentation rather than tabular data generation.	yes	The study demonstrates direct clinical application in lung cancer screening and prediction.	yes	The study includes original research with clear empirical evaluation and metrics.	yes	The study reports quantifiable outcomes including accuracy, F1 score, and AUC metrics.	Exclude	The study focuses on lung cancer screening using CT scan images and data augmentation techniques, which fundamentally conflicts with the requirement for tabular data generation. Despite demonstrating strong clinical relevance and empirical evaluation, the research fails key screening criteria by using imaging data instead of tabular data, rendering it ineligible for inclusion. The methodological approach, while innovative, does not align with the specified research parameters.	1.4	Exclude	Exclude	No tabular data
Deep Convolutional Generative Adversarial Networks to Enhance Artificial Intelligence in Healthcare: A Skin Cancer Application	La Salvia M., Torti E., Leon R., Fabelo H., Ortega S., Martinez-Vega B., Callico G.M., Leporati F.	2022	maybe	The paper focuses on clinical applications (skin cancer diagnosis) rather than purely technical aspects, but it appears to be primarily focused on the technical solution of generating synthetic data.	no	The study focuses on hyperspectral images, which are explicitly mentioned as the data format, and this fails to meet the criterion requiring only tabular data generation.	yes	The study involves epidermal lesions and skin cancer, suggesting human clinical data as the basis.	yes	Based on the abstract's structure and content, this appears to be an original research article.	yes	The study involves active creation of synthetic data using a deep convolutional GAN, meeting this criterion.	yes	The study uses AI methods (specifically GANs) for synthetic data generation, meeting this criterion.	yes	The study demonstrates direct clinical application in skin cancer diagnosis.	maybe	The abstract indicates empirical evaluation was performed, but doesn't provide clear metrics for assessing clinical utility.	maybe	While the abstract mentions "experimental results show effectiveness," it doesn't explicitly detail quantifiable outcomes or metrics.	Exclude	The study focuses on generating synthetic hyperspectral images for skin cancer diagnosis using deep learning techniques, which fundamentally conflicts with the requirement for tabular data generation. Despite demonstrating clinical application and original research in synthetic data creation, the use of image-based data disqualifies the study from meeting the core screening criteria. The technical approach and clinical intent are strong, but the data format represents a critical exclusion factor.	1.4	Exclude	Exclude	No tabular data
Deep learning enables fast and accurate quantification of MRI-guided near-infrared spectral tomography for breast cancer diagnosis	Feng J., Tang Y., Lin S., Jiang S., Xu J., Zhang W., Geng M., Dang Y., Wei C., Li Z., Sun Z., Jia K., Pogue B.W., Paulsen K.D.	2025	yes	The study focuses on clinical applications, specifically breast cancer diagnosis using MR-guided NIRST imaging, rather than purely technical, privacy, methodological or educational aspects.	maybe	The study deals with imaging data (MRI and NIRST), not tabular data, which is a critical failure of a core criterion.	yes	The study uses data from 38 clinical imaging exams of patients with breast abnormalities.	yes	This appears to be an original research article with empirical evaluation and results.	yes	The abstract mentions using synthetic data for training, indicating active creation of synthetic data.	yes	While the specific AI method isn't named, the abstract indicates using deep learning for synthetic data generation.	yes	The study demonstrates direct clinical application in breast cancer diagnosis with clear diagnostic outcomes.	yes	The study includes clear empirical evaluation with statistical analysis and performance metrics.	yes	The study reports specific quantifiable outcomes including sensitivity (87.5%), specificity (92.9%), and diagnostic accuracy (89.5%).	Exclude	The study demonstrates strong clinical application in breast cancer diagnosis using synthetic data and deep learning, with robust empirical evaluation and quantifiable outcomes. However, the research fundamentally fails to meet the screening criteria due to its focus on imaging data rather than tabular data generation, rendering it ineligible for further review. Despite high-quality methodology and significant clinical insights, the data format represents a critical disqualifying factor.	1.4	Exclude	Exclude	No tabular data
Deep learning for cranioplasty in clinical practice: Going from synthetic to real patient data.	Kodym, Oldrich, Spanel, Michal, Herout, Adam	2021	yes	The study focuses on clinical applications, specifically cranioplasty and cranial implant design, rather than purely technical, privacy, methodological or educational aspects.	maybe	The study deals with volumetric data and 3D skull reconstructions, not tabular data, which is a critical mismatch with the inclusion criteria.	yes	The study uses clinical data from patients indicated for cranioplasty.	yes	This appears to be an original research article with novel methodology and empirical results.	yes	The study involves active creation of synthetic data using deep learning methods, specifically for skull patches and cranial implant shapes.	yes	The study uses AI methods (CNNs) for generating synthetic data, though it's for 3D volumetric data rather than tabular data.	yes	The study demonstrates direct clinical applications in cranioplasty and cranial implant design.	yes	The study includes original research with clear empirical evaluation and metrics for assessing the method's performance.	yes	The study reports clear quantifiable outcomes including surface distance metrics (0.67mm), curvature error reduction (12%), and Dice coefficient (0.88).	Exclude	The study demonstrates strong clinical relevance in cranioplasty with advanced AI techniques for skull reconstruction, utilizing human clinical data and reporting quantifiable outcomes. However, the research focuses on volumetric 3D data generation rather than tabular data, which represents a critical deviation from the specified screening criteria. Despite meeting most other requirements, this fundamental data format mismatch prevents the study from fully qualifying under the established screening parameters.	1.4	Exclude	Exclude	No tabular data
Deep Learning Model for Diagnosing and Classifying Subtypes of Chronic Pulmonary Aspergillosis in Chest CT	Wei J., Zhou L., Zhang D., Guo G., Li Z., Fang J., Yan X., Li Y., Zhang X., Huang C., Lan R., Shi C., Liu D., Luo L., Long C., Chen H., Ye Y.	2025	yes	The study focuses on clinical applications, specifically diagnosing chronic pulmonary aspergillosis (CPA) and its subtypes, which is directly clinical rather than purely technical.	maybe	The study focuses on CT images, which are imaging data, not tabular data.	yes	The study uses human clinical data, specifically chest CT images from 660 patients.	yes	This appears to be an original research article with clear methodology and results.	yes	The study involves active creation of synthetic data using AI, specifically mentioning "artificial intelligence generation technology" for data augmentation.	yes	The study uses AI methods for synthetic data generation, though the specific method (GANs, VAEs, etc.) isn't explicitly stated.	yes	The study demonstrates direct clinical application in diagnosis and classification of CPA.	yes	The study includes thorough empirical evaluation with multiple test sets and clear metrics.	yes	The study reports clear quantifiable outcomes including sensitivity, accuracy, F1 score, Matthews correlation coefficient, and AUC.	Exclude	The study demonstrates strong clinical application in diagnosing chronic pulmonary aspergillosis using AI-generated synthetic data, with robust empirical evaluation and quantifiable outcomes. However, the research focuses on imaging data rather than tabular data, which disqualifies it from meeting the systematic review's specific screening criteria. Despite its methodological rigor and clinical relevance, the imaging data format prevents inclusion in the review.	1.4	Exclude	Exclude	No tabular data
Deep Learning Synthesis of White-Blood From Dark-Blood Late Gadolinium Enhancement Cardiac Magnetic Resonance.	Jaspers, Tim J M, Martens, Bibi, Crawley, Richard, Jada, Lamis, Amirrajab, Sina, Breeuwer, Marcel, Holtackers, Robert J, Chiribiri, Amedeo, Scannell, Cian M	2024	maybe	The study focuses on clinical applications (cardiac imaging) rather than purely technical aspects, but it is primarily focused on image generation rather than direct clinical decision-making.	no	The study deals with medical imaging data (LGE cardiac MRI), not tabular data, which is explicitly excluded by the criterion.	yes	The study uses data from 215 human patients.	yes	The study appears to be an original research article with clear methodology and results.	yes	The study involves active creation of synthetic data using AI (CycleGAN and contrastive unpaired translation models).	yes	The study uses AI methods (specifically GANs) for synthetic data generation.	maybe	While the study has potential clinical applications, it focuses on image generation rather than direct clinical applications like diagnosis or treatment planning. The clinical utility is indirect.	yes	The study includes clear empirical evaluation with specific metrics and comparison with real images.	yes	The study reports quantifiable outcomes including scar burden quantification and image quality scores.	Exclude	The study involves synthetic medical image generation using AI, utilizing data from 215 patients and demonstrating quantifiable outcomes through advanced machine learning techniques. However, the research focuses on medical imaging data, which explicitly violates the screening criteria requiring tabular data generation. Despite meeting multiple other criteria, the fundamental data format disqualifies the study from further consideration.	1.4	Exclude	Exclude	No tabular data

Deep learning with cinematic rendering: fine-tuning deep neural networks using photorealistic medical images	Mahmood F., Chen R., Sudarsky S., Yu D., Durr N.J.	2018	no	The study focuses on technical aspects of image processing and synthetic data generation for endoscopy, rather than direct clinical applications.	no	The study deals with medical imaging data (CT and endoscopy images), not tabular data.	maybe	The study uses CT data and porcine (pig) colon endoscopy images, not human clinical data.	yes	This appears to be an original research article with novel methodology and empirical evaluation.	yes	The study involves active creation of synthetic data using cinematic rendering and deep learning techniques.	maybe	While the study does generate synthetic data, it's focused on image generation rather than tabular data generation.	no	The study focuses on technical aspects of depth estimation in endoscopy rather than direct clinical applications like diagnosis or treatment planning.	yes	The study includes clear empirical evaluation with specific metrics.	yes	The study reports specific quantifiable outcomes (56.87% and 27.49% error reduction).	Exclude	The study focuses on technical image processing using cinematic rendering and deep learning, which diverges from the required clinical application and tabular data generation criteria. Despite demonstrating empirical evaluation and quantifiable outcomes, the research primarily addresses imaging techniques with porcine data, failing to meet key screening requirements for clinical utility and data format. Consequently, the paper does not align with the systematic review's core screening criteria.	1.4	Exclude	Exclude	No tabular data
Deep learning-based markerless lung tumor tracking in stereotactic radiotherapy using Siamese networks	Grama D., Dahele M., van Rooij W., Slotman B., Gupta D.K., Verbakel W.F.A.R.	2023	yes	The study focuses on clinical applications in radiotherapy, specifically tumor tracking during radiation delivery, which is a clinical application rather than purely technical.	no	The study primarily deals with imaging data (kV images, CT scans) rather than tabular data, which is a critical failure of this core criterion.	yes	The study uses both phantom data and real patient data from six patients, which meets the requirement for using human clinical data.	yes	The study appears to be an original research article with clear methodology and results.	yes	The study creates synthetic data (DRRs) from 4D planning CT scans using deep learning methods, which meets the requirement for original data creation.	maybe	While the study does generate synthetic data using AI (deep Siamese networks), it's not generating tabular data but rather synthetic images (DRRs), which only partially meets this criterion.	yes	The study has direct clinical applications in radiotherapy treatment delivery and tumor tracking, which meets the requirement for clinical application.	yes	The study includes original empirical evaluation with clear metrics comparing the Siamese model against a conventional benchmark method, which meets the requirement for empirical evaluation.	yes	The study reports clear quantifiable outcomes including mean absolute distance measurements and correlation coefficients, which meets the requirement for quantifiable outcomes.	Exclude	The study focuses on radiotherapy tumor tracking using deep learning, generating synthetic images from CT scans and demonstrating clinical applications with quantifiable outcomes. However, the research primarily involves imaging data rather than tabular data, which critically disqualifies it from meeting the core screening requirements. Despite high-quality methodology and empirical evaluation, the fundamental data format criterion is not satisfied.	1.4	Exclude	Exclude	No tabular data
Deep Reinforcement Learning-based Automatic Augmentation for Gastrointestinal Disease Classification	Wrona A., Atanasious M.M.H., Delli Priscoli F.	2025	no	The study focuses primarily on technical aspects (image augmentation and classification) rather than clinical applications. While it mentions "clinical diagnostics," the focus is on the AI methodology.	no	The study deals with medical imaging data, not tabular data. This is explicitly stated in the abstract ("medical imaging data," "image classification").	maybe	While the study mentions "gastrointestinal diseases," it's not explicitly stated whether the data is from human patients.	yes	This appears to be an original research article with novel methodology and results.	yes	The study involves active creation of synthetic data using AI methods (GANs and VAEs are mentioned).	maybe	While the study does use AI methods for synthetic data generation, it's focused on image data rather than tabular data.	no	The study focuses on improving image classification accuracy rather than direct clinical applications like diagnosis or treatment planning.	yes	The study includes empirical evaluation with clear metrics (test accuracy).	yes	The study reports quantifiable outcomes (3% improvement in image classification).	Exclude	The study primarily focuses on technical image classification methodology using AI techniques, which diverges from the required clinical and tabular data generation criteria. While demonstrating synthetic data generation and empirical evaluation, the research fails to meet key screening requirements related to clinical application, data format, and direct patient-focused outcomes. The technical nature of the work and its emphasis on image processing render it incompatible with the specified screening parameters.	1.4	Exclude	Exclude	No tabular data
DeepFake electrocardiograms using generative adversarial networks are the beginning of the end for privacy issues in medicine	Thambawita V., Isaksen J.L., Hicks S.A., Ghouse J., Ahlberg G., Linneberg A., Grarup N., Ellervik C., Olesen M.S., Hansen T., Graff C., Holstein-Rathlou N.-H., Strumke I., Hammer H.L., Maleckar M.M., Halvorsen P., Riegler M.A., Kanters J.K.	2021	no	The paper focuses on generating synthetic ECGs, which is primarily a technical/methodological contribution rather than a clinical application. While ECGs are used in clinical settings, the paper doesn't demonstrate direct clinical applications.	no	The study focuses on ECG data, which is time-series data, not tabular data.	yes	The study uses real ECGs from population studies, indicating human clinical data.	yes	This appears to be an original research article with novel methodology and results.	yes	The study clearly involves active creation of synthetic data using AI (GANs).	yes	The study uses GANs to generate synthetic data.	no	The paper doesn't demonstrate direct clinical applications. It focuses on generating synthetic ECGs for privacy preservation and research sharing, rather than clinical use.	maybe	The study includes empirical evaluation of the synthetic data generation methods, but not of clinical utility.	no	While the study reports some metrics (ECG intervals and amplitudes), these are focused on data quality rather than clinical outcomes.	Exclude	The study focuses on generating synthetic ECGs using GANs, which fails key screening criteria by using time-series data instead of tabular data and lacking direct clinical applications. While the research demonstrates technical innovation in synthetic data generation, it does not meet the required clinical focus and outcome metrics for inclusion. The methodology is primarily oriented toward privacy preservation and research data sharing rather than clinical utility.	1.4	Exclude	Exclude	No tabular data
DeepFake knee osteoarthritis X-rays from generative adversarial neural networks deceive medical experts and offer augmentation potential to automatic classification.	Prezja, Fabi, Paloneva, Juha, Polonen, Ilkka, Niinimaki, Esko, Ayramo, Sami	2022	no	The study focuses primarily on technical aspects of generating synthetic X-ray images rather than direct clinical applications. While it has potential clinical implications, the primary focus is on the technical generation and validation of synthetic images.	no	The study deals with X-ray images, not tabular data. This is explicitly clear from the abstract which mentions "X-ray images" multiple times.	yes	The study uses real knee joint X-rays with osteoarthritis severity, which implies human clinical data.	yes	This appears to be an original research article with novel methodology and results.	yes	The study actively creates synthetic data using GANs, generating 320,000 synthetic X-ray images from 5,556 real images.	yes	The study uses AI methods (specifically GANs) to generate synthetic data.	maybe	While the study has potential clinical applications in osteoarthritis classification, it doesn't directly demonstrate clinical applications. The focus is more on the technical validation of the synthetic images.	yes	The study includes clear empirical evaluation through expert validation and classification tasks with specific metrics.	yes	The study reports quantifiable outcomes including classification accuracy metrics and expert validation results.	Exclude	The study focuses on generating synthetic X-ray images using GANs, which fundamentally conflicts with the requirement for tabular data generation. While demonstrating technical innovation in synthetic data creation, the research fails to meet key screening criteria related to data format and clinical application focus. The technical approach and image-based methodology disqualify the study from meeting the specified screening requirements.	1.4	Exclude	Exclude	No tabular data
Deepfake-driven Disinformation Dissemination in Biotechnology: Mechanisms and Governance Strategies	Cao D.	2025	no	The paper focuses on security and privacy aspects of deepfake detection in biotech, rather than clinical applications. It's primarily about protecting against synthetic data threats rather than using synthetic data for clinical purposes.	no	The paper discusses genetic data and medical imagery, but not specifically tabular data generation. It appears to focus on various data types including images and genetic information.	maybe	While the paper mentions clinical trial outcomes, it's not clear if it uses actual human clinical data as its basis. The focus appears to be on security mechanisms.	yes	The abstract suggests this is an original research article proposing a new governance strategy and detection system.	no	While the paper discusses deepfake technology, it focuses on detection and protection rather than active creation of synthetic data for clinical use.	no	The paper discusses deepfake technology but in the context of detection and protection, not generation for clinical purposes.	no	The paper focuses on security and protection against deepfakes rather than direct clinical applications like diagnosis or treatment.	maybe	While the paper proposes a model, it's not clear if it includes empirical evaluation with clear metrics.	no	The abstract doesn't mention specific quantifiable outcomes or metrics for evaluation.	Exclude	The study primarily addresses cybersecurity and deepfake detection in biotechnology, falling short of the screening criteria focused on clinical applications and synthetic data generation. While presenting an original research approach, the paper does not meet key requirements related to clinical utility, tabular data generation, or direct patient care applications. The research is more oriented toward protecting against synthetic data threats rather than leveraging synthetic data for clinical purposes.	1.4	Exclude	Exclude	No tabular data
Deep-Learning-Based Electrical Noise Removal Enables High Spectral Optoacoustic Contrast in Deep Tissue	Dehner C., Olefir I., Chowdhury K.B., Justel D., Ntziachristos V.	2022	maybe	The paper focuses on technical aspects (signal processing and noise reduction) rather than direct clinical applications. While it has clinical relevance, the primary focus is on the technical methodology.	no	The study deals with imaging data (optoacoustic tomography) and signal data, not tabular data.	yes	Yes, the study includes human clinical data (human breast imaging).	yes	Yes, this appears to be an original research article with novel methodology and results.	maybe	The study does create synthetic data, but it's used for training a denoising model rather than being the primary focus of the research.	maybe	While synthetic data is used, it's not the main focus and is used for training rather than being the primary output. The synthetic data generation method isn't clearly specified as using AI methods like GANs or VAEs.	maybe	While the technology has clinical applications, the paper focuses on the technical aspects of noise reduction rather than direct clinical applications like diagnosis or treatment planning.	yes	Yes, the study includes empirical evaluation with clear metrics.	yes	Yes, the study reports quantifiable outcomes (19% higher blood vessel contrast).	Exclude	The study focuses on technical signal processing for optoacoustic tomography, primarily involving imaging data rather than tabular data, which disqualifies it from the systematic review. While the research includes human clinical data and demonstrates quantifiable outcomes, its technical methodology and non-tabular data format do not align with the specified screening criteria. The paper's emphasis on noise reduction techniques and imaging signals further confirms its exclusion from the review.	1.4	Exclude	Exclude	No tabular data
Deformable image registration with high-dimensional map constraint and alignment-synthesis enhancement feature fusion	Zhou H., Hu S., Wang Y., Zhang L., Li G., Qu C., Wang C., Zhang R., Liu S.	2025	no	The paper focuses on medical image registration, which is a technical aspect rather than a direct clinical application. While it has clinical relevance, it's primarily a technical methodology paper.	no	The study deals with medical imaging data (3D brain MR datasets), not tabular data. This clearly fails the criterion requiring only tabular data generation.	yes	The study uses human clinical data (3D brain MR datasets).	yes	This appears to be an original research article with novel methodology and experimental results.	maybe	The paper does involve creating synthetic data (LSDFs), but these are generated from existing images rather than using AI to create entirely new synthetic data.	no	While the paper does involve synthetic data generation, it's not using AI methods like GANs or VAEs to generate tabular data. Instead, it generates synthetic LSDFs from existing images.	no	While medical image registration has clinical applications, the paper focuses on the technical methodology rather than demonstrating direct clinical applications.	yes	The study includes empirical evaluation with "extensive experiments" on three datasets.	yes	The paper reports performance comparisons with state-of-the-art methods, indicating quantifiable outcomes.	Exclude	The study focuses on medical image registration using synthetic data generation techniques, primarily addressing technical methodological aspects rather than direct clinical applications. While the research involves human clinical data and demonstrates empirical evaluation, it fundamentally fails key screening criteria by working with imaging data instead of tabular data and not using AI methods for synthetic data generation. The paper's technical orientation and imaging-based approach significantly deviate from the specified screening requirements.	1.4	Exclude	Exclude	No tabular data
De-identifying Spanish medical texts - named entity recognition applied to radiology reports	Perez-Diez I., Perez-Moraga R., Lopez-Cerdan A., Salinas-Serrano J.-M., la Iglesia-Vaya M.	2021	no	The paper focuses on anonymization and de-identification of medical texts, which is primarily a technical/privacy aspect rather than a clinical application. While it has healthcare relevance, it's not directly focused on clinical applications.	no	The study deals with text data (radiology reports and EHRs), not tabular data. This is explicitly clear from the abstract.	yes	The study uses real human clinical data (radiology reports and EHRs).	yes	This appears to be an original research article with novel methodology and empirical evaluation.	no	The study does involve creating synthetic data through randomization of entities, but this is not using AI methods like GANs or VAEs. Instead, it uses a randomization algorithm.	no	While synthetic data is generated, it's through randomization rather than AI methods like GANs or VAEs. The focus is on anonymization rather than synthetic data generation for clinical purposes.	no	The study focuses on data anonymization rather than direct clinical applications like diagnosis or treatment planning.	yes	The study includes empirical evaluation with clear metrics for assessing the anonymization strategy.	yes	The study reports quantifiable outcomes (recall metrics of 97.18% and 69.18% for different datasets).	Exclude	The study focuses on text data anonymization using randomization techniques, which diverges from the required tabular data and AI-driven synthetic data generation criteria. Despite demonstrating empirical evaluation and using human clinical data, the research fails to meet key screening requirements related to clinical applications and synthetic data generation methods. Consequently, the paper does not align with the specified screening criteria for inclusion.	1.4	Exclude	Exclude	No tabular data
Detecting anomalous sequences in electronic health records using higher-order tensor networks	Niu H., Omitaomu O.A., Langston M.A., Olama M., Ozmen O., Klasky H.B., Laurio A., Sauer B., Ward M., Nebeker J.	2022	maybe	The paper focuses on anomaly detection in EHR data, which has clinical applications, but appears to be primarily a technical/methodological paper about the detection algorithm. While it has clinical relevance, it's not directly focused on clinical applications.	no	The study deals with sequence data from EHRs, which is not purely tabular data. The focus on "sequences" and "subsequences" indicates time-series data, which is explicitly excluded in the criteria.	yes	The study uses real-world EHR data, which implies human clinical data.	yes	This appears to be an original research article presenting new algorithms and their evaluation.	no	The abstract mentions using both synthetic data and real-world EHR data, but doesn't indicate active creation of synthetic data using AI. The synthetic data appears to be used for testing rather than being the focus of creation.	no	While synthetic data is mentioned, there's no indication that AI methods (GANs, VAEs, etc.) are used for generating synthetic data. The focus is on anomaly detection algorithms.	maybe	While the study has potential clinical applications in improving patient care processes, it doesn't directly demonstrate clinical applications like diagnosis or treatment planning.	yes	The study includes empirical evaluation comparing performance on synthetic and real-world data.	maybe	The abstract mentions that the algorithm "outperforms baseline methods" but doesn't specify clinical metrics.	Exclude	The study focuses on anomaly detection in electronic health records using sequence data, which deviates from the required tabular data format and lacks direct AI-driven synthetic data generation. While the research involves human clinical data and includes empirical evaluation, it primarily represents a technical methodology paper with limited direct clinical application. The core screening criteria related to synthetic data generation and tabular data format are not met.	1.4	Exclude	Exclude	No tabular data
Detecting the clinical features of difficult-to-treat depression using synthetic data from large language models.	Lorge, Isabelle, Joyce, Dan W, Taylor, Niall, Nevado-Holgado, Alejo, Cipriani, Andrea, Kormilitzin, Andrey	2025	yes	The study focuses on clinical applications, specifically detecting difficult-to-treat depression (DTD) using EHR data.	no	The study primarily deals with narrative (free-text) EHR data, not tabular data, which is a critical mismatch with the inclusion criteria.	yes	The study uses human clinical data from electronic health records.	yes	This appears to be an original research article with novel methodology and results.	yes	The study uses GPT3.5 to actively create synthetic data for training their model.	maybe	While AI (GPT3.5) is used to generate synthetic data, it's generating text data rather than tabular data as required by the criteria.	yes	The study demonstrates direct clinical application in detecting DTD and extracting prognostic factors.	yes	The study includes empirical evaluation with clear metrics on both synthetic and clinical test sets.	yes	The study reports quantifiable outcomes including F1 scores (0.70 and 0.85) and precision metrics.	Exclude	The study focuses on detecting difficult-to-treat depression using synthetic data generation and AI techniques, demonstrating strong clinical applications and empirical evaluation. However, the research primarily involves text-based electronic health record data, which critically fails the requirement for tabular data generation. Despite meeting most screening criteria, the data format disqualifies the study from inclusion.	1.4	Exclude	Exclude	No tabular data

Development of a simple numerical model for trabecular bone structures	Pena-Solorzano C.A., Albrecht D.W., Paganin D.M., Harris P.C., Hall C.J., Bassed R.B., Dimmock M.R.	2019	maybe	The paper focuses on generating synthetic bone structures for phantoms and quality assurance, which is more technical/methodological than clinical. While it mentions potential ML applications, it's not primarily focused on clinical applications.	no	The study deals with volumetric and structural data of bone microarchitecture, not tabular data.	yes	The study uses real human bone data as a basis for the synthetic generation.	yes	This is clearly an original research article with novel methodology and results.	maybe	The study does involve active creation of synthetic data, but uses mathematical modeling rather than AI methods.	no	While synthetic data is generated, it's through mathematical modeling rather than AI methods like GANs or VAEs.	no	The primary applications mentioned are for quality assurance and validation processes, not direct clinical applications like diagnosis or treatment.	yes	The study includes clear empirical evaluation with specific metrics comparing synthetic to real structures.	yes	The study reports specific quantifiable metrics (mean thickness error, standard deviation differences).	Exclude	The study focuses on generating synthetic bone structures using mathematical modeling, which diverges from the required AI-driven tabular data generation approach. While the research demonstrates empirical evaluation and uses human clinical data, it fails to meet key screening criteria related to data format, synthetic data generation method, and direct clinical applications. The technical methodology and non-AI approach significantly limit its alignment with the specified screening requirements.	1.4	Exclude	Exclude	No tabular data
Differences in technical and clinical perspectives on AI validation in cancer imaging: mind the gap!.	Chouvarda, Ioanna, Colantonio, Sara, Verde, Ana S C, Jimenez-Pastor, Ana, Cerda-Alberich, Leonor, Metz, Yannick, Zacharias, Lithin, Nabhani-Gebara, Shereen, Bobowicz, Maciej, Tsakou, Gianna, Lekadir, Karim, Tsiknakis, Manolis, Marti-Bonmati, Luis, Papanikolaou, Nikolaos	2025	maybe	The paper focuses on AI validation strategies in cancer imaging, which is clinical in nature. However, it appears to be more focused on methodological aspects of validation rather than direct clinical applications.	no	The paper discusses cancer imaging data, which is not tabular data. It explicitly mentions "cancer imaging" multiple times and discusses segmentation metrics, indicating imaging data is the focus.	maybe	The study focuses on cancer imaging, which implies human clinical data, but the paper itself doesn't directly work with clinical data - it surveys professionals about their practices.	yes	This appears to be an original research article based on survey responses from 49 professionals, analyzing trends and patterns.	no	While synthetic data is mentioned, it's discussed as a topic of discussion among researchers rather than the paper actively creating synthetic data. The paper is a survey of practices rather than original synthetic data creation.	no	The paper mentions synthetic data but only in the context of discussing validation strategies. It doesn't involve actual generation of synthetic data using AI methods.	no	The paper focuses on validation strategies rather than direct clinical applications. While it's in the context of cancer imaging, it doesn't demonstrate specific clinical applications.	no	While the paper includes empirical evaluation of survey responses, it doesn't evaluate synthetic data's clinical utility.	no	The paper reports on survey responses and trends but doesn't appear to include specific quantifiable clinical outcomes.	Exclude	The study focuses on AI validation strategies in cancer imaging, primarily examining methodological practices through a survey of professionals, but does not meet key screening criteria related to synthetic tabular data generation or direct clinical applications. While the research provides insights into AI validation approaches, it lacks the specific elements of original synthetic data creation, quantifiable clinical outcomes, and empirical evaluation of synthetic data's clinical utility.	1.4	Exclude	Exclude	No tabular data
Diffusion MRI GAN synthesizing fibre orientation distribution data using generative adversarial networks	Vellmer S., Aydogan D.B., Roine T., Cacciola A., Picht T., Fekonja L.S.	2025	no	The paper focuses on generating synthetic medical imaging data (FODs), which is primarily technical rather than clinical. While it mentions potential clinical applications, the focus appears to be on the technical methodology.	no	The study deals with 3D MRI volumes and FODs, which are complex imaging data formats, not tabular data.	yes	The study uses data from the Human Connectome Project (HCP), which is human clinical data.	yes	Based on the abstract's structure and content, this appears to be an original research article.	yes	The study actively creates synthetic data using an alpha-WGAN combining GAN and VAE approaches.	yes	The study uses AI methods (specifically GANs and VAEs) for synthetic data generation.	no	While the paper mentions potential clinical applications for rare pathologies, it doesn't demonstrate direct clinical applications. The focus is on the technical generation of synthetic data.	maybe	The study includes empirical evaluation of the synthetic data generation process, but focuses on technical validation rather than clinical utility.	maybe	The abstract mentions that the synthetic FODs "produce anatomically accurate fibre bundles and connectomes, with properties matching those from our validation dataset," but doesn't provide specific quantifiable clinical outcomes.	Exclude	The study focuses on generating synthetic medical imaging data using AI techniques, primarily addressing technical methodology rather than direct clinical applications. While the research involves human clinical data and uses advanced generative models, it fails key screening criteria by working with complex imaging data instead of tabular formats and not demonstrating clear clinical utility. The technical nature of the work and lack of quantifiable clinical outcomes suggest exclusion from the systematic review.	1.4	Exclude	Exclude	No tabular data
DIMOND: DIffusion Model OptimizatioN with Deep Learning	Li Z., Bilgic B., Lee H.-H., Ying K., Huang S.Y., Liao H., Tian Q.	2024	maybe	The paper focuses on clinical applications of diffusion MRI for brain imaging and microstructure mapping, but it appears to be primarily a technical/methodological paper about improving parameter estimation. While it has clinical applications, the focus seems to be on the technical aspects.	no	The study deals with imaging data (diffusion MRI), not tabular data, which is a clear violation of the inclusion criteria.	yes	The study uses in vivo human brain data.	yes	This appears to be an original research article presenting a new framework (DIMOND).	maybe	The paper does involve AI-based generation of synthetic data, but it's for the purpose of training/optimizing the model rather than creating synthetic clinical data for clinical use.	maybe	While the paper does use AI methods (deep learning) to generate synthetic data, it's for technical optimization rather than generating synthetic clinical data.	maybe	While the paper mentions clinical applications, it focuses on the technical aspects of improving parameter estimation rather than direct clinical applications.	yes	The study includes empirical evaluation with clear metrics for assessing performance.	yes	The paper reports quantifiable outcomes in terms of model fitting time and performance metrics.	Exclude	The study focuses on a technical methodology for diffusion MRI parameter estimation using deep learning, which primarily involves imaging data rather than tabular clinical data. While the research demonstrates empirical evaluation and uses human brain data, it fails to meet key screening criteria, particularly the requirement for tabular data generation. The paper's technical orientation and imaging-based approach significantly limit its alignment with the specified screening requirements.	1.4	Exclude	Exclude	No tabular data
Discovering digital tumor signatures-using latent code representations to manipulate and classify liver lesions	Kleesiek J., Kersjes B., Ueltzhoffer K., Murray J.M., Rother C., Kothe U., Schlemmer H.-P.	2021	no	The study focuses primarily on technical aspects of image generation and feature extraction rather than direct clinical applications. While it has potential clinical implications, the focus is on the technical methodology.	no	The study deals with medical imaging (CT scans) and image generation, not tabular data. This is explicitly clear from the abstract's focus on "liver tumor lesions" and "CT images."	yes	The study uses human clinical data (CT scans from patients with liver lesions).	yes	This appears to be an original research article with novel methodology and empirical evaluation.	yes	The study actively creates synthetic data using AI methods (specifically an implicit autoencoder with GAN-like components).	no	While the study does generate synthetic data using AI methods, it generates synthetic images rather than tabular data.	maybe	While the study has potential clinical applications (tumor detection and characterization), it doesn't directly demonstrate clinical applications but rather focuses on the technical capability to generate synthetic images.	yes	The study includes empirical evaluation with clear metrics and validation by radiologists.	yes	The study reports quantifiable outcomes including accuracy, sensitivity, and specificity metrics for lesion detection.	Exclude	The study focuses on technical image generation using AI methods for liver tumor lesions, primarily dealing with medical imaging rather than tabular data. While demonstrating sophisticated synthetic data creation and empirical validation, the research fails to meet key screening criteria related to clinical application and data format. The methodology shows promise for potential clinical applications but does not directly address the specified screening requirements.	1.4	Exclude	Exclude	No tabular data
Domain adaptation strategies for 3D reconstruction of the lumbar spine using real fluoroscopy data	Jecklin S., Shen Y., Gout A., Suter D., Calvet L., Zingg L., Straub J., Cavalcanti N.A., Farshad M., Furnstahl P., Esfandiari H.	2024	maybe	The paper focuses on clinical applications (surgical navigation in orthopedic surgeries) rather than purely technical aspects, though it has a strong technical component in developing the X23D system.	no	The study primarily deals with imaging data (fluoroscopic images and 3D reconstructions), not tabular data, which is a clear violation of the criterion.	yes	The study uses real intraoperative fluoroscopic images from human patients, though it also works with synthetic data.	yes	This appears to be an original research article with novel contributions and empirical evaluation.	maybe	The study does involve active creation of synthetic data, but it's focused on synthetic fluoroscopic images rather than tabular data.	maybe	While the study does use AI methods (deep learning) for synthetic data generation, it's specifically for imaging data, not tabular data.	yes	The study demonstrates direct clinical applications in surgical navigation and planning.	yes	The study includes original research with clear empirical evaluation and metrics.	yes	The study reports quantifiable outcomes (84% F1 score, 81.1ms computational time).	Exclude	The study focuses on surgical navigation using AI-generated synthetic fluoroscopic images, which fundamentally conflicts with the requirement for tabular data generation. Despite demonstrating strong clinical applications and empirical evaluation, the imaging-based approach disqualifies the research from meeting the core screening criteria. The paper's technical innovation and clinical relevance are overshadowed by its non compliance with the specified data format requirements.	1.4	Exclude	Exclude	No tabular data
Domain agnostic 2D-3D deformable registration Application to fluoroscopic guidance without contrast agent	Lecomte-Denis F., Verde J., Dillenseger J.-L., Cotin S.	2025	maybe	The paper focuses on clinical applications (percutaneous needle insertions) rather than purely technical aspects, but the primary focus appears to be on the technical methodology.	no	The study deals with fluoroscopic images and displacement fields, not tabular data.	maybe	The study involves fluoroscopic imaging which implies human clinical data, though this isn't explicitly stated.	yes	This appears to be an original research article presenting a novel method.	maybe	The study does create synthetic data using AI (Digitally Reconstructed Radiographs), but this is for training rather than the primary focus of the study.	no	While synthetic data is used, it's not tabular data generation using AI methods like GANs or VAEs. The synthetic data is in the form of images.	yes	The study demonstrates direct clinical application in percutaneous needle insertions.	maybe	The study includes empirical evaluation of the method's performance, though specific metrics aren't detailed in the abstract.	maybe	While the abstract mentions "accurate prediction," it doesn't explicitly report quantifiable metrics.	Exclude	The study focuses on a technical methodology for 3D displacement field estimation using fluoroscopic images, which fundamentally differs from tabular data generation. Critical screening criteria related to data format and synthetic data generation are not met, primarily due to the image-based nature of the research. While demonstrating clinical potential in percutaneous needle insertions, the paper does not align with the systematic review's specific requirements for tabular data and AI-driven synthetic data generation.	1.4	Exclude	Exclude	No tabular data
Domain randomization using synthetic electrocardiograms for training neural networks	Kaisti M., Laitala J., Wong D., Airola A.	2023	maybe	The paper focuses on clinical applications (ECG analysis and cardiac disease classification) rather than purely technical aspects, though it has a strong methodological component.	no	The study focuses on ECG data, which is time-series data, not tabular data, violating the inclusion criteria.	yes	The study uses human clinical data (real ECG recordings) as its basis.	yes	This appears to be an original research article with novel methodology and empirical evaluation.	yes	The study involves active creation of synthetic data using neural networks and domain randomization.	yes	The study uses AI methods (neural networks) to generate synthetic data, though the exact method (GAN, VAE, etc.) isn't specified.	yes	The study demonstrates direct clinical applications in ECG analysis and cardiac disease classification.	yes	The study includes original research with clear empirical evaluation and metrics for assessing synthetic data's utility.	yes	The study reports quantifiable outcomes in terms of model performance and detection accuracy.	Exclude	The study involves synthetic ECG data generation using neural networks, demonstrating clinical applications in cardiac disease classification with quantifiable performance metrics. However, the research focuses on time-series ECG data rather than tabular data, which critically disqualifies it from meeting the systematic review's screening criteria. Despite strong methodological rigor and empirical evaluation, the data format represents a fundamental exclusion factor.	1.4	Exclude	Exclude	No tabular data
DRSegNet: A cutting-edge approach to Diabetic Retinopathy segmentation and classification using parameter-aware Nature-Inspired optimization	Kamal S.A., Du Y., Khalid M., Farrash M., Dhelim S.	2024	yes	The study focuses on clinical applications, specifically diabetic retinopathy diagnosis, rather than purely technical, privacy, methodological or educational aspects.	maybe	The study appears to focus on medical imaging data (retinal images) rather than tabular data, as explicitly mentioned in the context of "medical image analysis" and the use of FCEDN (Fully Convolutional Encoder-Decoder Networks).	yes	The study uses human clinical data, specifically the IDRiD dataset for diabetic retinopathy.	yes	This appears to be an original research article with novel methodology and results.	yes	The study actively creates synthetic data using GANs, as explicitly stated: "using Generative Adversarial Networks (GANs) to generate high-quality synthetic data."	yes	The study uses AI methods (specifically GANs) for synthetic data generation.	yes	The study demonstrates direct clinical application in diagnosis of diabetic retinopathy.	yes	The study includes empirical evaluation with clear metrics and substantial evaluation plan.	yes	The study reports clear quantifiable outcomes (99.87% accuracy, 99.33% sensitivity, 99.78% specificity).	Exclude	The study focuses on diabetic retinopathy diagnosis using synthetic data generation and AI techniques, demonstrating strong clinical application and quantifiable outcomes. However, the research primarily involves medical imaging data rather than tabular data, which disqualifies it from meeting the specific screening criteria. Despite robust methodology and high-performance metrics, the data format prevents the study from being included in the review.	1.4	Exclude	Exclude	No tabular data
ECG synthesis for cardiac arrhythmias: Integrating self-supervised learning and generative adversarial networks.	Simone, Lorenzo, Bacciu, Davide, Gervasi, Vincenzo	2025	yes	The study focuses on clinical applications, specifically arrhythmia classification using ECG data, which is a clinical application rather than purely technical.	maybe	The study deals with ECG time series data, which is explicitly excluded in the criteria as it's not tabular data.	yes	The study uses human clinical data from established ECG datasets (MIT-BIH, BIDMC, PTB).	yes	This appears to be an original research article with novel methodology and empirical evaluation.	yes	The study actively creates synthetic data using their proposed ECGAN model, which is a generative AI approach.	yes	The study uses AI methods (specifically a conditional generative architecture with GAN principles) to generate synthetic data.	yes	The study demonstrates direct clinical application in arrhythmia classification and diagnosis.	yes	The study includes clear empirical evaluation with specific metrics and comparison to state-of-the-art models.	yes	The study reports quantifiable outcomes, specifically a 2.4% improvement in arrhythmia classification accuracy.	Exclude	The study presents a novel AI-driven approach for generating synthetic ECG data with clinical applications in arrhythmia classification, demonstrating strong empirical validation and quantifiable improvements in diagnostic accuracy. However, the research focuses on time series data, which explicitly violates the screening criteria's requirement for tabular data generation. Despite meeting most other screening requirements, this fundamental deviation disqualifies the study from further consideration.	1.4	Exclude	Exclude	No tabular data

Efficient GAN-based Chest Radiographs (CXR) augmentation to diagnose coronavirus disease pneumonia.	Albahli, Saleh	2020	maybe	The paper focuses on clinical applications (COVID-19 detection from chest images), but it's primarily an imaging study rather than focusing on tabular data generation.	no	The study deals with chest X-Ray/CT images, not tabular data.	yes	The study uses human clinical data (chest images from COVID-19 patients), meeting this criterion.	yes	This appears to be an original research article with methods and results, meeting this criterion.	yes	The study does use GAN-based synthetic data generation, which meets this criterion.	yes	The study uses GANs for synthetic data generation, meeting this criterion.	yes	The study demonstrates direct clinical application in COVID-19 detection, meeting this criterion.	yes	The study includes empirical evaluation with clear metrics, meeting this criterion.	yes	The study reports quantifiable outcomes (89% accuracy), meeting this criterion.	Exclude	The study focuses on COVID-19 detection using chest imaging and GAN-based synthetic data generation, demonstrating strong clinical application and empirical evaluation. However, the research fundamentally fails the screening criteria due to its use of imaging data instead of tabular data, rendering it ineligible for inclusion despite meeting multiple other requirements.	1.4	Exclude	Exclude	No tabular data
Efficient Photoacoustic Image Synthesis with Deep Learning.	Rix, Tom, Dreher, Kris K, Nolke, Jan-Hinrich, Schellenberg, Melanie, Tizabi, Minu D, Seitel, Alexander, Maier-Hein, Lena	2023	no	The paper focuses on technical aspects of photoacoustic imaging and synthetic data generation for imaging, rather than direct clinical applications. While it mentions "clinical application" potential, the focus is primarily methodological.	no	The study deals with imaging data (photoacoustic images), not tabular data. This is explicitly clear from the abstract's focus on image synthesis and visualization.	maybe	The study uses human forearm images, but primarily for validation rather than as the basis for synthetic data generation.	yes	This appears to be an original research article with novel methodology and empirical evaluation.	yes	The study does involve active creation of synthetic data using AI (deep learning methods), specifically for photoacoustic image synthesis.	maybe	While the study does use AI methods (neural networks) for synthetic data generation, it's for imaging data rather than tabular data.	no	While the paper mentions potential clinical applications, it doesn't demonstrate direct clinical applications. The focus is on the technical methodology.	yes	The study includes original research with clear empirical evaluation and metrics for assessing the synthetic data generation method.	yes	The study reports quantifiable outcomes, specifically speed improvements (100x faster) and accuracy comparisons with Monte Carlo simulations.	Exclude	The study focuses on technical photoacoustic imaging methodology using deep learning for synthetic data generation, primarily involving imaging data rather than tabular clinical information. While demonstrating innovative AI techniques for image synthesis, the research lacks direct clinical applications and does not meet key screening criteria related to tabular data and clinical utility. The work represents a methodological exploration with potential future clinical implications, but falls short of the systematic review's specific requirements.	1.4	Exclude	Exclude	No tabular data
Employing Domain Indexes to Efficiently Query Medical Data from Multiple Repositories	Oliveira P.H., Scabora L.C., Cazzolato M.T., Oliveira W.D., Paixao R.S., Traina A.J.M., Traina C.	2019	no	The paper focuses on technical aspects (content-based retrieval and indexing) rather than clinical applications. While it's in a healthcare context, the primary focus is on data retrieval efficiency.	no	The study deals with mammogram images, not tabular data. This is explicitly mentioned in the abstract.	yes	The study uses real-world mammogram data, which is human clinical data.	yes	This appears to be an original research article presenting a new method (Domain Index) with experimental evaluation.	maybe	While the study mentions using synthetic data, it's not clear if they actively created it using AI methods. The synthetic data appears to be used for scalability testing rather than being the main focus.	maybe	The abstract doesn't specify the method used to generate synthetic data, and it's not the primary focus of the study.	no	The paper focuses on technical aspects of data retrieval rather than direct clinical applications like diagnosis or treatment planning.	no	The study includes empirical evaluation, but it's focused on technical performance metrics rather than clinical utility.	no	The study reports quantifiable outcomes, but they are related to computational performance (53% and 81% performance gains) rather than clinical metrics.	Exclude	The study primarily focuses on technical data retrieval methods for mammogram images, failing key screening criteria related to clinical applications and tabular data generation. While using real-world clinical data and presenting original research, the paper does not demonstrate direct clinical utility or synthetic data generation using AI methods. The research is fundamentally a technical optimization study rather than a clinically-oriented investigation.	1.4	Exclude	Exclude	No tabular data
End-to-end trained encoder decoder convolutional neural network for fetal electrocardiogram signal denoising.	Fotiadou, Eleni, Konopczynski, Tomasz, Hesser, Jurgen, Vullings, Rik	2020	maybe	The paper focuses on clinical applications (fetal ECG enhancement) rather than purely technical aspects, but it is primarily a technical solution to a clinical problem.	no	The study deals with ECG data, which is time-series data, not tabular data.	yes	The study uses human clinical data (fetal ECGs).	yes	This appears to be an original research article with novel methodology and results.	no	The study uses AI to enhance existing ECG signals rather than creating synthetic data from scratch. While it mentions "synthetic data" in experiments, this appears to be for testing purposes rather than the main focus.	no	While the study uses AI methods (deep convolutional network), it's not generating synthetic tabular data but rather enhancing existing ECG signals.	yes	The study has direct clinical applications in fetal monitoring and medical decision-making.	yes	The study includes empirical evaluation with clear metrics and comparison with real data.	yes	The study reports clear quantifiable outcomes (SNR improvements, RMSE for PR and QT intervals).	Exclude	The study focuses on fetal ECG signal enhancement using AI, but critically fails to meet key screening criteria due to its use of time-series data instead of tabular data and its emphasis on signal processing rather than synthetic data generation. While demonstrating clinical relevance and empirical evaluation, the fundamental methodological approach disqualifies it from the systematic review's specific requirements.	1.4	Exclude	Exclude	No tabular data
Enhancement of Fairness in AI for Chest X-ray Classification.	Jackson, Nicholas J, Yan, Chao, Malin, Bradley A	2024	yes	The study focuses on clinical applications, specifically improving fairness in diagnostic AI for chest X-ray interpretation, which is a clinical application rather than purely technical.	maybe	The study uses chest X-ray images (MIMIC-CXR dataset), not tabular data, which is a critical failure of a core criterion.	yes	The study uses real clinical data from MIMIC-CXR, which is a dataset of human chest X-rays.	yes	This appears to be an original research article with empirical evaluation, not a review or commentary.	yes	The study involves active creation of synthetic data through augmentation, as evidenced by "synthetic data augmentation" being one of the methods tested.	yes	The study uses AI methods for synthetic data generation, though the specific method (GAN, VAE, etc.) isn't specified in the abstract.	yes	The study demonstrates direct clinical application in diagnostic imaging and fairness in medical decision-making.	yes	The study includes clear empirical evaluation with specific metrics and confidence intervals.	yes	The study reports quantifiable outcomes including AUC scores and percentage reduction in disparities (74.7% and 10.6%).	Exclude	The study demonstrates strong clinical relevance and empirical rigor in addressing AI bias in medical imaging, with comprehensive quantifiable outcomes and original research methodology. However, the research fundamentally fails to meet the screening criteria due to its focus on imaging data rather than tabular data generation, which represents a critical disqualifying factor. Despite meeting most other screening requirements, this core deviation prevents the study from being considered within the specified research parameters.	1.4	Exclude	Exclude	No tabular data
Enhancing classification of cells procured from bone marrow aspirate smears using generative adversarial networks and sequential convolutional neural network	Hazra D., Byun Y.-C., Kim W.J.	2022	maybe	The study focuses on technical aspects of image classification and synthetic data generation, rather than direct clinical applications. While it's in a medical context, the primary focus is on the technical methodology.	maybe	The study deals with microscopic cell images, not tabular data. This is explicitly clear from the abstract's focus on "microscopic cell images" and image classification.	yes	The study uses human clinical data from bone marrow aspirate smears.	yes	This appears to be an original research article with novel methodology and results.	yes	The study actively creates synthetic data using a novel GAN architecture (C-WGAN-GP).	yes	The study uses AI methods (specifically GANs) for synthetic data generation.	maybe	While the study is in a medical context, it doesn't demonstrate direct clinical applications. It focuses on improving classification accuracy rather than direct clinical decision-making.	yes	The study includes original research with clear empirical evaluation and metrics.	yes	The study reports clear quantifiable outcomes including classification accuracy (96.98%), sensitivity, and specificity metrics.	Exclude	The study focuses on synthetic data generation for microscopic cell image classification using GANs, but fails key screening criteria by not addressing direct clinical applications and using image data instead of tabular data. Despite demonstrating high-quality technical methodology and empirical evaluation, the research does not meet the fundamental requirements for clinical-focused synthetic data research.	1.4	Exclude	Exclude	No tabular data
Enhancing Left Ventricular Segmentation in Echocardiograms Through GAN-Based Synthetic Data Augmentation and MultiResUNet Architecture	Kumar V., Sharma N.M., Mahapatra P.K., Dogra N., Maurya L., Ahmad F., Dahiya N., Panda P.	2025	no	The study focuses on technical aspects (image segmentation) rather than direct clinical applications, even though it has clinical implications. The primary focus is on the technical methodology rather than clinical applications.	no	The study deals with imaging data (echocardiograms) rather than tabular data, as explicitly stated in the abstract's focus on image segmentation.	yes	The study uses human clinical data (echocardiograms) from the EchoNet-Dynamic dataset.	yes	This appears to be an original research article with clear methodology and results.	yes	The study actively creates synthetic data using GANs, as explicitly stated in the methodology.	yes	The study uses GANs for synthetic data generation, though for images rather than tabular data.	maybe	While the study has clinical implications for cardiovascular disease diagnosis and monitoring, it focuses on the technical aspect of image segmentation rather than direct clinical applications.	yes	The study includes original research with clear empirical evaluation and metrics.	yes	The study reports clear quantifiable outcomes (Dice Similarity Coefficient of 95.68% and IoU of 91.62%).	Exclude	The study focuses on technical image segmentation using GANs for echocardiogram analysis, which deviates from the required tabular data format and direct clinical application criteria. Despite demonstrating sophisticated AI-driven synthetic data generation and reporting quantifiable outcomes, the research fails to meet key screening requirements related to data type and clinical focus. Consequently, the paper does not align with the systematic review's core screening parameters.	1.4	Exclude	Exclude	No tabular data
Enhancing Patient-Trial Matching With Large Language Models: A Scoping Review of Emerging Applications and Approaches.	Chen, Hongyu, Li, Xiaohan, He, Xing, Chen, Aokun, McGill, James, Webber, Emily C, Xu, Hua, Liu, Mei, Bian, Jiang	2025	no	The paper focuses on clinical applications (patient-trial matching), but it's a review of LLM applications rather than a primary study of synthetic data generation.	no	The abstract doesn't explicitly discuss synthetic data generation or tabular data formats. While patient data is mentioned, the focus is on LLM applications.	maybe	The study involves human clinical data in the context of patient-trial matching, but as a review rather than primary research.	no	This is explicitly stated to be a "scoping review" of 24 studies, not an original research article.	no	The abstract mentions "reliance on synthetic data sets" but doesn't describe active creation of synthetic data. Instead, it appears to be reviewing existing systems that use synthetic data.	no	While synthetic data is mentioned, the paper doesn't focus on the generation of synthetic data using AI methods. It's about LLM applications in trial matching.	maybe	The paper discusses clinical applications (patient-trial matching), but as a review of existing systems rather than demonstrating direct applications.	no	As a review paper, it doesn't include original empirical evaluation.	no	The abstract mentions "improved accuracy and scalability" but as findings from reviewed studies rather than original research outcomes.	Exclude	A scoping review of large language models in patient-trial matching fails to meet multiple screening criteria, particularly those related to original research, synthetic data generation, and empirical evaluation. While the study addresses clinical applications and involves human clinical data, its review nature and focus on LLM applications rather than synthetic data creation disqualify it from meeting the specified screening requirements.	1.4	Exclude	Exclude	Wrong publication type
Enhancing robustness and generalization in microbiological few-shot detection through synthetic data generation and contrastive learning.	Ebert, Nikolas, Stricker, Didier, Wasenmuller, Oliver	2025	maybe	The paper focuses on clinical applications (hygiene monitoring and colony detection) rather than purely technical aspects, but it is more focused on the technical implementation than clinical outcomes.	no	The study deals with image data (agar plates and bacterial colonies), not tabular data.	no	The study uses agar plate images and bacterial colonies, not human clinical data or patient-related healthcare information.	yes	This appears to be an original research article with novel methodology and empirical results.	yes	The study does involve active creation of synthetic data using AI (diffusion-based generator model).	no	While the study does use AI for synthetic data generation, it's for images rather than tabular data.	maybe	While the application is in hygiene monitoring, which has clinical relevance, it's not directly focused on diagnosis, prognosis, or treatment planning.	yes	The study includes original research with clear empirical evaluation and metrics.	yes	The study reports quantifiable outcomes (mAP scores, AP50 score).	Exclude	The study focuses on image-based bacterial colony detection using AI-generated synthetic data, which fundamentally differs from the required tabular data format. Critical screening criteria are not met, particularly the requirements for human clinical data and tabular data generation. Despite demonstrating technical innovation in synthetic data creation, the research falls outside the specified systematic review parameters.	1.4	Exclude	Exclude	No tabular data
Escarcitys: A framework for enhancing medical image classification performance in scarcity of trainable samples scenarios.	Wang, Tianxiang, Dai, Qun, Xiong, Wei	2025	maybe	The paper focuses on medical image analysis and disease diagnosis, which is clinical in nature. However, it appears to be primarily focused on the technical aspects of image generation and classification, rather than purely clinical applications.	no	The study explicitly deals with medical images, not tabular data, which is a clear violation of the criterion requiring only tabular data generation.	maybe	The study mentions using "real medical image datasets" but doesn't explicitly specify if these are human clinical data. However, given the context of disease diagnosis, it's likely human clinical data.	yes	This appears to be an original research article with novel methodology and experimental validation.	yes	The study does involve active creation of synthetic data using AI (specifically a "disease probability map-guided diffusion generation model").	no	While the study does generate synthetic data using AI methods, it's specifically for medical images rather than tabular data.	yes	The study focuses on disease diagnosis, which is a direct clinical application.	yes	The study includes empirical evaluation on "four real medical image datasets" with clear validation of their approach.	maybe	The abstract mentions conducting "detailed experiments" and validating "effectiveness," suggesting quantifiable outcomes, though specific metrics aren't detailed.	Exclude	The study focuses on medical image analysis and synthetic data generation using AI, but critically fails to meet key screening criteria by working with medical images instead of tabular data. Despite demonstrating strong clinical application and empirical evaluation, the fundamental mismatch in data format disqualifies the research from meeting the specified screening requirements.	1.4	Exclude	Exclude	No tabular data
Ethical Application of Generative Artificial Intelligence in Medicine.	Hasan, Sayyida S, Fury, Matthew S, Woo, Joshua J, Kunze, Kyle N, Ramkumar, Prem N	2025	no	The paper discusses clinical applications of AI, but primarily from an ethical and regulatory perspective rather than focusing on specific clinical applications. It's more of a commentary on the implications of AI in healthcare.	no	The abstract doesn't specifically discuss tabular data generation. It mentions synthetic data broadly but doesn't specify the format.	no	The paper discusses healthcare and patient data in general terms but doesn't present specific work with human clinical data.	no	This is explicitly labeled as "Level V, expert opinion" and appears to be a commentary or perspective piece rather than original research.	no	The paper doesn't describe active creation of synthetic data. It discusses synthetic data generation in general terms but doesn't present original data creation work.	no	While synthetic data is mentioned, the paper doesn't detail specific AI methods for generating synthetic data. It's discussed as a general concept rather than presenting specific implementation.	no	While clinical applications are mentioned (diagnosis, treatment planning), they're discussed theoretically rather than demonstrated through specific applications.	no	There is no empirical evaluation presented. The paper appears to be a theoretical discussion of challenges and considerations.	no	The paper doesn't report any quantifiable outcomes. It's focused on ethical and regulatory considerations.	Exclude	This expert opinion piece discusses generative AI in healthcare from an ethical and regulatory perspective, lacking original research, empirical evaluation, or specific synthetic data generation methods. The document primarily explores theoretical challenges and considerations surrounding AI's integration into clinical settings, without presenting quantifiable outcomes or demonstrating direct clinical applications. Consequently, the paper does not meet the established screening criteria for a research study on synthetic data generation in healthcare.	1.4	Exclude	Exclude	Wrong publication type

Evaluating synthetic neuroimaging data augmentation for automatic brain tumour segmentation with a deep fully-convolutional network	Asadi F., Angsuwatanakul T., O'Reilly J.A.	2024	maybe	The study focuses on technical aspects of image segmentation and synthetic data generation for training AI models, rather than direct clinical applications.	maybe	The study deals with medical imaging data (FLAIR MRI) and segmentation masks, not tabular data.	yes	The study uses real clinical neuroimaging data from human patients with gliomas.	yes	This appears to be an original research article with empirical evaluation.	yes
Evaluation of Generative Adversarial Networks for High-Resolution Synthetic Image Generation of Circumpapillary Optical Coherence Tomography Images for Glaucoma	Sreejith Kumar A.J., Chong R.S., Crowston J.G., Chua J., Bujor I., Husain R., Vithana E.N., Girard M.J.A., Ting D.S.W., Cheng C.-Y., Aung T., Popa-Cherecheanu A., Schmetterer L., Wong D.	2022	no	The study focuses on generating synthetic OCT images for training deep learning models, which is primarily a technical/methodological application rather than a direct clinical application.	no	The study deals with OCT imaging data, which is not tabular data. The abstract specifically mentions "circumpapillary OCT images" and "OCT scans."	yes	The study uses human clinical data, specifically OCT scans from normal and glaucomatous eyes.	yes	This appears to be an original research article with clear methodology and results.	yes
Examining the effect of synthetic data augmentation in polyp detection and segmentation.	Adjei, Prince Ebenezer, Lonseko, Zenebe Markos, Du, Wenju, Zhang, Han, Rao, Nini	2022	maybe	The study focuses on technical aspects of image analysis and data augmentation rather than direct clinical applications. While it's in a medical context, the focus is on improving model performance through synthetic data generation.	maybe	The study deals with medical images (colonoscopy images) rather than tabular data. This is explicitly clear from the abstract's focus on image analysis and polyp detection.	yes	The study uses colonoscopy images from human patients as its basis.	yes	This appears to be an original research article with clear methodology and results.	yes
Exceptional performance with minimal data using a generative adversarial network for alzheimer's disease classification.	Wong, Pui Ching, Abdullah, Shahrum Shah, Shapiai, Mohd Ibrahim	2024	maybe	The study focuses on technical aspects of data augmentation and classification rather than direct clinical applications. While it's in the medical domain, the primary focus is on the technical solution to data scarcity.	maybe	The study deals with MRI imaging data, not tabular data. This is explicitly stated in the abstract ("Medical image datasets", "MRI data").	yes	The study uses human clinical data from the OASIS database, which contains real patient MRI data.	yes	Based on the abstract, this appears to be an original research article with novel implementation and results.	yes
Explainable synthetic image generation to improve risk assessment of rare pediatric heart transplant rejection.	Giuste, Felipe O, Sequeira, Ryan, Keerthipati, Vikranth, Lais, Peter, Mirzazadeh, Ali, Mohseni, Arshawn, Zhu, Yuanda, Shi, Wenqi, Marteau, Benoit, Zhong, Yishan, Tong, Li, Das, Bibhuti, Shehata, Bahig, Deshpande, Shriprasad, Wang, May D	2023	yes	The study focuses on clinical applications, specifically the detection of pediatric heart transplant rejection through microscopic analysis of biopsy samples.	maybe	The study focuses on image data (digital images of biopsied tissue) rather than tabular data, which is a critical mismatch with the screening criteria.	yes	The study uses human clinical data from pediatric heart transplant patients.	yes	This appears to be an original research article with novel methodology and results.	yes
Exploring YouTube's Recommendation System in the Context of COVID-19 Vaccines: Computational and Comparative Analysis of Video Trajectories	Ng Y.M.M., Pham K.H., Luengo-Oroz M.	2023	no	The paper focuses on analyzing YouTube's recommendation algorithm and vaccine-related content, which is not primarily a clinical application. While it has health implications, it's more of a social media analysis study.	no	The study deals with video recommendations and content analysis, not tabular data generation.	no	The study does not use human clinical data or patient-related healthcare information. It analyzes YouTube video recommendations.	yes	This appears to be an original research article with empirical evaluation.	no
Fold-stratified cross-validation for unbiased and privacy-preserving federated learning.	Bey, Romain, Goussault, Romain, Grolleau, Francois, Benchoufi, Mehdi, Porcher, Raphael	2020	no	The paper focuses on a methodological aspect (fold-stratified cross-validation) rather than a clinical application. While it's related to healthcare data, it's primarily about a validation methodology.	yes	The study deals with EHRs (Electronic Health Records) which are tabular data.	yes	The study uses MIMIC-III, which is a real clinical database of human patient data.	yes	This appears to be an original research article introducing a new methodology.	no
Foundation models for radiology: fundamentals, applications, opportunities, challenges, risks, and prospects	Akinci D'Antonoli T., Bluethgen C., Cuocolo R., Klontzas M.E., Ponsiglione A., Kocak B.	2025	maybe	The paper discusses clinical applications in radiology, but appears to be a comprehensive overview rather than focusing on specific clinical applications. It mentions "image interpretation, report generation, integrative diagnostics" but seems more focused on general challenges and opportunities.	no	The paper discusses imaging data and appears to focus primarily on radiological images rather than tabular data. This is a clear violation of the criterion requiring only tabular data.	maybe	The paper discusses radiology and healthcare applications, implying human clinical data, but doesn't explicitly state whether it uses human clinical data.	no	The abstract suggests this is a comprehensive overview or review paper ("provides a comprehensive overview") rather than original research, which fails this criterion.	maybe
Free-breathing and instantaneous abdominal T2 mapping via single-shot multiple overlapping-echo acquisition and deep learning reconstruction.	Lin, Xi, Dai, Lixing, Yang, Qinqin, Yang, Qizhi, He, Hongjian, Ma, Lingceng, Liu, Jingjing, Cheng, Jingliang, Cai, Congbo, Bao, Jianfeng, Chen, Zhong, Cai, Shuhui, Zhong, Jianhui	2023	maybe	The paper focuses on clinical applications (abdominal T2 mapping for medical imaging), but it's primarily a technical/methodological paper about developing a new imaging technique. While it has clinical applications, the focus is on the technical implementation.	no	The study deals with imaging data (MRI), not tabular data. This is a clear violation of the inclusion criteria.	yes	Yes, the study uses human clinical data, including patient measurements and liver lesion analysis.	yes	Yes, this appears to be an original research article with clear methodology and results.	maybe
From local counterfactuals to global feature importance: efficient, robust, and model-agnostic explanations for brain connectivity networks.	Alfeo, Antonio Luca, Zippo, Antonio G, Catrambone, Vincenzo, Cimino, Mario G C A, Toschi, Nicola, Valenza, Gaetano	2023	maybe	The paper focuses on explainable AI for mental state classifications using fMRI data, which has clinical applications. However, the primary focus appears to be on the technical methodology (BoCSoR) rather than direct clinical applications.	no	The study uses fMRI data, which is imaging data, not tabular data.	yes	The study uses fMRI data from the Human Connect project, which involves human subjects.	yes	Yes, this appears to be an original research article presenting a new methodology (BoCSoR).	no

Evaluating synthetic neuroimaging data augmentation for automatic brain tumour segmentation with a deep fully-convolutional network	The study actively creates synthetic data using StyleGAN2-ada for generating FLAIR images and segmentation masks.	yes	The study uses AI methods (StyleGAN2-ada) for synthetic data generation.	maybe	While the ultimate goal is clinical (glioma evaluation), the study focuses on the technical aspects of image segmentation rather than direct clinical applications like diagnosis or treatment planning.	yes	The study includes clear empirical evaluation with specific metrics and validation/test sets.	yes	The study reports quantifiable outcomes (Dice coefficients, computational metrics).	Exclude	The study focuses on technical image segmentation using synthetic data generation for glioma imaging, which fundamentally diverges from the screening criteria's requirements for clinical applications and tabular data. Despite demonstrating sophisticated AI techniques and empirical evaluation, the research fails key screening criteria by emphasizing methodological aspects of image processing rather than direct clinical utility. Consequently, the paper does not align with the specified review parameters.	1.4	Exclude	Exclude	No tabular data
Evaluation of Generative Adversarial Networks for High-Resolution Synthetic Image Generation of Circumpapillary Optical Coherence Tomography Images for Glaucoma	The study actively creates synthetic data using generative adversarial networks (GANs), specifically "Progressively growing generative adversarial network models."	yes	The study uses AI methods (GANs) to generate synthetic data, though for images rather than tabular data.	maybe	While the ultimate goal is glaucoma detection, the study focuses on the technical aspect of generating synthetic images rather than direct clinical applications.	yes	The study includes thorough empirical evaluation with clear metrics and comparison between synthetic and real image performance.	yes	The study reports clear quantifiable outcomes including AUC scores for glaucoma detection and expert evaluation metrics.	Exclude	The study focuses on generating synthetic OCT images using generative adversarial networks for glaucoma detection, which primarily represents a technical methodology rather than a direct clinical application. While the research demonstrates sophisticated AI techniques and uses human clinical data, it fails key screening criteria by dealing with imaging data instead of tabular data and emphasizing technical aspects over clinical utility. The paper's approach, though innovative, does not align with the specified screening requirements for clinical and tabular data generation.	1.4	Exclude	Exclude	No tabular data
Examining the effect of synthetic data augmentation in polyp detection and segmentation.	The study actively creates synthetic data using a pix2pix model, which is a type of GAN.	yes	The study uses AI methods (specifically a pix2pix model) to generate synthetic data.	maybe	While the study is in a medical context, it focuses on improving model performance for polyp detection rather than direct clinical applications like diagnosis or treatment planning.	yes	The study includes original research with clear empirical evaluation and metrics.	yes	The study reports clear quantifiable outcomes including F1 score, intersection over union, precision, and recall.	Exclude	The study focuses on generating synthetic colonoscopy images using AI techniques, which fundamentally conflicts with the requirement for tabular data generation. Despite demonstrating strong methodological rigor and empirical evaluation, the research fails to meet critical screening criteria related to data format and clinical application focus. Consequently, the paper does not align with the specified screening requirements.	1.4	Exclude	Exclude	No tabular data
Exceptional performance with minimal data using a generative adversarial network for alzheimer's disease classification.	The study actively creates synthetic data using GANs, as clearly stated in the abstract.	yes	The study uses GANs for synthetic data generation, which is explicitly mentioned.	maybe	While the study is about AD classification, it focuses on the technical aspects of improving classification accuracy rather than demonstrating direct clinical applications or decision-making.	yes	The study includes empirical evaluation with clear metrics for assessing the synthetic data's utility.	yes	The study reports quantifiable outcomes (classification accuracy above 80%).	Exclude	The study focuses on technical aspects of Alzheimer's disease classification using GANs for MRI data augmentation, which fails key screening criteria related to tabular data and direct clinical applications. Despite demonstrating synthetic data generation and reporting quantifiable outcomes, the research primarily addresses methodological challenges rather than clinical utility, rendering it unsuitable for the systematic review.	1.4	Exclude	Exclude	No tabular data
Explainable synthetic image generation to improve risk assessment of rare pediatric heart transplant rejection.	The study involves active creation of synthetic data using AI (specifically mentions "generative adversarial network models" and "synthetic data augmentation").	maybe	While the study does use AI for synthetic data generation, it's generating synthetic images rather than tabular data.	yes	The study demonstrates direct clinical application in diagnosis and early detection of transplant rejection.	yes	The study includes original research with clear empirical evaluation and metrics.	yes	The study reports clear quantifiable outcomes (AUROC of 98.84% and 95.56%, biopsy-level sensitivity of 83.33%).	Exclude	The study demonstrates a sophisticated AI approach for detecting pediatric heart transplant rejection using synthetic image data generation, with strong clinical applications and quantifiable outcomes. However, the research fundamentally deviates from the screening criteria by focusing on image-based analysis rather than tabular data generation, which represents a critical disqualifying factor. Despite meeting most other criteria with high-quality methodology and empirical results, the data format mismatch prevents the study from satisfying the core screening requirements.	1.4	Exclude	Exclude	No tabular data
Exploring YouTube's Recommendation System in the Context of COVID-19 Vaccines: Computational and Comparative Analysis of Video Trajectories	While the study uses synthetic data collection methods, these are for simulating user interactions rather than creating synthetic clinical data.	no	The synthetic data used is for simulating user interactions with YouTube's algorithm, not for generating synthetic clinical tabular data.	no	While the study has implications for public health, it does not demonstrate direct clinical applications like diagnosis, prognosis, or treatment planning.	maybe	The study includes original research with clear empirical evaluation and metrics, but not for clinical utility.	maybe	The study reports quantifiable outcomes (percentage of antivaccine videos, recommendation patterns), but these are not clinical metrics.	Exclude	The study focuses on YouTube's recommendation algorithm and vaccine-related content, which does not align with clinical data generation or direct healthcare applications. Despite being an original research article with empirical evaluation, the work fails to meet key screening criteria related to clinical data, tabular data generation, and synthetic data creation for healthcare purposes. The research is primarily a social media analysis that falls outside the systematic review's clinical focus.	1.4	Exclude	Exclude	No tabular data
Fold-stratified cross-validation for unbiased and privacy-preserving federated learning.	The paper does not involve creating synthetic data using AI. While it mentions using synthetic data in simulations, it's not about generating synthetic data.	no	The paper uses synthetic data in simulations but does not generate synthetic data using AI methods.	no	The paper focuses on a methodological improvement for cross-validation rather than direct clinical applications.	no	While the paper includes empirical evaluation, it's focused on the methodology's performance rather than clinical utility.	no	The study reports on methodological performance metrics rather than clinical outcomes.	Exclude	The study presents a methodological approach to cross-validation in electronic health records, focusing on preventing data leakage through fold-stratified techniques. While using real clinical data and demonstrating empirical evaluation, the research does not involve AI-generated synthetic data or direct clinical applications. The paper primarily addresses a technical methodology for data validation rather than meeting the specific screening criteria for synthetic data generation and clinical utility.	1.4	Exclude	Exclude	No synthetic data generation
Foundation models for radiology: fundamentals, applications, opportunities, challenges, risks, and prospects	While the paper mentions "synthetic data creation" as one application, it's not clear if the paper itself involves active creation of synthetic data. It appears to be more of an overview paper.	maybe	The paper mentions synthetic data creation as one potential application of FMs, but doesn't appear to focus specifically on synthetic tabular data generation using AI methods.	maybe	While clinical applications are mentioned, the paper appears to be discussing potential applications rather than demonstrating direct clinical applications.	no	The abstract doesn't indicate any original empirical evaluation or clear metrics for assessing synthetic data's clinical utility.	no	The abstract doesn't mention any specific quantifiable outcomes or metrics, suggesting this is more of an overview paper.	Exclude	The abstract describes a comprehensive overview of foundation models in radiology, focusing on imaging data and potential applications rather than original research with synthetic tabular data generation. Multiple screening criteria are not met, including the requirement for tabular data, original research, and empirical evaluation with quantifiable outcomes. The paper appears to be a review discussing potential opportunities and challenges in AI applications, which disqualifies it from the systematic review.	1.4	Exclude	Exclude	Wrong publication type
Free-breathing and instantaneous abdominal T2 mapping via single-shot multiple overlapping-echo acquisition and deep learning reconstruction.	The study does use synthetic data generation, but it's for training their deep learning model rather than being the primary focus of the research.	maybe	While the study does use synthetic data generation ("synthetic data generation flow based on Bloch simulation"), it's not the main focus and appears to be using traditional simulation methods rather than AI-based synthetic data generation.	yes	The study demonstrates direct clinical applications in abdominal imaging and lesion detection.	yes	Yes, the study includes empirical evaluation with clear metrics and both simulation and in vivo experiments.	yes	Yes, the study reports clear quantifiable outcomes including NMSE, R2 values, Pearson's correlation coefficient, and T2 values for different tissue types.	Exclude	The study focuses on a technical imaging method using MRI, which primarily involves imaging data rather than tabular data, thus failing key screening criteria. While the research demonstrates clinical applications and uses synthetic data generation, its core methodology centers on developing an imaging technique rather than AI-driven synthetic data creation. Consequently, the paper does not align with the specified systematic review requirements.	1.4	Exclude	Exclude	No tabular data
From local counterfactuals to global feature importance: efficient, robust, and model-agnostic explanations for brain connectivity networks.	While the study mentions using synthetic data, it appears to be using existing synthetic datasets rather than actively creating new synthetic data.	no	The abstract mentions using synthetic data but doesn't specify the generation method. It appears to be using existing synthetic data rather than generating new synthetic data using AI methods.	maybe	While the study has potential clinical applications in mental state classification, it focuses more on the technical aspects of explainability rather than direct clinical applications.	maybe	The study includes empirical evaluation, but focuses on technical performance rather than clinical utility.	maybe	The study reports on computational efficiency and robustness to feature correlation, but these are technical metrics rather than clinical outcomes.	Exclude	The study focuses on explainable AI for mental state classification using fMRI data, which primarily involves technical methodology rather than direct clinical applications. While the research uses human clinical data and presents an original approach, it fails key screening criteria by utilizing imaging data instead of tabular data and not actively generating synthetic data. The technical nature of the research and its focus on computational efficiency rather than clinical outcomes further limit its alignment with the specified screening requirements.	1.4	Exclude	Exclude	No tabular data

Gan-based data augmentation to improve breast ultrasound and mammography mass classification	Jimenez-Gaona Y., Carrion-Figueroa D., Lakshminarayanan V., Jose Rodriguez-Alvarez M.	2024	no	The study focuses primarily on technical aspects of GAN performance and image quality metrics rather than clinical applications. While it's in a clinical domain (breast cancer), the emphasis is on the technical methodology.	no	The study deals with medical imaging data (mammography and ultrasound), not tabular data. This is explicitly clear from the abstract mentioning ROIs and image quality metrics.	yes	The study uses real medical imaging data from human patients for breast cancer diagnosis.	yes	This appears to be an original research article with novel findings and empirical evaluation.	yes	The study actively creates synthetic data using various GAN methods (WGAN-GP, Cycle GAN, Conditional GAN, SNGAN).	yes	The study clearly uses AI methods (specifically GANs) for synthetic data generation.	maybe	While the study is in the clinical domain of breast cancer diagnosis, it focuses more on the technical aspects of data augmentation rather than direct clinical applications.	yes	The study includes thorough empirical evaluation with multiple metrics and clear assessment of synthetic data quality.	yes	The study reports clear quantifiable outcomes including classification accuracy and various image quality metrics.	Exclude	The study focuses on technical aspects of synthetic data generation for breast cancer imaging using GANs, primarily examining image quality and classification performance. While demonstrating sophisticated AI techniques and empirical evaluation, the research fails key screening criteria by using imaging data instead of tabular data and emphasizing methodological rather than direct clinical applications. Consequently, the study does not align with the specified screening requirements for clinical data research.	1.4	Exclude	Exclude	No tabular data
GAN-based synthetic FDG PET images from T1 brain MRI can serve to improve performance of deep unsupervised anomaly detection models	Zotova D., Pinon N., Trombetta R., Bouet R., Jung J., Lartizien C.	2025	no	The study focuses primarily on technical aspects of image translation and generation, rather than direct clinical applications. While it has clinical implications, the primary focus is on the technical methodology.	no	The study deals with medical imaging data (PET and MRI), not tabular data. This is explicitly clear from the abstract's focus on image translation.	yes	The study uses human clinical data, specifically brain PET and MRI images from both normal subjects and epilepsy patients.	yes	This appears to be an original research article with clear methodology and results.	yes	The study actively creates synthetic data using GAN-based frameworks, which is clearly described in the methods section.	yes	The study uses AI methods (specifically GANs) for synthetic data generation, though for images rather than tabular data.	maybe	While the study has clinical implications for epilepsy lesion detection, the primary focus is on the technical aspects of image translation rather than direct clinical applications.	yes	The study includes clear empirical evaluation with specific metrics and comparison between real and synthetic data.	yes	The study reports quantifiable outcomes including SSIM, PSNR values, and sensitivity metrics (74% sensitivity).	Exclude	The study focuses on medical image translation using GANs, generating synthetic PET images from MRI data, but fails key screening criteria by working with imaging data instead of tabular data. While demonstrating technical innovation in synthetic data generation and showing clinical potential for epilepsy lesion detection, the research does not meet the fundamental requirements of tabular data analysis and direct clinical application focus.	1.4	Exclude	Exclude	No tabular data
Generalizable medical image enhancement using structure-preserved diffusion models	Chen L., Yu X., Lin H., Niu K., Li H.	2025	maybe	The paper focuses on medical image enhancement for clinical applications, which is primarily technical rather than clinical. While it has clinical implications, the focus is on the technical methodology of image enhancement.	no	The study deals with medical images, not tabular data. This is a clear violation of the criterion.	maybe	The study uses medical imaging data, which implies human clinical data, though this isn't explicitly stated in the abstract.	yes	This appears to be an original research article with novel methodology and experimental results, meeting this criterion.	yes	The study does involve active creation of synthetic data using AI (diffusion models), meeting this criterion.	no	While the study does use AI methods (diffusion models) for data generation, it's focused on image data rather than tabular data, failing the criterion.	maybe	While the study has clinical applications in image enhancement, it doesn't directly demonstrate clinical applications like diagnosis or treatment planning. It's more focused on the technical enhancement process.	yes	The study includes empirical evaluation with experiments on four datasets, meeting this criterion.	maybe	The abstract mentions that GEDM "outperformed state-of-the-art methods in image enhancement," suggesting quantifiable outcomes, though specific metrics aren't detailed.	Exclude	The study focuses on medical image enhancement using AI techniques, primarily addressing technical methodological challenges rather than direct clinical applications. While the research involves synthetic data generation and empirical evaluation, it fundamentally fails to meet key screening criteria, particularly the requirement for tabular data format. Consequently, the paper does not align with the systematic review's core requirements.	1.4	Exclude	Exclude	No tabular data
Generating and evaluating synthetic data in digital pathology through diffusion models.	Pozzi, Matteo, Noei, Shahryar, Robbi, Erich, Cima, Luca, Moroni, Monica, Munari, Enrico, Torresani, Evelin, Jurman, Giuseppe	2024	no	The study focuses on technical aspects of synthetic data generation and evaluation methodology rather than clinical applications. While it's in the medical domain, it's primarily about the generation and evaluation pipeline.	no	The study deals with Whole Slide Images (WSIs) and image tiles, not tabular data. This is explicitly mentioned in the abstract.	yes	The study uses human clinical data (GTEx dataset of Whole Slide Images).	yes	This appears to be an original research article presenting a new pipeline and methodology.	yes	The study involves active creation of synthetic data using AI (specifically diffusion models).	yes	The study uses AI methods (diffusion models) for synthetic data generation.	no	The study doesn't demonstrate direct clinical applications. It focuses on the technical aspects of generating and evaluating synthetic data for computational pathology.	yes	The study includes original research with empirical evaluation, though the metrics focus on technical aspects rather than clinical utility.	maybe	While the study mentions evaluation metrics, these are focused on data quality and similarity rather than clinical outcomes.	Exclude	The study focuses on synthetic data generation for computational pathology using diffusion models, but fails key screening criteria by working with image data instead of tabular data and lacking direct clinical applications. While demonstrating technical innovation in synthetic data creation, the research does not meet the systematic review's requirements for clinical utility and data format.	1.4	Exclude	Exclude	No tabular data
Generation of a Melanoma and Nevus Data Set from Unstandardized Clinical Photographs on the Internet	Cho S.I., Navarrete-Dechent C., Daneshjou R., Cho H.S., Chang S.E., Kim S.H., Na J.-I., Han S.S.	2023	no	The study focuses primarily on technical aspects of image data generation and AI model training, rather than direct clinical applications. While it's in the medical domain, the focus is on creating training data for AI systems.	no	The study deals with image data (dermatologic images), not tabular data. This is explicitly clear from the abstract which discusses image processing and CNNs.	yes	The study uses human clinical data (dermatologic images of melanoma and nevus).	yes	This appears to be an original research article with clear methodology and results.	yes	The study actively creates synthetic data using AI (StyleGAN2-ADA) for generating synthetic images.	no	While the study does generate synthetic data using AI (GANs), it's for images rather than tabular data.	maybe	While the ultimate goal is clinical (diagnosing melanoma), the study itself focuses on data generation rather than direct clinical applications.	yes	The study includes original research with clear empirical evaluation and metrics.	yes	The study reports quantifiable outcomes (AUROC for determining malignant neoplasms).	Exclude	The study focuses on generating synthetic dermatologic images using AI techniques, primarily addressing technical data generation rather than direct clinical applications. While the research involves human clinical data and demonstrates quantifiable outcomes, it fundamentally fails to meet key screening criteria due to its use of image data instead of tabular data. The technical nature of the study and its focus on AI training data generation disqualify it from meeting the systematic review's core requirements.	1.4	Exclude	Exclude	No tabular data
Generative adversarial network based data augmentation for CNN based detection of Covid-19.	Gulakala, Rutwik, Markert, Bernd, Stoffel, Marcus	2022	maybe	The paper focuses on clinical applications (Covid-19 diagnosis) rather than purely technical aspects, but it has a strong technical component in developing the GAN architecture.	no	The study focuses on X-ray images, not tabular data, which is a clear violation of the criterion requiring only tabular data generation.	yes	The study uses human clinical data (Covid-19 X-ray images).	yes	This appears to be an original research article with novel contributions in both GAN architecture and CNN model.	yes	The study does involve active creation of synthetic data using AI (specifically a GAN architecture).	no	While the study does use AI methods (GAN) for synthetic data generation, it's for images rather than tabular data.	yes	The study demonstrates direct clinical application in Covid-19 diagnosis.	yes	The study includes empirical evaluation with clear metrics for assessing performance.	yes	The study reports quantifiable outcomes (99.2% test accuracy).	Exclude	The study focuses on Covid-19 diagnosis using X-ray images and AI techniques, demonstrating strong clinical application and empirical evaluation. However, the research fundamentally fails to meet the screening criteria due to its use of image data instead of tabular data generation, which represents a critical disqualifying factor. Despite achieving high diagnostic accuracy and presenting novel AI methodologies, the study does not align with the specified data format requirements.	1.4	Exclude	Exclude	No tabular data
Generative adversarial network for glioblastoma ensures morphologic variations and improves diagnostic model for isocitrate dehydrogenase mutant type.	Park, Ji Eun, Eun, Dain, Kim, Ho Sung, Lee, Da Hyun, Jang, Ryoung Woo, Kim, Namkug	2021	no	The study focuses on generating synthetic medical images using GANs, which is primarily a technical/methodological application rather than a direct clinical application.	no	The study deals with medical imaging data (MRI), not tabular data. This is explicitly stated in the abstract with mentions of "synthetic images" and "MRI data."	yes	The study uses human clinical data, specifically brain MRI scans from patients with IDH-mutant glioblastomas.	yes	This appears to be an original research article with novel findings and empirical evaluation.	yes	The study actively creates synthetic data using GANs, training on real data and generating new synthetic images.	yes	The study uses GANs for synthetic data generation, which is a valid AI method.	maybe	While the study has potential clinical applications in diagnosis, it focuses more on the technical aspects of generating synthetic images rather than direct clinical decision-making.	yes	The study includes thorough empirical evaluation with validation sets and clear metrics.	yes	The study reports clear quantifiable outcomes including diagnostic accuracy (90.9% and 93.2%) and odds ratios for predictive factors.	Exclude	The study focuses on generating synthetic medical images using GANs, which primarily represents a technical methodology rather than a direct clinical application. While demonstrating sophisticated AI-driven data generation and reporting quantifiable diagnostic outcomes, the research fundamentally deals with imaging data instead of tabular data, thus failing key screening criteria. The technical nature and imaging format disqualify the study from meeting the systematic review's core requirements.	1.4	Exclude	Exclude	No tabular data
Generative adversarial networks and synthetic patient data: current challenges and future perspectives	Arora A.	2022	no	The paper appears to be a review/overview of synthetic data applications rather than focusing on specific clinical applications. While it mentions clinical research, it also covers privacy and education aspects.	maybe	The abstract doesn't specify the type of data being generated. While it mentions "datasets," it doesn't explicitly state whether these are tabular or other formats.	maybe	The paper mentions patient data, but it's unclear whether it actually uses human clinical data or just discusses the concept.	no	This appears to be a review paper rather than original research, as indicated by phrases like "This paper focuses upon three key uses" and the broad overview nature of the content.	no	The paper discusses AI-generated synthetic data but appears to be reviewing the concept rather than actively creating new synthetic data.	no	While the paper mentions generative AI and GANs, it appears to be discussing these methods generally rather than implementing them.	no	While clinical research is mentioned as one of three applications, the paper doesn't appear to demonstrate specific clinical applications.	no	There's no indication of original empirical evaluation or clear metrics for assessing synthetic data's clinical utility.	no	The abstract doesn't mention any specific quantifiable outcomes or metrics.	Exclude	The abstract describes a review paper exploring synthetic data generation in healthcare, focusing on conceptual potential rather than empirical research. While discussing AI-driven data creation across clinical research, privacy, and education domains, the text lacks specific implementation details, quantifiable outcomes, or direct clinical applications. The overview nature of the paper suggests a theoretical exploration of synthetic data's possibilities without presenting original research or concrete methodological insights.	1.4	Exclude	Exclude	Wrong publication type
Generative adversarial networks for anonymous acneic face dataset generation.	Zein, Hazem, Chantaf, Samer, Fournier, Regis, Nait-Ali, Amine	2024	no	The paper focuses primarily on technical aspects of generating synthetic facial images for acne classification, rather than direct clinical applications. While it has medical relevance, the focus is on the data generation methodology.	no	The study deals with image data (facial images), not tabular data. This is explicitly clear from the abstract's focus on "pathological human face images."	maybe	While the study uses human facial images as a basis, it's focused on generating synthetic images rather than working directly with clinical data.	yes	This appears to be an original research article with novel methodology and results.	yes	The study actively creates synthetic data using AI (StyleGAN-based algorithm), not using pre-existing synthetic datasets.	yes	The study uses AI methods (StyleGAN) for synthetic data generation, though for images rather than tabular data.	no	The study doesn't demonstrate direct clinical applications. It focuses on generating synthetic data for potential future use in classification systems.	yes	The study includes empirical evaluation with clear metrics for assessing the synthetic data's utility.	yes	The study reports quantifiable outcomes (97.6% accuracy using InceptionResNetv2).	Exclude	The study focuses on generating synthetic facial images for acne classification using AI techniques, which fundamentally deviates from the required tabular data format and lacks direct clinical application. Despite demonstrating technical innovation in synthetic data generation and reporting quantifiable outcomes, the research fails to meet critical screening criteria related to data type and clinical utility. Consequently, the paper does not align with the systematic review's core requirements.	1.4	Exclude	Exclude	No tabular data
Generative Adversarial Networks for the Creation of Realistic Artificial Brain Magnetic Resonance Images	Kazuhiro K., Werner R.A., Toriumi F., Javadi M.S., Pomper M.G., Solnes L.B., Verde F., Higuchi T., Rowe S.P.	2018	no	The study focuses primarily on technical validation of synthetic image generation rather than clinical applications. While it mentions potential clinical applications, these are not directly demonstrated.	no	The study deals with medical imaging data (MRI), not tabular data. This is explicitly clear from the abstract discussing T1-weighted brain images.	yes	The study uses real human clinical data from 30 healthy individuals and 33 patients with cerebrovascular accident.	yes	This appears to be an original research article with empirical evaluation.	yes	The study actively creates synthetic data using DCGAN, a type of AI generative model.	maybe	While the study does use AI (DCGAN) for synthetic data generation, it's for images rather than tabular data.	no	The study focuses on validating the synthetic images rather than demonstrating direct clinical applications. The clinical applications mentioned are potential future uses.	yes	The study includes clear empirical evaluation with specific metrics and validation by radiologists.	yes	The study reports quantifiable outcomes in terms of radiologist accuracy scores (e.g., "accuracy for the NRs was 0.55 and 0.30").	Exclude	The study focuses on synthetic brain MRI image generation using DCGAN, which fundamentally conflicts with the requirement for tabular data generation. While demonstrating technical innovation in AI-generated medical images, the research fails to meet critical screening criteria related to data format and direct clinical applications. Consequently, the paper does not align with the systematic review's core requirements.	1.4	Exclude	Exclude	No tabular data

Generative artificial intelligence enables the generation of bone scintigraphy images and improves generalization of deep learning models in data-constrained environments.	Haberl, David, Ning, Jing, Kluge, Kilian, Kumpf, Katarina, Yu, Josef, Jiang, Zewen, Constantino, Claudia, Monaci, Alice, Starace, Maria, Haug, Alexander R, Calabretta, Raffaella, Camoni, Luca, Bertagna, Francesco, Mascherbauer, Katharina, Hofer, Felix, Albano, Domenico, Sciagra, Roberto, Oliveira, Francisco, Costa, Durval, Nitsche, Christian, Hacker, Marcus, Spielvogel, Clemens P	2025	no	The study focuses on generating synthetic medical images (bone scintigraphy scans) rather than clinical applications. While it has clinical implications, the primary focus is on the technical generation of synthetic images.	no	The study deals with medical imaging data (bone scintigraphy scans), not tabular data. This is explicitly clear from the abstract.	yes	The study uses real human clinical data from 9,170 patients and validates on 6,448 patients across multiple sites.	yes	This appears to be an original research article with clear methodology and results.	yes	The study actively creates synthetic data using generative AI methods, training a model on real data to generate new synthetic scans.	yes	The study uses AI methods (specifically generative AI) to create synthetic data, though for images rather than tabular data.	maybe	While the study has clinical implications (detecting bone metastases and cardiac amyloidosis), the primary focus is on the data generation method rather than direct clinical application.	yes	The study includes thorough empirical evaluation with reader studies, performance metrics, and clinical outcome validation.	yes	The study reports clear quantifiable outcomes including AUC improvements (33% and 5% for different conditions) and clinical outcome associations.	Exclude	The study focuses on generating synthetic medical images using generative AI, which fundamentally deviates from the screening criteria's requirement for tabular data generation. While demonstrating robust methodology and clinical relevance, the research fails key screening requirements by concentrating on image data rather than tabular clinical information. The technical approach and empirical validation are strong, but do not align with the specified screening parameters.	1.4	Exclude	Exclude	No tabular data
Generative Artificial Intelligence Use in Healthcare: Opportunities for Clinical Excellence and Administrative Efficiency.	Bhuyan, Soumitra S, Sateesh, Vidyoth, Mukul, Naya, Galvankar, Alay, Mahmood, Asos, Nauman, Muhammad, Rai, Akash, Bordoloi, Kahuwa, Basu, Urmi, Samuel, Jim	2025	maybe	The paper discusses both clinical and non-clinical applications, but appears to be a broad overview rather than focusing specifically on clinical applications. While clinical applications are mentioned, they're not the exclusive focus.	no	The abstract mentions synthetic data generation but doesn't specify if it's tabular data. It also discusses medical images, which is explicitly excluded by the criteria.	maybe	While the paper discusses healthcare applications, it's unclear if it specifically uses human clinical data as its basis.	no	This appears to be a review or overview paper rather than original research, as it "examines various clinical and non-clinical applications" and doesn't present specific research findings.	no	While synthetic data generation is mentioned, it's unclear if the paper involves active creation of synthetic data or just discusses it as a concept. The abstract suggests this is more of an overview paper.	maybe	The paper mentions synthetic data generation, but doesn't specify the methods used (GANs, VAEs, etc.). It's mentioned as one of many applications rather than being the focus.	maybe	Clinical applications are mentioned (treatment plans, risk prediction, surgical outcomes), but the paper appears to be discussing these at a high level rather than demonstrating specific applications.	no	There's no indication of original empirical evaluation or clear metrics for assessing synthetic data's clinical utility.	no	The abstract doesn't mention any specific quantifiable outcomes or metrics. It discusses potential benefits in general terms.	Exclude	The abstract presents a broad overview of generative AI in healthcare, lacking the specific focus on tabular synthetic data generation and empirical research required by the screening criteria. Multiple aspects of the paper deviate from the desired research parameters, including its review-style approach, discussion of non-tabular data formats, and absence of quantifiable clinical outcomes. Consequently, the paper does not align sufficiently with the detailed screening requirements for inclusion.	1.4	Exclude	Exclude	Wrong publication type
Generative modeling of the Circle of Willis using 3D-StyleGAN.	Aydin, Orhun Utku, Hilbert, Adam, Koch, Alexander, Lohrke, Felix, Rieger, Jana, Tanioka, Satoru, Frey, Dietmar	2024	maybe	The study focuses primarily on technical aspects of generating synthetic medical imaging data rather than direct clinical applications. While it mentions potential clinical applications, these are future possibilities rather than the focus of the study.	maybe	The study deals with 3D medical imaging data (TOF MRA volumes) rather than tabular data. This is explicitly stated in the abstract and is a core focus of the research.	yes	The study uses human clinical data (1782 individual TOF MRA scans from 6 open source datasets).	yes	This appears to be an original research article with novel methodology and results.	yes	The study actively creates synthetic data using AI (3D StyleGANv2 architecture) rather than using pre-existing synthetic datasets.	yes	The study uses AI methods (StyleGANv2) to generate synthetic data, though for imaging rather than tabular data.	maybe	While the study mentions potential clinical applications, it focuses on the technical aspects of data generation and segmentation. The only application demonstrated is vessel segmentation, which is more of a technical task than a direct clinical application.	yes	The study includes thorough empirical evaluation with clear metrics and comparison against real data.	yes	The study reports clear quantifiable outcomes (FID, MD, AUC-PRD, Dice coefficient, Hausdorff distance).	Exclude	The study focuses on generating synthetic medical imaging data using AI, specifically 3D TOF MRA volumes of the Circle of Willis, which fundamentally differs from tabular data generation. While demonstrating technical sophistication in synthetic data creation and reporting quantifiable outcomes, the research primarily addresses methodological aspects of data generation rather than direct clinical applications. Consequently, the study does not align with the specified screening criteria for clinical tabular data research.	1.4	Exclude	Exclude	No tabular data
GluGAN: Generating Personalized Glucose Time Series Using Generative Adversarial Networks	Zhu T., Li K., Herrero P., Georgiou P.	2023	yes	The study focuses on clinical applications in diabetes management, specifically glucose prediction and automated insulin delivery, which is clearly a clinical application rather than purely technical.	maybe	The study deals with time series data from continuous glucose monitoring sensors, which is explicitly excluded in the criteria as non-tabular data.	yes	The study uses data from 47 T1D subjects across three clinical datasets.	yes	This appears to be an original research article with novel methodology and empirical evaluation.	yes	The study actively creates synthetic data using GluGAN, a GAN-based framework, rather than using pre-existing synthetic datasets.	yes	The study uses AI methods (specifically GANs with RNN modules) to generate synthetic data.	yes	The study demonstrates direct clinical applications in diabetes management, including glucose prediction and automated insulin delivery.	yes	The study includes clear empirical evaluation with multiple metrics and comparison against baseline models.	yes	The study reports quantifiable outcomes including root mean square error for predictors and various clinical metrics.	Exclude	The study presents a novel AI-driven approach for generating synthetic glucose time series data using GANs, demonstrating strong clinical applications in diabetes management with empirical evaluation across multiple datasets. However, the research focuses on time series data generation, which explicitly violates the screening criteria requiring tabular data formats. Despite meeting most other criteria with high quality, this fundamental deviation from the data format requirement leads to exclusion.	1.4	Exclude	Exclude	No tabular data
Identifying Heterogeneous Micromechanical Properties of Biological Tissues via Physics-Informed Neural Networks.	Wu, Wensi, Daneker, Mitchell, Turner, Kevin T, Jolley, Matthew A, Lu, Lu	2025	no	The paper focuses on technical aspects of material property estimation using machine learning, rather than clinical applications. While it mentions biological tissues, the focus is on mechanical properties and engineering approaches.	no	The study deals with mechanical properties and displacement/strain data, which appears to be non-tabular data. It focuses on "full-field" properties and spatial distributions.	no	While the study mentions biological tissues (brain, tricuspid valve, breast cancer), it appears to focus on mechanical properties rather than clinical patient data.	yes	This appears to be an original research article with novel methodology and results.	no	The study uses physics-informed neural networks (PINNs) but doesn't appear to create synthetic data. Instead, it uses experimental or existing data to train the models.	no	While the paper mentions "synthetic data," it's not generating synthetic tabular data using AI methods. The focus is on using PINNs to analyze existing data.	no	The paper doesn't demonstrate direct clinical applications. It focuses on mechanical property estimation for engineering purposes.	maybe	The study includes empirical evaluation with clear metrics, but these are focused on mechanical property estimation rather than clinical utility.	maybe	The study reports quantifiable outcomes (prediction accuracies and computational efficiency), but these are not clinical metrics.	Exclude	The study focuses on technical mechanical property estimation using physics-informed neural networks, primarily addressing engineering challenges rather than clinical applications. Despite mentioning biological tissues, the research lacks direct clinical utility, synthetic data generation, and patient-related healthcare information. Consequently, the paper does not meet the majority of the specified screening criteria for clinical synthetic data research.	1.4	Exclude	Exclude	No tabular data
Identifying Obviously Artificial Medical Images Produced by a Generative Adversarial Network.	O'Reilly, Jamie A, Asadi, Fawad	2022	no	The study focuses on technical aspects of synthetic image generation and validation rather than clinical applications. While it mentions "clinical relevance," the focus is on the technical challenge of identifying artificial images.	no	The study deals with medical images (CT scans), not tabular data. This is explicitly stated in the abstract and is a clear violation of the inclusion criteria.	yes	The study uses CT images from patient data as its basis.	yes	This appears to be an original research article with novel methodology and results.	yes	The study uses a progressively-grown generative adversarial network (PGGAN) to actively create synthetic data.	maybe	While the study does use AI methods (GANs) for synthetic data generation, it's for images rather than tabular data.	no	The study focuses on technical validation of synthetic images rather than direct clinical applications. The clinical relevance mentioned is future-oriented and theoretical.	yes	The study includes original empirical evaluation with clear metrics.	yes	The study reports specific metrics (accuracy of 75.5%, sensitivity of 0.743, specificity of 0.769).	Exclude	The study focuses on synthetic medical image generation using AI techniques, but fails key screening criteria by working with image data instead of tabular data and lacking direct clinical applications. While demonstrating technical innovation in synthetic image validation, the research does not meet the specified requirements for clinical focus and data format. The paper's primary contribution is a methodological approach to identifying artificial medical images, which diverges from the intended screening parameters.	1.4	Exclude	Exclude	No tabular data
Image generation by GAN and style transfer for agar plate image segmentation	Andreini P., Bonechi S., Bianchini M., Mecocci A., Scarselli F.	2020	no	The study focuses on technical aspects of image generation and segmentation rather than clinical applications. While it's in a medical context, it's primarily about the technical methodology.	no	The study deals with image data (agar plate images) rather than tabular data. This is explicitly clear from the abstract's focus on "computer vision tasks" and "image generation."	no	The study uses bacterial colony images from agar plates, not human clinical data or patient-related healthcare information.	yes	This appears to be an original research article with novel methodology and results.	yes	The study actively creates synthetic data using AI (specifically GANs) to generate synthetic bacterial colony images.	no	While the study does use AI for synthetic data generation, it's for images rather than tabular data. The method uses GANs but for image synthesis.	no	While the study has potential clinical relevance (infection recognition), it doesn't demonstrate direct clinical applications. It focuses on the technical aspects of image segmentation.	yes	The study includes empirical evaluation with clear technical metrics, though not clinical ones.	maybe	The study mentions "comparable performances" but doesn't clearly specify clinical metrics. The outcomes appear to be technical rather than clinical.	Exclude	The study focuses on technical image generation for bacterial colony segmentation using AI, which does not meet the screening criteria for clinical tabular data research. Specifically, the work involves image data, lacks direct clinical applications, and does not use human patient information, rendering it outside the systematic review's scope. While methodologically sound, the research fails to align with the required clinical and data format parameters.	1.4	Exclude	Exclude	No tabular data
Image translation for medical image generation: Ischemic stroke lesion segmentation	Platscher M., Zopes J., Federau C.	2022	no	The study focuses on technical aspects of image generation and segmentation rather than direct clinical applications. While it's in a medical context, the primary focus is on the technical methodology.	no	The study deals with medical imaging data (MRI) and segmentation masks, not tabular data. This is explicitly clear from the abstract's focus on "image-to-image translation" and "magnetic resonance images."	yes	The study uses human clinical data as its basis, specifically MRI images of stroke patients.	yes	This appears to be an original research article with novel methodology and results.	yes	The study actively creates synthetic data using AI methods, specifically using image-to-image translation models and GANs.	no	While the study does use AI methods for synthetic data generation, it's for imaging data rather than tabular data.	maybe	While the study has potential clinical applications in stroke detection, it focuses more on the technical aspects of improving segmentation algorithms rather than direct clinical applications.	yes	The study includes thorough empirical evaluation with clear metrics and comparison against clinical data.	yes	The study reports clear quantifiable outcomes, including Dice scores (72.8% vs 67.3%) and inter-reader agreement metrics.	Exclude	The study focuses on technical image generation for stroke detection using AI, primarily involving medical imaging data rather than tabular data. While demonstrating innovative synthetic data creation and empirical evaluation, the research fails to meet key screening criteria related to data format and clinical application focus. The methodology centers on improving segmentation algorithms through image-to-image translation, which diverges from the specified screening requirements.	1.4	Exclude	Exclude	No tabular data
Image-based shading correction for narrow-FOV truncated pelvic CBCT with deep convolutional neural networks and transfer learning	Rossi M., Belotti G., Paganelli C., Pella A., Barcellini A., Cerveri P., Baroni G.	2021	maybe	The paper focuses on improving CBCT image quality for radiation therapy, which is a clinical application. However, it's primarily a technical solution for image processing rather than a direct clinical application.	no	The study deals with imaging data (CBCT and CT scans), not tabular data.	yes	The study uses real-world clinical data from six different patients.	yes	This is clearly an original research article with novel methodology and results.	maybe	The study does use AI (U-Net CNN) to generate synthetic CBCT scans, but this is primarily for training purposes rather than the main focus of the study.	no	While the study does generate synthetic data using AI, it's for imaging data, not tabular data.	maybe	While the study has clinical relevance for radiation therapy, it's focused on technical image processing rather than direct clinical applications like diagnosis or treatment planning.	yes	The study includes original research with clear empirical evaluation and metrics.	yes	The study reports clear quantifiable metrics (PSNR, SSIM, HU differences, contrast-to-noise ratio).	Exclude	The study focuses on improving CBCT image quality using deep learning techniques, but primarily involves imaging data rather than tabular data generation. While demonstrating clinical relevance and empirical evaluation, the research fails key screening criteria related to data format and synthetic data generation. Consequently, the paper does not meet the systematic review's specific requirements for inclusion.	1.4	Exclude	Exclude	No tabular data
Impact of endocrine disruptors from mother's diet on immuno-hormonal orchestration of brain development and introduction of the virtual human twin tool.	Fucic, A, Mantovani, A, Vena, J, Bloom, M S, Sincic, N, Vazquez, M, Aguado-Sierra, J	2023	no	The paper appears to be primarily focused on understanding mechanisms and developing models rather than direct clinical applications. While it mentions potential correlations with diseases like autism, it seems more mechanistic/theoretical than clinically applied.	no	The abstract mentions "virtual brain models" and "multi-physics/multi-scale modelling strategies" but doesn't specifically indicate tabular data generation. The focus appears to be on complex modeling approaches rather than tabular data.	maybe	The paper discusses human populations and patient data, but primarily in the context of future modeling rather than current analysis.	no	This appears to be a review or perspective paper rather than original research, as indicated by phrases like "this paper aims to present" and the focus on describing mechanisms and future approaches.	no	While the paper mentions AI and synthetic data, it appears to be discussing future applications ("will be performed") rather than presenting original synthetic data creation.	no	The paper mentions synthetic data but in the context of future work ("will enable") rather than presenting actual synthetic data generation using AI methods.	no	While the paper touches on clinical conditions (autism), it doesn't demonstrate direct clinical applications but rather discusses mechanisms and potential future modeling approaches.	no	The paper appears to be theoretical/review-focused rather than presenting original empirical evaluation.	no	The abstract doesn't mention any specific quantifiable outcomes or metrics.	Exclude	The manuscript appears to be a theoretical review exploring potential future applications of AI and synthetic data in understanding developmental neurotoxicity, rather than presenting original research with empirical data generation. While touching on clinical conditions like autism, the paper lacks direct clinical applications, quantifiable outcomes, and actual synthetic data creation, focusing instead on describing mechanisms and future modeling strategies. The work does not meet the specified screening criteria for an original research article with synthetic data generation and clinical utility.	1.4	Exclude	Exclude	Wrong publication type

Improved detection accuracy of red lesions in retinal fundus images with superlearning approach	Sahoo M., Ghorai S., Mitra M., Pal S.	2023	maybe	The paper focuses on clinical applications (diabetic retinopathy detection), but appears to be primarily a technical/methodological paper about image processing and classification. While it has clinical relevance, the focus seems to be on the technical approach rather than clinical applications.	no	The study deals with imaging data (retinal images), not tabular data.	yes	Yes, the study uses human clinical data (retinal images from diabetic patients).	yes	Yes, this is an original research article with clear methodology and results.	no	While the paper mentions "synthetic data creation ratios," it appears to be using these for handling data imbalance rather than creating synthetic clinical data. The focus is on real image data.	no	The paper does not appear to use AI methods for synthetic data generation. The "synthetic data" mentioned is likely for data augmentation or balancing, not for generating synthetic clinical data.	maybe	While the study has clinical relevance (diabetic retinopathy detection), it appears to focus more on the technical aspects of lesion detection rather than direct clinical applications.	yes	Yes, the study includes empirical evaluation with clear metrics.	yes	Yes, the study reports quantifiable outcomes (accuracy metrics of 93.5% and 97.88%).	Exclude	The study focuses on diabetic retinopathy detection using imaging data, which fundamentally conflicts with the requirement for tabular data generation. While demonstrating clinical relevance and empirical evaluation, the research primarily addresses technical image processing methods rather than synthetic data creation for clinical applications. Consequently, the paper does not meet critical screening criteria related to data format and synthetic data generation.	1.4	Exclude	Exclude	No tabular data
Improved myelin water fraction mapping with deep neural networks using synthetically generated 3D data	Vasylechko S.D., Warfield S.K., Kurugol S., Afacan O.	2024	maybe	The paper focuses on clinical applications (myelin water fraction mapping) rather than purely technical aspects, but it is primarily focused on the technical development of the generative model.	no	The study focuses on 3D imaging data, not tabular data, which is a clear violation of the criterion as it specifically deals with MRI imaging data.	yes	The study uses human clinical data as its basis, as evidenced by the discussion of clinical practice and test-retest datasets.	yes	This appears to be an original research article with novel methodology and empirical evaluation.	yes	The study does involve active creation of synthetic data using AI (specifically a generative model combining MR physics signal decay model with probabilistic parametric T2 model).	maybe	While the study does generate synthetic data using AI methods, it's for imaging data rather than tabular data.	yes	The study demonstrates direct clinical applications in myelin water fraction mapping, which is relevant for neurological assessment.	yes	The study includes original research with clear empirical evaluation and metrics for assessing the synthetic data's utility.	yes	The study reports clear quantifiable outcomes including nRMSE (<7%) and CoV metrics.	Exclude	The study focuses on generating synthetic 3D MRI data for myelin water fraction mapping, demonstrating strong clinical applications and empirical evaluation. However, the research fundamentally fails the screening criteria by using imaging data instead of tabular data, which disqualifies it from further consideration. Despite meeting multiple other criteria, the data format requirement represents a critical exclusion point.	1.4	Exclude	Exclude	No tabular data
Improving breast mass classification by shared data with domain transformation using a generative adversarial network	Muramatsu C., Nishio M., Goto T., Oiwa M., Morita T., Yakami M., Kubo T., Togashi K., Fujita H.	2020	no	The study focuses on technical aspects of using synthetic data for training CNNs rather than direct clinical applications. While it's in a medical context, the primary focus is on the machine learning methodology.	no	The study deals with medical images (mammograms and CT scans) rather than tabular data. This is explicitly clear from the abstract mentioning "medical image dataset" and "mammograms."	yes	The study uses human clinical data (mammograms showing benign and malignant masses).	yes	This appears to be an original research article with empirical evaluation and results.	yes	The study actively creates synthetic data using a cycle generative adversarial network (cGAN) for domain transformation.	yes	The study uses AI methods (specifically cGAN) for synthetic data generation.	maybe	While the study involves breast cancer classification, which is a clinical application, the focus is more on the technical aspects of using synthetic data for training rather than direct clinical utility.	yes	The study includes original research with clear empirical evaluation and metrics.	yes	The study reports clear quantifiable outcomes including classification accuracy and AUC.	Exclude	The study focuses on synthetic data generation for medical image classification using AI techniques, but fails key screening criteria by working with medical images instead of tabular data and emphasizing technical methodology over direct clinical applications. Despite demonstrating original research with quantifiable outcomes and using human clinical data, the fundamental mismatch with required data format and application focus disqualifies the study from meeting the screening requirements.	1.4	Exclude	Exclude	No tabular data
Improving machine learning based bitewing segmentation with synthetic data	Tolstaya E., Tichy A., Paris S., Schwendicke F.	2025	no	The study focuses on technical aspects of image segmentation and synthetic data generation rather than direct clinical applications. While it has clinical relevance, the primary focus is methodological.	no	The study deals with medical imaging (bitewing radiographs) rather than tabular data. This is explicitly clear from the abstract.	yes	The study uses real dental radiographs from human patients.	yes	This appears to be an original research article with clear methodology and results.	yes	The study actively creates synthetic data using AI methods (diffusion model and GAN).	no	While the study does use AI methods for synthetic data generation, it's for images rather than tabular data.	maybe	While the study has potential clinical applications in dental imaging, it focuses more on the technical aspects of segmentation rather than direct clinical decision-making.	yes	The study includes original research with clear empirical evaluation and metrics.	yes	The study reports clear quantifiable metrics (precision, Dice score, recall, F1 score, ROC AUC).	Exclude	The study focuses on synthetic data generation for medical image segmentation, primarily addressing technical methodological challenges rather than direct clinical applications. While demonstrating sophisticated AI techniques for dental radiograph analysis, the research fundamentally deals with imaging data and technical approaches, which diverges from the specified screening criteria focused on tabular data and clinical utility. Consequently, the study does not align with the core requirements of the screening framework.	1.4	Exclude	Exclude	No tabular data
Improving prediction of blood cancer using leukemia microarray gene data and Chi2 features with weighted convolutional neural network.	Alabdulqader, Ebtisam Abdullah, Alarfaj, Aisha Ahmed, Umer, Muhammad, Eshmawi, Ala' Abdulmajid, Alsubai, Shtwai, Kim, Tai-Hoon, Ashraf, Imran	2024	yes	The study focuses on clinical applications (blood cancer diagnosis) rather than purely technical aspects. While it involves machine learning, the primary goal is clinical diagnosis.	maybe	The study uses microarray gene data, which is not tabular data in the traditional sense. Microarray data is typically high-dimensional and requires specialized processing, falling outside the scope of simple tabular data generation.	yes	The study uses human clinical data (leukemia microarray gene data from patients).	yes	This appears to be an original research article with novel methodology and empirical evaluation.	maybe	While the study uses SMOTE-Tomek to create synthetic data, this is a traditional oversampling technique, not an AI-based synthetic data generation method like GANs or VAEs.	maybe	The synthetic data generation is done using SMOTE-Tomek, which is a statistical oversampling technique, not an AI-based method as specified in the criteria.	yes	The study demonstrates direct clinical application in blood cancer diagnosis.	yes	The study includes original research with clear empirical evaluation and metrics.	yes	The study reports quantifiable outcomes, specifically 99.9% accuracy and includes k-fold cross-validation metrics.	Exclude	The study focuses on blood cancer diagnosis using machine learning and microarray gene data, demonstrating clinical application and quantifiable outcomes. However, the research does not meet key screening criteria due to its use of non-tabular data and traditional statistical oversampling techniques rather than AI-based synthetic data generation methods. Consequently, the paper would be excluded from the systematic review based on these methodological limitations.	1.4	Include	Exclude	No tabular data
Improving robustness of automatic cardiac function quantification from cine magnetic resonance imaging using synthetic image data	Gheorghita B.A., Itu L.M., Sharma P., Suciu C., Wetzl J., Geppert C., Ali M.A.A., Lee A.M., Piechnik S.K., Neubauer S., Petersen S.E., Schulz-Menger J., Chitiboi T.	2022	maybe	The paper focuses on clinical applications (cardiac function quantification from MRI), but it's primarily a technical solution to a clinical problem. While it has clinical relevance, the focus appears to be on the technical methodology.	no	The study deals with MRI imaging data, not tabular data. This clearly fails to meet the criterion which specifically excludes imaging data.	yes	The study uses human cardiac MRI data as its basis, including both healthy subjects and cases with reduced ejection fraction.	yes	This appears to be an original research article with novel methodology and results.	yes	The study actively creates synthetic data using a generative adversarial model.	yes	The study uses AI methods (GAN) to generate synthetic data.	yes	The study demonstrates direct clinical application in cardiac function quantification, specifically for measuring ejection fraction.	yes	The study includes empirical evaluation with clear metrics comparing predictions to ground truth values.	yes	The study reports quantifiable outcomes in terms of prediction accuracy compared to ground truth values.	Exclude	The study focuses on generating synthetic cardiac MRI data using AI methods, demonstrating clinical applications in cardiac function quantification with quantifiable outcomes. However, the research primarily involves imaging data, which explicitly disqualifies it from meeting the systematic review's screening criteria. Despite strong methodological rigor and clinical relevance, the imaging data format prevents inclusion.	1.4	Exclude	Exclude	No tabular data
Improving sensitivity through data augmentation with synthetic lymph node metastases for AI-based analysis of PSMA PET-CT images.	Tragardh, Elin, Ulen, Johannes, Enqvist, Olof, Edenbrandt, Lars, Larsson, Mans	2024	yes	The study focuses on clinical applications, specifically detecting lymph node metastases in prostate cancer patients using AI, rather than purely technical, privacy, methodological or educational aspects.	maybe	The study focuses on medical imaging (PET-CT images) rather than tabular data, which is a critical mismatch with the inclusion criteria.	yes	The study uses real clinical data from prostate cancer patients and compares against human readers.	yes	This is clearly an original research article with novel methodology and results.	yes	The study actively creates synthetic data using AI-based data augmentation, generating new synthetic lymph node metastases.	yes	The study uses AI methods to generate synthetic data, though the specific AI method (GAN, VAE, etc.) isn't explicitly stated.	yes	The study demonstrates direct clinical application in cancer diagnosis and detection of lymph node metastases.	yes	The study includes thorough empirical evaluation with clear metrics and comparison against both human readers and previous AI models.	yes	The study reports clear quantifiable outcomes including sensitivity (84% vs 78% vs 79%) and false positive/negative rates.	Exclude	The study demonstrates a sophisticated AI approach to detecting lymph node metastases in prostate cancer using synthetic data augmentation, with strong clinical relevance and quantifiable outcomes. However, the research focuses on medical imaging data rather than tabular data, which represents a critical deviation from the specified screening criteria. Despite meeting most other requirements with high precision, this fundamental mismatch prevents the study from fully aligning with the screening protocol.	1.4	Exclude	Exclude	No tabular data
Improving Suicidal Ideation Detection in Social Media Posts: Topic Modeling and Synthetic Data Augmentation Approach.	Ghanadian, Hamideh, Nejadgholi, Isar, Al Osman, Hussein	2025	maybe	The study focuses on clinical applications (suicidal ideation detection) rather than purely technical aspects, but it is primarily focused on social media analysis rather than direct clinical applications.	no	The study deals with text data from social media, not tabular data, which is a clear violation of the criterion.	no	The study uses social media data rather than direct clinical or patient healthcare information.	yes	This appears to be an original research article with clear methodology and results.	yes	The study does involve active creation of synthetic data using AI (GPT-3.5 Turbo), so this criterion is met.	no	While the study does use AI for synthetic data generation, it's generating text data rather than tabular data, which does not meet the criterion.	maybe	While the study has potential clinical implications for suicide detection, it's focused on social media analysis rather than direct clinical applications.	yes	The study includes original research with clear empirical evaluation and metrics.	yes	The study reports quantifiable outcomes (F1-scores of 0.87 to 0.91 and 0.70 to 0.90).	Exclude	The study focuses on social media text analysis for suicidal ideation detection, which fundamentally diverges from the required tabular clinical data format. Critical screening criteria related to data type, clinical application, and patient healthcare information are not met, rendering the research incompatible with the systematic review's core requirements. Despite methodological rigor and empirical evaluation, the paper fails to align with the essential screening parameters.	1.4	Exclude	Exclude	No tabular data
Improving unified information extraction in Chinese mental health domain with instruction-tuned LLMs and type-verification component.	Cai, Zijie, Fang, Hui, Liu, Jianhua, Xu, Ge, Long, Yunfei, Guan, Yin, Ke, Tianci	2025	maybe	The paper focuses on clinical applications (mental health information extraction) rather than purely technical aspects, but it is primarily focused on the technical framework rather than direct clinical applications.	no	The study deals with unstructured text data rather than tabular data, which clearly fails to meet the criterion for tabular data generation.	maybe	The study uses mental health-related text data, but it's unclear if this is based on real patient data or purely synthetic scenarios, so there is insufficient information to fully assess this criterion.	yes	Yes, this is an original research article with clear methodology and results.	maybe	The study does use AI (ChatGPT) to generate synthetic data, but this is for creating training data for the LLMs rather than generating synthetic clinical data for clinical use, so it does not fully meet the intended spirit of this criterion.	no	While the study does use AI to generate synthetic data, it's not generating synthetic tabular clinical data. Instead, it's generating synthetic text data for training purposes, so it does not meet the criterion.	maybe	While the study has potential clinical applications in mental health support, it doesn't demonstrate direct clinical applications in diagnosis, prognosis, or treatment planning. It's more focused on information extraction, so it only partially meets this criterion.	no	The study includes empirical evaluation, but not of clinical utility. The evaluation focuses on technical performance metrics, so it does not meet this criterion.	no	The study reports performance metrics for the information extraction task, but these aren't clinical outcomes, so it does not meet this criterion.	Exclude	The study focuses on mental health text information extraction using AI, but fundamentally diverges from the screening criteria by working with unstructured text data rather than tabular clinical data. While demonstrating technical innovation in language model applications, the research lacks direct clinical outcomes, quantifiable clinical metrics, and synthetic tabular data generation. Consequently, the paper does not align with the specified screening requirements for clinical synthetic data research.	1.4	Exclude	Exclude	No tabular data
In silico assessment of nanoparticle toxicity powered by the Enalos Cloud Platform: Integrating automated machine learning and synthetic data for enhanced nanosafety evaluation	Varsou D.-D., Kolokathis P.D., Antoniou M., Sidiropoulos N.K., Tsoumanis A., Papadiamantis A.G., Melagraki G., Lynch I., Afantitis A.	2024	no	The study focuses on nanomaterial safety assessment, which is not a clinical application. While it has health implications, it's primarily focused on material science and toxicity assessment rather than clinical applications.	yes	The study appears to work with tabular data (dose-response toxicity data), though this isn't explicitly stated.	no	The study uses nanoparticle toxicity data, not human clinical or patient-related healthcare information. It specifically mentions avoiding animal testing.	yes	This appears to be an original research article presenting a novel workflow and methodology.	yes	The abstract mentions using "synthetic data generation techniques" to broaden the dataset, indicating that the study involves active creation of synthetic data using AI.	yes	The study uses synthetic data generation, though the specific AI methods (GANs, VAEs, etc.) aren't specified.	no	While the study has health implications, it doesn't demonstrate direct clinical applications like diagnosis, prognosis, or treatment planning.	yes	The study includes empirical evaluation with clear metrics for assessing the model's performance.	maybe	The study mentions prediction reliability and includes a three-step applicability domain method, but specific clinical metrics aren't mentioned.	Exclude	The study focuses on nanomaterial safety assessment using synthetic data generation techniques, but lacks direct clinical applications and does not involve human clinical data. While the research demonstrates empirical evaluation and original data creation, it fundamentally addresses material science and toxicity assessment rather than healthcare interventions. Consequently, the work does not meet the core screening criteria for clinical research.	1.4	Exclude	Exclude	No direct clinical application

Incorporating spatial information in deep learning parameter estimation with application to the intravoxel incoherent motion model in diffusion-weighted MRI.	Kaandorp, Misha P T, Zijlstra, Frank, Karimi, Davood, Gholipour, Ali, While, Peter T	2025	no	The paper focuses on technical aspects of medical image analysis and parameter estimation, rather than direct clinical applications. While it has medical relevance, it's primarily a methodological paper.	no	The study deals with imaging data (DWI) and synthetic data for training, not tabular data.	maybe	The study uses in vivo DWI data from a healthy volunteer, but this is not the primary focus - it's used for validation rather than being the basis for synthetic data generation.	yes	This appears to be an original research article with novel methodology and empirical evaluation.	maybe	The study does create synthetic data, but it's for training purposes in a technical context rather than for clinical data generation.	no	While synthetic data is used, it's fractal-noise-based synthetic data for training neural networks, not AI-generated synthetic clinical data.	no	The paper focuses on technical improvements in parameter estimation rather than direct clinical applications like diagnosis or treatment planning.	no	The study includes empirical evaluation with clear metrics, but these are focused on technical performance rather than clinical utility.	no	The study reports quantifiable outcomes, but these are technical performance metrics rather than clinical outcomes.	Exclude	The study primarily focuses on technical aspects of medical image analysis using deep learning, with a methodological approach that does not meet the screening criteria for clinical applications or synthetic data generation. While the research involves human data and includes empirical evaluation, it lacks direct clinical utility, quantifiable clinical outcomes, and does not generate synthetic clinical data for practical healthcare purposes. The paper's technical nature and imaging data focus disqualify it from meeting the systematic review's core requirements.	1.4	Exclude	Exclude	No tabular data
INSAFEDARE Project: Innovative Applications of Assessment and Assurance of Data and Synthetic Data for Regulatory Decision Support	Gallos P., Matragkas N., Islam S.U., Epiphaniou G., Hansen S., Harrison S., van Dijk B., Haas M., Pappous G., Brouwer S., Torlontano F., Abbasi S.F., Pournik O., Churm J., Mantas J., Parra-Calderon C.L., Petkousis D., Weber P., Dzingina B., Mraidha C., Maple C., Achterberg J., Spruit M., Saratsioti E., Moustaghfir Y., Arvanitis T.N.	2024	no	The paper appears to focus on regulatory aspects and quality assurance rather than direct clinical applications. While it mentions healthcare delivery, the primary focus seems to be on medical device regulation and training programs.	no	The abstract doesn't specify the type of data being used. While it mentions "datasets," there's no clear indication that it's specifically tabular data.	no	The abstract doesn't explicitly mention whether human clinical data is used. The focus seems to be on medical devices and regulatory frameworks.	no	This appears to be a project description paper rather than an original research article with empirical results.	no	While synthetic data generation is mentioned, it's discussed as a general capability rather than describing specific implementation. The paper appears to be more about a project framework than actual data creation.	no	The paper mentions synthetic data generation as a capability but doesn't specify the methods used (GANs, VAEs, etc.). It's discussed as part of a broader regulatory framework.	no	While the paper mentions healthcare delivery, it doesn't demonstrate direct clinical applications. The focus is on regulatory decision-making and quality assurance.	no	The paper appears to be describing a project framework rather than presenting empirical evaluation results.	no	The abstract doesn't mention any specific quantifiable outcomes or metrics.	Exclude	The abstract describes a project framework focused on medical device regulation and quality assurance, lacking direct clinical applications, empirical research, or quantifiable outcomes. While mentioning synthetic data generation, the paper does not demonstrate original research with specific AI methods or clear clinical utility metrics. The document appears more oriented towards regulatory processes and training programs rather than meeting the specified screening criteria for clinical synthetic data research.	1.4	Exclude	Exclude	Wrong publication type
I-SIRch: AI-powered concept annotation tool for equitable extraction and analysis of safety insights from maternity investigations.	Singh, Mohit Kumar, Cosma, Georgina, Waterson, Patrick, Back, Jonathan, Jun, Gyuchan Thomas	2024	maybe	The paper focuses on clinical applications in maternity care, specifically analyzing incident investigation reports to improve patient safety. However, it's primarily focused on the technical tool (I-SIRch) rather than direct clinical applications.	no	The study appears to work with text reports rather than tabular data. The abstract mentions "incident investigation reports" and "sentences," indicating non-tabular data format.	yes	The study uses real maternity incident investigation reports from human clinical settings.	yes	This is clearly an original research article with novel methodology and empirical evaluation.	no	While the study uses AI, it's not creating synthetic data but rather analyzing existing reports. The mention of "synthetic data" in testing refers to evaluation data, not creation.	no	The study does not generate synthetic tabular data. While it mentions synthetic data in testing, this is not the focus of the research.	maybe	While the study has implications for clinical practice (improving maternal safety), it's primarily focused on developing an analytical tool rather than direct clinical applications.	maybe	The study includes empirical evaluation with clear metrics, but these are for the tool's performance rather than clinical utility of synthetic data.	maybe	The study reports clear metrics (90% accuracy, 93% recall, 87% precision) but these are for the tool's performance rather than clinical outcomes.	Exclude	The study focuses on developing an AI tool for analyzing maternity incident reports, but does not meet key screening criteria related to synthetic data generation, tabular data format, and direct clinical applications. While the research involves human clinical data and demonstrates empirical evaluation, it primarily represents a methodological innovation in text analysis rather than a study of synthetic data creation or clinical utility. Consequently, the paper does not align sufficiently with the specified screening requirements for inclusion.	1.4	Exclude	Exclude	No tabular data
Kernel-based learning from both qualitative and quantitative labels: application to prostate cancer diagnosis based on multiparametric MR imaging	Niaf E., Flamary R., Rouviere O., Lartizien C., Canu S.	2014	maybe	The paper focuses on clinical applications (prostate cancer diagnosis) rather than purely technical aspects, but it is primarily about developing a new SVM method with clinical applications as a use case.	no	The study focuses on medical imaging data (MRI), not tabular data.	yes	The study uses human clinical data (prostate MRI images) for its evaluation.	yes	This appears to be an original research article presenting a new method (P-SVM) with empirical evaluation.	no	The paper does not involve creating synthetic data using AI. While it mentions "synthetic data sets" for testing, these are not the focus of the study.	no	The study does not involve generating synthetic tabular data using AI methods. It uses real MRI data and some synthetic datasets for testing the SVM method.	yes	The study demonstrates direct clinical application in prostate cancer diagnosis.	yes	The study includes original research with empirical evaluation and clear metrics for assessing performance.	yes	The study reports quantifiable outcomes in terms of classification performance and probability predictions.	Exclude	The study focuses on a probabilistic support vector machine method for prostate cancer diagnosis using MRI imaging, which fundamentally diverges from synthetic tabular data generation requirements. While demonstrating clinical application and empirical evaluation, the research fails key screening criteria by using imaging data and not creating synthetic tabular data through AI methods. Consequently, the paper does not align with the specified research focus on synthetic data generation.	1.4	Exclude	Exclude	No tabular data
KI-MAG: A knowledge-infused abstractive question answering system in medical domain	Zafar A., Sahoo S.K., Bhardawaj H., Das A., Ekbal A.	2024	maybe	The paper focuses on clinical applications through medical question-answering, but it appears to be more focused on the technical aspects of the QA system rather than direct clinical applications. The focus seems to be on improving the QA model itself rather than clinical decision-making.	no	The paper deals with text data (question-answer pairs) rather than tabular data. This clearly fails to meet the criterion for tabular data generation.	no	The abstract doesn't explicitly mention using human clinical data or patient information. It appears to be working with general medical knowledge rather than specific clinical data.	yes	This appears to be an original research article with novel contributions (KI-MAG model), meeting this criterion.	yes	The paper does involve active creation of synthetic data using AI, specifically generating synthetic question-answer pairs. This meets the criterion.	maybe	While the paper does generate synthetic data, it's not generating tabular data using methods like GANs or VAEs. Instead, it generates text-based question-answer pairs.	maybe	While the system is intended for medical applications, the abstract doesn't demonstrate direct clinical applications like diagnosis or treatment planning. It focuses on improving QA accuracy in general.	maybe	The study includes empirical evaluation with clear metrics, but these metrics are for QA performance rather than clinical utility.	maybe	The study reports quantifiable outcomes (Blue-1, Blue-2, Blue-3, and Blue-4 scores), but these are related to QA performance rather than clinical outcomes.	Exclude	The study focuses on a medical question-answering system using text-based synthetic data generation, which diverges from the required tabular data format and direct clinical application criteria. While demonstrating original research with empirical evaluation, the paper lacks clear connection to human clinical data and does not meet the specific screening requirements for synthetic tabular data generation in clinical settings.	1.4	Exclude	Exclude	No tabular data
Large scale simulation of labeled intraoperative scenes in unity.	Dowrick, Thomas, Davidson, Brian, Gurusamy, Kurinchi, Clarkson, Matthew J	2022	no	The study focuses on technical aspects of image segmentation and data generation for surgical training, rather than direct clinical applications.	no	The study deals with image data (laparoscopic video images) rather than tabular data.	yes	The study uses real patient data for validation (10 separate patients).	yes	This appears to be an original research article with clear methodology and results.	yes	The study actively creates synthetic data using Unity game engine for simulation.	maybe	While the study generates synthetic data, it uses Unity game engine rather than AI methods like GANs or VAEs.	no	The study focuses on improving training data for image-guided surgery rather than direct clinical applications like diagnosis or treatment planning.	yes	The study includes original research with clear empirical evaluation and metrics.	yes	The study reports clear quantifiable outcomes (DICE scores) for model performance.	Exclude	The study focuses on technical image segmentation using synthetic data generation through a game engine, which diverges from the required tabular data and direct clinical application criteria. While demonstrating empirical research with quantifiable outcomes, the methodology primarily addresses technical aspects of surgical training data rather than meeting the specified screening requirements for clinical AI applications.	1.4	Exclude	Exclude	No tabular data
Latent space autoencoder generative adversarial model for retinal image synthesis and vessel segmentation.	Radha, K, Karuna, Yepuganti	2025	no	The study focuses primarily on technical aspects of image generation and segmentation, rather than direct clinical applications. While it mentions disease diagnosis, this is not the primary focus.	no	The study deals with imaging data (fundus images) rather than tabular data. This is explicitly clear from the abstract's focus on "retinal fundus images" and "image-to-image translation."	yes	The study uses human clinical data (retinal fundus images from diabetic patients) as its basis.	yes	Based on the abstract, this appears to be an original research article with novel methodology and empirical evaluation.	yes	The study actively creates synthetic data using AI (specifically GANs and latent space auto-encoders) rather than using pre-existing synthetic datasets.	no	While the study does use AI methods (GANs) for synthetic data generation, it generates images rather than tabular data.	maybe	While the study has potential clinical applications in diabetic retinopathy diagnosis, it focuses more on the technical aspects of image generation and segmentation rather than direct clinical applications.	yes	The study includes original research with empirical evaluation, testing the synthetic data's effectiveness for segmentation tasks.	yes	The study reports quantifiable outcomes in terms of segmentation performance, though specific metrics aren't detailed in the abstract.	Exclude	The study focuses on generating synthetic retinal fundus images using AI techniques, which fundamentally deviates from the required tabular data format. Despite demonstrating original research with empirical evaluation and using human clinical data, the imaging-based approach disqualifies the paper from meeting critical screening criteria. The technical methodology, while innovative, does not align with the specified research parameters.	1.4	Exclude	Exclude	No tabular data
Learning 3D medical image keypoint descriptors with the triplet loss	Loiseau-Witon N., Kechichian R., Valette S., Bartoli A.	2022	no	The paper focuses on technical aspects (3D keypoint descriptor learning) rather than clinical applications. While it uses CT scans, the primary focus is on the technical methodology of keypoint matching.	no	The study deals with 3D CT scan data, which is imaging data, not tabular data.	yes	The study uses CT scans from human subjects, though the exact nature of the data (clinical vs. research) isn't explicitly stated.	yes	This appears to be an original research article with novel methodology and experimental results.	maybe	The study does create synthetic data, but it's semi-synthetic data through warping of existing CT scans rather than using AI to generate entirely new data.	no	While the study does generate synthetic data, it's not using AI methods like GANs or VAEs for generation. Instead, it uses geometric transformations to create semi-synthetic data.	no	The paper doesn't demonstrate direct clinical applications. It focuses on technical aspects of keypoint matching and registration.	yes	The study includes empirical evaluation with clear metrics and benchmarks.	yes	The study reports quantifiable outcomes in terms of descriptor performance and registration accuracy.	Exclude	The study focuses on technical methodology for 3D keypoint descriptor learning using CT scans, which primarily addresses computational challenges rather than direct clinical applications. While the research involves human clinical data and includes empirical evaluation, it fails to meet key screening criteria related to tabular data generation, AI-driven synthetic data creation, and clinical utility. The technical nature of the work and lack of clear clinical outcomes prevent it from satisfying the comprehensive screening requirements.	1.4	Exclude	Exclude	No tabular data
Leveraging deep learning algorithms for synthetic data generation to design and analyze biological networks.	Achuthan, Srisairam, Chatterjee, Rishov, Kotnala, Sourabh, Mohanty, Atish, Bhattacharya, Supriyo, Salgia, Ravi, Kulkarni, Prakash	2022	no	The paper appears to be primarily methodological, focusing on explaining and reviewing different deep learning methods for synthetic data generation, rather than focusing on clinical applications.	no	The abstract explicitly mentions both structured and unstructured data formats, including text and images, which violates the criterion requiring only tabular data generation.	maybe	While the paper mentions healthcare applications, it also discusses non-human data (DNA sequences, cancer cell cultures), so it's unclear if human clinical data is used.	no	The abstract suggests this is more of a review or overview paper rather than original research, given its broad scope and focus on explaining different methods.	maybe	It's unclear if the paper itself creates new synthetic data or just reviews these methods.	maybe	The paper clearly involves AI methods for synthetic data generation, including specific mention of GANs and VAEs, but it's not clear if this is original implementation or just review.	no	The paper mentions generating "realistic clinical notes" but doesn't demonstrate direct clinical applications like diagnosis or treatment planning. It appears more focused on the technical aspects.	no	The abstract doesn't indicate any original empirical evaluation or clear metrics for assessing clinical utility. It appears to be more descriptive and methodological.	no	The abstract doesn't mention any specific quantifiable outcomes or metrics for evaluating the synthetic data's utility.	Exclude	The abstract describes a methodological overview of synthetic data generation techniques in healthcare, focusing on deep learning methods across structured and unstructured data formats. While touching on healthcare applications, the paper appears to be a review that lacks direct clinical implementation, quantifiable outcomes, and empirical evaluation of synthetic data's clinical utility. The broad scope and technical orientation suggest limited alignment with specific clinical research screening criteria.	1.4	Exclude	Exclude	Wrong publication type
Leveraging Open-Source Large Language Models for Data Augmentation in Hospital Staff Surveys: Mixed Methods Study.	Ehrett, Carl, Hegde, Sudeep, Andre, Kwame, Liu, Dixizi, Wilson, Timothy	2024	no	The study focuses on educational applications (medical education) and text classification rather than direct clinical applications. While it mentions "patient care practices," this appears to be a secondary consideration.	no	The study deals with text data (survey responses) rather than tabular data. This is explicitly clear from the abstract's focus on text classification tasks.	maybe	While the data comes from hospital staff surveys, it's not directly patient-related healthcare information.	yes	This appears to be an original research article with clear methodology and results.	yes	The study actively creates synthetic data using LLMs (specifically LLaMA and Alpaca models) for data augmentation.	maybe	While the study does generate synthetic data using AI methods (LLMs), it's for text rather than tabular data.	no	The primary application is educational (medical education) rather than direct clinical applications like diagnosis or treatment planning.	yes	The study includes original research with clear empirical evaluation and metrics.	yes	The study reports quantifiable outcomes (AUC of 0.87 with SD 0.02) for the classification task.	Exclude	The study focuses on text-based data augmentation for medical education using large language models, which diverges from the required clinical and tabular data generation criteria. While demonstrating original research with quantifiable outcomes, the paper primarily addresses educational applications and text classification, failing to meet key screening requirements for clinical synthetic data generation. The research's methodological approach and outcomes do not align with the specified screening criteria for clinical synthetic data studies.	1.4	Exclude	Exclude	No tabular data

M.I.N.I.-KID interviews with adolescents: a corpus-based language analysis of adolescents with depressive disorders and the possibilities of continuation using Chat GPT	Jarvers I., Ecker A., Donabauer P., Kampa K., Weissenbacher M., Schleicher D., Kandsperger S., Brunner R., Ludwig B.	2024	yes	The study focuses on clinical applications, specifically early identification of depressive symptoms in adolescents through a chatbot system.	no	The study primarily deals with text data (interview transcripts and chatbot responses) rather than tabular data, which is a critical mismatch with the inclusion criteria.	yes	The study uses real clinical data from human adolescents with and without depressive disorders.	yes	This appears to be an original research article with clear methodology and results.	yes	The study actively creates synthetic data using Chat GPT to generate new interview responses.	maybe	While the study does use AI (Chat GPT) for synthetic data generation, it's generating text rather than tabular data, which doesn't align with the specific requirement for tabular data generation.	yes	The study has direct clinical applications in mental health diagnosis and early identification of depressive symptoms.	yes	The study includes original research with clear empirical evaluation and metrics.	yes	The study reports quantifiable outcomes, including classification accuracy of up to 97%.	Exclude	The study focuses on generating synthetic interview responses for adolescent depression detection using Chat GPT, but critically fails to meet the tabular data generation requirement. While demonstrating strong clinical application and empirical research methodology, the text-based data format disqualifies the paper from inclusion in the systematic review. The research provides valuable insights into AI-assisted mental health screening, but does not align with the specific data generation criteria.	1.4	Exclude	Exclude	No tabular data
Mapping and characterizing endometrial implants by registering 2D transvaginal ultrasound to 3D pelvic magnetic resonance images	Yavariabdi A., Bartoli A., Samir C., Artigues M., Canis M.	2015	no	The paper focuses on a technical method (slice-to-volume registration) rather than a clinical application. While it has medical imaging implications, the primary focus is on the technical methodology.	no	The study deals with imaging data (2D ultrasound and 3D MRI), not tabular data.	yes	The study uses clinical data (TVUS and MR images) from human patients.	yes	This appears to be an original research article with novel methodology and results.	no	The study creates "semi-synthetic" data through deformation of existing data, but this isn't AI-based synthetic data generation.	no	While the study does generate synthetic data, it's not using AI methods (GANs, VAEs, etc.) but rather geometric deformation techniques.	no	While the method could potentially be used for clinical applications (mapping endometrial implants), the abstract doesn't demonstrate direct clinical applications or outcomes.	no	The study includes empirical evaluation with clear metrics, but these are focused on technical performance rather than clinical utility.	no	The study reports quantifiable metrics (mean square error, Hausdorff distance, Dice similarity coefficient), but these are technical performance metrics rather than clinical outcomes.	Exclude	The study focuses on a technical imaging registration method using geometric deformation techniques, which fails to meet key screening criteria related to clinical applications, tabular data generation, and AI-based synthetic data creation. While the research involves human clinical data and demonstrates empirical evaluation, it primarily addresses technical methodology rather than direct clinical outcomes or AI-driven synthetic data generation.	1.4	Exclude	Exclude	No tabular data
Medigan: A Python library of pretrained generative models for medical image synthesis	Osuala R., Skorupko G., Lazrak N., Garrucho L., Garcia E., Joshi S., Jouide S., Rutherford M., Prior F., Kushibar K., Diaz O., Lekadir K.	2023	no	The paper focuses on a technical framework (medigan) for sharing generative models rather than direct clinical applications. While it mentions clinical applications, these are secondary to the technical implementation.	no	The paper explicitly focuses on imaging data (mammography, endoscopy, x-ray, MRI) rather than tabular data.	maybe	The paper uses medical imaging data, which implies human clinical data, though this isn't explicitly stated.	yes	This appears to be an original research article presenting a new framework, not a review or commentary.	maybe	The paper involves AI-based synthetic data generation, but primarily focuses on sharing existing pretrained models rather than creating new synthetic data. It's more about the framework than the generation process.	maybe	While the paper does involve AI methods (GANs) for synthetic data generation, it's focused on sharing existing models rather than the generation process itself.	no	While clinical applications are mentioned, they're not the primary focus. The paper is more about the technical framework for sharing models. The clinical applications are secondary.	maybe	The paper includes some empirical evaluation, but it's primarily focused on the technical aspects of the framework rather than clinical utility.	maybe	The paper reports some quantifiable outcomes (FID metrics) but these are focused on the technical aspects rather than clinical utility.	Exclude	The study primarily focuses on a technical framework for sharing generative models in medical imaging, which diverges from the required tabular data and direct clinical application criteria. While the research involves AI and medical data, its emphasis on model sharing and imaging modalities disqualifies it from meeting the systematic review's specific screening requirements. The paper's technical orientation and imaging data focus represent significant deviations from the desired research parameters.	1.4	Exclude	Exclude	No tabular data
Minimal data requirement for realistic endoscopic image generation with Stable Diffusion	Kaleta J., Dall'Alba D., Plotka S., Korzeniowski P.	2024	maybe	The paper focuses on computer-assisted surgical systems, which is a clinical application. However, it appears to be more focused on the technical aspects of image generation rather than direct clinical outcomes.	no	The study deals with image data (laparoscopic images), not tabular data.	yes	The study uses data from laparoscopic cholecystectomy, which involves human clinical data.	yes	This appears to be an original research article with novel methodology and results.	yes	The study does involve active creation of synthetic data using AI (Stable Diffusion model).	no	While the study does generate synthetic data using AI, it's focused on image-to-image translation rather than tabular data generation.	maybe	While the ultimate goal is to improve surgical systems, the paper focuses more on the technical aspects of image generation rather than demonstrating direct clinical applications.	maybe	The study includes empirical evaluation with clear metrics, but these are focused on technical performance rather than clinical utility.	maybe	The study reports quantifiable outcomes (Intersection over Union metrics), but these are technical performance metrics rather than clinical outcomes.	Exclude	The study focuses on image-to-image translation for surgical systems using AI, but fails key screening criteria by working with image data instead of tabular data. While demonstrating technical innovation in synthetic data generation, the research does not meet the required format and application specifications for the systematic review. Consequently, the paper does not align sufficiently with the predefined screening requirements.	1.4	Exclude	Exclude	No tabular data
Monotonic Gaussian Process for spatio-temporal disease progression modeling in brain imaging data	Abi Nader C., Ayache N., Robert P., Lorenzi M.	2020	maybe	The paper focuses on clinical applications, specifically analyzing disease trajectories in brain imaging for neurodegeneration. However, it appears to be primarily a technical/methodological paper introducing a new probabilistic generative model.	no	The study focuses on brain images, which are not tabular data. This clearly fails this criterion as imaging data is explicitly excluded.	yes	The study uses "large-scale imaging data from a clinical study" involving human patients.	yes	This appears to be an original research article introducing a new method.	no	While the paper uses a generative model, it's not clear if it's creating synthetic data for clinical use. The model appears to be used for analyzing real imaging data rather than generating synthetic clinical data.	no	The paper mentions testing on synthetic data, but this appears to be for validation purposes rather than generating synthetic clinical data for practical use. The focus is on analyzing real imaging data.	maybe	While the study has clinical relevance in analyzing neurodegeneration, it's not clear if it demonstrates direct clinical applications like diagnosis or treatment planning. It appears more focused on understanding disease progression patterns.	maybe	The study includes empirical evaluation, but the metrics appear to be more technical than clinically focused.	maybe	The abstract mentions "favourably comparing with standard blind source separation approaches" but doesn't clearly specify clinical outcome metrics.	Exclude	The study focuses on a probabilistic generative model for analyzing brain imaging data, which fundamentally fails the screening criteria due to its non-tabular data format and technical methodology. While the research involves human clinical data and demonstrates some clinical relevance, it does not meet the core requirements for synthetic data generation or direct clinical application. The paper's primary emphasis on technical modeling and imaging analysis disqualifies it from the specified screening criteria.	1.4	Exclude	Exclude	No tabular data
Motion Estimation by Deep Learning in 2D Echocardiography: Synthetic Dataset and Validation	Evain E., Sun Y., Faraz K., Garcia D., Saloux E., Gerber B.L., De Craene M., Bernard O.	2022	maybe	The paper focuses on clinical applications (echocardiography motion estimation) rather than purely technical aspects, but it has a strong technical component in developing the deep learning solution.	no	The study deals with imaging data (echocardiography sequences) rather than tabular data, which is a clear violation of the criterion.	yes	The study uses real patient data from two different clinical datasets (30 patients each).	yes	This appears to be an original research article with novel methodology and empirical evaluation.	yes	The study does involve active creation of synthetic data using AI through their "novel simulation pipeline."	maybe	While the study does generate synthetic data, it's for imaging sequences rather than tabular data. The AI methods used are for motion estimation, not data generation.	yes	The study demonstrates direct clinical applications in cardiac function characterization and myocardial deformation indices.	yes	The study includes thorough empirical evaluation with clear metrics and comparison to existing methods.	yes	The study reports clear quantifiable outcomes including endpoint error measurements and correlation metrics.	Exclude	The study focuses on echocardiography motion estimation using deep learning and synthetic data generation, demonstrating strong clinical applications and empirical evaluation. However, the research primarily involves imaging data sequences rather than tabular data, which critically disqualifies it from meeting the core screening requirements. Despite high-quality methodology and quantifiable outcomes, the fundamental data format deviation prevents the study from passing the screening criteria.	1.4	Exclude	Exclude	No tabular data
Moving Beyond CT Body Composition Analysis: Using Style Transfer for Bringing CT-Based Fully-Automated Body Composition Analysis to T2-Weighted MRI Sequences.	Haubold, Johannes, Pollok, Olivia Barbara, Holtkamp, Mathias, Salhofer, Luca, Schmidt, Cynthia Sabrina, Bojahr, Christian, Straus, Jannis, Schaarschmidt, Benedikt Michael, Borys, Katarzyna, Kohnke, Judith, Wen, Yutong, Opitz, Marcel, Umutlu, Lale, Forsting, Michael, Friedrich, Christoph M, Nensa, Felix, Hosch, Rene	2025	maybe	The paper focuses on clinical applications (body composition analysis) rather than purely technical aspects, but it is primarily a technical implementation paper with clinical applications as a secondary focus.	no	The study deals with imaging data (MRI and CT) rather than tabular data, which fails to meet the criterion requiring only tabular data generation.	yes	Yes, the study uses real MRI sequences from 120 patients.	yes	Yes, this is clearly an original research article with detailed methodology and results.	maybe	The study does use AI (CycleGAN) to create synthetic data, but this is used for image-to-image translation rather than tabular data generation.	no	While the study does generate synthetic data using AI (CycleGAN), it's for imaging data, not tabular data.	yes	The study demonstrates direct clinical applications in body composition analysis, which has clear clinical relevance.	yes	Yes, the study includes original research with clear empirical evaluation and metrics.	yes	Yes, the study reports quantifiable outcomes including Dice scores, Surface Dice, and Hausdorff Distance metrics.	Exclude	The study focuses on body composition analysis using MRI and CT imaging, which fundamentally conflicts with the requirement for tabular data generation. While the research demonstrates clinical relevance and includes empirical evaluation with quantifiable outcomes, the core data format and synthetic data generation approach do not align with the specified screening criteria. Consequently, the paper does not meet the essential requirements for inclusion.	1.4	Exclude	Exclude	No tabular data
Multi-modal brain tumor segmentation via conditional synthesis with Fourier domain adaptation	Khalil Y.A., Ayaz A., Lorenz C., Weese J., Pluim J., Breeuwer M.	2024	no	The study focuses on technical aspects of image synthesis and segmentation rather than direct clinical applications. While it has clinical relevance, the primary focus is on the technical methodology.	no	The study deals with medical imaging (MRI) data, not tabular data. This is explicitly clear from the abstract's focus on "multi-modal magnetic resonance imaging" and image synthesis.	yes	The study uses human clinical data (brain tumor MRI images) as its basis.	yes	This appears to be an original research article with novel methodology and empirical results.	yes	The study actively creates synthetic data using a conditional GAN approach, which is clearly described in the abstract.	no	While the study does use AI (GANs) for synthetic data generation, it generates images rather than tabular data.	maybe	While the study has clinical relevance for diagnosis and treatment planning, it focuses more on the technical aspects of image segmentation rather than direct clinical applications.	yes	The study includes clear empirical evaluation with specific metrics and comparisons.	yes	The study reports quantifiable outcomes in terms of Dice scores and performance improvements.	Exclude	The study focuses on technical image synthesis for brain tumor segmentation using GANs, which fundamentally diverges from the screening criteria's requirements for tabular data and direct clinical applications. While demonstrating sophisticated AI-driven medical imaging techniques, the research fails to meet key screening criteria related to data format, application focus, and synthetic data generation. The technical methodology, though innovative, does not align with the specified screening parameters.	1.4	Exclude	Exclude	No tabular data
NaroNet: Discovery of tumor microenvironment elements from highly multiplexed images	Jimenez-Sanchez D., Ariz M., Chang H., Matias-Guiu X., de Andrea C.E., Ortiz-de-Solorzano C.	2022	maybe	The paper focuses on clinical applications (cancer diagnosis and prognosis) rather than purely technical aspects, but it has a strong technical component in developing NaroNet.	no	The study primarily deals with imaging data (multiplex-stained tissue images and mass cytometry images), not tabular data.	yes	The study uses human clinical data from endometrial and breast cancer patients.	yes	This appears to be an original research article presenting a novel framework (NaroNet).	maybe	While the study mentions synthetic data, it's used for validation rather than being the primary focus. The synthetic data is used to simulate multiplex-immunostained images.	no	The study does generate synthetic data, but it's for image simulation rather than tabular data generation.	yes	The study demonstrates direct clinical applications in cancer diagnosis and prognosis.	yes	The study includes empirical evaluation on both synthetic and real datasets with clear clinical applications.	maybe	The abstract mentions "outstanding predictions" but doesn't explicitly detail the quantifiable metrics used.	Exclude	The study focuses on a deep learning framework for cancer diagnosis using imaging data, which fundamentally conflicts with the requirement for tabular data generation. While demonstrating strong clinical applications and empirical evaluation, the research fails to meet critical screening criteria related to data format and synthetic data generation. The imaging-centric approach disqualifies the paper from inclusion in a review targeting tabular synthetic data methodologies.	1.4	Exclude	Exclude	No tabular data
Nonrigid registration of dynamic medical imaging data using nD + t B-splines and a groupwise optimization approach.	Metz, C T, Klein, S, Schaap, M, van Walsum, T, Niessen, W J	2011	no	The paper focuses on technical aspects (image registration method) rather than clinical applications. While it has medical imaging applications, the primary focus is on the technical methodology.	no	The study deals with imaging data (CT, ultrasound, MRI) rather than tabular data. This is explicitly clear from the abstract's focus on "dynamic medical imaging data."	yes	The study uses human clinical data (CT scans of lungs and heart, ultrasound and MRI images).	yes	This appears to be an original research article presenting a new registration method with empirical validation.	no	The paper does not involve creating synthetic data using AI. While it mentions using synthetic data for validation, this is not the focus of the study.	no	The study does not generate synthetic tabular data using AI methods. It uses synthetic data for validation but doesn't create it.	maybe	While the method has potential clinical applications (e.g., left ventricular volume measurement), the paper focuses on the technical methodology rather than direct clinical applications.	yes	The study includes original research with clear empirical evaluation and metrics.	yes	The study reports quantifiable outcomes (subvoxel accuracy, 3% deviation from manual annotations).	Exclude	The study focuses on a technical image registration method using medical imaging data, which fundamentally diverges from the screening criteria's requirements for AI-generated synthetic tabular data. Despite demonstrating empirical validation and using human clinical data, the research fails key criteria by concentrating on imaging techniques rather than synthetic data generation or direct clinical applications. Consequently, the paper does not align with the specified screening requirements.	1.4	Exclude	Exclude	No tabular data

On the usability of synthetic data for improving the robustness of deep learning based segmentation of cardiac magnetic resonance images	Al Khalil Y., Amirrajab S., Lorenz C., Weese J., Pluim J., Breeuwer M.	2023	no	The study focuses on technical aspects of image segmentation and synthetic data generation for improving model robustness, rather than direct clinical applications.	no	The study deals with medical imaging (CMR images) rather than tabular data generation.	yes	The study uses cardiac magnetic resonance images from human patients.	yes	This appears to be an original research article with novel methodology and empirical evaluation.	yes	The study actively creates synthetic data using GANs, specifically a "segmentation-informed conditional GAN."	maybe	While the study does use AI methods (GANs) for synthetic data generation, it's for images rather than tabular data.	no	The study focuses on improving segmentation model performance rather than direct clinical applications like diagnosis or treatment planning.	yes	The study includes thorough empirical evaluation with clear metrics and comparison across multiple datasets.	yes	The study reports quantifiable metrics (Dice score and Hausdorff distance) for evaluating performance.	Exclude	The study focuses on technical image segmentation using GANs for cardiac magnetic resonance images, which fundamentally diverges from the screening criteria's requirements for clinical applications and tabular data generation. Despite demonstrating rigorous methodology and empirical evaluation, the research fails key screening criteria by concentrating on imaging data and model performance rather than direct clinical utility. Consequently, the paper does not align with the specified screening requirements for inclusion.	1.4	Exclude	Exclude	No tabular data
One for multiple: Physics-informed synthetic data boosts generalizable deep learning for fast MRI reconstruction	Wang Z., Yu X., Wang C., Chen W., Wang J., Chu Y.-H., Sun H., Li R., Li P., Yang F., Han H., Kang T., Lin J., Yang C., Chang S., Shi Z., Hua S., Li Y., Hu J., Zhu L., Zhou J., Lin M., Guo J., Cai C., Chen Z., Guo D., Yang G., Qu X.	2025	no	The study focuses primarily on technical aspects of MRI image reconstruction rather than clinical applications. While it has medical implications, the core focus is on the technical methodology.	no	The study deals with imaging data (MRI), not tabular data. This is explicitly clear from the abstract's focus on MRI reconstruction.	yes	The study uses human clinical data, specifically mentioning "in vivo MRI reconstructions" and validation with "2 neuro and 2 cardiovascular patient populations."	yes	This appears to be an original research article presenting a novel framework (PISF).	yes	The study involves active creation of synthetic data using deep learning methods, as evidenced by their "Physics-Informed Synthetic data learning Framework."	yes	The study uses AI methods (deep learning) to generate synthetic data, though for imaging rather than tabular data.	maybe	While the study has clinical implications for MRI reconstruction, it doesn't directly demonstrate clinical applications like diagnosis or treatment planning. It's more focused on the technical aspects of image reconstruction.	yes	The study includes empirical evaluation with clear metrics and validation by medical professionals across multiple scenarios.	yes	The study reports quantifiable outcomes, including reduction in reliance on real-world data "by up to 96%" and validation through medical professionals.	Exclude	The study focuses on a technical methodology for MRI image reconstruction using synthetic data generation, which fundamentally diverges from the screening criteria's requirements for tabular data and direct clinical applications. Despite demonstrating sophisticated AI-driven synthetic data techniques and involving human clinical data, the research fails to meet key screening criteria related to data format and clinical focus. Consequently, the paper does not align with the specified screening requirements.	1.4	Exclude	Exclude	No tabular data
Optimized automated cardiac MR scar quantification with GAN-based data augmentation	Lustermans D.R.P.R.M., Amirrajab S., Veta M., Breeuwer M., Scannell C.M.	2022	maybe	The paper focuses on clinical applications (cardiac MRI analysis and scar quantification) rather than purely technical aspects, but it has a strong technical component in the deep learning pipeline.	no	The study deals with imaging data (MRI), not tabular data, which is a clear violation of the criterion.	yes	The study uses human clinical data from the EMIDEC challenge, which involves cardiac MRI data from human patients.	yes	This appears to be an original research article with novel methodology and results.	yes	The study uses a conditional GAN to generate synthetic data, which meets this criterion.	maybe	The study uses AI (GAN) for synthetic data generation, but for imaging data rather than tabular data.	yes	The study demonstrates direct clinical application in cardiac scar quantification, which is relevant for diagnosis and treatment planning.	yes	The study includes original research with clear empirical evaluation and metrics for assessing the synthetic data's utility.	yes	The study reports clear quantifiable outcomes using Dice similarity coefficient (DSC) metrics.	Exclude	The study involves cardiac MRI analysis using synthetic data generation through a conditional GAN, demonstrating clinical applications in scar quantification with quantifiable outcomes. However, the research focuses on imaging data rather than tabular data, which fundamentally disqualifies it from meeting the primary screening criteria. Despite strong methodological rigor and clinical relevance, the data format represents a critical deviation from the specified requirements.	1.4	Exclude	Exclude	No tabular data
Optimized Combination of Multiple Graphs with Application to the Integration of Brain Imaging and (epi)Genomics Data	Bai Y., Pascal Z., Calhoun V., Wang Y.-P.	2020	maybe	The paper focuses on clinical applications (schizophrenia diagnosis) rather than purely technical aspects, but it has a strong methodological component.	no	The study uses multiple data types including SNP, fMRI, and DNA methylation data. While some of these are tabular, fMRI data is imaging data, which violates this criterion.	yes	The study uses human clinical data from 184 subjects with schizophrenia.	yes	This appears to be an original research article with novel methodology and empirical evaluation.	no	The abstract mentions using synthetic data for validation but doesn't indicate active creation of synthetic data as a primary focus. The synthetic data appears to be used as a validation tool rather than the main research focus.	no	While synthetic data is mentioned, it's not clear if AI methods were used for generation. The focus is on semi-supervised learning rather than synthetic data generation.	yes	The study demonstrates clinical application in diagnosis of schizophrenia.	yes	The study includes empirical evaluation with clear metrics and comparison to existing approaches.	yes	The study reports classification accuracy as a quantifiable outcome.	Exclude	The study involves multi-omics data analysis for schizophrenia diagnosis using semi-supervised learning, but fails to meet key screening criteria due to the inclusion of imaging data and lack of focus on synthetic data generation. While demonstrating clinical application and empirical evaluation, the research does not align with the specific requirements for synthetic data research, particularly in terms of data format and generation methodology.	1.4	Exclude	Exclude	No tabular data
Optimizing Pix2Pix GAN With Attention Mechanisms for AI-Driven Polyp Segmentation in IoMT-Enabled Smart Healthcare	Mazumdar H., Chakraborty C., Sathvik M., Jayakumar P., Kaushik A.	2025	maybe	The paper focuses on clinical applications (polyp segmentation in colonoscopy), but it's primarily a technical implementation of a GAN for image processing. While it has clinical implications, the focus appears to be on the technical methodology rather than clinical applications.	no	The study deals with imaging data (colonoscopy images), not tabular data. This is a clear violation of the criterion for tabular data generation.	yes	Yes, the study uses human clinical data (colonoscopy images from patients).	yes	Yes, this appears to be an original research article with novel methodology and empirical evaluation.	maybe	While the paper mentions using synthetic data, it's unclear if they actively created it or used existing synthetic datasets. The abstract doesn't explicitly state their role in creating the synthetic data.	no	The paper does use GANs, but for image segmentation rather than tabular data generation. This doesn't meet the specific requirement for tabular data generation.	yes	Yes, the study has direct clinical applications in polyp detection and colorectal cancer screening.	yes	Yes, the study includes empirical evaluation with "rigorous tests on multiple public colonoscopy datasets."	yes	Yes, the study reports quantifiable outcomes in terms of segmentation performance and accuracy.	Exclude	The study focuses on image-based polyp segmentation using GANs, which fundamentally conflicts with the requirement for tabular data generation. While demonstrating clinical relevance and empirical evaluation, the research primarily addresses technical image processing methodology rather than synthetic tabular data creation. Consequently, the paper does not meet critical screening criteria for inclusion in the systematic review.	1.4	Exclude	Exclude	No tabular data
Predicting classifier performance with limited training data: Applications to computer-aided diagnosis in breast and prostate cancer	Basavanhally A., Viswanath S., Madabhushi A.	2015	maybe	The paper focuses on clinical applications (cancer detection and classification) but primarily from a methodological perspective of classifier selection and performance prediction. While it has clinical relevance, it's more focused on the technical aspects of classifier evaluation.	no	The paper deals with imaging data (histopathology and magnetic resonance spectroscopy), not tabular data.	yes	Yes, the study uses human clinical data (prostate and breast cancer imaging data).	yes	Yes, this appears to be an original research article with novel methodology and empirical evaluation.	no	The paper does not involve active creation of synthetic data using AI. While it mentions "synthetic data" briefly, this appears to be for validation purposes rather than the main focus of the study.	no	The paper does not involve generating synthetic tabular data using AI methods. The synthetic data mentioned is likely for validation of their classifier selection method.	maybe	While the study has clinical relevance (cancer detection), it focuses more on the methodology of classifier selection rather than direct clinical applications.	yes	Yes, the study includes original research with clear empirical evaluation and metrics.	yes	Yes, the study reports quantifiable outcomes in terms of error rates and interquartile ranges.	Exclude	The study primarily focuses on imaging data and classifier selection methodology, which fundamentally conflicts with the screening criteria requiring tabular data and AI-driven synthetic data generation. While the research involves human clinical data and demonstrates quantifiable outcomes, its technical approach and data format disqualify it from meeting the core screening requirements. The methodological emphasis on classifier performance prediction rather than direct clinical applications further reduces its alignment with the specified criteria.	1.4	Exclude	Exclude	No tabular data
Predictive modeling for occupational safety outcomes and days away from work analysis in mining operations	Yedla A., Kakhki F.D., Jannesari A.	2020	no	The paper focuses on occupational safety in mining, which is not a clinical application. While it deals with health and safety, it's in an industrial context rather than a clinical one.	maybe	The study does work with tabular data, but also includes narrative/text data. The synthetic data augmentation mentioned appears to be for text data using word embeddings.	no	The study uses mining accident data, which is human-related but not clinical healthcare data.	yes	This appears to be an original research article with empirical evaluation and results.	no	The study uses synthetic data augmentation, but it's specifically for text data using word embeddings, not for generating new tabular data.	no	While the study does use synthetic data augmentation, it's for text data using word embeddings, not for generating synthetic tabular data using AI methods like GANs or VAEs.	no	The application is occupational safety prediction, not clinical diagnosis, prognosis, or treatment planning.	yes	The study includes clear empirical evaluation with specific metrics and comparisons between different models.	yes	The study reports quantifiable outcomes including F1 scores, mean squared error, and predictive performance metrics.	Exclude	The study focuses on mining safety using machine learning techniques, which fundamentally diverges from clinical healthcare applications. Despite employing sophisticated data analysis methods and generating quantifiable outcomes, the research fails to meet critical screening criteria related to clinical data, synthetic data generation, and healthcare-specific applications. Consequently, the paper does not align with the specified review requirements for clinical AI research.	1.4	Exclude	Exclude	No synthetic data generation
Quantitative analysis in clinical applications of brain MRI using independent component analysis coupled with support vector machine	Chai J.-W., Chen C.C. C., Chiang C.-M., Ho Y.-J., Chen H.-M., Ouyang Y.-C., Yang C.-W., Lee S.-K., Chang C.-I.	2010	maybe	The paper focuses on clinical applications (brain tissue quantification and classification), but it's primarily a technical methodology paper. While it has clinical relevance, the focus appears to be on the technical aspects of the ICA+SVM method.	no	The study uses imaging data (MRI), not tabular data.	yes	Yes, the study uses both synthetic and real clinical MR data from human brains.	yes	Yes, this appears to be an original research article with clear methodology and results.	maybe	The study uses synthetic data, but it's not clear if this is AI-generated synthetic data. The abstract mentions "synthetic MR data" but doesn't specify the generation method.	no	While synthetic data is used, there's no indication that AI methods (GANs, VAEs, etc.) were used for generation. The focus is on ICA and SVM for analysis.	yes	The study demonstrates clinical applications in brain tissue quantification and classification, which could be used for diagnosis.	yes	Yes, the study includes empirical evaluation with clear metrics.	yes	Yes, the study reports quantifiable outcomes including Tanimoto indexes and coefficient of variations.	Exclude	The study focuses on brain tissue classification using MRI data, which fundamentally fails the screening criteria due to its use of imaging data instead of tabular data. While demonstrating clinical relevance and empirical evaluation, the research does not meet key requirements for synthetic data generation using AI methods. The technical methodology and imaging-based approach disqualify it from the systematic review.	1.4	Exclude	Exclude	No tabular data
Rapid whole-heart CMR with single volume super-resolution	Steeden J.A., Quail M., Gotschy A., Mortensen K.H., Hauptmann A., Arridge S., Jones R., Muthurangu V.	2020	yes	The study focuses on clinical applications of MRI imaging in cardiology, specifically improving whole heart imaging acquisition times while maintaining diagnostic quality.	no	The study deals with medical imaging data (MRI), not tabular data. This is a critical failure of a core criterion.	yes	The study uses real patient data from 40 patients and includes clinical validation.	yes	This appears to be an original research article with clear methodology and results.	yes	The study creates synthetic data using AI methods, specifically creating low-resolution versions of high-resolution MRI images.	maybe	While the study does use AI (U-Net) and synthetic data, it's for image super-resolution rather than tabular data generation.	yes	The study demonstrates direct clinical applications in cardiac MRI imaging and diagnosis.	yes	The study includes thorough empirical evaluation with clear metrics and comparison against reference data.	yes	The study reports multiple quantifiable outcomes including vessel diameters, image quality metrics, and diagnostic scoring.	Exclude	The study focuses on clinical cardiac MRI imaging using AI techniques for super-resolution, demonstrating strong clinical applications and empirical validation. However, the research fundamentally fails to meet the screening criteria due to its use of imaging data instead of tabular data, which represents a critical disqualifying factor for inclusion. Despite meeting most other criteria with high-quality methodology and quantifiable outcomes, the data format mismatch prevents systematic review consideration.	1.4	Exclude	Exclude	No tabular data
Recognition of anxiety and depression using gait data recorded by the kinect sensor: a machine learning approach with data augmentation	Shoryabi M., Hajipour A., Shoeibi A., Foroutannia A.	2025	maybe	The study focuses on clinical applications (mental health assessment) rather than purely technical aspects, but it is primarily a diagnostic/assessment tool rather than a direct clinical intervention.	no	The study uses gait data, which is time-series data, not tabular data, violating the requirement for only tabular data generation.	yes	The study uses human participants (50 participants) and clinical data (GAD-7 and BDI-II scales).	yes	This appears to be an original research article with empirical results, not a review or commentary.	no	The study uses "additive Gaussian noise method" for data augmentation, which is not an AI-based synthetic data generation method.	no	The synthetic data generation method used (Gaussian noise) is not an AI method like GANs or VAEs.	yes	The study demonstrates direct clinical application in mental health assessment and diagnosis.	yes	The study includes original research with clear empirical evaluation and metrics.	yes	The study reports clear quantifiable outcomes (classification accuracy of 61.67% for anxiety and 86.53% for depression).	Exclude	The study involves gait analysis for mental health assessment using human participants and clinical data, demonstrating quantifiable outcomes and empirical evaluation. However, critical methodological limitations include the use of time-series data instead of tabular data and non-AI synthetic data generation techniques, which disqualify the paper from meeting key screening criteria. These fundamental deviations from the required methodological approach render the study unsuitable for inclusion.	1.4	Exclude	Exclude	No tabular data
Reducing windmill artifacts in clinical spiral CT using a deep learning-based projection raw data upsampling: Method and robustness evaluation.	Magonov, Jan, Maier, Joscha, Erath, Julien, Sunnegardh, Johan, Fournie, Eric, Stierstorfer, Karl, Kachelries, Marc	2024	no	The paper focuses on technical aspects of CT imaging (reducing windmill artifacts) rather than clinical applications. While it's in a medical context, it's primarily about image processing technology.	no	The study deals with CT imaging data, which is not tabular data. It focuses on image reconstruction and raw data interpolation.	maybe	The study uses both real patient scans and synthetic phantom data, but the focus is on technical imaging aspects rather than clinical healthcare information.	yes	This appears to be an original research article with clear methodology and results.	no	While the study uses synthetic data, it's not creating synthetic clinical data for clinical purposes. Instead, it's generating synthetic CT scans for technical validation.	no	The study does use AI methods (deep learning) for data processing, but not for generating synthetic tabular clinical data. The synthetic data is for technical validation of CT imaging.	no	The study focuses on technical image quality improvement rather than direct clinical applications like diagnosis or treatment planning.	no	The study includes thorough empirical evaluation with clear metrics, but these are technical rather than clinical metrics.	no	The study reports quantifiable outcomes (RMSE) but these are technical metrics rather than clinical outcomes.	Exclude	The study focuses on technical CT imaging improvements using deep learning, primarily addressing image processing challenges rather than clinical applications. While the research involves synthetic data and patient scans, it does not meet the screening criteria for synthetic tabular clinical data generation or direct clinical utility. The paper's technical orientation and imaging-specific methodology diverge from the targeted research scope.	1.4	Exclude	Exclude	No tabular data

Rethinking Copy-Paste for Consistency Learning in Medical Image Segmentation.	Huang, Senlong, Ge, Yongxin, Liu, Dongfang, Hong, Mingjian, Zhao, Junhan, Loui, Alexander C	2025	maybe	The paper focuses on medical image segmentation, which is a technical aspect rather than a direct clinical application. While it has clinical relevance, the primary focus is on the technical methodology of semi-supervised learning and copy-paste techniques.	no	The paper deals with medical images, not tabular data. This is explicitly clear from the focus on image segmentation and the datasets mentioned (cardiac structures, prostate structures, etc.).	yes	Yes, the study uses human clinical data, as evidenced by the use of medical imaging datasets from various clinical domains.	yes	Yes, this appears to be an original research article with novel methodology and experimental results.	no	While the paper involves creating synthetic data through copy-paste techniques, this is not using AI methods like GANs or VAEs for generation. Instead, it uses a manual copy-paste approach with consistency learning.	no	The paper does involve synthetic data generation, but not through the specified AI methods (GANs, VAEs, etc.). The synthetic data is created through copy-paste techniques.	maybe	While the paper has clinical relevance through medical image segmentation, it doesn't directly demonstrate clinical applications like diagnosis or treatment planning. It focuses on the technical aspects of improving segmentation accuracy.	yes	Yes, the study includes extensive empirical evaluation across six medical image datasets with clear metrics.	yes	Yes, the study reports quantifiable outcomes, specifically Dice scores for segmentation performance.	Exclude	The study focuses on medical image segmentation using semi-supervised learning techniques, which diverges from the specified screening criteria by involving image data rather than tabular data and using copy-paste methods instead of AI-driven synthetic data generation. While the research demonstrates clinical relevance and includes empirical evaluation with quantifiable outcomes, it fundamentally does not align with the core requirements of synthetic data generation using AI methods. Consequently, the paper fails to meet several key screening criteria.	1.4	Exclude	Exclude	No tabular data
Revolutionizing prostate cancer screening: Machine learning-driven FTIR analysis of biofluidic EVs integrated with clinical profiles	Wong L.-W., Bu Z.-H., Supramaniam J., Goh B.-H., Lee L.-H., Lee W.-L.	2025	yes	The study focuses on clinical applications, specifically prostate cancer detection and diagnosis, rather than purely technical, privacy, methodological or educational aspects.	maybe	The study primarily uses FTIR spectral data and clinical data, but the spectral data is not tabular in nature. FTIR data is typically time-series or spectral data, which falls outside the tabular data requirement.	yes	The study uses data from 22 prostate cancer patients and 31 healthy controls, clearly using human clinical data.	yes	The study appears to be an original research article with clear methodology and results.	maybe	The study uses SMOTE (Synthetic Minority Oversampling Technique) which is a statistical oversampling method, not an AI-based synthetic data generation method. This doesn't meet the criterion for AI-based synthetic data creation.	maybe	While the study does generate synthetic data, it uses SMOTE rather than AI methods like GANs or VAEs. This doesn't meet the specific requirement for AI-based synthetic data generation.	yes	The study demonstrates direct clinical application in prostate cancer detection and diagnosis.	yes	The study includes clear empirical evaluation with defined metrics and methodology.	yes	The study reports quantifiable outcomes, specifically an AUC of 0.9167 for prostate cancer prediction.	Exclude	The study focuses on prostate cancer detection using FTIR spectral and clinical data, but fails to meet key screening criteria related to tabular data format and AI-based synthetic data generation. While demonstrating strong clinical application and empirical evaluation, the methodology relies on statistical oversampling techniques rather than AI-driven synthetic data creation, which disqualifies it from meeting the systematic review's specific requirements.	1.4	Exclude	Exclude	No tabular data
Rhi3DGen: Analyzing Rhinophyma using 3D face models and synthetic data	Mohanty A., Sutherland A., Bezbradica M., Javidnia H.	2023	no	The study focuses on technical aspects of image generation and classification rather than direct clinical applications. While it's in a medical context, it's primarily about the technical methodology.	no	The study deals with 3D image data and synthetic images, not tabular data. This is explicitly clear from the abstract mentioning "3D parametric modeling" and "images."	yes	The study uses real-world Rhinophyma images and generates synthetic data based on human clinical conditions.	yes	This appears to be an original research article with novel methodology and results.	yes	The study actively creates synthetic data using 3D parametric modeling, though not specifically using AI methods like GANs or VAEs.	no	While synthetic data is generated, it's not using AI methods for tabular data generation as specified in the criteria. Instead, it uses 3D parametric modeling for image generation.	maybe	While the study has potential clinical applications in diagnosis, it focuses more on the technical aspects of data generation and classification rather than direct clinical utility.	yes	The study includes empirical evaluation with clear metrics and validation using GradCAM visualization.	yes	The study reports quantifiable outcomes (95% recall in classification).	Exclude	The study focuses on 3D image generation for Rhinophyma using parametric modeling, which fundamentally differs from the required tabular data generation using AI methods. Despite demonstrating technical innovation and quantifiable outcomes, the research fails to meet critical screening criteria related to data format and synthetic data generation methodology. Consequently, the paper does not align with the systematic review's specific requirements.	1.4	Exclude	Exclude	No tabular data
Riemannian frameworks for the harmonization of resting-state functional MRI scans	Honnorat N., Seshadri S., Killiany R., Blangero J., Glahn D.C., Fox P., Habes M.	2024	no	The paper focuses on technical aspects of MRI harmonization rather than direct clinical applications. While it mentions "relevant clinical information," the primary focus is on the technical methodology of harmonizing MRI scans.	no	The study deals with imaging data (fMRI) and time series data, not tabular data. This is explicitly clear from the abstract's focus on "resting-state functional MRI scans" and "time series."	yes	The study uses human clinical data from multiple sources including the ABIDE consortium, Human Connectome Project, and Framingham Heart Study.	yes	This appears to be an original research article with novel methodology and empirical evaluation.	maybe	While the paper mentions generating synthetic data, it's not clear if this is done using AI methods. The synthetic data appears to be used for testing the harmonization method rather than being the primary focus.	maybe	The abstract doesn't specify the method used for synthetic data generation. While synthetic data is mentioned, there's no indication of AI methods being used.	no	While the paper mentions "relevant clinical information associated with aging or brain disorders," it doesn't demonstrate direct clinical applications. The focus is on the technical harmonization process.	maybe	The study includes empirical evaluation with clear metrics for the harmonization process, but not for clinical utility.	no	The study reports on the success of harmonization but doesn't clearly specify clinical outcome metrics.	Exclude	The study focuses on technical MRI harmonization using imaging and time series data, which deviates from the required tabular data format and clinical application criteria. While the research involves human clinical data and demonstrates empirical evaluation, it lacks direct clinical applications and clear AI-driven synthetic data generation methods. The technical methodology and imaging data approach disqualify the paper from meeting the specified screening requirements.	1.4	Exclude	Exclude	No tabular data
Robust Collaborative Clustering of Subjects and Radiomic Features for Cancer Prognosis	Liu H., Li H., Habes M., Li Y., Boimel P., Janopaul-Naylor J., Xiao Y., Ben-Josef E., Fan Y.	2020	maybe	The paper focuses on radiomic analysis and clinical outcomes prediction, which is a clinical application. However, it appears to be primarily a methodological paper about feature dimensionality reduction.	no	The study deals with radiomic features from FDG-PET/CT imaging data, which is not tabular data in the traditional sense. It involves imaging data analysis.	yes	The study uses real clinical data from rectal cancer patients.	yes	This appears to be an original research article presenting a new method for feature dimensionality reduction.	no	The paper does not involve creating synthetic data using AI. While it mentions experiments on synthetic data, this appears to be for validation rather than the main focus.	no	The paper does not generate synthetic tabular data using AI methods. The synthetic data mentioned is likely for testing the method rather than being the focus of the study.	yes	The study demonstrates clinical applications in patient stratification and prediction of clinical outcomes.	yes	The study includes empirical evaluation on both synthetic and real clinical data with clear comparisons to alternative methods.	yes	The abstract mentions evaluation results comparing performance with alternative methods, suggesting quantifiable outcomes.	Exclude	The study focuses on radiomic feature dimensionality reduction for rectal cancer patients, utilizing imaging data and demonstrating clinical applications in patient stratification and outcome prediction. However, the research does not meet key screening criteria related to synthetic tabular data generation, as it primarily involves methodological analysis of imaging features rather than AI-driven synthetic data creation. Consequently, the paper falls short of the specified screening requirements despite its clinical relevance.	1.4	Exclude	Exclude	No tabular data
Roles of AI-Based Synthetic Data in Health Economics and Outcomes Research	Lai T.C., Ngorsuraches S.	2025	no	The paper appears to be primarily focused on methodological aspects and potential applications rather than specific clinical applications. It's an overview paper discussing synthetic data in health economics and outcomes research (HEOR).	maybe	The abstract doesn't specify the type of data being discussed. While it mentions "data" generally, there's no explicit mention of tabular data specifically.	maybe	The paper mentions health economics and outcomes research, suggesting human clinical data context, but doesn't explicitly discuss using human clinical data.	no	This appears to be a review or perspective paper rather than original research, as indicated by phrases like "provide a concise overview" and the general discussion nature.	no	The paper appears to be discussing synthetic data generation in general terms rather than presenting original data creation. It's more of an overview paper.	no	While synthetic data is the main topic, the paper doesn't appear to present specific AI methods for generating synthetic data. It's discussing the concept broadly.	no	While the paper discusses potential applications in HEOR, it doesn't demonstrate direct clinical applications. It's more theoretical.	no	The paper doesn't appear to include original empirical evaluation. It's discussing potential applications and challenges rather than presenting empirical results.	no	The abstract doesn't mention any specific quantifiable outcomes or metrics.	Exclude	The abstract presents a methodological overview of synthetic data in health economics and outcomes research, lacking specific empirical evidence, original data creation, or direct clinical applications. While discussing potential uses of synthetic data, the paper fails to meet key screening criteria related to original research, quantifiable outcomes, and concrete clinical utility. The work appears more conceptual than research-driven, focusing on theoretical potential rather than demonstrating practical implementation.	1.4	Exclude	Exclude	Wrong publication type
Segmentation of brain magnetic resonance images using deep learning classification and multi-modal composition	Kala R., Deepa P.	2020	maybe	The paper focuses on clinical applications (brain tumor detection and segmentation) rather than purely technical aspects, but it is primarily a technical methodology paper.	no	The study uses MRI imaging data, not tabular data, which is explicitly excluded by this criterion.	yes	The study uses human clinical data (brain tumor MRI scans from BRATS 2013 database).	yes	This appears to be an original research article with clear methodology and results.	no	The paper does not involve creating synthetic data. It uses real MRI data from the BRATS 2013 database and mentions "Synthetic data set" only as one of the validation sets.	no	The paper does not generate synthetic data using AI methods. While it uses deep learning, it's for segmentation, not data generation.	yes	The study demonstrates direct clinical applications in diagnosis and treatment planning for brain tumors.	yes	The study includes original research with clear empirical evaluation and metrics.	yes	The study reports quantifiable outcomes including Dice Similarity Coefficient, Jaccard similarity index, accuracy, precision, sensitivity, and specificity.	Exclude	The study focuses on brain tumor segmentation using deep learning techniques, but critically fails key screening criteria by using imaging data instead of tabular data and not generating synthetic data. Despite demonstrating strong clinical applications and empirical evaluation, the fundamental mismatch with synthetic data generation and data format requirements disqualifies the paper from meeting the core screening criteria.	1.4	Exclude	Exclude	No tabular data
Seizure onset zone classification based on imbalanced iEEG with data augmentation	Zhao X., Sole-Casals J., Sugano H., Tanaka T.	2022	maybe	The paper focuses on clinical applications (seizure onset zone identification for epilepsy surgery), but also has significant technical components. However, the clinical application is primary.	no	The study deals with iEEG (intracranial electroencephalogram) data, which is time-series data. This explicitly fails the criterion requiring only tabular data.	yes	The study uses human clinical iEEG data from epilepsy patients.	yes	This appears to be an original research article with novel methodology and results.	no	The study creates synthetic data using a novel EEG augmentation method (EEGAug), but this appears to be a statistical/technical method rather than AI-based generation.	no	While the study generates synthetic data, it uses a frequency domain transformation method rather than AI methods like GANs or VAEs.	yes	The study has direct clinical applications in epilepsy surgery planning and diagnosis.	yes	The study includes empirical evaluation comparing their method against other approaches.	yes	The study reports comparative performance metrics against other methods, though specific numbers aren't provided in the abstract.	Exclude	The study focuses on epilepsy seizure onset zone identification using iEEG data, but fails key screening criteria due to its use of time-series data and non-AI synthetic data generation methods. While demonstrating clinical relevance and empirical evaluation, the research does not meet the specific requirements for tabular data and AI-driven synthetic data creation. Consequently, the paper would be excluded from the systematic review based on these technical limitations.	1.4	Exclude	Exclude	No tabular data
Semantic segmentation dataset authoring with simplified labels	Uramoto L., Hayashi Y., Oda M., Kitasaka T., Mori K.	2025	no	The paper focuses on semantic segmentation of laparoscopic images, which is a technical/methodological aspect rather than a direct clinical application. While it has medical relevance, it's primarily about improving dataset creation and annotation processes.	no	The study deals with laparoscopic images, not tabular data. This is explicitly clear from the abstract's focus on semantic segmentation of images.	yes	The study uses laparoscopic images from surgical procedures, which involves human clinical data.	yes	This appears to be an original research article with novel methodology and empirical evaluation.	no	While the paper discusses synthetic data, it's not focused on creating synthetic data using AI. Instead, it focuses on creating simplified labels for real images.	no	The paper mentions synthetic data only briefly and doesn't focus on generating synthetic tabular data using AI methods.	no	While the study has medical relevance, it doesn't demonstrate direct clinical applications like diagnosis or treatment planning. It's focused on improving dataset creation processes.	maybe	The study includes original research with empirical evaluation and clear metrics, but these metrics are for annotation quality rather than clinical utility.	maybe	The study reports quantifiable outcomes (Dice scores) but these are related to annotation accuracy rather than clinical outcomes.	Exclude	The study focuses on semantic segmentation of laparoscopic images, primarily addressing methodological challenges in dataset creation rather than direct clinical applications. While using human clinical data and presenting original research, the work fails key screening criteria by dealing with image data instead of tabular data and not generating synthetic data using AI methods. The research is more technically oriented toward improving annotation processes than demonstrating clinical utility.	1.4	Exclude	Exclude	No tabular data
Semi-supervised GAN-based Radiomics Model for Data Augmentation in Breast Ultrasound Mass Classification.	Pang, Ting, Wong, Jeannie Hsiu Ding, Ng, Wei Lin, Chan, Chee Seng	2021	yes	The study focuses on clinical applications (breast mass classification) rather than purely technical aspects. While it involves technical elements (GANs), these are in service of a clinical goal.	maybe	The study focuses on medical imaging (ultrasound images) rather than tabular data, which is a clear violation of this criterion.	yes	The study uses real human clinical data (1447 ultrasound images from a tertiary hospital).	yes	This appears to be an original research article with clear methodology and results.	yes	The study actively creates synthetic data using a semi-supervised GAN method, not using pre-existing synthetic datasets.	yes	The study uses AI methods (specifically GANs) for synthetic data generation, though for images rather than tabular data.	yes	The study demonstrates direct clinical application in breast mass classification for diagnosis.	yes	The study includes original research with empirical evaluation and clear metrics.	yes	The study reports clear quantifiable outcomes (accuracy 90.41%, sensitivity 87.94%, specificity 85.86%).	Exclude	The study demonstrates strong clinical application in breast mass classification using AI-generated synthetic ultrasound images, with robust empirical evaluation and quantifiable outcomes. However, the research fundamentally fails the screening criteria by focusing on medical imaging data rather than tabular data, which is a critical exclusion factor. Despite meeting most other criteria with high quality, the data format disqualifies the study from further consideration.	1.4	Exclude	Exclude	No tabular data

Semi-supervised semantic segmentation of prostate and organs-at-risk on 3D pelvic CT images	Zhang Z., Zhao T., Gay H., Zhang W., Sun B.	2021	no	The study focuses on technical aspects of image segmentation and data augmentation rather than clinical applications. While it mentions radiotherapy treatment planning, this is more of a potential application rather than the focus of the study.	no	The study deals with 3D CT images, which are not tabular data. This is explicitly stated in the abstract ("3D pelvic CT image semantic segmentation").	yes	The study uses human clinical data (male pelvic CT images).	yes	This appears to be an original research article with novel methodology and experimental results.	yes	The study actively creates synthetic data using GANs for data augmentation.	yes	The study uses GANs to generate synthetic data, though for images rather than tabular data.	maybe	While the study mentions radiotherapy treatment planning as an application, it doesn't directly demonstrate clinical applications. The focus is on the technical performance of the segmentation method.	yes	The study includes original research with clear empirical evaluation and metrics.	yes	The study reports clear quantifiable metrics (Dice similarity coefficient, average Hausdorff distance, and average surface Hausdorff distance).	Exclude	The study focuses on technical image segmentation using GANs for 3D CT images, which fundamentally conflicts with the requirement for tabular data generation. While demonstrating original research with quantifiable outcomes, the paper fails key screening criteria by emphasizing methodological innovation over direct clinical applications and using imaging rather than tabular data formats. Consequently, the research does not align with the systematic review's core requirements.	1.4	Exclude	Exclude	No tabular data
Semi-supervised task-driven data augmentation for medical image segmentation	Chaitanya K., Karani N., Baumgartner C.F., Erdil E., Becker A., Donati O., Konukoglu E.	2021	no	The paper focuses on technical aspects of image segmentation and data augmentation rather than clinical applications. While it's in a medical context, the focus is on the technical methodology.	no	The study deals with medical imaging data (cardiac, prostate, and pancreas images), not tabular data.	yes	The study uses human clinical imaging data from cardiac, prostate, and pancreas datasets.	yes	This appears to be an original research article with novel methodology and experimental results.	yes	The study involves active creation of synthetic data using AI methods for data augmentation.	maybe	While the study does generate synthetic data using AI methods, it's for image augmentation rather than tabular data generation.	no	The study focuses on improving segmentation methodology rather than demonstrating direct clinical applications like diagnosis or treatment planning.	yes	The study includes empirical evaluation on three medical datasets with clear comparative metrics.	yes	The study reports comparative performance metrics against standard augmentation and semi-supervised approaches.	Exclude	The study focuses on technical image segmentation methodology using synthetic data generation, which fundamentally diverges from the required clinical application and tabular data criteria. Despite involving human clinical imaging data and demonstrating empirical evaluation, the research fails to meet key screening requirements related to clinical focus and data format. Consequently, the paper does not align with the specified screening criteria for inclusion.	1.4	Exclude	Exclude	No tabular data
SimPET-An open online platform for the Monte Carlo simulation of realistic brain PET data. Validation for 18F-FDG scans	Paredes-Pacheco J., Lopez-Gonzalez F.J., Silva-Rodriguez J., Efthimiou N., Ninerola-Baizan A., Ruibal A., Roe-Vellve N., Aguiar P.	2021	maybe	The paper focuses on technical aspects of PET image simulation rather than direct clinical applications. While it has clinical relevance, it's primarily a methodological paper about creating synthetic PET data.	no	The study focuses on imaging data (PET scans), not tabular data.	yes	Yes, the study uses real human clinical data from 25 healthy subjects as a basis for validation.	yes	Yes, this appears to be an original research article with clear methodology and results.	no	The study does involve active creation of synthetic data, but uses Monte Carlo simulation (SimSET) rather than AI methods.	no	While the study generates synthetic data, it uses Monte Carlo simulation rather than AI methods like GANs or VAEs.	maybe	The paper focuses on technical validation rather than direct clinical applications. While it mentions potential use for "converting control subject databases," it doesn't demonstrate direct clinical applications.	yes	Yes, the study includes original empirical evaluation with clear metrics for assessing the synthetic data's quality.	yes	Yes, the study reports quantifiable outcomes including Bland-Altman differences and SPM voxel-wise comparisons.	Exclude	The study focuses on PET image simulation using Monte Carlo methods, which fundamentally differs from the required AI-driven tabular data generation approach. Critical screening criteria related to data format, synthetic data generation method, and direct clinical applications are not met, rendering the paper unsuitable for the systematic review. Despite demonstrating empirical evaluation and using human clinical data, the core methodological approach disqualifies the study from inclusion.	1.4	Exclude	Exclude	No tabular data
SinGAN-Seg: Synthetic training data generation for medical image segmentation	Thambawita V., Salehi P., Sheshkal S.A., Hicks S.A., Hammer H.L., Parasa S., de Lange T., Halvorsen P., Riegler M.A.	2022	no	The paper focuses primarily on technical aspects of image generation and segmentation, rather than clinical applications. While it mentions supporting doctors, it doesn't describe specific clinical applications.	no	The study explicitly focuses on medical images and segmentation masks, not tabular data. This is clearly stated throughout the abstract.	maybe	While the study mentions medical data, it doesn't explicitly state whether it uses human clinical data. The abstract mentions using an "open dataset" but doesn't specify if it's human clinical data.	yes	This appears to be an original research article with novel methodology and empirical evaluation.	yes	The study involves active creation of synthetic data using AI (SinGAN-Seg pipeline), not just using pre-existing synthetic datasets.	yes	The study uses AI methods (specifically a GAN variant) for synthetic data generation.	no	The study focuses on technical performance of segmentation models rather than direct clinical applications. No specific clinical use cases are described.	yes	The study includes clear empirical evaluation with both qualitative and quantitative comparisons.	yes	The study reports quantifiable outcomes in terms of model performance comparisons between real and synthetic data.	Exclude	The study focuses on a technical approach to synthetic medical image generation using AI, primarily addressing methodological challenges in data creation rather than direct clinical applications. While demonstrating innovative synthetic data generation techniques, the research fails to meet key screening criteria, particularly around tabular data format and clinical utility. The paper's emphasis on technical performance and image segmentation methodology disqualifies it from the systematic review's requirements.	1.4	Exclude	Exclude	No tabular data
Single-shell NODDI using dictionary-learner-estimated isotropic volume fraction	Faiyaz A., Doyley M., Schifitto G., Zhong J., Uddin M.N.	2022	maybe	The paper focuses on technical aspects of MRI data processing and analysis rather than direct clinical applications. While it has potential clinical implications, the primary focus is on the technical methodology of NODDI parameter estimation.	no	The study deals with imaging data (diffusion MRI) rather than tabular data.	yes	The study does use human clinical data (both HCP and clinical dataset).	yes	Yes, this appears to be an original research article with novel methodology and empirical evaluation.	no	While the study uses AI (dictionary learning approach), it's not creating synthetic clinical data for clinical use, but rather using AI to process and analyze existing imaging data.	no	The study does use synthetic data simulation, but not for generating tabular clinical data. Instead, it's used for testing the imaging analysis method.	maybe	While the method could have clinical applications, the abstract doesn't demonstrate direct clinical applications. It focuses on technical validation of the imaging analysis method.	maybe	The study includes empirical evaluation with clear metrics, but these are focused on technical performance rather than clinical utility.	maybe	The study reports quantifiable outcomes (error ~5% in white and gray matter), but these are technical metrics rather than clinical outcomes.	Exclude	The study primarily focuses on technical MRI data processing methodology using AI, which diverges significantly from the screening criteria's emphasis on clinical applications and tabular data generation. While the research involves human clinical data and includes empirical evaluation, it fails to meet key requirements around synthetic data creation, direct clinical outcomes, and tabular data format. Consequently, the paper does not align with the specified screening criteria for clinical synthetic data research.	1.4	Exclude	Exclude	No tabular data
Single-shot T2 mapping via multi-slice information sharing based on switching modulation patterns multiple overlapping-echo detachment imaging	Dai C., Yang Q., Zhou J., Zhu L., Lin L., Wang J., Cai C., Cai S.	2025	maybe	The paper focuses on improving MRI imaging techniques, specifically T2 mapping, which has clinical applications, but the primary focus appears to be technical/methodological rather than clinical.	no	The study does not focus on tabular data generation, but rather deals with imaging data (MRI) and uses synthetic data for training a U-Net model for image processing.	yes	Yes, the study uses human clinical data, including experiments on healthy volunteers and patients with meningioma or glioblastoma.	yes	Yes, this is clearly an original research article with detailed methodology and results.	maybe	The study uses AI (U-Net) for training, but the synthetic data creation is not the primary focus. The synthetic data is used as a tool for training rather than being the main subject of study.	no	While the study uses synthetic data (3000/7000 synthetic data with geometric/brain patterns), it's not focused on generating synthetic tabular data. The synthetic data is used for training an image processing model.	maybe	The study demonstrates potential clinical applications in MRI imaging, but these are secondary to the technical improvements being proposed.	yes	Yes, the study includes thorough empirical evaluation with clear metrics and statistical analyses.	yes	Yes, the study reports quantifiable outcomes including SSIM, RMSE, and various statistical analyses.	Exclude	The study primarily focuses on technical improvements in MRI imaging techniques, using synthetic data for training an image processing model, which diverges from the systematic review's core criteria of synthetic tabular data generation. While the research demonstrates clinical potential and includes empirical evaluation with human data, it fundamentally does not align with the review's specific requirements for tabular data generation and synthetic data methodology.	1.4	Exclude	Exclude	No tabular data
Sleep Quality, Nutrient Intake, and Social Development Index Predict Metabolic Syndrome in the Tlalpan 2020 Cohort: A Machine Learning and Synthetic Data Study.	Gutierrez-Esparza, Guadalupe, Martinez-Garcia, Mireya, Ramirez-delReal, Tania, Groves-Miralrio, Lucero Elizabeth, Marquez, Manlio F, Pulido, Tomas, Amezcua-Guerra, Luis M, Hernandez-Lemus, Enrique	2024	maybe	The study focuses on clinical applications (Metabolic Syndrome prediction and analysis) rather than purely technical aspects, but it appears to be more of an analytical study than a direct clinical application.	yes	The study appears to use tabular data (patient characteristics, nutrients, social factors) and doesn't mention any non-tabular formats.	yes	The study uses human clinical data from a Mexico City cohort, including patient characteristics and health outcomes.	yes	This appears to be an original research article with empirical analysis and results.	no	The abstract doesn't mention any synthetic data generation. Instead, it uses real data from a Mexico City cohort and applies data balancing techniques (SMOTE, ADASYN).	no	No evidence of AI-based synthetic data generation. The study uses real data and traditional data balancing techniques.	maybe	While the study has clinical relevance, it's more focused on analysis and prediction rather than direct clinical applications like diagnosis or treatment planning.	yes	The study includes empirical evaluation with clear metrics and analysis.	yes	The study reports quantifiable outcomes (balanced accuracy of 87%) and identifies specific predictors.	Exclude	The study focuses on Metabolic Syndrome prediction using real clinical data from a Mexico City cohort, employing machine learning techniques to analyze patient characteristics and health outcomes. While demonstrating strong empirical research with quantifiable results, the paper does not involve synthetic data generation or AI-driven data creation, which are critical screening requirements. Consequently, the study fails to meet key criteria related to synthetic data generation and direct clinical application.	1.4	Exclude	Exclude	No synthetic data generation
Spectral laplace-beltrami wavelets with applications in medical images	Tan M., Qiu A.	2015	maybe	The paper focuses on technical aspects (spectral graph wavelet transform and Laplace-Beltrami operator) rather than clinical applications. While it has a clinical application in Alzheimer's classification, this appears to be a secondary demonstration rather than the primary focus.	no	The study primarily deals with geometric and shape data (hippocampal shapes), not tabular data.	yes	Yes, the study uses human clinical data (hippocampal shapes from Alzheimer's patients and normal subjects).	yes	Yes, this appears to be an original research article with novel technical contributions.	no	The paper does not involve creating synthetic data using AI. While it mentions using synthetic data, this appears to be for technical validation rather than AI-generated synthetic data creation.	no	The study does not involve generating synthetic tabular data using AI methods. The synthetic data mentioned is for technical validation of wavelet transforms.	maybe	While there is a clinical application (Alzheimer's classification), this appears to be a demonstration of the technical method rather than the primary focus of the paper.	yes	Yes, the study includes empirical evaluation with clear metrics.	yes	Yes, the study reports quantifiable outcomes (96% sensitivity and 90% specificity for classification).	Exclude	The study primarily focuses on technical mathematical methods for wavelet transforms and geometric data analysis, with a secondary clinical application in Alzheimer's classification. While the research involves human clinical data and reports quantifiable outcomes, it fundamentally fails key screening criteria related to tabular data generation and AI-driven synthetic data creation. The paper's technical orientation and non-tabular data format disqualify it from meeting the specified screening requirements.	1.4	Exclude	Exclude	No tabular data
Survey on sampling conditioned brain images and imaging measures with generative models	Cheong S., Lee H., Kim W.H.	2025	no	The paper appears to be a review/overview of generative modeling techniques rather than focusing on specific clinical applications. While it mentions potential clinical applications, it's primarily a technical/methodological review.	no	The paper focuses on brain imaging data, which is explicitly excluded by the criteria as it's not tabular data.	maybe	While it mentions clinical applications and human brain imaging, it's not clear if the paper itself works with human clinical data.	no	The abstract explicitly states this is a "comprehensive overview" paper, indicating it's a review rather than original research.	maybe	While the paper discusses AI-based synthetic data generation, it's not clear if it involves original data creation or just reviews existing methods.	yes	The paper clearly discusses AI methods (VAEs, GANs, diffusion models) for synthetic data generation.	maybe	The paper mentions potential clinical applications (diagnostic accuracy, personalized treatments) but appears to be discussing these generally rather than demonstrating them directly.	no	As a review paper, it doesn't appear to include original empirical evaluation.	no	The abstract doesn't mention any specific quantifiable outcomes or metrics.	Exclude	The systematic review criteria were not met due to the paper being a methodological review focused on brain imaging data, which falls outside the specified requirements for tabular clinical data and original research. Multiple key screening criteria were failed, including the need for empirical evaluation, quantifiable outcomes, and direct clinical application, rendering the paper ineligible for inclusion.	1.4	Exclude	Exclude	Wrong publication type
Synthesis and quality assessment of combined time-series and static medical data using a real-world time-series generative adversarial network	Kim J., Choo H., Shin S.-Y., Song K.D.	2024	yes	The study focuses on clinical applications, specifically colorectal cancer survival prediction and medical data synthesis for clinical use.	maybe	The abstract explicitly mentions "time-series data" which is specifically excluded in the criteria.	yes	The study uses real-world data from 15,799 patients with colorectal cancer.	yes	Based on the detailed methodology and results presented, this appears to be an original research article.	yes	The study actively creates synthetic data using RTSGAN, generating 53,005 synthetic data points from 15,799 real patient records.	yes	The study uses GANs (specifically RTSGAN) for synthetic data generation.	yes	The study demonstrates clinical application in predicting five-year survival in colorectal cancer patients.	yes	The study includes comprehensive empirical evaluation with both quantitative and qualitative methods.	yes	The study reports multiple quantifiable metrics including AUC scores (0.99 and 0.98), Hellinger distance (0-0.25), and propensity mean squared error (0.223).	Exclude	The study demonstrates strong alignment with most screening criteria, including original research, clinical application, and empirical evaluation of synthetic data generation for colorectal cancer survival prediction. However, the use of time-series data represents a critical disqualifying factor, as this format is explicitly excluded from the screening requirements. Despite comprehensive methodology and quantifiable outcomes, the data format prevents the study from meeting all specified criteria.	1.4	Exclude	Exclude	No tabular data

Synthesis of CT images from digital body phantoms using CycleGAN.	Russ, Tom, Goerttler, Stephan, Schnurr, Alena-Kathrin, Bauer, Dominik F, Hatamikia, Sepideh, Schad, Lothar R, Zollner, Frank G, Chung, Khanlian	2019	maybe	The paper focuses on technical aspects of image synthesis rather than direct clinical applications. While it has potential clinical implications, the primary focus is on the technical methodology of generating synthetic CT images.	maybe	The study focuses on medical imaging (CT scans), not tabular data. This is explicitly clear from the abstract's focus on image synthesis and analysis.	maybe	The study uses XCAT body phantoms rather than real human clinical data. While these are anatomically accurate models, they don't constitute actual human clinical data.	yes	This appears to be an original research article with clear methodology and results.	yes	The study does involve active creation of synthetic data using AI (specifically CycleGAN), meeting this criterion.	maybe	While the study does use AI methods (CycleGAN) for synthetic data generation, it's for images rather than tabular data.	maybe	The study demonstrates a technical application (blood vessel segmentation) but doesn't directly address clinical applications like diagnosis or treatment planning.	yes	The study includes clear empirical evaluation with specific metrics and comparisons.	yes	The study reports quantifiable outcomes (dice similarity coefficient, noise power spectrum correlation coefficient, annotation accuracy).	Exclude	The study focuses on technical image synthesis using CycleGAN, primarily addressing methodological aspects of CT image generation rather than direct clinical applications. While demonstrating sophisticated AI techniques for synthetic data creation, the research uses XCAT phantoms and imaging data, which diverges from the specified screening criteria of tabular clinical data generation. Consequently, the paper does not align with the core requirements for inclusion in a review of clinical synthetic data generation.	1.4	Exclude	Exclude	No tabular data
Synthesizing CTA Image Data for Type-B Aortic Dissection using Stable Diffusion Models	Abaid A., Ali Farooq M., Hynes N., Corcoran P., Ullah I.	2024	maybe	The study focuses on generating synthetic cardiac CTA images for cardiovascular image processing, which has clinical applications. However, the primary focus appears to be on the technical aspects of image generation rather than direct clinical applications.	no	The study focuses on medical imaging data (CTA images), not tabular data.	yes	The study uses real cardiac CTA images as input, which implies human clinical data.	yes	This appears to be an original research article with novel implementation and evaluation.	yes	The study does involve active creation of synthetic data using AI (Stable Diffusion).	maybe	While the study does use AI methods (Stable Diffusion) for synthetic data generation, it's for images rather than tabular data.	maybe	While the study mentions cardiovascular image processing, it doesn't clearly demonstrate direct clinical applications like diagnosis or treatment planning.	yes	The study includes both quantitative and qualitative assessment by clinicians, indicating empirical evaluation.	maybe	The abstract mentions "quantitative analysis" but doesn't specify the metrics used.	Exclude	The study focuses on generating synthetic cardiac CTA images using Stable Diffusion, which primarily involves medical imaging data rather than tabular data. While demonstrating original research with empirical evaluation and human clinical data, the methodology fundamentally conflicts with the screening criteria's requirement for tabular data generation. Consequently, the research does not meet the essential screening requirements for inclusion.	1.4	Exclude	Exclude	No tabular data
Synthetic data & the future of Women's Health: A synergistic relationship	Delanerolle G., Phiri P., Cavalini H., Benfield D., Shetty A., Bouchareb Y., Shi J.Q., Zemkoho A.	2023	maybe	The paper discusses clinical applications in women's health, but appears to be more of a perspective piece about the potential of synthetic data rather than a specific clinical application.	maybe	The abstract doesn't specify the type of data being generated. While it mentions "clinical characteristics," it doesn't explicitly state whether this is tabular data.	yes	The paper focuses on women's health and clinical characteristics, indicating use of human clinical data.	no	This is explicitly described as a "perspective" piece, not an original research article.	no	The paper appears to be a perspective piece discussing the potential of synthetic data rather than actively creating synthetic data.	maybe	While the paper discusses synthetic data generation using AI techniques, it doesn't appear to implement these methods directly.	maybe	While it discusses potential clinical applications, particularly in women's health and epidemiology, it doesn't demonstrate specific clinical applications.	no	As a perspective piece, it doesn't include original empirical evaluation.	no	The abstract doesn't mention any specific quantifiable outcomes or metrics.	Exclude	A perspective piece discussing synthetic data in women's health that lacks original research, empirical evaluation, and quantifiable outcomes. While exploring potential clinical applications and AI techniques for data generation, the study does not meet key screening criteria for an original research article with direct clinical implementation. The abstract provides a conceptual overview but falls short of demonstrating concrete synthetic data generation or clinical utility.	1.4	Exclude	Exclude	Wrong publication type
Synthetic data for annotation and extraction of family history information from clinical text.	Brekke, Pal H, Rama, Taraka, Pilan, Ildiko, Nytro, Oystein, Ovrelid, Lilja	2021	no	The study focuses on NLP methodology and text processing rather than direct clinical applications. While it deals with clinical text, the primary focus is on developing NLP techniques.	no	The study deals with text data (clinical notes) rather than tabular data. This is explicitly clear from the abstract's focus on "clinical text" and "Natural Language Processing."	yes	The study uses human clinical data, specifically "de-identified records for patients with genetic heart disease."	yes	This appears to be an original research article with clear methodology and results.	yes	The study involves active creation of synthetic data, specifically "synthetically produced clinical statements."	maybe	While synthetic data is created, the abstract doesn't specify the AI methods used (GANs, VAEs, etc.). It appears to be more focused on text generation for NLP purposes.	no	The study focuses on NLP methodology for text processing rather than direct clinical applications like diagnosis or treatment planning.	yes	The study includes empirical evaluation with clear metrics and comparison between synthetic and real clinical data.	yes	The study reports clear quantifiable outcomes (precision, recall, F1-scores for entity recognition and relation extraction).	Exclude	The study focuses on Natural Language Processing methodology for clinical text processing, primarily using synthetic text generation for NLP techniques rather than direct clinical applications. While the research involves human clinical data and demonstrates empirical evaluation with quantifiable outcomes, it fails key screening criteria by using text data instead of tabular data and lacking direct clinical utility. The methodology's primary purpose is NLP technique development, which disqualifies it from meeting the systematic review's core requirements.	1.4	Exclude	Exclude	No tabular data
Synthetic data in medicine: Legal and ethical considerations for patient profiling	Nisevic M., Milojevic D., Spajic D.	2025	no	The paper focuses primarily on legal, ethical, and regulatory aspects of synthetic data rather than clinical applications. While it mentions clinical applications, these are discussed in the context of regulatory frameworks rather than direct clinical implementation.	maybe	The abstract doesn't specify the type of data being generated. While it mentions "patient profiling," it doesn't explicitly state whether this involves tabular data or other formats.	no	The paper discusses patient data in the context of profiling, but doesn't appear to directly work with human clinical data.	yes	This appears to be an original research article analyzing legal and ethical frameworks, though not in the clinical sense we're looking for.	no	The paper appears to be a legal-ethical analysis rather than a study involving active creation of synthetic data. It discusses synthetic data generation methods but doesn't implement them.	no	While the paper discusses synthetic data generation methods, it doesn't appear to actually implement or evaluate these methods. It's more focused on regulatory frameworks.	no	While clinical applications are mentioned, they're discussed in the context of regulatory frameworks rather than direct clinical implementation or evaluation.	no	There's no indication of empirical evaluation or clinical utility metrics. The paper appears to be a theoretical analysis of legal and ethical implications.	no	The paper doesn't appear to report any quantifiable clinical outcomes. It focuses on legal and ethical frameworks rather than performance metrics.	Exclude	The paper primarily focuses on legal and ethical frameworks for synthetic data, failing to meet key screening criteria related to clinical applications, data generation, and empirical evaluation. Despite discussing patient profiling and regulatory contexts, the study lacks direct clinical implementation, quantifiable outcomes, and original synthetic data creation. Consequently, the abstract does not align with the specified research requirements for clinical synthetic data studies.	1.4	Exclude	Exclude	No synthetic data generation
Synthetic Genitourinary Image Synthesis via Generative Adversarial Networks: Enhancing Artificial Intelligence Diagnostic Precision	Van Booven D.J., Chen C.-B., Malpani S., Mirzabeigi Y., Mohammadi M., Wang Y., Kryvenko O.N., Punnen S., Arora H.	2024	no	The study focuses on technical aspects of generating synthetic images for computational pathology, rather than direct clinical applications.	no	The study deals with image data (histopathological images), not tabular data.	yes	The study uses human clinical data (genitourinary tissue samples).	yes	This appears to be an original research article with novel methodology and results.	yes	The study actively creates synthetic data using GANs.	maybe	While the study uses AI (GANs) for synthetic data generation, it's for images rather than tabular data.	no	While the study has potential clinical applications, it doesn't demonstrate direct clinical applications. The abstract mentions future work for "tissue classification, segmentation, and disease detection" but doesn't report actual clinical applications.	maybe	While there is empirical evaluation of the synthetic images, it's focused on technical validation rather than clinical utility.	maybe	The study reports some quantifiable metrics (RIS, FID, pathologist approval rating) but these are for image quality rather than clinical outcomes.	Exclude	The study focuses on generating synthetic histopathological images using GANs, which fundamentally deviates from the required tabular data format and lacks direct clinical application demonstration. Despite using human clinical data and employing AI for synthetic data generation, the research primarily addresses technical image generation challenges rather than meeting the specified screening criteria for clinical utility and data type.	1.4	Exclude	Exclude	No tabular data
Synthetic hardware performance analysis in virtualized cloud environment for healthcare organization	Tan C.-H., Teh Y.-W.	2013	no	The paper focuses on technical aspects (cloud computing, database operations, hardware performance) rather than clinical applications. It's primarily about IT infrastructure and performance monitoring.	maybe	The study uses TPC-H benchmark data, which is synthetic tabular data. However, this is not healthcare-specific data.	no	The study does not use human clinical data or patient-related healthcare information. It focuses on IT infrastructure and performance monitoring.	yes	This appears to be an original research article with empirical evaluation.	no	The paper uses pre-existing synthetic data (TPC-H benchmark) rather than actively creating new synthetic data using AI methods.	no	While synthetic data is used, it's not generated using AI methods. The TPC-H benchmark is a standard database benchmarking tool.	no	The paper does not demonstrate direct clinical applications. It's focused on IT infrastructure and database performance.	no	The study includes empirical evaluation, but not of clinical utility. The evaluation focuses on IT performance metrics.	no	While the paper includes performance metrics, these are related to IT infrastructure rather than clinical outcomes.	Exclude	The study focuses on IT infrastructure and database performance monitoring using synthetic benchmark data, failing to meet most clinical research screening criteria. Specifically, it lacks human clinical data, AI-driven synthetic data generation, clinical applications, and quantifiable clinical outcomes. The research is primarily a technical exploration of cloud computing performance rather than a clinical investigation.	1.4	Exclude	Exclude	No synthetic data generation
Synthetic Lung Ultrasound Data Generation Using Autoencoder With Generative Adversarial Network.	Fatima, Noreen, Mento, Federico, Afrakhteh, Sajjad, Perrone, Tiziano, Smargiassi, Andrea, Inchingolo, Riccardo, Demi, Libertario	2025	maybe	The study focuses on technical aspects of data augmentation and AI model performance rather than direct clinical applications. While it's in a medical context, the primary focus is on the technical methodology.	maybe	The study deals with lung ultrasound images, not tabular data. This is explicitly stated in the abstract and is a clear violation of this criterion.	yes	The study uses lung ultrasound data from human patients, though this is somewhat implicit in the context.	yes	Based on the abstract, this appears to be an original research article with novel methodology and empirical evaluation.	yes	The study actively creates synthetic data using AI (specifically a SA-GAN), not using pre-existing synthetic datasets.	maybe	While the study does use AI methods (GANs) for synthetic data generation, it's for images rather than tabular data.	maybe	The study focuses on technical aspects of data augmentation and classification performance rather than direct clinical applications like diagnosis or treatment planning.	yes	The study includes original research with empirical evaluation, comparing GAN-based augmentation with traditional techniques.	yes	The study reports quantifiable outcomes through similarity metrics and classification performance comparisons.	Exclude	The study focuses on technical image data augmentation using AI, which fundamentally conflicts with the screening criteria requiring tabular data and direct clinical applications. Despite demonstrating original research with empirical evaluation, the methodology centers on image processing and machine learning techniques rather than clinical utility, rendering it non-compliant with the specified screening requirements.	1.4	Exclude	Exclude	No tabular data
Synthetic Medical Images for Robust, Privacy-Preserving Training of Artificial Intelligence: Application to Retinopathy of Prematurity Diagnosis	Coyner A.S., Chen J.S., Chang K., Singh P., Ostmo S., Chan R.V.P., Chiang M.F., Kalpathy-Cramer J., Campbell J.P.	2022	no	The study focuses on technical aspects of AI model training and synthetic data generation for medical imaging, rather than direct clinical applications.	no	The study deals with medical imaging data (retinal fundus images) and vessel maps, not tabular data.	yes	The study uses real clinical data from 963 preterm infants.	yes	This appears to be an original research article with clear methodology and results.	yes	The study actively creates synthetic data using PGANs (Progressively growing generative adversarial networks).	no	While the study does generate synthetic data using AI (PGANs), it's for images rather than tabular data.	maybe	While the study has potential clinical implications for ROP diagnosis, it focuses more on the technical validation of synthetic data generation rather than direct clinical applications.	yes	The study includes thorough empirical evaluation with clear metrics and comparison against expert performance.	yes	The study reports clear quantifiable outcomes including AUC scores, kappa values, and other metrics.	Exclude	The study focuses on synthetic medical image generation using AI, which fundamentally deviates from the required tabular data format and clinical application focus. While demonstrating sophisticated technical methodology in generating synthetic retinal images, the research fails to meet critical screening criteria related to data type and direct clinical utility. Consequently, the paper does not align with the systematic review's core requirements.	1.4	Exclude	Exclude	No tabular data
Synthetic Patient-Physician Conversations Simulated by Large Language Models: A Multi-Dimensional Evaluation.	Haider, Syed Ali, Prabha, Srinivasagam, Gomez-Cabello, Cesar Abraham, Borna, Sahar, Genovese, Ariana, Trabilsy, Maissa, Collaco, Bernardo G, Wood, Nadia G, Bagaria, Sanjay, Tao, Cui, Forte, Antonio Jorge	2025	maybe	The study focuses on generating synthetic clinical conversations, which is more educational/methodological than clinical application. While it has healthcare implications, it's primarily about the technical generation of synthetic dialogue.	no	The study focuses on generating text-based dialogue transcripts, not tabular data.	yes	The study uses human clinical scenarios (plastic surgery) as its basis, though it's generating synthetic versions of these scenarios.	yes	The study appears to be an original research article with clear methodology and results.	yes	The study actively creates synthetic data using AI (specifically LLMs) rather than using pre-existing datasets.	no	While the study does generate synthetic data using AI, it's generating text-based dialogue rather than tabular data.	maybe	The study focuses on educational and research applications rather than direct clinical applications like diagnosis or treatment planning.	yes	The study includes clear empirical evaluation with multiple raters, statistical analysis, and specific metrics.	yes	The study reports quantifiable outcomes through a seven-criterion rubric with 5-point Likert scales and statistical analysis.	Exclude	The study generates synthetic clinical dialogue using Large Language Models, focusing on educational and research applications rather than direct clinical utility. While demonstrating rigorous methodology and empirical evaluation, the research fails to meet key screening criteria by producing text-based dialogue instead of tabular data and lacking direct clinical application. The work provides insights into AI-generated medical conversations but does not align with the specified research parameters.	1.4	Exclude	Exclude	No tabular data
Synthetic Versus Classic Data Augmentation: Impacts on Breast Ultrasound Image Classification	Medghalchi Y., Zakariaei N., Rahmim A., Hacihaliloglu I.	2025	no	The study focuses on technical aspects of data augmentation and image analysis rather than direct clinical applications. While it's in a medical context, the primary focus is on comparing technical methods.	no	The study deals with ultrasound images, not tabular data. This is explicitly stated multiple times in the abstract.	yes	The study uses human clinical data (breast ultrasound images).	yes	This appears to be an original research article with empirical evaluation.	yes	The study involves active creation of synthetic data using AI methods (specifically GANs and diffusion-based models).	no	While the study does involve synthetic data generation using AI methods, it's for images rather than tabular data.	maybe	While the ultimate goal is breast cancer diagnosis, the study focuses on comparing technical methods rather than demonstrating direct clinical applications.	yes	The study includes empirical evaluation comparing different augmentation techniques.	maybe	The abstract mentions "diagnostic accuracy" but doesn't specify the metrics that will be used.	Exclude	The study focuses on technical aspects of ultrasound image analysis using synthetic data generation, which fundamentally conflicts with the screening criteria's requirements for tabular data and direct clinical applications. While the research involves human clinical data and AI methods, its primary emphasis on image-based data augmentation and technical methodology disqualifies it from meeting the specified screening requirements.	1.4	Exclude	Exclude	No tabular data

Synthetically enhanced: unveiling synthetic data's potential in medical imaging research	Khosravi B., Li F., Dapamede T., Rouzrokh P., Gamble C.U., Trivedi H.M., Wyles C.C., Sellergren A.B., Purkayastha S., Erickson B.J., Gichoya J.W.	2024	no	The study focuses on technical aspects of image generation and model generalizability rather than direct clinical applications. While it uses medical imaging, the primary focus is on the technical methodology.	no	The study deals with medical imaging (Chest X-rays) rather than tabular data. This is explicitly clear from the abstract's focus on CXR images and image generation.	yes	The study uses human clinical data from multiple datasets (CheXpert, MIMIC-CXR, and Emory Chest X-ray).	yes	This appears to be an original research article with clear methodology and results.	yes	The study actively creates synthetic data using AI (specifically DDPMs) rather than using pre-existing synthetic datasets.	no	While the study does use AI for synthetic data generation, it generates images rather than tabular data. The method (DDPMs) is appropriate but the data format is not.	maybe	While the study involves pathology classification, it focuses more on the technical aspects of improving model generalizability rather than direct clinical applications.	yes	The study includes original research with clear empirical evaluation and metrics.	yes	The study reports clear quantifiable outcomes using AUROC values and statistical significance.	Exclude	The study focuses on generating synthetic chest X-ray images using AI, which fundamentally deviates from the required tabular data format and clinical application focus. While demonstrating technical sophistication in medical imaging and synthetic data generation, the research fails to meet key screening criteria related to data type and direct clinical utility. The methodology, though rigorous, does not align with the specified research parameters.	1.4	Exclude	Exclude	No tabular data
Synthetically trained convolutional neural networks for improved tensor estimation from free-breathing cardiac DTI	Weine J., van Gorkum R.J.H., Stoeck C.T., Vishnevskiy V., Kozerke S.	2022	maybe	The paper focuses on clinical applications (cardiac imaging and tensor estimation) rather than purely technical aspects, but it has a strong technical component in developing the CNN method.	no	The study deals with imaging data (cDTI) and tensor data, not tabular data, which is a clear violation of the criterion.	yes	The study uses human clinical data from "five healthy subjects" and includes in-vivo data.	yes	This is clearly an original research article with novel methodology and empirical evaluation.	maybe	The study does involve active creation of synthetic data using AI (CNN), but it's for imaging data rather than tabular data.	maybe	While the study does generate synthetic data using AI methods (CNN), it's specifically for imaging data rather than tabular data.	yes	The study demonstrates direct clinical applications in cardiac imaging and tissue classification.	yes	The study includes clear empirical evaluation with specific metrics and comparison to conventional methods.	yes	The study reports quantifiable outcomes including "tensor estimation accuracy and precision" and classification performance.	Exclude	The study focuses on cardiac imaging and tensor estimation using synthetic data generation, but critically fails to meet the requirement of tabular data format. While demonstrating strong clinical applications and empirical evaluation, the research primarily involves imaging data, which disqualifies it from meeting the core screening criteria. The technical approach and clinical relevance are robust, but the data type fundamentally misaligns with the specified requirements.	1.4	Exclude	Exclude	No tabular data
SynthEye: Investigating the Impact of Synthetic Data on Artificial Intelligence-assisted Gene Diagnosis of Inherited Retinal Disease	Veturi Y.A., Woof W., Lazebnik T., Moghul I., Woodward-Court P., Wagner S.K., Cabral de Guimaraes T.A., Daich Varela M., Liefers B., Patel P.J., Beck S., Webster A.R., Mahroo O., Keane P.A., Michaelides M., Balaskas K., Pontikos N.	2023	no	The study focuses on technical aspects of image generation and classification rather than direct clinical applications. While it's in a medical context, the primary focus is on the AI methodology.	no	The study deals with medical imaging data (fundus autofluorescence images), not tabular data. This is explicitly stated in the abstract and is a critical mismatch with the criteria.	yes	The study uses real clinical data from 1800 patients with confirmed genetic diagnosis of IRD genes.	yes	This appears to be an original research article with detailed methodology and results.	yes	The study actively creates synthetic data using StyleGAN2, a generative adversarial network.	yes	The study uses AI methods (specifically GANs) for synthetic data generation.	maybe	While the study is in a clinical context (IRD diagnosis), it focuses more on the technical aspects of synthetic data generation and classification rather than direct clinical applications.	yes	The study includes thorough empirical evaluation with clear metrics and comparison against baseline models.	yes	The study reports clear quantifiable outcomes including AUROC, Cohen's Kappa, and BRISQUE scores.	Exclude	The study focuses on synthetic data generation for inherited retinal disease diagnosis using medical imaging, which fundamentally conflicts with the requirement for tabular data. While demonstrating sophisticated AI techniques and using human clinical data, the research primarily addresses technical methodological aspects rather than direct clinical applications. Consequently, the paper fails to meet critical screening criteria related to data format and clinical application focus.	1.4	Exclude	Exclude	No tabular data
SynthSeg: Segmentation of brain MRI scans of any contrast and resolution without retraining	Billot B., Greve D.N., Puonti O., Thielscher A., Van Leemput K., Fischl B., Dalca A.V., Iglesias J.E.	2023	maybe	The paper focuses on medical image segmentation, which is a technical application. While it has clinical implications, the primary focus appears to be on the technical methodology rather than direct clinical applications.	no	The study deals with medical imaging data (MRI, CT scans), not tabular data.	yes	The study uses human clinical data (brain and cardiac scans from diverse populations).	yes	This appears to be an original research article presenting a new method (SynthSeg).	yes	The study does involve active creation of synthetic data using AI (SynthSeg).	no	While the study does generate synthetic data using AI, it's for image segmentation rather than tabular data generation.	no	While the method has clinical applications in image segmentation, the abstract doesn't demonstrate direct clinical applications like diagnosis or treatment planning. It focuses more on the technical aspects of segmentation.	maybe	The study includes empirical evaluation with 5,000 scans across multiple modalities, but the evaluation appears to be technical rather than clinical.	no	The abstract mentions comparing performance with other methods but doesn't explicitly state clinical metrics. It focuses more on technical performance metrics.	Exclude	The study focuses on medical image segmentation using synthetic data generation, but fails key screening criteria by using imaging data instead of tabular data and emphasizing technical methodology over direct clinical applications. While demonstrating innovative AI techniques for medical imaging, the research does not meet the specific requirements for clinical data generation and quantifiable clinical outcomes.	1.4	Exclude	Exclude	No tabular data
Towards cross-modal organ translation and segmentation: A cycle- and shape-consistent generative adversarial network.	Cai, Jinzheng, Zhang, Zizhao, Cui, Lei, Zheng, Yefeng, Yang, Lin	2019	maybe	The paper focuses on technical aspects of image synthesis and segmentation rather than direct clinical applications. While it mentions potential clinical uses, these are secondary to the technical methodology.	no	The study focuses on medical imaging data (CT, MRI, X-rays), not tabular data. This is explicitly clear from the abstract.	yes	The study uses human clinical imaging data (cardiovascular CT, abdominal CT, mammography X-rays).	yes	This appears to be an original research article with novel methodology and empirical evaluation.	yes	The study does involve active creation of synthetic data using AI (CNN-based generators).	maybe	While the study does use AI methods (CNNs) for synthetic data generation, it's for images rather than tabular data.	maybe	While the paper mentions potential clinical applications (cross-modality registration, training samples), it doesn't demonstrate direct clinical applications like diagnosis or treatment planning.	maybe	The study includes empirical evaluation, but the metrics are technical (image synthesis quality, segmentation performance) rather than clinical utility.	maybe	The abstract mentions performance evaluation but doesn't specify clinical metrics. The outcomes appear to be technical rather than clinical.	Exclude	The study focuses on medical image synthesis using AI techniques, primarily addressing technical methodological aspects rather than direct clinical applications. While the research involves human clinical imaging data and demonstrates original AI-driven data generation, it fails to meet key screening criteria related to tabular data, clinical utility, and quantifiable clinical outcomes. The paper's technical orientation and imaging-based approach significantly diverge from the specified screening requirements.	1.4	Exclude	Exclude	No tabular data
Towards markerless surgical tool and hand pose estimation.	Hein, Jonas, Seibold, Matthias, Bogo, Federica, Farshad, Mazda, Pollefeys, Marc, Furnstahl, Philipp, Navab, Nassir	2021	maybe	The paper focuses on surgical tool tracking and pose estimation, which is a clinical application. However, it appears to be more focused on the technical aspects of data generation and pose estimation rather than direct clinical applications.	no	The study deals with RGB image data and 3D pose estimation, not tabular data.	yes	The study uses surgical scenarios and real surgical data, which implies human clinical data.	yes	This appears to be an original research article presenting new methods and results.	maybe	The paper describes creating synthetic data through a rendering pipeline, which is AI-based. However, it's for image data, not tabular data.	no	While the paper does involve synthetic data generation using AI methods, it's specifically for RGB images and 3D pose estimation, not tabular data.	maybe	While the study has potential clinical applications in computer-assisted surgery, it focuses more on the technical aspects of data generation and pose estimation rather than direct clinical applications.	yes	The study includes empirical evaluation with clear metrics for assessing the performance of their methods.	yes	The study reports quantifiable outcomes (3D vertex error of 16.7mm on synthetic data and 13.8mm on real data).	Exclude	The study focuses on surgical tool tracking using RGB image data and 3D pose estimation, which fundamentally differs from the required tabular data generation criteria. While the research demonstrates technical innovation in synthetic data creation and includes empirical evaluation, it fails to meet key screening requirements related to data format and clinical application focus. Consequently, the paper does not align with the systematic review's specific methodological constraints.	1.4	Exclude	Exclude	No tabular data
Towards toxic and narcotic medication detection with rotated object detectors	Peng J., Wang F., Ma X., Chen Z., Fu Z., Hu Y., Zhou X., Wang L.	2023	maybe	The paper focuses on clinical applications (medication management and detection), but it's primarily a technical implementation of object detection. While it has clinical relevance, it's more focused on the technical solution than clinical outcomes.	no	The study uses image data (pictures of medication bottles) rather than tabular data. This clearly fails this criterion as it's specifically focused on computer vision and image processing.	maybe	The study uses hospital-collected images of medications, which is related to clinical settings but not directly patient-related healthcare information.	yes	This is clearly an original research article with novel methodology and empirical results.	maybe	While the study mentions using synthetic data, it's not clear if they actively created it using AI methods or just used existing synthetic data. The focus is on using this data for training rather than the creation process itself.	maybe	The abstract mentions using "six thousand synthetic data" but doesn't specify the method of generation. It appears to be using these data for training rather than focusing on the generation process.	maybe	While the application is in medication management, which is clinically relevant, the focus is more on the technical implementation of object detection rather than direct clinical decision-making.	yes	The study includes extensive experiments and clear evaluation metrics.	yes	The study reports clear metrics (mean average precision of 0.811, inference time <300ms).	Exclude	The study primarily focuses on a technical computer vision solution for medication detection using image data, which fundamentally conflicts with the requirement for tabular data generation. While demonstrating clinical relevance in medication management, the research fails to meet critical screening criteria, particularly the data format specification. The technical implementation and empirical evaluation, though robust, do not align with the specified research parameters.	1.4	Exclude	Exclude	No tabular data
Training and validating a treatment recommender with partial verification evidence	Unnikrishnan V., Puga C., Schleicher M., Niemann U., Langguth B., Schoisswohl S., Mazurek B., Cima R., Lopez-Escamez J.A., Kikidis D., Vellidou E., Pryss R., Schlee W., Spiliopoulou M.	2025	yes	The study focuses on clinical decision support systems for treatment recommendations, which is clearly a clinical application.	yes	The study appears to work with tabular data from clinical trials, though this isn't explicitly stated.	yes	The study uses data from 240+ tinnitus patients recruited across 5 clinical centers.	yes	This appears to be an original research article presenting a new method for training clinical decision support systems.	no	The abstract doesn't describe creating synthetic data. While it mentions synthetic data as a potential future direction, this isn't part of the current study.	no	The study doesn't involve generating synthetic data. It uses real RCT data and discusses synthetic data only as a future possibility.	yes	The study directly addresses clinical decision-making for treatment recommendations.	yes	The study includes empirical evaluation using RCT data and presents clear metrics for assessment.	yes	The study evaluates treatment effectiveness and mentions predictive performance metrics.	Exclude	The study presents a clinical decision support system using real-world clinical trial data for tinnitus treatment recommendations, demonstrating strong clinical application and empirical evaluation. However, the research does not involve synthetic data generation, which disqualifies it from meeting key screening criteria focused on AI-driven synthetic data creation. Despite its methodological rigor and clinical relevance, the absence of synthetic data generation prevents the study from meeting the core requirements.	1.4	Exclude	Exclude	No synthetic data generation
Training machine learning models with synthetic data improves the prediction of ventricular origin in outflow tract ventricular arrhythmias	Doste R., Lozano M., Jimenez-Perez G., Mont L., Berruezo A., Penela D., Camara O., Sebastian R.	2022	yes	The study focuses on clinical applications, specifically predicting the site of origin for ventricular arrhythmias before ablation procedures.	no	The study primarily deals with ECG signals, which are time-series data, not tabular data. This is a critical failure of a core criterion.	yes	The study uses patient-specific geometries and validates on real patient data from 365 patients.	yes	This appears to be an original research article with novel methodology and results.	yes	The study actively creates synthetic data through "detailed electrophysiological simulations" of OTVAs.	maybe	While the study generates synthetic data, it uses electrophysiological simulations rather than AI methods like GANs or VAEs. The machine learning component is used for classification, not data generation.	yes	The study has direct clinical applications in predicting site of origin for arrhythmias, which is crucial for ablation procedures.	yes	The study includes thorough empirical evaluation with cross-validation and testing on multiple clinical datasets.	yes	The study reports clear quantifiable outcomes including accuracy metrics (>0.96 for simulated data, 0.86 and 0.84 for real data).	Exclude	The study demonstrates strong clinical relevance in predicting ventricular arrhythmia site of origin through synthetic data generation and machine learning classification. However, the research fundamentally fails to meet the tabular data format requirement, as it primarily works with ECG time-series signals rather than tabular data. Despite excellent methodological rigor and quantifiable clinical outcomes, this critical deviation from the screening criteria prevents study inclusion.	1.4	Exclude	Exclude	No tabular data
Training of a deep learning based digital subtraction angiography method using synthetic data	Duan L., Eulig E., Knaup M., Adamus R., Lell M., Kachelriess M.	2024	no	The study focuses primarily on technical aspects of image processing and generation rather than direct clinical applications. While it has potential clinical utility, the focus is on the technical methodology.	no	The study deals with imaging data (DSA and fluoroscopic images) rather than tabular data. This is explicitly clear from the abstract's focus on image processing and generation.	yes	The study uses human clinical data, including whole-body CT scans and clinical fluoroscopic data.	yes	This appears to be an original research article with clear methodology and results.	yes	The study involves active creation of synthetic data using AI methods, specifically generating synthetic DSA image pairs using deep learning approaches.	maybe	While the study does generate synthetic data using AI methods, it's focused on imaging data rather than tabular data as required by criterion 2.	maybe	While the study has potential clinical applications in diagnosis, it's primarily focused on the technical aspects of image generation rather than direct clinical applications.	yes	The study includes original research with clear empirical evaluation and metrics.	yes	The study reports clear quantifiable outcomes including PSNR, SSIM, accuracy, precision, and Dice scores.	Exclude	The study focuses on generating synthetic digital subtraction angiography images using AI techniques, primarily addressing technical image processing challenges rather than direct clinical applications. While demonstrating sophisticated data generation and empirical evaluation, the research fails to meet key screening criteria by concentrating on imaging data and technical methodology instead of tabular clinical data. Consequently, the paper does not align with the specified screening requirements for clinical synthetic data research.	1.4	Exclude	Exclude	No tabular data

UC-NfNet: Deep learning-enabled assessment of ulcerative colitis from colonoscopy images	Turan M., Durmus F.	2022	yes	The study focuses on clinical applications, specifically the classification of ulcerative colitis severity through colonoscopy imaging for clinical decision-making.	maybe	The study focuses on imaging data (colonoscopy images) rather than tabular data, which is a critical failure of a core criterion.	yes	The study uses human clinical data from colonoscopy images of UC patients.	yes	This appears to be an original research article with novel methodology and results.	yes	The abstract mentions a "synthetic data generation pipeline," suggesting active creation of synthetic data.	yes	While the specific AI method isn't detailed, the study involves synthetic data generation for medical imaging.	yes	The study demonstrates direct clinical application in disease classification and diagnosis.	yes	The study includes empirical evaluation with clear metrics, including comparison with other models and a reader study with five gastroenterologists.	yes	The study reports quantifiable outcomes through classification performance metrics and inter-reader agreement studies.	Exclude	The study focuses on an AI-powered ulcerative colitis classification method using colonoscopy imaging, demonstrating strong clinical application and empirical evaluation. However, the research fundamentally fails to meet the screening criteria due to its use of imaging data instead of the required tabular data format. Despite robust methodology and quantifiable outcomes, this critical deviation disqualifies the study from meeting the specified screening requirements.	1.4	Exclude	Exclude	No tabular data
Ultrafast Simultaneous T1, T2, T2*, PD, DELTAB0, and B1 Mapping via Longitudinal Magnetization Controlled MOLED Acquisition	Chen W., Kang T., Lin Q., Guo X., Wu J., Li S., Zheng Y., Lin J., Lin L., Wang J., Qu X., Chen Z., Cai S., Cai C.	2025	maybe	The paper focuses on clinical applications (mqMRI for diagnosis and treatment), but it's primarily a technical/methodological paper about improving imaging acquisition methods. While it has clinical implications, the primary focus is on the technical solution.	no	The study deals with imaging data (MRI), not tabular data. This is a clear violation of the criterion.	yes	The study includes validation on human brains, meeting this criterion.	yes	Yes, this appears to be an original research article with novel methodology and results.	yes	The study does use AI (deep neural network) to generate synthetic data from Bloch simulation, meeting this criterion.	maybe	While synthetic data is used, it's for training an imaging reconstruction algorithm, not for generating tabular clinical data. This doesn't meet the intended spirit of the criterion.	maybe	While the paper has clinical applications (diagnosis and treatment), it's focused on the technical imaging methodology rather than direct clinical applications.	maybe	The study includes empirical evaluation with clear metrics, but again these are technical rather than clinical metrics.	maybe	The study reports quantifiable outcomes (acquisition speed, image quality metrics), but these are technical rather than clinical outcomes.	Exclude	The study focuses on a technical imaging methodology using synthetic data generation for MRI reconstruction, which fundamentally deviates from the required tabular data format. While demonstrating clinical potential and using human brain data, the research primarily addresses technical imaging challenges rather than direct clinical applications. Consequently, the paper does not meet the core screening criteria for inclusion in the systematic review.	1.4	Exclude	Exclude	No tabular data
Understanding glioblastoma invasion using physically-guided neural networks with internal variables	Ayensa-Jimenez J., Doweidar M.H., Sanz-Herrera J.A., Doblare M.	2022	no	The paper focuses on technical aspects (PGNNIV, microfluidic devices) rather than direct clinical applications. While it mentions potential clinical applications ("virtual therapies and drug design"), these are future possibilities rather than the current focus.	no	The study uses synthetic data, but it's not clear if it's tabular data. The abstract mentions "in silico tests" and "microfluidic devices" data, which likely involves time-series or other non-tabular data formats.	no	The study uses in vitro cell cultures and microfluidic devices, not human clinical data or patient information.	yes	Yes, this appears to be an original research article with novel methodology and results.	maybe	Yes, the study involves active creation of synthetic data using AI (PGNNIV), though it's based on a "previously validated model."	maybe	While the study does generate synthetic data using AI (PGNNIV), it's not clear if this is specifically tabular data generation.	no	While the paper mentions potential clinical applications, it doesn't demonstrate direct clinical applications. The focus is on understanding tumor evolution in controlled laboratory conditions.	no	The study includes empirical evaluation, but it's focused on technical performance rather than clinical utility.	no	The abstract doesn't clearly specify quantifiable clinical outcomes. It mentions "unravelling capacity" and "predictive power" but doesn't provide specific metrics.	Exclude	The study focuses on technical aspects of tumor evolution using microfluidic devices and AI, primarily involving in vitro cell cultures rather than human clinical data. While demonstrating innovative methodology for understanding Glioblastoma invasion, the research lacks direct clinical applications, quantifiable clinical outcomes, and tabular data generation. The work represents a technical exploration with potential future clinical implications, but does not meet the specified screening criteria for clinical research.	1.4	Exclude	Exclude	No tabular data
Unsupervised Cross-Modality Domain Adaptation Network for X-Ray to CT Registration	Zheng S., Yang X., Wang Y., Ding M., Hou W.	2022	maybe	The paper focuses on 2D/3D registration for radiotherapy and image guided surgeries, which is a clinical application. However, the primary focus appears to be on the technical aspects of the registration process rather than direct clinical outcomes.	no	The study deals with imaging data (2D x-rays and 3D CT/CBCT) rather than tabular data, which clearly fails to meet the criterion for tabular data generation.	yes	The study uses clinical imaging data (CT and CBCT), which implies human clinical data.	yes	This appears to be an original research article presenting a new method (UCMDAN).	maybe	While the study involves synthetic data, it's not clear if it actively creates new synthetic data or uses existing synthetic datasets. The abstract mentions "synthetic data from pre-operative volume" but doesn't specify if this is newly generated.	no	The study does involve synthetic data, but it's used for domain adaptation rather than generating new tabular data. The focus is on image registration and domain adaptation.	maybe	While the application is in radiotherapy and surgery, the paper focuses more on the technical aspects of registration rather than direct clinical applications.	yes	The study includes empirical evaluation through experiments on CT and CBCT datasets.	yes	The abstract mentions "extensive experiments" and comparison with state-of-the-art approaches, suggesting quantifiable outcomes, though specific metrics aren't detailed.	Exclude	The study focuses on 2D/3D image registration for radiotherapy and surgery, utilizing imaging data rather than tabular data, which fundamentally disqualifies it from meeting key screening criteria. While the research demonstrates technical innovation in domain adaptation and involves clinical imaging, it fails to align with the specific requirements for synthetic tabular data generation and clinical application focus. The paper's technical orientation and imaging-based approach render it unsuitable for the intended screening framework.	1.4	Exclude	Exclude	No tabular data
Unsupervised detection and analysis of changes in everyday physical activity data	Sprint G., Cook D.J., Schmitter-Edgecombe M.	2016	maybe	The paper focuses on clinical/health applications (physical activity monitoring and health interventions) rather than purely technical aspects. However, it's more focused on the technical methodology (PACD framework) than clinical outcomes.	no	The study deals with time-series data from wearable sensors, which explicitly violates this criterion as it's not tabular data.	yes	Yes, the study uses real data from older adults in a health intervention study.	yes	Yes, this appears to be an original research article with novel methodology and empirical evaluation.	no	While the paper mentions using synthetic data, it appears to be using it for evaluation rather than creating synthetic data as its primary focus. The synthetic data creation method is not specified.	no	The abstract doesn't specify the method used for synthetic data generation, and it's not the primary focus of the study.	maybe	While the study has health-related applications, it's more focused on activity monitoring and change detection rather than direct clinical applications like diagnosis or treatment planning.	yes	Yes, the study includes empirical evaluation using both synthetic and real data.	maybe	The study reports on change detection capabilities, but the specific metrics aren't clearly detailed in the abstract.	Exclude	The study focuses on physical activity change detection using wearable sensor data, but critically fails to meet key screening criteria, particularly the requirement for tabular data generation. While demonstrating empirical evaluation and using human clinical data, the time-series data format and technical methodology orientation disqualify it from meeting the specified screening requirements.	1.4	Exclude	Exclude	No tabular data
Unsupervised detection and removal of muscle artifacts from scalp EEG recordings using canonical correlation analysis, wavelets and random forests	Anastasiadou M.N., Christodoulakis M., Papathanasiou E.S., Papacostas S.S., Mitsis G.D.	2017	no	The paper focuses on technical aspects (artifact detection and removal from EEG recordings) rather than clinical applications. While it mentions potential clinical use, the primary focus is methodological.	no	The study deals with EEG recordings, which are time-series data, not tabular data.	yes	The study uses human clinical data (EEG recordings from ten patients with epilepsy).	yes	This appears to be an original research article with novel algorithms and empirical evaluation.	no	While the paper mentions "synthetic data constructed from the original observations," this is used for feature selection, not as the primary focus of the study.	no	The paper does not focus on synthetic data generation using AI methods. The synthetic data mentioned is used as a tool for feature selection.	no	While the paper mentions potential clinical applications, it focuses on signal processing rather than direct clinical applications like diagnosis or treatment planning.	yes	The study includes original research with clear empirical evaluation using both simulation and experimental data.	yes	The study reports quantifiable outcomes in terms of classification performance and goodness-of-fit values.	Exclude	The study primarily focuses on technical signal processing methods for EEG artifact detection, using time-series data rather than tabular data, and does not center on synthetic data generation or direct clinical applications. While the research involves human clinical data and includes empirical evaluation with quantifiable outcomes, it fails to meet key screening criteria related to synthetic data generation and clinical application focus. The methodological approach and technical nature of the research diverge from the systematic review's core requirements.	1.4	Exclude	Exclude	No tabular data
Unsupervised Reverse Domain Adaptation for Synthetic Medical Images via Adversarial Training	Mahmood F., Chen R., Durr N.J.	2018	maybe	The paper focuses on clinical applications (depth estimation in endoscopy for colonoscopy and surgery), but also has significant technical components. However, the clinical application is central to the work.	no	The study focuses on medical imaging data, not tabular data. This is a clear violation of the inclusion criteria.	maybe	The study uses pig colon data rather than human clinical data, though it's intended for human clinical applications.	yes	This appears to be an original research article with novel methodology and empirical results.	yes	The study does involve active creation of synthetic data using AI methods, specifically for medical imaging.	maybe	While the study does generate synthetic data using AI methods, it's for imaging rather than tabular data.	yes	The study demonstrates direct clinical applications in endoscopy and surgery.	yes	The study includes original research with clear empirical evaluation and metrics.	yes	The study reports quantifiable outcomes (78.7% improvement in structural similarity).	Exclude	The study focuses on medical imaging and synthetic data generation for endoscopic depth estimation, but critically fails to meet the requirement of using tabular data. While demonstrating strong clinical applications and empirical evaluation, the use of imaging data and pig colon models prevents full alignment with the specified screening criteria. The research presents innovative AI-driven synthetic data techniques but does not satisfy the fundamental data format requirement.	1.4	Exclude	Exclude	No tabular data
Unsupervised Segmentation of 3D Microvascular Photoacoustic Images Using Deep Generative Learning	Sweeney P.W., Hacker L., Lefebvre T.L., Brown E.L., Grohl J., Bohndiek S.E.	2024	maybe	The paper focuses on clinical applications (vascular network segmentation) rather than purely technical aspects, but it is primarily a technical methodology paper with clinical applications as a secondary focus.	no	The study deals with 3D photoacoustic imaging data, not tabular data, which is a clear violation of the criterion.	yes	The study includes both in vitro and in vivo data, including patient-derived breast cancer xenograft models and clinical angiograms.	yes	This appears to be an original research article with novel methodology and results.	maybe	The study does involve active creation of synthetic data using AI (VAN-GAN), but this is for training purposes rather than the primary focus.	no	While the study uses AI (GAN) for synthetic data generation, it's for image data, not tabular data.	yes	The study demonstrates clinical applications in vascular network segmentation, which has direct clinical relevance.	yes	The study includes original research with clear empirical evaluation and metrics.	yes	The study reports quantifiable outcomes (F1 scores comparing VAN-GAN vs. U-Net).	Exclude	The study involves photoacoustic imaging and synthetic data generation using AI, but fundamentally focuses on imaging data rather than tabular data, which disqualifies it from meeting key screening criteria. While demonstrating clinical applications and quantifiable outcomes, the research's primary methodology and data format do not align with the specified requirements for inclusion.	1.4	Exclude	Exclude	No tabular data
Unsupervised translation of vascular masks to NIR-II fluorescence images using Attention-Guided generative adversarial networks.	Fang, Lu, Sheng, Huaixuan, Li, Huizhu, Li, Shunyao, Feng, Sijia, Chen, Mo, Li, Yunxia, Chen, Jun, Chen, Fuchun	2025	no	The paper focuses on technical aspects of image generation rather than direct clinical applications. While it mentions "medical imaging," the primary focus is on the technical methodology of generating synthetic images.	no	The study deals with imaging data (NIR-II fluorescence images), not tabular data.	maybe	The abstract doesn't explicitly state whether the vascular masks are from human subjects. While it's likely related to human clinical data, this isn't clearly specified.	yes	This appears to be an original research article presenting a new GAN approach.	yes	The study does involve active creation of synthetic data using AI (specifically GANs).	maybe	While the study does use AI methods (GANs) for synthetic data generation, it's for images rather than tabular data.	no	The paper focuses on image generation methodology rather than direct clinical applications like diagnosis or treatment planning.	maybe	The study includes empirical evaluation, but focuses on technical performance rather than clinical utility.	no	The study reports "quantitative metrics" for image quality, but these are technical metrics rather than clinical outcomes.	Exclude	The study focuses on generating synthetic near-infrared fluorescence images using AI techniques, which fundamentally diverges from the required tabular data generation and clinical application criteria. Despite involving AI-driven data synthesis, the research is primarily a technical methodology paper centered on image generation rather than direct clinical utility or patient-related healthcare outcomes. Consequently, the work fails to meet multiple critical screening requirements for clinical data generation and application.	1.4	Exclude	Exclude	No tabular data
Using deep clustering to improve fMRI dynamic functional connectivity analysis	Spencer A.P.C., Goodfellow M.	2022	no	The paper focuses on technical aspects of fMRI data analysis and clustering methods rather than direct clinical applications. While it mentions clinical populations, the primary focus is on methodological improvements to clustering techniques.	no	The study deals with fMRI data and dynamic functional connectivity analysis, which is not tabular data but rather imaging/time-series data.	yes	The study does use human clinical data (fMRI data from human subjects) as part of its evaluation.	yes	This appears to be an original research article with novel methodology and empirical evaluation.	no	While the study uses synthetic data, it appears to be using existing synthetic datasets for evaluation rather than actively creating new synthetic data using AI.	no	The study uses synthetic data but doesn't appear to generate it using AI methods. Instead, it uses synthetic datasets to evaluate different clustering approaches.	no	While the study mentions clinical populations, it doesn't demonstrate direct clinical applications like diagnosis or treatment planning. It focuses on improving clustering methodology.	yes	The study includes original research with empirical evaluation and clear metrics for assessing performance.	yes	The study reports quantifiable outcomes related to clustering performance and state temporal properties.	Exclude	The study primarily focuses on technical methodological improvements in fMRI data clustering, using imaging and time-series data rather than tabular clinical data. While demonstrating empirical research with human subjects and quantifiable outcomes, the work lacks direct clinical applications and does not involve AI-driven synthetic data generation. Consequently, the research does not align with the specified screening criteria for clinical AI data generation.	1.4	Exclude	Exclude	No tabular data

Using diffusion models to generate synthetic labeled data for medical image segmentation	Saragih D.G., Hibi A., Tyrrell P.N.	2024	no	The study focuses on technical aspects of image generation and segmentation model performance, rather than direct clinical applications.	no	The study deals with medical images (polyp images) rather than tabular data generation.	yes	The study uses human clinical data (polyp images from the GI tract).	yes	This appears to be an original research article with clear methodology and results.	yes	The study actively creates synthetic data using diffusion models.	maybe	While the study does use AI methods (diffusion models) for synthetic data generation, it's for images rather than tabular data.	no	The study focuses on improving segmentation model performance rather than direct clinical applications like diagnosis or treatment planning.	yes	The study includes original research with clear empirical evaluation and metrics.	yes	The study reports clear metrics (FID, MS-SSIM, Dice score, IoU).	Exclude	The study focuses on generating synthetic medical images using diffusion models, which fundamentally deviates from the required tabular data generation criteria. While demonstrating technical sophistication in image synthesis and segmentation, the research fails to meet key screening requirements around clinical application and data format. The methodology, though rigorous, does not align with the specified screening parameters for clinical data generation.	1.4	Exclude	Exclude	No tabular data
Using publicly available data to predict recreational cannabis legalization at the county-level: A machine learning approach.	Montgomery, Barrett Wallace, Tong, Xiaoran, Vsevolozhskaya, Olga, Anthony, James C	2024	no	The paper focuses on policy prediction rather than clinical applications. While it uses health-related data (substance use and mental illness), the primary application is policy analysis, not clinical care.	maybe	The study uses tabular data from census, election data, and health surveys. While it meets the tabular data requirement, the focus is on policy prediction rather than clinical data.	no	While the study uses some health-related data (substance use and mental illness), this is population-level data for policy analysis rather than individual clinical data.	yes	This is an original research article with clear methodology and results.	no	The study uses "synthetic data augmentation" but this appears to be for oversampling minority classes rather than creating new synthetic data for clinical purposes.	no	While synthetic data is mentioned, it's used for data augmentation in machine learning rather than generating synthetic clinical data.	no	The study focuses on policy prediction rather than clinical applications like diagnosis, treatment, or patient care.	no	The study includes empirical evaluation with clear metrics, but for policy prediction rather than clinical utility.	no	The study reports quantifiable outcomes (macro f1 score of 0.61), but these are for policy prediction accuracy, not clinical outcomes.	Exclude	The study primarily focuses on policy prediction using machine learning techniques, which diverges significantly from clinical research requirements. Despite using tabular data and synthetic data augmentation, the research lacks direct clinical applications, patient-centered outcomes, and healthcare-specific utility. Consequently, the paper does not align with the specified screening criteria for clinical synthetic data research.	1.4	Exclude	Exclude	No synthetic data generation
Using Synthetic Training Data for Deep Learning-Based GBM Segmentation.	Lindner, Lydia, Narnhofer, Dominik, Weber, Maximilian, Gsaxner, Christina, Kolodziej, Malgorzata, Egger, Jan	2019	yes	The study focuses on clinical applications (GBM segmentation) rather than purely technical aspects, even though it involves technical elements. The primary focus is on clinical utility.	maybe	The study deals with medical imaging data (MRI), not tabular data, which is a clear violation of the core criterion.	yes	The study uses human clinical data (MRI images of GBMs) as its basis.	yes	This appears to be an original research article with novel methodology and results.	yes	The study involves active creation of synthetic data using AI methods, specifically for training a U-Net-based segmentation network.	yes	The study explicitly mentions using "a semi-automatic method for generating synthetic GBM data," though the specific AI method (GAN, VAE, etc.) isn't detailed.	yes	The study demonstrates direct clinical application in tumor segmentation, which is crucial for diagnosis and treatment planning.	yes	The study includes empirical evaluation using real MRI images of GBMs.	yes	The abstract mentions evaluation of "segmentation performance," implying quantifiable metrics, though specific metrics aren't detailed.	Exclude	The study focuses on synthetic data generation for glioblastoma segmentation using AI, demonstrating strong clinical application and empirical evaluation. However, the research fundamentally fails to meet the screening criteria due to its use of medical imaging data instead of tabular data, which represents a critical disqualifying factor. Despite meeting multiple other criteria, the data format requirement renders the study non-compliant with the specified screening parameters.	1.4	Exclude	Exclude	No tabular data
Utility of deep learning networks for the generation of artificial cardiac magnetic resonance images in congenital heart disease.	Diller, Gerhard-Paul, Vahle, Julius, Radke, Robert, Vidal, Maria Luisa Benesch, Fischer, Alicia Jeanette, Bauer, Ulrike M M, Sarikouch, Samir, Berger, Felix, Beerbaum, Philipp, Baumgartner, Helmut, Orwat, Stefan	2020	no	The study focuses primarily on technical aspects (image generation and segmentation) rather than clinical applications. While it's in a medical context, the primary focus is on the technical methodology.	no	The study deals with medical imaging data (MRI frames), not tabular data. This is explicitly stated in the abstract and is a clear violation of this criterion.	yes	The study uses real cardiac MRI data from 303 patients with Tetralogy of Fallot.	yes	This appears to be an original research article with clear methodology and results.	yes	The study actively creates synthetic data using PG-GANs, which is clearly described in the methods section.	maybe	While the study does use AI (PG-GANs) for synthetic data generation, it's generating images rather than tabular data.	no	The study doesn't demonstrate direct clinical applications. It focuses on technical performance of segmentation algorithms rather than clinical decision-making or patient outcomes.	yes	The study includes clear empirical evaluation comparing synthetic vs. real data performance.	yes	The study reports quantifiable outcomes, specifically segmentation performance metrics.	Exclude	The study focuses on generating synthetic cardiac MRI images using progressive generative adversarial networks, which fundamentally deviates from the required tabular data generation criteria. Despite demonstrating technical sophistication in image synthesis and segmentation, the research fails to meet key screening requirements related to data format and clinical application focus. Consequently, the paper does not align with the systematic review's core methodological constraints.	1.4	Exclude	Exclude	No tabular data
Venous thromboembolism in the era of machine learning and artificial intelligence in medicine	Reyes Gil M., Pantanowitz J., Rashidi H.H.	2024	maybe	The paper appears to be a review of AI applications in VTE management, including clinical aspects. However, it seems to cover both technical and clinical aspects broadly.	no	The abstract doesn't specifically discuss tabular data generation. While it mentions synthetic data, it doesn't specify the format.	maybe	The paper discusses VTE in human clinical contexts, but doesn't appear to directly work with clinical data.	no	The abstract explicitly states this is a "review" paper, not original research.	no	The paper discusses synthetic data but appears to be reviewing existing approaches rather than creating new synthetic data.	maybe	While synthetic data is mentioned, it's discussed as a general concept rather than presenting specific AI methods for generating synthetic tabular data.	maybe	The paper discusses clinical applications of AI in VTE management, but as a review rather than demonstrating specific applications.	no	As a review paper, it doesn't present original empirical evaluation.	no	The abstract doesn't mention specific quantifiable outcomes or metrics.	Exclude	A comprehensive review exploring AI's potential in venous thromboembolism management fails to meet key screening criteria, particularly lacking original research, empirical evaluation, and quantifiable outcomes. While discussing synthetic data and clinical applications, the paper does not demonstrate direct data generation or provide specific metrics for assessing AI's clinical utility. The review's broad perspective and theoretical approach prevent it from satisfying the required methodological and research standards.	1.4	Exclude	Exclude	Wrong publication type
Where, why, and how is bias learned in medical image analysis models? A study of bias encoding within convolutional networks using synthetic data.	Stanley, Emma A M, Souza, Raissa, Wilms, Matthias, Forkert, Nils D	2025	maybe	The paper focuses on understanding algorithmic bias in medical imaging models, which is more methodological/technical than clinical. While it has clinical implications, it's primarily a technical study of bias mechanisms.	no	The study uses synthetic brain MRI data, which is imaging data, not tabular data.	no	The study uses synthetic brain MRI data, not real human clinical data.	yes	This appears to be an original research article with clear methodology and findings.	maybe	The study does create synthetic data, but it's not clear if AI methods are used for this creation. The focus is on using the synthetic data to study bias, not on the generation process itself.	no	While synthetic data is used, it's MRI imaging data, not tabular data. The generation method isn't specified in the abstract.	maybe	While the study has implications for clinical applications, it doesn't directly demonstrate clinical applications like diagnosis or treatment planning.	yes	The study includes original research with empirical evaluation and clear metrics.	yes	The study reports quantifiable outcomes related to bias encoding and shortcut learning.	Exclude	The study focuses on algorithmic bias in medical imaging using synthetic MRI data, which fundamentally differs from the required tabular data format and human clinical data specifications. While demonstrating original research with quantifiable outcomes, the paper fails to meet critical screening criteria related to data type, clinical application, and empirical evaluation of synthetic data generation. Consequently, the research does not align with the predefined screening requirements.	1.4	Exclude	Exclude	No tabular data
Advancing AI Data Ethics in Nursing: Future Directions for Nursing Practice, Research, and Education.	Ball Dunlap, Patricia A, Michalowski, Martin	2024	no	The paper appears to be primarily focused on ethical considerations and frameworks rather than clinical applications. While it mentions healthcare and nursing, it's from an ethical and educational perspective rather than a clinical application perspective.	maybe	The abstract doesn't specifically discuss tabular data generation. It mentions "real-world and synthetic data" broadly but doesn't specify formats.	no	While healthcare is mentioned, the paper doesn't appear to work with actual clinical data. It's focused on ethical frameworks and principles.	no	This appears to be a viewpoint/commentary paper that includes a literature review, not an original research article.	no	The paper doesn't appear to involve active creation of synthetic data. It discusses data ethics and principles but doesn't describe generating synthetic data.	no	While synthetic data is mentioned, there's no indication of AI methods being used for generation. The focus is on ethical considerations rather than generation techniques.	no	The paper doesn't demonstrate direct clinical applications. It focuses on ethical considerations and recommendations for nursing practice.	no	The paper doesn't include empirical evaluation. It presents ethical principles and recommendations based on a literature review.	no	No quantifiable outcomes are mentioned. The paper appears to be theoretical/ethical in nature rather than presenting measurable results.	Exclude	The abstract describes an ethical perspective on AI and data in nursing, focusing on principles and recommendations rather than empirical research or synthetic data generation. It fails to meet multiple screening criteria, including original data creation, clinical application, and quantifiable outcomes, primarily presenting a theoretical viewpoint on data ethics. The paper does not demonstrate direct clinical utility or involve active synthetic data generation using AI methods.	0.1	Exclude	Exclude	Wrong publication type
AI in Neurology: Everything, Everywhere, All at Once Part 3: Surveillance, Synthesis, Simulation, and Systems	Rizzo M.	2025	no	The paper appears to be a review focusing on broad applications of AI in healthcare, including data management, education, and care. While it mentions clinical applications, it's not focused on specific clinical applications but rather provides a broad overview.	maybe	The abstract mentions "real-world and synthetic data" but doesn't specify the format. There's no clear indication that it focuses exclusively on tabular data.	maybe	The paper discusses healthcare networks and clinical trials, suggesting human clinical data is involved, but this is in the context of a review rather than original research.	no	The abstract explicitly states this is a "review" (part 3 of a series), which fails this criterion.	no	The paper appears to be a review rather than original research creating synthetic data. It discusses AI's role in synthetic data but doesn't indicate active creation of new synthetic data.	no	While synthetic data is mentioned, the paper doesn't appear to focus on the generation methods themselves, and there's no mention of specific AI methods like GANs or VAEs.	no	While clinical applications are mentioned, they are discussed at a high level in a review context rather than demonstrating specific direct clinical applications.	no	As a review paper, it doesn't include original empirical evaluation.	no	The abstract doesn't mention any specific quantifiable outcomes or metrics.	Exclude	This review paper fails multiple screening criteria by not presenting original research, lacking empirical evaluation, and not demonstrating direct clinical applications with quantifiable outcomes. While discussing AI in healthcare broadly, the abstract does not meet the specific requirements for synthetic data generation, clinical focus, or original research methodology. The high-level review nature of the paper fundamentally disqualifies it from the systematic review's targeted research parameters.	0.1	Exclude	Exclude	Wrong publication type
All Together Now: Data Work to Advance Privacy, Science, and Health in the Age of Synthetic Data.	Fernandez-Rhodes, Lindsay, Wagner, Jennifer K	2025	no	The paper appears to be primarily focused on ethical, legal, and social implications (ELSI) of synthetic data in biomedicine, rather than clinical applications. It discusses data practices, transparency, and regulatory challenges.	maybe	The abstract does not specifically discuss tabular data generation. It mentions synthetic data and digital twins in general terms but doesn't specify the data format.	maybe	While the paper discusses biomedical data, it doesn't specifically focus on human clinical data or patient-related healthcare information. It's more about general data practices.	no	This appears to be a workshop description or position paper rather than an original research article.	no	The paper does not describe active creation of synthetic data. Instead, it discusses the broader implications and challenges of synthetic data use.	no	While synthetic data is mentioned, the paper does not focus on the generation methods or techniques. It's more about the implications and challenges of using synthetic data.	no	The paper does not demonstrate direct clinical applications. It focuses on ethical, legal, and social implications rather than clinical use cases.	no	The abstract does not indicate any empirical evaluation or clear metrics. It appears to be a discussion of challenges and implications rather than an empirical study.	no	No quantifiable outcomes are mentioned in the abstract. The paper appears to be qualitative in nature, discussing challenges and implications.	Exclude	The abstract describes a workshop on ethical, legal, and social implications of synthetic data in biomedicine, focusing on challenges and implications rather than clinical applications or data generation techniques. It fails to meet multiple screening criteria, including original research, empirical evaluation, quantifiable outcomes, and direct clinical applications. The paper appears to be a qualitative discussion of data practices and challenges rather than a research study on synthetic data generation.	0.1	Exclude	Exclude	Wrong publication type
AML diagnostics in the 21st century: Use of AI	Haferlach T., Eckardt J.-N., Walter W., Maschek S., Kather J.N., Pohlkamp C., Middeke J.M.	2025	maybe	The paper discusses clinical applications in AML diagnostics, but this is a review paper rather than a study of specific applications. While it mentions clinical applications, it's primarily focused on reviewing the landscape.	no	The abstract doesn't specifically discuss tabular data generation. It mentions various diagnostic methods and data types but doesn't focus on synthetic tabular data generation.	maybe	The paper discusses human clinical data in the context of AML diagnostics, but doesn't appear to directly work with this data.	no	This is explicitly a review paper ("This review delves into…"), which fails this criterion as we're looking for original research articles.	no	While synthetic data is mentioned, the paper doesn't appear to involve active creation of synthetic data. It only discusses synthetic data as a topic within the broader review.	no	The paper mentions synthetic data but doesn't describe implementing or testing any specific synthetic data generation methods. It's discussed as a general concept rather than a specific implementation.	maybe	While clinical applications are discussed, this is in a review context rather than demonstrating specific applications.	no	As a review paper, it doesn't include original empirical evaluation.	no	The abstract doesn't mention any specific quantifiable outcomes or metrics.	Exclude	This review paper on AI in acute myeloid leukemia diagnostics fails to meet multiple screening criteria, particularly the requirement for original research with empirical evaluation. While discussing clinical applications and synthetic data, the abstract lacks specific implementation of synthetic data generation, quantifiable outcomes, and direct clinical utility testing. The review's broad landscape perspective and lack of original research disqualify it from meeting the systematic review's stringent screening requirements.	0.1	Exclude	Exclude	Wrong publication type
Artificial intelligence for glaucoma: state of the art and future perspectives	Barao R.C., Hemelings R., Pinto L.A., Pazos M., Stalmans I.	2025	maybe	The paper appears to be a review of AI applications in glaucoma diagnosis and management, which is clinical in nature. However, it's not clear if it focuses on specific clinical applications or is more of a general overview.	no	The abstract mentions color fundus photography and optical coherence tomography, which are imaging data formats, not tabular data.	yes	The paper discusses glaucoma diagnosis and management in a clinical context, suggesting human clinical data is involved.	no	The abstract explicitly states this is a "review" paper.	no	While the abstract mentions "synthetic data," it appears to be discussing this as part of a broader review rather than actively creating synthetic data.	no	The paper mentions synthetic data and deep learning, but it's unclear if it involves actual generation of synthetic tabular data. The focus appears to be on imaging data.	yes	The paper discusses clinical applications in glaucoma diagnosis and management.	no	As a review paper, it likely doesn't include original empirical evaluation.	maybe	While the paper mentions "diagnostic capabilities," it's not clear if specific quantifiable outcomes are reported.	Exclude	This review paper on AI in glaucoma fails key screening criteria by focusing on imaging data rather than tabular data and not presenting original research with synthetic data generation. Multiple critical requirements are not met, including the need for an original research article with empirical evaluation of synthetic tabular data, rendering the paper ineligible for inclusion in the systematic review.	0.1	Exclude	Exclude	Wrong publication type

CMMIQA: a prompt-driven cross-modality multi-organ medical image quality assessment model	Xun S., Sun Y., Wang Y., Wang M., Wu M., Liu X., Tan T.	2025	maybe	The paper focuses on medical image quality assessment, which is a technical aspect rather than a direct clinical application. While it has clinical relevance, it's primarily focused on the technical methodology of image quality assessment.	no	The study deals with 3D medical imaging data (CT and MRI), not tabular data.	yes	Yes, the study uses real medical imaging data from human patients.	yes	Yes, this appears to be an original research article with clear methodology and results.	no	The paper explicitly states they used "real rather than synthetic data" to create their database.	no	The study does not involve synthetic data generation. It focuses on quality assessment of real medical images.	maybe	While the study has clinical relevance in terms of image quality assessment, it doesn't demonstrate direct clinical applications like diagnosis or treatment planning. It's more focused on the technical assessment of image quality.	yes	Yes, the study includes empirical evaluation with clear metrics.	yes	Yes, the study reports quantifiable outcomes (SRCC, PLCC, and RMSE metrics).	Exclude	The study focuses on medical image quality assessment using real imaging data, which fundamentally diverges from the screening criteria requiring synthetic tabular data generation. Critical requirements around synthetic data creation, tabular data format, and direct clinical applications are not met, rendering the paper unsuitable for the systematic review. The research primarily addresses technical image quality assessment methodology rather than the specified research parameters.	0.1	Exclude	Exclude	No tabular data
Data Augmentation for Deep-Learning-Based Multiclass Structural Damage Detection Using Limited Information	Dunphy K., Fekri M.N., Grolinger K., Sadhu A.	2022	no	The study focuses on structural health monitoring of infrastructure, not clinical applications, which is a critical mismatch with the screening criteria.	no	The study deals with image data of concrete surfaces, not tabular data, which is explicitly stated in the abstract and is another critical mismatch.	no	The study uses infrastructure/concrete surface data, not human clinical or patient-related healthcare information.	yes	This appears to be an original research article with novel findings and empirical evaluation.	yes	The study actively creates synthetic data using GANs, which is clearly described in the abstract.	yes	The study uses GANs for synthetic data generation, which meets this criterion.	no	The application is for structural health monitoring, not clinical applications like diagnosis, prognosis, or treatment planning.	yes	The study includes original research with clear empirical evaluation and metrics for assessing the synthetic data's utility.	yes	The study reports quantifiable outcomes including classification performance metrics and accuracy measurements.	Exclude	The study focuses on structural health monitoring using image data and GANs, which fundamentally diverges from clinical research requirements involving human healthcare data. Critical screening criteria related to clinical applications, tabular data format, and patient-related information are not met, rendering the research non-compliant with the specified screening parameters. Despite demonstrating strong methodological rigor in synthetic data generation, the core focus and data type disqualify the study from meeting the established screening criteria.	0.1	Exclude	Exclude	No tabular data
GAN-based synthetic medical image augmentation for increased CNN performance in liver lesion classification	Frid-Adar M., Diamant I., Klang E., Amitai M., Goldberger J., Greenspan H.	2018	yes	The study focuses on clinical applications, specifically medical image classification for liver lesion diagnosis, which is a clinical application rather than purely technical.	maybe	The study deals with medical imaging (CT images) and image generation, not tabular data, which is a critical failure of this core criterion.	yes	The study uses real CT images of human liver lesions as its basis.	yes	This appears to be an original research article with novel methodology and results.	yes	The study actively creates synthetic data using GANs, not using pre-existing synthetic datasets.	maybe	While the study does use AI (GANs) for synthetic data generation, it generates images rather than tabular data.	yes	The study demonstrates direct clinical application in diagnosis of liver lesions.	yes	The study includes original research with clear empirical evaluation and metrics.	yes	The study reports clear quantifiable outcomes (sensitivity and specificity metrics).	Exclude	The study focuses on medical image generation using GANs for liver lesion classification, demonstrating strong clinical application and empirical evaluation. However, the research fundamentally fails key screening criteria by generating synthetic images rather than tabular data, which disqualifies it from the systematic review. Despite meeting most other criteria with high-quality methodology and quantifiable outcomes, the imaging format represents a critical exclusion factor.	0.1	Exclude	Exclude	No tabular data
Generation of synthetic EEG data for training algorithms supporting the diagnosis of major depressive disorder	Carrle F.P., Hollenbenders Y., Reichenbach A.	2023	maybe	The paper focuses on clinical applications (MDD diagnosis) rather than purely technical aspects, though it has a strong methodological component.	no	The study focuses on EEG time-series data, which is explicitly excluded in the criteria as "time-series data like EEGs and ECGs."	yes	The study uses human clinical data from MDD patients and healthy controls.	yes	This is an original research article combining a systematic review with empirical evaluation.	yes	The study actively creates synthetic data using GANs with CNNs.	yes	The study uses AI methods (GANs) to generate synthetic data.	yes	The study demonstrates direct clinical application in MDD diagnosis.	yes	The study includes original empirical evaluation with clear metrics for assessing synthetic data's utility.	yes	The study reports quantifiable outcomes in terms of diagnostic accuracy improvements (up to 10%).	Exclude	The study focuses on generating synthetic EEG data for major depressive disorder diagnosis using generative adversarial networks, demonstrating strong clinical application and empirical evaluation. However, the research fails a critical screening criterion by utilizing time-series EEG data, which is explicitly excluded from the specified data format requirements. Despite meeting most other criteria with robust methodology and quantifiable outcomes, this single disqualifying factor prevents the study from passing the screening process.	0.1	Exclude	Exclude	No tabular data
Inverse consistent non-rigid image registration based on robust point set matching.	Yang, Xuan, Pei, Jihong, Shi, Jingli	2014	no	The paper focuses on technical aspects of image registration algorithms rather than clinical applications. While it mentions medical images, the focus is on the algorithmic improvement of point matching techniques.	no	The study deals with image data and point sets, not tabular data generation.	no	While medical images are mentioned, the focus is on the technical aspects of image registration rather than using human clinical data as the basis for analysis.	yes	This appears to be an original research article with methods, results, and conclusions.	no	The study does not involve creating synthetic data using AI. While it mentions "synthetic data sets," these appear to be used for validation rather than being the focus of AI generation.	no	The paper does not involve generating synthetic tabular data using AI methods. It focuses on image registration techniques.	no	The paper does not demonstrate direct clinical applications. It focuses on improving image registration algorithms without specific clinical use cases.	no	The study includes empirical evaluation, but not of clinical utility. The evaluation focuses on technical performance metrics.	no	The study reports quantifiable outcomes, but these are related to algorithmic performance (inverse consistent errors, registration errors) rather than clinical metrics.	Exclude	The study focuses on technical image registration algorithms using point matching techniques, primarily addressing computational methodology rather than clinical applications. Despite involving medical images, the research does not generate synthetic tabular data, demonstrate direct clinical utility, or report clinically relevant outcomes. The paper's technical nature and lack of clinical focus result in failure to meet most of the specified screening criteria.	0.1	Exclude	Exclude	No tabular data
Large Language Models in an App: Conducting a Qualitative Synthetic Data Analysis of How Snapchat's "My AI" Responds to Questions About Sexual Consent, Sexual Refusals, Sexual Assault, and Sexting	Marcantonio T.L., Avery G., Thrash A., Leone R.M.	2024	no	The study focuses on educational/communication aspects rather than clinical applications. While it deals with sexual health, it's examining AI's role in education and information provision, not clinical decision-making or patient care.	no	The study does not involve tabular data generation. It focuses on AI-generated text responses to questions.	no	The study does not use clinical data or patient-related healthcare information. It uses AI responses to educational questions.	yes	This appears to be an original research article with empirical evaluation of AI responses.	no	While the study uses AI (My AI), it's not creating synthetic data but rather analyzing existing AI responses to questions.	no	The study does not involve generating synthetic tabular data. It's analyzing AI-generated text responses.	no	The study focuses on educational applications rather than direct clinical applications like diagnosis or treatment.	no	The study does include empirical evaluation, but not of synthetic data's clinical utility.	no	While the study includes analysis, it appears to be primarily qualitative content analysis rather than quantifiable clinical metrics.	Exclude	The study focuses on AI's educational responses to sexual health questions, which fundamentally diverges from the specified screening criteria related to clinical applications, synthetic data generation, and quantifiable clinical outcomes. Despite being an original research article, the work fails to meet key requirements around clinical data, tabular data generation, and direct clinical utility. The research is primarily an educational communication analysis rather than a clinical or synthetic data investigation.	0.1	Exclude	Exclude	No tabular data
Leveraging domain knowledge for synthetic ultrasound image generation: a novel approach to rare disease AI detection.	Mendez, M, Castillo, F, Probyn, L, Kras, S, Tyrrell, P N	2025	yes	The study focuses on clinical applications (hemarthrosis detection in hemophilia patients) rather than purely technical aspects, though there is significant technical focus on the generation methodology.	maybe	The study focuses on ultrasound images, not tabular data, which is a clear violation of the core criterion requiring tabular data only.	yes	The study uses human clinical data from both hemophiliac and non-hemophiliac patients.	yes	This appears to be an original research article with clear methodology and results sections.	yes	The study actively creates synthetic data using deep generative models, specifically mentioning content and context generators.	yes	The study uses AI methods (deep generative models) for synthetic data generation, though for images rather than tabular data.	yes	The study demonstrates direct clinical application in diagnosis (detection of hemarthrosis).	yes	The study includes original research with clear empirical evaluation and metrics.	yes	The study reports quantifiable outcomes through expert evaluations, statistical analysis, and specific metrics (domain-invariant perceptual distance and Frechet inception distance).	Exclude	The study demonstrates strong clinical application and original research in synthetic data generation for hemarthrosis detection, utilizing deep generative models with human clinical data. However, the research focuses on ultrasound image generation rather than tabular data, which critically disqualifies it from meeting the specified screening criteria. Despite robust methodology and quantifiable outcomes, the non-tabular data format represents a fundamental exclusion from the systematic review's scope.	0.1	Exclude	Exclude	No tabular data
mHealth Systems Need a Privacy-by-Design Approach: Commentary on "Federated Machine Learning, Privacy-Enhancing Technologies, and Data Protection Laws in Medical Research: Scoping Review".	Tewari, Ambuj	2023	no	The paper appears to be primarily focused on technical and legal aspects of privacy in mHealth systems, rather than clinical applications. It discusses privacy regulations and privacy-enhancing technologies.	no	The abstract does not specifically mention tabular data generation. It only briefly mentions "private synthetic data generation" without specifying the format.	no	The abstract doesn't indicate any direct use of human clinical data.	no	This appears to be a commentary or letter responding to a scoping review paper, not an original research article.	no	While synthetic data generation is mentioned, it's only referenced as an emerging technology and not as something the paper actively implements or studies.	no	The paper only mentions synthetic data generation as a future consideration, not as a method being implemented or studied.	no	No direct clinical applications are demonstrated in the abstract.	no	The abstract doesn't indicate any empirical evaluation or metrics.	no	No quantifiable outcomes are mentioned in the abstract.	Exclude	The abstract represents a commentary on privacy and legal aspects of mobile health systems, lacking direct clinical applications, empirical research, or synthetic data generation. It fails to meet multiple screening criteria, including original research, quantifiable outcomes, and clinical utility, primarily discussing privacy regulations and emerging technologies from a theoretical perspective.	0.1	Exclude	Exclude	Wrong publication type
Neural network for 3D inertial confinement fusion shell reconstruction from single radiographs	Wolfe B.T., Han Z., Ben-Benjamin J.S., Kline J.L., Montgomery D.S., Merritt E.C., Keiter P.A., Loomis E., Patterson B.M., Kuettner L., Wang Z.	2021	no	The study focuses on technical applications in inertial confinement fusion (ICF) and x-ray radiography, rather than clinical applications.	no	The study deals with radiographic imaging data, which is not tabular data. It specifically mentions "2D radiographs" and "3D volumes."	no	The study uses inertial confinement fusion data, not human clinical or patient-related healthcare information.	yes	Based on the abstract, this appears to be an original research article with novel methodology and results.	yes	The study does involve active creation of synthetic data using AI (CNNs) for training purposes.	maybe	While the study uses synthetic data generation, it's for radiographic images rather than tabular data.	no	The application is in physics/engineering (ICF) rather than clinical applications.	yes	The study includes empirical evaluation with clear metrics for assessing the synthetic data's utility in 3D reconstruction.	yes	The study appears to report quantifiable outcomes related to 3D reconstruction accuracy and comparisons with multiple LOS measurements.	Exclude	The study focuses on technical applications in inertial confinement fusion using radiographic imaging, which fundamentally diverges from clinical research requirements. Critical screening criteria related to clinical applications, human clinical data, and tabular data generation are not met, rendering the paper unsuitable for the systematic review. Despite demonstrating synthetic data generation and empirical evaluation, the research falls outside the specified domain of clinical healthcare applications.	0.1	Exclude	Exclude	No tabular data
Prognostication of zooplankton-driven cholera pathoepidemiological Dynamics: Novel Bayesian-regularized deep NARX neuroarchitecture.	Raja, Muhammad Junaid Ali Asif, Sultan, Adil, Chang, Chuan-Yu, Shu, Chi-Min, Kiani, Adiqa Kausar, Shoaib, Muhammad, Raja, Muhammad Asif Zahoor	2025	no	The paper focuses on mathematical modeling of cholera transmission rather than direct clinical applications. While it has health implications, it's primarily a technical/mathematical modeling study.	no	The study uses time-series data for modeling cholera transmission dynamics, which explicitly violates the criterion of only tabular data generation.	no	While the study involves human population vectors, it doesn't use actual human clinical data or patient-related healthcare information.	yes	This appears to be an original research article with novel methodology and results.	no	While the study generates synthetic data, it uses a "modified Adams-Bashforth-Moulton predictor corrector numerical scheme" rather than AI methods for data generation.	no	The synthetic data generation is done through mathematical modeling rather than AI methods like GANs or VAEs.	no	The study focuses on epidemiological modeling rather than direct clinical applications like diagnosis, prognosis, or treatment planning.	maybe	The study includes empirical evaluation with clear metrics, but these are focused on mathematical modeling performance rather than clinical utility.	maybe	The study reports quantifiable outcomes (mean square error, error analysis), but these are related to model performance rather than clinical metrics.	Exclude	The study focuses on mathematical modeling of cholera transmission using numerical methods, which diverges significantly from the required criteria of AI-generated synthetic clinical data. Multiple screening requirements are not met, including the use of time-series data, lack of AI-driven synthetic data generation, and absence of direct clinical applications. The research represents a technical epidemiological modeling approach rather than a clinical data generation study.	0.1	Exclude	Exclude	No tabular data

Regulatory Challenges in AI/ML-Enabled Medical Devices: A Scoping Review and Conceptual Framework	Kaladharan S., Manayath D., Gopalakrishnan R.	2024	no	The paper focuses on regulatory challenges and policy considerations rather than clinical applications. While it discusses AI/ML in medical devices, it's primarily from a regulatory and policy perspective.	maybe	The abstract doesn't specifically discuss tabular data generation. While it mentions "synthetic data use" as one theme, it's not clear if this refers to tabular data.	no	The paper discusses medical devices in healthcare systems but doesn't specifically work with human clinical data.	no	This is a scoping review article, not an original research article.	no	The paper is a scoping review of existing literature and doesn't involve active creation of synthetic data.	no	While synthetic data is mentioned as a theme, the paper doesn't involve actual generation of synthetic data using AI methods.	no	The focus is on regulatory challenges rather than direct clinical applications.	no	The paper conducts a literature review but doesn't include original empirical evaluation of synthetic data's clinical utility.	no	The paper appears to be qualitative in nature, analyzing regulatory challenges and proposing frameworks rather than reporting quantifiable outcomes.	Exclude	The scoping review focuses on regulatory challenges of AI/ML medical devices, failing to meet multiple screening criteria related to clinical applications, synthetic data generation, and empirical research. Specifically, the paper lacks original data creation, quantifiable outcomes, and direct clinical utility, instead providing a policy-oriented analysis of regulatory landscapes. The study does not involve active synthetic data generation or demonstrate direct clinical applications, rendering it non-compliant with the specified screening requirements.	0.1	Exclude	Exclude	Wrong publication type
Robust deep learning for eye fundus images: Bridging real and synthetic data for enhancing generalization	Oliveira G.C., Rosa G.H., Pedronette D.C.G., Papa J.P., Kumar H., Passos L.A., Kumar D.	2024	yes	The study focuses on clinical applications (AMD detection) rather than purely technical aspects, even though it includes technical elements. The primary goal is clinical diagnosis.	maybe	The study focuses on medical imaging (eye-fundus images) rather than tabular data, which is a clear violation of the core criterion.	yes	The study uses human clinical data from multiple databases (iChallenge-AMD, ODIR-2019, RIADD, STARE).	yes	This appears to be an original research article with novel methodology and results.	yes	The study actively creates synthetic data using AI (StyleGAN2 and other GAN architectures).	yes	The study uses AI methods (specifically GANs) for synthetic data generation.	yes	The study demonstrates direct clinical application in AMD detection and diagnosis.	yes	The study includes thorough empirical evaluation with clear metrics and validation.	yes	The study reports clear quantifiable outcomes (85% accuracy, FID scores, comparison with human experts).	Exclude	The study demonstrates exceptional clinical application in AMD detection using AI-generated synthetic medical images, with robust empirical evaluation and quantifiable outcomes. However, the research fundamentally fails the screening criteria by focusing on medical imaging data rather than tabular data, which represents a critical exclusion factor. Despite meeting most other screening requirements with high precision, the data format disqualifies the study from further consideration.	0.1	Exclude	Exclude	No tabular data
Synthetic data generation in healthcare: A scoping review of reviews on domains, motivations, and future applications.	Rujas, Miguel, Martin Gomez Del Moral Herranz, Rodrigo, Fico, Giuseppe, Merino-Barbancho, Beatriz	2025	no	The paper is a review paper focusing on surveying synthetic data applications rather than presenting a specific clinical application.	maybe	The abstract mentions "unstructured data, particularly images" as predominant, but doesn't specify if tabular data is included, suggesting a broader scope than just tabular data.	maybe	While it covers healthcare domains, the abstract doesn't specify whether the reviewed studies use human clinical data.	no	This is explicitly a review paper ("This review aims to..."), not an original research article.	no	As a review paper, it doesn't involve active creation of synthetic data but rather surveys existing literature.	maybe	While the paper discusses synthetic data generation methods, it's reviewing existing work rather than implementing them.	maybe	While it discusses healthcare domains (Oncology, Neurology, Cardiology), it's reviewing applications rather than demonstrating them directly.	no	The paper doesn't include original empirical evaluation but rather analyzes existing literature.	no	As a review paper, it doesn't report original quantifiable outcomes.	Exclude	This systematic review of synthetic data in healthcare fails key screening criteria by being a review paper rather than original research, lacking direct empirical evaluation, and not actively creating synthetic data. The study provides a comprehensive overview of synthetic data applications across healthcare domains but does not meet the specific requirements for inclusion, such as demonstrating original quantifiable outcomes or involving active data generation.	0.1	Exclude	Exclude	Wrong publication type
Synthetic data in machine learning for medicine and healthcare.	Chen, Richard J, Lu, Ming Y, Chen, Tiffany Y, Williamson, Drew F K, Mahmood, Faisal	2021	no	No abstract available	no	No abstract available	no	No abstract available	no	No abstract available	no	No abstract available	no	No abstract available	no	No abstract available	no	No abstract available	no	No abstract available	Exclude	No abstract available	0.1	Exclude	Exclude	Wrong publication type
