# Supplementary material for: Translation readiness of model-based synthetic tabular data in healthcare: a systematic review and governance audit
Source: J Am Med Inform Assoc. 2026 Jun 18;33(10):2008–28. doi: 10.1093/jamia/ocag102 (PMC13630292; doi:10.1093/jamia/ocag102)
Supplement: Supplementary file 1 [file ocag102_supplementary_data.zip › Supplementary Table 2.pdf]

Supplementary Table 2. Summary of clinical applications and performance outcomes. This table summarises the primary use case and reported performance for the synthetic data in each of the 37 included studies. It details the primary motivation category (augmentation, privacy, in-silico trials, or bias mitigation), the specific clinical purpose, the primary outcome metrics used for evaluation, and a direct comparison of the performance of models trained on synthetic versus real data

Article	Primary motivation category	Specific purpose of synthetic data	Primary clinical outcome metrics	Comparison with real data performance
**Abbaszadeh Bavil Soflaei et al. (2025)**	Augmentation	Advance breast cancer diagnosis by addressing class imbalance.	Precision, recall, F1-score, AUC-ROC, accuracy.	Models trained on CTGAN-balanced data outperformed models trained on the original real data. For example, SVM accuracy was 99.3% on CTGAN-balanced data vs 98.25% on the original dataset; AUC 0.99 vs 0.98.
**Ahmadian et al. (2024)**	Augmentation	Overcome data scarcity in radiomics/radiogenomics models. Augment limited tabular data for robust model training.	AUC for predictive performance. Quality (Chi-square (CS), Basic Statistical Correlation (BSC)).	Synthetic data consistently enhanced AUC (e.g., 9.6% for n=200, 16.7% for n=1000). 94% of runs yielded higher AUC than real data alone.
**Ahmed et al. (2025)**	Augmentation	Address limited labeled data availability. Augment existing datasets to improve ML model training and reduce sampling bias.	Accuracy, precision, recall, F1-score, AUC-ROC.	Original: KNN/LR had 97.37% test accuracy. TVAE synthetic: H2OXGBoost performed best (95.3% test accuracy), approaching original performance. GC Synthetic: Performed poorly.
**Ahn et al. (2025)**	Privacy	Overcome data availability challenges, underrepresentation, and data-sharing regulations. Enable privacy-preserving research and accelerate hepatology research.	Statistical Fidelity (MMD, Wasserstein distance). Clinical Utility (Kaplan-Meier survival curves, MELD score AUC). Privacy Preservation (DCR, Attribute Disclosure Risk, k-Anonymity).	Fidelity: Synthetic dataset "nearly indistinguishable" from real data (MMD=0.002). Clinical Utility: Similar survival patterns and maintained MELD score performance (AUC 0.839 vs. 0.844). Privacy: Robust privacy protection confirmed.
**Araújo et al. (2025)**	Augmentation	To improve prediction of doxorubicin induced cardiomyopathy using ML methods for patients treated for primary breast cancer. Generation of synthetic data sets which mitigate small sample sizes, missing values, and dataset class imbalances while avoiding the need for resource-intensive data collection.	AUROC, sensitivity, specificity, precision, accuracy, balanced accuracy, F1 score.	AUROC: DAS (0.85) outperformed original (0.64), SDV (0.52), SMOTE (0.69), and ADASYN (0.72). The model could not distinguish real from synthetic data (AUROC 0.50).
**Arora and Arora (2023)**	Privacy	Act as a replacement for real data for model training, enabling dataset augmentation and preserving data privacy.	Prediction performance (comparability), Fidelity (histogram analysis, pMSE).	All algorithms trained on synthetic or combined data performed comparably to models trained on real data alone.
**Azizi et al. (2021)**	Privacy	Address data availability challenges due to privacy requirements. Enable broader access to clinical trial data for secondary analyses.	Univariate distributions (KL-divergence), Bivariate relationships, Multivariate Cox models (HR CI overlap).	Main conclusion replicated directionally (HR CI overlap 61% OS, 86% DFS). Survival curves showed close resemblance.
**Bahudian and Valdovinos (2025)**	Augmentation	Demonstrate synthetic data generation and its utility for predicting ventricular functional status and increasing data availability.	Fidelity (histograms, PDF, PCA), Misclassification rates for ventricular dysfunction.	A neural network trained on synthetic data classified ventricular dysfunction in real patients with low misclassification error (9.44-10.99%), showing it was "as useful as real data".
**Christoforou et al. (2025)**	Privacy	Overcome challenges in accessing real-world data due to privacy. Streamline data transfer, ethics approvals, and decrease costs. Ensure privacy and clinical utility.	Privacy (%EM), Clinical behaviour (C-indexes, Hazard Ratios), Feature distribution.	Clinical utility: Similar C-indexes (real 0.701 vs. synthetic 0.699). Every synthetic HR was within the 95% CI of the real HR for 4/5 datasets. Best performer: TVAE.
**D'Amico et al. (2023)**	Augmentation	Generate artificial data to resolve lack of/incomplete information and enable data augmentation. Accelerate translational and clinical research, including trials.	Fidelity (distribution, correlation, PCA), Privacy (IMS analysis, NNDR), Clinical outcomes (survival, trial endpoints).	Fidelity: High fidelity (clinical/genomic similarity scores >90%) and strong privacy protection. Clinical utility: Replicated trial clinical endpoints and enabled development of molecular classifications.
**Eckardt et al. (2024)**	In-silico trials	Mimic clinical trials to create synthetic control cohorts, bypass data sharing issues, and foster research.	Fidelity, Privacy (exact matches), Clinical outcomes (CR rates, EFS, OS, Kaplan-Meier-Divergence).	Outcomes: CR rates showed no significant differences. OS curves were similar for NFlow but deviated for CTAB-GAN+. Survival curves were deemed plausible and usable.
**Fisher et al. (2019)**	In-silico trials	Predict future states and disease progression. Run simulated clinical trials (optimise design, create synthetic control arms).	AUC of logistic regression model to distinguish real vs. synthetic data.	Synthetic data was challenging to distinguish from real data (consistent AUC of ~0.5), indicating high fidelity.
**Foraker et al. (2021)**	Privacy	Enable creation/analysis of clinical data similar to original EHR. Support data sharing for COVID-19 research and accelerate discovery.	Odds Ratios (OR) for admission. Feature importance. Epidemiologic Curves.	Statistical equivalency: Previous work showed statistical equivalency. ORs and Feature Importance values were same or similar. Epidemic Curves showed no significant differences.
**Ganguli et al. (2023)**	Privacy	Address limitations in sharing sensitive patient data. Train new/improve ML models and create an anonymised dataset.	Accuracy, loss, principal component distribution, ML classifier performance (AUC ROC, Accuracy, Precision, Recall, F1 Score).	A small synthetic cohort had statistically comparable ML performance to the full real dataset. Volume-matched synthetic data led to statistically better ML performance than original data.
**Ghareeb et al. (2024)**	Privacy	Mimic real data to enable external validation and global data sharing while preserving privacy. Assure good quality of synthetic data.	Sensitivity, specificity, NPV, AUC, precision, F1 score, Matthews correlation coefficient (MCC).	Models trained on synthetic data achieved comparable AUC, precision, F1 and MCC to those trained on real data.
**Greenberg et al. (2023)**	Privacy	Overcome patient privacy/data ownership concerns in EHR research. Facilitate EHR data acquisition/sharing to characterise spine surgery outcomes.	Descriptive statistics comparing variables. Prediction of 30-day readmissions/complications. Discrimination (AUCs).	Descriptive: Replicated most properties of real data. Predictive: Closely simulated predictive performance, with comparable discrimination (AUCs).
**Guillaudeux et al. (2023)**	Privacy	Protect individual privacy while retaining statistical relevance and utility. Provide patient-centric synthetic data to facilitate data sharing.	Signal maintenance (HR, AUC, F-score). Privacy metrics (DCR, NNDR, local cloaking, hidden rate).	Utility: Avatar showed similar signal maintenance to Synthpop/CT-GAN (e.g., AIDS HR: Avatar 0.40 vs. Original 0.49). Privacy: Individual avatars were indistinguishable other generated avatars.

**Gurcan and Soylu (2024)**	Augmentation	Address class imbalance in healthcare datasets to achieve more balanced results in health analytics and improve cancer prediction.	Accuracy, precision, recall, F1 score, ROC AUC.	GAN-based resampling markedly improved classification performance: the average ROC AUC increased from a baseline of ~0.8276 to >0.9734.
**Hameed and Alamgir (2022)**	Augmentation	Overcome data shortage and patient privacy issues. Eliminate class imbalance and data scarcity to improve ML models' accuracy for clinical practice.	F-beta score, sensitivity, specificity, AUC, PPV, NPV.	Imbalance: SMOTE was more effective than downsampling. GANs/VAE: RF on CTGAN (50:50) had the highest F-beta score (0.7). Overall, synthetic data improved ML accuracy.
**Hsieh et al. (2025)**	Augmentation	Enhance clinical datasets by generating contextual synthetic data to improve model performance and address limitations in incomplete/imbalanced datasets.	Accuracy, MSE, Precision, Recall, F1-score. Feature importance.	Model performance: DALL-M improved model performance (e.g., 25% increase in Precision/Recall for XGBoost). Expert input further increased Precision.
**Jacobson et al. (2023)**	Augmentation	Augment dataset to enable algorithms to generate more stable and reliable predictions, less sensitive to noise.	Similarity of synthetic data distribution, Correlation score, RMSE.	When models trained on synthetic data were evaluated on the real 18-patient dataset, performance decreased, indicating limited generalisation to authentic data.
**Kim et al. (2024)**	Augmentation	Enhance prediction model performance in small patient groups. Preserve statistical/clinical integrity and patient privacy.	Statistical similarity (Hellinger distance), Clinical validity, Reidentification risk, Model performance (Decision Tree, RF, XGBoost).	Training on synthetic data showed the highest performance (DT 0.850, RF 0.836, XGBoost 0.790), outperforming real data alone.
**Koloi et al. (2023)**	Augmentation	Augment clinical research databases with subject shortage. Increase ML model predictive performance.	Quality/reliability of distributions (Kolmogorov-Smirnov (KS) test, Chi-Squared (CS) test).	Distribution reliability: Gaussian Copula and Tabular Preset showed superior reliability (GC: KS 0.90, CS 0.98).
**Kuo et al. (2023)**	Bias mitigation	Mitigate confidentiality issues and overcome mode collapse in GANs to capture imbalanced distributions and support downstream ML algorithms.	Mode collapse mitigation, Realisticness of variables, Patient disclosure risk, Utility for downstream model building.	The extended setup achieved higher diversity and mitigated mode collapse. RL agents on extended synthetic data suggested similar actions to real data, especially for minority groups.
**Laatifi et al. (2023)**	Augmentation	Solve limited dataset availability due to acquisition costs. Enable collaborative research and model building.	Precision, recall, F1 score.	Models trained on synthetic data and tested on real data showed high precision and recall. The Gradient Boosting Classifier demonstrated superior performance relative to other models.
**Lin and Lyu (2025)**	Augmentation	Address class imbalance. Generate high-utility samples for classifier training to improve predictive performance.	Fidelity (Wasserstein Distance, KLD, JS Divergence), Predictive performance (Accuracy, F1-score, ROC-AUC, PR-AUC).	The GAN Balanced dataset consistently had the highest average performance across all metrics, showing significant improvements over the original training data ($p<0.001$ for all).
**Mahmoud et al. (2023)**	Augmentation	Improve data quality for early diagnosis/personalised treatment. Address sparse, imbalanced, or irrelevant attributes.	Visualisations (means, SDs, etc.), Classification performance (accuracy, sensitivity, specificity, etc.).	Classification (Immunotherapy, RF): Synthetic data had higher precision (91% vs 81%), recall (96% vs 82%), and f-measure (93% vs 81%) than real data.
**Nagro (2025)**	Augmentation	Address imbalanced datasets and limited data availability to improve model generalisability, accuracy, and robustness.	Precision, recall, F1-score, AUC-ROC, accuracy.	Model performance: XGBoost/LightGBM accuracy improved from 68-69% to 81% (CTGAN-enhanced). Ensemble stacking with CTGAN achieved 87.29% accuracy.
**Ogwel et al. (2024)**	Augmentation	Increase sample size for stable/reliable predictions of linear growth faltering for children with diarrhoea. Address imbalanced, limited, or privacy-sensitive datasets to overcome biases and ensure model fairness.	Sensitivity, specificity, PPV, NPV, F1 score, AUC, PRAUC.	Performance: Combined (real & synthetic) data resulted in a better prediction performance than real data alone.
**Paladugu et al. (2025)**	Augmentation	Addressing limited availability of data regarding non-invasive indicators of intracranial pressure (ICP) in the context of spaceflight and other austere environments where invasive monitoring would be unsuitable.	Predictive accuracy (Mean Squared Error (MSE)), Fidelity (Kolmogorov-Smirnov tests, Jensen-Shannon divergence).	Predictive accuracy: GAN-generated synthetic data substantially lowered MSE compared to a baseline model trained solely on original data.
**Sharma et al. (2024)**	Augmentation	Augmentation of existing microbiome data using deep learning framework phylaGAN (combination of C-GAN and autoencoder) to overcome small sample sizes, dataset imbalances and cost limitations associated with data collection while achieving complexity comparable to real-world data.	AUC, Sensitivity, Specificity, PPV, NPV.	Augmentation benefit: multiple machine learning methods performed better using phylaGAN-augmented datasets compared to original data.
**Sood et al. (2023)**	Privacy	Model interdependencies between PD risk and prodromal markers. Generate realistic synthetic data for sharing and simulation.	Area under the ROC curve (AUC).	Predictive models trained on synthetic data had ~10% lower AUC than models trained on real data.
**Theodorou et al. (2025)**	Bias mitigation	Address imbalanced datasets and rare outcomes. Enhance algorithmic performance, reduce bias, and ensure equitable treatment.	F1 Score for mortality prediction. Subgroup performance gaps.	Mortality prediction: FairPlay boosted performance up to 21% improvement in F1 Score. Bias reduction: Consistently reduced subgroup performance gaps.
**Titar and Ramanathan (2024)**	In-silico trials	Generative modeling to address high-dimensional, imbalanced tabular data for virtual clinical trial simulations.	Dissimilarity measures for univariate distributions, Probability Density plots.	Satisfactory performance for continuous biomarkers, variable performance for categorical and binary biomarkers. Poorer approximation of categorical variable distibutions with minor group frequencies <10%.
**Topşir et al. (2025)**	Augmentation	Data augmentation to address class imbalance and generate realistic/diverse samples for complex data structures to enable better model generalisation.	Accuracy, Precision, Recall, F1-Score (Macro Avg).	Augmentation impact: Significant improvement in model performance with GANs, MixUp, Oversampling. GANs performance: GANs showed a "particularly robust ability" to generate synthetic data.
**Tran et al. (2023)**	Augmentation	Enlarge the learning dataset and balance classes via Bayesian imputation to develop an optimised prediction tool for 2-year all-cause mortality.	AUC-ROC, accuracy, sensitivity, specificity, PPV, NPV.	Creating a synthetic population and using only seven variables improved the optimised model's AUC to 0.81 compared with the base models.

**Yang et al. (2024)**	Augmentation	Generate feature-aligned/category-aware EHR data to improve performance of classifiers.	Distribution Similarity Analysis, Clinical evaluation, Model results for prediction tasks (accuracy, F1 score, AUC).	Classifier performance: A balanced synthetic dataset was beneficial for robust classifier performance. Experienced surgeons perceived synthetic EMRs to closely resemble real EMRs.
