# Supplementary material for: Translation readiness of model-based synthetic tabular data in healthcare: a systematic review and governance audit
Source: J Am Med Inform Assoc. 2026 Jun 18;33(10):2008–28. doi: 10.1093/jamia/ocag102 (PMC13630292; doi:10.1093/jamia/ocag102)
Supplement: Supplementary file 1 [file ocag102_supplementary_data.zip › Supplementary Table 3.pdf]

Supplementary Table 3. Temporal distribution and pre-2019 screening exclusions. This table reports the publication years of included studies and the reasons records from 2010-2018 were excluded at screening

Year	Included studies
2019	1
2020	0
2021	2
2022	1
2023	12
2024	9
2025	12

Category	Count
2010-2018 screened records	39
Excluded for no tabular data	30
Excluded for no synthetic data generation	8
Excluded for no AI generation	1
