# Supplementary material for: Translation readiness of model-based synthetic tabular data in healthcare: a systematic review and governance audit
Source: J Am Med Inform Assoc. 2026 Jun 18;33(10):2008–28. doi: 10.1093/jamia/ocag102 (PMC13630292; doi:10.1093/jamia/ocag102)
Supplement: Supplementary file 1 [file ocag102_supplementary_data.zip › Supplementary Table 4.pdf]

Supplementary Table 4. TRUST-SD derivation matrix. This table maps each TRUST-SD reporting item to the corresponding reporting gap observed in the review, the supporting rationale base, and the reason the item is needed.

Item	TRUST-SD domain	Reporting requirement	Observed gap in this review	Supporting source / rationale base	Why this item is needed
1.1	Context and intended use	Clinical question and target population	Clinical purposes were usually stated, but user, workflow, and decision-threshold details were often sparse.	TRIPOD+AI; IMDRF SaMD clinical evaluation; review findings	Readers need to know the clinical decision context before judging whether utility, privacy, and regulatory evidence are proportionate.
1.2	Context and intended use	Intended application: decision support, trial design, service planning, or quality improvement	Applications were heterogeneous and often indirect.	Review taxonomy; FDA MDDT context-of-use logic	Application category determines the level of validation and governance evidence expected.
1.3	Context and intended use	Deployment paradigm: no-release or delayed-release	Most studies did not state whether synthetic data would replace real-data access or only accelerate early analysis.	van Breugel et al.; review findings	No-release settings require stronger privacy and fidelity claims than delayed-release workflows validated later on real data.
1.4	Context and intended use	Intended end-users and decision context	Few studies specified who would use the downstream output or how it would affect care.	IMDRF SaMD clinical evaluation; TRIPOD+AI; review findings	User and workflow specificity are needed for clinical translation and risk assessment.
2.1	Source data transparency	Real-data source, setting, and time period	Source datasets varied widely, and generalisability was often limited by single-centre or narrow cohorts.	TRIPOD+AI; review findings	Source context determines transportability and risk of inherited bias.
2.2	Source data transparency	Sample size, missingness, and preprocessing steps	Several studies used small, incomplete, imbalanced, or heavily preprocessed datasets.	TRIPOD+AI; review findings	Synthetic data quality depends directly on source-data preparation.
2.3	Source data transparency	Known biases, limitations, or underrepresented groups in source data	Bias propagation and minority-group underrepresentation were repeatedly reported limitations.	van Breugel et al.; review findings	Synthetic data can amplify source-data limitations if these are not documented.
2.4	Source data transparency	Ethical approval and source-data governance	Ethics and governance statements were inconsistent and often unrelated to synthetic-data release assumptions.	HHS HIPAA de-identification guidance; GDPR/data protection principles	Source-data governance remains relevant even when the output is synthetic.
3.1	Generation method	Generator architecture	Generation methods ranged from GANs and VAEs to Bayesian networks, diffusion models, LLMs, and proprietary systems.	Review findings	Architecture affects fidelity, privacy risk, reproducibility, and failure modes.
3.2	Generation method	Training regime, hyperparameters, and software/library used	Technical reporting was heterogeneous; some studies used established libraries while others gave limited implementation detail.	TRIPOD+AI; review findings	Reproducibility requires enough detail to recreate the synthetic-data pipeline.
3.3	Generation method	Privacy mechanism: none, operational controls, or formal model	Formal privacy models were uncommon (9/37).	HHS HIPAA de-identification guidance; Zhang et al.; van Breugel et al.	Privacy claims require explicit mechanisms and attack-model assumptions.
3.4	Generation method	Synthetic dataset size and post-processing	Synthetic-to-real ratios varied markedly and could affect overfitting, utility, and privacy.	Review findings	Dataset scale and post-processing influence both downstream performance and disclosure risk.
4.1	Utility evaluation	Distributional fidelity metrics	Most utility assessments used distributional or correlation similarity metrics.	Kaabachi et al.; review findings	Distributional fidelity is useful but insufficient alone.
4.2	Utility evaluation	Downstream task utility on held-out real data	Many studies reported downstream performance, but clinical validation was usually internal.	TRIPOD+AI; review findings	Synthetic data should be evaluated for the task it is intended to support.
4.3	Utility evaluation	Validation depth: internal, external, or prospective	34/37 studies relied on internal validation; none reported prospective evaluation in routine care.	IMDRF SaMD clinical evaluation; review findings	External and prospective validation are central to translation readiness.
4.4	Utility evaluation	Calibration assessment where applicable	Calibration was rarely foregrounded despite decision-support use cases.	TRIPOD+AI	Clinical prediction models require calibration evidence, not only discrimination.
5.1	Privacy evaluation	Re-identification risk assessment	Privacy reporting was heterogeneous; many studies had no explicit privacy mechanism.	HHS HIPAA de-identification guidance; Zhang et al.; review findings	Privacy risk is context-dependent and should be evaluated rather than assumed.
5.2	Privacy evaluation	Membership inference attack testing where feasible	Attack-based privacy testing was infrequent.	Zhang et al.; van Breugel et al.	Generative models can leak training-set information even when direct identifiers are absent.
5.3	Privacy evaluation	Governance position under applicable law and residual risk	Few studies linked privacy claims to legal/governance status.	GDPR/EHDS context; HHS HIPAA guidance	Synthetic data may or may not be treated as anonymised/de-identified depending on context and residual risk.
6.1	Fairness evaluation	Subgroup-specific fidelity	Most studies did not assess whether synthetic data preserved subgroup distributions.	van Breugel et al.; review findings	Mode collapse can underrepresent minority groups.
6.2	Fairness evaluation	Subgroup-specific downstream utility	Only 6/37 reported explicit subgroup or fairness metrics.	TRIPOD+AI; review findings	Overall utility can mask subgroup harms.
6.3	Fairness evaluation	Mode collapse or mode invention assessment	Few studies explicitly assessed minority-subgroup omission or implausible profile generation.	van Breugel et al.; review findings	Synthetic-data failures can affect equity and clinical safety.
7.1	Regulatory and governance alignment	Applicable data protection framework	Data-protection law was mentioned inconsistently.	GDPR/EHDS; HHS HIPAA guidance	Regulatory context affects release and reuse conditions.
7.2	Regulatory and governance alignment	Medical-device or software-regulation relevance where applicable	Explicit SaMD/device-regulation discussion was rare.	IMDRF SaMD key definitions; IMDRF SaMD clinical evaluation	Synthetic-data pipelines that inform diagnostic/prognostic software may require device-regulatory evidence depending on intended use and jurisdiction.
7.3	Regulatory and governance alignment	Use in regulatory-meaningful evidence, including synthetic/external control arms	Several studies proposed synthetic controls, but regulatory alignment was limited.	FDA MDDT guidance; RWE guidance; review findings	Synthetic controls require context-of-use-specific evidence acceptable to regulators.
